# "It’s Feeding the Beast": Lessons for Governance of Public Health Surveillance and Response From an Australian Case Study Analysis

**DOI:** 10.34172/ijhpm.8605

**Published:** 2025-05-18

**Authors:** Stephanie M. Topp, Alexandra Edelman, Thu Nguyen, Emma S. McBryde, Sue Devine, Tammy Allen, Jeffrey Warner, Julie Mudd, Paul F. Horwood

**Affiliations:** ^1^Public Health and Tropical Medicine, College of Medicine and Dentistry, James Cook University, Townsville, QLD, Australia.; ^2^Menzies School of Health Research, Charles Darwin University, Darwin, NT, Australia.; ^3^Australian Institute of Tropical Health and Medicine, James Cook University, Townsville, QLD, Australia.; ^4^College of Medicine and Dentistry, James Cook University, Townsville, QLD, Australia.

**Keywords:** Public Health, Health System, Communicable Disease Control, Surveillance and Response, Governance, Queensland

## Abstract

**Background:**

Public health is a core governmental responsibility, with ministries or departments of health responsible for setting and ensuring adherence to standards, managing performance and instituting reforms as required. Although North Queensland (NQ), Australia has a well-developed health infrastructure, the COVID-19 pandemic exposed significant vulnerabilities in its public health surveillance and response system. Globally, research has highlighted how human and cultural elements ("system software") influence the effectiveness of infrastructure, governance, and data systems ("system hardware"). This study examines the interaction between these elements to examine specific governance challenges and opportunities for strengthening communicable disease surveillance and response in NQ.

**Methods:**

Using an embedded case study design, we analysed four disease units—COVID-19, tuberculosis (TB), arboviruses, and sexually transmitted infections (STIs)—through interviews (n=47), document review, and observations across NQ health services (October 2020–December 2021). Data were mapped against Sheikh and colleagues’ hardware-software framework to examine the nature of governance bottlenecks in this region of northern Australia.

**Results:**

Two key governance challenges emerged: (1) Accountability deficits—Hospital and Health Services (HHSs) lacked clear reporting or performance monitoring systems within Queensland’s devolved health service governance model, contributing to inconsistent prioritisation of resourcing for communicable disease functions by health service leadership. Within HHSs, public health units (PHUs) faced systemic underfunding, with prevention services accounting for as little as 0.1% of some health service budgets. (2) Data governance failures—Fragmented, siloed data systems, restrictive data-sharing norms, and risk-averse culture hindered coordinated surveillance and response efforts. Weak interoperability and mistrust in data-sharing partnerships further compromised system effectiveness.

**Conclusion:**

This study highlights how political, normative, and structural factors shape public health performance alongside the more commonly assessed functional and technical dimensions. Findings suggest the need to improve performance monitoring systems, leadership, and data governance to build an effective, accountable, and data-driven surveillance and response system in NQ.

## Background

**Key Messages**

**Implications for policy makers**

- In Australia, the public health surveillance and response systems for communicable disease are orchestrated through a vertical integration and coordination. At different levels of this vertical governance of communicable disease, the interplay between the systems hardware (material elements) and software (human and cultural elements) is crucial to its efficacy.
- The current lack of performance monitoring systems, organisational structure, leadership, and the sub-optimal systems of data collation, governance and use are contributing to a stressed communicable disease surveillance and response systems in North Queensland (NQ).
- Communicable disease surveillance and response systems in NQ should improve performance monitoring systems, organisational structure, leadership, and data governance to avoid stretching to its limits and ameliorate its efficacy.

**Implications for the public**

Communicable disease surveillance and response systems are designed to monitor, detect, and respond to health threats and are influenced by a myriad of factors. As a result, governance of those systems is complex and multi-faceted, involving the interplay between health systems hardware and software. Understanding the interconnectedness between these two elements is useful to identify opportunities to strengthening infectious disease preparedness. The findings of this research identify two key features of communicable disease surveillance and response systems in North Queensland (NQ) that affect their performance. The findings suggest that efforts be made in strengthening accountability and data systems to enhance future outcomes, which will benefit the Australian public.

The governance of public health functions is essential for delivering high-quality preventive, promotive and protective services, responding effectively to health risks, enabling equitable access to healthcare, and facilitating public trust and engagement.^1^ As in any other areas of the health system, good governance of public health should ensure that services are delivered optimally and that resources are used judiciously to achieve the best population health outcomes.^2^ Recent global health challenges relating to communicable disease control for COVID-19, among others, have demonstrated how the structure and agility of public health governance can significantly impact disease containment and management, in turn shaping population health outcomes.^3^

 In Australia, the efficacy of public health governance is vested in and dependent on effective vertical integration and coordination.^4^ The public health surveillance and response systems for communicable diseases are orchestrated through a centralised framework, underpinned by the Australian Government Department of Health. This national level sets uniform health policies and coordinates nationwide disease surveillance and response, including via the National Notifiable Diseases Surveillance System which facilitates the collection and analysis of data on a range of diseases mandated for reporting.^5^

 At the state level, state and territory jurisdictions operate under these broader national health guidelines while implementing and tailoring them to fit with local conditions and needs.^4^ In the state of Queensland, for example, public health governance is structured around the Queensland Health Department and its affiliated services, which operate under both state legislation, such as the Public Health Act 2005 (Qld)^6^ and the Public Health Regulation 2018 (Qld),^7^ and in coordination with the Australian Federal Government. Queensland Health (the state Department of Health) is tasked with delivering, managing, and ensuring the quality and accessibility of hospital, healthcare services, and public health functions across the state. It operates through a network of 16 independently managed Hospital and Health Services (HHSs), each of which is a statutory body governed by a Hospital and Health Board, accountable to the state government for local service delivery and public health functions.^8^

 While this multi-level approach is meant to ensure a comprehensive and agile response to public health threats including communicable diseases, the interaction between different system components at different levels in the vertical governance of communicable diseases is a political and complex process, not always responding optimally to public health threats. Despite a body of research on essential public health functions^9^ and growing recognition of the need for systems-thinking to strengthen health systems more broadly,^10-12^ less is known empirically and theoretically about the complex interactions among (national and sub-national) institutions and their consequential effects on public health functions, especially with regards to communicable disease surveillance and response. This study fills this gap by building on Sheikh and colleagues’ “hardware-software” framework.^13^ This framework recognizes that, in any health system, the hardware component comprises the tangible, material components of a system – such as infrastructure, health workers, medicines, and commodities; and the software component refers to the interests, relationships and practice-based norms of the human stakeholders whose decisions and actions bring the health system to life.^13^ In this framework, Sheikh et al. stresses that any system, including communicable disease surveillance and response system, relies on the interplay between the hardware and software.^13^

 Guided by the Sheikh and colleagues’ framework, this study examines the function of the software, and how it works together with the hardware in the communicable disease surveillance and response system in north and far North Queensland (NQ) (inclusive of the Torres Strait Islands) to identify opportunities for improvement. Given the vertical governance of public health in NQ, the paper takes a further step in examining the intersections of state and local public health systems, particularly how localised (sub-state) public health surveillance and response systems are structured. As the COVID-19 experience demonstrated, understanding *how* surveillance and response functions are governed and coordinated at different levels is a necessary first step to identifying opportunities for strengthening overall preparedness for infectious disease outbreaks and future pandemics.^14^

## Methods

###  Study Design and Data Collection

 We adopted an embedded single case study design with the case defined as the surveillance and response systems in the broader NQ region; and four diseases or groups of diseases as case units to provide an anchor for exploring more specifically how surveillance and response functions are delivered across different areas: COVID-19, mosquito-transmitted arboviruses and malaria, sexually transmitted infections and blood-borne viruses (STI/BBVs), and tuberculosis (TB). Selection of these case units was based on their relevance to the NQ context (ie, higher prevalence and/or risk of disease compared with southern and metropolitan areas of Australia) and aimed to reflect diverse organisational, biological, regulatory, and political characteristics. For the four case units, we followed a similar process of stakeholder and process mapping; generation of detailed case unit reports; member checking and, then at the whole of case study level, comparative analysis linking to the broader policy context.

 Data collection occurred between October 2020 and December 2021, spanning three phases. The first phase entailed creating maps of surveillance and response processes and stakeholders primarily through the review of policy documents and other official organisational documentation. Interviews with three key informants with senior roles in the NQ public health system were also conducted to enhance the lead investigators’ understanding of important contextual details.

 The second phase comprised key informant interviews (n = 47) to produce comprehensive reports for each embedded case unit. To identify potential interviewees, we utilised organisational charts from public websites along with the investigator team’s network. Criterion and snowball sampling, which are types of purposive sampling techniques used in qualitative research, were used to select interview participants based on their roles within and expertise on healthcare delivery and planning entities in NQ.^15^ They included a diverse range of professions such as clinical staff (including infectious disease and emergency department physicians); public health staff (including nurses, environmental health officers, public health medical officers, and epidemiologists); personnel involved in pathology and scientific services; and mid-level managers including medical superintendents. The snowballing techniques helped identify additional interviewees in the data collection process. Among the total, 33 (72%) were Queensland Health employees working variously in public health and affiliated units, hospitals, or administration; 5 (11%) were from the Aboriginal and Torres Strait Islander Community Controlled Health Organisation(s) (ACCHO) sector; 7 (15%) were academics or researchers, and one participant (2%) was retired. Seventeen (36%) of participants were female and 5 (11%) were Aboriginal and/or Torres Strait Islander. The decision to stop interviews at 47 was guided by the concept of theoretical saturation, where additional interviews no longer yielded new insights pertinent to the study’s research questions.

 Interviews were in-depth and semi-structured and conducted by one or two investigators (ST and AE) with the aid of an interview guide slightly adapted for each case unit (Supplementary file 1). Potential participants were initially contacted via email with a summary of the study and an invitation to participate. Those who agreed to take part coordinated with the research team to schedule an interview at a convenient time and location, with approximately one-quarter of interviews conducted virtually. Participants provided written consent for audio-recording and interviews took 30 minutes on average. Participants were highly engaged and reflected on their direct operational roles within the public health system. Many held leadership, clinical, or administrative responsibilities related to communicable disease surveillance and response, providing rich, contextually grounded insights. Their willingness to participate and share detailed accounts of system challenges and practices was notable and indicative of the relevance of the research to their work.

 A third phase of data collection involved three instances of (pre-approved) unstructured observations in three NQ HHSs public health offices. Observations were made to better understand the primary activities and relationships pertinent to communicable disease surveillance and response within the primary public health decision-making sites in NQ. This entailed researchers (AE and ST) attending regular meetings and observing busy office environments for several days at each site, as well as shadowing senior staff in their typical work routines.

###  Analysis

 Interview transcripts from both phases were processed verbatim and coded inductively using NVivo [QSR]. To form the coding framework, two investigators (AE and ST) coded a set of four transcripts (one for each unit of study), then convened to discuss their strategies and decide on a suitable analysis structure. To produce initial case-unit reports (n = 4) inductive coding was undertaken, overarching themes inductively derived and refined deductively with reference to the health system and governance theories described above. Data derived from document analysis and observation were utilised to generate preliminary process and stakeholder maps, and to aid the analysis of the interview data via triangulation. Also, during this phase, provisional findings from each case were discussed with individuals holding executive roles within healthcare service organisations and critical policy roles within government units and disseminated at local scientific forums.^16-18^ Comparative analysis of the results across the four case units was then focused on discerning factors influencing the governance of the wider communicable disease surveillance and response system in the region.^19^ More in-depth reporting on individual (unit-specific) issues is the focus of a separate analysis.

 The Townsville HHS Human Research Ethics Committee (HREC) granted ethics approval (HREC/2019/QTHS/59811) and reciprocal approval was received from James Cook University HREC on 15 January 2020. Site-specific governance approval was secured from the Townsville HHS, Cairns and Hinterland HHS, Torres and Cape. Transcripts from participant interviews are securely stored on password-protected computers, in accordance with HREC and university ethical requirements. Due to the sensitive nature of the topics discussed in the interviews, the raw data cannot be publicly shared, but anonymised excerpts are provided within the manuscript, and further information can be requested from the corresponding author with appropriate ethical considerations. Copies of publicly available documents used in the analysis were imported into qualitative analysis software, with access dates recorded. Public Health budget allocations for individual HHS budgets were extracted from publicly available Service Agreements and supplied in Supplementary file 2.

## Results

 With Sheikh’s hardware-software framework in mind, this section critically analyses the interaction between hardware and software components through a focus on two key themes identified during cross-unit analysis. The themes were identified as key challenges to the effective governance of communicable disease surveillance and response systems in all four case-units and across NQ. Below, we outline each of these in turn and provide illustrative examples from the case unit findings.

###  Whose Eye Is on the Public Health Ball? The Accountability Challenge

 The first theme relates to weak lines of accountability for public health functions through the Queensland state health system, and their impact on stewardship and resourcing of communicable disease surveillance and control. Where relevant, we include discussion of accountability for *general* public health functions as well as examples of communicable disease surveillance and response examined in the case study and embedded case units.

####  Performance Management for Public Health

 Queensland HHSs and their embedded public health units (PHUs) are at the centre of Queensland’s public health system. These 16 state-funded statutory entities are formed under the *Hospital and Health Board Act 2011.*^20^ The Act is operationalised via Service Level Agreements which establish a legal requirement for HHSs, via the PHU, to provide specialist communicable disease epidemiology and surveillance, disease prevention and control services, and a range of other public health functions.^21^

 Notwithstanding that Service Level Agreements do establish HHSs’ legal accountability for public health functions, there is little detail in those agreements to support operationalisation of public health activity. The key performance and outcome indicators for each HHS, for example (Table 1), focus primarily on hospital performance and related functions. Only a handful of thematically disparate public health indicators are included (eg, oral health, smoking cessation, adolescent vaccination) and none focus on communicable disease surveillance or response. Despite a legal requirement to deliver on a broad range of public health functions, the monitoring framework for HHS performance provides only a very limited basis for evaluating delivery of a full scope of public health activities in this area.

**Table 1:** HHS Performance Measures Extracted From Service Level Agreements for five North Queensland Hospital and Health Services 2019/2020-2021/2022

**Performance Domain**	**Key Performance Indicators**	**Safety and Quality Markers**	**Outcome Indicators**
Safe	Hospital acquired complications Emergency length of stay Number of emergency department stays greater than 24 hours Emergency department wait time by triage category Rate of face-to-face community follow up within 1-7 days of discharge from an acute mental health inpatient unit	Number of wholly preventable sentinel events Hospital standardised mortality ratio Healthcare-associated *Staphylococcus aureus* (including MRSA) bacteraemia Severity Assessment Code closure rates Unplanned readmission rates	Rate of seclusion events per 1000 acute mental health admitted patient days Rate of absent without approval from acute mental health inpatient care
Timely	Patient off stretcher time Elective surgery (patients treated within clinically recommended time) Specialist outpatients (patients offered appointments within clinically recommended time) Gastrointestinal endoscopy (patients treated within clinically recommended time) Access to oral health services (patient waiting times)	-	Reperfusion therapy for acute ischaemic stroke Access to emergency dental care
Equitable	Potentially preventable hospitalisations – First Nations People Telehealth utilisation rates	-	First Nations people representation in the workforce Completed general courses of oral healthcare for first Nations people adult patients Low birthweight
Efficient	Forecast operating position Average sustainable Queensland Health FTE Capital expenditure performance	-	
Effective	-	-	Uptake of the smoking cessation clinical pathway for public hospital inpatients and dental clients Potentially preventable hospitalisations – diabetes and non-diabetes complications % Oral health activity which is preventive Cardiac rehabilitation Adolescent vaccinations administered via the School Immunisation Program
Patient centred	Proportion of mental health service episodes with a documented care plan Proportion of beds vacated by 11 am	-	Complaints resolved within 35 calendar days Advanced care planning

Abbreviations: HHS, Hospital and Health Service; MRSA, Methicillin-resistant *Staphylococcus aureus*; FTE, full time equivalent.

 “*So what happens is [HHSs] get the bucket of money and the staff and the profile and the delegations and all of the authority on behalf of their customer [but] there is no specifics in terms of the deliverables and there’s no mechanisms for accountability” *(Interviewee 9).

####  Organisational Positioning of Public Health Units

 In the absence of a robust mechanism for state-to-HHS monitoring of public health performance, the organisational position of *PHUs* within each HHS, and the profile and capabilities of HHS leadership vis-à-vis public health, take on particular significance, as two factors likely to influence the internal accountability for public health functions. Prior to the establishment of the HHSs in 2012, PHUs were semi-autonomous organisational units within the state health system with a direct budget allocation from the state government. At that time the NQ region had two PHUs based in Cairns and Townsville respectively, which operated a hub-and-spoke model across the geographic area now covered by five northern HHSs. In 2011, the two autonomous PHUs were repositioned *within* the newly created Cairns & Hinterland, and Townsville HHSs respectively.^22^ Although still reporting to the central Communicable Disease Branch of Queensland Health, these embedded PHUs were now operationally part of their respective HHSs. It is worth noting that the budgets for PHUs were now under the discretion of the executive leadership of these statutory, Board-governed HHSs.

####  Leadership of Public Health Functions

 The organisational shift of PHUs from semi-autonomous entities to embedded within HHSs, while arguably located closer to the populations of focus, had several significant adverse effects on public health and communicable disease surveillance and response systems. Multiple participants described the HHS executives to whom they reported (in the northern regions if not beyond) as having a dominant professional focus on clinical acute services and hospital performance. Understanding of, or experience in, planning and service delivery for population and public health, as described below, was limited.

 “*Population health […] – that is an absolute struggle to get a conversation about. Because the acute sector, we manage reported cases – who comes to the ED [Executive Director], and we manage how long it takes to treat you, then you go home. So the system doesn’t in itself look at the big picture” *(Int 45).

 “*We just are in the dark ages [regarding public health in NQ] because there isn’t that resident expertise. […] they can be the best [HHS] administrators in the world, but […] we don’t have anybody in leadership that has public health [experience]” *(Int 39).

 In an example from the COVID-19 case unit, some participants described having to work hard to convince HHS executives that PHUs had a role to play in planning COVID-19 contact tracing and surveillance, owing to the executives’ limited understanding of the public health legal and technical arrangements:

 “*They [HHS Executive] were just ignoring the PHU. They thought that they were going to run COVID-19 and contact tracing and surveillance and all the responses. So [the PHU] had to write to the CEO [Chief Executive Officer] and tell them [the HHS risked] breaking the law [regarding who was] an authorised contact tracer” *(Int 15).

 Lack of attention or priority to public health functions also translated into (deepening) resourcing challenges. Prior to 2011, PHUs were allocated a direct budget from Queensland Health. Once embedded within the HHSs, PHUs were dependent on HHS executives for this same budget. While acknowledging broader system-wide budget cuts that affected all health services, participants also described a perception of shrinking budgets for public health specifically relative to total HHS funding, an impression influenced by the lack transparency in the budgeting processes:

 “*It’s done by the CEO and chief financial officer. That’s it, they just kind of go down there, chat, come back, and it’s very ‘secret squirrel.’ Then [our PHU is] just given our budget” *(Int 27).

 Detailed HHS budgets were not able to be accessed as part of this study. However, prospective allocations by purchasing category were extracted from publicly accessible Service Level Agreements (Table 2) and indicate the small proportions of HHS spending on public health activities. In 2019/2020, for example, public health excluding COVID-19 specific activities, represented only between 0.4% and 2.1% of total HHS (non-capital) allocations across the five northern HHSs. This increased slightly to between 1.8% and 3.1% of total HHS allocations in 2024/2025.

**Table 2:** Prospective Allocations to Identifiable Public Health Activities^a^ in Five North Queensland HHS Service Level Agreements: 2019-2024^b^

**Cairns and Hinterland HHS**	**2019/2020**	**2020/2021**	**2022/2023**	**2024/2025**
Prevention services – public health	20 863 637	22 013 172	23 213 334	23 333 500
Other specified public health allocations (eg, environmental health/oral health/breast screen/child health checks)	1 142 565	224 574	19 090 682	21 558 724
COVID-19 specific allocations	6 150 051	4 181 831	0	0
HHS total budget	1 025 880 042	1 025 550 523	1 179 097 839	1 469 088 459
Public health excl. COVID-19 allocations as % of total	2.1%	2.2%	3.6%	3.1%
Public Health incl. COVID-19 allocations as % of total	2.7%	2.6%	3.6%	3.1%
**Mackay HHS**	**2019/2020**	**2020/2021**	**2022/2023**	**2024/2025**
Prevention services – public health	2 191 963	2 126 380	2 060 171	2 033 543
Other specified public health allocations (eg, environmental health/oral health/breast screen/child health checks)	1 083	8 099	9 318 214	11 643 693
COVID-19 specific allocations	3 971 354	570 000	0	0
HHS total budget	484 904 604	484 174 163	562 167 967	707 182 424
Public health excl. COVID-19 allocations as % of total	0.5%	0.4%	2.0%	1.9%
Public Health incl. COVID-19 allocations as % of total	1.3%	0.6%	2.0%	1.9%
**North West HHS**	**2019/2020**	**2020/2021**	**2022/2023**	**2024/2025**
Prevention services – public health	100 000	30 574	1 060 425	812 600
Other specified public health allocations (eg, environmental health/oral health/breast screen/child health checks)	780 050	808 929	2 563 460	4 113 516
COVID-19 specific allocations	1 890 683	3 100 000	0	0
HHS total budget	199 849 620	197 749 448	220 153 165	284 670 967
Public health excl. COVID-19 allocations as % of total	0.4%	0.4%	1.6%	1.7%
Public Health incl. COVID-19 allocations as % of total	1.4%	2.0%	1.6%	1.7%
**Torres and Cape HHS**	**2019/2020**	**2020/2021**	**2022/2023**	**2024/2025**
Prevention services – public health	985 560	1 137 275	2 896 381	3 997 419
Other specified public health allocations (eg, environmental health/oral health/breast screen/child health checks)	177 404	178 954	4 676 855	6 408 489
COVID-19 specific allocations	2 532 176	3 031 375	0	0
HHS total budget	239 521 404	238 742 427	276 888 677	373 928 650
Public health excl. COVID-19 allocations as % of total	0.5%	0.6%	2.7%	2.8%
Public Health incl. COVID-19 allocations as % of total	1.5%	1.8%	2.7%	2.8%
**Townsville HHS**	**2019/2020**	**2020/2021**	**2022/2023**	**2024/2025**
Prevention services – public health	8 969 240	8 273 478	8 486 961	10 387 812
Other specified public health allocations (eg, environmental health/oral health/breast screen/child health checks)	13 617	39 620	14 267 942	16 537 234
COVID-19 specific allocations	3 433 586	2 720 989	0	0
HHS total budget	1 077 360 823	1 082 165 639	1 202 952 724	1 504 042 888
Public health excl. COVID-19 allocations as % of total	0.8%	0.8%	1.9%	1.8%
Public Health incl. COVID-19 allocations as % of total	1.2%	1.0%	1.9%	1.8%

Abbreviation: HHS, Hospital and Health Service. ^a^ Table includes the major Public Health allocation category of *Preventive Services – Public Health*, which funds HHS Public Health Unit activities. Additional specific line-item allocations of public health relevance such as *Environmental Health*, *Oral Health*, *Child Checks*, *Breast Screening* are included where identified. We could not identify if any partial allocations made under non-public health budget line items and recognise the potential for underestimates in this area. We included all COVID-19 spending (some of which was not public-health focused) in 2019/2020 and 2020/2021. ^b^ All figures in AU$. Data drawn from: 2019/2020-2021/2022 Service Agreements Table 4 (Prevention Services – Public Health/Total Allocations) and Table 6 (Other Specific Funding – Environmental Health); and 2022/2023-2023/2024 Service Agreements Table 4 (HHS Total Funding Allocation by Funding Source 2022/2023 and 2023/2024) and Table 6 (Discretely Funded Programs). All agreements to be found at Supplementary file 2.

 Many PHU functions were enabled or boosted via short term project funding from the state or federal governments. During the 2019/2020 period, for instance, designated (project specific) COVID-19 allocations represented between 0.3% and 1.1% of total HHS (non-capital) allocations, and special funding was also received for rheumatic heart disease surveillance, vector control activities in the Torres Strait, and the *North Queensland Aboriginal and Torres Strait Islander STI Action Plan 2016-2021 *among others.^23^ Yet reliance on these siloed and fixed-term funding streams complicated, rather than alleviated, planning for public health activities including communicable disease surveillance and response activities.

 The challenge of being accountable for legally mandated public health functions in a geographically vast region with a dispersed population and high needs with limited resources, was a recurring theme in interviews for all case units. In an example from the STI/BBV case unit, despite significant attention devoted by sexual health teams in the two large Cairns and Townsville PHUs, and an injection of state government funds via the *North Queensland Aboriginal and Torres Strait Islander STI Action Plan 2016-2021*, various participants described the insufficiency of resources and/or region-wide coordination mechanisms to sustainably address the high burden of disease. Some, although not all, argued that the prevailing hospital-focus of HHS leadership made it difficult to achieve investment in longer-term public health strategies that lay outside the realm of curative care – such as proactive engagement with communities to improve theacceptability and coverage of STI testing; or effective interventions to address the upstream social, cultural, and environmental determinants of STI/BBV incidence.

 “*[Individual interactions] make people feel really good because they’re used to that clinical way of working… [But], treating five people, doing a one-and-a-half-hour sexual history on five people, is not going to control your outbreak” *(Int 28).

 Overall, despite HHSs’ legal responsibility to deliver public health services, the case units demonstrated a limited basis for state-level performance monitoring of HHS public health functions, and a lack of “soft” (leadership and norms-based) mechanisms to support HHS’ internal accountability for strategic public health activity. While the PHUs worked hard to deliver mandated services, the limited public health experience of HHS executive-level leadership and patchy appreciation of the need to anticipate and respond to risk (rather than curative service demand) contributed to overall low priority given to resourcing or prioritising of public health within the broader HHS operations.

###  Data Compatibility and Data Use – the Planning Challenge

 Another theme identified in cross unit analysis related to chronic information and planning challenges arising from three intersecting issues with data. The first was multiple, siloed, and often inaccessible data systems; the second was data governance systems and their impact on data sharing across work units and organisations; and the third (related) issue was the culture of data use for public health within the health services.

####  Surveillance and Data Systems

 Across the four case units, challenges of data quality, fragmentation and interoperability, and their impact on public health response capabilities were recurring themes. While public health data is collected by multiple entities in Queensland, including hospitals, primary care providers (government and Aboriginal Community Controlled service sector) and public health agencies, at the time of writing there was no centralised system for managing, curating, or sharing this data in support of public health decision-making. The proliferation of different data collection systems (Table 3 provides a summary of the major ones) and databases contributed to challenges relating to duplicate but unlinked patient identifiers and non-standard data definitions; and as a result, poor inter-operability. Multiple participants in different roles reflected on the difficulty of gaining a comprehensive understanding of population health status, which was necessary to plan public health interventions in the sensitive cross-border region of the Torres Strait where monitoring of TB and multi-drug resistant TB is imperative:

 “*So the pathology results are in a different system to the health record system in the hospital. Different to the paper notes in the hospital. Different to [Emergency Department Encounter Summary] EDES. So I don’t know how we don’t kill people as a matter of routine” *(Int 34).

 “*Data linkage is just an example of how dysfunctional all of the things are, right? We don’t need data linkage if we had a functional system. So, it’s getting - these days, getting data out of [ Aus Lab] to do stuff is very difficult” *(Int 1).

**Table 3:** Illustrative List of Data Systems Supporting Communicable Disease Surveillance and Response Functions in North Queensland^a^

**Data System Name**	**Data System Description**
CDSS	Aims to track and control the spread of communicable diseases within the state. CDSS collects data from multiple sources, including laboratories, hospitals, and healthcare providers.
NOCS	Focuses on monitoring and reporting of notifiable diseases and conditions. It serves as a surveillance system for diseases that are legally required to be reported to public health authorities. NOCS collects data from healthcare providers, laboratories, and other reporting entities.
Queensland Health IMS	Designed to support the management of health incidents and emergencies, such as infectious disease outbreaks or natural disasters. IMS facilitates the collection and analysis of real-time data from various sources, including hospitals, emergency departments, and PHUs. It provides a centralized platform for coordinating response efforts, resource allocation, and communication among different stakeholders. IMS captures information on case counts, hospital capacities, response actions, and logistics, enabling a coordinated and effective public health response during crises.
QSAS	QSAS is a database that stores data from a variety of surveys, including the Queensland Health Survey, the National Aboriginal and Torres Strait Islander Health Survey, and the National Drug Strategy Household Survey.
The QPHL	Maintains a database of laboratory results. This database is used to track the spread of infectious diseases and to identify new or emerging pathogens.
Queensland HIS	A central repository of health information that encompasses a wide range of data. It collects and stores data from various sources, including hospitals, clinics, and health programs. HIS captures information on hospital admissions, emergency department visits, outpatient services, and other healthcare encounters.
QHAPDC	Specifically dedicated to collecting and analysing data related to hospital admissions. It captures detailed information on patients admitted to public and private hospitals in Queensland.

Abbreviations: CDSS, Communicable Diseases Surveillance System; NOCS, Notifiable Conditions System; IMS, Incident Management System; QSAS, Queensland Health Survey Analytic System; QPHL, Queensland Public Health Laboratory; HIS, Health Information System; QHAPDC, Queensland Hospital Admitted Patient Data Collection; PHUs, public health units. ^a^ Data systems identified during this study were those relevant to the four case units (COVID-19, arbovirus, STIs, and TB). These may not represent a comprehensive list of all public health data systems.

 Concerns were also prevalent among participants interviewed for the STI/BBVA case unit, in the context of a generalised epidemic that started as an outbreak in western Queensland in 2012. As one informant noted, lack of inter-operability between data systems used by the HHS Sexual Health Units, by general practitioners (another key point of testing), and by PHUs (responsible for surveillance and population level responses) meant that:

 “*We [are] really trying to look at the [STI] surveillance numbers and try to work out where our little hot spots [are…] But as we know with data, it’s very difficult to get real-time numbers and sometimes there is a huge time lag between some of the information that we get” *(Int 6).

####  Data Governance

 Poorly understood data governance legislation and weak and inconsistent protocols for enacting data governance processes were also common issues raised within the STI/BBV, COVID-19 and TB case units. While Queensland has various policies (including Health Records Act 2001, Information Privacy Act 2009,^24^ Public Health Act 2005) governing the collection, use, and disclosure of health information, a lack of consistent protocols and guidance for when and how each should be applied meant each organisational unit and sub-unit makes its own decisions. Multiple informants spoke to a risk averse culture within Queensland Health as individuals and units sought to avoid situations where they might be reprimanded for stepping outside of policy.

 “*[We] have a data governance procedure for the unit, which doesn’t actually mention giving out any information […the data reports] seem to be only confined to Queensland Health because that’s the only people we can trust. [But] part of the service that we should be providing […] is actually to give people that data” *(Int 29).

 For those external to Queensland Health, this often manifested as difficulties in identifying who was the “data custodian” for the various data sources.

 “*It was so difficult to find the right person who is responsible for this data management. So we had – going three, four, five, six people, and then in the end – and that person was also not responsible” *(Int 41*).*

 The concept of “data sovereignty” was also discussed, particularly in relation to sharing surveillance data across government and non-government sectors in support of public health responses to STI/BBVs. In this realm, participants described some reluctance in the ACCHO sector to share data with Queensland Health because of a historical lack of reciprocity in data sharing arrangements and significant distrust arising.

 “*Queensland Health have the prevalence data, we have the screening data, and we don’t talk […] It’s data sovereignty. We’ve been burned before by giving over data, only for the funders to go and fund someone else […] We’ve just been burned in the sector. There’s a high, high level of distrust” *(Int 23).

 “*All the same patients that come in. Same patients come in, I’m sitting there [in the ACCHO] and you’re the Queensland Health staff right there and we’re all seeing the same people […] the information sharing, that’s the difficult part […] you have MOUs [memoranda of understanding] and stuff like that, but it doesn’t seem to actually work” *(Int 7).

####  Surveillance for Surveillance’s Sake

 Several case unit analyses demonstrated a culture of continuous data collection and surveillance for communicable disease surveillance but disconnected from any subsequent public health response. In the arbovirus case unit, participants described issues of data completeness and accuracy, with some cases being missed or underreported, but also a broader issue relating to whether surveillance systems and the data they produced were fit for purpose.

 “*Some of the entomologists [in Brisbane] have invented a way of detecting viruses in the mosquitoes because of their saliva […] But the problem is it doesn’t produce actionable intelligence. Ross River [virus], for example, we know the vectors are ubiquitous and cannot be controlled. We know that the virus comes and goes according to what’s going on in the marsupials [...] What are we supposed to do with this? So it’s a half-baked surveillance system” *(Int 15).

 The transition away from sentinel (pig) surveillance to mosquito surveillance for Japanese encephalitis, was another example of surveillance activity disconnected from actionable strategies:

 “*While mosquito surveillance is important […] the problem is – you can test tens of thousands of mosquitos and not get a single positive, even during an outbreak […] Where you’re really going to detect stuff is by working with the vets. And doing better surveillance in piggeries. And you know – actually having those conversations where you’ve got reporting channels, so when you’ve got something like reproductive failure in pigs, that [information is] going to come to people who are going to [say]: ‘ … ’ we really need to be looking at Japanese encephalitis!” *(Int 47).

 Several long-standing public health leaders, as well as the participants involved in arbovirus surveillance highlighted the critical role of human networks in enabling real-time conversations. These networks not only had the capacity to anticipate official alerts but also facilitated subsequent interpretation of data. Except for the COVID-19 case unit, (where exceptional human and financial resources facilitated the establishment of effective networks very quickly) participants described weak data feedback mechanisms and a decline in the human networks essential for transitioning from surveillance to planning and implementing public health responses to identified risks. To the contrary, several accounts highlighted that within HHSs and PHUs, a prevailing norm was to view data collection and compilation as the final step, rather than the beginning of a public health action cycle. Participants linked this norm to the (above reported) challenges with public health leadership and performance monitoring systems within HHSs:

 “*A lot of the problem is the surveillance; […] it’s one way, […] it’s feed the beast: ‘You have complied with the surveillance system’ [so you’ve done your job]” *(Int 15*).*

## Discussion

 This study aimed to identify areas for improvement in the governance of communicable disease surveillance and response system in NQ. Analysis pointed to limitations in both the strategic and implementation capabilities of those systems, influenced by not only regulatory factors such as non-alignment of legislation and HHS Service Level Agreements but also the central role of leadership, and organisational cultures. By using a systems-analysis approach, this study highlights the role of both “hardware” and “software” elements and emphasizes the need for greater alignment between regulatory, normative, and cultural elements in the public health system to improve future performance. In what follows, we discuss the dynamic interactions between hardware and software features related to (*i*) public health performance monitoring; (*ii*) data management and use, reflecting on their implications for policy and practice.

 Public health is a core governmental responsibility, with ministries or departments of health responsible for setting and ensuring adherence to standards, managing performance and instituting reforms as required.^25^ However, as demonstrated in the case of communicable disease surveillance and response in NQ, these functions can be undermined by weaknesses in performance monitoring, resource tracking and accountability systems.

 A key weakness in NQ’s public health governance is the limited ability to monitor the delivery of public health functions. Performance indicators found in Service Level Agreements between the Queensland state government and HHSs provide only a narrow basis for assessing public health service provision. These limitations are compounded by a lack of financial transparency for public health spending, making it difficult to assess past, or plan effectively for future, investments in the area. At both local and jurisdictional levels, weaknesses in resource allocation and accountability mirror national challenges. Shiell et al highlight the broader absence of clear financial tracking for public health spending across Australia and recommend reinstating annual public health spending reports by the Australian Institute of Health and Welfare, which were discontinued after 2008–2009.^26^

 Compounding weak monitoring of public health spending and performance in NQ were issues of organisational structure and leadership. Case study findings pointed to the challenges created by limited public health expertise among HHS senior leadership paired with the reliance of PHU directors on HHS executives for their annual budget. Informants in the TB, STI-BBV and arbovirus case units all highlighted the need for continual advocacy within their HHSs to secure even minimal operating funds. Reliance on short-term “project” funding to carry out surveillance and response work effectively constrained these public health functions due to turnover of personnel (on fixed term contracts) and associated loss of corporate knowledge and expertise. Such piecemeal and sporadic funding has been identified as a feature of the under-resourced national public health landscape too.^26^

 The cumulative effect of these structural and leadership gaps is a weakening of accountability for public health functions in the region. Although HHSs hold legal responsibility for key communicable disease surveillance and response functions, budgeting decisions are often delinked from actual or predicted public health needs.

 Without clear lines of accountability and beyond the current reliance on PHU internal processes and individual commitment, the risks of lapses in NQ’s underfunded communicable disease control systems are both real and largely unreported. Such gaps could lead to delays in disease outbreak identification and intensify public health crises. These risks are already evident within Australia, as demonstrated in the surveillance failures that allowed Japanese encephalitis—previously controlled—to spread into the southern states in 2022.^27,28^ Similar weaknesses in surveillance and oversight have had more severe consequences internationally. During the West African Ebola epidemic (2014 and 2016), for example, weak oversight that resulted in incomplete, incorrect, or untimely data collection was heavily implicated in the rapid spread and subsequent scope of the outbreak.^29^

 The second key finding of this study relates to the sub-optimal systems of data collation, governance, and use for communicable disease surveillance and response. Findings from the current study pointed to long-standing challenges in the STI-BBVs, arbovirus and TB data systems, with multiple accounts of missing or unavailable data highlighting the localised impacts of poor inter-operability caused by a wide range of disconnected data systems.

 System fragmentation significantly impedes communicable disease surveillance, pandemic planning, and response capacity and is not newly reported here. Rowe et al found that communicable disease registers across Australian states and territories lacked interoperability, limiting the ability to track disease epidemiology and respond swiftly to outbreaks.^30^ Poor data linkage for health policy and planning both within and between Australian jurisdictions is recognised as a major and longstanding challenge; and in 2017, the Productivity Commission recommended that Australia establish “enduring linkage systems” for health data to inform public policy and enable cross-jurisdictional comparison,^31^ although to date, it is not clear how far this recommendation has being progressed.

 Beyond technical fragmentation, this study also highlights how weak data governance frameworks interact with institutional cultures of risk aversion, further undermining data sharing and use. A lack of consistent guidance on data sharing—particularly concerning data sovereignty—contributes to distrust and reluctance among public health entities at different levels to share surveillance data. As a result, even when surveillance data is collected, it is not necessarily shared or used for planning purposes. Addressing these weaknesses requires investment not only in technical skills and digital platforms but also in clear policies that enable well-monitored but open data-sharing practices.^32^ Indeed, an effective “public health intelligence” function depends as much on trust and well-networked experts as on technical capabilities. Developing a culture of responsible data sharing, supported by clear governance frameworks and professional norms, is essential for strengthening public health decision-making and outbreak response capacity.^33^

 This study demonstrates the usefulness of applying a systems-based perspective to public health governance. By using Sheikh and colleagues’ hardware-software framework,^13^ the study highlights how political, normative, and structural factors shape public health performance alongside the more commonly assessed functional and technical dimensions. In 2023, Queensland Health published a state-wide review of public health which noted a range of similar hardware challenges, particularly fragmentation of the public health resourcing, activities and responsibilities, and calling among other things for improved coordination across multiple stakeholders.^34^ This study supports those conclusions but adds depth by illustrating how the governance of communicable disease surveillance and control is being shaped not only by “hardware” (organisational and regulatory) but also “software” (values, norms and relationship) factors. It makes an important contribution given the tendency for reform or service strengthening efforts to focus on operational efficiencies and workforce competencies while glossing over the relational, cultural, and institutional determinants of capacity and effectiveness.

 With consistently low levels of public health spending relative to other OECD (the Organisation for Economic Co-operation and Development) nations recently reported by Shiell et al,^26^ it is likely that the hardware challenges relating to public health resourcing reported above are not unique to NQ. Softwarechallenges too may have relevance elsewhere in Australia given the currently limited availability of public health leadership training outside of jurisdiction- and city-specific programs in New South Wales, Victoria and Perth.^35-37^ Thus, while the governance issues identified in this study are shaped by one state’s specific health system architecture, the core finding—that communicable disease and more broadly public health governance must account for the interplay between formal structures as well as norms and informal power dynamics—is likely to have analytical relevance to other settings with decentralised public health systems, where balancing local autonomy with system-wide accountability remains a persistent challenge.

## Conclusion

 Communicable disease surveillance and response systems are designed to monitor, detect, and respond to health threats and are influenced by a myriad of factors. To explain the gaps in governance of the public health surveillance in NQ, this study identified a combination of hardware features related to poor and disconnected data systems and software features related to leadership and data-sharing culture that affect its performance. The findings highlight the importance of understanding software elements to identify opportunities for strengthening the governance of communicable disease surveillance and response system.

## Acknowledgements

 We would like to acknowledge and thank all the participants for their time and generosity in sharing their experiences and providing critical feedback during dissemination forums.

## Ethical issues

 This project received approval from the Townsville HHS Human Research Ethics Committee on 28 November 2019 (HREC/2019/QTHS/59811) and reciprocal approval from James Cook University HREC on January 15, 2020. Site-specific governance approval was secured from the Townsville HHS, Cairns and Hinterland HHS, Torres and Cape HHS.

## Conflicts of interest

 Authors declare that they have no conflicts of interest.

## Supplementary files


Supplementary file 1. Question Guide – In-Depth Interviews With Key Stakeholders.

Supplementary file 2. Service Agreements for Five NQ Hospital and Health Services.
