# Supplementary material for: "It’s Feeding the Beast": Lessons for Governance of Public Health Surveillance and Response From an Australian Case Study Analysis
Source: Int J Health Policy Manag. 2025 May 18;14:8605. doi: 10.34172/ijhpm.8605 (PMC12257200; doi:10.34172/ijhpm.8605)
Supplement: Supplementary file 1 — Question Guide – In-Depth Interviews With Key Stakeholders. [file ijhpm-14-8605-s001.pdf]

Article title: "It's Feeding the Beast": Lessons for Governance of Public Health Surveillance and Response From an Australian Case Study Analysis

Journal name: International Journal of Health Policy and Management (IJHPM)

Authors' information: Stephanie M. Topp[1]*, Alexandra Edelman[2], Thu Nguyen[1], Emma S. McBryde[3], Sue Devine[1], Tammy Allen[1], Jeffrey Warner[1], Julie Mudd[4], Paul F. Horwood[1]

[1]Public Health and Tropical Medicine, College of Medicine and Dentistry, James Cook University, Townsville, QLD, Australia.

[2]Menzies School of Health Research, Charles Darwin University, Darwin, NT , Australia.

[3]Australian Institute of Tropical Health and Medicine, James Cook University, Townsville, QLD, Australia.

[4]College of Medicine and Dentistry, James Cook University, Townsville, QLD, Australia.

***Correspondence to:** Stephanie M. Topp; Email: stephanie.topp@jcu.edu.au

Citation: Topp SM, Edelman A, Nguyen T, et al. "It's feeding the beast": Lessons for governance of public health surveillance and response from an Australian case study analysis.Int J Health Policy Manag. 2025;14:8605. doi:10.34172/ijhpm.8605

Supplementary file 1. Question Guide – In-Depth Interviews With Key Stakeholders

Many thanks for agreeing to participate in this interview.

1. Could you please introduce yourself and your current role?
2. Can you describe what involvement and responsibilities you have for surveillance and/or response to [COVID-19 / TB / arboviruses] in your current role?
3. How important do you believe surveillance and response to [COVID-19 / STI-BBV / TB / arboviruses] are for Queensland specifically and Australia more generally? [why]

Thinking about surveillance first:

4. From your perspective what are they key strengths of the current [COVID-19 / STI-BBV / TB / arboviruses] surveillance systems in north Queensland?
5. Which organisations, and which individuals would you say play critical roles in the current system? [consider all phases: data collection; data integration; analysis and interpretation; public health action; dissemination; system evaluation]
6. Based on your experience, are there any areas of the surveillance system you would like to strengthen? [eg: resourcing; interoperability; governance; information systems]
7. In your experience, how do issues of trust and inter-organisational / jurisdictional relationships influence surveillance for [influenza / TB / arboviruses]? [explain]

Coming to public health action / response systems:

8. What are the key pathways and mechanisms currently in place to respond to cases or outbreaks of [COVID-19 / STI-BBV / TB / arboviruses]?
9. Thinking about those pathways, are there any individuals whose role is particularly important to the maintenance and function of effective response planning or implementation?
10. Thinking broadly, what would you say are the human strengths and weaknesses of our response system for [COVID-19 / STI-BBV / TB / arboviruses]?
11. In your experience, do issues of trust and inter-organisational / jurisdictional relationships influence public health and service responsiveness in this area? [explain]
12. If we were to try to strengthen communicable disease response systems in north Queensland and Australia more broadly, what would you see as a priority?

Thank you for sharing these experiences; is there anything I haven't asked about that you feel it is important to understand in relation to the surveillance and response systems locally or nationally?
