# Supplementary material for: "It’s Feeding the Beast": Lessons for Governance of Public Health Surveillance and Response From an Australian Case Study Analysis
Source: Int J Health Policy Manag. 2025 May 18;14:8605. doi: 10.34172/ijhpm.8605 (PMC12257200; doi:10.34172/ijhpm.8605)
Supplement: Supplementary file 2 — Service Agreements for Five NQ Hospital and Health Services. [file ijhpm-14-8605-s002.pdf]

Service Agreement

2019/20 – 2021/22

Cairns and Hinterland Hospital and Health Service

July 2020 Revision

Queensland Government

Cairns and Hinterland Hospital and Health Service

Service Agreement 2019/20 - 2021/22, July 2020 Revision

Published by the State of Queensland (Queensland Health), July 2020

This document is licensed under a Creative Commons Attribution 3.0 Australia licence. To view a copy of this licence, visit creativecommons.org/licenses/by/3.0/au

© State of Queensland (Queensland Health) **2020**

You are free to copy, communicate and adapt the work, as long as you attribute the State of Queensland (Queensland Health).

For more information contact:
Contracting and Performance Management Branch, Department of Health, GPO Box 48, Brisbane QLD 4001, email SAM@health.qld.gov.au, phone (07) 3708 5869.

An electronic version of this document is available at www.health.qld.gov.au/system-governance/health-system/managing/default.asp

Disclaimer:
The content presented in this publication is distributed by the Queensland Government as an information source only. The State of Queensland makes no statements, representations or warranties about the accuracy, completeness or reliability of any information contained in this publication. The State of Queensland disclaims all responsibility and all liability (including without limitation for liability in negligence) for all expenses, losses, damages and costs you might incur as a result of the information being inaccurate or incomplete in any way, and for any reason reliance was placed on such information.

Contents

1. Introduction 6
2. Interpretation 6
3. Legislative and regulatory framework 7
4. Health system priorities 7
5. Objectives of the Service Agreement 9
6. Scope 9
7. Performance and Accountability Framework 9
8. Period of this Service Agreement 10
9. Amendments to this Service Agreement 10
10. Publication of amendments 10
11. Cessation of service delivery 10
12. Commencement of a new service 11
13. Provision of data to the Chief Executive 12
14. Dispute resolution 12
15. Force Majeure 15
16. Hospital and Health Service accountabilities 16
17. Department accountabilities 16
18. Insurance 18
19. Indemnity 18
20. Indemnity arrangements for officers, employees and agents 19
21. Legal proceedings 19
22. Sub-contracting 19
23. Counterparts 20

Execution 21

Schedule 1 HHS Accountabilities 22

1. Purpose 22
2. Registration, credentialing and scope of clinical practice 22
3. Clinical Services Capability Framework 22
4. Clinical Prioritisation Criteria 23
5. Service delivery 23
6. Accreditation 24
7. Responsive regulatory process for accreditation 25
8. Achieving health equity for First Nations Queenslanders 26
9. Provision of Clinical Products/Consumables in outpatient settings 27
10. Capital, land, buildings, equipment and maintenance 28
11. Occupational health and safety 30
12. Workforce management 30
13. Medically authorised ambulance transports 31

Schedule 2 Funding, purchased activity and services 32

1. Purpose 32
2. Delivery of purchased activity 32
3. Financial adjustments 33

4. Funding sources 50
5. Funds disbursement 52
6. Purchased services 61
7. Teaching training and research 69
Schedule 3 Performance Measures 72
1. Purpose 72
2. Performance Measures 72
Schedule 4 Data Supply Requirements 77
1. Purpose 77
2. Principles 77
3. Roles and responsibilities 77
Attachment A Data Supply Requirements 79
Schedule 5 Amendments to this Service Agreement 81
1. Purpose 81
2. Principles 81
3. Process to amend this Service Agreement 81
Schedule 6 Definitions 86
Key Documents 93
Abbreviations 95

Figures

Figure 1 Dispute resolution process ... 13
Figure 2 Inter-HHS dispute resolution process ... 15
Figure 3 Amendment Proposal negotiation and resolution ... 83

Tables

Table 1 Specific Funding Commitments ... 34
Table 2 Purchasing Incentives 2020/21 (Summary) ... 49
Table 3 Hospital and Health Service funding sources 2020/21 ... 51
Table 4 HHS Finance and Activity Schedule 2019/20 – 2021/22 – Summary by Purchasing Hierarchy ... 53
Table 5 Minor Capital and Equity ... 55
Table 6 HHS Finance and Activity Schedule 2019/20 – 2021/22 Other Funding Detail ... 56
Table 7 Specified Grants ... 59
Table 8 Hospital and Health Service Service Agreement and State Level Block Payments to state managed funds from Commonwealth payments into national funding pool ... 60
Table 9 HHS Performance Measures – Key Performance Indicators ... 73
Table 10 HHS Performance Measures - Safety and Quality Markers ... 74
Table 11 HHS Performance Measures – Outcome Indicators ... 75
Table 12 Clinical data ... 79
Table 13 Non-clinical data ... 80
Table 14 Amendment Window Exchange Dates ... 82

1. Introduction

1.1 The Queensland Public Sector Health System is committed to strengthening performance and improving services and programs in order to meet the needs of the community and deliver improved health outcomes to all Queenslanders.

1.2 The development of Service Agreements between the Chief Executive and Hospital and Health Services (HHSs), assists this process by formally assigning accountability for the high-level outcomes and targets to be met during the period to which the Service Agreement relates.

1.3 The content and process for the preparation of this Service Agreement is consistent with the requirements of the *Hospital and Health Boards Act 2011*. As such this Service Agreement specifies:

(a) the Health Services and other services to be provided by the HHS;

(b) the funding which is provided to the HHS for the provision of these services and the way in which the funding is to be provided;

(c) the Performance Measures that the HHS will meet for the services provided;

(d) data supply requirements; and

(e) other obligations of the Parties.

1.4 Fundamental to the success of this Service Agreement is a strong collaboration between the HHS and its Board and the Department. This collaboration is supported through regular Performance Review Meetings attended by representatives from both the HHS and the Department which provide a forum within which a range of aspects of HHS and system wide performance are discussed and jointly addressed.

2. Interpretation

Unless expressed to the contrary, in this Service Agreement:

(a) words in the singular include the plural and vice versa;

(b) any gender includes the other genders;

(c) if a word or phrase is defined its other grammatical forms have corresponding meanings;

(d) “includes” and “including” are not terms of limitation;

(e) no rule of construction will apply to a clause to the disadvantage of a Party merely because that Party put forward the clause or would otherwise benefit from it;

(f) a reference to:

(i) a Party is a reference to a Party to this Service Agreement;

(ii) a person includes a partnership, joint venture, unincorporated association, corporation and a government or statutory body or authority;

(iii) a person includes the person’s legal personal representatives, successors, assigns and persons substituted by novation;

(g) any legislation includes subordinate legislation under it and includes that legislation and subordinate legislation as modified or replaced;

(h) a reference to a role, function or organisational unit is deemed to transfer to an equivalent successor role, function or organisational unit in the event of organisational change or restructure in either Party;

(i) an obligation includes a warranty or representation and a reference to a failure to comply with an obligation includes a breach of warranty or representation;

(j) headings do not affect the interpretation of this Service Agreement;

(k) unless the contrary intention appears, a reference to a Schedule, annexure or attachment is a reference to a Schedule, annexure or attachment to this Service Agreement; and

(l) unless the contrary intention appears, words in the Service Agreement that are defined in Schedule 6 ‘Definitions’ have the meaning given to them in that Schedule.

3. Legislative and regulatory framework

3.1 This Service Agreement is regulated by the National Health Reform Agreement and the provisions of the *Hospital and Health Boards Act 2011.*

3.2 The National Health Reform Agreement requires the State of Queensland to establish Service Agreements with each HHS for the purchasing of health services and to implement a performance and accountability framework including processes for remediation of poor performance. The *Hospital and Health Boards Act 2011* states under section 35(3) that the Service Agreement executed between the Chief Executive and the Hospital and Health Board Chair binds each of them.

3.3 The *Hospital and Health Boards Act 2011* recognises and gives effect to the principles and objectives of the national health system agreed by the commonwealth, state and territory governments, including the Medicare principles and health system principles set out in section 4. Section 5 of the *Hospital and Health Boards Act 2011* states that the object of the Act is to establish a Public Sector Health System that delivers high-quality hospital and other Health Services to persons in Queensland having regard to the principles and objectives of the national health system. This Service Agreement is an integral part of implementing these objectives and principles.

4. Health system priorities

4.1 Ensuring the provision of Public Sector Health Services across Queensland requires clear priorities, supportive leadership and staff who work together and across each level of the Public Sector Health System. The Parties recognise that they each have a mutual and reciprocal obligation to work collaboratively with each other, with other Hospital and Health

Services (HHS) and with the Queensland Ambulance Service in the best interests of the Queensland Public Sector Health System.

4.2 The priorities, goals and outcomes for the Queensland Public Sector Health System are defined through:

(a) *Our Future State: Advancing Queensland's Priorities* - the Queensland Government's objectives for the community; and

(b) *My health, Queensland's future: Advancing health 2026* – the vision and strategy for Queensland's health system.

4.3 The Parties will also work collaboratively to deliver the *Queensland Health 2020/21 System Priorities.* The *Queensland Health 2020/21 System Priorities* establishes a tactical framework which will ensure that the Queensland Public Sector Health System delivers sustainable, high quality and timely Health Services during 2020/21, whilst remaining positioned to respond effectively to the COVID-19 pandemic.

4.4 Additionally, the Queensland Government, Premier or the Minister for Health and Minister for Ambulance Services (The Minister) may articulate key priorities, themes and issues from time to time.

4.5 HHSs have a responsibility to ensure that the delivery of Public Sector Health Services in Queensland is consistent with these strategic directions and priorities.

4.6 The Parties will collectively identify, develop, implement and evaluate strategies that support the delivery of priorities identified by the Minister, and which align with a Value-Based Healthcare approach to the delivery of Health Services.

4.7 In accordance with section 9 of the *Financial and Performance Management Standard 2009*, HHSs are required to develop a strategic plan. The HHS's strategic plan will reflect local priorities and will be developed considering the shared Queensland priorities outlined in the Queensland Government's objectives for the community, the Ministers' articulated priorities and *My health, Queensland's future: Advancing health 2026*.

4.8 The Parties have a collective responsibility to contribute to a sustainable Public Sector Health System in Queensland. Planning and delivery of Health Services will be aligned with the system planning agenda set out in *Queensland Health System Outlook to 2026 for a sustainable health service* in order to ensure a coordinated, system-wide response to growing demand for Health Services.

4.9 In delivering Health Services, HHSs are required to meet the applicable conditions of all national agreements and national partnership agreements between the Queensland Government and the Commonwealth Government and commitments under any related implementation plans.

4.10 This Service Agreement is underpinned by and is to be managed in line with the following supporting documents:

(a) Queensland Health System Outlook to 2026 for a sustainable health service;

(b) Performance and Accountability Framework 2020/21; and

(c) Purchasing Policy and Funding Guidelines 2020/21.

5. Objectives of the Service Agreement

This Service Agreement is designed to:

(a) specify the Health Services, teaching, research and other services to be provided by the HHS;

(b) specify the funding to be provided to the HHS for the provision of the services;

(c) specify the Performance Measures for the provision of the services;

(d) specify the performance and other data to be provided by the HHS to the Chief Executive;

(e) provide a platform for greater public accountability; and

(f) facilitate the achievement of State and Commonwealth Government priorities, services, outputs and outcomes while ensuring local input.

6. Scope

6.1 This Service Agreement outlines the services that the Department will purchase from the HHS during the period of this Service Agreement.

6.2 This Service Agreement does not cover the provision of clinical and non-clinical services by the Department, including the Queensland Ambulance Service, to the HHS. Separate arrangements will be established for those services provided by Health Support Queensland and eHealth Queensland.

7. Performance and Accountability Framework

7.1 The Performance and Accountability Framework sets out the framework within which the Department, as the overall manager of Public Health System Performance, monitors and assesses the performance of Public Sector Health Services in Queensland. The systems and processes employed for this purpose include, but are not limited to, assessing and monitoring HHS performance, reporting on HHS performance and, as required, intervening to manage identified performance issues.

7.2 During 2020/21 the Performance and Accountability Framework will support delivery of the *Queensland Health System Priorities 2020/21* which focus on realising positive changes to the Public Sector Health System through providing sustainable, timely, safe and high-quality Health Services in the right setting whilst remaining ready to respond to the COVID-19 pandemic.

7.3 The Performance and Accountability Framework uses Performance Measures to monitor the extent to which HHSs are delivering the high-level objectives set out in this Service Agreement. The Key Performance Indicators and other measures of performance against which the HHS will be assessed and benchmarked are detailed in Schedule 3 of this Service Agreement.

7.4 The Parties agree to constructively implement the Performance and Accountability Framework.

8. Period of this Service Agreement

8.1 This Service Agreement commences on the Effective Date and expires on 30 June 2022. The Service Agreement framework is in place for three years in order to provide HHSs with a level of guidance regarding funding and purchased activity for the outer years.

8.2 In this Service Agreement, references to years are references to the period commencing on 1 July and ending on 30 June unless otherwise stated.

8.3 Using the provisions of the *Hospital and Health Boards Act 2011* as a guide, the Parties will enter into funding and purchased activity negotiations for the following year six months before the end of the current year.

8.4 In accordance with the *Hospital and Health Boards Act 2011* the Parties will enter negotiations for the next Service Agreement at least six months before the expiry of the existing Service Agreement.

9. Amendments to this Service Agreement

9.1 Section 39 of the *Hospital and Health Boards Act 2011* requires that, if the Chief Executive or the HHS wish to amend the terms of a Service Agreement, the Party wishing to amend the Service Agreement must give written notice of the proposed amendment to the other Party.

9.2 The process for amending this Service Agreement is set out in Schedule 5 of this Service Agreement.

10. Publication of amendments

The Department will publish each executed Deed of Amendment within 14 days of the date of execution on www.health.qld.gov.au/system-governance/health-system/managing/default.asp.

11. Cessation of service delivery

11.1 The HHS is required to deliver the Health Services and other services outlined in this Service Agreement for which funding is provided in Schedule 2. Any changes to service delivery must ensure maintenance of care and minimise disruptions to patients.

11.2 The Department and HHS may Terminate or temporarily Suspend a Health Service or other service by mutual agreement having regard to the following obligations:

(a) any proposed Termination or Suspension must be made in writing to the other Party;

(b) where it is proposed to Terminate or Suspend a Statewide Service, or a Regional Service, the HHSs which are in receipt of that service must also be consulted;

(c) the Parties must agree on a reasonable notice period following which Termination, or Suspension, will take effect; and

(d) patient needs, workforce implications, relevant government policy and HHS sustainability are to be considered.

11.3 The Department, in its role as the Queensland Public Health System manager:

(a) may, in its unfettered discretion, not support a requested Termination or Suspension and require the HHS to maintain the service; and

(b) will reallocate existing funding and activity for the Terminated or Suspended service inclusive of baseline Service Agreement funding and in-year growth funding on a pro-rata basis.

11.4 The HHS will:

(a) work with the Department to ensure continuity of care and a smooth transfer of the service to an alternative provider where this is necessary; and

(b) minimise any risk or inconvenience to patients associated with service Termination, Suspension or transfer.

11.5 In the event that a sustainable alternative provider cannot be identified, and this is required, the service and associated patient cohort will continue to remain the responsibility of the HHS.

12. Commencement of a new service

12.1 In the event that the HHS wishes to commence providing a new Health Service, the HHS will notify the Department in writing in advance of commencement.

12.2 The Department will provide a formal response regarding the proposed new Health Service to the HHS in writing. The Department may not agree to purchase the new Health Service or to provide funding on either a recurrent or non-recurrent basis.

12.3 In the event that a change to an established Referral Pathway is proposed which would result in the direction of patient referrals to an alternative HHS on a temporary or a permanent basis:

(a) the new Referral Pathway must be agreed by all impacted HHSs prior to its implementation; and

(b) following agreement of the new Referral Pathway, if there is an identifiable and agreed impact to funding the Department will redistribute funding and activity between HHSs in alignment with new Referral Pathway.

13. Provision of data to the Chief Executive

The HHS will provide to the Chief Executive the performance data and other data, including data pursuant to ad hoc requests, set out in Schedule 4 of this Service Agreement in accordance with the Schedule, including in relation to the form, manner and the times required for the provision of data.

14. Dispute resolution

14.1 The dispute resolution process set out below is designed to resolve disputes which may arise between the Parties to this Service Agreement in a final and binding manner.

14.2 These procedures and any disputes addressed or to be addressed by them are subject to the provisions of the *Hospital and Health Boards Act 2011*, including in respect of any directions issued under that legislation or by Government in respect of any dispute.

14.3 Resolution of disputes will be through a tiered process commencing with the Performance Review Meeting and culminating, if required, with the Minister, as illustrated in Figure 1.

14.4 Use of the dispute resolution process set out in this clause should only occur following the best endeavours of both Parties to agree a resolution to an issue at the local level. The dispute resolution process is not intended for the resolution of ongoing issues or performance related issues. At each stage of the dispute resolution process, the Parties agree to cooperate.

14.5 If a dispute arises in connection with this Service Agreement (including in respect of interpretation of the terms of this Service Agreement), then either Party may give the other a written Notice of Dispute.

14.6 The Notice of Dispute must be provided to the D-SA Contact Person if the notice is being given by the HHS and to the HHS-SA Contact Person if the notice is being given by the Department.

14.7 The Notice of Dispute must contain the following information:

(a) a summary of the matter in dispute;

(b) an explanation of how the Party giving the Notice of Dispute believes the dispute should be resolved and reasons to support that belief;

(c) any information or documents to support the Notice of Dispute; and

(d) a definition and explanation of any financial or Service delivery impact of the dispute.

Figure 1 Dispute resolution process

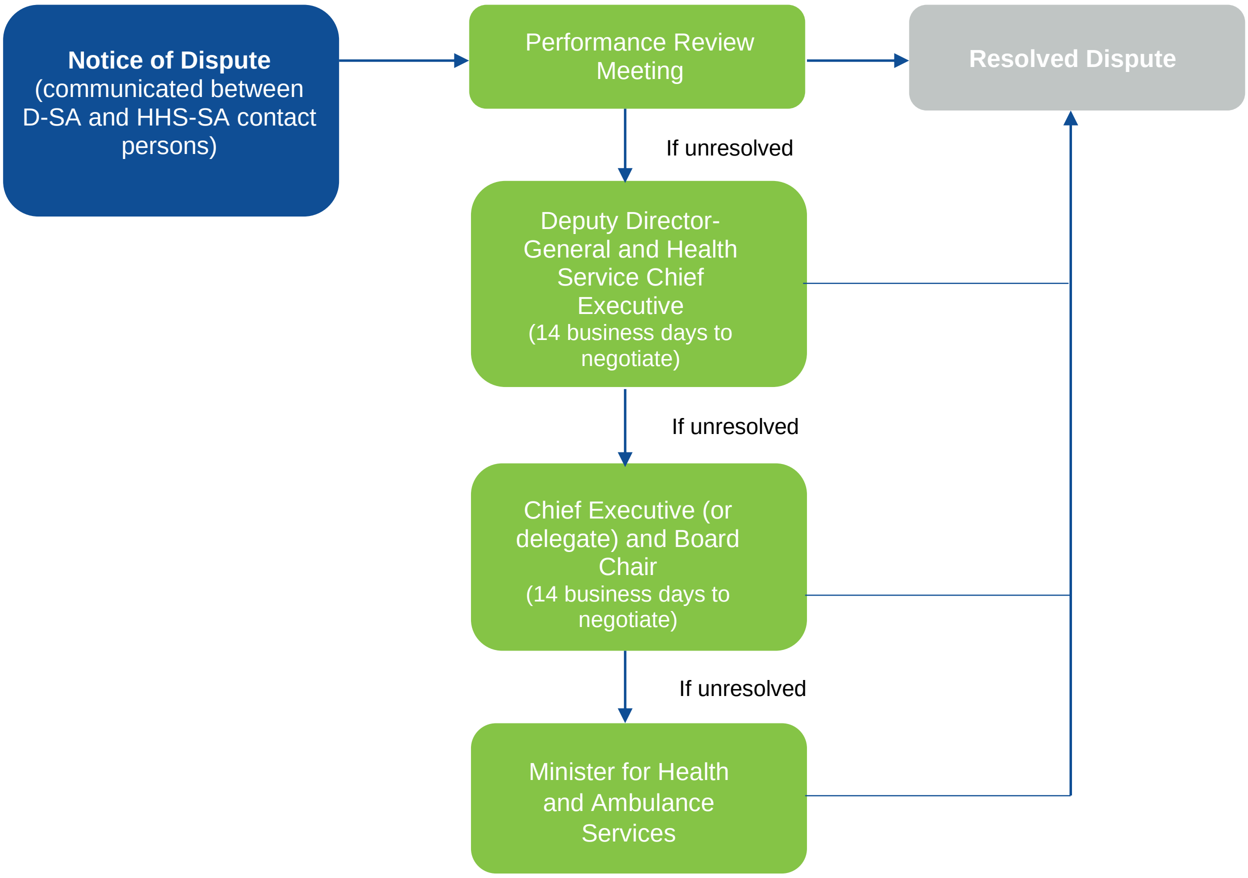

14.8 Resolution of a dispute

(a) Resolution of a dispute at any level is final. The resolution of the dispute is binding on the Parties but does not set a precedent to be adopted in similar disputes between other Parties.

(b) The Parties agree that each dispute (including the existence and contents of each Notice of Dispute) and any exchange of information or documents between the Parties in connection with the dispute is confidential and must not be disclosed to any third party without the prior written consent of the other Party, other than if required by law and only to the extent required by law.

14.9 Continued performance

Notwithstanding the existence of one or more disputes, the HHS must continue to perform and comply with this Service Agreement to the best of their abilities given the circumstances.

14.10 Disputes arising between Hospital and Health Services

(a) In the event of a dispute arising between two or more HHSs (an Inter-HHS Dispute), the process set out in Figure 2 will be initiated. Resolution of Inter-HHS Disputes will be through a tiered process, commencing with local resolution and culminating if required with formal and binding arbitration by the Minister under the provisions of the *Hospital and Health Boards Act 2011*, section 44.

(b) If the HHS wishes to escalate a dispute, the HHS will be expected to demonstrate that best endeavours (including Chair and Board involvement) to resolve the dispute between all parties at an informal and local level have taken place.

(c) Management of inter-HHS relationships should be informed by the following principles:

(i) HHSs should maintain (for both the base level of funding and growth) the proportion of out of HHS work undertaken unless as a result of agreed repatriation of patients.

(ii) All HHSs manage patients from their own catchment population if it is within their clinical capability to do so as specified by the Clinical Services Capability Framework.

(iii) Where it is proposed that a Health Service move from one HHS to another, agreement between the respective Health Service Chief Executives will be secured prior to any change in patient flows. Once agreed, funding will follow the patient.

(iv) All HHSs abide by the agreed dispute resolution process.

(v) All HHSs operate in a manner which is consistent with the health system principles and objectives as set out in the National Health Reform Agreement and the *Hospital and Health Boards Act 2011*.

Figure 2 Inter-HHS dispute resolution process

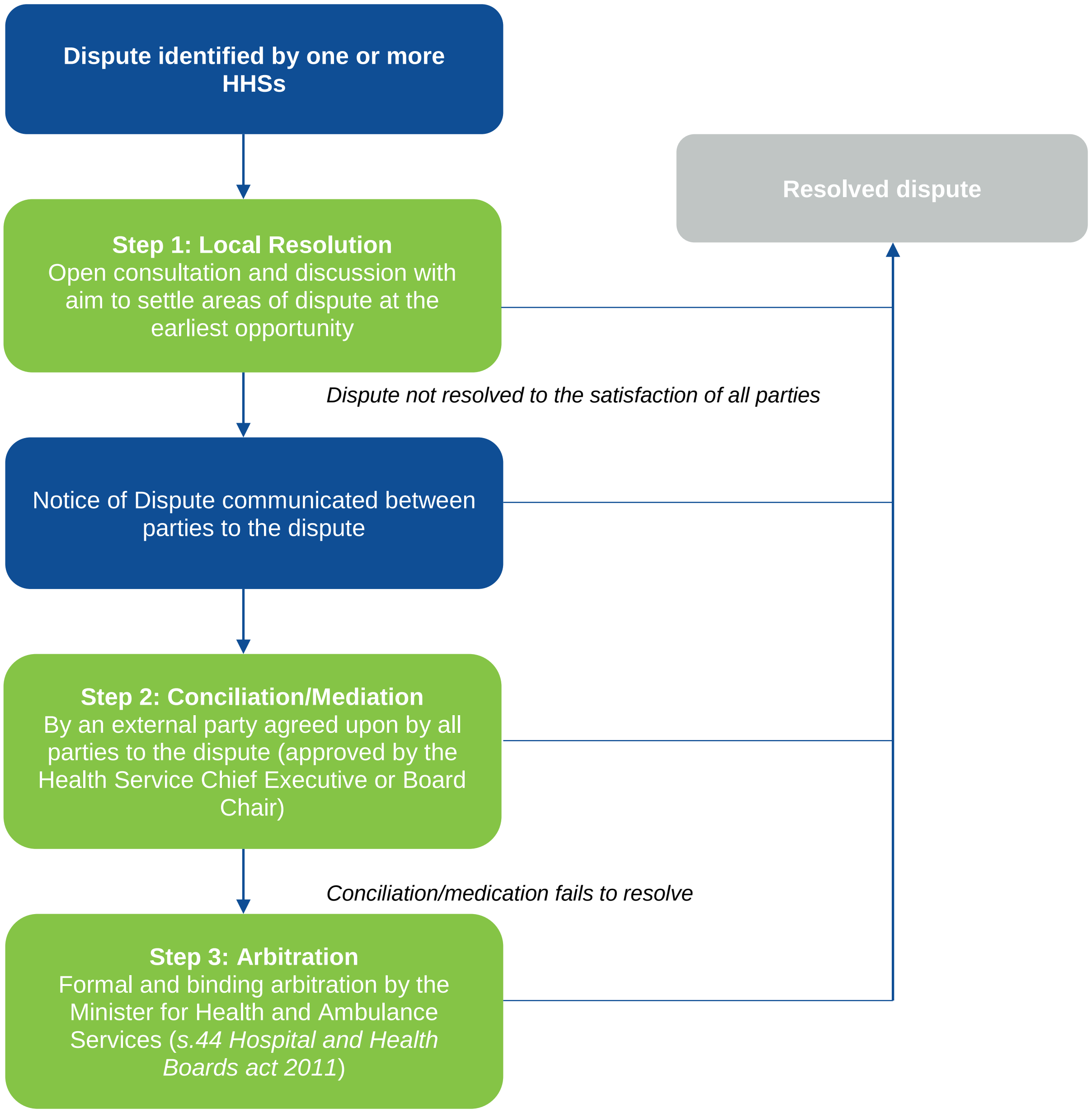

15. Force Majeure

15.1 If a Party (Affected Party) is prevented or hindered by Force Majeure from fully or partly complying with any obligation under this Service Agreement, that obligation may (subject to the terms of this Force Majeure clause) be suspended, provided that if the Affected Party wishes to claim the benefit of this Force Majeure clause, it must:

(a) give prompt written notice of the Force Majeure to the other Party of:

(i) the occurrence and nature of the Force Majeure;

(ii) the anticipated duration of the Force Majeure;

(iii) the effect the Force Majeure has had (if any) and the likely effect the Force Majeure will have on the performance of the Affected Party's

obligations under this Service Agreement; and

(iv) any disaster management plan that applies to the party in respect of the Force Majeure.

(b) use its best endeavours to resume fulfilling its obligations under this Service Agreement as promptly as possible; and

(c) give written notice to the other Party within five days of the cessation of the Force Majeure.

15.2 Without limiting any other powers, rights or remedies of the Chief Executive, if the Affected Party is the HHS and the delay caused by the Force Majeure continues for more than 14 days from the date that the Chief Executive determines that the Force Majeure commenced, the Chief Executive may give directions to the HHS regarding the HHS's performance or non-performance of this Service Agreement during the Force Majeure and the HHS must comply with that direction.

15.3 Neither Party may terminate this Service Agreement due to a Force Majeure event.

16. Hospital and Health Service accountabilities

16.1 Without limiting any other obligations of the HHS, it must comply with:

(a) the terms of this Service Agreement;

(b) all legislation applicable to the HHS, including the *Hospital and Health Boards Act 2011*;

(c) all Cabinet decisions applicable to the HHS;

(d) all Ministerial directives applicable to the HHS;

(e) all agreements entered into between the Queensland and Commonwealth governments applicable to the HHS;

(f) all regulations made under the *Hospital and Health Boards Act 2011*;

(g) all Industrial Instruments applicable to the HHS; and

(h) all health service directives applicable to the HHS.

16.2 The HHS will ensure that the accountabilities set out in Schedule 1 of this Service Agreement are met.

17. Department accountabilities

17.1 Without limiting any other obligations of the Department, it must comply with:

(a) the terms of this Service Agreement;

(b) the legislative requirements as set out within the *Hospital and Health Boards Act 2011*;

(c) all regulations made under the *Hospital and Health Boards Act 2011*; and

(d) all Cabinet decisions applicable to the Department.

17.2 The Department will work in collaboration with HHSs to ensure the Public Sector Health System delivers high quality hospital and other Health Services to persons in Queensland having regard to the principles and objectives of the national health system. In support of realising this objective, in accordance with section 5 of the *Hospital and Health Boards Act 2011* the Department will:

(a) provide overall management of the Queensland Public Sector Health System including health system planning, coordination and standard setting;

(b) provide the HHS with funding specified under Schedule 2 of this Service Agreement;

(c) provide and maintain payroll and rostering systems to the HHS unless agreed otherwise between the Parties;

(d) operate 13 HEALTH as a first point of contact for health advice with timely HHS advice and information where appropriate to local issues; and

(e) balance the benefits of a local and system-wide approach.

17.3 The Department will endeavour to purchase services in line with Clinical Prioritisation Criteria, where these have been developed, in order to improve equity of access and reflect the scope of publicly funded services.

17.4 The Department will maintain a public record of the Clinical Service Capability Framework levels for all public facilities based on the information provided by HHSs.

17.5 **Workforce management**

The Chief Executive agrees to appoint Health Service Employees to:

(a) perform work for the HHS for the purpose of enabling the HHS to perform its functions and exercise powers under the *Hospital and Health Boards Act 2011;* and

(b) deliver the services specified in this Service Agreement.

17.6 The Chief Executive, whether in the capacity of a Person Conducting a Business or Undertaking as an employer or as a building owner or occupier, will consult, cooperate and coordinate with the HHS to ensure legal compliance with the *Work Health and Safety Act 2011* and other legislation as it applies to the scope, nature and location of operations associated with this Service Agreement. This includes, but is not limited to:

(a) codes of practice;

(b) electrical safety legislation;

(c) building and fire safety legislation; and

(d) workers’ compensation legislation.

18. Insurance

18.1 The HHS must hold and maintain for the period of this Service Agreement the types and levels of insurances that the HHS considers appropriate to cover its obligations under this Service Agreement.

18.2 Without limiting the types and levels of insurances that the HHS considers appropriate, any insurance policies taken out by the HHS under this clause must include appropriate coverage for the following:

(a) public and product liability insurance;

(b) professional indemnity insurance; and

(c) workers' compensation insurance in accordance with the *Workers' Compensation and Rehabilitation Act 2003* (Qld).

18.3 The HHS will be deemed to comply with its requirements under clauses 18.1 and 18.2(a) and 18.2(b) if it takes out and maintains a current insurance policy with the Queensland Government Insurance Fund.

18.4 Any insurance policies held by the HHS pursuant to this clause must be effected with an insurer that is authorised and licensed to operate in Australia.

18.5 The HHS must maintain a current register of all third-party guarantees.

18.6 The HHS must, if requested by the Department, promptly provide a sufficiently detailed certificate of currency and/or insurance and policy documents for each insurance policy held by the HHS pursuant to this clause.

18.7 The HHS warrants that any exclusions and deductibles that may be applicable under the insurance policies held pursuant to this clause will not impact on the HHS's ability to meet any claim, action or demand or otherwise prejudice the Department's rights under this Service Agreement.

18.8 The HHS must immediately advise the Department if any insurance policy, as required by this clause, is materially modified or cancelled.

19. Indemnity

19.1 The HHS indemnifies the Department against all and any liabilities, claims, actions, demands, costs and expenses made by any person which may be brought against or made upon or incurred by the Department arising directly or indirectly from or in connection with any of the following:

(a) any wilful, unlawful or negligent act or omission of the HHS, a Health Service Employee, Health Executive, Senior Health Service Employee or an officer, employee or agent working for the HHS in the course of the performance or attempted or purported performance of this Service Agreement;

(b) any penalty imposed for breach of any applicable law in relation to the HHS's performance of this Service Agreement; and

(c) a breach of this Service Agreement,

except to the extent that any act or omission by the Department caused or contributed to the liability, claim, action, demand, cost or expense.

19.2 The indemnity referred to in this clause will survive the expiration or termination of this Service Agreement.

20. Indemnity arrangements for officers, employees and agents

20.1 Indemnity arrangements for officers, employees or agents working for the Public Sector Health System are administered in accordance with the following policy documents, as amended from time to time:

(a) Indemnity for Queensland Health Medical Practitioners HR Policy I2 (QH-POL-153:2014); and

(b) Queensland Government Indemnity Guideline.

20.2 The costs of indemnity arrangements provided for Health Service Employees, Health Executives, Senior Health Service Employees, or officers, employees or agents working for the HHS are payable by the HHS.

21. Legal proceedings

21.1 This clause applies if there is any demand, claim, liability or legal proceeding relating to assets, contracts, agreements or instruments relating to the HHS, whether or not they are:

(a) transferred to an HHS under section 307 of the *Hospital and Health Boards Act 2011*; or

(b) retained by the Department.

21.2 Subject to any law, each party must (at its own cost) do all things, execute such documents and share such information in its possession and control that is relevant, and which is reasonably necessary, to enable the other party to institute or defend (as the case may be) any demand, claim, liability or legal proceeding.

22. Sub-contracting

22.1 The Parties acknowledge that the HHS may sub-contract the provision of Health Services and other services that are required to be performed by the HHS under this Service Agreement.

22.2 The HHS must ensure that any sub-contractor who has access to confidential information (as defined in section 139 of the *Hospital and Health Boards Act 2011*) and/or personal information (as defined in section 12 of the *Information Privacy Act 2009*) complies with obligations no less onerous than those imposed on the HHS.

22.3 The HHS agrees that the sub-contracting of services:

(a) will not transfer responsibility for provision of the services to the sub-contractor; and

(b) will not relieve the HHS from any of its liabilities or obligations under this Service Agreement, including but not limited to obligations concerning the provision of data in accordance with Schedule 4 (Data Supply Requirements).

23. Counterparts

23.1 This Service Agreement may be executed in two or more identical copy counterparts, each of which together will be deemed an original, but all of which together will constitute one and the same instrument.

23.2 In the event that any signature executing this Service Agreement or any part of this Service Agreement is delivered by facsimile transmission or by scanned e-mail delivery of a '.pdf' format data file or equivalent, the signature will create a valid and binding obligation of the Party executing (or on whose behalf the signature is executed) with the same force and effect as if the signature page were an original.

23.3 For execution under this clause 23 to be valid the entire Service Agreement upon execution by each individual Party must be delivered to the remaining parties.

Execution

A. The terms of this Service Agreement were agreed under the provisions set out in the *Hospital and* Health *Boards Act,* section 35 on 27 June 2019, and were subsequently amended by the Deed of Amendment entered into pursuant to section 39 of the *Hospital and Health Boards Act 2011* and executed on 24 January 2020; 15 May 2020; 3 June 2020; [insert May 2020 Extra-ordinary Amendment Window execution date] and 29 July 2020.

B. This revised Service Agreement consolidates amendments arising from:

- 2019/20 Amendment Window 2 (in-year variation);
- 2019/20 Amendment Window 3 (in-year variation);
- 2020/21 Amendment Window 1 (annual budget build);
- April 2020 Extra-ordinary Amendment Window; and
- May 2020 Extra-ordinary Amendment Window.

C. Execution source documents are available on the service agreement website https://www.health.qld.gov.au/system-governance/health-system/managing/agreements-deeds.

Schedule 1 HHS Accountabilities

1. Purpose

Without limiting any other obligations of the HHS, this Schedule 1 sets out the key accountabilities that the HHS is required to meet under the terms of this Service Agreement.

2. Registration, credentialing and scope of clinical practice

2.1 The HHS must ensure that:

(a) all persons (including off site reporting radiologists) who provide a clinical service for which there is a national or Queensland legal requirement for registration, have and maintain current registration throughout their employment and only practise within the scope of that registration;

(b) all persons who perform roles for which eligibility for membership of a professional association is a mandatory requirement, have and maintain current eligibility of membership of the relevant professional association throughout their employment in the role; and

(c) all persons who provide a clinical service, and who fall within the scope of current credentialing policies (i.e. including medical, dental, nursing, midwifery and allied health), have a current scope of clinical practice and practise within that scope of clinical practice (which includes practising within their registration conditions and within the scope of the Clinical Services Capability Framework of the facility/s at which the service is provided).

2.2 Confirmation of registration and/or professional memberships is to be undertaken in accordance with the processes outlined in 'Health Professionals Registration: medical officers, nurses, midwives and other health professionals HR Policy B14 (QH-POL-147:2016)', as amended from time to time.

3. Clinical Services Capability Framework

3.1 The HHS must ensure that:

(a) all facilities have undertaken a baseline self-assessment against the Clinical Services Capability Framework (version 3.2);

(b) the Department is notified when a change to the Clinical Services Capability Framework baseline self-assessment occurs through the established public hospital Clinical Services Capability Framework notification process; and

(c) in the event that a Clinical Services Capability Framework module is updated or a new module is introduced, a self-assessment is undertaken against the relevant module and submitted to the Department.

3.2 The HHS is accountable for attesting to the accuracy of the information contained in any Clinical Services Capability Framework self-assessment submitted to the Department.

4. Clinical Prioritisation Criteria

4.1 The HHS must ensure that:

(a) processes for access to specialist surgical and medical services in line with Clinical Prioritisation Criteria are implemented, where these have been developed, in order to improve equity of access to specialist services; and

(b) General Practice Liaison Officer and Business Practice Improvement Officer programs are maintained in order to deliver improved access to specialist outpatient services, including through (but not limited to) their contribution to the development and implementation of Statewide Clinical Prioritisation Criteria.

5. Service delivery

5.1 The HHS will work with collaboratively with other healthcare service providers to ensure that an integrated pathway of care is in place for patients. This will include, but is not limited to:

(a) other HHSs;

(b) Primary Care providers;

(c) non-government organisations; and

(d) private providers.

5.2 The HHS must ensure that:

(a) the Health Services and other outlined in this Service Agreement, for which funding is provided in Schedule 2 ‘Funding and Purchased Activity and Services’ continue to be provided;

(b) the obligations regarding the payment and planning for blood and blood products and best practice as set out under the National Blood Agreement are fulfilled for the facilities for which funding is provided; and

(c) the *Queensland Organ Donation Strategy 2018-2020* is implemented in order to support an increase in organ donation rates in Queensland.

5.3 Through accepting the funding levels defined in Schedule 2 of this Service Agreement, the HHS accepts responsibility for the delivery of the associated programs and reporting requirements to State and Commonwealth bodies as defined by the Department.

6. Accreditation

6.1 All Queensland public hospitals, day procedure services and health care centres (howsoever titled) managed within the framework of HHSs are to maintain accreditation under the Australian Health Service Safety and Quality Accreditation Scheme[1].

6.2 Accreditation will be assessed against the National Safety and Quality Health Service standards[2] (NSQHS) second edition.

6.3 Residential aged care facilities will maintain accreditation by the Aged Care Quality and Safety Commission (ACQSC).

6.4 General practices owned or managed by the HHS are to be externally accredited in accordance with the current edition of the Royal Australian College of General Practitioners (RACGP) accreditation standards and in line with the National General Practice Accreditation Scheme.

6.5 For the purpose of accreditation, the performance of the HHS against the NSQHS and the performance of general practices owned or managed by the HHS against the RACGP accreditation standards can only be assessed by accrediting agencies that are approved by the Australian Commission on Safety and Quality in Healthcare (ACSQHC).

6.6 The HHS will select their accrediting agency from among the approved accrediting agencies. The ACSQHC and the RACGP provide a list of approved accrediting agencies which are published on their respective websites (www.safetyandquality.gov.au and www.racgp.org.au.

6.7 If the HHS does not meet the NSQHS standards accreditation requirements, the HHS has 60 days to address any not met actions. If the HHS does not meet the other accreditation standards requirements (RACGP and ACQSC), a remediation period will be defined by the accrediting agency.

6.8 Following assessment against NSQHS, ACQSC and RACGP standards, the HHS will provide to the Executive Director, Patient Safety and Quality Improvement Service, Department.

(a) immediate advice if a significant patient risk (one where there is a high probability of a substantial and demonstrable adverse impact for patients) is identified during an onsite visit, also identifying the plan of action and timeframe to remedy the issue as negotiated between the surveyors/assessors and/or the respective accrediting agency and the HHS;

(b) a copy of any 'not met' reports within two days of receipt of the report by the HHS;

(c) the accreditation report within seven days of receipt of the report by the HHS; and

(d) immediate advice should any action be rated not-met by the accrediting agency following the remediation period of an accreditation event, resulting in the facility or service not being accredited. Responsive regulatory processes may be enacted under clause 7 below.

---

[1] www.safetyandquality.gov.au/our-work/assessment-to-the-nsqhs-standards/australian-health-service-safety-and-quality-accreditation-scheme/
[2] www.safetyandquality.gov.au/our-work/assessment-to-the-nsqhs-standards/

6.9 The award recognising that the facility or service has met the required accreditation standards will be issued by the assessing accrediting agency for the period determined by their respective accreditation scheme.

6.10 The HHS will apply to an approved accrediting agency for a re-accreditation assessment prior to the expiry of their current accreditation period.

6.11 Where the HHS funds non-government organisations to deliver health and human services the HHS will ensure, from the Effective Date of this Service Agreement, that:

(a) within 12 months HHS procurement processes and service agreements with contracted non-government organisations specify the quality accreditation requirements for mental health services as determined by the Department; and

(b) as the quality accreditation requirements for subsequent funded service types are determined by the Department, procurement processes and service agreements with contracted non-government organisations reflect these requirements within 12 months of their formal communication by the Department to HHSs.

7. Responsive regulatory process for accreditation

7.1 A responsive regulatory process is utilised in the following circumstances:

(a) where a significant patient risk is identified by a certified accrediting agency during an accreditation process; and/or

(b) where an HHS has failed to address ‘not met’ actions of the specified standards within required timeframes.

7.2 An initial regulatory response will begin with a process of verifying the scope, scale and implications of the reported issues, a review of documentation, and may include one or more site visits by nominated specialty experts.

7.3 The regulatory process may include one or a combination of the following actions:

(a) seek further information from the HHS;

(b) request a progress report for the implementation of an action plan;

(c) escalate non-compliance and/or risk to the Performance Review Meeting;

(d) provide advice, information on options or strategies that could be used to address the non-met actions within a designated time frame; and/or

(e) connect the hospital to other hospitals that have addressed similar deficits or have exemplar practice in this area.

7.4 In the case of serious or persistent non-compliance and where required action is not taken by the HHS the response may be escalated. The Department may undertake one or a combination of the following actions:

(a) restrict specified practices/activities in areas/units or services of the HHS where the specified standards have not been met;

(b) suspend particular services at the HHS until the area/s of concern are resolved; and

(c) suspend all service delivery at a facility within an HHS for a period of time.

8. Achieving health equity for First Nations Queenslanders

8.1 The *Queensland Health Statement of Action towards Closing the Gap in Health Outcomes* is a commitment to addressing systemic barriers that may in any way contribute to preventing the achievement of health equity for all First Nations people. The statement is expected to mobilise renewed efforts and prompt new strategies for achieving health equity for First Nations Queenslanders.

8.2 The HHS will develop a Health Equity Strategy (previously referred to as the Closing the Gap Health Plan) to demonstrate the HHS's activities towards achieving health equity for First Nations people. The Health Equity Strategy will supersede the existing Closing the Gap Health Plan and act as the principal accountability mechanism between community and Government in the pursuit of Health Equity for First Nations Queenslanders.

8.3 The Health Equity Strategy will:

(a) be co-designed, co-developed and co-implemented by the First Nations community and the HHS; and

(b) demonstrate an evidence-based approach to priority setting.

8.4 The HHS will review the Health Equity Strategy at least once every three years and will publish the Health Equity Strategy in a way that allows it to be accessed by members of the public.

8.5 In line with the *Queensland Health Statement of Action towards Closing the Gap in Health Outcomes,* the HHS will ensure that commitment and leadership is demonstrated through implementing actions outlined in the Health Equity Strategy. The actions will, at a minimum:

(a) promote and provide opportunities to embed the representation of First Nations people in leadership, governance and the workforce;

(b) improve local engagement and partnerships between the HHS and First Nations people, communities and organisations to enable co-design, co-development and co-implementation;

(c) improve transparency, reporting and accountability in Closing the Gap progress; and

(d) demonstrate co-design, co-development, co-implementation and co-leadership of health programs and strategies.

8.6 The HHS will:

(a) establish and maintain an ongoing dialogue with the Department to progress and report on key actions taken and Health Service initiatives aligned to the *Queensland Health Statement of Action towards Closing the Gap in Health Outcomes*;

(b) establish and maintain an ongoing dialogue with the Department to progress and report on key actions taken and health service initiatives and strategies to attract,

recruit, support and retain a First Nations people workforce and workforce models commensurate to the HHS population and aligned to the benchmarks prescribed in the Workforce Diversity and Inclusion Strategy 2017-2022; and

(c) report publicly on progress against the Health Equity Strategy. Progress will be reported on an annual basis as a minimum.

9. Provision of Clinical Products/Consumables in outpatient settings

9.1 Upon discharge as an inpatient or outpatient, and where products/consumables are provided free of charge or at a subsidised charge, the Treating HHS will bear the initial costs of products/consumables provided to the patient/consumer as part of their care. These costs will be met by the Treating HHS for a sufficient period of time to ensure the patient/consumer incurs no disruption to their access to the Clinical Products/Consumables.

9.2 Unless otherwise determined by the HHS providing the Clinical Products/Consumables, ongoing direct costs (beyond an initial period following discharge as an inpatient) of the provided products/consumables will be borne by the Residential HHS of the outpatient/consumer.

9.3 Where guidelines exist (e.g. Guideline for Compression Garments for Adults with Lymphoedema: Eligibility, Supply and Costing and Guideline for Home Enteral Nutrition Services for Outpatients: Eligibility, Supply and Costing), standardised eligibility criteria and charges should apply.

9.4 Where a patient is supplied with medicines on discharge, or consequent to an outpatient appointment, that are being introduced to a patient's treatment, the Treating HHS will provide prescription(s) and an adequate initial supply. This will comprise:

(a) for medicines reimbursable under the Pharmaceutical Benefits Scheme (PBS), including the Section 100 Highly Specialised Drugs Program – the quantity that has been clinically-appropriately prescribed or the maximum PBS supply, whichever is the lesser; or

(b) for non-reimbursable medicines, one month's supply or a complete course of treatment, whichever is the lesser.

9.5 For medicines that are non-reimbursable under the PBS, and which are not included in the Queensland Health List of Approved Medicines (LAM), the Residential HHS will be responsible for ongoing supply, provided that the Treating HHS has provided the Residential HHS with documentary evidence of the gatekeeping approval at the Treating HHS for the non-LAM medicine. This evidence may be:

(a) a copy of the individual patient approval; or

(b) where the medicine is subject to a 'blanket approval' at the Treating HHS, a copy of the blanket approval, and a statement that the patient meets the criteria to be included under that approval.

9.6 This evidence is to be provided pro-actively to the Director of Pharmacy (or, for non-Pharmacist sites, the Director of Nursing and the HHS Director of Pharmacy) for the hospital nominated under clause 9.8 below.

9.7 For non-reimbursable medicines listed on the LAM for the condition being treated, the Residential HHS is responsible for ongoing supplies.

9.8 The Treating HHS will inform the patient about the ongoing supply arrangements and agree which hospital, within the patient's Residential HHS, they should attend for repeat supplies. The patient will be advised to contact the pharmacy at the nominated hospital regarding their requirements at least a week before attending for repeat supply.

9.9 PBS-reimbursable prescriptions issued by a public hospital may be dispensed at any other public hospital that has the ability to claim reimbursement. Patients may, in accordance with hospital policy, be encouraged to have their PBS prescriptions dispensed at a private pharmacy of their choice.

10. Capital, land, buildings, equipment and maintenance

10.1 **Capital**

(a) The HHS will:

(i) achieve annual capital expenditure within an acceptable variance to its allocation in the State's published Budget Paper 3 – Capital Statement, as specified in the capital expenditure performance KPI target.

(ii) record capital expenditure data in the capital intelligence portal each month. Data will be published through the System Performance Reporting (SPR) platform.

(iii) achieve all Priority Capital and Health Technology Equipment Replacement Program capital expenditure requirements and associated delivery milestones, as funded, and undertake all capital expenditure performance reporting requirements in the capital intelligence portal on a monthly basis.

(iv) comply with all other capital program reporting requirements, as identified in Schedule 4, Table 13.

10.2 **Asset Management**

(a) The Service Agreement includes funding provision for regular maintenance of the HHS's building portfolio.

(b) The Department has determined that a total sustainable budget allocation that equates to a minimum of 2.81% of the un-depreciated asset replacement value of the Queensland Public Health System's building portfolio is required to sustain the building assets to achieve expected life-cycles. The sustainable budget allocation is a combination of operational and capital maintenance funding.

(c) The HHS will conduct a comprehensive assessment of the maintenance demand for the HHS's building portfolio to ascertain the total maintenance funding

requirements of that portfolio. The assessment must identify the following for the portfolio:

(i) regulatory requirements;

(ii) best practice requirements;

(iii) condition-based requirements;

(iv) lifecycle planning requirements; and

(v) reactive maintenance estimates based on historical information, including backlog maintenance liabilities and risk mitigation strategies.

(d) The HHS will allocate an annual maintenance budget that reasonably takes into account the maintenance demand identified by the assessment in its reasonable considerations, without limiting the scope of such reasonable considerations including financial affordability linked to risk assessment. The annual maintenance budget will equate to either:

(i) 2.81% of the un-depreciated asset replacement value of the HHS's building portfolio; or

(ii) an alternative percentage amount determined by the HHSs as a result of its considerations.

(e) The HHS will submit an annual asset management and maintenance plan, approved by the Health Service Chief Executive, to the Department that:

(i) outlines the maintenance demand assessment undertaken by the HHS under Schedule 1, clause 10.2(c)

(ii) confirms the annual maintenance budget determined by the HHS under Schedule 1, clause 10.2(d)

(f) The HHS will submit an annual Statement of Building Portfolio Compliance to the Department for each year of the Term of this Service Agreement.

(g) The HHS will continue to proactively develop and address the recommendations within the final Asset Management Capability Report that was issued to the HHS as part of the transfer notice process.

10.3 **Property**

(a) The HHS will ensure building and infrastructure assets are managed in accordance with the specifications of any relevant transfer notices published as a gazette notice by the Minister under section 273A of the *Hospital and Health Boards Act 2011.*

(b) For land, buildings and parts of buildings where the Department is, or is intended to be, the exclusive occupier under specific occupancy or ground leases implemented pursuant to clauses 1.7 (c) and 1.8 respectively (where applicable) of a transfer notice, the Department is deemed to be in control of that land, building or part of a building for the purpose of work health and safety law.

10.4 Nothing in clause 10.3(b) of Schedule 1:

(a) removes any work health and safety responsibilities shared with another party or parties in accordance with work health and safety law; or

(b) limits the arrangements for the provision of work health and safety services provided in clause 11.

11. Occupational health and safety

11.1 The HHS, whether in the capacity of a Person Conducting a Business or Undertaking as an employer or as a building owner or occupier, will consult, cooperate and coordinate to ensure legal compliance with the *Work Health and Safety Act 2011* and other legislation as it applies to the scope, nature and location of operations associated with this Service Agreement. This includes, but is not limited to:

(a) codes of practice;

(b) electrical safety legislation;

(c) building and fire safety legislation; and

(d) workers' compensation legislation.

11.2 The HHS will establish, implement and maintain a health and safety management system which conforms to recognised health and safety management system standard AS/NZS 4801 *Occupational Health and Safety Management System* or ISO45001 *Occupational Health and Safety Management Systems* or another standard as agreed with the Chief Executive.

11.3 The HHS will monitor health and safety performance and will provide to the Chief Executive reports of a type, and at the intervals, agreed between the Parties, or as reasonably specified by the Chief Executive.

11.4 The Chief Executive will monitor health and safety performance at the system level. Where significant health and safety risks are identified, or performance against targets is identified as being outside tolerable levels, the Chief Executive may request further information from the HHS to address the issue(s) and/or make recommendations for action.

12. Workforce management

12.1 Subject to a delegation by the Chief Executive under section 46 of the *Hospital and Health Boards Act 2011*, the HHS is responsible for the day-to-day management (the HR Management Functions) of the Health Service Employees provided by the Chief Executive to perform work for the HHS under this Service Agreement.

(a) The HHS will exercise its decision-making power in relation to all HR Management Functions which may be delegated to it by the Chief Executive under section 46 of the *Hospital and Health Boards Act 2011*, in respect of the Health Service Employees, in a lawful and reasonable manner and with due diligence, and in accordance with:

(i) terms and conditions of employment specified by the Department in accordance with section 66 of the *Hospital and Health Boards Act 2011;*

(ii) health service directives, issued by the Chief Executive under section 47 of the *Hospital and Health Boards Act 2011*;

(iii) health employment directives, issued by the Chief Executive under section 51A of the *Hospital and Health Boards Act 2011;*

(iv) any policy document that applies to the Health Service Employee;

(v) any Industrial Instrument that applies to the Health Service Employee;

(vi) the relevant HR delegations manual; and

(vii) any other relevant legislation.

12.2 The HHS must ensure that Health Service Employees are suitably qualified to perform their required functions.

12.3 Persons appointed in an HHS as a Health Executive or Senior Health Service Employees are employees of the HHS

12.4 All HHSs will provide to the Chief Executive human resource, workforce, and health and safety reports of a type, and at the intervals, agreed between the Parties, or as reasonably specified by the Chief Executive.

13. Medically authorised ambulance transports

13.1 The HHS will:

(a) utilise the Queensland Ambulance Service (QAS) for all road ambulance services not provided by the HHS. This includes both paramedic level and patient transport level services where the patient requires clinical care;

(b) follow the *Medically Authorised Ambulance Transports Operational Standards* when utilising QAS services; and

(c) ensure that performance data for ambulance services authorised by the HHS is collected and provided to the Department in line with agreed data supply requirements.

Schedule 2 Funding, purchased activity and services

1. Purpose

This Schedule 2 sets out:

(a) The activity purchased by the Department from the HHS (Table 4, Table 6 and Table 8);

(b) The funding provided for delivery of the purchased activity (Table 4; Table 5; Table 6; and Table 7);

(c) Specific funding commitments (Table 1);

(d) The criteria and processes for financial adjustments associated with the delivery of purchased activity and specific funding commitments;

(e) The sources of funding that this Service Agreement is based on and the manner in which these funds will be provided to the HHS (Table 3); and

(f) An overview of the purchased Health Services and other services which the HHS is required to provide throughout the period of this Service Agreement.

2. Delivery of purchased activity

2.1 The Department and the HHS will monitor actual activity against purchased levels.

2.2 The HHS has a responsibility to actively monitor variances from purchased activity levels and will notify the Department immediately via the D-SA Contact Person as soon as the HHS becomes aware of significant variances.

2.3 The HHS will also notify the Department of deliberate changes to the consistent recording of activity within year that would result in additional activity being recorded for existing Health Services.

2.4 If the HHS wishes to move activity between purchased activity types and levels, for example, activity moving from outpatients to inpatients or from one inpatient Service Related Group (SRG) to another, the HHS must negotiate this with the Department based on a sound needs based rationale.

2.5 With the exception of the programs, services and projects that are specified in Table 1, during 2020/21 no financial adjustment will be applied where the HHS is unable to deliver or exceeds the activity that has been funded, in recognition of the Commonwealth Government’s treatment of the National Health Reform Agreement to support the response to the COVID-19 pandemic.

2.6 The activity purchased through this Service Agreement for 2020/21 is based on the activity purchased recurrently in 2019/20 and includes the productivity dividend.

2.7 The activity purchased in the Service Agreement for 2021/22 will be based on the activity purchased recurrently in 2020/21 including the productivity dividend.

2.8 Any resulting changes to funding and purchased activity levels will be actioned through the processes outlined in Schedules 2 and 5 of this Service Agreement.

2.9 The Department is required to report HHS activity data to the Independent Hospital Pricing Authority and the Administrator of the National Health Funding Pool. The HHS will maintain its efforts to accurately record the delivered activity and submit this data to the Department in accordance with the requirements set out in Schedule 4.

2.10 The HHS should refer to the supporting document to this Service Agreement 'Healthcare Purchasing Policy and Funding Guidelines 2020/21' for details regarding the calculation of Weighted Activity Units. Supporting documents are available on-line as detailed in Appendix 1.

3. Financial adjustments

3.1 **Specific funding commitments**

(a) As part of the Service Agreement Value, the services, programs and projects set out in Table 1 have been purchased by the Department from the HHS. These services will be the focus of detailed monitoring by the Department.

(b) The HHS will promptly notify the D-SA Contact Person if the HHS forecasts an inability to achieve commitments linked to the specific funding commitments included in Table 1.

(c) On receipt of any notice under clause 3.1(b) of Schedule 2, it is at the discretion of the Chief Executive (or delegate) to withdraw allocated funding pro rata to the level of under delivery if the program is not being delivered according to the program objective or is not being delivered in full.

(d) If the Chief Executive (or delegate) decides to withdraw allocated funding, the Chief Executive (or delegate) will immediately issue an Adjustment Notice to the HHS-SA Contact Person confirming any adjustment that has been made in accordance with this clause 3.1 of Schedule 2. The Adjustment Notice will be issued through the process set out in clause 3.4 or 3.5 of Schedule 5.

(e) Following receipt of an Adjustment Notice under clause 3.1(d) of Schedule 2, the Parties will comply with the Adjustment Notice and immediately take steps necessary to give effect to the requirements of that Adjustment Notice.

(f) The Parties acknowledge that adjustments made under this clause 3.1 of Schedule 2 may vary the Service Agreement Value and/or a specific value recorded in Table 1.

(g) Where the Service Agreement Value and/or a specific value recorded in Table 1 is varied, the variation will be recorded in a Deed of Amendment issued following the next available Amendment Window.

Table 1 Specific Funding Commitments

Service/Program/Project	Funding	Activity	Timeframe	Conditions
BreastScreen	*$3,316,568*<br><br>$4,150,011 (comprises screening activity funding; Incentive funding; fixed allocations – e.g. Biomedical Technology Services, lease of premises and 2.5% non-labour escalation)	16,000 total screens Incentive 1: 1,300 screens Incentive 2: 150 screens<br><br>15,900 total screens Incentive 1: 1,300 screens Incentive 2: 150 screens	*2019/20*<br><br>2020/21	Provision of BreastScreen services targeting women aged 50-74 years old (women 40-49 years and 75+ are also eligible, although not actively recruited).<br>Incentive funding:<br>• Incentive 1: $30 per screen for out of hours screens in the target age group 50 to 74, where BreastScreen Queensland (BSQ) Registry appointment time is before 8am on weekdays, 5pm and after on weekdays and all-day weekends; and<br>• Incentive 2: $90 per screen for 1st and 2nd screens in the target age group 50 to 74 above the 2015/16 BSQ Service specific baseline.<br>Note Incentive 1 and Incentive 2 activity is a subset of the total screening activity.<br>Funds may be withdrawn should the HHS not meet their screening target.
North Queensland Aboriginal and Torres Strait Islander Sexually Transmissible Infections Action Plan 2016-2021	*$1,696,594*<br>$1,696,594	*0*<br>0	*2019/20*<br>2020/21	The HHS will implement and support the required actions under the *North Queensland Aboriginal and Torres Strait Islander Sexually Transmissible Infections Action Plan 2016-2021* (the Plan) including the delivery of initiatives and outcomes outlined in memorandum CECTF-19/5767.<br>Funding may be adjusted and/or unspent funds redirected or recovered where project performance requirements are not met.
• Contact Tracing Support Officer position	*$73,800*<br>$75,720	*0*<br>0	*2019/20*<br>2020/21	Progress with contact tracing is to be reported through the Plan.<br>If the position is not fully operational between 1 November 2018 and 30 June 2021, the HHS is to return unspent labour costs to the Communicable Diseases Branch.

Service/Program/Project	Funding	Activity	Timeframe	Conditions
Cairns South Health Precinct	$3,244,839<br>$4,603,687 (recurrent)	0<br>TBC	2020/21<br>2021/22	Part-year operational funding to support 12 renal chairs based at Cairns South Health Precinct.<br>Funding is calculated on an agreed start date of 1 September 2020 and may be subject to adjustment should the service not be fully operational by this date.<br>Continued funding is subject to assessment by the Department of an implementation review to be delivered by the HHS no later than 28 February 2021. The implementation review must include evaluation of patient profile, models of care, workforce allocation, facility costs and potential revenue streams along with other relevant topics determined by the HHS.<br>Activity may be added once the service is established.
Making Tracks towards closing the gap in health outcomes for Indigenous Queenslanders by 2033 - Investment Strategy 2018-21 including the *Queensland Aboriginal and Torres Strait Islander Rheumatic Heart Disease Action Plan 2018-2021*	*$2,754,511*<br>$2,554,511	*0*<br>0	*2019/20*<br>2020/21	The HHS will deliver the initiatives and outcomes outlined in the performance requirements issued by the Aboriginal and Torres Strait Islander Health Division in memorandum C-ECTF-19/5767.<br>The HHS will implement and support the required actions under the *Queensland Aboriginal and Torres Strait Islander Rheumatic Heart Disease Action Plan 2018-2021* and outlined in the memorandum C-ECTF-19/5767.<br>Funding may be adjusted and/or unspent funds redirected or recovered where project performance requirements are not met.
• Making Tracks: Collaboration in Health (MaTCH)	*$55,300*	*0*	*2019/20*	The HHS will deliver the initiatives and outcomes outlined in the performance requirements issued by the Aboriginal and Torres Strait Islander Health Division in memorandum C-ECTF-20/1314.<br>Funding may be adjusted and/or unspent funds redirected or recovered where project performance requirements are not met.

Service/Program/Project	Funding	Activity	Timeframe	Conditions
Connecting Care to Recovery 2016-2021: A plan for Queensland's Mental Health				
• Independent Patient Rights Advisers	$356,000 (recurrent)	*0*	*2017/18*	Independent Patient Rights Advisors are to be employed or engaged in accordance with *the Mental Health Act 2016* and the Chief Psychiatrist Policy on *Independent Patient Rights Advisors*.
• Adult Step Up Step Down Unit (SUSD)	$271,750 (recurrent)<br>$1,234,690 (recurrent)	*0*<br>*0*	*2018/19*<br>*2019/20*	Additional 2.0 Full Time Equivalents to transition the Adult Prevention and Recovery Centre to an SUSD in line with model of service.
• Youth Step Up Step Down Unit (Y-SUSD)	$476,904 (recurrent)	*0*	*2018/19*	Operational funding (clinical) for Y-SUSD
• Ed-LinQ	*$162,500*	*0*	*2019/20*	Expand the reach of the existing Ed-LinQ program in Cairns and Hinterland HHS.
Prisoner health services - revised base funding	$7,943,501 (recurrent)	*0*	*2019/20*	The HHS will use this funding to provide primary health care services for prisoners in Lotus Glen Correctional Centre in accordance with the Memorandum of Understanding established between Queensland Health and Queensland Corrective Services.
Community Mental Health Growth Allocation	$811,800 (recurrent)	*0*	*2018/19*	Provision of funding to employ 6 additional Full Time Equivalents (FTEs) in support of the HHSs initiatives to enhance Older Persons Community Mental Health Services.<br>Recruitment of FTEs to be monitored by the Mental Health Alcohol and Other Drugs Branch on a regular basis using the Mental Health Establishment Collection, with adjustments to be made in-year if FTEs not all recruited permanently.

Service/Program/Project	Funding	Activity	Timeframe	Conditions
Hospital Car Parking Concession Scheme	$10,000 (recurrent)<br><br>*$25,000*<br>$25,000* (recurrent)	*0*<br><br>*0*<br>0	*2019/20*<br><br>*2019/20*<br>2020/21	If program performance requirements are not met in-year funding may be withdrawn.<br>Monthly reporting of car parking concessions data on ‘Car Parking Concessions Reporting template’ required.<br>Annual Hospital and Health Service Car Parking Concessions Criteria reporting required.<br>*Top-up to assist with administrative costs.
Evolve Therapeutic Services (ETS)	*$1,657,942*	*0*	*2019/20*	Provision of ETS within allocated resources and in line with the state-wide ETS Manual, noting variation in local contexts.<br>Reporting requirements as defined by the Mental Health, Alcohol and Other Drugs Branch.<br>If program performance requirements are not met in-year funding may be adjusted proportional to the under delivery against the agreed target.
High risk foot patients seen/managed within 48 hours of referral to ambulatory services	$260,923 (recurrent)<br><br>$184,821 (recurrent)<br><br>$108,718 (recurrent)	*56 WAUs (Q21)*<br><br>*38 WAUs (Q22)*<br><br>*22 WAUs (Q21)*	*2018/19*<br><br>*2019/20*<br><br>*2018/19*	The HHS will provide services as specified in the 2019/20 Ambulatory High-Risk Foot Services specification sheet published on QHEPS.<br>This recurrent allocation is to fund a full-time clinical position to support high risk foot services in other HHSs across Queensland via telehealth.

Service/Program/Project	Funding	Activity	Timeframe	Conditions
Oral Health Services	$13,514,109 (recurrent)	261,175 WOOS	2020/21	Delivery consistent with the Oral Health Policy Framework.<br>Funding does not include Commonwealth National Partnership Agreement funding.<br>Funding may be adjusted where the total oral health activity delivered varies from the purchased levels.<br>Oral health activity (WOOS) for the 0-15 year age group shall not be less than that achieved in 2017/18.<br>Oral health activity (WOOS) includes activity claimed under the Child Dental Benefits Scheme but excludes general dental treatment delivered under general anaesthetic in public hospitals.
• National Partnership Agreement (NPA) on Adult Public Dental Services – First Nations	$144,073	2,620 WOOS	2020/21	Queensland is required to meet two performance targets during 2020/21, which are the 30 September 2020 target (for 1 April to 30 September 2020) and the 31 March 2021 target (for 1 October 2020 to 31 March 2021). HHSs must collectively meet these targets.<br>Funding may be adjusted where the total oral health activity delivered varies from the purchased levels.
Another 100 Midwives (Nursing)	*$691,658*<br>$709,536<br>$337,117	*0*<br>0<br>0	*2019/20*<br>2020/21<br>2021/22	The HHS will deliver the initiatives and outcomes outlined in the performance requirements as per memo C-ECTF-18/8074. Funding may be withdrawn if requirements are not met.

Service/Program/Project	Funding	Activity	Timeframe	Conditions
Nurse Navigators	*$3,360,934*<br>$3,468,328<br>$3,468,328<br>$3,468,328 (recurrent)	*0*<br>0<br>0<br>0	*2019/20*<br>2020/21<br>2021/22<br>2022/23	The total Nurse Navigator Program allocation (2015/16 – 2019/20) is 19 NG7 and 1 NG8. All Nurse Navigator Program Full Time Equivalent (FTE) is required to be appointed to a position ID that has 'Nurse Navigator' within the position title.<br>The HHS is ineligible to appoint Nurse Navigator Program FTE to any pre-existing permanent positions which have been renamed to include 'Nurse Navigator' in the position title.<br>The HHS is required to report monthly on:<br>• Employed Nurse Navigator FTE;<br>• Number of Nurse Navigator plans in place; and<br>• Number of patients seen by Nurse Navigators.
COVID-19 First Nations Response	$3,881,831	0	2020/21	The HHS will implement and deliver the required actions under the HHS First Nations COVID-19 response including the delivery of initiatives and outcomes outlined in memorandum C-ECTF-20-9652.<br>Funding is one-off in nature for discrete and time-limited activities that are directly attributable to managing the impacts of COVID-19 for First Nations peoples and must be in-scope under the existing financial guidelines for COVID-19 expenditure.<br>HHSs are to retain appropriate supporting documentation to substantiate all expenditure under the National Partnership Agreement.
Specialist Outpatient Strategy<br>• General Practitioner with a Special Interest (GPwSI)	$315,310 (recurrent)	33 WAUs (Q22)	2020/21	The HHS will deliver the initiatives and outcomes outlined in memo C-ECTF-19/6469.

Service/Program/Project	Funding	Activity	Timeframe	Conditions
Care in the Right Setting (CaRS):<br>• Specialist Palliative Care Services Clinical Support<br>• Fast-Track Pain Management<br>• Better Health North Queensland: Regional Healthcare in the Home	*$174,000*<br>$591,446<br>*$118,000*<br>*$761,500*	*0*<br>0<br>*0*<br>*0*	*2019/20*<br>2020/21<br>*2019/20*<br>*2019/20*	Services will be provided consistent with the CaRS application(s).<br>If Service commencement does not align with the agreed implementation timeframes funding may be withdrawn on a pro-rata basis.<br>If the agreed Service levels are not provided, funding may be withdrawn.<br>Activity levels will be monitored regularly and cooperation with external evaluators is required.<br>Where the Service includes Service provision to another (receiving) HHS:<br>• If staffing is not available within the HHS to meet the agreed Service levels, the HHS will make alternate arrangements to ensure that the agreed Service levels are provided; and<br>• If the agreed Service levels are not provided, funding may be withdrawn and provided to the receiving HHS.
Residential aged care facility Support Services (RaSS)	*$130,000*<br>$300,000	*0*<br>0	*2019/20*<br>2020/21	The HHS will deliver the initiatives and outcomes as outlined in the RaSS and Hospital in The Home COVID-19 Funding - memorandum C-ECTF 20/4081.<br>The funding allocated is linked to the National Partnership Agreement on COVID-19 healthcare response and as such it will be a requirement of the HHS to capture accurate activity and expenditure of services delivered under this program. Unspent funds may be withdrawn and returned to the department.
Hospital in The Home (HITH)	*$720,000*<br>$600,000	*148 WAUs*<br>124 WAUs	*2019/20*<br>2020/21	The HHS will deliver the initiatives and outcomes as outlined in the Residential Aged Care Facility Support Services and HITH COVID-19 Funding - memorandum C-ECTF 20/4081.<br>The HHS will provide a reconciliation of expenditure through End of Year, and unspent funds may be withdrawn and returned to the department.

Service/Program/Project	Funding	Activity	Timeframe	Conditions
Safe and healthy drinking water in Indigenous Local Government areas	*$1,696,030*<br>$1,859,521<br>$1,935,733<br>$1,969,243<br>$2,007,377	*0*<br>0<br>0<br>0<br>0	*2019/20*<br>2020/21<br>2021/22<br>2022/23<br>2023/24	Funding is provided to work with the local government areas covering Aboriginal and Torres Strait Islander discrete communities to improve the capacity of water treatment plant operators such that they can provide a safe and continuous supply of drinking water to their communities.<br>Once sustained improvement in the safety and continuity of the drinking water supply is achieved in the identified Council areas, capacity building should extend to the areas of waste water and solid waste.<br>The HHS will also:<br>• Provide training and mentoring to project officers employed by Townsville HHS, Central Queensland HHS and Darling Downs HHS;<br>• Deliver an annual workshop for Indigenous water treatment plant operators; and<br>• Lead the development of a culturally appropriate training package for Indigenous water treatment plant operators equivalent to the existing Certificate III in Water Operations (with input from the Water Unit, Townsville HHS, Central Queensland HHS and Darling Downs HHS).<br>Funding may only be used for the purposes of the program and the Department may recover or redirect unspent funding.

Service/Program/Project	Funding	Activity	Timeframe	Conditions
Enterprise Bargaining (EB)	*$22,113,559*<br>$6,472,869<br>$1,916 (comprises both recurrent and non-recurrent funding)	*0*<br>0<br>0	*2019/20*<br>2020/21<br>2021/22	Funding has been allocated in full for the following EB agreements:<br>• *Nurses and Midwives (Queensland Health and Department of Education and Training) Certified Agreement (EB10) 2018* (Base wages and certain entitlements); and<br>• *Medical Officers (Queensland Health) Certified Agreement (MOCA5) 2018.*<br>Legislative amendments have been introduced under the *Industrial Relations Act 2016* to give effect to a 2.5% increases under the following agreements as new agreements are yet to be certified:<br>• *Queensland Public Health Sector Certified Agreement (No.9) 2016;*<br>• *Queensland Health, Building, Engineering & Maintenance Services Certified Agreement (No.6) 2016;* and<br>• *Health Practitioners' and Dental Officers (Queensland Health) Certified agreement (No. 2) 2016.*<br>Funding which has been allocated recurrently in previous years has been recalled for the following streams as wage increase are not yet approved:<br>• HES-DSO;<br>• SES-SO; and<br>• VMO.<br>Subject to the terms and conditions of the agreements once executed a funding adjustment may be required. Full details can be found on the Budget and Analysis SharePoint platform.
• Nurses and Midwives EB10 Innovation Fund – Reviving Rural Dementia Care Project	*$183,560*<br>$88,131	*0*<br>0	*2019/20*<br>2020/21	Funding has been provided under the *Nurses and Midwives (Queensland Health and Department of Education) Certified Agreement (EB10) 2018,* clause 44.6, Innovation Fund.<br>The HHS will deliver the project, evaluation and reporting as outlined in memo C-ECTF-19/8776.<br>Funding may be withdrawn if project requirements are not met.

Service/Program/Project	Funding	Activity	Timeframe	Conditions
Rapid Results Program<br>*Delivering what matters: Frail and Older persons initiative*<br>• RACF support services (RaSS)<br>• Geriatric Emergency Department Intervention (GEDI)	$600,000 (recurrent)<br>$600,000 (recurrent)	*0*<br>*0*	*2019/20*<br>*2019/20*	Funding is provided to implement the core principals of a Residential Aged Care Facility Acute Care Support Service and Geriatric Emergency Department Intervention through a hybrid model of care at Cairns Hospital from 1 July 2019.<br>The HHS will:<br>• Establish the service in line with agreed timelines;<br>• Establish a Steering Committee to provide oversight of the service development and operation;<br>• Comply with the agreed reporting requirements, including progress against the identified project outcomes and performance measures; and<br>• Participate in learning sessions and statewide working group meetings.<br>If these conditions are not met or if Dedicated Frail Older Persons models of care are ceased, funding may be withdrawn.
Rapid Results Program<br>*Delivering what matters: Advancing Kidney Care 2026 Collaborative*	$1,178,800 (recurrent)	*126 WAUs (Q22 part WAU backed)*<br>253 WAUs (Q22 fully WAU backed)	*2019/20*<br>2020/21	Funding is provided for transplant coordination, vascular access coordination, supported home haemodialysis and kidney supportive care under the *Advancing Kidney Care 2026 Collaborative*.<br>The HHS will ensure that the reporting requirements established for this initiative are met, including the provision of quarterly progress against agreed implementation milestones and outcome measures.

Service/Program/Project	Funding	Activity	Timeframe	Conditions
Rapid Results Program<br>*Delivering what matters: Networked Cardiac Services*	$1,453,354 (recurrent)<br>*$165,000 (non-recurrent)*	*12 WAUs (Q22)*	*2019/20*	Funding is provided for the provision of Networked Cardiac Services.<br>The HHS will:<br>• Meet the Service implementation timeframes and provide the Service levels established within the agreed business case;<br>• Provide Services to Torres and Cape HHS as established within the agreed business case; and<br>• Utilise the Queensland Cardiac Outcomes Registry outreach data module for all data capture and reporting.<br>If Service commencement does not align with the agreed implementation timeframes funding may be withdrawn on a pro-rata basis.<br>If staffing is not available within the HHS to meet the agreed Service levels, the HHS will make alternate arrangements to ensure that the agreed Service levels are provided.<br>If the agreed Service levels are not provided, funding may be withdrawn and provided to Torres and Cape HHS.<br>Activity levels will be monitored quarterly.

Service/Program/Project	Funding	Activity	Timeframe	Conditions
Planned Care Volume Targets – Elective Surgery	$52,417,948 (Funding in existing service agreement)	7,694 elective surgery separations aligned with the elective surgery data collection, as reported on SPR and any outsourced elective surgery activity. 10,815 WAUs (Q22).	2020/21	Funding has been quarantined to support the ongoing delivery of planned care volumes during 2020/21. Quarantined funds have been determined based on agreed average Q22 WAUs for 7,694 Elective Surgery Separations (volume target).<br>Volume targets to be reviewed and adjusted through the amendment window process to include any additional planned care investments.<br>Performance will be monitored quarterly with reconciliation of the agreed planned care activity targets (number of day case and overnight treated patients).<br>Should the agreed volume targets not be met, funding will be withdrawn at the calculated HHS average Q22 WAUs **per separation not delivered**:<br>Example:<br>1 Case = 1.41 Q22 WAUs or<br>$6,813 (QEP)<br>There will not be claw back of under delivery against Q22 WAUs target if the volume of elective day case and overnight separations has been delivered.

Service/Program/Project	Funding	Activity	Timeframe	Conditions
Planned Care Volume Targets – Gastrointestinal Endoscopy (GIE)	$12,686,251 (Funding in existing service agreement value)	5,279 Gastrointestinal Endoscopies aligned with the Gastrointestinal Endoscopy data collection, as reported on SPR and any outsourced GIE activity. 2,617 WAUs (Q22).	2020/21	Funding has been quarantined to support the ongoing delivery of planned care volumes during 2020/21. Quarantined funds have been determined based on agreed average Q22 WAUs for 5,279 Gastrointestinal Endoscopy Separations (volume target).<br>Volume targets to be reviewed and adjusted through the amendment window process to include any additional planned care investments.<br>Performance will be monitored quarterly with reconciliation of the agreed planned care activity targets (number treated GIE patients).<br>Should the agreed volume targets not be met, funding will be withdrawn at the calculated HHS average Q22 WAUs per separation not delivered:<br>Example:<br>1 Case = 0.5 Q22 WAUs or<br>$2,403 (QEP)<br>There will not be claw back of under delivery against Q22 WAUs target if the volume of GIE day case and overnight separations has been delivered.

Service/Program/Project	Funding	Activity	Timeframe	Conditions
Planned Care Volume Targets – Specialist Outpatients	$11,376,460 (Funding in existing service agreement value)	39,908 Specialist Outpatient initial service events as per the funding specification, and outsourced activity. 2,347 WAUs (Q22).	2020/21	Funding has been quarantined to support the ongoing delivery of planned care volumes during 2020/21. Quarantined funds have been determined based on agreed average Q22 WAUs for 39,908 Initial Service Events (volume target).<br>Volume targets to be reviewed and adjusted through the amendment window process to include any additional planned care investments.<br>Performance will be monitored quarterly with reconciliation of the agreed planned care activity targets (number of initial service events).<br>Should the agreed volume targets not be met, funding will be withdrawn at the calculated HHS average Q22 WAUs per initial service event not delivered:<br>Example:<br>1 Case = 0.059 Q22 WAUs or<br>$285 (QEP)<br>There will not be claw back of under delivery against Q22 WAUs target if the volume of initial service events has been delivered.

3.2 **National Partnership on COVID-19 Response**

(a) The Department will provide additional funding to the HHS under the State Public Health Payment component of the *National Partnership on COVID-19 Response* where actual additional cost is demonstrated.

(b) Funding will be provided where the HHS has:

(i) undertaken activity that is in-scope for the State Public Health Payment during the reporting period; and

(ii) met the reporting requirements in relation to COVID-19 expenditure, as prescribed under the *National Partnership on COVID-19 Response* and by the Department.

(c) The scope of the State Public Health Payment is defined as:

(i) additional costs that are attributable to the treatment of patients with diagnosed or suspected COVID-19; or

(ii) additional costs of activities directed at preventing the spread of COVID-19.

(d) Additional costs that are reimbursed through the State Public Health Payment will be excluded from the calculation of activity eligible for funding under the terms of the National Health Reform Agreement.

(e) There is no guarantee that the HHS will have access to additional funding under the State Public Health Payment.

(f) All funding that is provided through the State Public Health Payment will be subject to a reconciliation to be undertaken by the Administrator of the National Health Funding Pool. The HHS will be required to provide evidence to support claims for additional costs. If the HHS is unable to provide the required evidence on request, funding received may be recalled subject to reconciliation.

(g) Funding adjustments will be actioned through the process set out in clause 3.4 of Schedule 5 of this Service Agreement.

3.3 **Financial adjustments – other**

(a) The purchasing approach includes a range of funding adjustments which aim to incentivise high value care, that is care which delivers the best outcomes at an efficient cost, and dis-incentivise Low Benefit Care. This includes incentive payments for HHS who achieve quality targets in specific areas of priority. The purchasing incentives that apply to this Service Agreement are detailed in Table 2.

(b) The Department must reconcile the applicable purchasing incentives in Table 2 in line with the timeframes specified in the purchasing specification sheet included within the supporting document 'Purchasing Policy and Funding Guidelines 2020/21'. The Department must promptly provide a copy of the reconciliation statement to the HHS-SA Contact Person.

(c) Funding adjustments must be based on the requirements contained in the relevant specification sheet for that purchasing incentive.

(d) If the Parties are unable to reach agreement in relation to any funding adjustments that are identified, the provisions of clause 14 in the standard terms of this Service Agreement will apply to resolve the dispute.

(e) When the Parties have agreed on a funding adjustment, the Chief Executive (or delegate) will issue an Adjustment Notice to the HHS-SA Contact Person confirming any funding adjustment that has been made in accordance with this clause 3.3 of Schedule 2. The Adjustment Notice will be issued through the process set out in clause 3.4(c) of Schedule 5.

(f) Following receipt of an Adjustment Notice under clause 3.4(c) of Schedule 5, the Parties will comply with the Adjustment Notice and immediately take the steps necessary to give effect to the requirements of that Adjustment Notice.

(g) The Parties acknowledge that the funding adjustments may vary the Service Agreement Value recorded in Schedule 2. Where the Service Agreement Value recorded in Schedule 2 is varied, the variation will be recorded in a Deed of Amendment issued following the next available Amendment Window.

Table 2 Purchasing Incentives 2020/21 (Summary)

Incentive	Description	Scope	Status for 2020/21	Funding Adjustment
Quality Improvement Payment (QIP) – Antenatal Visits for First Nations Women	Incentive payments for achieving targets for:<br>• First Nations women attending an antenatal session during their first trimester, and attending at least 5 antenatal visits; and<br>• First Nations women stopping smoking	All HHSs (excluding Children's Health Queensland)	Continues as per 2019/20 with new targets	50% advance payments made to HHSs with balance paid retrospectively based on performance.
Quality Improvement Payment (QIP) - Smoking Cessation (Community Mental Health)	Incentive payments for achieving targets for community mental health patients clinically supported onto the Smoking Cessation Clinical Pathway	All HHSs (excluding Children's Health Queensland and Mater Public Hospitals)	Continues as per 2019/20 with new targets	Paid retrospectively
High Cost Home Support Program	Payment for high cost 24 hour home ventilated patients meeting the eligibility criteria, where funding is not available through existing sources	All HHSs	Continues as per 2019/20	Paid retrospectively based on forecast costs
Telehealth	Incentive payments for additional outpatient activity volume, provision of telehealth consultancy for Inpatients, Emergency Department and Outpatients episodes and Store and Forward assessments	Inpatients, Emergency Department, Outpatients, and Store and Forward - all HHSs	Continues as per 2019/20 with Outpatients scope expanded to include rural and remote facilities across all HHSs	Paid retrospectively
Sentinel Events	Zero payment for national sentinel events	All ABF public hospitals	Continues as per 2019/20	Retrospective adjustment

3.4 Public and private activity/Own Source Revenue

(a) Own Source Revenue comprises Grants and Contributions, User Charges and Other Revenues.

(b) Where an HHS is above its Own Source Revenue target in respect of private patients, it will be able to retain the additional Own Source Revenue with no compensating adjustments to funding from other sources.

(c) Conversely where an HHS is below its Own Source Revenue target in respect of private patients, it will experience a reduction in revenue with no compensating adjustments to funding from other sources.

(d) The HHS will make all efforts to identify and maximise revenue from appropriate third parties to ensure optimisation of available State and Commonwealth funding across the Queensland Public Sector Health System.

(e) The Own Source Revenue identified in Table 3 is an estimate generated by the HHS which allows all third-party funding sources associated with service delivery

to be identified. The HHS will ensure that this estimate is substantiated and accurate to ensure no significant variances to actual revenue generated.

(f) The HHS will routinely revise and update the estimate to ensure alignment between the Service Agreement and Queensland Treasury's reporting system (TRIDATA).

(g) Budget adjustments for changes in Own Source Revenue from private patients will be actioned through the process set out in Schedule 5 of this Service Agreement.

4. Funding sources

4.1 The four main funding sources contributing to the HHS Service Agreement value are:

(a) Commonwealth funding;

(b) State funding;

(c) Grants and Contributions; and

(d) Own Source Revenue.

4.2 Table 3 provides a summary of the funding sources for the HHS and mirrors the total value of the Service Agreement included in Table 4.

Table 3 Hospital and Health Service funding sources 2020/21

Funding Source	Value ($)
**NHRA Funding**	
Activity Based Funding	738,524,229
Clinical Education and Training[3]	-24,011,148
Own Source Revenue contribution in ABF funded services	-22,945,135
**Pool Account – ABF Funding (State and Commonwealth)[4]**	**691,567,946**
Block Funding	124,271,795
Clinical Education and Training[3]	24,011,148
**State Managed Fund – Block Funding (State and Commonwealth)[5]**	**148,282,943**
**Locally Receipted Funds (Including Grants)**	**12,440,356**
**Locally Receipted Own Source Revenue (ABF)**	**22,945,135**
**Locally Receipted Own Source Revenue (Other activities)**	**60,253,093**
**Department of Health Funding[6]**	**85,879,219**
**Total NHRA Funding**	**1,021,368,692**
**NPA Covid-19 Response**	
Activity Based Funding	-
**Hospital Services Payment – ABF Funding (State and Commonwealth)[7]**	**-**
Block Funding	-
Clinical Education and Training[3]	-
**Hospital Services Payment – Block Funding (State and Commonwealth)[5]**	**-**
**Public Health Funding (State and Commonwealth) [8]**	**4,181,832**
**Total NPA – COVID-19 Funding**	**4,181,832**
**TOTAL**	**1,025,550,524**

[3] Clinical Education and Training (CET) is classified as Teaching, Training and Research Funding under the National Model and funded as a Block Funded Service. Under the State Model, CET is included as 'Other ABF' and forms part of the ABF total. To comply with the requirements of the National Health Reform Agreement, funding must be paid as it is received, therefore from a Funding Source perspective, CET has been reclassified to Block Funding.

[4] Pool Account - ABF Funding (State and Commonwealth) includes: Inpatient; Critical Care; Emergency Department; Sub and Non Acute; Mental Health; and Outpatient activities each allocated a proportion of Other ABF Adjustments.

[5] State Managed Fund - Block Funding (State and Commonwealth) includes: block funded hospitals; standalone specialist mental health hospitals; community mental health; and teaching, training and research.

[6] Department of Health Funding represents funding by the Department for items not covered by the National Health Reform Agreement including such items as: Prevention, Promotion and Protection; Depreciation, and other Health Services.

7 Hospital Services Payment - Funding provided under the COVID-19 National Partnership Agreement for activity that is attributable to the diagnosis and treatment of Medicare eligible patients with COVID-19 or suspected of having COVID-19; elective surgeries that have been rescheduled to free up public hospital capacity to respond to the COVID-19 outbreak; and may include activities related to the care of public patients being treated in private hospitals.

[8] Public Health Payment - Funding provided under the COVID-19 National Partnership Agreement for the State public health system's activity attributable to the treatment of patients with diagnosed or suspected COVID-19 or for activities directed at preventing the spread of COVID-19.

5. Funds disbursement

5.1 The Chief Executive will direct the disbursement of both State and Commonwealth funding from the State's National Health Funding Pool Sub Account and the State Managed Fund to the HHS. The Service Agreement and State level block payments to State managed funds from Commonwealth payments into the national funding pool are stated in Table 8.

5.2 However, the State (represented by the Chief Executive) will not:

(a) redirect Commonwealth payments between HHSs;

(b) redirect Commonwealth payments between funding streams (e.g. from ABF to Block Funding); and/or

(c) adjust the payment calculations underpinning the Commonwealth's funding.

5.3 Payment of Activity Based Funding and Block Funding to the HHS will be on a fortnightly basis.

5.4 Further information on the disbursement of funds is available in the supporting document to this Service Agreement 'Purchasing Policy and Funding Guidelines 2020/21'.

Table 4 HHS Finance and Activity Schedule 2019/20 – 2021/22 – Summary by Purchasing Hierarchy

	ABF/NABF	ABF_SPLIT	Service Stream	2019/20 QWAU (Q22)	2019/20 Funding ($) (Price: $4847)	2020/21 QWAU (Q22)	2020/21 Funding) (Price: $4847)	2021/22 QWAU (QTBC)	2021/22 Funding (Price: $TBC)
Allocations excluding COVID-19	ABF	ABF	Inpatients	77,606	$352,161,660	78,650	$355,458,085		
			Outpatients	17,572	$85,982,318	17,744	$84,714,633		
			Procedures & Interventions	16,225	$74,417,263	16,105	$73,502,023		
			Emergency Department	18,382	$84,764,145	18,643	$85,186,650		
			Sub & Non-Acute	6,250	$28,783,492	6,346	$29,564,821		
			Mental Health	6,534	$30,053,374	6,634	$30,608,569		
			Prevention & Primary Care	3,286	$16,864,036	3,424	$17,808,227		
			Other ABF $	0	$32,269,112	0	$33,889,960		
		**ABF Total**		**145,855**	**$705,295,401**	**147,546**	**$710,732,967**		
		ABF Other	CET Funding	0	$23,849,816	0	$23,761,290		
			Specified Grants	0	$3,495,644	0	$4,029,972		
			PPP	0	$0	0	$0		
			EB Quarantined	0	$6,415,688	0	$0		
		**ABF Other Total**		**0**	**$33,761,148**	**0**	**$27,791,262**		
	Other Funding	Other Funding	Block Funded Services	12,625	$58,614,763	12,625	$60,916,891		
			Population Based Community Services	0	$91,775,364	0	$90,551,436		
			Other Specific Funding	0	$109,419,676	0	$109,362,963		
			PY Services moved to ABF	0	$0	0	$0		
			Prevention Services – Public Health	0	$20,863,637	0	$22,013,172		
		**Other Funding Total**		**12,625**	**$280,673,441**	**12,625**	**$282,844,463**		
	**Allocations excluding COVID-19 TOTAL**			**158,480**	**$1,019,729,991**	**160,171**	**$1,021,368,692**		

	ABF/NABF	ABF_SPLIT	Service Stream	2019/20 QWAU (Q22)	2019/20 Funding ($) (Price: $4847)	2020/21 QWAU (Q22)	2020/21 Funding) (Price: $4847)	2021/22 QWAU (QTBC)	2021/22 Funding (Price: $TBC)
**COVID-19 related allocations**	**ABF**	ABF	Inpatients	149	$720,000	0	$0		
			Outpatients	0	$300,000	0	$0		
			Procedures & Interventions	0	$0	0	$0		
			Emergency Department	0	$0	0	$0		
			Sub & Non-Acute	0	$0	0	$0		
			Mental Health	0	$0	0	$0		
			Prevention & Primary Care	0	$0	0	$0		
			Other ABF $	0	$0	0	$0		
		**ABF Total**		**149**	**$1,020,000**	**0**	**$0**		
		ABF Other	CET Funding	0	$0	0	$0		
			Specified Grants	0	$0	0	$0		
			PPP	0	$0	0	$0		
			EB Quarantined	0	$0	0	$0		
		**ABF Other Total**		**0**	**$0**	**0**	**$0**		
	**Other Funding**	Other Funding	Block Funded Services	0	$130,000	0	$300,000		
			Population Based Community Services	0	$0	0	$0		
			Other Specific Funding	0	$5,000,051	0	$3,881,831		
			PY Services moved to ABF	0	$0	0	$0		
			Prevention Services – Public Health	0	$0	0	$0		
		**Other Funding Total**		**0**	**$5,130,051**	**0**	**$4,181,831**		
	**COVID-19 Allocations TOTAL**			**149**	**$6,150,051**	**0**	**$4,181,831**		
**Grand Total**				**158,629**	**$1,025,880,042**	**160,171**	**$1,025,550,523**		

Table 5 Minor Capital and Equity

	2019/20 $	2020/21 $	2021/22 $
**Minor Capital & Equity**			
**Cash**			
CAH-Oct16-39 - Mossman Midwifery Group Practice	$0	$0	
SA 16-17.320 - Minor Capital funding Allocation 2016-17	$3,096,000	$3,096,000	
CAH-EoY1617-17 - BreastScreen Queensland - Capital A	$0	$0	
CAH-EoY1617-18 - Breastscreen Queensland- Capital B	$0	$0	
CAH-AW2-Oct17-24 NTFEP - eyeConnect	$0	$0	
CAH-AW3-Feb18-31 NTFEP - eyeConnect	$0	$0	
CAH-AW2-Oct18-22 BreastScreen Van for Cairns	$0	$0	
CAH-AW3-Feb19-14 Emergency Department - Capital	$0	$0	
CAH-AW2-OCT19-05 Breastscreen Van for Cairns	$850,000	$0	
CAH-AW3-FEB20-09 Lease funding swap per changes to AASB16 - equity component	$482,187	$0	
CAH-BB2021-45 Lease funding swap per changes to AASB16 - equity component	$0	$400,494	
**Non-Cash**			
-	-	-	
**Grand Total**	**$4,428,187**	**$3,496,494**	

Table 6 HHS Finance and Activity Schedule 2019/20 – 2021/22 Other Funding Detail

Agreement		Service Stream	Category	2019/20 $	2020/21 $	2021/22 $
Allocations excluding COVID-19	Other Funding	Block Funded Services	Block Funded Services	$58,614,763	$60,916,891	
		**Block Funded Services Total**		**$58,614,763**	**$60,916,891**	
		Population Based Community Services	Alcohol, Tobacco & Other Drugs	$4,558,721	$3,901,373	
			Community Care Programs	$1,855,634	$1,875,555	
			Community Mental Health	$20,325,645	$20,518,952	
			Community Mental Health – Child & Youth	$14,065,950	$12,834,816	
			Other Community Services	$9,740,983	$10,183,663	
			Other Funding Subsidy/(Contribution)	$37,307,041	$37,307,041	
			Primary Health Care	$3,921,390	$3,930,036	
		**Population Based Community Services Total**		**$91,775,364**	**$90,551,436**	
		Other Specific Funding	Aged Care Assessment Program	$1,270,418	$952,813	
			Commercial Activities	$0	$0	
			Consumer Information Services	$0	$0	
			Depreciation	$55,632,000	$56,274,001	
			Disability Residential Care Services	$3,623	$0	
			Environmental Health	$1,142,565	$224,574	
			Home & Community Care (HACC) Program	$5,447,000	$5,447,000	
			Home & Community Medical Aids & Appliances	$115,482	$40,482	
			Home Care Packages	$0	$0	
			Interstate Patients	$4,606,598	$4,606,598	
			Multi-Purpose Health Services	$2,686,733	$2,686,733	
			Prisoner Health Services	$7,943,501	$7,943,501	
			Oral Health	$0	$0	
			Patient Transport	$12,416,504	$12,416,504	
			Research	$703,817	$703,817	
			Residential Aged Care	$11,962	$11,962	
			Specific Allocations	$12,076,773	$12,692,279	
			State-Wide Functions	$2,441,939	$2,441,939	
			Transition Care	$2,920,761	$2,920,761	
		**Other Specific Funding Total**		**$109,419,676**	**$109,362,963**	
		Prevention Services – Public Health	Environmental Health (PH)	$6,385,703	$7,545,774	
			Other Community Services (PH)	$14,477,935	$14,467,399	

Agreement		Service Stream	Category	2019/20 $	2020/21 $	2021/22 $
		Prevention Services – Public Health Total		$20,863,637	$22,013,172	
	Allocations excluding COVID-19 Other Funding Total			$280,673,441	$282,844,463	
COVID-19 related allocations	Other Funding	Block Funded Services	Block Funded Services	$130,000	$300,000	
		Block Funded Services Total		$130,000	$300,000	
		Population Based Community Services	Alcohol, Tobacco & Other Drugs	$0	$0	
			Community Care Programs	$0	$0	
			Community Mental Health	$0	$0	
			Community Mental Health – Child & Youth	$0	$0	
			Other Community Services	$0	$0	
			Other Funding Subsidy/(Contribution)	$0	$0	
			Primary Health Care	$0	$0	
		Population Based Community Services Total		$0	$0	
		Other Specific Funding	Aged Care Assessment Program	$0	$0	
			Commercial Activities	$0	$0	
			Consumer Information Services	$0	$0	
			Depreciation	$0	$0	
			Disability Residential Care Services	$0	$0	
			Environmental Health	$0	$0	
			Home & Community Care (HACC) Program	$0	$0	
			Home & Community Medical Aids & Appliances	$0	$0	
			Home Care Packages	$0	$0	
			Interstate Patients	$0	$0	
			Multi-Purpose Health Services	$0	$0	
			Prisoner Health Services	$0	$0	
			Oral Health	$0	$0	
			Patient Transport	$0	$0	
			Research	$0	$0	
			Residential Aged Care	$0	$0	
			Specific Allocations	$5,000,051	$3,881,831	
			State-Wide Functions	$0	$0	
			Transition Care	$0	$0	
		Other Specific Funding Total		$5,000,051	$3,881,831	
			Environmental Health (PH)	$0	$0	

Agreement		Service Stream	Category	2019/20 $	2020/21 $	2021/22 $
		Prevention Services – Public Health	Other Community Services (PH)	$0	$0	
		**Prevention Services – Public Health Total**		**$0**	**$0**	
	**COVID-19 Allocations Other Funding Total**			**$5,130,051**	**$4,181,831**	
**Grand Total**				**$285,803,492**	**$287,026,294**	

Table 7 Specified Grants

Program	2019/20 $	2020/21 $	2021/22 $
High Cost Outliers	$2,779,168	$2,796,607	
Limited Indication Medication Scheme	$530,452	$533,781	
PET Service	-$0	-$0	
18-19 Purch Initiatives (Final reconciliation) - Rewards	$186,024	$0	
20-21 QIP - Antenatal care for Indigenous women	$0	$699,584	
**Grand Total**	**$3,495,644**	**$4,029,972**	

Table 8 Hospital and Health Service Service Agreement and State Level Block Payments to state managed funds from Commonwealth payments into national funding pool

State:	QLD	Service agreement for financial year:	2020/21
HHS	Cairns and Hinterland	Version for financial year:	
HHS ID		Version effective for payments from:	
		Version status:	07.07.2020

HHS ABF payment requirements:

Expected National Weighted Activity Unit (NWAU)		National efficient price (NEP) (as set by IHPA)
ABF Service group	Projected NWAU – N2021 (Draft)	
Admitted acute public services	78,286	$5,320
Admitted acute private services	4,249	$5,320
Emergency department services	18,526	$5,320
Non-admitted services	13,334	$5,320
Mental health services	5,796	$5,320
Sub-acute services	5,801	$5,320
**LHN ABF Total – excluding COVID-19**	**125,992**	**$5,320**
**LHN ABF Total – COVID-19 NPA**	**0**	**$5,320**

Note:

NWAU estimates do not take account of cross-border activity

Reporting requirements by HHS - total block funding paid (including Commonwealth) per HHS, as set out in Service Agreement:

Amount (Commonwealth and State) for each amount of block funding from state managed fund to LHN:	
**Block funding component**	**Estimated Commonwealth and state block funding contribution (ex GST)**
Block funded hospitals	$61,486,132
Community mental health services	$62,785,663
Teaching, Training and Research	$24,011,148
Home ventilation	$0
Other block funded services	$0
**Total block funding for LHN – excluding COVID-19**	**$148,282,943**
**Total funding for LHN under COVID-19 NPA State Public Health Payment**	**$4,181,831**

6. Purchased services

6.1 State-funded Outreach Services

(a) The HHS forms part of a referral network with other HHSs. Where state-funded Outreach Services are currently provided the HHS will deliver these Health Services in line with the following principles:

(i) historical agreements for the provision of Outreach Services will continue as agreed between HHSs;

(ii) funding will remain part of the providing HHS's funding base;

(iii) activity should be recorded at the HHS where the Health Service is being provided; and

(iv) the Department will purchase outreach activity based on the utilisation of the Activity Based Funding (ABF) price when Outreach Services are delivered in an ABF facility.

(b) Where new or expanded state-funded Outreach Services are developed the following principles will apply:

(i) the Department will purchase outreach activity based on the utilisation of the ABF price when Outreach Services are delivered in an ABF facility;

(ii) agreements between HHSs to purchase Outreach Services will be based on a cost recovery model, which will ensure providing sites are not financially disadvantaged and annual increases will be consistent with the ABF model;

(iii) any proposed expansion or commencement of Outreach Services will be negotiated between HHSs;

(iv) the HHS is able to purchase the Outreach Service from the most appropriate provider including private providers or other HHSs. However, when a change to existing Health Services is proposed, a transition period of at least 12 months will apply during which time the HHS will be required to continue to purchase Outreach Services from the HHS currently providing the Health Service;

(v) any changes to existing levels of Outreach Services need to be agreed to by both HHSs and any proposed realignment of funding should be communicated to the Department to ensure that any necessary funding changes are actioned as part of the Service Agreement amendment process and/or the annual negotiation of the Service Agreement Value; and

(vi) the activity should be recorded at the HHS where the Health Service is being provided.

(c) In the event of a disagreement regarding the continued provision of state-funded Outreach Services:

(i) any proposed cessation of Outreach Services will be negotiated between HHSs to mitigate any potential disadvantage or risks to either HHS; and

(ii) redistribution of funding will be agreed between the HHSs and communicated to the Department to action through the Service Agreement amendment processes outlined in Schedule 5 of this Service Agreement.

6.2 **Telehealth services**

(a) The HHS will support implementation of the Department Telehealth program, including the Telehealth Emergency Support Service. The HHS will collaborate with the Department, other HHSs, relevant non-government organisations and Primary Care stakeholders to contribute to an expanded network of Telehealth services to better enable a program of scheduled and unscheduled care.

(b) The HHS will ensure dedicated Telehealth Coordinators progress the Telehealth agenda locally, driving stakeholder engagement, adoption, planning and implementation activities that will support and grow Telehealth enabled services through substitution of existing face to face services and identification of new Telehealth enabled models of care.

(c) The HHS will ensure the Medical Telehealth Lead will collaborate with the network of HHS based Telehealth Coordinators and the Telehealth Support Unit to assist in driving promotion and adoption of Telehealth across the State through intra and cross-HHS clinician led engagement and change management initiatives as well as informing the development and implementation of clinical protocols and new Telehealth enabled models of care.

6.3 **Newborn hearing screening**

(a) In line with the National Framework for Neonatal Hearing Screening the HHS will:

(i) provide newborn hearing screening in all birthing hospitals and screening facilities; and

(ii) provide where applicable, co-ordination, diagnostic audiology, family support, and childhood hearing clinic services which meet the existing screening, audiology and medical protocols available from the Healthy Hearing website.

6.4 **Statewide Services**

The HHS has responsibility for the provision and/or coordination of the following Statewide Services. It is recommended that the HHS establish a Formal Agreement with the recipient HHSs regarding the roles and responsibilities of Statewide Service provision and receipt as described in the Definitions. In the event of a dispute regarding the provision of these services HHSs should refer to clause 14.9 of the main terms and conditions of this Service Agreement.

(a) **Rheumatic Heart Disease Register and Control Program**

The HHS will continue to host the Commonwealth funded statewide Rheumatic Heart Disease Register and Control Program, Queensland.

6.5 **Statewide and highly specialised clinical services**

The HHS will:

(a) participate in and contribute to the staged review of the purchasing model for identified Statewide and highly specialised clinical services; and

(b) collaborate with the Department and other HHSs in the development of Statewide Services Descriptions through the implementation of the Statewide Services Governance and Risk Management Framework. The Statewide Services Governance and Risk Management Framework guides the Department and HHSs in the strategic management, oversight and delivery of Statewide Services in order to optimise clinical safety and quality and ensure sustainability of services across Queensland.

6.6 **Regional Services**

The HHS has responsibility for the provision and/or coordination of the Regional Services listed below. It is recommended that the HHS establish a Formal Agreement with the recipient HHSs regarding the roles and responsibilities of Regional Service provision and receipt as described in the Definitions. In the event of a dispute regarding the provision of these services HHSs should refer to clause 14.9 of the main terms and conditions of this Service Agreement.

(a) **Basic physician training pathway**

(i) The HHS will undertake the recruitment, selection, allocation and education of Queensland Basic Physician Pathway Trainees for the Far-North rotation in conjunction with Townsville HHS.

(ii) These activities will be undertaken in line with the state-wide Queensland Basic Physician Training Pathway Model, supported by a Pathway Rotation Coordinator (Senior Medical Officer) and Pathway Project Officer, hosted in the HHS.

(b) **Community Forensic Outreach Service**

Services to Cairns and Hinterland and Torres and Cape HHS

(c) **Court liaison service**

Services to Cairns and Hinterland and Torres and Cape HHS

(d) **Mental health clinical cluster support program**

Services to Cairns and Hinterland, Mackay, North West, Torres and Cape and Townsville HHSs

(e) **Mental health clinical indicator program**

Services to Cairns and Hinterland, Mackay, North West, Torres and Cape and Townsville HHSs.

(f) **North Queensland Forensic Adolescent Mental Health Services**

Services to Cairns and Hinterland and Torres and Cape HHSs

6.7 **Rural and remote clinical support**

This clause does not apply to this HHS.

6.8 **Prevention Services, Primary Care and Community Health Services**

(a) The following funding arrangements will apply to the Prevention, Primary Care and Community Health Services delivered by the HHS:

(i) Department funding for Community Health Services. A pool of funding for these services is allocated to each HHS for a range of Community Health Services and must be used to meet local Primary Care and community healthcare and prevention needs including through delivery of the services identified in Table 6 and HHSs have the discretion to allocate funding across Primary Care and Community Health Services and Prevention Services according to local priorities.

(ii) Department specified funding models for consumer information services, disability, residential care, environmental health, prisoner health services, home and community medical aids, Primary Care, community mental health services, and alcohol and other drugs services. The funding specified for these programs is listed in Table 6 and Department Community Health Service grants.

(iii) Funding from other state government departments and the Commonwealth for specific programs (third party funded services).

(b) **Prevention Services**

The HHS will provide Prevention Services in line with public health related legislation and the service delivery and reporting requirements outlined in the *Public Health Practice Manual*, including:

(i) **Specialist Public Health Units**

The HHS will:

(A) provide a specialist communicable disease epidemiology and surveillance, disease prevention and control service;

(B) maintain and improve, using a public health approach, the surveillance, prevention and control of notifiable conditions, including the prevention and control of invasive and exotic mosquitos, in accordance with national and/or State guidelines and ensure clinical and provisional notification of specified notifiable conditions are reported in accordance with the *Public Health Act* and Public Health Regulations;

(C) provide a specialist environmental health service, which includes assessment and coordination of local responses to local environmental health risks;

(D) undertake regulatory monitoring, investigation, enforcement and compliance activity on behalf of the Department;

(E) utilise specialist public health units to support the HHS through the provision of advice on prevention strategies and evidence; and

(F) provide specialist communicable disease epidemiology and surveillance, disease prevention and control and environmental

health services to Torres and Cape HHS. This will include prevention, management and response activities for dengue; assessment and coordination of local responses to environmental health risks; and regulatory monitoring, enforcement and compliance activities on behalf of the Department. Where this includes visits to Cape York by Cairns-based Public Health Unit staff, office accommodation and related support will be made available for those staff during their visit.

(ii) **Preventive health services**

The HHS will:

(A) maintain delivery of risk factor prevention and early intervention programs and services targeting nutrition, physical activity, alcohol consumption, tobacco use, overweight and obesity and falls prevention

(B) maintain delivery of the school-based youth nursing program throughout Queensland secondary schools

(C) promote brief interventions, lifestyle modification programs and other prevention, promotion or early intervention activities.

(iii) **Immunisation services**

The HHS will maintain or improve existing immunisation coverage through continuation of current immunisation services including:

(A) national immunisation program;

(B) opportunistic immunisation in healthcare facilities;

(C) special immunisation programs; and

(D) delivery of the annual school immunisation program in accordance with the Guideline for Immunisation Services (QH-GDL-955:2014).

(iv) **Sexually transmissible infections including HIV and viral hepatitis**

The HHS will maintain and improve, using a public health approach, the prevention, testing, treatment and contact tracing of blood borne viruses and sexually transmissible infections with a continued focus on relevant identified target populations such as First Nations people and culturally and linguistically diverse populations through Services including, but not limited to:

(A) public health units;

(B) sexual health services;

(C) infectious diseases services;

(D) viral hepatitis services;

(E) syphilis surveillance services;

(F) needle and syringe programs; and

(G) existing clinical outreach and support programs in place between HHSs.

(v) **Tuberculosis services**

(A) The HHS will ensure there is no financial barrier for any person to tuberculosis diagnostic and management services and will ensure that services are available in accordance with the Tuberculosis Control health service directive (qh-hsd-040:2018) and Protocol (qh-hsdptl-040-1:2018).

(B) The Cairns Tuberculosis Control Unit will provide the following services to Cairns and Hinterland and Torres and Cape HHSs:

- patient assessment and management of mycobacterial diseases;
- outpatient services onsite and clinics offsite;
- clinics as required at private health facilities, educational facilities and public areas and workplaces;
- screening services; and
- BCG vaccination services.

(vi) **Public Health Events of State Significance**

The HHS will comply with the *Declaration and Management of a Public Health Event of State Significance* health service directive (qh-hsd-046:2014).

(vii) **Cancer screening services**

The HHS will:

(A) increase cervical screening rates for women in rural and remote areas and refer clients to relevant preventive health programs such as BreastScreen Queensland and My Health for Life by maintaining the existing Mobile Women's Health Service;

(B) provide the Department with an annual report detailing the services provided by the Mobile Women’s Health Service:

- across Cairns and Hinterland HHS; and
- for Townsville HHS – Cardwell only;

(C) ensure that all cervical screening services provided by the HHS are delivered in accordance with the National Competencies for Cervical Screening Providers and national cervical screening policy documents; and

(D) provide timely, appropriate, high quality and safe follow-up diagnostic services within the HHS for National Cervical Screening Program participants in accordance with the *National Cervical Screening Program Guidelines for the Management of Screen-detected Abnormalities, Screening in Specific*

Populations and Investigation of Abnormal Vaginal Bleeding (2017) and national cervical screening policy documents.

(E) develop, implement and evaluate a plan to increase participation in bowel cancer screening and provide the Department with an evaluation report at the end of 2021/22 for the following catchment:

- Cairns and Hinterland HHS; and
- Torres and Cape HHS;

(F) provide timely, appropriate, high quality and safe diagnostic assessment services for National Bowel Cancer Screening Program participants in accordance with the National Health and Medical Research Council's *Clinical Guidelines for Prevention, Early Detection and Management of Colorectal Cancer* (2017). Services to be provided:

- across Cairns and Hinterland HHS.

(G) develop and implement a local service management plan to increase participation in and guide the delivery of accessible breast screening for women in the target age group (50-74 years) through a BreastScreen Australia accredited service. The screening and assessment services should be delivered in accordance with the BreastScreen Queensland (BSQ) Quality Standards Protocols and Procedures Manual, BreastScreen Australia National Accreditation Standards and national policies. Services to be provided:

- across Cairns and Hinterland HHS;
- across Torres and Cape HHS;
- within North West HHS for the Carpentaria SA2 only; and
- within Townsville HHS for Tully SA2 only;

(H) allow the use of the HHS BSQ mobile asset by other HHSs during periods where practical to maximise utilisation of BSQ mobile fleet;

(I) negotiate reciprocal utilisation of BSQ mobile assets with the Townsville HHS;

(J) schedule screening services through provision of BSQ mobile vans to increase accessibility for women living in rural and remote areas. While screening schedules are ideally finalised by HHSs six months in advance, confirmation of mobile sites is required by the BSQ Registry eight weeks prior to commencement at each site to ensure invitations for screening are prepared and distributed to women in the catchment area; and

(K) develop and implement infrastructure plans to manage BSQ

asset lifecycle performance and replacement schedules including mobile vans. The repair and maintenance services for the BSQ mobile service fleet will be managed and administered by the Mobile Dental Clinic Workshop in Metro South HHS. The HHS will notify the Mobile Dental Clinic Workshop of any repair and maintenance issues and liaise with the Mobile Dental Clinic Workshop to arrange scheduled servicing. The Mobile Dental Clinic Workshop will meet the costs for these services subject to availability of allocated funding for this purpose in any given financial year.

6.9 **Oral health services**

The HHS will ensure that:

(a) oral health services are provided to the Eligible Population at no cost to the patient[9] and that the current range of clinical services will continue;

(b) oral health services fulfil the relevant obligations related to Commonwealth Government dental funding program/s;

(c) service delivery is consistent with the oral health policy framework, and

(d) the repair, maintenance and relocation service for the mobile dental fleet continues to be provided by the Mobile Dental Clinic Workshop in Metro South HHS.

6.10 **Prisoner health services**

The HHS will:

(a) provide health Services for prisoners consistent with the principles, responsibilities and requirements specified in the Memorandum of Understanding (Prisoner Health Services) between Queensland Health and Queensland Corrective Services;

(b) provide the Department with an annual report regarding the provision of these Services; and

(c) establish local collaborative arrangements with Queensland Corrective Services to improve the health and well-being of prisoners and to contribute to the safe operation of the correctional centre.

6.11 **Refugee health**

(a) The HHS will implement a health service for refugees, special humanitarian entrants and asylum seekers which provides:

(i) standard initial health assessments, including catch-up vaccination;

(ii) coordination of short-term health management; and

(iii) supported referral to existing services for continuing care, in particular to general practitioners to conduct the medical component of the refugee

[9] The HHS may provide oral health services on a fee-for-service basis to non-eligible patients in rural and remote areas where private dental services are not available.

health assessment.

(b) This service will be operated out of Cairns Clinic.

6.12 **Adult sexual health clinical forensic examinations**

(a) The HHS will:

(i) provide 24-hour access to clinical forensic examinations for adult victims of sexual assault who present at a public hospital; and

(ii) provide the Department with a quarterly report on the number of examinations provided.

(b) The Service provided will be consistent with the principles of the Queensland Government inter-agency guidelines for responding to people who have experienced sexual assault and any standards issued pursuant to a Health Service Directive.

7. Teaching training and research

The HHS will provide the teaching, training and research programs for which funding is provided within Schedule 2 of this Service Agreement and as described below:

7.1 **Clinical education and training**

(a) The HHS will:

(i) continue to support and align with the current Student Placement Deed Framework which governs clinical placements from relevant tertiary education providers in Queensland HHS facilities;

(ii) comply with the obligations and responsibilities of Queensland Health under the Student Placement Deed, as appropriate, as operator of the facility at which the student placement is taking place;

(iii) comply with the terms and conditions of students from Australian education providers participating in the Student Placement Deed Framework;

(iv) only accept clinical placements of students from Australian education provides participating in the Student Placement Deed Framework;

(v) continue to provide training placements consistent with and proportionate to the capacity of the HHS. This includes, but is not limited to, planning and resourcing for clinical placement offers in collaboration with other HHSs and the Department, and the provision of placements for the following professional groups relevant to the HHS:

(A) medical students

(B) nursing and midwifery students

(C) pre-entry clinical allied health students

(D) interns

(E) rural generalist trainees

(F) vocational medical trainees

(G) first year nurses and midwives

(H) re-entry to professional register nursing and midwifery candidates

(I) dental students

(J) allied health rural generalist training positions

(K) Aboriginal and Torres Strait Islander Health Workers and Aboriginal and Torres Strait Islander Health Practitioners

(vi) participate in vocational medical rotational training schemes, facilitate the movement of vocational trainees between HHSs and work collaboratively across HHSs to support education and training program outcomes;

(vii) report, at the intervals and in the format agreed between the Parties, to the Department on the pre-entry clinical placements provided under the Student Placement Deed Framework;

(viii) comply with the state-wide vocational medical training pathway models including:

(A) The Queensland Basic Physician Training Network;

(B) The Queensland General Medicine Advanced Training Network;

(C) The Queensland Intensive Care Training Pathway;

(D) The Queensland Basic Paediatric Training Network;

(E) The Queensland General Paediatric Advanced Training Network; and

(F) The Queensland Neonatal and Perinatal Medicine Advanced Training Network;

(ix) support the provision of placements by the Queensland Physiotherapy Placement Collaborative for physiotherapy pre-entry students via the Physiotherapy Pre-registration Clinical Placement Agreement;

(x) provide clinical area placements for dietetics pre-entry students from additional funding provided through relevant agreements with Universities; and

(xi) oversight profession specific (psychology) and inter-professional statewide allied health clinical education programs.

(b) In addition, the Health Practitioners and Dental Officers (Queensland Health) Certified Agreement (No 2) 2016 (the HP agreement) requires Hospital and Health Services to:

(i) continue to support development of allied health research capacity through continued implementation and retention of health practitioner research positions provided through the HP agreement; and

(ii) support development of allied health clinical education capacity through continued implementation and retention of clinical educator positions provided through the HP agreement, continuing to provide allied health pre-entry clinical placements and maintaining support for allied health HP 3 to 4 rural development pathway positions.

7.2 **Health and medical research**

The HHS will:

(a) Articulate an investment strategy for research (including research targets and Performance Measures) which integrates with the clinical environment to improve clinical outcomes;

(b) Develop mechanisms for completing administrative governance for the approval of research in line with a national benchmark of 25 days (Standard Operating Procedures for Queensland Health Research Governance Officers 2013);

(c) Develop mechanisms for monitoring site research activity in line with jurisdictional commitments and National Health and Medical Research Council Guidelines (*Framework for* Monitoring *Guidance for the national approach to single ethical review of multi-centre research, January 2012*); and

(d) Develop systems to capture research and development expenditure and revenue data and associated information on research.

Schedule 3 Performance Measures

1. Purpose

This Schedule 3 outlines the Performance Measures that apply to the HHS.

2. Performance Measures

2.1 The Performance and Accountability Framework uses Performance Measures to monitor the extent to which the HHS is delivering the high-level objectives set out in this Service Agreement.

2.2 Each Performance Measure is identified under one of four categories:

(a) Safety and Quality Markers which together provide timely and transparent information on the safety and quality of services provided by the HHS;

(b) Key Performance Indicators (KPIs) which are focused on the delivery of key strategic objectives and statewide targets. KPI performance will inform HHS performance assessments;

(c) Outcome Indicators which provide information on specific activities and interventions where there is a reasonable expectation or evidence that they make a positive contribution towards improving the health status and experience of patients; and

(d) supporting indicators which provide contextual information and enable an improved understanding of performance, facilitate benchmarking of performance across HHSs and provide intelligence on potential future areas of focus. Supporting indicators are available on-line as referenced in Appendix 1 to this Service Agreement.

2.3 The HHS should refer to the relevant attribute sheet for each Performance Measure for full details. These are available on-line as referenced in Appendix 1 to this Service Agreement.

2.4 The Performance Measures identified in Table 9; Table 10 and Table 11 are applicable to the HHS unless otherwise specified within the attribute sheet.

2.5 The HHS will meet the target for each KPI identified in Table 9 as specified in the attribute sheet.

2.6 The Performance Measures identified in italic text are for future development.

2.7 Further information on the performance assessment process is provided in the supporting document to this Service Agreement, Performance and Accountability Framework 2020/21 referenced at Appendix 1 to this Service Agreement.

Table 9 HHS Performance Measures – Key Performance Indicators

Key Performance Indicators	
**Safe**	
The health and welfare of service users is paramount	
• Minimise risk	• Avoid harm from care
• Transparency and openness	• Learn from mistakes
**Title**	
Hospital Acquired Complications	
Emergency length of stay:<br>• % of Emergency Department attendances who are admitted as an inpatient, including to a short stay unit, and whose Emergency Department length of stay is within 4 hours	
Number of Emergency Department stays greater than 24 hours	
Emergency Department wait time by triage category	
Rate of face to face community follow up within 1-7 days of discharge from an acute mental health inpatient unit	
**Timely**	
Care is provided within an appropriate timeframe	
• Treatment within clinically recommended time	
**Title**	
Patient off stretcher time:<br>• % of patients transferred from Queensland Ambulance Service into the Emergency Department within 30 minutes	
Elective surgery:<br>• % of category 1 patients treated within the clinically recommended time	
Elective surgery:<br>• Number of ready for care patients waiting longer than the clinically recommended timeframe for their category	
Specialist outpatients:<br>• % of category 1 patients who receive their initial specialist outpatient appointment within the clinically recommended time	
Specialist outpatients:<br>• Number of ready for care category 1 patients waiting longer than clinically recommended for their initial specialist outpatient appointment	
Gastrointestinal endoscopy:<br>• % of category 4 patients who are treated within the clinically recommended time	
Gastrointestinal endoscopy:<br>• Number of patients waiting longer than clinically recommended timeframe for their category	
Access to oral health services:<br>• % of patients on the general care dental wait list waiting for less than the clinically recommended time	

Equitable	
Consumers have access to healthcare that is responsive to need and addresses health inequalities	
• Fair access based on need	• Addresses inequalities
**Title**	
Potentially Preventable Hospitalisations – First Nations People	
Telehealth utilisation rates:<br>• Number of non-admitted telehealth service events	
**Efficient**	
Available resources are maximised to deliver sustainable, high quality healthcare	
• Avoid waste	• Minimise financial risk
• Sustainable/productive	• Maximise available resources
**Title**	
Forecast operating position:<br>• Full year<br>• Year to date	
Average sustainable Queensland Health FTE	
Capital expenditure performance	
**Patient Centred**	
Providing Healthcare that is respectful of and responsive to individual patient preferences, needs and values	
• Patient involved in care	• Patient feedback
• Respects patient/person values and preferences	• Care close to home
**Title**	
Proportion of mental health service episodes with a documented care plan	
Proportion of beds vacated by 11am	

Table 10 HHS Performance Measures - Safety and Quality Markers

Safety and Quality Markers	
**Safe**	
The health and welfare of service users is paramount	
• Minimise risk<br>• Transparency and openness	• Avoid harm from care<br>• Learn from mistakes
**Title**	
Sentinel Events:<br>• Number of wholly preventable sentinel events	
Hospital Standardised Mortality Ratio	
Healthcare-associated Staphylococcus Aureus (including MRSA) bacteraemia:<br>• Rate per 10,000 patient days	
Severity Assessment Code (SAC) closure rates:<br>• % of incidents closed within the prescribed timeframe	
Unplanned Readmission Rates	

Table 11 HHS Performance Measures – Outcome Indicators

**Outcome Indicators**	
**Safe**	
**The health and welfare of service users is paramount**	
• Minimise risk<br>• Transparency and openness	• Avoid harm from care<br>• Learn from mistakes
**Title**	
Rate of seclusion events per 1,000 acute mental health admitted patient days	
Rate of absent without approval from acute mental health inpatient care	
**Timely**	
Care is provided within an appropriate timeframe	
• Treatment within clinically recommended time	
**Title**	
Reperfusion therapy for acute ischaemic stroke:<br>• Proportion of patients treated with either IV thrombolytic drugs or endovascular clot retrieval	
Access to emergency dental care:<br>• % of emergency courses of care for adult dental patients that commence within the recommended waiting times	
**Equitable**	
Consumers have access to healthcare that is responsive to need and addresses health inequalities	
• Fair access based on need	• Addresses inequalities
**Title**	
First Nations people representation in the workforce:<br>• % of the workforce who identify as being First Nations people	
Completed general courses of oral health care for First Nations people adult patients	
Low birthweight:<br>• % of low birthweight babies born to Queensland mothers	
**Patient Centred**	
Providing Healthcare that is respectful of and responsive to individual patient preferences, needs and values	
• Patient involved in care	• Patient feedback
• Respects patient/person values and preferences	• Care close to home
**Title**	
Complaints resolved within 35 calendar days	
Advance care planning:<br>• The proportion of approaches made to people who are identified as being at risk of dying within the next 12 months, or suitable for an advance care planning discussion, and who are offered the opportunity to consider, discuss and decide their preferences for care at the end of life	

Effective	
Healthcare that delivers the best achievable outcomes through evidence-based practice	
• Evidence based practice	• Care integration
• Treatment directed to those who benefit	• Optimise Health
• Clinical Capability	

**Title**
Uptake of the smoking cessation clinical pathway for public hospital inpatients and dental clients
Potentially Preventable Hospitalisations – diabetes complications:<br>• The number and proportion of hospitalisations of people with Diabetes complications that could have potentially been prevented through the provision of appropriate non-hospital health services.
Potentially Preventable Hospitalisations – non-diabetes complications:<br>• The number and proportion of hospitalisations of people with non-Diabetes complications that could have potentially been prevented through the provision of appropriate non-hospital health services.
% of oral health activity which is preventive
Cardiac rehabilitation:<br>• Proportion of public cardiac patients that are referred to cardiac rehabilitation and complete a timely patient journey
Adolescent vaccinations administered via the statewide School Immunisation Program

Schedule 4 Data Supply Requirements

1. Purpose

1.1 *The Hospital and Health Boards Act 2011*[10] (s.16(1)(d)) provides that the Service Agreement will state the performance data and other data to be provided by an HHS to the Chief Executive, including how, and how often, the data is to be provided.

1.2 This Schedule 4 specifies the data to be provided by the HHS to the Chief Executive and the requirements for the provision of the data.

2. Principles

2.1 The following principles guide the collection, storage, transfer and disposal of data:

(a) trustworthy – data is accurate, relevant, timely, available and secure;

(b) private – personal information is protected in accordance with the law;

(c) valued – data is a core strategic asset;

(d) managed – collection of data is actively planned, managed and compliant; and

(e) quality – data provided is complete, consistent, undergoes regular validation and is of sufficient quality to enable the purposes outlined in clause 3.2 of this Schedule 4 to be fulfilled.

2.2 The Parties agree to constructively review the data supply requirements as set out in this Schedule 4 on an ongoing basis in order to:

(a) ensure data supply requirements are able to be fulfilled; and

(b) minimise regulatory burden.

3. Roles and responsibilities

3.1 **Hospital and Health Services**

(a) The HHS will:

(i) provide, including the form and manner and at the times specified, the data specified in the data supply requirements (Attachment A to this Schedule 4) in accordance with this Schedule 4;

(ii) provide data in accordance with the provisions of the *Hospital and Health Boards Act 2011, Public Health Act 2005* and *Private Health Facilities Act 1999;*

---

[10] Section 143(2)(a) of the *Hospital and Health Boards Act 2011* provides that the disclosure of confidential information (as defined in s.139 of the Act) to the Chief Executive by an HHS under a service agreement is a disclosure permitted by an Act.

(iii) provide other HHSs with routine access to data, that is not Patient Identifiable Data, for the purposes of benchmarking and performance improvement;

(iv) provide data as required to facilitate reporting against the Performance Measures set out in Schedule 3 of this Service Agreement;

(v) provide data as specified within the provision of a health service directive;

(vi) provide activity data that complies with the national data provision timeframes required under the Independent Hospital Pricing Authority (IHPA) data plan for Commonwealth funding. Details of the timeframes are specified in the 'Commonwealth Efficient Growth Funding and National Weighted Activity Units (NWAUs)' specification sheet included in the supporting document Purchasing Policy and Funding Guidelines 2020/21 and the clinical placement data supply requirements; and

(vii) as requested by the Chief Executive from time to time, provide to the Chief Executive data, whether or not specified in this Schedule 4 or the Service Agreement, as specified by the Chief Executive in writing to the HHS in the form and manner and at the times specified by the Chief Executive.

(b) Data that is capable of identifying patients will only be disclosed as permitted by, and in accordance with, the *Hospital and Health Boards Act 2011, Public Health Act 2005 and the Private Health Facilities Act 1999.*

3.2 **Department**

The Department will:

(a) produce a monthly performance report which includes:

(i) actual activity compared with purchased activity levels;

(ii) any variance(s) from purchased activity;

(iii) performance information as required by the Department to demonstrate HHS performance against the Performance Measures specified in Schedule 3 of this Service Agreement; and

(iv) performance information as required by the Department to demonstrate the achievement of commitments linked to specifically allocated funding included in Schedule 2 of this Service Agreement.

(b) utilise the data sets provided for a range of purposes including:

(i) to fulfil legislative requirements;

(ii) to deliver accountabilities to state and commonwealth governments;

(iii) to monitor and promote improvements in the safety and quality of Health Services;

(iv) to support clinical innovation; and

(c) advise the HHS of any updates to data supply requirements as they occur.

Attachment A Data Supply Requirements

The HHS should refer to the relevant minimum data set for full details. These are available on-line as referenced in Appendix 1.

Table 12 Clinical data

Data Set	Data Custodian
Aged Care Assessment Team data via the Aged Care Evaluation (ACE) database	Strategic Policy Unit
Alcohol Tobacco and Other Drug Treatment Services	Mental Health Alcohol and Other Drugs Branch
Alcohol and Other Drugs Establishment Collection	Mental Health Alcohol and Other Drugs Branch
Allied Health Clinical Placement Activity Data	Allied Health Professions Office of Queensland
Australian and New Zealand Intensive Care Society (ANZICS) Data Collection	Healthcare Improvement Unit
BreastScreening Clinical Data	Executive Director, Preventive Health Branch
Clinical Incident Data Set	Patient Safety and Quality Improvement Service
Clinical Placement Data (excluding Allied Health)	Workforce Strategy Branch
Consumer Feedback Data Set	Patient Safety and Quality Improvement Service
Elective Surgery Data Collection	Healthcare Improvement Unit
Emergency Data Collection	Healthcare Improvement Unit
Gastrointestinal Endoscopy Data Collection	Healthcare Improvement Unit
Hand Hygiene Compliance Data	Communicable Diseases Branch
Healthcare Infection Surveillance Data	Communicable Diseases Branch
Maternal Deaths	Queensland Maternal and Perinatal Quality Council (through Statistical Services Branch)
Mental Health Act Data	Mental Health Alcohol and Other Drugs Branch
Mental Health Activity Data Collection	Mental Health Alcohol and Other Drugs Branch
Mental Health Carer Experience Survey Collection	Mental Health Alcohol and Other Drugs Branch
Mental Health Establishments Collection	Mental Health Alcohol and Other Drugs Branch
Monthly Activity Collection (including admitted and non-admitted patient activity and bed availability data)	Statistical Services Branch
Newborn Hearing Screening	Children's Health Queensland
Notifications Data	Chief Health Officer
Patient Experience Survey Data	Patient Safety and Quality Improvement Service
Patient Level Costing and Funding Data	HHS Funding and Costing Unit
Perinatal Data Collection	Statistical Services Branch
Queensland Bedside Audit	Patient Safety and Quality Improvement Service
Queensland Health Non-Admitted Patient Data Collection	Statistical Services Branch
Queensland Hospital Admitted Patient Data Collection	Statistical Services Branch
Queensland Needle and Syringe Program (QNSP) data	Chief Health Officer
Queensland Opioid Treatment Program Admissions and Discharges	Chief Health Officer
Radiation Therapy Data Collection	Healthcare Improvement Unit

Data Set	Data Custodian
Residential Mental Health Care Collections	Mental Health Alcohol and Other Drugs Branch
Schedule 8 Dispensing data	Chief Health Officer
School Immunisation Program – Annual Outcome Report	Communicable Diseases Branch
Specialist Outpatient Data Collection	Healthcare Improvement Unit
National Notifiable Diseases Surveillance System	Chief Health Officer
Vaccination Administration data	Chief Health Officer
Variable Life Adjusted Display (VLAD) CM (collection of hospital investigations)	Patient Safety and Quality Improvement Service
Your Experience of Service (YES) Survey Collection (Mental Health)	Mental Health Alcohol and Other Drugs Branch

Table 13 Non-clinical data

Non-Clinical Data Set	Data Custodian
Asbestos management data	Capital and Asset Services Branch
Asset Management<br>• Planning<br>• Maintenance<br>• Maintenance Budget<br>• Statement of Building Portfolio Compliance<br>• Benchmarking & Performance Data	Capital and Asset Services Branch
Conduct and Performance Excellence (CaPE)	Human Resources Branch
Expenditure	Finance Branch
Financial and Residential Activity Collection (FRAC)	Statistical Services Branch
Graduate Nursing Recruitment Data Statewide using the Public Service Commission Graduate Portal System	Office of the Chief Nursing and Midwifery Officer
Hospital Car Parks (including Government Portfolio Model funding arrangements)	Capital and Asset Services Branch
Minimum Obligatory Human Resource Information (MOHRI)	Finance Branch
Minor Capital Funding Program expenditure & forecast data	Finance Branch
Recruitment Data	Human Resources Branch
Revenue	Finance Branch
Queensland Health Workforce & Work Health & Safety Data	Human Resources Branch
Queensland Integrated Safety Information Project (QISIP)Solution Minimum Data Set	Human Resources Branch
Statewide employment matters	Human Resources Branch
Sustaining Capital Reporting Requirements (other than minor capital)	Capital and Asset Services Branch
Whole of Government Asset Management Policies data	Capital and Asset Services Branch

Schedule 5 Amendments to this Service Agreement

1. Purpose

This Schedule 5 sets out the mechanisms through which this Service Agreement may be amended during its term, consistent with the requirements of the *Hospital and Health Boards Act 2011.*

2. Principles

2.1 It is acknowledged that the primary mechanism through which HHS funding adjustments are made is through the budget build process that is undertaken annually in advance of the commencement of the financial year. This approach is intended to provide clarity, certainty and transparency in relation to funding allocations.

2.2 Amendments to the clauses of this Service Agreement should be progressed for consideration as part of the annual budget build process.

2.3 It is recognised that there is a requirement to vary funding and activity in-year. The following principles will guide amendments and amendment processes:

(a) funding allocations to HHSs should occur as early as possible within a financial year if unable to be finalised in advance of a given financial year;

(b) the number of Amendment Windows each year should be minimised to reduce the administrative burden on HHSs and the Department;

(c) Amendment Proposals should be minimised wherever possible and should always be of a material nature;

(d) Amendment Windows 2 and 3 are not intended to include funding or activity variations that could have been anticipated in advance of the financial year;

(e) Amendment Windows are intended to provide a formal mechanism to transact funding or activity variations in response to emerging priorities;

(f) Extraordinary Amendment Windows are not intended to be routinely used.

2.4 The Department remains committed to the ongoing simplification and streamlining of amendment processes.

3. Process to amend this Service Agreement

3.1 The Parties recognise the following mechanisms through which an amendment to this Service Agreement can be made:

(a) Amendment Windows;

(b) Extraordinary Amendment Windows;

(c) periodic adjustments; and

(d) end of year financial adjustments.

3.2 Amendment Windows

(a) In order for the Department to manage amendments across all HHS Service Agreements and their effect on the delivery of Public Sector Health Services in Queensland, proposals to amend this Service Agreement will be negotiated and finalised during set periods of time during the year (Amendment Windows).

(b) Amendment Windows are the primary mechanism through which amendments to this Service Agreement are made.

(c) Amendment Windows occur three times within a given financial year:

(i) Amendment Window 1: Annual Budget Build;

(ii) Amendment Window 2: In-year variation; and

(iii) Amendment Window 3: In-year variation.

(d) A Party that wants to amend the terms of this Service Agreement must give an Amendment Proposal to the other party.

(e) While a Party may submit an Amendment Proposal at any time, an Amendment Proposal will only be formally negotiated and resolved during one of the Amendment Windows outlined in Table 14 (excluding Extraordinary Amendment Windows).

Table 14 Amendment Window Exchange Dates

Amendment Window	Exchange Date	Primary Focus
Amendment Window 2: In-year variation	4 October 2019	2019/20 in-year variations
Amendment Window 3: In-year variation	14 February 2020	2019/20 in-year variations
Amendment Window 1: Annual Budget Build	27 March 2020	2020/21 budget build
Amendment Window 2: In-year variation	9 October 2020	2020/21 in-year variations
Amendment Window 3: In-year variation	12 February 2021	2020/21 in-year variations
Amendment Window 1: Annual Budget Build	26 March 2021	2021/22 budget build
Amendment Window 2: In-year variation	8 October 2021	2021/22 in-year variations
Amendment Window 3: In-year variation	11 February 2022	2021/22 in-year variations

(f) An Amendment Proposal is made by:

(i) the responsible Deputy Director-General signing and providing an Amendment Proposal to the HHS-SA Contact Person through Healthcare Purchasing and System Performance Division prior to the commencement of any Amendment Window; or

(ii) the Health Service Chief Executive signing and providing an Amendment Proposal to the D-SA Contact Person prior to the commencement of any Amendment Window.

(g) A Party giving an Amendment Proposal must provide the other Party with the following information:

(i) the rationale for the proposed amendment;

(ii) the precise drafting for the proposed amendment;

(iii) any information and documents relevant to the proposed amendment; and

(iv) details and explanation of any financial, activity or service delivery impact of the amendment.

(h) Negotiation and resolution of Amendment Proposals will occur during the Negotiation Period through a tiered process, as outlined in Figure 3.

Figure 3 Amendment Proposal negotiation and resolution

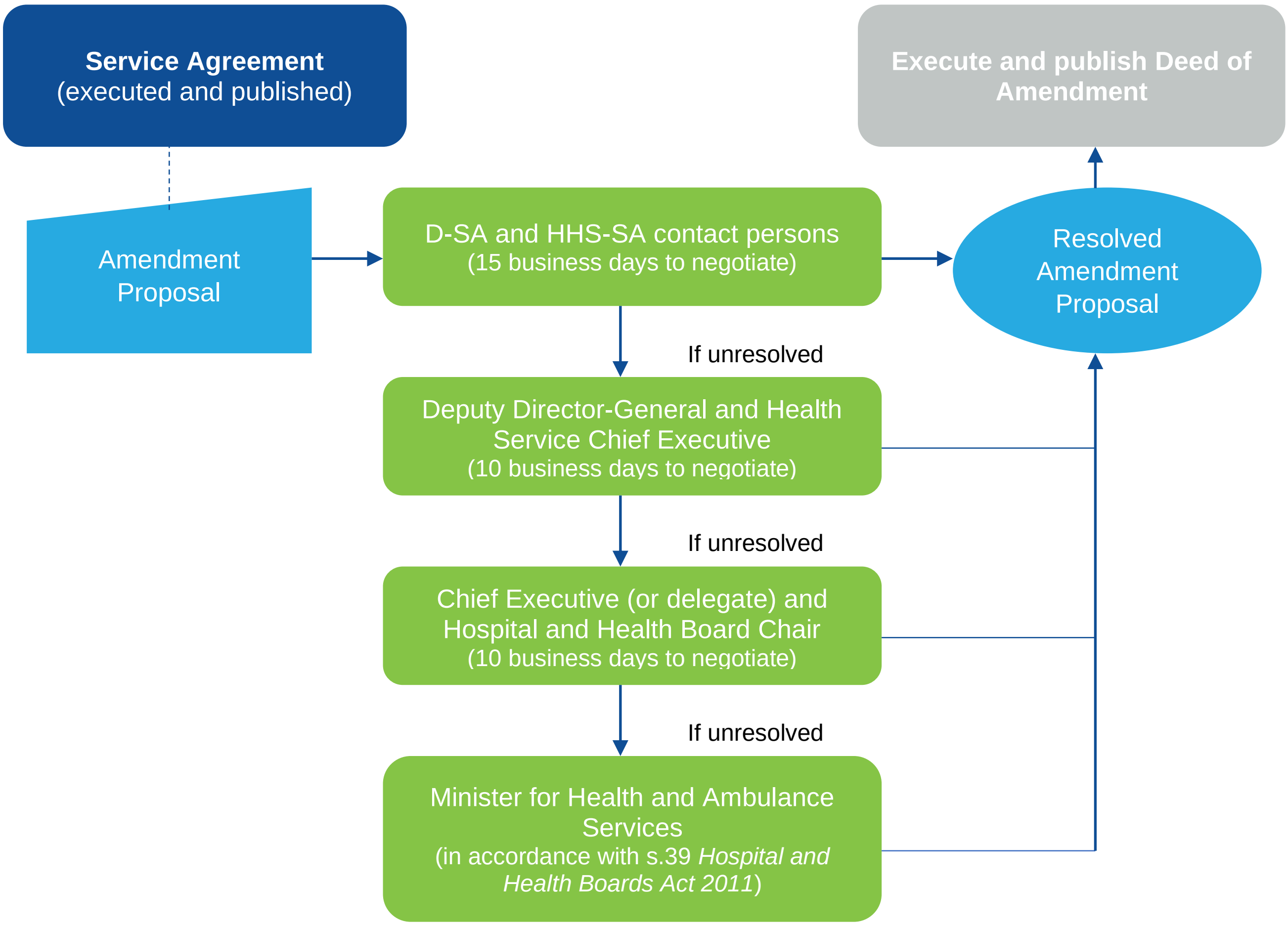

(i) The timeframes identified in Figure 3 may be reduced in the complete discretion of the Chief Executive.

(j) In accordance with section 39(5) of the *Hospital and Health Boards Act 2011*, the Parties must include any terms decided by the Minister in the Service Agreement.

(k) If the Chief Executive at any time:

(i) considers that an amendment agreed with the HHS may or will have associated impacts on other HHSs; or

(ii) considers it appropriate for any other reasons,

then the Chief Executive may:

(iii) propose further amendments to any HHS affected; and

(iv) may address the amendment and/or associated impacts of the

amendment in other ways, including through the exercise of any statutory powers and/or statutory directions under the *Hospital and Health Boards Act 2011*.

(l) Amendment Proposals that are resolved will be documented in a Deed of Amendment to this Service Agreement and executed by the Parties.

(m) Only upon execution of a Deed of Amendment by the Parties will the amendments documented by that Deed of Amendment be deemed to be an amendment to this Service Agreement.

3.3 **Extraordinary Amendment Windows**

(a) A Party that wants to amend the terms of this Service Agreement outside of an Amendment Window outlined in Table 14 must give an Extraordinary Amendment Proposal to the other Party.

(b) An Extraordinary Amendment Proposal may only be formally negotiated and resolved outside of an Amendment Window outlined in Table 14 to facilitate funding allocations where an urgent priority needs to be addressed in a timely manner and an Amendment Window is not available within an acceptable timeframe.

(c) An Extraordinary Amendment Proposal that is issued by or on behalf of the Chief Executive must be given to the HHS-SA Contact Person through Healthcare Purchasing and System Performance Division.

(d) An Extraordinary Amendment Proposal that is issued by or on behalf of the HHS must be given to the D-SA Contact Person through Healthcare Purchasing and System Performance Division.

(e) An Extraordinary Amendment Proposal may be issued by or on behalf of either Party at any time, noting the requirement that it relate to an urgent priority that necessitates timely resolution.

(f) Negotiation and resolution of Extraordinary Amendment Proposals will be through a tiered process as outlined in Figure 3.

(g) The timeframes identified in Figure 3 may be reduced in the complete discretion of the Chief Executive.

(h) In accordance with section 39(5) of the *Hospital and Health Boards Act 2011*, the Parties must include any terms decided by the Minster in the Service Agreement.

(i) Extraordinary Amendment Proposals that are resolved must be executed by both Parties.

(j) The Parties must comply with the terms of the Extraordinary Amendment Proposal from the date that the final Party executed the Extraordinary Amendment Proposal.

(k) The terms of an executed Extraordinary Amendment Proposal will be documented in a Deed of Amendment to this Service Agreement and executed by the Parties. Once executed, the Deed of Amendment will expressly exclude the application of the Extraordinary Amendment Proposal and only the terms of the Deed of Amendment will apply.

3.4 Periodic adjustments

(a) The Service Agreement Value may be adjusted outside of an Amendment Window to allow for funding variations that:

(i) occur on a periodic basis;

(ii) are referenced in the Service Agreement; and

(iii) are based on a clearly articulated formula.

(b) Adjustments to the Service Agreement Value and purchased activity that are required as a result of a periodic adjustment will be made following agreement between the Parties of the data on which the adjustment is based.

(c) The Chief Executive (or delegate) will issue an Adjustment Notice to the HHS-SA Contact Person confirming any funding adjustment that has been made.

(d) Following receipt of an Adjustment Notice, the Parties will comply with the Adjustment Notice and immediately take the steps necessary to give effect to the requirements of the Adjustment Notice.

(e) A Deed of Amendment will not be issued immediately following periodic adjustment. The HHS will be provided with a summary of all transactions made through periodic adjustment on completion.

(f) Any funding adjustments agreed through periodic adjustment which result in a variation to the Service Agreement Value, purchased activity or the requirements specified within Schedule 2 of this Service Agreement will be formalised in a Deed of Amendment issued following the next available Amendment Window.

3.5 End of financial year adjustments

(a) End of year financial adjustments may be determined after the financial year end outside of the Amendment Window process.

(b) The scope will be defined by the Department and informed by Queensland Government Central Agency requirements.

(c) The Department will provide the HHS with a reconciliation of all Service Agreement funding and purchased activity for the prior financial year. This will reflect the agreed position between the Parties following conclusion of the end of year financial adjustments process.

(d) The impact of end of year financial adjustments on subsequent year funding and activity will be incorporated in the Service Agreement through the Deed of Amendment executed following the next available Amendment Window.

(e) This clause will survive expiration of this Service Agreement.

Schedule 6 Definitions

In this Service Agreement:

Activity Based Funding (ABF) means the funding framework for publicly-funded health care services delivered across Queensland. The ABF framework applies to those Queensland public sector health service facilities which are operationally large enough and have the systems which are required to support the framework. The ABF framework allocates health funding to these hospitals based on standardised costs of health care services (referred to as 'activities') delivered. The Framework promotes smarter health care choices and better care by placing greater focus on the value of the health care delivered for the amount of money spent.

Adjustment Notice means the written notice of a proposed funding adjustment made by or on behalf of the Chief Executive in accordance with the terms of this Service Agreement.

Administrator of the National Health Funding Pool means the position established by the *National Health Reform Amendment (Administrator and National Funding Body) Act 2012* for the purposes of administering the National Health Funding Pool according to the National Health Reform Agreement.

Agreement means this Service Agreement.

Ambulatory Care means the care provided to hospital patients who are not admitted to the hospital, such as patients of emergency departments and outpatient clinics. Can also be used to refer to care provided to patients of community-based (non-hospital) healthcare services.

Amendment Proposal means the written notice of a proposed amendment to the terms of this Service Agreement as required under section 39 of the *Hospital and Health Boards Act 2011*.

Amendment Window means the period within which Amendment Proposals are negotiated and resolved. Amendment Windows commence on the relevant Exchange Date as specified in Table 14 Schedule 5 and end at the conclusion of the Negotiation Period.

Block Funding means funding for those services which are outside the scope of ABF.

Business Day means a day which is not a Saturday, Sunday or public holiday in Brisbane.

Chair means the Chair of the Hospital and Health Board.

Chief Executive means the chief executive of the Department.

Clinical Product/Consumable means a product that has been Clinically Prescribed.

Clinically Prescribed means prescribed by appropriately qualified and credentialed clinicians relative to the product.

Clinical Prioritisation Criteria means Statewide minimum criteria to determine if a referral to specialist medical or surgical outpatients is appropriate and, if so, the urgency of that referral.

Clinical Services Capability Framework means the Clinical Services Capability Framework for Public and Licensed Private Health Facilities which provides a standard set of minimum capability criteria for service delivery and planning. The Framework outlines the minimum service requirements, staffing, support services and risk considerations for both public and private health services to ensure safe and appropriately supported clinical service delivery. It applies to all public and private licensed facilities in Queensland. References to the Clinical Services Capability

Framework in this Service Agreement mean the most recent approved version unless otherwise specified.

Community Health Service means non-admitted patient Health Services, excluding hospital outpatient services, typically delivered outside of a hospital setting.

Deed of Amendment means the resolved amendment proposals.

Department means the department administering the *Hospital and Health Boards Act 2011* (Qld), which, at the date of this Service Agreement is known as 'Queensland Health'. To avoid any doubt, the term does not include the Hospital and Health Services.

D-SA Contact Person means the position nominated by the Department as the primary point of contact for all matters relating to this Service Agreement.

Effective Date means1 July 2019.

Efficient Growth means the increased in-scope activity-based services delivered by a HHS measured on a year to year basis in terms of both the Queensland efficient price for any changes in the volume of services provided and the growth in the national efficient price of providing the existing volume of services.

Eligible Population (Oral Health Services) refers to the proportion of the population for whom publicly funded oral health services is to be provided and is defined by the following criteria:

(a) adults, and their dependents, who are Queensland residents; eligible for Medicare and, where applicable, currently in receipt of benefits from at least one of the following concession cards:

 (i) Pensioner Concession Card issued by the Department of Veteran's Affairs;

 (ii) Pensioner Concession Card issued by Centrelink;

 (iii) Health Care Card (this includes Low Income Health Care Card Holders who are automatically eligible for services);

 (iv) Commonwealth Seniors Health Card;

 (v) Queensland Seniors Card.

(b) children who are Queensland residents or attend a Queensland school, are eligible for Medicare, and are:

 (i) eligible for dental program/s funded by the Commonwealth Government; or

 (ii) four years of age or older and have not completed Year 10 of secondary school; or

 (iii) dependents of current concession card holders or hold a current concession card.

Exchange Date means the date on which the Parties must provide Amendment Proposals for negotiation, as specified in Table 14 Schedule 5.

Extraordinary Amendment Window means an Amendment Window that occurs outside of the Amendment Windows specified in Table 14 Schedule 5, in accordance with the provisions of clause 3.3 of Schedule 5.

Force Majeure means an event:

(a) which is outside of the reasonable control of the Party claiming that the event has occurred; and

(b) the adverse effects of which could not have been prevented or mitigated against by that Party by reasonable diligence or precautionary measures, and includes lightning, earthquake, fire, cyclone, flood, natural disasters, health pandemics, acts of terrorism, riots, civil disturbances, industrial disputes and strikes (other than strikes involving that Party, its' agents, employees or suppliers), war (declared or undeclared), revolution, or radioactive contamination.

Formal Agreement means an agreed set of roles and responsibilities relating to the provision and receipt of services designated as Statewide or Regional:

(a) Statewide or Regional service provision

- (i) ensure equitable and timely access to entire catchment (clinical and non-clinical)
- (ii) provide training and consultation Services where this is appropriate within the agreed model of care (clinical and non-clinical)
- (iii) timely discharge or return of patients to their place of residence (clinical Services)
- (iv) adequate communication practices to enable ongoing effective local health care, including with the patient's General Practitioner where required (clinical Services)

(b) Recipient HHS

- (i) utilisation of standardised referral criteria, where they exist, to ensure appropriate use of Statewide Services (clinical services)
- (ii) timely acceptance of patients being transferred out of Statewide Services (back-transfers) (clinical Services)
- (iii) equitable access to ongoing local health care as required (clinical services)

Health Executive means a person appointed as a health executive under section 67(2) of the *Hospital and Health Boards Act 2011.*

Health Service has the same meaning as set out in section 15 of the *Hospital and Health Boards Act 2011.*

Health Service Chief Executive means a health service chief executive appointed for an HHS under section 33 of the *Hospital and Health Boards Act 2011.*

Health Service Employee means all person, appointed as a 'health service employee' for the HHS under section 67(1) of the *Hospital and Health Boards Act 2011.*

Hospital and Health Board means the hospital and health board appointed under section 23 of the *Hospital and Health Boards Act 2011*.

Hospital and Health Service or **HHS** means the Hospital and Health Service to which this Agreement applies unless otherwise specified.

HHS-SA Contact Person means the position nominated by the HHS as the primary point of contact for all matters relating to this Service Agreement.

HR Management Functions means the formal system for managing people within the HHS, including recruitment and selection; onboarding; induction and orientation; capability, learning and development; industrial and employee relations; performance management; work health and safety and well-being; workforce planning; diversity and inclusion; and workforce consultation, engagement and communication.

Industrial Instrument means an industrial instrument made under the *Industrial Relations Act 2016.*

Inter-HHS Dispute means a dispute between two or more HHSs.

Key Performance Indicator means a measure of performance that is used to evaluate the HHSs success in meeting key priorities.

Low Benefit Care means use of an intervention where evidence suggests it confers no or very little benefit on patients, or the risk of harm exceeds the likely benefit.

Minister means the Minister administering the *Hospital and Health Boards Act 2011* (Qld).

National Health Reform Agreement means the document titled *National Health Reform Agreement* made between the Council of Australian Governments (CoAG) in 2011, and incorporating all subsequent amendments agreed between the Commonwealth of Australia and the States and Territories.

Negotiation Period means a period of no less than 15 business days (or such longer period agreed in writing between the Parties) from each Exchange Date.

Notice of Dispute means the written notice of a dispute provided by the Chief Executive or the HHS to the other party or the written notice of a dispute provided by an HHS to another HHS.

Outcome Indicator means a measure of performance that provides information on specific activities and interventions where there is a reasonable expectation or evidence that they make a positive contribution towards improving the health status and experience of patients;

Outreach Service means a Health Service delivered on sites outside of the HHS area to meet or complement local service need. Outreach services include Health Services provided from one HHS to another as well as Statewide Services that may provide Health Services to multiple sites.

Own Source Revenue means, as per Section G3 of the *National Healthcare Agreement*, 'private patients, compensable patients and ineligible persons may be charged an amount for public hospital services as determined by the state and territory'. The funding for these patients is called own source revenue and includes:

(a) Medicare ineligible patients, such as overseas visitors (not covered under reciprocal agreements), people in community detention and overseas students studying in Australia

(b) compensable patients with an alternate funding source, such as:

   (i) workers' compensation insurers;

   (ii) motor vehicle accident insurers;

   (iii) personal injury insurers;

   (iv) Department of Defence; and/or

   (v) Department of Veterans' Affairs; and

   Medicare eligible patients can elect to be treated as a public or private patient, allowing HHS' to recoup a portion of the healthcare service delivery cost.

Party means each of the Chief Executive and the HHS to which this Service Agreement applies.

Patient Identifiable Data means data that could lead to the identification of an individual either directly (for example by name), or through a combination of pieces of data that are unique to that individual.

Performance Review Meeting means the forum established which routinely reviews and discusses a range of aspects of HHS and system wide performance in accordance with the accountabilities contained within this Service Agreement and the Performance and Accountability Framework. Attendance at Performance Review Meetings comprises:

(a) the D-SA Contact Person and the HHS-SA Contact Person;

(b) executives nominated by the Department; and

(c) executives nominated by the HHS.

Performance Measure means a quantifiable indicator that is used to assess how effectively the HHS is meeting identified priorities and objectives.

Person Conducting a Business or Undertaking takes the meaning as defined in the *Work Health and Safety Act 2011,* section 5.

Prevention Services means programs that prevent illness and injury, promote health and wellbeing, create healthy and safe environments, reduce health inequalities and address factors in those communities whose health status is the lowest.

Primary Care means first level healthcare provided by a range of healthcare professionals in socially appropriate and accessible ways and supported by integrated referral systems. It includes health promotion, illness prevention, care of the sick, advocacy and community development.

Public Health Event of State Significance means an event where the actual or potential impact extends beyond the community service by a particular Hospital and Health Service.

Public Sector Health Service has the same meaning as set out in the *Hospital and Health Boards Act 2011.*

Public Sector Health System means the Queensland public sector health system, which is comprised of the Hospital and Health Services and the Department.

Quality Improvement Payment (QIP) means a non-recurrent payment due to the HHS for having met the goals set out in the QIP Purchasing Incentive Specification.

Queensland Government Central Agency means one or all of the Department of the Premier and Cabinet, Queensland Treasury, the Queensland Audit Office, the Public Service Commission and the Office of the Integrity Commissioner.

Regional Service means a clinical (direct or indirect patient care) or non-clinical Health Service funded and delivered, or coordinated and monitored, by an HHS with a catchment of two or more HHSs, but not on a Statewide basis as defined in this Schedule. Service delivery includes facility based, outreach and telehealth service models.

Referral Pathway means the process by which a patient is referred from one clinician to another in order to access the Health Services required to meet their healthcare needs.

Residential HHS means the HHS area, as determined by the *Hospital and Health Boards Regulation 2012*, in which the patient normally resides.

Safety and Quality Marker means a measure of performance that provides timely and transparent information on the safety and quality of Health Services provided by the HHS;

Schedule means this Schedule to the Service Agreement.

Senior Health Service Employee means a person appointed under section 67(2) of the *Hospital and Health Boards Act 2011* in a position prescribed as a 'senior health service employee position' under the *Hospital and Health Boards Regulation 2012.*

Service Agreement means this service agreement including the Schedules and annexures, as amended from time to time.

Service Agreement Value means the figure set out in Schedule 2 as the expected annual value of the services purchased by the Department through this Service Agreement.

State means the State of Queensland.

Statement of Building Portfolio Compliance means a declaration completed by the HHS stating that it has maintained compliance with all mandatory Acts, Regulations, Australian Standards and Codes of Practice applicable to the HHS' building portfolio.

Statewide Service means a service that is delivered by a lead provider to the State. A Statewide Service may be:

(a) a clinical service that is:

(i) a low volume, highly specialised Health Service delivered from a single location;

(ii) a highly specialised, or high risk[11], Health Service delivered in multiple locations or

(iii) a prevention and/or health promotion service.

(b) a support service that is required to enable the delivery of specific direct clinical services; or

(c) services that have a primary role to provide clinical education services and/or training programs.

Statewide Service Description means a document that defines the Service to be provided by the HHS on a statewide basis and how the Statewide Service will be accessed and used by other HHSs across the State, including but not limited to:

(a) an overview of the Statewide Service;

(b) components of the Statewide Service;

(c) eligibility criteria;

(d) Service referrals and pathways; and

(e) governance and capability arrangements for the Statewide Service.

---

[11] A Health Service that, due to its nature, poses an increased threat of ongoing sustainability, efficiency and affordability.

Supporting Indicator means a measure of performance that provides contextual information to support an assessment of HHS performance.

Suspend and Suspension means to cause the temporary cessation of a service provided by the HHS under the terms of this Service Agreement. Suspension may result from, but is not exclusively due to, limitations in workforce capacity or issues regarding the safety or quality of the service provided.

Telehealth means the delivery of Health Services and information using telecommunication technology, including:

(a) live interactive video and audio links for clinical consultations and education;

(b) store and forward systems to enable the capture of digital images, video, audio and clinical data stored on a computer and forwarded to another location to be studied and reported on by specialists;

(c) teleradiology to support remote reporting and provision of clinical advice associated with diagnostic images; and

(d) telehealth services and equipment for home monitoring of health.

Terminate and Termination means the permanent cessation of a service provided by the HHS under the terms of this Service Agreement.

Treating HHS means the HHS area, as determined by the *Hospital and Health Boards Regulation 2012*, in which a patient is receiving treatment.

Value-Based Healthcare means delivering what matters most to patients in the most efficient way. Value-Based Healthcare is characterised by:

(a) the identification of clearly defined population segments of patients with similar needs around which clinically integrated teams organise and deliver care, rather than designing and organising care around medical specialities, procedures or facilities;

(b) a focus on delivering health outcomes and the experience of receiving care as defined from the patient perspective, not just the system or clinical perspective;

(c) connection between outcomes and the costs required to deliver the outcomes; and

(d) an integrated approach across the full cycle of care with a focus on the goal of health rather than just treatment.

Key Documents

Hospital and Health Services Service Agreements and supporting documents including:

(a) Hospital and Health Services Service Agreements

(b) Queensland Health System Outlook to 2026 for a sustainable health service

(c) Performance and Accountability Framework 2020/21

(d) Purchasing Policy and Funding Guidelines 2020/21

are available at: www.health.qld.gov.au/system-governance/health-system/managing/agreements-deeds

My health, Queensland's future: Advancing health 2026

www.health.qld.gov.au/__data/assets/pdf_file/0025/441655/vision-strat-healthy-qld.pdf

Queensland Health 2020-2021 System Priorities

[link to follow]

Department of Health Strategic Plan

www.health.qld.gov.au/system-governance/strategic-direction/plans/doh-plan

Guideline for Immunisation Services

https://www.health.qld.gov.au/__data/assets/pdf_file/0026/147545/qh-gdl-955.pdf

Queensland Health Statement of Action towards Closing the Gap in health outcomes

https://qheps.health.qld.gov.au/atsihb/html/statement-of-action

HHS Performance Measures and Attribute Sheets

https://qheps.health.qld.gov.au/purchasing-performance/hhs-agreements/performance-kpis

Data Supply Requirements

https://qheps.health.qld.gov.au/purchasing-performance/hhs-agreements/data-reporting-requirements

Australian Commission on Safety and Quality in Healthcare – National Safety and Quality Health Service Standards

https://www.safetyandquality.gov.au/standards/nsqhs-standards

Statewide Services Governance and Risk Management Framework

https://qheps.health.qld.gov.au/spb/html/statewide-services/statewide-services-governance-and-risk-management-framework

Public Health Practice Manual

https://qheps.health.qld.gov.au/__data/assets/pdf_file/0035/667754/public-health-prac-man.pdf

National Healthcare Agreement

http://www.federalfinancialrelations.gov.au/content/national_agreements.aspx

National Health Reform Agreement

www.federalfinancialrelations.gov.au/content/national_health_reform.aspx

Abbreviations

ACQSC	Aged Care Quality and Safety Commission
ABF	Activity Based Funding
ACSQHC	Australian Commission on Safety and Quality in Healthcare
CET	Clinical Education and Training
D-SA	Department – Service Agreement
HHS	Hospital and Health Service
HHS-SA	Hospital and Health Service – Service Agreement
HITH	Hospital in the Home
KPI	Key Performance Indicator
LAM	List of Approved Medicines
Non-ABF	Non-Activity Based Funding
NPA	National Partnership Agreement
NSQHS	National Safety and Quality Health Service Standards
NWAU	National Weighted Activity Unit
PBS	Pharmaceutical Benefits Scheme
QAS	Queensland Ambulance Service
QIP	Quality Improvement Payment
QWAU	Queensland Weighted Activity Unit
RACGP	Royal Australian College of General Practitioners
SA2	Statistical Area Level 2

This page is intentionally left blank.

Queensland Health
www.health.qld.gov.au

Queensland Government

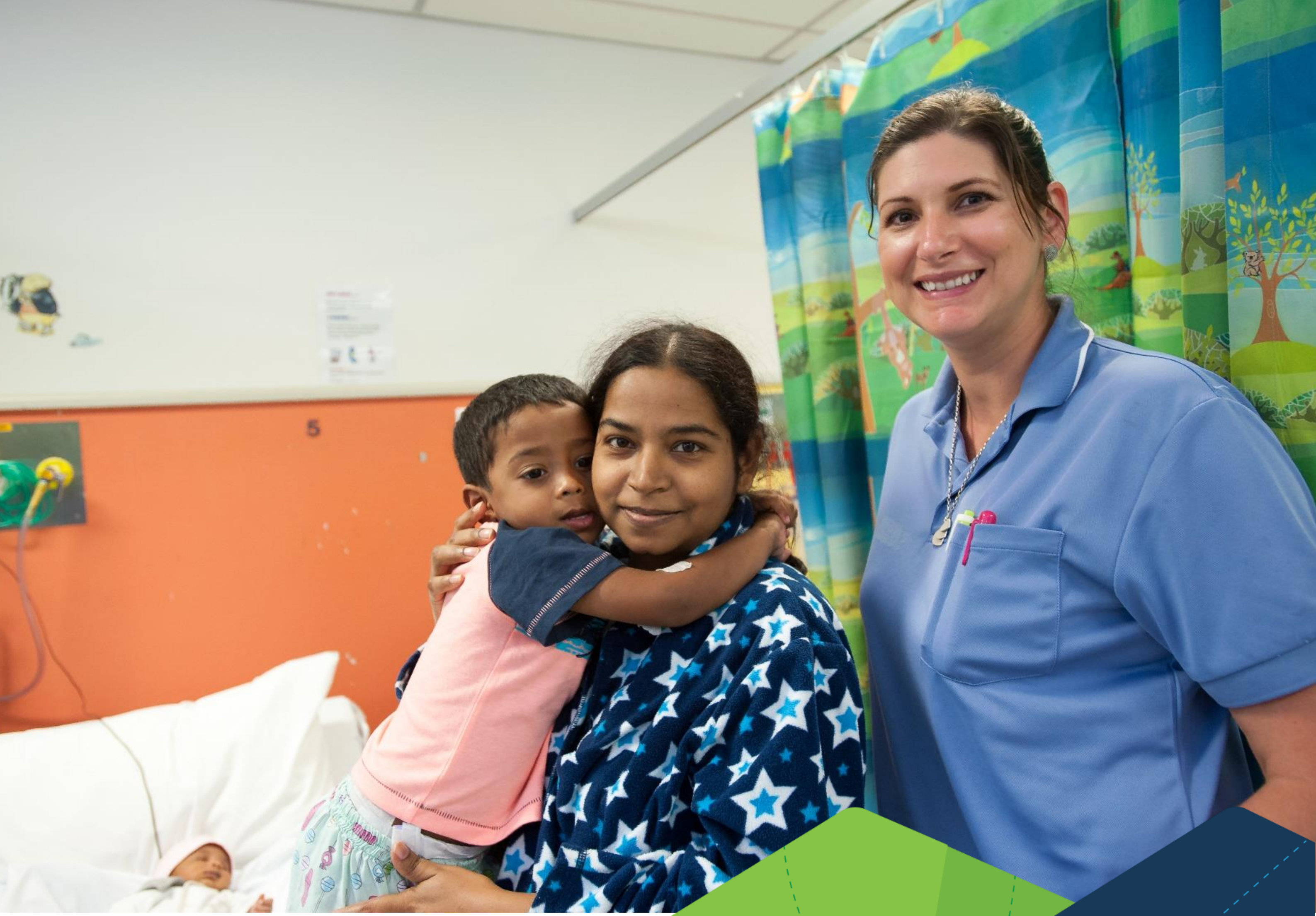

Queensland Health

Service Agreement 2022/23 – 2024/25

Cairns and Hinterland Hospital and Health Service

December 2024 Revision

Queensland Government

Cairns and Hinterland Hospital and Health Service, Service Agreement 2022/23 - 2024/25

Published by the State of Queensland, (Queensland Health), December 2024

This document is licensed under a Creative Commons Attribution 3.0 Australia licence. To view a copy of this licence, visit creativecommons.org/licenses/by/3.0/au

© State of Queensland (Queensland Health) **2024**

You are free to copy, communicate and adapt the work, as long as you attribute the State of Queensland (Queensland Health).

For more information contact:

Contracting and Performance Management Branch, Department of Health, GPO Box 48, Brisbane QLD 4001
Tel (+61) (07) 3708 5869

Email SAM@health.qld.gov.au

An electronic version of this document is available at www.health.qld.gov.au/system-governance/health-system/managing/default.asp

Disclaimer:
The content presented in this publication is distributed by the Queensland Government as an information source only. The State of Queensland makes no statements, representations or warranties about the accuracy, completeness or reliability of any information contained in this publication. The State of Queensland disclaims all responsibility and all liability (including without limitation for liability in negligence) for all expenses, losses, damages and costs you might incur as a result of the information being inaccurate or incomplete in any way, and for any reason reliance was placed on such information.

Acknowledgement

We acknowledge the Traditional and Cultural Custodians of the lands, waters, and seas across Queensland, pay our respects to Elders past and present, and recognise the role of current and emerging leaders in shaping a better health system.

We recognise the First Nations peoples in Queensland are both Aboriginal Peoples and Torres Strait Islander Peoples, and support the cultural knowledge, determination, and commitment of Aboriginal and Torres Strait Islander communities in caring for health and wellbeing for millennia.

We recognise the ancestral lands of the many traditional and cultural custodians, which comprises the Cairns and Hinterland geographical footprint, where we work to provide safe and quality health services.

From the lands of the Kuku Yalanji in the north (Mossman), Tagalaka in the west (Croydon) and Girramay in the south (Tully) and all other tribes and clans therein, including the diaspora of Torres Strait Islanders and other first peoples who made this region their home, we offer our deep respect.

Cairns and Hinterland Hospital and Health Service is proud to recognise and celebrate the cultural diversity of our communities and workforce at the following locations:

Location	Traditional Custodians
Atherton Hospital	Tableland Yidinji
Babinda Multipurpose Health Service	Wanyurr Majay
Cairns Hospital	Gimuy Walaburra Yidinji & Yirrganydji
Cow Bay	Kuku Yalanji
Chillagoe Hospital	Wakaman
Croydon Primary Health Care	Tgalaka
Dimbulah Primary Health Care	Bar Barrum
Forsayth and Georgetown Primary Health Care	Ewamian
Gordonvale Hospital	Malanbarra Yidinji
Gurriny Yealamucka Health Service	Gunggandji & Mandingalbay Yidinji
Herberton Hospital	Jirrabal
Innisfail Hospital	Mamu
Jumbun Primary Health Care	Girramay
Kuranda Medical Service	Djabugay
Malanda Primary Health Care	Ngadjon Jii
Mareeba Hospital	Muluridji
Millaa Millaa Primary Health Care	Mamu
Mission Beach Community Health Centre	Djiru Warrangburra
Mossman Multipurpose Health Service	Kuku Yalanji
Mount Garnet Primary Health Care	Bar Barrum
Ravenshoe Primary Health Care	Jirrabal
Tully Hospital	Gulnay

Contents

Acknowledgement ... 3
Contents ... 4
Tables ... 5
1. Introduction ... 6
2. Scope ... 6
3. Services ... 6
4. Performance and Accountability Framework ... 7
5. Outcomes Framework ... 7
6. Data supply requirements ... 8
7. Hospital and Health Service accountabilities ... 8
8. Department accountabilities ... 10
9. Achieving health equity with First Nations Queenslanders ... 10
10. Dispute Resolution ... 11
11. General ... 11
12. Counterparts ... 12
Execution ... 13
Schedule 1 HHS profile ... 14
1. HHS profile ... 14
Schedule 2 Funding and purchased activity and services ... 15
1. Introduction ... 15
2. Purchased health services ... 16
3. Teaching, training, and research ... 18
4. Delivery of purchased activity ... 20
5. Delivery of purchased services ... 21
6. Financial adjustments ... 21
7. Funding sources ... 25
8. Funds disbursement ... 25
Schedule 3 Department of Health Provided Services ... 32
1. In scope services and service schedules ... 32
Schedule 4 Performance Measures ... 33
1. Performance Measures ... 33
Schedule 5 Amendments to this service agreement ... 37
1. Agreed process to amend this service agreement ... 37
Appendix 1 Reference Documents ... 39

Tables

Table 1 Statewide Services....16
Table 2 Financial adjustments applied on breach of activity thresholds....22
Table 3 Funding Adjustments 2024/25....22
Table 4 HHS Funding by Outcomes Framework Care Domain....30
Table 5 HHS Total Funding Allocation by Funding Source 2024/25....30
Table 6 National Health Reform Funding....29
Table 7 Discretely Funded Programs (Non-ABF)....31
Table 8 Department of Health Provided Services and Service Schedules....34
Table 9 HHS Performance Measures – Key Performance Indicators....35
Table 10 HHS Performance Measures – Safety and Quality Markers....34
Table 11 HHS Performance Measures – Outcome Indicators....35
Table 12 Statewide Health Equity Key Performance Measures....36

1. Introduction

1.1 In performing this service agreement, the Department and the Hospital and Health Service (HHS) will act consistent with the object of the *Hospital and Health Boards Act 2011* (Qld) (Act).

1.2 The Department and the HHS operate as part of a networked system and agree to work collaboratively with each other, with other HHSs and with the Queensland Ambulance Service in the best interests of the Queensland public sector health system.

1.3 The parties will ensure that planning and delivery of health services is consistent with the strategies and priorities set out by government. The parties recognise the importance of the HHS' Health Equity Strategy (as defined in the Act) and the parties' commitment to improving health and wellbeing outcomes and achieving health equity for Aboriginal and Torres Strait Islander peoples.

2. Scope

2.1 This service agreement covers the period from 1 July 2022 to 30 June 2025.

2.2 The parties have identified the services to be provided by the HHS, the funding for the provision of those services, the performance measures applicable to the services and data requirements.

3. Services

3.1 In delivering services, the HHS is required to meet:

(a) the applicable conditions of all national agreements and national partnership agreements between the Queensland Government and the Commonwealth Government and commitments under any related implementation plans; and

(b) the applicable conditions of each agreement or arrangement for funding between the Department and the HHS and commitments under any related implementation plans, which conditions and commitments may be recorded separately to this service agreement but for which funding is (or becomes) provided in Schedule 2.

3.2 The HHS is required to deliver the services outlined in this service agreement for which funding is provided in Schedule 2.

3.3 Where issues arise which prevent the HHS from providing a service or necessitate a reduction in the level or scope of a service provided, prompt notification must be made to the Department and impacted HHSs, with appropriate details. The HHS must minimise any clinical risk or adverse impact to patient experience that may result from service disruption. The Department will respond to the HHS on any requirements it has concerning service delivery and any adjustments triggered by under delivery.

3.4 If the HHS wishes to terminate or reduce service levels for a service for which funding is provided in Schedule 2, this will remain subject to negotiation and agreement by the Department at its discretion.

3.5 For any new services proposed during the term of this service agreement, the parties agree:

(a) the service must meet a demonstrated clinical need and provide value for money;

(b) the commencement of a new service, including the implementation of new models of care, may occur where the service has been commissioned by the Department, a funding stream is in place and any conditions relating to the funding have already been agreed; and

(c) if the HHS wishes to commence providing a new service that has not been commissioned by the Department, this will remain subject to agreement by the Department at its discretion.

3.6 It is acknowledged that there may, from time to time, need to be service delivery changes between HHSs. Management of inter-HHS relationships should be informed by the following principles:

(a) HHSs should maintain the proportion of out of HHS work undertaken unless as a result of agreed repatriation of patients;

(b) each HHS should manage patients from its own catchment population if it is within its clinical capability to do so as specified by the Clinical Services Capability Framework; and

(c) where it is proposed that a service move from one HHS to another, the Department will consider, as part of its review under clauses 3.4 and 3.5, whether the respective Health Service Chief Executives endorse the proposed change in patient flows; and the funding required to follow the patient.

4. Performance and Accountability Framework

4.1 The Queensland Health Performance and Accountability Framework (the Performance and Accountability Framework) sets out the framework within which the Department monitors and manages the performance of public sector health services in Queensland.

4.2 The parties will act consistent with the Performance and Accountability Framework.

5. Outcomes Framework

5.1 Queensland Health is embarking on a strategic shift in funding focus from “volume” to “outcome” using the Outcomes Framework. This approach aims to link the resources and services required and delivered as part of healthcare activities, to health outcomes for individuals and the population.

5.2 The Outcomes Framework takes a three-tiered approach:

(a) The System Tier (Tier 1), which acts as a strategic tier, and includes four domains to measure the contribution of Queensland Health to the system outcomes.

(b) The Operational Tier (Tier 2) which includes nine (9) Clinical Care Domains, reflecting areas that are important to deliver change and improvement in the short to medium term, and to operationalise the Outcomes Framework.

(c) The Tactical Tier (Tier 3) provides scaffolding to select initiatives for implementation as specific pressures arise. These pressures may include areas identified for improvement through Tier 2.

5.3 In consultation with the State-wide Clinical Networks the indicators below are under further development and shadowing.

Indicator	Care Domain	Clinical Leadership
Percentage of patients who have HBA1C ordered during hospital admission	Chronic and Complex	Diabetes Network
Time to treatment for breast, colorectal and lung cancers	Cancer Care	Cancer Care Network

5.4 Schedule 4 maps existing indicators in the Performance and Accountability Framework to the care domains of the Outcomes Framework.

6. Data supply requirements

6.1 The HHS will provide the Department with all clinical and non-clinical data that is reasonably required to support the effective management of the public sector health system. This will include, but is not limited to, data that is required to:

(a) fulfil legislative obligations;

(b) deliver accountabilities and obligations to State and Commonwealth Governments; including related to the provision and reconciliation of activity data by the Administrator of the National Health Funding Pool;

(c) monitor and support performance improvement;

(d) manage this service agreement;

(e) support clinical innovation; and

(f) facilitate evaluation and audit.

6.2 The parties agree and acknowledge that:

(a) the Department will keep the HHS informed of the Department's data requirements; and

(b) data will be provided as required, or permitted, by law.

6.3 Further details on data supply requirements, including principles that guide the collection, storage, transfer and disposal of data and prescribed timeframes for data submission, are provided online as detailed in Appendix 1.

7. Hospital and Health Service accountabilities

7.1 The HHS will perform its obligations under this service agreement.

7.2 As applicable to the HHS and its services, the HHS will comply with:

(a) legislation and subordinate legislation, including the Act;

(b) cabinet decisions;

(c) Ministerial directives;

(d) agreements entered into between the Queensland and Commonwealth governments (or agreements with others in furtherance of such agreements), of which it is informed;

(e) agreements entered into between the Department and other Queensland Government entities, of which it is informed;

(f) agreements entered into with another HHS(s), including Networked Services Agreements;

(g) all industrial instruments;

(h) all health service directives and health employment directives; and

(i) all policies, guidelines, and implementation standards, including human resource policies.

7.3 As part of the commitment to achieving First Nations health equity, the HHS will prioritise the elimination of racial discrimination and institutional racism within its service and ensure that Aboriginal and Torres Strait Islander peoples have access to culturally safe and responsive health services.

7.4 The HHS will ensure that effective health service planning and delivery systems are in place, working in collaboration with the Department.

7.5 To support the achievement of the Queensland-Commonwealth Partnership's (QTP's) vision and commitment to work together to tackle health system challenges that cannot be overcome by any one organisation, HHSs are required to prepare and submit Joint Regional Needs Assessments in accordance with the framework provided online as detailed in Appendix 1.

7.6 HHSs must operate clinical service delivery consistent with the National Quality and Safety Standards. The HHS is expected to escalate any concerns that arise at the conclusion of a formalised assessment.

7.7 The HHS will ensure that health service employees employed by the Chief Executive[1] who perform work for the HHS are managed in accordance with any applicable delegations and directions from the Chief Executive. The HHS will ensure that effective asset management systems are in place (available online, as detailed in Appendix 1), that comply with the *Queensland Government Building Policy Framework and Guideline*, while working in collaboration with the Department.

7.8 The HHS will maintain accreditation to the standards required by the Department.

7.9 The HHS will appropriately perform and fulfil its functions under the Act.

[1] In this service agreement the term Chief Executive takes the meaning applied in the Act, which is the Chief Executive of Queensland Health, but which is generally referred to as the Director-General.

7.10 The HHS will provide to the Chief Executive reports of a type, and at the intervals, agreed between the parties, or as reasonably specified by the Chief Executive.

8. Department accountabilities

8.1 The Department will perform its obligations under this service agreement including, in return for the HHS performing its obligations and delivering the services, providing funding to the HHS as stipulated in this service agreement (as amended).

8.2 The Department will:

(a) comply with applicable legislation and subordinate legislation, including the Act, as relates to this service agreement;

(b) perform the system manager role (as defined in the Act) through the Chief Executive; and

(c) provide a range of services to the HHS as set out in Schedule 3.

8.3 The Chief Executive will appoint health service employees to perform work for the HHS for the purpose of enabling the HHS to perform its functions under the Act.

8.4 The Chief Executive will consult, cooperate, and coordinate with the HHS to ensure legal compliance with the *Work Health and Safety Act 2011* and other legislation as it applies to the scope, nature and location of operations associated with this service agreement.

8.5 The Chief Executive will appropriately perform and fulfil their functions under the Act.

9. Achieving health equity with First Nations Queenslanders

9.1 Through legislative amendments to the Act and the Hospital and Health Boards Regulation 2012, and the release of *Making Tracks Together: Queensland's Aboriginal and Torres Strait Islander Health Equity Framework* (2021), Queensland Health has strengthened its commitment to improving health and wellbeing outcomes and achieving health equity with First Nations peoples.

9.2 The HHS will develop and resource a First Nations Health Equity Strategy, compliant with legislative requirements. An implementation plan, accompanying the strategy, demonstrates the HHS's activities and key performance measures to achieve health equity with First Nations peoples. The Health Equity Strategy will act as the principal accountability mechanism between the Aboriginal and Torres Strait Islander community and the HHS in achieving health equity with First Nations Queenslanders and forms a key part of the Queensland Government's commitment to the *National Agreement on Closing the Gap* (2020).

9.3 The HHS is required to review the Health Equity Strategy at least once every three years and will publish the Health Equity Strategy in a way that allows it to be accessed by members of the public.

9.4 The HHS will ensure that commitment and leadership is demonstrated through implementing the actions and achieving the key performance measures outlined in the Health Equity Strategy.

9.5 The HHS will report publicly every year on progress against the Health Equity Strategy.

9.6 The HHS will support the implementation of other supplementary policies and strategies to drive health equity across the public health system, including relevant election commitments.

9.7 The HHS will participate as a partner in the implementation and achievement of Queensland's *HealthQ32 First Nations First Strategy 2032* in addition to HHS commitments within their Health Equity Strategy.

10. Dispute Resolution

10.1 Where a dispute arises in connection to this agreement, either between the department and one or more HHSs or between HHSs, every effort should be made to resolve the dispute at the local level. If local resolution cannot be achieved, the dispute resolution processes, accessible through Appendix 1, must be followed.

11. General

11.1 **Sub-contracting**

(a) The HHS must have appropriate systems in place to ensure that any subcontractor is accredited (as applicable), qualified, and otherwise fit to perform any services for which it is contracted.

(b) The HHS must ensure that any sub-contractor who has access to confidential information (as defined in the Act) or personal information (as defined in the Information Privacy Act 2009 (Qld)) complies with obligations no less onerous than those imposed on the HHS.

11.2 **Insurance**

The HHS must:

(a) hold and maintain the types and levels of insurances that the HHS considers appropriate according to its functions and obligations; and

(b) comply with reasonable requests or directions from the Department in this regard.

11.3 **Amendment**

The process for amending this service agreement is provided for under the Act and further outlined in Schedule 5.

12. Counterparts

12.1 This service agreement may be executed in two or more identical copy counterparts, each of which together will be deemed an original, but all of which together will constitute one and the same instrument.

12.2 In the event that any signature executing this service agreement or any part of this service agreement is delivered by facsimile transmission or by scanned e-mail delivery of a ‘.pdf’ format data file or equivalent of the entire agreement, the signature will create a valid and binding obligation of the party executing (or on whose behalf the signature is executed) with the same force and effect as if the signature page were an original. For the avoidance of doubt, this service agreement may be in the form of an electronic document and may be electronically signed.

12.3 For execution under this clause 12 to be valid, the entire service agreement upon execution by each individual party must be delivered to the remaining parties.

Execution

The terms of this Service Agreement were agreed under the provisions set out in the Hospital and Health Boards Act, section 35 on 29 June 2022, and were subsequently amended by the Deeds of Amendment entered into pursuant to section 39 of the Hospital and Health Boards Act 2011 and executed on 1 February 2023, 3 May 2023, 6 July 2023, 7 February 2024, 5 April 2024, 7 August 2024 and 16 December 2024.

This revised Service Agreement consolidates amendments arising from:

- Periodic Adjustment – COVID-19 Funding Transfer September 2022
- Periodic Adjustment – COVID-19 Funding Transfer October 2022
- 2022/23 Amendment Window 2 (in year variation)
- Periodic Adjustment – COVID-19 Funding Transfer December 2022
- 2022/23 Amendment Window 3 (in year variation)
- Periodic Adjustment – COVID-19 Funding Transfer April 2023
- Extraordinary Amendment Window – May 2023
- 2023/24 Amendment Window 1 (Budget Build)
- 2023/24 Amendment Window 2 (in year variation)
- 2023/24 Amendment Window 3 (in year variation)
- 2024/25 Amendment Window 1 (Budget Build)
- 2024/25 Amendment Window 2 (in year variation)

Schedule 1 HHS profile

1. HHS profile

This Schedule does not apply to this HHS.

Schedule 2 Funding and purchased activity and services

This Schedule 2 sets out:

(a) the services which are to be provided by the HHS;

(b) the activity purchased by the Department from the HHS;

(c) the funding provided for delivery of the purchased activity;

(d) the allocation of funding provided against the care domains of the Outcomes Framework;

(e) the criteria and processes for financial adjustments associated with the delivery of purchased activity and specific funding allocations; and

(f) the sources of funding that this service agreement is based on and the way these funds will be provided to the HHS.

1. Introduction

1.1 The HHS will deliver the services for which funding is provided in this Schedule 2. In providing these services, the HHS will ensure that:

(a) all statewide and national policy frameworks, guidelines, protocols and implementation standards applicable to the service provided are followed;

(b) participation in national programs is facilitated and supported, including where these programs are provided by the Commonwealth Government;

(c) service delivery partnerships, including with other HHSs, primary care organisations and non-government organisations, are maintained and operate effectively;

(d) collaboration and engagement with other service providers and stakeholders is initiated and maintained to ensure that an integrated system of treatment, care and support is in place and to facilitate the delivery of comprehensive and effective services. This may include but is not limited to:

(i) other HHSs;

(ii) non-government organisations;

(iii) Aboriginal and Torres Strait Islander community-controlled health organisations;

(iv) Queensland Ambulance Service;

(v) services provided through the Department of Health (for example, Pathology Queensland);

(vi) primary care providers;

(vii) other government departments and agencies; and

(viii) private providers;

(e) models of care and service delivery arrangements are consistent with evidence-based practice and offer value for money;

(f) services are provided on an equitable basis to the community, and processes are in place to ensure that services reach identified target populations, high risk groups and hard to reach communities;

(g) referral networks and pathways continue to operate effectively; and

(h) innovation and continuous improvement are supported.

2. Purchased health services

2.1 Table 4 shows the allocation of funding from the Department to the HHS across the care domains of the Outcomes Framework. Table 5, Table 6, and Table 7 outline the activity and service streams which the Department agrees to purchase from the HHS pursuant to this service agreement.

2.2 More generally, this will include the following:

2.3 **Statewide Services**

(a) The designation of a service as a statewide service (either a clinical statewide service or a clinical support statewide service) will be determined by the Department, consistent with the stipulated governance arrangements for such services.

(b) The HHS will:

(i) collaborate with the Department and other HHSs in the implementation of, and adherence to, the governance and oversight arrangements for statewide services;

(ii) participate in, and contribute to, the staged review of the purchasing model for identified statewide services; and

(iii) ensure that referral pathways in and out of each statewide service are followed.

2.4 **Clinical Statewide Services and Clinical Support Statewide Services provided**

This clause does not apply to this HHS.

Table 1 Statewide Services

This table does not apply to this HHS.

2.5 **Regional services**

(a) The HHS has responsibility for the provision and/or coordination of the following regional services:

(i) Basic physician training pathway

(ii) Community Forensic Outreach Service

(iii) Court liaison services

(iv) Court liaison services for young people

(v) Forensic adolescent mental health services

(vi) Mental health clinical cluster support program

(vii) Mental Health Act delegate

(viii) Mental health clinical indicator team program

2.6 **Prevention services and public health services**

(a) The HHS will provide a range of prevention and public health services to promote and protect health, prevent illness and disease, and manage risk, including:

(i) Specialist Public Health Units

(ii) environmental health services, including risk assessment, regulation and enforcement in relation to environmental hazards, food safety, medicines and therapeutic goods, mosquitos and other vectors, pest management, poisons, radiation safety, chemical safety and water quality;

(iii) communicable disease services including immunisation, blood-borne viruses, sexually transmissible infections, infection control, notifiable conditions, mosquito-borne disease and tuberculosis;

(iv) management of incidents, emergencies and disasters, and disease outbreak readiness and response services;

(v) preventive health services;

(vi) population health screening including, but not limited to, cancer screening services and newborn blood spot screening;

(vii) public health epidemiology and surveillance;

(viii) mitigation and adaptation in response to climate risks.

(b) Services will be provided in line with public health related legislation and the service delivery and reporting requirements outlined in the Public Health Service Schedule and supported by the *Public Health Practice Manual*, as these relate to the services provided.

(c) Delivery of these services may be coordinated through specialist public health units, sexual health services, tuberculosis services, other areas of the HHS, or a combination of these.

2.7 **Aboriginal and Torres Strait Islander health services**

The HHS will provide Aboriginal and Torres Strait Islander specific health services and initiatives consistent with the principles and objectives of the Queensland Government's *Making Tracks toward closing the gap in health outcomes for Indigenous Queenslanders by 2033 – Policy and Accountability Framework* and the priorities committed to in the

HHS's Health Equity Strategy. These services and initiatives will be delivered in line with guidance from the First Nations Health Office and the *First Nations First Strategy 2032.*

2.8 **Mental health, alcohol, and other drugs services**

The HHS will provide treatment, care and support for individuals who are, or may be, experiencing substance use disorders and/or other mental health disorders, mental health crisis and suicidal distress, and their families and carers. Services will be delivered in line with guidance from the Mental Health, Alcohol and Other Drugs Strategy and Planning Branch.

2.9 **Oral health services**

The HHS will provide oral health services to people who meet the eligibility criteria for accessing public dental services in Queensland. Services will be delivered in line with guidance from the Office of the Chief Dental Officer.

2.10 **Prisoner health services**

The HHS will provide services for prisoners consistent with the principles, responsibilities and requirements specified in the Memorandum of Understanding (Prisoner Health Services) between Queensland Health and Queensland Corrective Services.

2.11 **Youth detention services**

This clause does not apply to this HHS.

2.12 **Refugee health**

The HHS will operate a health service for refugees, special humanitarian entrants and asylum seekers.

2.13 **State-funded outreach services**

(a) Where state-funded outreach services are provided:

(i) funding for the service will remain part of the providing HHS's funding allocation; and

(ii) the activity must be recorded at the HHS where the outreach service is being provided.

(b) Any changes to the provision of outreach services will follow the requirements set out in clause 3 of this service agreement.

3. Teaching, training, and research

The HHS will provide the teaching, training and research programs for which funding is provided within Schedule 2 and as described below.

3.1 **Clinical education and training**

(a) The HHS will provide education and training placements for the following professional groups consistent with and proportionate to the capacity of the HHS

and will support and align with stipulated placement terms governing clinical placements in Queensland Health facilities:

(i) medical students;

(ii) nursing and midwifery students;

(iii) pre-entry clinical allied health students;

(iv) interns;

(v) rural generalist trainees;

(vi) vocational medical trainees;

(vii) first year nurses and midwives;

(viii) re-entry to professional register nursing and midwifery candidates;

(ix) dental students;

(x) allied health rural generalist training positions;

(xi) Aboriginal and Torres Strait Islander Health Workers and Aboriginal and Torres Strait Islander Health Practitioners.

(b) The HHS will comply with the state-wide vocational medical training pathways.

(c) The HHS will support profession specific and inter-profession statewide allied health clinical education programs.

(d) The HHS will continue to implement and retain the following positions provided through clauses to the *Health Practitioners and Dental Officers (Queensland Health) Certified Agreement (No 4) 2022*:

(i) health practitioner research positions provided through the Research Package for Health Practitioners; and

(ii) clinical educator positions provided through the Clinical Education Management Initiative for Health Practitioners.

(e) The HHS will maintain or increase its contribution of staff to the Queensland Country Relieving Doctors and the receiving HHS will be responsible for wages, clinical governance, and appropriate supervision of the junior medical relievers.

3.2 Statewide training, education, and research

This clause does not apply to this HHS.

3.3 Health and medical research

The HHS will:

(a) develop and implement a strategy to drive increased research activity and its translation into clinical practice;

(b) support increased and equitable access to clinical trials for patients;

(c) support clinicians to undertake research linked to their practice; and

(d) ensure high quality and timely research governance approval processes.

4. Delivery of purchased activity

4.1 The HHS is required to maintain accurate activity forecasts in the purchased target module of the Decision Support System (DSS) at all times. This information is imperative to the Department's assessment of State performance against the national Soft Cap and for outer-year planning. Activity forecasts must accurately reflect financial forecasts reported to the Finance Branch monthly.

4.2 The Department and the HHS will monitor actual activity against purchased levels and will act as necessary to ensure delivery of purchased levels is achieved. The HHS has a responsibility to only recognise revenue that is linked with actual activity delivered against purchased volumes.

4.3 The HHS will actively monitor variances from purchased activity levels and notify the Department as soon as the HHS becomes aware of significant variances.

4.4 The HHS will also notify the Department of deliberate changes to the consistent recording of activity within year that would result in additional activity being recorded for existing health services.

4.5 The HHS will undertake regular quality audits. The HHS is encouraged to publish its data quality framework describing audits undertaken and results achieved. For further information, refer to the Delivery of Purchased Activity – Requirement for Quality Audits specification sheet as detailed in Appendix 1.

4.6 If the HHS wishes to convert activity between purchased activity types, programs, and levels the HHS must negotiate this with the Department based on a sound needs based rationale.

4.7 The Department will reconcile in-scope activity, as defined in the Activity Reconciliation specification sheet (available online, as detailed in Appendix 1), delivered by the HHS against the purchased in-scope activity targets outlined in Table 5.

4.8 Activity reconciliations will be undertaken in the applicable End of Year Technical Amendment Window and subsequent Amendment Window 2 and will be derived through application of the methodology which is documented in the Activity Reconciliation specification sheet.

4.9 Should the HHS be unable to deliver the activity that has been funded a financial adjustment will be applied.

4.10 Funding and corresponding activity that is withdrawn from the HHS may be reallocated to an alternate provider that can undertake the activity.

4.11 Any resulting changes to funding and purchased activity levels will be actioned through the processes outlined in Schedules 2 and 5 of this service agreement.

4.12 The HHS will maintain its efforts to accurately record the delivered activity and submit this data to the Department in accordance with the agreed requirements.

5. Delivery of purchased services

5.1 As part of the service agreement, the Department purchases a range of services (or deliverables) from the HHS for the delivery of certain programs and projects.

5.2 These program or project services may be the subject of separately agreed conditions tied to that funding and the focus of detailed monitoring by the Department.

5.3 Conditions may include, but are not limited to:

(a) establishment and/or commencement of services;

(b) delivery of activity;

(c) workforce obligations;

(d) establishment of oversight committees;

(e) opening or upgrades to facilities;

(f) program evaluation;

(g) program management;

(h) reporting or notification obligations; and

(i) attainment of performance standards.

5.4 The HHS will ensure that the conditions are achieved within the stipulated time period.

5.5 The HHS will notify the Department if the HHS forecasts an inability to achieve program or project objectives or the conditions.

5.6 The Department may withdraw allocated funding pro rata to the level of under delivery if the services for a specified program or project are not being fully delivered according to the objectives or conditions.

5.7 Any resulting changes to funding and purchased activity levels will be actioned through the processes outlined in Schedules 2 and 5 of this service agreement.

6. Financial adjustments

6.1 Activity targets

(a) The Department will initiate a joint process with the HHS to determine whether a financial adjustment should be applied in relation to any purchased activity which has breached the thresholds identified bi-annually. This process will take into account any relevant matters that have been identified in a review/analysis of the breach as well as the outcomes of the activity plan implemented to address the activity breach.

(b) Activity will be monitored at the purchasing hierarchy level. Providing the HHS meets all relevant KPIs and specific funding allocations, the HHS has the ability to negotiate the transfer of activity across the purchasing hierarchy with the Department.

(c) Table 2 demonstrates the financial adjustment that will be applied when activity thresholds have been breached.

(d) The HHS may not utilise the provisions within AASB15 Revenue from Contracts with Customers to override the application of any financial adjustment made by the Department in line with Table 2.

Table 2 Financial adjustments applied on breach of activity thresholds

Example of Breach	Description	Financial Adjustment
Over performance	Activity exceeds that specified in the service agreement value for in-scope activity as shown in Table 5.	Purchasing contracts are capped and an HHS will not be paid for additional activity apart from activity that is in scope for the identified purchasing incentives as set out in Table 3 (where applicable.)
Under performance	Activity is below that specified for in-scope activity as shown in Table 5.	Purchased activity and the related funding will be withdrawn at 100% of the Queensland Efficient Price and reallocated to an alternate provider that can undertake the activity. The reconciliation will be undertaken as outlined in the Activity Reconciliation Specification. Refer to Table 5 for the HHS QWAU target.
Failure to deliver on service commitments linked to specific funding allocations	Specific funding allocations<br>National Partnership Agreements.	It is at the discretion of the Department to withdraw allocated funding pro rata to the level of under delivery.
For all other types of activity variance, any financial adjustment will be made at the discretion of the Department.		

6.2 Purchasing approach

(a) The purchasing approach includes a range of funding adjustments (purchasing incentives and ABF model localisations) that aim to incentivise high quality and high priority activity, support innovation and evidence-based practice, deliver additional capacity through clinically and cost-effective models of care and dis-incentivise care providing insufficient or no benefit for patients. This includes incentive payments for HHSs who achieve targets in specific priority areas. The funding adjustments are detailed in Table 3. The Department must reconcile the applicable funding adjustments in Table 3 in line with the timeframes specified in the relevant purchasing specification sheet. The Department must provide a copy of the reconciliation statement to the HHS.

(b) Funding adjustments must be based on the requirements contained in the relevant purchasing specification sheet.

(c) Any resulting changes to funding and purchased activity levels will be actioned through the processes outlined in Schedules 2 and 5 of this service agreement.

Table 3 Funding adjustments 2024/25

Funding adjustment	
**Purchasing incentives**	
Models of care/workforce	This program includes a range of initiatives focusing on incentivising:<br>• specific models of care; and<br>• the use of workforce operating at top of scope where there may be long wait lists and staff have not been available in a traditional model of care.
**ABF model localisations**	
Child Health Checks	QWAU loading for every in-scope check performed.
Unqualified neonate funding	Reduced Diagnosis Related Group (DRG) QWAU for all maternal delivery episodes with a liveborn outcome, discounted by the Diagnosis Related Group (DRG), with QWAUs re-allocated for unqualified neonates.
Maternity care for First Nations women	QWAUs to incentivise maternity care provided to First Nations mothers during pregnancy and to incentivise smoking cessation during pregnancy.
Sentinel events	Payment withdrawn for sentinel events as per the national ABF model.
Advance Care Planning (ACP)	QWAUs for HHSs offering ACP discussions to admitted patients, non-admitted outpatients, community health patients and Emergency Department patients.
Emergency Department Did Not Wait (DNW)	Zero QWAUs for DNWs.
Fractured neck of femur	QWAUs reduced by 20% for non-timely surgical treatment of fractured neck of femur.
Hospital in The Home (HITH)	QWAUs increased by 12.5% for Hospital in the Home (HITH) admissions of Residential Aged Care Facility residents.
Out-of-scope services	Nil QWAUs for out-of-scope procedures.
Pre-operative bed days	QWAUs for long stay days above the upper trim point less ICU days reduced equivalent to pre-operative days, up to a maximum of 3 days, for elective episodes.
Smoking cessation (community mental health)	QWAUs for smoking cessation activity for community mental health patients.
Smoking cessation (inpatients)	QWAUs for smoking cessation activity for publicly funded inpatients.
Stroke care	10% QWAU loading for acute stroke patients admitted to Queensland Stroke Clinical Network-endorsed stroke unit care.
Telehealth (admitted patients)	QWAUs for provider-end of in-scope admitted patient telehealth activity.
Allied Health Led Workforce for Pelvic Health and Gastroenterology	QWAU loading for an in-scope service event for Pelvic Health and Gastroenterology recorded against an Other Health Professional.
Remote Patient Monitoring	QWAU loading for an in-scope non-admitted remote patient monitoring encounter per month per patient.

Surgery Connect reimbursements

(d) The HHS will reimburse the Department for the actual costs of activity for nominated patient referrals to the Surgery Connect program where:

(i) The HHS has nominated the patient referral as HHS funded or HHS Direct on entry of the referral in the Surgery Connect Activity Navigator (SCAN); and

(ii) The HHS Chief Finance Officer has recorded approval of the nomination in SCAN;

or

(iii) The HHS has obtained the Department's agreement to retrospectively convert a defined patient cohort to HHS funded status in SCAN.

(e) The HHS may only request retrospective conversion of activity to HHS funded within the financial year in which the activity has taken place.

(f) Any resulting changes to funding and purchased activity levels will be actioned through the processes outlined in Schedules 2 and 5 of this service agreement.

6.3 Financial adjustments – other

(a) Notwithstanding the provisions regarding the recognition of revenue as stipulated in AASB1058 Income of Not-for-Profit Entities and/or AASB15 Revenue from Contracts with Customers, the Department may seek to recover funding from an HHS that was provided through this service agreement which has:

(i) not been utilised in accordance with its intended purpose; and/or

(ii) not been utilised within the prescribed time period to deliver the agreed outcomes/services.

(b) If the Chief Executive (or delegate) determines that previously allocated funding is to be recovered, any resulting changes to funding and purchased activity levels will be actioned through the processes outlined in Schedules 2 and 5 of this service agreement.

6.4 Public and private activity/own source revenue

(a) Own source revenue comprises grants and contributions, user charges and other revenues.

(b) Where an HHS is above its own source revenue target, it will be able to retain the additional own source revenue with no compensating adjustments to funding from other sources where the additional revenue is not attributable to a private patient consistent with clauses A9 to A13 and A44 of the *National Health Reform Agreement.*

(c) Where an HHS is below its own source revenue target in respect of private patients, it will experience a reduction in revenue with no compensating adjustments to funding from other sources.

(d) The HHS will make all efforts to identify and maximise revenue from appropriate third parties to ensure optimisation of available State and Commonwealth funding across the Queensland public sector health system.

(e) The own source revenue identified in Table 5 is an estimate generated by the HHS which allows all third-party funding sources associated with service delivery

to be identified. The HHS will ensure that this estimate is substantiated and accurate to ensure no significant variances to actual revenue generated.

(f) The HHS will routinely revise and update the estimate to ensure alignment between the service agreement and Queensland Treasury's reporting system (TRIDATA).

(g) Budget adjustments for changes in own source revenue from private patients will be actioned through the processes set out in Schedule 5 of this service agreement.

7. Funding sources

7.1 The four main funding sources contributing to the HHS service agreement value are:

(a) Commonwealth funding;

(b) State funding;

(c) grants and contributions; and

(d) own source revenue.

7.2 Table 5 provides a summary of the funding sources for the HHS and the total value of the service agreement.

7.3 The HHS must undertake regular quality audits to check for potential duplicates in funding source, in particular the National Health Reform Agreement and Medicare given the Commonwealth's contribution to both funding sources. The HHS should take active steps to remedy areas of concern. A consumer's choice of funding arrangement should be reflected on a patient election form.

8. Funds disbursement

8.1 The Department agrees to pay the HHS the amount described in Table 5 of Schedule 2, subject to:

(a) parliamentary appropriation and adequate funds being allocated to the Department; and

(b) the terms of this service agreement.

8.2 All payments under this service agreement will be made in accordance with the requirements of the *National Health Reform Agreement* and the Act.

8.3 The Chief Executive will direct the disbursement of both State and Commonwealth funding from the State Pool Account, the State Managed Fund, and the Department of Health Expenditure account to the HHS.

8.4 However, the State (represented by the Chief Executive) will not:

(a) redirect Commonwealth payments between HHSs;

(b) redirect Commonwealth payments between funding streams (e.g., from ABF to block funding); and/or

(c) adjust the payment calculations underpinning the Commonwealth's funding.

8.5 The Department will pay state-funded activity-based funding, block funding and system manager funding to the HHS on a fortnightly basis in line with receipt of the State Appropriation Payment. The payment made will be equal to 1/26th of the state-funded component of the service agreement value described in Table 5.

8.6 The Department will pay Commonwealth-funded activity-based funding and block funding monthly in line with receipt of the Commonwealth payment. The payment made will be equal to 1/12th of the Commonwealth funded component of the service agreement value described in Table 5.

8.7 Where the parties have agreed to amend the service agreement value or the Commonwealth contribution amount changes, the fortnightly and/or monthly payments will be adjusted to reflect the amended service agreement value.

8.8 Service agreement payments may be made outside of these timeframes with the approval of the Department's Chief Finance Officer.

Table 4 HHS Funding by Outcomes Framework Care Domain 2024/25

Care Domain	Funding $	QWAU (Q27)
Prevention, early intervention, and primary health care	$156,960,559	10,479
Trauma and illness	$373,451,169	58,281
Mental health and alcohol and other drugs	$147,174,810	18,214
Cancer	$101,963,333	15,343
Planned care	$199,899,666	31,813
Maternity and neonates	$75,624,452	12,125
Chronic and complex	$323,479,742	49,009
Statewide services	$5,421,728	228
Depreciation	$85,113,000	0
**TOTAL**	**$1,469,088,459**	**195,492**

Table 5 HHS Total Funding Allocation by Funding Source 2024/25

Funding Source	24-25 NWAU (N2425)	24-25 QWAU (Q27)	24-25 Agreed ($)
**NHRA Funding**			
**ABF Pool**			
**ABF Funding (In scope NHRA)[2]**			
Commonwealth	155,061		$384,528,938
State		155,816	$502,070,292
State Specified Grants			$28,495,842
State-wide Services			$0
Restoring Planned Care	2,367	2,043	$12,520,893
Long Stay Patient Recovery Funding	1,312	1,315	$9,297,775
**Total ABF Funding (in scope NHRA)**	**158,740**	**159,173**	**$936,913,740**
**State Managed Fund**			
**Block Funding (State and Commonwealth)**			
Small rural hospital		10,731	$67,970,457
Teaching, Training and Research			$28,941,047
Other Mental Health	6,004	6,004	$70,878,765
Non-Admitted Home Ventilation			$0
Residential Mental Health Services		2,621	$7,922,404
Other Non-admitted Service			$0
Highly Specialised Therapies			$89,170
Other Public Hospital Programs			$0
Total NHRA Funding	158,740	178,529	$1,112,715,583
**Out of Scope NHRA**			
**Queensland ABF Model**			
DVA		1,199	$7,048,647
NIISQ/MAIC		470	$2,763,956
Oral Health		2,299	$15,702,692
Oral Health – FFA		0	$0
BreastScreen		632	$4,707,008
Child Health Checks		62	$1,149,024
**Total Queensland ABF Funding**		**4,662**	**$31,371,327**
**Discretely Funded Programs[3]**			
Department of Health			$116,814,834
Locally Receipted Funds			$9,951,108
Research (Other OSR)			$0
**Total Discretely Funded Programs**			**$126,765,942**

[2] The split between Commonwealth and State NHRA funding will change during the year as the purchased activity targets are updated and the National Health Funding Board updates their payment advice accordingly.

[3] Includes all other (non-ABF) State and Commonwealth funded health services including, but not limited to, Prisoner Health, Public Health and Prevention Services.

Funding Source	24-25 NWAU (N2425)	24-25 QWAU (Q27)	24-25 Agreed ($)
**Own Source Revenue**			
Private Patient Admitted Revenue[4]	2,626	2,589	$15,221,064
Pharmaceuticals Benefits Scheme		4,263	$56,677,674
Non-Admitted Services		4,544	$8,265,587
Other Activities[5]		890	$25,663,087
Oral Health – CDBS		0	$625,000
**Total Own Source Revenue**	**-**	**12,286**	**$106,452,412**
Locally Receipted Funds (exc. Discretely Funded Programs)[6]			$6,670,195
Depreciation			$85,113,000
**GRAND TOTAL**	**158,740**	**195,477**	**$1,469,088,459**

**Pool Accounts**			
ABF Pool (National Health Funding Pool)[7]			$968,285,067
State Managed Fund[8]			$175,801,843
System Manager			$116,814,834

[4] The estimated value of the revenue earned from private patients, based on OSR estimates provided by the HHS.

[5] Incorporates all OSR which is not identified elsewhere in Table 5.

[6] Includes items such as training programs and donations. Does not include locally receipted funds associated with discretely funded programs, e.g.Transition Care.

[7] Articulates the financial payment made to support in-scope ABF services under the NHRA including DVA, NIISQ, MAIC and BreastScreen Services. Applies to all HHSs except Central West HHS and Torres and Cape HHS.

[8] Articulates the payment made for block funded services under the NHRA, DVA, NIISQ and MAIC services.

Table 6 National Health Reform Funding

NHRA Funding Type	No. of In-scope services (NWAU)	No. of Out-of-scope services (QWAU)	Total Services (WAU)	ABF NEP ($)	State Price ($)	Funding for In-scope services at NEP ($)	Cwlth contribution for In-scope services ($)	State contribution for In-scope services ($)	State contribution for Out-of-scope services ($)	Total Cwlth and State contribution ($)
National Efficient Price (NEP)		*a,b*		*c*	*d*			*e*		
**ABF Allocation (NWAU)**										
Emergency Department	23,037	411	23,448	$6,465	$5,878	148,934,727	57,128,630	78,825,060	2,736,097	138,689,787
Acute Admitted	93,324	2,164	95,488	$6,465	$5,878	603,339,796	231,430,082	319,323,078	14,397,204	565,150,363
Admitted Mental Health	6,646	31	6,677	$6,465	$5,878	42,965,376	16,480,730	22,739,816	204,587	39,425,134
Sub-Acute	10,057	1,585	11,642	$6,465	$5,878	65,015,556	24,938,775	34,410,075	10,547,987	69,896,837
Non-Admitted	21,998	3,803	25,801	$6,465	$5,878	142,214,098	54,550,720	75,268,105	25,304,120	155,122,945
**Total ABF Allocation**	**155,061**	**7,994**	**163,055**			**1,002,469,553**	**384,528,938**	**530,566,134**	**53,189,995**	**968,285,067**
**Block Allocation**										
Teaching, Training, and Research						0	7,690,046	21,251,001	0	28,941,047
Small and Rural Hospitals[9]						0	19,528,104	48,442,352	0	67,970,457
Other Mental Health						0	29,371,638	49,429,532	0	78,801,169

[9] Incorporating small regional and rural public hospitals, four specialist mental health facilities (Baillie Henderson Hospital, Jacaranda Place – Queensland Adolescent Extended Treatment Centre, The Park – Centre for Mental Health and Kirwan Rehabilitation Unit) and the Ellen Barron Family Centre.

NHRA Funding Type	No. of In-scope services (NWAU)	No. of Out-of-scope services (QWAU)	Total Services (WAU)	ABF NEP ($)	State Price ($)	Funding for In-scope services at NEP ($)	Cwlth contribution for In-scope services ($)	State contribution for In-scope services ($)	State contribution for Out-of-scope services ($)	Total Cwlth and State contribution ($)
Non-Admitted Home Ventilation						0	0	0	0	0
Other Non-Admitted Services						0	0	0	0	0
Other Public Hospital Programs						0	0	0	0	0
Highly Specialised Therapies						0	77,224	11,946	0	89,170
**Total Block Allocation**						**0**	**56,667,012**	**119,134,831**	**0**	**175,801,843**
**Grand Total Funding Allocation**										**1,144,086,910**

Notes

a. QWAU refers to Queensland Weighted Activity Units in Q27 phase (built on N2425)
b. DVA, NIISQ/MAIC, Oral Health, Child Health Checks and BreastScreen
c. Queensland Efficient Price used to Purchase growth QWAUs
d. NWAU x NEP
e. State funding transacted through the Pool/State Managed Fund Account; not covered under the NHRA
- NWAU estimates do not take account of cross-border activity.

Table 7 Discretely Funded Programs (Non-ABF)

Discretely Funded Programs	Revenue Models	$
Aged Care Assessment Program	Commonwealth	$1,359,026
Alcohol, Tobacco and Other Drugs	State	$6,151,356
Community Health Programs	State	$37,078,933
Interstate Patients (QLD residents)	State	$4,468,800
Other State Funding	State	$9,088,129
Patient Transport: PTSS	State	$9,975,775
Patient Transport: Aeromedical Retrieval	State	$4,971,226
Patient Transport	State	$0
Prevention Services and Public Health	Commonwealth	$23,333,500
	State	$58,437
Prisoner Primary Health Services	State	$11,946,655
	Capitation	$992,306
Torres Strait Treaty	Commonwealth	$0
Multi-purpose Health Services	Commonwealth	$4,314,790
Residential Aged Care Services	Commonwealth	$293,925
	Locally Receipted Funds	$0
	State	$1,650,766
Transition Care	Locally Receipted Funds	$3,739,294
	State	$1,128,848
Research	Commonwealth	$2,361
	OSR	$0
Home and Community Care (HACC) Program	Locally Receipted Funds	$6,211,814
**Discretely Funded Programs Total**		**$126,765,942**
**TOTAL**		**$126,765,942**

Schedule 3 Department of Health Provided Services

1. In scope services and service schedules

Table 8 Department of Health provided services and service schedules

Provider	Service provided	Link to Service Statement
Corporate Services Division (CSD)	• Corporate Enterprise Solutions<br>• Finance Branch:<br>o Accounts Payable Service Provision<br>o Banking and Payment Services<br>• Central Pharmacy<br>• Group Linen Services<br>• Transport and Logistic Services<br>• Supply Chain Services	CSD Service Schedules
eHealth Queensland (eHQ)	• ICT Service	eHQ Service Schedule
Queensland Public Health and Scientific Services Division (QPHaSS)	• Pathology Queensland<br>• Biomedical Technical Services<br>• Public Health Services	QPHaSS Service Schedules

Schedule 4 Performance Measures

1. Performance Measures

1.1 The performance of the HHS will be measured according to the assessment criteria and processes described in the Performance and Accountability Framework.

1.2 Existing performance indicators are mapped to the care domains of the Outcomes Framework.

1.3 The detailed specification for each of the performance measures listed in this service agreement are provided through performance measure attribute sheets.

1.4 The performance measures identified in this service agreement are applicable to the HHS unless otherwise specified within the attribute sheet.

1.5 HHSs are also required to report against the agreed Statewide Health Equity Key Performance Measures (Table 12).

Table 9 HHS Performance Measures – Key Performance Indicators

Outcomes Framework Care Domain	Key Performance Indicators	Indicator Number
Chronic and complex	Hospital Acquired Complications (IHACPA code 8, 11, 13, 14)	31
Chronic and complex	Potentially Preventable Hospitalisations – First Nations Peoples:<br>• Diabetes complications<br>• Selected conditions	 37a<br>37b
Chronic and complex	Potentially avoidable deaths – First Nations Peoples	70
Maternity and neonates	Hospital Acquired Complications (IHACPA code 15,16)	31
Maternity and neonates	Proportion of healthy birthweight babies (2,500 grams to 4,499 grams at birth) – Aboriginal and Torres Strait Islander babies	71
Mental health, alcohol, and other drugs	Face to face community follow up within 1-7 days of discharge from an acute mental health inpatient unit	26
Mental health, alcohol, and other drugs	Proportion of mental health and alcohol and other drug service episodes with a documented care plan	27
Mental health, alcohol, and other drugs	Suicide count and rate – First Nations Peoples	72
Other	Average sustainable Queensland Health FTE	50
Other	Capital expenditure performance	51
Other	Forecast operating position:<br>• Full year<br>• Year to date	 48<br>49
Planned care	Category 1 elective surgery patients treated within the clinically recommended timeframe	7
Planned care	Elective surgery patients waiting longer than the clinically recommended timeframe	9
Planned care	Proportion of overnight inpatients discharged by 10am	12

Outcomes Framework Care Domain	Key Performance Indicators	Indicator Number
Planned care	Category 4 gastrointestinal endoscopy patients treated within the clinically recommended timeframe	13
Planned care	Gastrointestinal endoscopy patients waiting longer than the clinically recommended timeframe	16
Planned care	Category 1 patients who receive their initial specialist outpatient appointment within the clinically recommended timeframe	17
Planned care	Patients waiting longer than clinically recommended for their initial specialist outpatient appointment	19
Planned care	Telehealth utilisation rates:<br>Number of non-admitted telehealth service events	20
Planned care	Hospital Acquired Complications (IHACPA code 1,2,3,4,6,7,9,10,12)	31
Planned care	Missed Opportunity to Treat – Outpatients	73
Prevention, early intervention, and primary health care	Access to oral health services (adults)	21
Prevention, early intervention, and primary health care	Access to oral health services (children)	67
Prevention, early intervention, and primary health care	Potentially avoidable deaths – First Nations Peoples	70
Prevention, early intervention, and primary health care	Suicide count and rate – First Nations Peoples	72
Trauma and illness	Hospital Access Target (Admitted Patients)<br>• % of emergency stays within 4 hours	1
Trauma and illness	Hospital Access Target (All Patients)<br>• % of emergency stays within 4 hours	3
Trauma and illness	Emergency Department wait time by triage category	4
Trauma and illness	Emergency Department stays greater than 24 hours	5
Trauma and illness	Patient off stretcher time	6
Trauma and illness	Lost Minutes	61
Trauma and illness	Emergency Surgery patients treated in hours	62
Trauma and illness	Emergency Surgery patients treated in time	63
Trauma and illness	Transfer of care	69

Table 10 HHS Performance Measures - Safety and Quality Markers

Outcomes Framework Care Domain	Safety and Quality Markers	Indicator Number
Maternity and neonates	Sentinel Events	32
Planned care	Sentinel Events	32

Outcomes Framework Care Domain	Safety and Quality Markers	Indicator Number
Planned care	Hospital Standardised Mortality Ratio	33
Planned care	Severity Assessment Code (SAC1) analysis completion rates	34
Planned care	Healthcare-associated Staphylococcus Aureus (including MRSA) bacteraemia	35
Planned care	Patient Reported Experience	68

Table 11 HHS Performance Measures – Outcome Indicators

Outcomes Framework Care Domain	Outcome Indicators	Indicator Number
Chronic and complex	Potentially Preventable Hospitalisations (diabetes complications)	38
Chronic and complex	Potentially Preventable Hospitalisations (non-diabetes complications)	39
Chronic and complex	Advance care planning	43
Chronic and complex	Cardiac rehabilitation	44
Maternity and neonates	% of low birthweight babies born to Queensland mothers	41
Mental health, alcohol, and other drugs	Rate of seclusion events	28
Mental health, alcohol, and other drugs	Rate of absent without approval from acute mental health inpatient care	29
Mental health, alcohol, and other drugs	Smoking cessation clinical pathway	42
Other	First Nations peoples' representation in the workforce	47
Planned care	Complaints resolved within 35 calendar days	36
Planned care	Smoking cessation clinical pathway	42
Prevention, early intervention, and primary health care	General oral health care for First Nations peoples	22
Prevention, early intervention, and primary health care	The percentage of oral health activity which is preventive	23
Prevention, early intervention, and primary health care	Access to emergency dental care	24
Prevention, early intervention, and primary health care	Smoking cessation clinical pathway	42
Prevention, early intervention, and primary health care	Adolescent vaccinations administered via the statewide School Immunisation Program	45

Table 12 Statewide Health Equity Key Performance Measures

Outcomes Framework Care Domain	Key Performance Measures	Indicator Number
Chronic and complex	Advance care planning	43
Chronic and complex	Integrated care pathways - Rural and Remote HHSs:<br>• Care pathway in place for patients with identified co-morbidities	60
Maternity and neonates	Proportion of healthy birthweight babies (2,500 grams to 4,499 grams at birth) – Aboriginal and Torres Strait Islander babies	71
Mental health, alcohol, and other drugs	Face to face community follow up within 1-7 days of discharge from an acute mental health inpatient unit	26
Mental health, alcohol, and other drugs<br>Chronic and complex	Suicide count and rate – First Nations People	72
Other	First Nations peoples' representation in the workforce	47
Planned care	Category 1 elective surgery patients treated within the clinically recommended timeframe	7
Planned care	Category 2 and 3 elective surgery patients treated within the clinically recommended timeframe	8
Planned care	Category 1 patients who receive their initial specialist outpatient appointment within the clinically recommended timeframe	17
Planned care	Category 2 and 3 patients who receive their initial specialist outpatient appointment within the clinically recommended timeframe	18
Prevention, early intervention, and primary health care	General oral health care for First Nations peoples	22
Prevention, early intervention, and primary health care	Potentially avoidable deaths – First Nations peoples	70

Schedule 5 Amendments to this service agreement

1. Agreed process to amend this service agreement

1.1 The parties acknowledge that this service agreement is subject to amendment, which will generally occur through:

(a) amendment windows;

(b) extraordinary amendment;

(c) periodic adjustments; and

(d) end of financial year reconciliation.

1.2 **Amendment windows**

(a) There will be set periods of time nominated by the Department during the year (amendment windows) in which a party may propose an amendment and the parties will endeavour to negotiate and finalise proposals to amend this service agreement.

(b) Amendment proposals that are agreed will be documented in a deed of amendment to this service agreement.

(c) Further details on the amendment window process, including the timing of amendment windows, is provided online as detailed in Appendix 1.

1.3 **Extraordinary Amendment**

(a) Outside an amendment window, the Department and the HHS agree to limit any proposal to amend the terms of this service agreement to those where there is an urgent priority need to facilitate a funding allocation (extraordinary amendment). The parties will endeavour to negotiate and finalise any such proposal urgently.

(b) The process for submitting, negotiating, and resolving an extraordinary amendment is available online as detailed at Appendix 1.

(c) Agreed extraordinary amendments will be reflected in a notice issued by the Chief Executive and countersigned as accepted by the HHS. The notice will be replaced when the extraordinary amendment is subsequently formalised in a deed of amendment issued following the next amendment window.

1.4 **Periodic adjustments**

(a) The service agreement value (and corresponding purchased activity) may be adjusted at any time to reflect funding variations that:

(i) occur on a periodic basis or in line with adjustments permitted for specific funding allocations;

(ii) are referenced in the service agreement; and

(iii) are based on a clearly articulated formula, an agreed basis or such other reasonably substantiated basis tied to performance.

(b) Periodic adjustments will be reflected in an adjustment notice issued by the Chief Executive (or delegate) to the HHS, based on relevant data, and subsequently formalised in a deed of amendment issued following the next amendment window.

1.5 **End of financial year reconciliation**

(a) There will be an end of financial year reconciliation process, with the scope defined by the Department and informed by Queensland Government Central Agency requirements.

(b) The Department will provide the HHS with a reconciliation of all service agreement funding and purchased activity for the prior financial year. This will reflect the position following conclusion of the end of financial year adjustments process.

(c) The impact of end of financial year adjustments on subsequent year funding and activity will be incorporated in the service agreement through the deed of amendment executed following the next available amendment window.

(d) This clause will survive expiration of this service agreement.

Appendix 1 Reference Documents

Service Agreement:

- Data supply requirements
- Delivery of Purchased Activity – Requirement for Quality Audits specification sheet
- Dispute resolution process - current
- First Nations First Strategy 2032
- Funding Outcomes Framework
- Hospital and Health Boards Act 2011
- Joint Regional Needs Assessment Framework
- Making Tracks Together: Queensland's Aboriginal and Torres Strait Islander Health Equity Framework
- Making Tracks toward closing the gap in health outcomes for Indigenous Queenslanders by 2033 - Policy and Accountability Framework
- National Agreement on Closing the Gap
- National Health Reform Agreement (NHRA) 2020-25
- Performance Measures Attribute Sheets
- Public Health Practice Manual
- Queensland Government Building Policy Framework and Guideline
- Queensland Health Performance and Accountability Framework
- Service agreement amendment processes
- Specifications supporting the Healthcare Purchasing Model
- Statewide services reference material

Supporting Policy documents

- Aboriginal and Torres Strait Islander Health Workforce Strategic Framework 2016-2026
- Department of Health Strategic Plan 2021-2025

- HEALTHQ32: A vision for Queensland's health system
- My health, Queensland's future: Advancing health 2026
- Queensland Health Equity, Diversity, and Inclusion Statement of Commitment
- System Outlook to 2026 - for a sustainable health service

Contracting and Performance Management Branch
GPO Box 48
Brisbane QLD 4001 Australia

Tel (+61) (07) 3708 5869

www.health.qld.gov.au

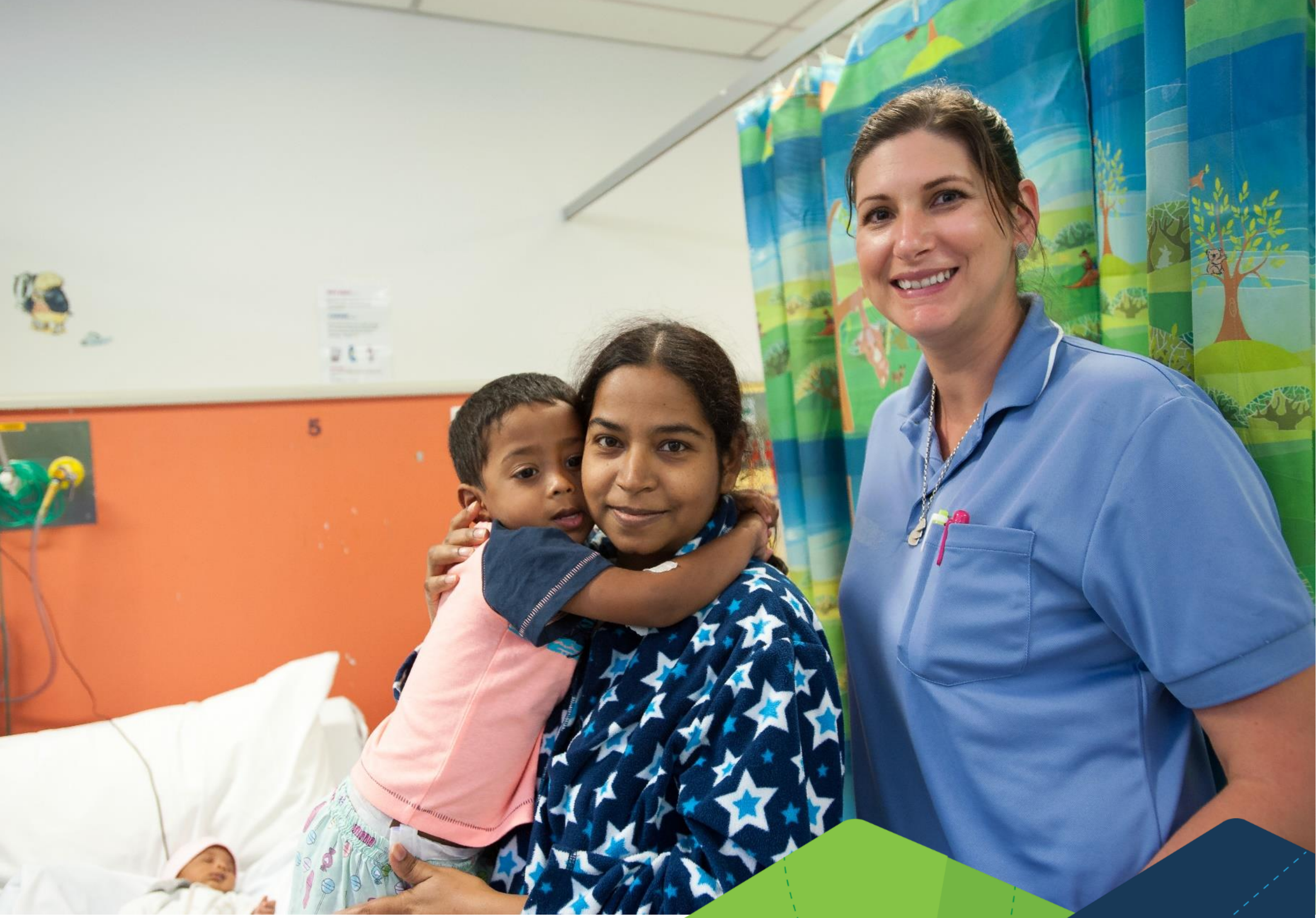

Queensland Health

Service Agreement 2022/23 – 2024/25

Cairns and Hinterland Hospital and Health Service

Queensland Government

Cairns and Hinterland Hospital and Health Service, Service Agreement 2022/23 - 2024/25

Published by the State of Queensland, (Queensland Health), July 2022

This document is licensed under a Creative Commons Attribution 3.0 Australia licence. To view a copy of this licence, visit creativecommons.org/licenses/by/3.0/au

© State of Queensland (Queensland Health) **2022**

You are free to copy, communicate and adapt the work, as long as you attribute the State of Queensland (Queensland Health).

For more information contact:

Contracting and Performance Management Branch, Department of Health, GPO Box 48, Brisbane QLD 4001
Tel (+61) (07) 3708 5869

Email SAM@health.qld.gov.au

An electronic version of this document is available at www.health.qld.gov.au/system-governance/health-system/managing/default.asp

Disclaimer:
The content presented in this publication is distributed by the Queensland Government as an information source only. The State of Queensland makes no statements, representations or warranties about the accuracy, completeness or reliability of any information contained in this publication. The State of Queensland disclaims all responsibility and all liability (including without limitation for liability in negligence) for all expenses, losses, damages and costs you might incur as a result of the information being inaccurate or incomplete in any way, and for any reason reliance was placed on such information.

Acknowledgement

We acknowledge the Traditional and Cultural Custodians of the lands, waters and seas across Queensland, pay our respects to Elders past and present, and recognise the role of current and emerging leaders in shaping a better health system.

We acknowledge the First Nations people in Queensland are both Aboriginal Peoples and Torres Strait Islander Peoples, and support the cultural knowledge, determination and commitment of Aboriginal and Torres Strait Islander communities in caring for health and wellbeing for millennia.

We recognise the ancestral lands of the many traditional and cultural custodians, which comprises the Cairns and Hinterland geographical footprint, where we work to provide safe and quality health services.

From the lands of the Kuku Yalanji in the north (Mossman), Tagalaka in the west (Croydon) and Girramay in the south (Tully) and all other tribes and clans therein, including the diaspora of Torres Strait Islanders and other first peoples who made this region their home, we offer our deep respect.

Cairns and Hinterland Hospital and Health Service is proud to recognise and celebrate the cultural diversity of our communities and workforce at the following locations:

Location	Traditional Custodians
Atherton Hospital	Tableland Yidinji
Babinda Multipurpose Health Service	Wanyurr Majay
Cairns Hospital	Gimuy Walaburra Yidinji & Yirrganydji
Cow Bay	Kuku Yalanji
Chillagoe Hospital	Wakaman
Croydon Primary Health Care	Tgalaka
Dimbulah Primary Health Care	Bar Barrum
Forsayth and Georgetown Primary Health Care	Ewamian
Gordonvale Hospital	Malanbarra Yidinji
Gurriny Yealamucka Health Service	Gunggandji & Mandingalbay Yidinji
Herberton Hospital	Jirrabal
Innisfail Hospital	Mamu
Jumbun Primary Health Care	Girramay
Kuranda Medical Service	Djabugay
Malanda Primary Health Care	Ngadjon Jii
Mareeba Hospital	Muluridji
Millaa Millaa Primary Health Care	Mamu
Mission Beach Community Health Centre	Djiru Warrangburra
Mossman Multipurpose Health Service	Kuku Yalanji
Mount Garnet Primary Health Care	Bar Barrum
Ravenshoe Primary Health Care	Jirrabal
Tully Hospital	Gulnay

Contents

Acknowledgement ... 3
Contents ... 4
Tables ... 5
1. Introduction ... 6
2. Scope ... 6
3. Services ... 6
4. Performance and Accountability Framework ... 7
5. Data supply requirements ... 7
6. Hospital and Health Service accountabilities ... 8
7. Department accountabilities ... 9
8. Achieving health equity with First Nations Queenslanders ... 9
9. General ... 10
10. Counterparts ... 11
Execution ... 12
Schedule 1 HHS profile ... 13
1. HHS profile ... 13
Schedule 2 Funding and purchased activity and services ... 14
1. Introduction ... 14
2. Purchased health services ... 15
3. Teaching training and research ... 17
4. Delivery of purchased activity ... 18
5. Delivery of purchased services ... 19
6. Financial adjustments ... 20
7. Funding sources ... 24
8. Funds disbursement ... 24
Schedule 3 Performance Measures ... 30
1. Performance Measures ... 30
Schedule 4 Amendments to this service agreement ... 32
1. Agreed process to amend this service agreement ... 32
Appendix 1 Reference Documents ... 34

Tables

Table 1 Statewide Services 15
Table 2 Financial adjustments applied on breach of activity thresholds 20
Table 3 Purchasing Incentives 2022/23 21
Table 4 Cairns and Hinterland HHS Total Funding Allocation by Funding Source 2022/23 26
Table 5 National Health Reform Funding 28
Table 6 Discretely Funded Programs (Non-ABF) 29
Table 7 HHS Performance Measures – Key Performance Indicators 30
Table 8 HHS Performance Measures - Safety and Quality Markers 31
Table 9 HHS Performance Measures – Outcome Indicators 31

1. Introduction

1.1 In performing this service agreement, the Department and the Hospital and Health Service (HHS) will act consistently with the object of the *Hospital and Health Boards Act 2011* (Qld) (Act).

1.2 The Department and the HHS operate as part of a networked system and agree to work collaboratively with each other, with other HHSs and with the Queensland Ambulance Service in the best interests of the Queensland public sector health system.

1.3 The parties will ensure that planning and delivery of health services is consistent with the strategies and priorities set out by government. The parties recognise the importance of the HHS' Health Equity Strategy (as defined in the Act) and the parties commitment to improving health and wellbeing outcomes and achieving health equity for Aboriginal and Torres Strait Islander peoples.

2. Scope

2.1 This service agreement covers the period from 1 July 2022 to 30 June 2025.

2.2 The parties have identified the services to be provided by the HHS, the funding for the provision of those services, the performance measures applicable to the services and data requirements.

3. Services

3.1 In delivering services, the HHS is required to meet:

(a) the applicable conditions of all national agreements and national partnership agreements between the Queensland Government and the Commonwealth Government and commitments under any related implementation plans; and

(b) the applicable conditions of each agreement or arrangement for funding between the Department and the HHS and commitments under any related implementation plans, which conditions and commitments may be recorded separately to this service agreement but for which funding is (or becomes) provided in Schedule 2.

3.2 The HHS is required to deliver the services outlined in this service agreement for which funding is provided in Schedule 2.

3.3 Where issues arise which prevent the HHS from providing a service or necessitate a reduction in the level or scope of a service provided, prompt notification must be made to the Department and impacted HHSs, with appropriate details. The HHS must minimise any clinical risk or adverse impact to patient experience that may result from service disruption. The Department will respond to the HHS on any requirements it has concerning service delivery and any adjustments triggered by under delivery.

3.4 If the HHS wishes to terminate or reduce service levels for a service for which funding is provided in Schedule 2, this will remain subject to negotiation and agreement by the Department at its discretion.

3.5 For any new services proposed during the term of this service agreement, the parties agree:

(a) the service must meet a demonstrated clinical need and provide value for money;

(b) the commencement of a new service, including the implementation of new models of care, may occur where the service has been commissioned by the Department, a funding stream is in place and any conditions relating to the funding have already been agreed; and

(c) if the HHS wishes to commence providing a new service that has not been commissioned by the Department, this will remain subject to agreement by the Department at its discretion.

3.6 It is acknowledged that there may from time to time need to be service delivery changes between HHSs. Management of inter-HHS relationships should be informed by the following principles:

(a) HHSs should maintain the proportion of out of HHS work undertaken unless as a result of agreed repatriation of patients;

(b) each HHS should manage patients from its own catchment population if it is within its clinical capability to do so as specified by the Clinical Services Capability Framework; and

(c) where it is proposed that a service move from one HHS to another, the Department will consider, as part of its review under clause 3.4 and 3.5, whether the respective Health Service Chief Executives endorse the proposed change in patient flows; and the funding required to follow the patient.

4. Performance and Accountability Framework

4.1 The Queensland Health Performance and Accountability Framework (the Performance and Accountability Framework) sets out the framework within which the Department monitors and manages the performance of public sector health services in Queensland.

4.2 The parties will act consistently with the Performance and Accountability Framework.

5. Data supply requirements

5.1 The HHS will provide the Department with all clinical and non-clinical data that is reasonably required to support the effective management of the public sector health system. This will include, but is not limited to, data that is required to:

(a) fulfil legislative obligations;

(b) deliver accountabilities and obligations to State and Commonwealth Governments; including related to the provision and reconciliation of activity data by the Administrator of the National Health Funding Pool;

(c) monitor and support performance improvement;

(d) manage this service agreement;

(e) support clinical innovation; and

(f) facilitate evaluation and audit.

5.2 The parties agree and acknowledge that:

(a) the Department will keep the HHS informed of the Department's data requirements; and

(b) data will be provided as required, or permitted, by law.

6. Hospital and Health Service accountabilities

6.1 The HHS will perform its obligations under this service agreement.

6.2 As applicable to the HHS and its services, the HHS will comply with:

(a) legislation and subordinate legislation, including the Act;

(b) cabinet decisions;

(c) Ministerial directives;

(d) agreements entered into between the Queensland and Commonwealth governments (or agreements with others in furtherance of such agreements), of which it is informed;

(e) agreements entered into between the Department and other Queensland Government entities, of which it is informed;

(f) all industrial instruments;

(g) all health service directives and health employment directives; and

(h) all policies, guidelines and implementation standards, including human resource policies.

6.3 As part of the commitment to achieving First Nations health equity, the HHS will prioritise the elimination of racial discrimination and institutional racism within its service and ensure that Aboriginal and Torres Strait Islander peoples have access to culturally safe and responsive health services.

6.4 The HHS will ensure that effective health service planning and delivery systems are in place, working in collaboration with the Department.

6.5 The HHS will ensure that health service employees employed by the Chief Executive[1] who perform work for the HHS are managed in accordance with any applicable delegations and directions from the Chief Executive.

6.6 The HHS will ensure that effective asset management systems are in place, working in collaboration with the Department.

6.7 The HHS will maintain accreditation to the standards required by the Department.

6.8 The HHS will appropriately perform and fulfil its functions under the Act.

6.9 The HHS will provide to the Chief Executive reports of a type, and at the intervals, agreed between the parties, or as reasonably specified by the Chief Executive.

7. Department accountabilities

7.1 The Department will perform its obligations under this service agreement including, in return for the HHS performing its obligations and delivering the services, providing funding to the HHS as stipulated in this service agreement (as amended).

7.2 The Department will:

(a) comply with applicable legislation and subordinate legislation, including the Act, as relates to this service agreement; and

(b) perform the system manager role (as defined in the Act) through the Chief Executive;

7.3 The Chief Executive will appoint health service employees to perform work for the HHS for the purpose of enabling the HHS to perform its functions under the Act.

7.4 The Chief Executive will consult, cooperate, and coordinate with the HHS to ensure legal compliance with the *Work Health and Safety Act 2011* and other legislation as it applies to the scope, nature and location of operations associated with this service agreement.

7.5 The Chief Executive will appropriately perform and fulfil their functions under the Act.

8. Achieving health equity with First Nations Queenslanders

8.1 Through legislative amendments to the Act and the Hospital and Health Boards Regulation 2012, and the release of *Making Tracks Together: Queensland's Aboriginal and Torres Strait Islander Health Equity Framework* (2021), Queensland Health has strengthened its commitment to improving health and wellbeing outcomes and achieving health equity for First Nations peoples.

8.2 The HHS will develop a Health Equity Strategy to demonstrate the HHS's activities and key performance measures to achieve health equity with First Nations peoples that is compliant with legislative requirements. The Health Equity Strategy will act as the principal

[1] In this service agreement the term Chief Executive takes the meaning applied in the Act, which is the Chief Executive of Queensland Health, but which is generally referred to as the Director-General.

accountability mechanism between community and the HHS in achieving health equity for First Nations Queenslanders and forms a key part of the Queensland Government's commitment to the *National Agreement on Closing the Gap* (2020).

8.3 The HHS will review the Health Equity Strategy at least once every three years and will publish the Health Equity Strategy in a way that allows it to be accessed by members of the public.

8.4 The HHS will ensure that commitment and leadership is demonstrated through implementing the actions and achieving the key performance measures outlined in the Health Equity Strategy.

8.5 The HHS will report publicly on progress against the Health Equity Strategy.

8.6 The HHS will support the implementation of other supplementary policies and strategies to drive health equity across the public health system, including relevant election commitments.

8.7 The HHS will participate as a partner in the design, development and implementation of the new *Queensland First Nations Health Workforce Strategy for Action.*

9. General

9.1 Sub-contracting

(a) The HHS must have appropriate systems in place to ensure that any subcontractor is accredited (as applicable), qualified, and otherwise fit to perform any services for which it is contracted.

(b) The HHS must ensure that any sub-contractor who has access to confidential information (as defined in the Act) or personal information (as defined in the *Information Privacy Act 2009* (Qld)) complies with obligations no less onerous than those imposed on the HHS.

9.2 Insurance

The HHS must:

(a) hold and maintain the types and levels of insurances that the HHS considers appropriate according to its functions and obligations; and

(b) comply with reasonable requests or directions from the Department in this regard.

9.3 Amendment

The process for amending this service agreement is provided for under the Act and further outlined in Schedule 4.

10. Counterparts

10.1 This service agreement may be executed in two or more identical copy counterparts, each of which together will be deemed an original, but all of which together will constitute one and the same instrument.

10.2 In the event that any signature executing this service agreement or any part of this service agreement is delivered by facsimile transmission or by scanned e-mail delivery of a '.pdf' format data file or equivalent of the entire agreement, the signature will create a valid and binding obligation of the party executing (or on whose behalf the signature is executed) with the same force and effect as if the signature page were an original. For the avoidance of doubt, this service agreement may be in the form of an electronic document and may be electronically signed.

10.3 For execution under this clause 10 to be valid the entire service agreement upon execution by each individual party must be delivered to the remaining parties.

Execution

Executed as an agreement in Queensland

Signed by the Chief Executive, Queensland Health:)
)

....

Signature of Chief Executive

SHAUN DRUMMOND

....

Name of Chief Executive (print)

....

(date)

Signed for and on behalf of the Cairns and Hinterland Hospital and Health Service:)
)

....

Signature of Hospital and Health Board Chair

Clive Skarott

....

Name of Hospital and Health Board Chair (print)

17/6/2022

....

(date)

Execution

Executed as an agreement in Queensland

Signed by the Chief Executive, Queensland)
Health:)

....

Signature of Chief Executive

SHAUN DRUMMOND

....

Name of Chief Executive (print)

29 June 2022

....

(date)

Signed for and on behalf of the Cairns and)
Hinterland Hospital and Health Service:)

....

Signature of Hospital and Health Board Chair

....

Name of Hospital and Health Board Chair (print)

....

(date)

Schedule 1 HHS profile

1. HHS profile

This Schedule does not apply to this HHS.

Schedule 2 Funding and purchased activity and services

This Schedule 2 sets out:

(a) the services which are to be provided by the HHS;

(b) the activity purchased by the Department from the HHS;

(c) the funding provided for delivery of the purchased activity;

(d) the criteria and processes for financial adjustments associated with the delivery of purchased activity and specific funding allocations;

(e) the sources of funding that this service agreement is based on and the manner in which these funds will be provided to the HHS

1. Introduction

1.1 The HHS will deliver the services for which funding is provided in this Schedule 2. In providing these services, the HHS will ensure that:

(a) all statewide and national policy frameworks, guidelines, protocols and implementation standards applicable to the service provided are followed;

(b) participation in national programs is facilitated and supported, including where these programs are provided by the Commonwealth Government;

(c) service delivery partnerships, including with other HHSs, primary care organisations and non-government organisations, are maintained and operate effectively;

(d) collaboration and engagement with other service providers and stakeholders is initiated and maintained to ensure that an integrated system of treatment, care and support is in place and to facilitate the delivery of comprehensive and effective services. This may include but is not limited to:

(i) other HHSs;

(ii) non-government organisations;

(iii) Aboriginal and Torres Strait Islander community-controlled health organisations;

(iv) Queensland Ambulance Service;

(v) services provided through the Department of Health (for example, Pathology Queensland);

(vi) primary care providers;

(vii) other government departments and agencies; and

(viii) private providers;

(e) models of care and service delivery arrangements are consistent with evidence-based practice and offer value for money;

(f) services are provided on an equitable basis to the community, and processes are in place to ensure that services reach identified target populations, high risk groups and hard to reach communities;

(g) referral networks and pathways continue to operate effectively; and

(h) innovation and continuous improvement is supported.

2. Purchased health services

2.1 Table 4, Table 5 and Table 6 outline the activity and service streams which the Department agrees to purchase from the HHS pursuant to this service agreement.

2.2 More generally, this will include the following:

2.3 **Statewide Services**

(a) The designation of a service as a statewide service (either a clinical statewide service or a clinical support statewide service) will be determined by the Department, consistent with the stipulated governance arrangements for such services.

(b) The HHS will:

(i) collaborate with the Department and other HHSs in the implementation of, and adherence to, the governance and oversight arrangements for statewide services;

(ii) participate in, and contribute to, the staged review of the purchasing model for identified statewide services; and

(iii) ensure that referral pathways in and out of each statewide service are followed.

2.4 **Clinical Statewide Services and Clinical Support Statewide Services provided**

This clause does not apply to this HHS.

Table 1 Statewide Services

This table does not apply to this HHS.

2.5 **Regional services**

The HHS has responsibility for the provision and/or coordination of the following regional services:

(a) Basic physician training pathway;

(b) Community Forensic Outreach Service;

(c) Court liaison services;

(d) Court liaison services for young people;

(e) Forensic adolescent mental health services;

(f) Mental health clinical cluster support program;

(g) Mental Health Act delegate; and

(h) Mental health clinical indicator team program.

2.6 Prevention services and population health services

(a) The HHS will provide a range of services with a focus on the prevention of ill-health and disease, including:

  (i) Specialist Public Health Units;

  (ii) preventive health services;

  (iii) immunisation services;

  (iv) sexually transmissible infections including HIV and viral hepatitis;

  (v) tuberculosis services; and

  (vi) population health screening including, but not limited to, cancer screening services and newborn blood spot screening.

(b) Services will be provided in line with public health related legislation and the service delivery and reporting requirements outlined in the *Public Health Practice Manual*, as these relate to the services provided.

2.7 Aboriginal and Torres Strait Islander health services

The HHS will provide Aboriginal and Torres Strait Islander specific health services and initiatives consistent with the principles and objectives of the Queensland Government's *Making Tracks towards closing the gap in health outcomes for Indigenous Queenslanders by 2022 – Policy and Accountability Framework*. These service and initiatives will be delivered in line with guidance from the Aboriginal and Torres Strait Islander Health Division.

2.8 Mental health alcohol and other drugs services

The HHS will provide treatment, care and support for individuals who are, or may be, experiencing substance use disorders and/or other mental health disorders, mental health crisis and suicidal distress, and their families and carers. Services will be delivered in line with guidance from the Mental Health Alcohol and Other Drugs Branch:

2.9 Oral health services

The HHS will provide oral health services to people who meet the eligibility criteria for accessing public dental services in Queensland. Services will be delivered in line with guidance from the Office of the Chief Dental Officer.

2.10 Prisoner health services

The HHS will provide services for prisoners consistent with the principles, responsibilities and requirements specified in the Memorandum of Understanding (Prisoner Health Services) between Queensland Health and Queensland Corrective Services.

2.11 Youth detention services

This clause does not apply to this HHS.

2.12 Refugee health

The HHS will operate a health service for refugees, special humanitarian entrants and asylum seekers.

2.13 **State-funded outreach services**

(a) Where state-funded outreach services are provided;

(i) funding for the service will remain part of the providing HHS's funding allocation; and

(ii) the activity must be recorded at the HHS where the outreach service is being provided

(b) Any changes to the provision of outreach services will follow the requirements set out in clause 3 of this service agreement.

3. Teaching training and research

The HHS will provide the teaching, training and research programs for which funding is provided within Schedule 2 and as described below.

3.1 **Clinical education and training**

(a) The HHS will provide education and training placements for the following professional groups consistent with and proportionate to the capacity of the HHS and will support and align with stipulated placement terms governing clinical placements in Queensland Health facilities;

(i) medical students;

(ii) nursing and midwifery students;

(iii) pre-entry clinical allied health students;

(iv) interns;

(v) rural generalist trainees;

(vi) vocational medical trainees;

(vii) first year nurses and midwives;

(viii) re-entry to professional register nursing and midwifery candidates;

(ix) dental students;

(x) allied health rural generalist training positions;

(xi) Aboriginal and Torres Strait Islander Health Workers and Aboriginal and Torres Strait Islander Health Practitioners.

(b) The HHS will comply with the state-wide vocational medical training pathways.

(c) The HHS will support profession specific and inter-profession statewide allied health clinical education programs.

(d) The HHS will continue to implement and retain the following positions provided through clauses to the *Health Practitioners and Dental Officers (Queensland Health) Certified Agreement (No 3)*:

(i) health practitioner research positions provided through the Research Package for Health Practitioners; and

(ii) clinical educator positions provided through the Clinical Education

Management Initiative for Health Practitioners.

(e) The HHS will maintain or increase its contribution of staff to the Queensland Country Relieving doctors program and the receiving HHS will be responsible for wages, clinical governance and appropriate supervision of the junior medical relievers.

3.2 **Statewide training, education and research**

This clause does not apply to this HHS.

3.3 **Health and medical research**

The HHS will:

(a) develop and implement a strategy to drive increased research activity and its translation into clinical practice;

(b) support increased and equitable access to clinical trials for patients;

(c) support clinicians to undertake research linked to their practice; and

(d) ensure high quality and timely research governance approval processes.

4. Delivery of purchased activity

4.1 The Department and the HHS will monitor actual activity against purchased levels and will take action as necessary to ensure delivery of purchased levels is achieved. The HHS has a responsibility to only recognise revenue that is linked with actual activity delivered against purchased volumes.

4.2 The HHS will actively monitor variances from purchased activity levels and notify the Department as soon as the HHS becomes aware of significant variances.

4.3 The HHS will also notify the Department of deliberate changes to the consistent recording of activity within year that would result in additional activity being recorded for existing health services.

4.4 If the HHS wishes to convert activity between purchased activity types, programs and levels the HHS must negotiate this with the Department based on a sound needs based rationale.

4.5 The Department will reconcile in-scope activity, as defined in the Activity Reconciliation specification sheet (available online, as detailed in Appendix 1), delivered by the HHS against the purchased in-scope activity targets outlined in Table 4.

4.6 Activity reconciliation will be undertaken in February (for the July to December period) and August (for the January to June period) each year and will be derived through application of the methodology which is documented in the Activity Reconciliation specification sheet.

4.7 Should the HHS be unable to deliver the activity that has been funded a financial adjustment will be applied.

4.8 Under delivery of in-scope activity, as defined in the Activity Reconciliation specification sheet, will be withdrawn from the HHS at 100% of the Queensland Efficient Price (QEP).

4.9 Funding and corresponding activity that is withdrawn from the HHS may be reallocated to an alternate provider that can undertake the activity.

4.10 Any resulting changes to funding and purchased activity levels will be actioned through the processes outlined in Schedules 2 and 4 of this service agreement.

4.11 The HHS will maintain its efforts to accurately record the delivered activity and submit this data to the Department in accordance with the agreed requirements.

5. Delivery of purchased services

5.1 As part of the service agreement, the Department purchases a range of services (or deliverables) from the HHS for the delivery of certain programs and projects.

5.2 These program or project services may be the subject of separately agreed conditions tied to that funding and the focus of detailed monitoring by the Department.

5.3 Conditions may include, but are not limited to:

(a) establishment and/or commencement of services;

(b) delivery of activity;

(c) workforce obligations;

(d) establishment of oversight committees;

(e) opening or upgrades to facilities;

(f) program evaluation;

(g) program management;

(h) reporting or notification obligations; and

(i) attainment of performance standards.

5.4 The HHS will ensure that the conditions are achieved within the stipulated time period.

5.5 The HHS will notify the Department if the HHS forecasts an inability to achieve program or project objectives or the conditions.

5.6 The Department may withdraw allocated funding pro rata to the level of under delivery if the services for a specified program or project are not being fully delivered according to the objectives or conditions.

5.7 Any resulting changes to funding and purchased activity levels will be actioned through the processes outlined in Schedules 2 and 4 of this service agreement.

6. Financial adjustments

6.1 Activity targets

(a) The Department will initiate a joint process with the HHS to determine whether a financial adjustment should be applied in relation to any purchased activity which has breached the thresholds identified bi-annually. This process will take into account any relevant matters that have been identified in a review/analysis of the breach as well as the outcomes of the activity plan implemented to address the activity breach.

(b) Activity will be monitored at the purchasing hierarchy level. Providing the HHS meets all relevant KPIs and specific funding allocations, the HHS has the ability to negotiate the transfer of activity across the purchasing hierarchy with the Department.

(c) Table 2 demonstrates the financial adjustment that will be applied when activity thresholds have been breached.

(d) The HHS may not utilise the provisions within AASB15 *Revenue from Contracts with Customers* to override the application of any financial adjustment made by the Department in line with Table 2.

Table 2 Financial adjustments applied on breach of activity thresholds

Example of Breach	Description	Financial Adjustment
Over performance	Activity exceeds that specified in the service agreement value for in-scope activity as shown in Table 4.	Purchasing contracts are capped and an HHS will not be paid for additional activity with the exception of activity that is in scope for the identified purchasing incentives as set out in Table 3.
Under performance	Activity is below that specified for in-scope activity as shown in Table 4.	Purchased activity and the related funding will be withdrawn at 100% of the Queensland Efficient Price and reallocated to an alternate provider that can undertake the activity.<br>Refer to Table 4 for the HHS QWAU target.
Failure to deliver on service commitments linked to specific funding allocations	Specific funding allocations<br>National Partnership Agreements.	It is at the discretion of the Department to withdraw allocated funding pro rata to the level of under delivery.
For all other types of activity variance, any financial adjustment will be made at the discretion of the Department.		

6.2 National Partnership on COVID-19 Response

(a) The Department will provide additional funding to the HHS under the *National Partnership on COVID-19 Response* where actual additional cost is demonstrated.

(b) Funding will be provided where the HHS has:

(i) undertaken activity that is in-scope for the State Public Health Payment, as defined under the *National Partnership on COVID-19 Response*, during the reporting period*;* and/or

(ii) undertaken activity that is in-scope for the Hospital Services Payment, as defined under the *National Partnership on COVID-19 Response*, during the reporting period; and

(iii) met the reporting requirements in relation to COVID-19 expenditure, as prescribed under the *National Partnership on COVID-19 Response* and by the Department.

(c) Additional costs that are reimbursed through the State Public Health Payment and the Hospital Services Payment will be excluded from the calculation of activity eligible for funding under the terms of the *National Health Reform Agreement*.

(d) There is no guarantee that the HHS will have access to additional funding under the State Public Health Payment or the Hospital Services Payment.

(e) All funding that is provided through the State Public Health Payment and the Hospital Services Payment will be subject to a reconciliation to be undertaken by the Administrator of the National Health Funding Pool. The HHS will be required to provide evidence to support claims for additional costs. If the HHS is unable to provide the required evidence with their expenditure claim, funding received may be recalled subject to reconciliation.

(f) Funding adjustments will be actioned through the processes outlined in Schedules 2 and 4 of this service agreement.

6.3 Purchasing incentives

(a) The purchasing approach includes a range of funding adjustments which aim to incentivise high quality and high priority activity, support innovation and evidence-based practice, deliver additional capacity through clinically and cost effective models of care and dis-incentivise care which provides insufficient or no benefit for patients. This includes incentive payments for HHSs who achieve targets in specific priority areas. The purchasing incentives are detailed in Table 3. The Department must reconcile the applicable purchasing incentives in Table 3 in line with the timeframes specified in the relevant purchasing specification sheet. The Department must provide a copy of the reconciliation statement to the HHS.

(b) Funding adjustments must be based on the requirements contained in the relevant purchasing specification sheet for that purchasing incentive.

(c) Any resulting changes to funding and purchased activity levels will be actioned through the processes outlined in Schedules 2 and 4 of this service agreement.

Table 3 Purchasing Incentives 2022/23

Incentive	
**Quality Improvement Payment (QIP)**	
Antenatal care for First Nations Women	Payments for achieving two Closing the Gap targets for First Nations women:<br>• to attend five or more antenatal visits with their first antenatal first taking place in the first trimester; and<br>• to stop smoking by 20 weeks gestation.
**Purchasing incentives**	
Virtual care incentive	Incentive funding to increase the number of specialist outpatient services which are provided in virtual settings.
Own source revenue growth	Incentivise the recognition of own source revenue through matching growth in own source revenue with public activity growth funding.

Incentive	
**ABF model localisations**	
Sentinel events	Payment withdrawn for sentinel events as per the national ABF model.
Advance Care Planning (ACP)	QWAUs for HHSs who offer ACP discussions to admitted patients, non-admitted outpatients, community health patients and Emergency Department patients.
Emergency Department Did Not Wait (DNW)	Zero QWAUs for DNWs.
Fractured neck of femur	QWAUs reduced by 20% for non-timely surgical treatment of fractured neck of femur.
Hospital in The Home (HITH)	QWAUs increased for Hospital in the Home (HITH) admissions of Residential Aged Care Facility residents.
Out-of-scope services	Nil QWAUs for out-of-scope procedures.
Pre-operative bed days	QWAUs for long stay days above the upper trim point less ICU days reduced equivalent to pre-operative days, up to a maximum of 3 days, for elective episodes.
Smoking cessation (community mental health)	QWAUs for smoking cessation activity for community mental health patients.
Smoking cessation (inpatients)	QWAUs for smoking cessation activity for publicly funded inpatients.
Stroke care	10% QWAU loading for acute stroke patients admitted to Statewide Stroke Clinical Network-endorsed stroke unit care.
Telehealth (admitted patients)	QWAUs for provider-end of in-scope admitted patient telehealth activity.
**Commissioning mechanisms**	
High-cost home support	Funding for approved individuals requiring 24-hour home ventilation.
Patient flow initiative	Provision of non-recurrent WAU-backed funding to participating HHS who successfully implement agreed recommendations.
Rapid access clinics	Recurrent WAU-backed funding to support the implementation of rapid access clinics to reduce pressure on emergency departments.
Expansion of sub-acute and long stay care	Additional funding to increase the availability of and access to care for sub-acute and long stay patients, thereby improving access to care in a range of settings and releasing capacity within acute facilities.
Connected Community Pathways	Funding to incentivise evidence-based and innovative models of care which promote the delivery of care outside acute facilities and support shared-care partnership arrangements.

6.4 **Surgery Connect reimbursements**

(a) The HHS will reimburse the Department for the actual costs of activity for nominated patient referrals to the Surgery Connect program where:

(i) The HHS has nominated the patient referral as HHS funded on entry of the referral in the Surgery Connect Activity Navigator (SCAN); and

(ii) The HHS Chief Finance Officer has recorded approval of the nomination in SCAN;

or

(iii) The HHS has obtained the Department's agreement to retrospectively convert a defined patient cohort to HHS funded status in SCAN.

(b) The HHS may only request retrospective conversion of activity to HHS funded within the financial year in which the activity has taken place.

(c) Any resulting changes to funding and purchased activity levels will be actioned through the processes outlined in Schedules 2 and 4 of this service agreement.

6.5 **Financial adjustments – other**

(a) Notwithstanding the provisions regarding the recognition of revenue as stipulated in AASB1058 *Income of Not-for-Profit Entities* and/or AASB15 *Revenue from Contracts with Customers*, the Department may seek to recover funding from an HHS that was provided through this service agreement which has:

(i) not been utilised in accordance with its intended purpose; and/or

(ii) not been utilised within the prescribed time period to deliver the agreed outcomes/services.

(b) If the Chief Executive (or delegate) determines that previously allocated funding is to be recovered, any resulting changes to funding and purchased activity levels will be actioned through the processes outlined in Schedules 2 and 4 of this service agreement.

6.6 **Public and private activity/own source revenue**

(a) Own source revenue comprises grants and contributions, user charges and other revenues.

(b) Where an HHS is above its own source revenue target, it will be able to retain the additional own source revenue with no compensating adjustments to funding from other sources where the additional revenue is not attributable to a private patient consistent with clauses A9 to A13 and A44 of the *National Health Reform Agreement.*

(c) Where an HHS is below its own source revenue target in respect of private patients, it will experience a reduction in revenue with no compensating adjustments to funding from other sources.

(d) The HHS will make all efforts to identify and maximise revenue from appropriate third parties to ensure optimisation of available State and Commonwealth funding across the Queensland public sector health system.

(e) The own source revenue identified in Table 4 is an estimate generated by the HHS which allows all third-party funding sources associated with service delivery

to be identified. The HHS will ensure that this estimate is substantiated and accurate to ensure no significant variances to actual revenue generated.

(f) The HHS will routinely revise and update the estimate to ensure alignment between the service agreement and Queensland Treasury's reporting system (TRIDATA).

(g) Budget adjustments for changes in own source revenue from private patients will be actioned through the processes set out in Schedule 4 of this service agreement.

7. Funding sources

7.1 The four main funding sources contributing to the HHS service agreement value are:

(a) Commonwealth funding;

(b) State funding;

(c) grants and contributions; and

(d) own source revenue.

7.2 Table 4 provides a summary of the funding sources for the HHS and the total value of the service agreement.

8. Funds disbursement

8.1 The Department agrees to pay the HHS the amount described in Table 4 of Schedule 2, subject to:

(a) parliamentary appropriation and adequate funds being allocated to the Department; and

(b) the terms of this service agreement.

8.2 All payments under this service agreement will be made in accordance with the requirements of the *National Health Reform Agreement* and the Act.

8.3 The Chief Executive will direct the disbursement of both State and Commonwealth funding from the State Pool Account, the State Managed Fund and the Department of Health Expenditure account to the HHS.

8.4 However, the State (represented by the Chief Executive) will not:

(a) redirect Commonwealth payments between HHSs;

(b) redirect Commonwealth payments between funding streams (e.g., from ABF to block funding); and/or

(c) adjust the payment calculations underpinning the Commonwealth's funding.

8.5 The Department will pay state-funded activity-based funding, block funding and system manager funding to the HHS on a fortnightly basis in line with receipt of the State Appropriation Payment. The payment made will be equal to 1/26th of the state-funded component of the service agreement value described in Table 4.

8.6 The Department will pay Commonwealth-funded activity-based funding and block funding on a monthly basis in line with receipt of the Commonwealth payment. The payment made will be equal to 1/12th of the Commonwealth funded component of the service agreement value described in Table 4.

8.7 Where the parties have agreed to amend the service agreement value or the Commonwealth contribution amount changes, the fortnightly and/or monthly payments will be adjusted to reflect the amended service agreement value.

8.8 Service agreement payments may be made outside of these timeframes with the approval of the Department's Chief Finance Officer.

Table 4 Cairns and Hinterland HHS Total Funding Allocation by Funding Source 2022/23

Funding Source	NWAU (N2122)	QWAU (Q24)	Value ($)
**NHRA Funding**			
**ABF Pool**			
ABF Funding (in scope NHRA)[2]			
Commonwealth[2]	140,357		$329,639,213
State		141,274	$387,941,594
State Specified Grants			$18,310,166
State-wide Services			$0
**State Managed Fund**			
Block Funding			
Small Rural Hospitals		12,720	$63,801,464
Teaching, Training & Research			$25,377,133
Non-Admitted Child & Youth Mental Health			$16,310,027
Non-Admitted Home Ventilation			$0
Non-Admitted Mental Health			$56,010,824
Other Non-Admitted Service			$0
Highly Specialised Therapies			$0
**Total NHRA Funding**	**140,357**	**153,994**	**$897,390,423**
**Out of Scope NHRA**			
**Queensland ABF Model**			
DVA		1,214	$7,519,225
NIISQ/MAIC		644	$3,145,809
Oral Health		2,668	$14,462,466
BreastScreen		748	$4,628,216
**Total Queensland ABF Funding**	**-**	**5,274**	**$29,755,716**
**Discretely Funded Programs[3]**			
Department of Health			$76,694,317
Locally receipted funds			$8,383,057
**Total Discretely Funded Programs**	**-**	**-**	**$85,077,374**
**Own Source Revenue**			
Private Patient Admitted Revenue[4]	*2,710*	2,995	$15,168,969
Non-Admitted Services		7,076	$14,358,776
Pharmaceuticals Benefits Scheme		3,836	$45,526,810
Other Activities[5]		1,218	$25,181,796
**Total Own Source Revenue**	**-**	**15,125**	**$100,236,351**

[2] The split between Commonwealth and State NHRA funding will change during the year as the purchased activity targets are updated and the National Health Funding Board updates their payment advice accordingly.

[3] Includes all other (non-ABF) State and Commonwealth funded health services including, but not limited to, Prisoner Health, Public Health and Prevention Services.

[4] The estimated value of the revenue earned from private patients, based on OSR estimates provided by the HHS.

[5] Incorporates all OSR which is not identified elsewhere in Table 4.

Funding Source	NWAU (N2122)	QWAU (Q24)	Value ($)
Locally Receipted Funds (exc. Discretely Funded Programs)[6]			$3,109,975
Depreciation			$63,528,000
**NPA COVID-19 Response**			
Hospital Services Payment			$0
State Public Health Payment			$0
COVID-19 Vaccine Payment			$0
**Total NPA COVID-19 Response Funding**	**-**	**-**	**$0**
**GRAND TOTAL**	**140,357**	**174,393**	**$1,179,097,839**

Pool Accounts			
ABF Pool (National Health Funding Pool)[7]			$765,646,690
State Managed Fund[8]			$161,499,449
System Manager			$76,694,317

[6] Includes items such as training programs and donations. Does not include locally receipted funds associated with discretely funded programs, e.g.Transition Care.
[7] Articulates the financial payment made to support in-scope ABF services under the NHRA including DVA, NIISQ, MAIC and Breastscreen Services. Applies to all HHSs except Central West HHS and Torres and Cape HHS.
[8] Articulates the payment made for block funded services under the NHRA, DVA, NIISQ and MAIC services.

Table 5 National Health Reform Funding

NHRA Funding Type	NWAU (N2122)	Commonwealth ($)	State ($)	Other State funding[9] DVA/MAIC/Oral Health/BreastScreen ($)	Total ($)
**National Efficient Price (NEP)**					**$5,597**
**ABF Allocation (NWAU)**					
Emergency Department	19,295	$45,315,672	$55,847,638	$5,922,545	$107,085,855
Acute Admitted	90,871	$213,418,977	$263,020,392	$0	$476,439,369
Admitted Mental Health	6,319	$14,841,624	$18,291,015	$0	$33,132,639
Sub-Acute	8,421	$19,776,332	$24,372,616	$0	$44,148,948
Non-Admitted	15,450	$36,286,608	$44,720,100	$23,833,171	$104,839,879
**Total ABF Pool Allocation**	**140,357**	**$329,639,213**	**$406,251,760**	**$29,755,716**	**$765,646,690**
**Block Allocation**					
Teaching Training and Research	-	$5,232,484	$20,144,650	-	$25,377,133
Small and Rural Hospitals[10]	-	$23,019,772	$40,781,693	-	$63,801,464
Non-Admitted Mental Health	-	$21,328,990	$34,681,834	-	$56,010,824
Non-Admitted Child & Youth Mental Health	-	$1,663,825	$14,646,202	-	$16,310,027
Non-Admitted Home Ventilation	-	$0	$0	-	$0
Other Non-Admitted Services	-	$0	$0	-	$0
Other Public Hospital Programs	-	$0	$0	-	$0
Highly Specialised Therapies	-	$0	$0	-	$0
**Total Block Allocation**	**-**	**$51,245,070**	**$110,254,379**	**-**	**$161,499,449**
**Grand Total Funding Allocation**					**$927,146,139**

[9] State funding transacted through the Pool Account; not covered under the NHRA

[10] Incorporating small regional and rural public hospitals, five specialist mental health facilities (Baillie Henderson Hospital, Jacaranda Place – Queensland Adolescent Extended Treatment Centre, The Park – Centre for Mental Health, Kirwan Rehabilitation Unit and Charters Towers Rehabilitation Unit) and the Ellen Barron Family Centre.

Table 6 Discretely Funded Programs (Non-ABF)

Discretely Funded Programs	$
Aged Care Assessment Program	$1,332,379
Alcohol, Tobacco and Other Drugs	$4,086,319
Community Health Programs	$14,923,094
Disability Residential Aged Care Services	$0
Home and Community Care Program (HACC)	$5,684,218
Interstate Patients (QLD residents)	$4,606,598
Multi-purpose Health Services	$3,380,071
Other State Funding	$1,483,426
Patient transport	$12,416,504
Prevention Services and Public Health	$23,213,334
Prisoner Health Services	$9,369,814
Research	$703,817
Transition Care	$2,698,839
Residential Aged Care Services	$1,178,962
**TOTAL**	**$85,077,374**

Schedule 3 Performance Measures

1. Performance Measures

1.1 The performance of the HHS will be measured according to the assessment criteria and processes described in the Performance and Accountability Framework.

1.2 The detailed specification for each of the performance measures listed in this service agreement are provided through performance measure attribute sheets.

1.3 The performance measures identified in this service agreement are applicable to the HHS unless otherwise specified within the attribute sheet.

1.4 HHSs are also required to report against the agreed key performance measures in their Health Equity Strategy.

Table 7 HHS Performance Measures – Key Performance Indicators

Key Performance Indicators
Hospital Acquired Complications
Hospital Access Target (admitted patients)<br>• % of emergency stays within 4 hours
Emergency Department stays greater than 24 hours
Emergency Department wait time by triage category
Face to face community follow up within 1-7 days of discharge from an acute mental health inpatient unit
Patient off stretcher time
Lost minutes per ambulance (in development)
Patient flow target: time between the decision to admit and patient leaving the Emergency Department (in development)
Category 1 elective surgery patients treated within the clinically recommended timeframe
Elective surgery patients waiting longer than the clinically recommended timeframe
Emergency Surgery (placeholder - measure to be determined)
Category 1 patients who receive their initial specialist outpatient appointment within the clinically recommended timeframe
Patients waiting longer than clinically recommended for their initial specialist outpatient appointment
Category 4 gastrointestinal endoscopy patients treated within the clinically recommended timeframe
Gastrointestinal endoscopy patients waiting longer than the clinically recommended timeframe
Access to oral health services (adults)
Access to oral health services (children)
Potentially Preventable Hospitalisations – First Nations peoples:<br>• Diabetes complications<br>• Selected conditions
Reduction in the proportion of Aboriginal and Torres Strait Islander failure to attend appointments
Telehealth utilisation rates:<br>• Number of non-admitted telehealth service events
Forecast operating position:<br>• Full year<br>• Year to date

Average sustainable Queensland Health FTE
Capital expenditure performance
Proportion of mental health and alcohol and other drug service episodes with a documented care plan
Proportion of overnight inpatients discharged by 10am

Table 8 HHS Performance Measures - Safety and Quality Markers

Safety and Quality Markers
Sentinel Events
Hospital Standardised Mortality Ratio
Healthcare-associated Staphylococcus Aureus (including MRSA) bacteraemia
Severity Assessment Code (SAC) analysis completion rates
Patient Reported Experience

Table 9 HHS Performance Measures – Outcome Indicators

Outcome Indicators
Rate of seclusion events
Rate of absent without approval from acute mental health inpatient care
Reperfusion therapy for acute ischaemic stroke
Access to emergency dental care
First Nations peoples representation in the workforce
General oral health care for First Nations peoples
% of low birthweight babies born to Queensland mothers
Complaints resolved within 35 calendar days
Advance care planning
Smoking cessation clinical pathway
Potentially Preventable Hospitalisations (diabetes complications)
Potentially Preventable Hospitalisations (non-diabetes complications)
The percentage of oral health activity which is preventive
Cardiac rehabilitation
Adolescent vaccinations administered via the statewide School Immunisation Program

Schedule 4 Amendments to this service agreement

1. Agreed process to amend this service agreement

1.1 The parties acknowledge that this service agreement is subject to amendment, which will generally occur through:

(a) amendment windows;

(b) extraordinary amendment;

(c) periodic adjustments; and

(d) end of financial year reconciliation.

1.2 **Amendment windows**

(a) There will be set periods of time nominated by the Department during the year (amendment windows) in which a party may propose an amendment and the parties will endeavour to negotiate and finalise proposals to amend this service agreement.

(b) Amendment proposals that are agreed will be documented in a deed of amendment to this service agreement.

(c) Further details on the amendment window process, including the timing of amendment windows, is provided online, as detailed in Appendix 1.

1.3 **Extraordinary Amendment**

(a) Outside an amendment window, the Department and the HHS agree to limit any proposal to amend the terms of this service agreement to those where there is an urgent priority need to facilitate a funding allocation (extraordinary amendment). The parties will endeavour to negotiate and finalise any such proposal urgently.

(b) The process for submitting, negotiating and resolving an extraordinary amendment is available online, as detailed at Appendix 1.

(c) Agreed extraordinary amendments will be reflected in a notice issued by the Chief Executive countersigned as accepted by the HHS, which notice will be replaced when the extraordinary amendment is subsequently formalised in a deed of amendment issued following the next amendment window.

1.4 **Periodic adjustments**

(a) The service agreement value (and corresponding purchased activity) may be adjusted at any time to reflect funding variations that:

(i) occur on a periodic basis or in line with adjustments permitted for specific funding allocations;

(ii) are referenced in the service agreement; and

(iii) are based on a clearly articulated formula, an agreed basis or such other reasonably substantiated basis tied to performance.

(periodic adjustment).

(b) Periodic adjustments will be reflected in an adjustment notice issued by the Chief Executive (or delegate) to the HHS, based on relevant data, and subsequently formalised in a deed of amendment issued following the next amendment window.

1.5 End of financial year reconciliation

(a) There will be an end of financial year reconciliation process, with the scope defined by the Department and informed by Queensland Government Central Agency requirements.

(b) The Department will provide the HHS with a reconciliation of all service agreement funding and purchased activity for the prior financial year. This will reflect the position following conclusion of the end of financial year adjustments process.

(c) The impact of end of financial year adjustments on subsequent year funding and activity will be incorporated in the service agreement through the deed of amendment executed following the next available amendment window.

(d) This clause will survive expiration of this service agreement.

Appendix 1 Reference Documents

Hospital and Health Boards Act 2011

National Health Reform Agreement (NHRA) 2020-25

System Outlook to 2026 - for a sustainable health service

Queensland Health Performance and Accountability Framework

My health, Queensland's future: Advancing health 2026

Department of Health Strategic Plan 2021-2025

Local Area Needs Assessment (LANA) Framework

Making Tracks Together: Queensland's Aboriginal and Torres Strait Islander Health Equity Framework

Making Tracks toward closing the gap in health outcomes for Indigenous Queenslanders by 2033 - Policy and Accountability Framework

Aboriginal and Torres Strait Islander Health Workforce Strategic Framework 2016-2026

National Agreement on Closing the Gap

Queensland Health Workforce Diversity and Inclusion Strategy 2017 to 2022

Performance Measures Attribute Sheets

Purchasing Initiatives and Funding Specifications

Public Health Practice Manual

National Partnership on COVID-19 Response

Statewide services reference material

Service agreement amendment processes

Data supply requirements

Contracting and Performance Management Branch
GPO Box 48
Brisbane QLD 4001 Australia

Tel (+61) (07) 3708 5869

www.health.qld.gov.au

Service Agreement

2019/20 – 2021/22

Mackay Hospital and Health Service

July 2020 Revision

Queensland Government

Mackay Hospital and Health Service

Service Agreement 2019/20 - 2021/22, July 2020 Revision

Published by the State of Queensland (Queensland Health), July 2020

This document is licensed under a Creative Commons Attribution 3.0 Australia licence. To view a copy of this licence, visit creativecommons.org/licenses/by/3.0/au

© State of Queensland (Queensland Health) **2020**

You are free to copy, communicate and adapt the work, as long as you attribute the State of Queensland (Queensland Health).

For more information contact:
Contracting and Performance Management Branch, Department of Health, GPO Box 48, Brisbane QLD 4001, email SAM@health.qld.gov.au, phone (07) 3708 5869.

An electronic version of this document is available at www.health.qld.gov.au/system-governance/health-system/managing/default.asp

Disclaimer:
The content presented in this publication is distributed by the Queensland Government as an information source only. The State of Queensland makes no statements, representations or warranties about the accuracy, completeness or reliability of any information contained in this publication. The State of Queensland disclaims all responsibility and all liability (including without limitation for liability in negligence) for all expenses, losses, damages and costs you might incur as a result of the information being inaccurate or incomplete in any way, and for any reason reliance was placed on such information.

Contents

1. Introduction ... 6
2. Interpretation ... 6
3. Legislative and regulatory framework ... 7
4. Health system priorities ... 7
5. Objectives of the Service Agreement ... 9
6. Scope ... 9
7. Performance and Accountability Framework ... 9
8. Period of this Service Agreement ... 10
9. Amendments to this Service Agreement ... 10
10. Publication of amendments ... 10
11. Cessation of service delivery ... 10
12. Commencement of a new service ... 11
13. Provision of data to the Chief Executive ... 12
14. Dispute resolution ... 12
15. Force Majeure ... 15
16. Hospital and Health Service accountabilities ... 16
17. Department accountabilities ... 16
18. Insurance ... 18
19. Indemnity ... 18
20. Indemnity arrangements for officers, employees and agents ... 19
21. Legal proceedings ... 19
22. Sub-contracting ... 19
23. Counterparts ... 20

Execution ... 21

Schedule 1 HHS Accountabilities ... 22

1. Purpose ... 22
2. Registration, credentialing and scope of clinical practice ... 22
3. Clinical Services Capability Framework ... 22
4. Clinical Prioritisation Criteria ... 23
5. Service delivery ... 23
6. Accreditation ... 24
7. Responsive regulatory process for accreditation ... 25
8. Achieving health equity for First Nations Queenslanders ... 26
9. Provision of Clinical Products/Consumables in outpatient settings ... 27
10. Capital, land, buildings, equipment and maintenance ... 28
11. Occupational health and safety ... 30
12. Workforce management ... 30
13. Medically authorised ambulance transports ... 31

Schedule 2 Funding, purchased activity and services ... 32

1. Purpose ... 32
2. Delivery of purchased activity ... 32
3. Financial adjustments ... 33

4. Funding sources 46
5. Funds disbursement 48
6. Purchased services 57
7. Teaching training and research 63
Schedule 3 Performance Measures 66
1. Purpose 66
2. Performance Measures 66
Schedule 4 Data Supply Requirements 71
1. Purpose 71
2. Principles 71
3. Roles and responsibilities 71
Attachment A Data Supply Requirements 73
Schedule 5 Amendments to this Service Agreement 75
1. Purpose 75
2. Principles 75
3. Process to amend this Service Agreement 75
Schedule 6 Definitions 80
Key Documents 87
Abbreviations 89

Figures

Figure 1 Dispute resolution process ... 13
Figure 2 Inter-HHS dispute resolution process ... 15
Figure 3 Amendment Proposal negotiation and resolution ... 77

Tables

Table 1 Specific Funding Commitments ... 34
Table 2 Purchasing Incentives 2020/21 (Summary) ... 45
Table 3 Hospital and Health Service funding sources 2020/21 ... 47
Table 4 HHS Finance and Activity Schedule 2019/20 – 2021/22 – Summary by Purchasing Hierarchy ... 49
Table 5 Minor Capital and Equity ... 51
Table 6 HHS Finance and Activity Schedule 2019/20 – 2021/22 Other Funding Detail ... 52
Table 7 Specified Grants ... 55
Table 8 Hospital and Health Service Service Agreement and State Level Block Payments to state managed funds from Commonwealth payments into national funding pool ... 56
Table 9 HHS Performance Measures – Key Performance Indicators ... 67
Table 10 HHS Performance Measures - Safety and Quality Markers ... 68
Table 11 HHS Performance Measures – Outcome Indicators ... 69
Table 12 Clinical data ... 73
Table 13 Non-clinical data ... 74
Table 14 Amendment Window Exchange Dates ... 76

1. Introduction

1.1 The Queensland Public Sector Health System is committed to strengthening performance and improving services and programs in order to meet the needs of the community and deliver improved health outcomes to all Queenslanders.

1.2 The development of Service Agreements between the Chief Executive and Hospital and Health Services (HHSs), assists this process by formally assigning accountability for the high-level outcomes and targets to be met during the period to which the Service Agreement relates.

1.3 The content and process for the preparation of this Service Agreement is consistent with the requirements of the *Hospital and Health Boards Act 2011*. As such this Service Agreement specifies:

(a) the Health Services and other services to be provided by the HHS;

(b) the funding which is provided to the HHS for the provision of these services and the way in which the funding is to be provided;

(c) the Performance Measures that the HHS will meet for the services provided;

(d) data supply requirements; and

(e) other obligations of the Parties.

1.4 Fundamental to the success of this Service Agreement is a strong collaboration between the HHS and its Board and the Department. This collaboration is supported through regular Performance Review Meetings attended by representatives from both the HHS and the Department which provide a forum within which a range of aspects of HHS and system wide performance are discussed and jointly addressed.

2. Interpretation

Unless expressed to the contrary, in this Service Agreement:

(a) words in the singular include the plural and vice versa;

(b) any gender includes the other genders;

(c) if a word or phrase is defined its other grammatical forms have corresponding meanings;

(d) “includes” and “including” are not terms of limitation;

(e) no rule of construction will apply to a clause to the disadvantage of a Party merely because that Party put forward the clause or would otherwise benefit from it;

(f) a reference to:

(i) a Party is a reference to a Party to this Service Agreement;

(ii) a person includes a partnership, joint venture, unincorporated association, corporation and a government or statutory body or authority;

(iii) a person includes the person's legal personal representatives, successors, assigns and persons substituted by novation;

(g) any legislation includes subordinate legislation under it and includes that legislation and subordinate legislation as modified or replaced;

(h) a reference to a role, function or organisational unit is deemed to transfer to an equivalent successor role, function or organisational unit in the event of organisational change or restructure in either Party;

(i) an obligation includes a warranty or representation and a reference to a failure to comply with an obligation includes a breach of warranty or representation;

(j) headings do not affect the interpretation of this Service Agreement;

(k) unless the contrary intention appears, a reference to a Schedule, annexure or attachment is a reference to a Schedule, annexure or attachment to this Service Agreement; and

(l) unless the contrary intention appears, words in the Service Agreement that are defined in Schedule 6 'Definitions' have the meaning given to them in that Schedule.

3. Legislative and regulatory framework

3.1 This Service Agreement is regulated by the National Health Reform Agreement and the provisions of the *Hospital and Health Boards Act 2011.*

3.2 The National Health Reform Agreement requires the State of Queensland to establish Service Agreements with each HHS for the purchasing of health services and to implement a performance and accountability framework including processes for remediation of poor performance. The *Hospital and Health Boards Act 2011* states under section 35(3) that the Service Agreement executed between the Chief Executive and the Hospital and Health Board Chair binds each of them.

3.3 The *Hospital and Health Boards Act 2011* recognises and gives effect to the principles and objectives of the national health system agreed by the commonwealth, state and territory governments, including the Medicare principles and health system principles set out in section 4. Section 5 of the *Hospital and Health Boards Act 2011* states that the object of the Act is to establish a Public Sector Health System that delivers high-quality hospital and other Health Services to persons in Queensland having regard to the principles and objectives of the national health system. This Service Agreement is an integral part of implementing these objectives and principles.

4. Health system priorities

4.1 Ensuring the provision of Public Sector Health Services across Queensland requires clear priorities, supportive leadership and staff who work together and across each level of the Public Sector Health System. The Parties recognise that they each have a mutual and reciprocal obligation to work collaboratively with each other, with other Hospital and Health

Services (HHS) and with the Queensland Ambulance Service in the best interests of the Queensland Public Sector Health System.

4.2 The priorities, goals and outcomes for the Queensland Public Sector Health System are defined through:

(a) *Our Future State: Advancing Queensland's Priorities* - the Queensland Government's objectives for the community; and

(b) *My health, Queensland's future: Advancing health 2026* – the vision and strategy for Queensland's health system.

4.3 The Parties will also work collaboratively to deliver the *Queensland Health 2020/21 System Priorities.* The *Queensland Health 2020/21 System Priorities* establishes a tactical framework which will ensure that the Queensland Public Sector Health System delivers sustainable, high quality and timely Health Services during 2020/21, whilst remaining positioned to respond effectively to the COVID-19 pandemic.

4.4 Additionally, the Queensland Government, Premier or the Minister for Health and Minister for Ambulance Services (The Minister) may articulate key priorities, themes and issues from time to time.

4.5 HHSs have a responsibility to ensure that the delivery of Public Sector Health Services in Queensland is consistent with these strategic directions and priorities.

4.6 The Parties will collectively identify, develop, implement and evaluate strategies that support the delivery of priorities identified by the Minister, and which align with a Value-Based Healthcare approach to the delivery of Health Services.

4.7 In accordance with section 9 of the *Financial and Performance Management Standard 2009*, HHSs are required to develop a strategic plan. The HHS's strategic plan will reflect local priorities and will be developed considering the shared Queensland priorities outlined in the Queensland Government's objectives for the community, the Ministers' articulated priorities and *My health, Queensland's future: Advancing health 2026*.

4.8 The Parties have a collective responsibility to contribute to a sustainable Public Sector Health System in Queensland. Planning and delivery of Health Services will be aligned with the system planning agenda set out in *Queensland Health System Outlook to 2026 for a sustainable health service* in order to ensure a coordinated, system-wide response to growing demand for Health Services.

4.9 In delivering Health Services, HHSs are required to meet the applicable conditions of all national agreements and national partnership agreements between the Queensland Government and the Commonwealth Government and commitments under any related implementation plans.

4.10 This Service Agreement is underpinned by and is to be managed in line with the following supporting documents:

(a) Queensland Health System Outlook to 2026 for a sustainable health service;

(b) Performance and Accountability Framework 2020/21; and

(c) Purchasing Policy and Funding Guidelines 2020/21.

5. Objectives of the Service Agreement

This Service Agreement is designed to:

(a) specify the Health Services, teaching, research and other services to be provided by the HHS;

(b) specify the funding to be provided to the HHS for the provision of the services;

(c) specify the Performance Measures for the provision of the services;

(d) specify the performance and other data to be provided by the HHS to the Chief Executive;

(e) provide a platform for greater public accountability; and

(f) facilitate the achievement of State and Commonwealth Government priorities, services, outputs and outcomes while ensuring local input.

6. Scope

6.1 This Service Agreement outlines the services that the Department will purchase from the HHS during the period of this Service Agreement.

6.2 This Service Agreement does not cover the provision of clinical and non-clinical services by the Department, including the Queensland Ambulance Service, to the HHS. Separate arrangements will be established for those services provided by Health Support Queensland and eHealth Queensland.

7. Performance and Accountability Framework

7.1 The Performance and Accountability Framework sets out the framework within which the Department, as the overall manager of Public Health System Performance, monitors and assesses the performance of Public Sector Health Services in Queensland. The systems and processes employed for this purpose include, but are not limited to, assessing and monitoring HHS performance, reporting on HHS performance and, as required, intervening to manage identified performance issues.

7.2 During 2020/21 the Performance and Accountability Framework will support delivery of the *Queensland Health System Priorities 2020/21* which focus on realising positive changes to the Public Sector Health System through providing sustainable, timely, safe and high-quality Health Services in the right setting whilst remaining ready to respond to the COVID-19 pandemic.

7.3 The Performance and Accountability Framework uses Performance Measures to monitor the extent to which HHSs are delivering the high-level objectives set out in this Service Agreement. The Key Performance Indicators and other measures of performance against which the HHS will be assessed and benchmarked are detailed in Schedule 3 of this Service Agreement.

7.4 The Parties agree to constructively implement the Performance and Accountability Framework.

8. Period of this Service Agreement

8.1 This Service Agreement commences on the Effective Date and expires on 30 June 2022. The Service Agreement framework is in place for three years in order to provide HHSs with a level of guidance regarding funding and purchased activity for the outer years.

8.2 In this Service Agreement, references to years are references to the period commencing on 1 July and ending on 30 June unless otherwise stated.

8.3 Using the provisions of the *Hospital and Health Boards Act 2011* as a guide, the Parties will enter into funding and purchased activity negotiations for the following year six months before the end of the current year.

8.4 In accordance with the *Hospital and Health Boards Act 2011* the Parties will enter negotiations for the next Service Agreement at least six months before the expiry of the existing Service Agreement.

9. Amendments to this Service Agreement

9.1 Section 39 of the *Hospital and Health Boards Act 2011* requires that, if the Chief Executive or the HHS wish to amend the terms of a Service Agreement, the Party wishing to amend the Service Agreement must give written notice of the proposed amendment to the other Party.

9.2 The process for amending this Service Agreement is set out in Schedule 5 of this Service Agreement.

10. Publication of amendments

The Department will publish each executed Deed of Amendment within 14 days of the date of execution on www.health.qld.gov.au/system-governance/health-system/managing/default.asp.

11. Cessation of service delivery

11.1 The HHS is required to deliver the Health Services and other services outlined in this Service Agreement for which funding is provided in Schedule 2. Any changes to service delivery must ensure maintenance of care and minimise disruptions to patients.

11.2 The Department and HHS may Terminate or temporarily Suspend a Health Service or other service by mutual agreement having regard to the following obligations:

(a) any proposed Termination or Suspension must be made in writing to the other Party;

(b) where it is proposed to Terminate or Suspend a Statewide Service, or a Regional Service, the HHSs which are in receipt of that service must also be consulted;

(c) the Parties must agree on a reasonable notice period following which Termination, or Suspension, will take effect; and

(d) patient needs, workforce implications, relevant government policy and HHS sustainability are to be considered.

11.3 The Department, in its role as the Queensland Public Health System manager:

(a) may, in its unfettered discretion, not support a requested Termination or Suspension and require the HHS to maintain the service; and

(b) will reallocate existing funding and activity for the Terminated or Suspended service inclusive of baseline Service Agreement funding and in-year growth funding on a pro-rata basis.

11.4 The HHS will:

(a) work with the Department to ensure continuity of care and a smooth transfer of the service to an alternative provider where this is necessary; and

(b) minimise any risk or inconvenience to patients associated with service Termination, Suspension or transfer.

11.5 In the event that a sustainable alternative provider cannot be identified, and this is required, the service and associated patient cohort will continue to remain the responsibility of the HHS.

12. Commencement of a new service

12.1 In the event that the HHS wishes to commence providing a new Health Service, the HHS will notify the Department in writing in advance of commencement.

12.2 The Department will provide a formal response regarding the proposed new Health Service to the HHS in writing. The Department may not agree to purchase the new Health Service or to provide funding on either a recurrent or non-recurrent basis.

12.3 In the event that a change to an established Referral Pathway is proposed which would result in the direction of patient referrals to an alternative HHS on a temporary or a permanent basis:

(a) the new Referral Pathway must be agreed by all impacted HHSs prior to its implementation; and

(b) following agreement of the new Referral Pathway, if there is an identifiable and agreed impact to funding the Department will redistribute funding and activity between HHSs in alignment with new Referral Pathway.

13. Provision of data to the Chief Executive

The HHS will provide to the Chief Executive the performance data and other data, including data pursuant to ad hoc requests, set out in Schedule 4 of this Service Agreement in accordance with the Schedule, including in relation to the form, manner and the times required for the provision of data.

14. Dispute resolution

14.1 The dispute resolution process set out below is designed to resolve disputes which may arise between the Parties to this Service Agreement in a final and binding manner.

14.2 These procedures and any disputes addressed or to be addressed by them are subject to the provisions of the *Hospital and Health Boards Act 2011*, including in respect of any directions issued under that legislation or by Government in respect of any dispute.

14.3 Resolution of disputes will be through a tiered process commencing with the Performance Review Meeting and culminating, if required, with the Minister, as illustrated in Figure 1.

14.4 Use of the dispute resolution process set out in this clause should only occur following the best endeavours of both Parties to agree a resolution to an issue at the local level. The dispute resolution process is not intended for the resolution of ongoing issues or performance related issues. At each stage of the dispute resolution process, the Parties agree to cooperate.

14.5 If a dispute arises in connection with this Service Agreement (including in respect of interpretation of the terms of this Service Agreement), then either Party may give the other a written Notice of Dispute.

14.6 The Notice of Dispute must be provided to the D-SA Contact Person if the notice is being given by the HHS and to the HHS-SA Contact Person if the notice is being given by the Department.

14.7 The Notice of Dispute must contain the following information:

(a) a summary of the matter in dispute;

(b) an explanation of how the Party giving the Notice of Dispute believes the dispute should be resolved and reasons to support that belief;

(c) any information or documents to support the Notice of Dispute; and

(d) a definition and explanation of any financial or Service delivery impact of the dispute.

Figure 1 Dispute resolution process

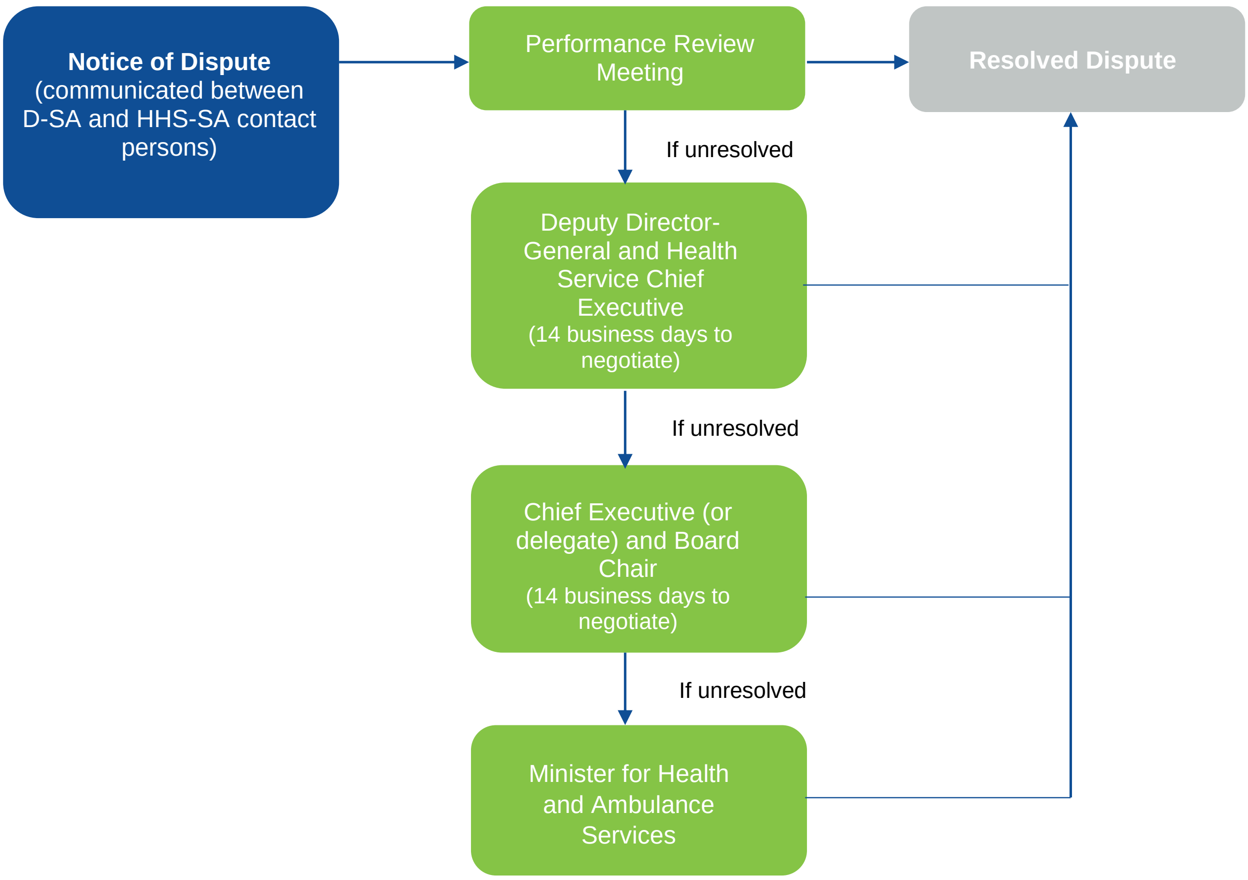

14.8 Resolution of a dispute

(a) Resolution of a dispute at any level is final. The resolution of the dispute is binding on the Parties but does not set a precedent to be adopted in similar disputes between other Parties.

(b) The Parties agree that each dispute (including the existence and contents of each Notice of Dispute) and any exchange of information or documents between the Parties in connection with the dispute is confidential and must not be disclosed to any third party without the prior written consent of the other Party, other than if required by law and only to the extent required by law.

14.9 Continued performance

Notwithstanding the existence of one or more disputes, the HHS must continue to perform and comply with this Service Agreement to the best of their abilities given the circumstances.

14.10 Disputes arising between Hospital and Health Services

(a) In the event of a dispute arising between two or more HHSs (an Inter-HHS Dispute), the process set out in Figure 2 will be initiated. Resolution of Inter-HHS Disputes will be through a tiered process, commencing with local resolution and culminating if required with formal and binding arbitration by the Minister under the provisions of the *Hospital and Health Boards Act 2011*, section 44.

(b) If the HHS wishes to escalate a dispute, the HHS will be expected to demonstrate that best endeavours (including Chair and Board involvement) to resolve the dispute between all parties at an informal and local level have taken place.

(c) Management of inter-HHS relationships should be informed by the following principles:

(i) HHSs should maintain (for both the base level of funding and growth) the proportion of out of HHS work undertaken unless as a result of agreed repatriation of patients.

(ii) All HHSs manage patients from their own catchment population if it is within their clinical capability to do so as specified by the Clinical Services Capability Framework.

(iii) Where it is proposed that a Health Service move from one HHS to another, agreement between the respective Health Service Chief Executives will be secured prior to any change in patient flows. Once agreed, funding will follow the patient.

(iv) All HHSs abide by the agreed dispute resolution process.

(v) All HHSs operate in a manner which is consistent with the health system principles and objectives as set out in the National Health Reform Agreement and the *Hospital and Health Boards Act 2011*.

Figure 2 Inter-HHS dispute resolution process

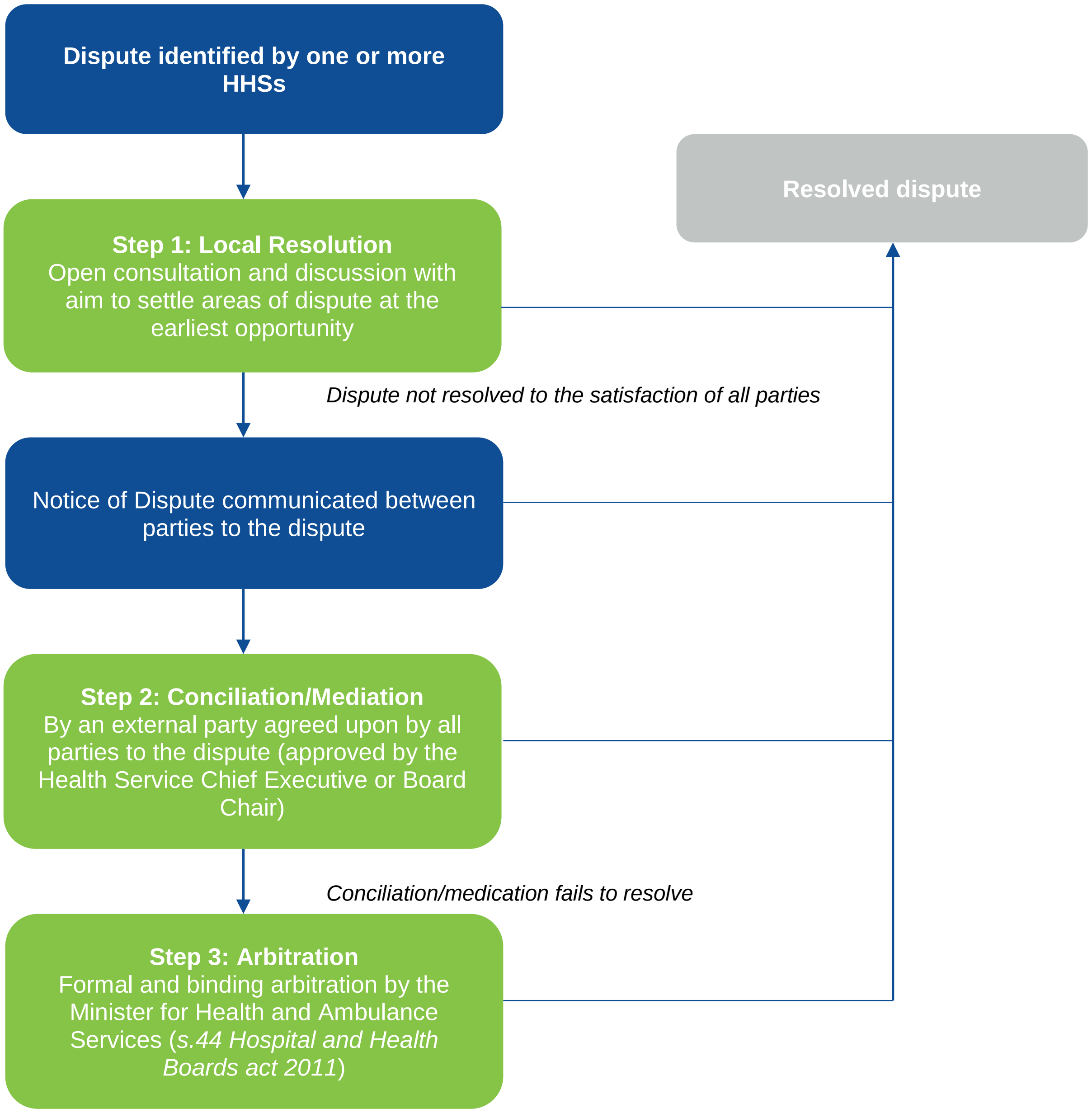

15. Force Majeure

15.1 If a Party (Affected Party) is prevented or hindered by Force Majeure from fully or partly complying with any obligation under this Service Agreement, that obligation may (subject to the terms of this Force Majeure clause) be suspended, provided that if the Affected Party wishes to claim the benefit of this Force Majeure clause, it must:

(a) give prompt written notice of the Force Majeure to the other Party of:

(i) the occurrence and nature of the Force Majeure;

(ii) the anticipated duration of the Force Majeure;

(iii) the effect the Force Majeure has had (if any) and the likely effect the Force Majeure will have on the performance of the Affected Party's

obligations under this Service Agreement; and

(iv) any disaster management plan that applies to the party in respect of the Force Majeure.

(b) use its best endeavours to resume fulfilling its obligations under this Service Agreement as promptly as possible; and

(c) give written notice to the other Party within five days of the cessation of the Force Majeure.

15.2 Without limiting any other powers, rights or remedies of the Chief Executive, if the Affected Party is the HHS and the delay caused by the Force Majeure continues for more than 14 days from the date that the Chief Executive determines that the Force Majeure commenced, the Chief Executive may give directions to the HHS regarding the HHS's performance or non-performance of this Service Agreement during the Force Majeure and the HHS must comply with that direction.

15.3 Neither Party may terminate this Service Agreement due to a Force Majeure event.

16. Hospital and Health Service accountabilities

16.1 Without limiting any other obligations of the HHS, it must comply with:

(a) the terms of this Service Agreement;

(b) all legislation applicable to the HHS, including the *Hospital and Health Boards Act 2011*;

(c) all Cabinet decisions applicable to the HHS;

(d) all Ministerial directives applicable to the HHS;

(e) all agreements entered into between the Queensland and Commonwealth governments applicable to the HHS;

(f) all regulations made under the *Hospital and Health Boards Act 2011*;

(g) all Industrial Instruments applicable to the HHS; and

(h) all health service directives applicable to the HHS.

16.2 The HHS will ensure that the accountabilities set out in Schedule 1 of this Service Agreement are met.

17. Department accountabilities

17.1 Without limiting any other obligations of the Department, it must comply with:

(a) the terms of this Service Agreement;

(b) the legislative requirements as set out within the *Hospital and Health Boards Act 2011*;

(c) all regulations made under the *Hospital and Health Boards Act 2011*; and

(d) all Cabinet decisions applicable to the Department.

17.2 The Department will work in collaboration with HHSs to ensure the Public Sector Health System delivers high quality hospital and other Health Services to persons in Queensland having regard to the principles and objectives of the national health system. In support of realising this objective, in accordance with section 5 of the *Hospital and Health Boards Act 2011* the Department will:

(a) provide overall management of the Queensland Public Sector Health System including health system planning, coordination and standard setting;

(b) provide the HHS with funding specified under Schedule 2 of this Service Agreement;

(c) provide and maintain payroll and rostering systems to the HHS unless agreed otherwise between the Parties;

(d) operate 13 HEALTH as a first point of contact for health advice with timely HHS advice and information where appropriate to local issues; and

(e) balance the benefits of a local and system-wide approach.

17.3 The Department will endeavour to purchase services in line with Clinical Prioritisation Criteria, where these have been developed, in order to improve equity of access and reflect the scope of publicly funded services.

17.4 The Department will maintain a public record of the Clinical Service Capability Framework levels for all public facilities based on the information provided by HHSs.

17.5 **Workforce management**

The Chief Executive agrees to appoint Health Service Employees to:

(a) perform work for the HHS for the purpose of enabling the HHS to perform its functions and exercise powers under the *Hospital and Health Boards Act 2011;* and

(b) deliver the services specified in this Service Agreement.

17.6 The Chief Executive, whether in the capacity of a Person Conducting a Business or Undertaking as an employer or as a building owner or occupier, will consult, cooperate and coordinate with the HHS to ensure legal compliance with the *Work Health and Safety Act 2011* and other legislation as it applies to the scope, nature and location of operations associated with this Service Agreement. This includes, but is not limited to:

(a) codes of practice;

(b) electrical safety legislation;

(c) building and fire safety legislation; and

(d) workers' compensation legislation.

18. Insurance

18.1 The HHS must hold and maintain for the period of this Service Agreement the types and levels of insurances that the HHS considers appropriate to cover its obligations under this Service Agreement.

18.2 Without limiting the types and levels of insurances that the HHS considers appropriate, any insurance policies taken out by the HHS under this clause must include appropriate coverage for the following:

(a) public and product liability insurance;

(b) professional indemnity insurance; and

(c) workers' compensation insurance in accordance with the *Workers' Compensation and Rehabilitation Act 2003* (Qld).

18.3 The HHS will be deemed to comply with its requirements under clauses 18.1 and 18.2(a) and 18.2(b) if it takes out and maintains a current insurance policy with the Queensland Government Insurance Fund.

18.4 Any insurance policies held by the HHS pursuant to this clause must be effected with an insurer that is authorised and licensed to operate in Australia.

18.5 The HHS must maintain a current register of all third-party guarantees.

18.6 The HHS must, if requested by the Department, promptly provide a sufficiently detailed certificate of currency and/or insurance and policy documents for each insurance policy held by the HHS pursuant to this clause.

18.7 The HHS warrants that any exclusions and deductibles that may be applicable under the insurance policies held pursuant to this clause will not impact on the HHS's ability to meet any claim, action or demand or otherwise prejudice the Department's rights under this Service Agreement.

18.8 The HHS must immediately advise the Department if any insurance policy, as required by this clause, is materially modified or cancelled.

19. Indemnity

19.1 The HHS indemnifies the Department against all and any liabilities, claims, actions, demands, costs and expenses made by any person which may be brought against or made upon or incurred by the Department arising directly or indirectly from or in connection with any of the following:

(a) any wilful, unlawful or negligent act or omission of the HHS, a Health Service Employee, Health Executive, Senior Health Service Employee or an officer, employee or agent working for the HHS in the course of the performance or attempted or purported performance of this Service Agreement;

(b) any penalty imposed for breach of any applicable law in relation to the HHS's performance of this Service Agreement; and

(c) a breach of this Service Agreement,

except to the extent that any act or omission by the Department caused or contributed to the liability, claim, action, demand, cost or expense.

19.2 The indemnity referred to in this clause will survive the expiration or termination of this Service Agreement.

20. Indemnity arrangements for officers, employees and agents

20.1 Indemnity arrangements for officers, employees or agents working for the Public Sector Health System are administered in accordance with the following policy documents, as amended from time to time:

(a) Indemnity for Queensland Health Medical Practitioners HR Policy I2 (QH-POL-153:2014); and

(b) Queensland Government Indemnity Guideline.

20.2 The costs of indemnity arrangements provided for Health Service Employees, Health Executives, Senior Health Service Employees, or officers, employees or agents working for the HHS are payable by the HHS.

21. Legal proceedings

21.1 This clause applies if there is any demand, claim, liability or legal proceeding relating to assets, contracts, agreements or instruments relating to the HHS, whether or not they are:

(a) transferred to an HHS under section 307 of the *Hospital and Health Boards Act 2011*; or

(b) retained by the Department.

21.2 Subject to any law, each party must (at its own cost) do all things, execute such documents and share such information in its possession and control that is relevant, and which is reasonably necessary, to enable the other party to institute or defend (as the case may be) any demand, claim, liability or legal proceeding.

22. Sub-contracting

22.1 The Parties acknowledge that the HHS may sub-contract the provision of Health Services and other services that are required to be performed by the HHS under this Service Agreement.

22.2 The HHS must ensure that any sub-contractor who has access to confidential information (as defined in section 139 of the *Hospital and Health Boards Act 2011*) and/or personal information (as defined in section 12 of the *Information Privacy Act 2009*) complies with obligations no less onerous than those imposed on the HHS.

22.3 The HHS agrees that the sub-contracting of services:

(a) will not transfer responsibility for provision of the services to the sub-contractor; and

(b) will not relieve the HHS from any of its liabilities or obligations under this Service Agreement, including but not limited to obligations concerning the provision of data in accordance with Schedule 4 (Data Supply Requirements).

23. Counterparts

23.1 This Service Agreement may be executed in two or more identical copy counterparts, each of which together will be deemed an original, but all of which together will constitute one and the same instrument.

23.2 In the event that any signature executing this Service Agreement or any part of this Service Agreement is delivered by facsimile transmission or by scanned e-mail delivery of a '.pdf' format data file or equivalent, the signature will create a valid and binding obligation of the Party executing (or on whose behalf the signature is executed) with the same force and effect as if the signature page were an original.

23.3 For execution under this clause 23 to be valid the entire Service Agreement upon execution by each individual Party must be delivered to the remaining parties.

Execution

A. The terms of this Service Agreement were agreed under the provisions set out in the *Hospital and* Health *Boards Act,* section 35 on 27 June 2019, and were subsequently amended by the Deed of Amendment entered into pursuant to section 39 of the *Hospital and Health Boards Act 2011* and executed on 24 January 2020; 15 May 2020; 17 June 2020; 25 June 2020 and 12 August 2020.

B. This revised Service Agreement consolidates amendments arising from:

- 2019/20 Amendment Window 2 (in-year variation);
- 2019/20 Amendment Window 3 (in-year variation);
- 2020/21 Amendment Window 1 (annual budget build);
- April 2020 Extra-ordinary Amendment Window; and
- May 2020 Extra-ordinary Amendment Window.

C. Execution source documents are available on the service agreement website https://www.health.qld.gov.au/system-governance/health-system/managing/agreements-deeds.

Schedule 1 HHS Accountabilities

1. Purpose

Without limiting any other obligations of the HHS, this Schedule 1 sets out the key accountabilities that the HHS is required to meet under the terms of this Service Agreement.

2. Registration, credentialing and scope of clinical practice

2.1 The HHS must ensure that:

(a) all persons (including off site reporting radiologists) who provide a clinical service for which there is a national or Queensland legal requirement for registration, have and maintain current registration throughout their employment and only practise within the scope of that registration;

(b) all persons who perform roles for which eligibility for membership of a professional association is a mandatory requirement, have and maintain current eligibility of membership of the relevant professional association throughout their employment in the role; and

(c) all persons who provide a clinical service, and who fall within the scope of current credentialing policies (i.e. including medical, dental, nursing, midwifery and allied health), have a current scope of clinical practice and practise within that scope of clinical practice (which includes practising within their registration conditions and within the scope of the Clinical Services Capability Framework of the facility/s at which the service is provided).

2.2 Confirmation of registration and/or professional memberships is to be undertaken in accordance with the processes outlined in 'Health Professionals Registration: medical officers, nurses, midwives and other health professionals HR Policy B14 (QH-POL-147:2016)', as amended from time to time.

3. Clinical Services Capability Framework

3.1 The HHS must ensure that:

(a) all facilities have undertaken a baseline self-assessment against the Clinical Services Capability Framework (version 3.2);

(b) the Department is notified when a change to the Clinical Services Capability Framework baseline self-assessment occurs through the established public hospital Clinical Services Capability Framework notification process; and

(c) in the event that a Clinical Services Capability Framework module is updated or a new module is introduced, a self-assessment is undertaken against the relevant module and submitted to the Department.

3.2 The HHS is accountable for attesting to the accuracy of the information contained in any Clinical Services Capability Framework self-assessment submitted to the Department.

4. Clinical Prioritisation Criteria

4.1 The HHS must ensure that:

(a) processes for access to specialist surgical and medical services in line with Clinical Prioritisation Criteria are implemented, where these have been developed, in order to improve equity of access to specialist services; and

(b) General Practice Liaison Officer and Business Practice Improvement Officer programs are maintained in order to deliver improved access to specialist outpatient services, including through (but not limited to) their contribution to the development and implementation of Statewide Clinical Prioritisation Criteria.

5. Service delivery

5.1 The HHS will work with collaboratively with other healthcare service providers to ensure that an integrated pathway of care is in place for patients. This will include, but is not limited to:

(a) other HHSs;

(b) Primary Care providers;

(c) non-government organisations; and

(d) private providers.

5.2 The HHS must ensure that:

(a) the Health Services and other outlined in this Service Agreement, for which funding is provided in Schedule 2 ‘Funding and Purchased Activity and Services’ continue to be provided;

(b) the obligations regarding the payment and planning for blood and blood products and best practice as set out under the National Blood Agreement are fulfilled for the facilities for which funding is provided; and

(c) the *Queensland Organ Donation Strategy 2018-2020* is implemented in order to support an increase in organ donation rates in Queensland.

5.3 Through accepting the funding levels defined in Schedule 2 of this Service Agreement, the HHS accepts responsibility for the delivery of the associated programs and reporting requirements to State and Commonwealth bodies as defined by the Department.

6. Accreditation

6.1 All Queensland public hospitals, day procedure services and health care centres (howsoever titled) managed within the framework of HHSs are to maintain accreditation under the Australian Health Service Safety and Quality Accreditation Scheme[1].

6.2 Accreditation will be assessed against the National Safety and Quality Health Service standards[2] (NSQHS) second edition.

6.3 Residential aged care facilities will maintain accreditation by the Aged Care Quality and Safety Commission (ACQSC).

6.4 General practices owned or managed by the HHS are to be externally accredited in accordance with the current edition of the Royal Australian College of General Practitioners (RACGP) accreditation standards and in line with the National General Practice Accreditation Scheme.

6.5 For the purpose of accreditation, the performance of the HHS against the NSQHS and the performance of general practices owned or managed by the HHS against the RACGP accreditation standards can only be assessed by accrediting agencies that are approved by the Australian Commission on Safety and Quality in Healthcare (ACSQHC).

6.6 The HHS will select their accrediting agency from among the approved accrediting agencies. The ACSQHC and the RACGP provide a list of approved accrediting agencies which are published on their respective websites (www.safetyandquality.gov.au and www.racgp.org.au.

6.7 If the HHS does not meet the NSQHS standards accreditation requirements, the HHS has 60 days to address any not met actions. If the HHS does not meet the other accreditation standards requirements (RACGP and ACQSC), a remediation period will be defined by the accrediting agency.

6.8 Following assessment against NSQHS, ACQSC and RACGP standards, the HHS will provide to the Executive Director, Patient Safety and Quality Improvement Service, Department.

(a) immediate advice if a significant patient risk (one where there is a high probability of a substantial and demonstrable adverse impact for patients) is identified during an onsite visit, also identifying the plan of action and timeframe to remedy the issue as negotiated between the surveyors/assessors and/or the respective accrediting agency and the HHS;

(b) a copy of any ‘not met’ reports within two days of receipt of the report by the HHS;

(c) the accreditation report within seven days of receipt of the report by the HHS; and

(d) immediate advice should any action be rated not-met by the accrediting agency following the remediation period of an accreditation event, resulting in the facility or service not being accredited. Responsive regulatory processes may be enacted under clause 7 below.

---

[1] www.safetyandquality.gov.au/our-work/assessment-to-the-nsqhs-standards/australian-health-service-safety-and-quality-accreditation-scheme/
[2] www.safetyandquality.gov.au/our-work/assessment-to-the-nsqhs-standards/

6.9 The award recognising that the facility or service has met the required accreditation standards will be issued by the assessing accrediting agency for the period determined by their respective accreditation scheme.

6.10 The HHS will apply to an approved accrediting agency for a re-accreditation assessment prior to the expiry of their current accreditation period.

6.11 Where the HHS funds non-government organisations to deliver health and human services the HHS will ensure, from the Effective Date of this Service Agreement, that:

(a) within 12 months HHS procurement processes and service agreements with contracted non-government organisations specify the quality accreditation requirements for mental health services as determined by the Department; and

(b) as the quality accreditation requirements for subsequent funded service types are determined by the Department, procurement processes and service agreements with contracted non-government organisations reflect these requirements within 12 months of their formal communication by the Department to HHSs.

7. Responsive regulatory process for accreditation

7.1 A responsive regulatory process is utilised in the following circumstances:

(a) where a significant patient risk is identified by a certified accrediting agency during an accreditation process; and/or

(b) where an HHS has failed to address 'not met' actions of the specified standards within required timeframes.

7.2 An initial regulatory response will begin with a process of verifying the scope, scale and implications of the reported issues, a review of documentation, and may include one or more site visits by nominated specialty experts.

7.3 The regulatory process may include one or a combination of the following actions:

(a) seek further information from the HHS;

(b) request a progress report for the implementation of an action plan;

(c) escalate non-compliance and/or risk to the Performance Review Meeting;

(d) provide advice, information on options or strategies that could be used to address the non-met actions within a designated time frame; and/or

(e) connect the hospital to other hospitals that have addressed similar deficits or have exemplar practice in this area.

7.4 In the case of serious or persistent non-compliance and where required action is not taken by the HHS the response may be escalated. The Department may undertake one or a combination of the following actions:

(a) restrict specified practices/activities in areas/units or services of the HHS where the specified standards have not been met;

(b) suspend particular services at the HHS until the area/s of concern are resolved; and

(c) suspend all service delivery at a facility within an HHS for a period of time.

8. Achieving health equity for First Nations Queenslanders

8.1 The *Queensland Health Statement of Action towards Closing the Gap in Health Outcomes* is a commitment to addressing systemic barriers that may in any way contribute to preventing the achievement of health equity for all First Nations people. The statement is expected to mobilise renewed efforts and prompt new strategies for achieving health equity for First Nations Queenslanders.

8.2 The HHS will develop a Health Equity Strategy (previously referred to as the Closing the Gap Health Plan) to demonstrate the HHS's activities towards achieving health equity for First Nations people. The Health Equity Strategy will supersede the existing Closing the Gap Health Plan and act as the principal accountability mechanism between community and Government in the pursuit of Health Equity for First Nations Queenslanders.

8.3 The Health Equity Strategy will:

(a) be co-designed, co-developed and co-implemented by the First Nations community and the HHS; and

(b) demonstrate an evidence-based approach to priority setting.

8.4 The HHS will review the Health Equity Strategy at least once every three years and will publish the Health Equity Strategy in a way that allows it to be accessed by members of the public.

8.5 In line with the *Queensland Health Statement of Action towards Closing the Gap in Health Outcomes,* the HHS will ensure that commitment and leadership is demonstrated through implementing actions outlined in the Health Equity Strategy. The actions will, at a minimum:

(a) promote and provide opportunities to embed the representation of First Nations people in leadership, governance and the workforce;

(b) improve local engagement and partnerships between the HHS and First Nations people, communities and organisations to enable co-design, co-development and co-implementation;

(c) improve transparency, reporting and accountability in Closing the Gap progress; and

(d) demonstrate co-design, co-development, co-implementation and co-leadership of health programs and strategies.

8.6 The HHS will:

(a) establish and maintain an ongoing dialogue with the Department to progress and report on key actions taken and Health Service initiatives aligned to the *Queensland Health Statement of Action towards Closing the Gap in Health Outcomes*;

(b) establish and maintain an ongoing dialogue with the Department to progress and report on key actions taken and health service initiatives and strategies to attract,

recruit, support and retain a First Nations people workforce and workforce models commensurate to the HHS population and aligned to the benchmarks prescribed in the Workforce Diversity and Inclusion Strategy 2017-2022; and

(c) report publicly on progress against the Health Equity Strategy. Progress will be reported on an annual basis as a minimum.

9. Provision of Clinical Products/Consumables in outpatient settings

9.1 Upon discharge as an inpatient or outpatient, and where products/consumables are provided free of charge or at a subsidised charge, the Treating HHS will bear the initial costs of products/consumables provided to the patient/consumer as part of their care. These costs will be met by the Treating HHS for a sufficient period of time to ensure the patient/consumer incurs no disruption to their access to the Clinical Products/Consumables.

9.2 Unless otherwise determined by the HHS providing the Clinical Products/Consumables, ongoing direct costs (beyond an initial period following discharge as an inpatient) of the provided products/consumables will be borne by the Residential HHS of the outpatient/consumer.

9.3 Where guidelines exist (e.g. Guideline for Compression Garments for Adults with Lymphoedema: Eligibility, Supply and Costing and Guideline for Home Enteral Nutrition Services for Outpatients: Eligibility, Supply and Costing), standardised eligibility criteria and charges should apply.

9.4 Where a patient is supplied with medicines on discharge, or consequent to an outpatient appointment, that are being introduced to a patient's treatment, the Treating HHS will provide prescription(s) and an adequate initial supply. This will comprise:

(a) for medicines reimbursable under the Pharmaceutical Benefits Scheme (PBS), including the Section 100 Highly Specialised Drugs Program – the quantity that has been clinically-appropriately prescribed or the maximum PBS supply, whichever is the lesser; or

(b) for non-reimbursable medicines, one month's supply or a complete course of treatment, whichever is the lesser.

9.5 For medicines that are non-reimbursable under the PBS, and which are not included in the Queensland Health List of Approved Medicines (LAM), the Residential HHS will be responsible for ongoing supply, provided that the Treating HHS has provided the Residential HHS with documentary evidence of the gatekeeping approval at the Treating HHS for the non-LAM medicine. This evidence may be:

(a) a copy of the individual patient approval; or

(b) where the medicine is subject to a 'blanket approval' at the Treating HHS, a copy of the blanket approval, and a statement that the patient meets the criteria to be included under that approval.

9.6 This evidence is to be provided pro-actively to the Director of Pharmacy (or, for non-Pharmacist sites, the Director of Nursing and the HHS Director of Pharmacy) for the hospital nominated under clause 9.8 below.

9.7 For non-reimbursable medicines listed on the LAM for the condition being treated, the Residential HHS is responsible for ongoing supplies.

9.8 The Treating HHS will inform the patient about the ongoing supply arrangements and agree which hospital, within the patient's Residential HHS, they should attend for repeat supplies. The patient will be advised to contact the pharmacy at the nominated hospital regarding their requirements at least a week before attending for repeat supply.

9.9 PBS-reimbursable prescriptions issued by a public hospital may be dispensed at any other public hospital that has the ability to claim reimbursement. Patients may, in accordance with hospital policy, be encouraged to have their PBS prescriptions dispensed at a private pharmacy of their choice.

10. Capital, land, buildings, equipment and maintenance

10.1 **Capital**

(a) The HHS will:

(i) achieve annual capital expenditure within an acceptable variance to its allocation in the State's published Budget Paper 3 – Capital Statement, as specified in the capital expenditure performance KPI target.

(ii) record capital expenditure data in the capital intelligence portal each month. Data will be published through the System Performance Reporting (SPR) platform.

(iii) achieve all Priority Capital and Health Technology Equipment Replacement Program capital expenditure requirements and associated delivery milestones, as funded, and undertake all capital expenditure performance reporting requirements in the capital intelligence portal on a monthly basis.

(iv) comply with all other capital program reporting requirements, as identified in Schedule 4, Table 13.

10.2 **Asset Management**

(a) The Service Agreement includes funding provision for regular maintenance of the HHS's building portfolio.

(b) The Department has determined that a total sustainable budget allocation that equates to a minimum of 2.81% of the un-depreciated asset replacement value of the Queensland Public Health System's building portfolio is required to sustain the building assets to achieve expected life-cycles. The sustainable budget allocation is a combination of operational and capital maintenance funding.

(c) The HHS will conduct a comprehensive assessment of the maintenance demand for the HHS's building portfolio to ascertain the total maintenance funding

requirements of that portfolio. The assessment must identify the following for the portfolio:

(i) regulatory requirements;

(ii) best practice requirements;

(iii) condition-based requirements;

(iv) lifecycle planning requirements; and

(v) reactive maintenance estimates based on historical information, including backlog maintenance liabilities and risk mitigation strategies.

(d) The HHS will allocate an annual maintenance budget that reasonably takes into account the maintenance demand identified by the assessment in its reasonable considerations, without limiting the scope of such reasonable considerations including financial affordability linked to risk assessment. The annual maintenance budget will equate to either:

(i) 2.81% of the un-depreciated asset replacement value of the HHS's building portfolio; or

(ii) an alternative percentage amount determined by the HHSs as a result of its considerations.

(e) The HHS will submit an annual asset management and maintenance plan, approved by the Health Service Chief Executive, to the Department that:

(i) outlines the maintenance demand assessment undertaken by the HHS under Schedule 1, clause 10.2(c)

(ii) confirms the annual maintenance budget determined by the HHS under Schedule 1, clause 10.2(d)

(f) The HHS will submit an annual Statement of Building Portfolio Compliance to the Department for each year of the Term of this Service Agreement.

(g) The HHS will continue to proactively develop and address the recommendations within the final Asset Management Capability Report that was issued to the HHS as part of the transfer notice process.

10.3 **Property**

(a) The HHS will ensure building and infrastructure assets are managed in accordance with the specifications of any relevant transfer notices published as a gazette notice by the Minister under section 273A of the *Hospital and Health Boards Act 2011.*

(b) For land, buildings and parts of buildings where the Department is, or is intended to be, the exclusive occupier under specific occupancy or ground leases implemented pursuant to clauses 1.7 (c) and 1.8 respectively (where applicable) of a transfer notice, the Department is deemed to be in control of that land, building or part of a building for the purpose of work health and safety law.

10.4 Nothing in clause 10.3(b) of Schedule 1:

(a) removes any work health and safety responsibilities shared with another party or parties in accordance with work health and safety law; or

(b) limits the arrangements for the provision of work health and safety services provided in clause 11.

11. Occupational health and safety

11.1 The HHS, whether in the capacity of a Person Conducting a Business or Undertaking as an employer or as a building owner or occupier, will consult, cooperate and coordinate to ensure legal compliance with the *Work Health and Safety Act 2011* and other legislation as it applies to the scope, nature and location of operations associated with this Service Agreement. This includes, but is not limited to:

(a) codes of practice;

(b) electrical safety legislation;

(c) building and fire safety legislation; and

(d) workers' compensation legislation.

11.2 The HHS will establish, implement and maintain a health and safety management system which conforms to recognised health and safety management system standard AS/NZS 4801 *Occupational Health and Safety Management System* or ISO45001 *Occupational Health and Safety Management Systems* or another standard as agreed with the Chief Executive.

11.3 The HHS will monitor health and safety performance and will provide to the Chief Executive reports of a type, and at the intervals, agreed between the Parties, or as reasonably specified by the Chief Executive.

11.4 The Chief Executive will monitor health and safety performance at the system level. Where significant health and safety risks are identified, or performance against targets is identified as being outside tolerable levels, the Chief Executive may request further information from the HHS to address the issue(s) and/or make recommendations for action.

12. Workforce management

12.1 Subject to a delegation by the Chief Executive under section 46 of the *Hospital and Health Boards Act 2011*, the HHS is responsible for the day-to-day management (the HR Management Functions) of the Health Service Employees provided by the Chief Executive to perform work for the HHS under this Service Agreement.

(a) The HHS will exercise its decision-making power in relation to all HR Management Functions which may be delegated to it by the Chief Executive under section 46 of the *Hospital and Health Boards Act 2011*, in respect of the Health Service Employees, in a lawful and reasonable manner and with due diligence, and in accordance with:

(i) terms and conditions of employment specified by the Department in accordance with section 66 of the *Hospital and Health Boards Act 2011;*

(ii) health service directives, issued by the Chief Executive under section 47 of the *Hospital and Health Boards Act 2011*;

(iii) health employment directives, issued by the Chief Executive under section 51A of the *Hospital and Health Boards Act 2011;*

(iv) any policy document that applies to the Health Service Employee;

(v) any Industrial Instrument that applies to the Health Service Employee;

(vi) the relevant HR delegations manual; and

(vii) any other relevant legislation.

12.2 The HHS must ensure that Health Service Employees are suitably qualified to perform their required functions.

12.3 Persons appointed in an HHS as a Health Executive or Senior Health Service Employees are employees of the HHS

12.4 All HHSs will provide to the Chief Executive human resource, workforce, and health and safety reports of a type, and at the intervals, agreed between the Parties, or as reasonably specified by the Chief Executive.

13. Medically authorised ambulance transports

13.1 The HHS will:

(a) utilise the Queensland Ambulance Service (QAS) for all road ambulance services not provided by the HHS. This includes both paramedic level and patient transport level services where the patient requires clinical care;

(b) follow the *Medically Authorised Ambulance Transports Operational Standards* when utilising QAS services; and

(c) ensure that performance data for ambulance services authorised by the HHS is collected and provided to the Department in line with agreed data supply requirements.

Schedule 2 Funding, purchased activity and services

1. Purpose

This Schedule 2 sets out:

(a) The activity purchased by the Department from the HHS (Table 4, Table 6 and Table 8);

(b) The funding provided for delivery of the purchased activity (Table 4; Table 5; Table 6; and Table 7);

(c) Specific funding commitments (Table 1);

(d) The criteria and processes for financial adjustments associated with the delivery of purchased activity and specific funding commitments;

(e) The sources of funding that this Service Agreement is based on and the manner in which these funds will be provided to the HHS (Table 3); and

(f) An overview of the purchased Health Services and other services which the HHS is required to provide throughout the period of this Service Agreement.

2. Delivery of purchased activity

2.1 The Department and the HHS will monitor actual activity against purchased levels.

2.2 The HHS has a responsibility to actively monitor variances from purchased activity levels and will notify the Department immediately via the D-SA Contact Person as soon as the HHS becomes aware of significant variances.

2.3 The HHS will also notify the Department of deliberate changes to the consistent recording of activity within year that would result in additional activity being recorded for existing Health Services.

2.4 If the HHS wishes to move activity between purchased activity types and levels, for example, activity moving from outpatients to inpatients or from one inpatient Service Related Group (SRG) to another, the HHS must negotiate this with the Department based on a sound needs based rationale.

2.5 With the exception of the programs, services and projects that are specified in Table 1, during 2020/21 no financial adjustment will be applied where the HHS is unable to deliver or exceeds the activity that has been funded, in recognition of the Commonwealth Government's treatment of the National Health Reform Agreement to support the response to the COVID-19 pandemic.

2.6 The activity purchased through this Service Agreement for 2020/21 is based on the activity purchased recurrently in 2019/20 and includes the productivity dividend.

2.7 The activity purchased in the Service Agreement for 2021/22 will be based on the activity purchased recurrently in 2020/21 including the productivity dividend.

2.8 Any resulting changes to funding and purchased activity levels will be actioned through the processes outlined in Schedules 2 and 5 of this Service Agreement.

2.9 The Department is required to report HHS activity data to the Independent Hospital Pricing Authority and the Administrator of the National Health Funding Pool. The HHS will maintain its efforts to accurately record the delivered activity and submit this data to the Department in accordance with the requirements set out in Schedule 4.

2.10 The HHS should refer to the supporting document to this Service Agreement 'Healthcare Purchasing Policy and Funding Guidelines 2020/21' for details regarding the calculation of Weighted Activity Units. Supporting documents are available on-line as detailed in Appendix 1.

3. Financial adjustments

3.1 **Specific funding commitments**

(a) As part of the Service Agreement Value, the services, programs and projects set out in Table 1 have been purchased by the Department from the HHS. These services will be the focus of detailed monitoring by the Department.

(b) The HHS will promptly notify the D-SA Contact Person if the HHS forecasts an inability to achieve commitments linked to the specific funding commitments included in Table 1.

(c) On receipt of any notice under clause 3.1(b) of Schedule 2, it is at the discretion of the Chief Executive (or delegate) to withdraw allocated funding pro rata to the level of under delivery if the program is not being delivered according to the program objective or is not being delivered in full.

(d) If the Chief Executive (or delegate) decides to withdraw allocated funding, the Chief Executive (or delegate) will immediately issue an Adjustment Notice to the HHS-SA Contact Person confirming any adjustment that has been made in accordance with this clause 3.1 of Schedule 2. The Adjustment Notice will be issued through the process set out in clause 3.4 or 3.5 of Schedule 5.

(e) Following receipt of an Adjustment Notice under clause 3.1(d) of Schedule 2, the Parties will comply with the Adjustment Notice and immediately take steps necessary to give effect to the requirements of that Adjustment Notice.

(f) The Parties acknowledge that adjustments made under this clause 3.1 of Schedule 2 may vary the Service Agreement Value and/or a specific value recorded in Table 1.

(g) Where the Service Agreement Value and/or a specific value recorded in Table 1 is varied, the variation will be recorded in a Deed of Amendment issued following the next available Amendment Window.

Table 1 Specific Funding Commitments

Service/Program/Project	Funding	Activity	Timeframe	Conditions
Making Tracks towards closing the gap in health outcomes for Indigenous Queenslanders by 2033 - Investment Strategy 2018-21	*$1,720,367* *($1,640,367 +$80,000*)* $1,640,367	*0* 0	*2019/20* 2020/21	The HHS will deliver the initiatives and outcomes outlined in the performance requirements issued by the Aboriginal and Torres Strait Islander Health Division in memorandum C-ECTF-19/5767. Funding may be adjusted and/or unspent funds redirected or recovered where project performance requirements are not met. * Funding of $80,000 deferred from 2018/19 to 2019/20.
North Queensland Aboriginal and Torres Strait Islander Sexually Transmissible Infections Action Plan 2016-2021	*$147,997**	*0*	*2019/20*	The HHS will implement and support the required actions under the *North Queensland Aboriginal and Torres Strait Islander Sexually Transmissible Infections Action Plan 2016-2021* (the Plan) including the delivery of initiatives and outcomes outlined in the 2019/20 funding schedule for sexual health outreach activities. Funding may be adjusted and/or unspent funds redirected or recovered where project performance requirements are not met. *$147,997 transferred from Townsville Hospital and Health Service 2019/20.
Residential aged care facility Support Services (RaSS)	*$570,000* $570,000	*0* 0	*2019/20* 2020/21	The HHS will deliver the initiatives and outcomes as outlined in the RaSS and Hospital in The Home COVID-19 Funding - memorandum C-ECTF 20/4081. The funding allocated is linked to the National Partnership Agreement on COVID-19 healthcare response and as such it will be a requirement of the HHS to capture accurate activity and expenditure of services delivered under this program. Unspent funds may be withdrawn and returned to the department.

Service/Program/Project	Funding	Activity	Timeframe	Conditions
Enterprise Bargaining (EB)	*$9,613,253*<br>$2,932,214<br>$1,916<br>(comprises both recurrent and non-recurrent funding)	*0*<br>0<br>0	*2019/20*<br>2020/21<br>2021/22	Funding has been allocated in full for the following EB agreements:<br>• *Nurses and Midwives (Queensland Health and Department of Education and Training) Certified Agreement (EB10) 2018* (Base wages and certain entitlements)*;* and<br>• *Medical Officers' (Queensland Health Certified Agreement (MOCA5) 2018*.<br>Legislative amendments have been introduced under the *Industrial Relations Act 2016* to give effect to a 2.5% increases under the following agreements as new agreements are yet to be certified:<br>• *Queensland Public Health Sector Certified Agreement (No.9) 2016;*<br>• *Queensland Health, Building, Engineering & Maintenance Services Certified Agreement (No.6) 2016;* and<br>• *Health Practitioners' and Dental Officers (Queensland Health) Certified agreement (No. 2) 2016.*<br>Funding which has been allocated recurrently in previous years has been recalled for the following streams as wage increase are not yet approved:<br>• HES-DSO;<br>• SES-SO; and<br>• VMO.<br>Subject to the terms and conditions of the agreements once executed a funding adjustment may be required. Full details can be found on the Budget and Analysis SharePoint platform.

Service/Program/Project	Funding	Activity	Timeframe	Conditions
Evolve Therapeutic Services (ETS)	*$279,362*	*0*	*2019/20*	Provision of ETS within allocated resources and in line with the state-wide ETS Manual, noting variation in local contexts.<br>Reporting requirements as defined by the Mental Health, Alcohol and Other Drugs Branch.<br>If program performance requirements are not met in-year funding may be adjusted proportional to the under delivery against the agreed target.
Planned Care Volume Targets – Elective Surgery	$25,693,799 (Funding in existing service agreement)	3,648 elective surgery separations aligned with the elective surgery data collection, as reported on SPR and any outsourced elective surgery activity.<br>5,301 WAUs (Q22)	2020/21	Funding has been quarantined to support the ongoing delivery of planned care volumes during 2020/21. Quarantined funds have been determined based on agreed average Q22 WAUs for 3,648 Elective Surgery Separations (volume target).<br>Volume targets to be reviewed and adjusted through the amendment window process to include any additional planned care investments.<br>Performance will be monitored quarterly with reconciliation of the agreed planned care activity targets (number of day case and overnight treated patients).<br>Should the agreed volume targets not be met, funding will be withdrawn at the calculated HHS average Q22 WAUs **per separation not delivered**:<br>Example:<br>1 Case = 1.45 Q22 WAUs or<br>$7,043 (QEP)<br>There will not be claw back of under delivery against Q22 WAUs target if the volume of elective day case and overnight separations has been delivered.

Service/Program/Project	Funding	Activity	Timeframe	Conditions
Planned Care Volume Targets – Gastrointestinal Endoscopy (GIE)	$6,683,078 (Funding in existing service agreement value)	3,300 Gastrointestinal Endoscopies aligned with the Gastrointestinal Endoscopy data collection, as reported on SPR and any outsourced GIE activity. 1,379 WAUs (Q22).	2020/21	Funding has been quarantined to support the ongoing delivery of planned care volumes during 2020/21. Quarantined funds have been determined based on agreed average Q22 WAUs for 3,300 Gastrointestinal Endoscopy Separations (volume target).<br>Volume targets to be reviewed and adjusted through the amendment window process to include any additional planned care investments.<br>Performance will be monitored quarterly with reconciliation of the agreed planned care activity targets (number treated GIE patients).<br>Should the agreed volume targets not be met, funding will be withdrawn at the calculated HHS average Q22 WAUs per separation not delivered:<br>Example:<br>1 Case = 0.42 Q22 WAUs or<br>$2,025 (QEP)<br>There will not be claw back of under delivery against Q22 WAUs target if the volume of GIE day case and overnight separations has been delivered.

Service/Program/Project	Funding	Activity	Timeframe	Conditions
Planned Care Volume Targets – Specialist Outpatients	$5,816,779 (Funding in existing service agreement value)	21,073 Specialist Outpatient initial service events as per the funding specification, and outsourced activity.<br>1,200 WAUs (Q22)	2020/21	Funding has been quarantined to support the ongoing delivery of planned care volumes during 2020/21. Quarantined funds have been determined based on agreed average Q22 WAUs for 21,073 Initial Service Events (volume target).<br>Volume targets to be reviewed and adjusted through the amendment window process to include any additional planned care investments.<br>Performance will be monitored quarterly with reconciliation of the agreed planned care activity targets (number of initial service events).<br>Should the agreed volume targets not be met, funding will be withdrawn at the calculated HHS average Q22 WAUs per initial service event not delivered:<br>Example:<br>1 Case = 0.057 Q22 WAUs or<br>$276 (QEP)<br>There will not be claw back of under delivery against Q22 WAUs target if the volume of initial service events has been delivered.

Service/Program/Project	Funding	Activity	Timeframe	Conditions
BreastScreen	*$1,920,116*<br><br>$1,933,034 (comprises screening activity funding; Incentive funding; fixed allocations – e.g. Biomedical Technology Services, leave of premises and 2.5% non-labour escalation	*9,700 total screens Incentive 1: 1,012 screens Incentive 2: 33 screens*<br><br>8,400 total screens Incentive 1:850 screens Incentive 2: Not accepted by HHS	*2019/20*<br><br>2020/21	Provision of BreastScreen services targeting women aged 50-74 years old (women 40-49 years and 75+ are also eligible, although not actively recruited).<br>Incentive funding:<br>• Incentive 1: $30 per screen for out of hours screens in the target age group 50 to 74, where BreastScreen Queensland (BSQ) Registry appointment time is before 8am on weekdays, 5pm and after on weekdays and all-day weekends; and<br>• Incentive 2: $90 per screen for 1st and 2nd screens in the target age group 50 to 74 above the 2015/16 BSQ Service specific baseline.<br>Note Incentive 1 and Incentive 2 activity is a subset of the total screening activity.<br>Funds may be withdrawn should the HHS not meet their screening target.
Hospital in The Home (HITH)	*$870,000*<br><br>$600,000	*179 WAUs*<br><br>124 WAUs	*2019/20*<br><br>2020/21	The HHS will deliver the initiatives and outcomes as outlined in the Residential Aged Care Facility Support Services and HITH COVID-19 Funding - memorandum C-ECTF 20/4081.<br>The HHS will provide a reconciliation of expenditure through End of Year, and unspent funds may be withdrawn and returned to the department.
Orthopaedic overnight beds	$3,600,000 (recurrent)	743 WAUs (Q22)	2020/21	Funding is provided for the continued operation of 9 orthopaedic overnight inpatient beds opened in 2019/20.<br>If the agreed service levels are not provided, funding may be withdrawn on a pro-rata basis.
Cardiac Catheter Laboratory	$988,915 (recurrent)	180 WAUs (Q22)	2020/21	Funding is provided for the expansion of the Cardiac Catheter lab service to a 24/7 service model.<br>If the agreed service levels are not provided, funding may be withdrawn on a pro-rata basis.

Service/Program/Project	Funding	Activity	Timeframe	Conditions
Nurse Navigators	*$1,788,933*<br>$1,877,591<br>$1,877,591<br>$1,877,591 (recurrent)	*0*<br>0<br>0<br>0	*2019/20*<br>2020/21<br>2021/22<br>2022/23	The total Nurse Navigator Program allocation (2015/16 – 2019/20) is 10 NG7 and 1 NG8. All Nurse Navigator Program FTE is required to be appointed to a position ID that has 'Nurse Navigator' within the position title.<br>The HHS is ineligible to appoint Nurse Navigator Program FTE to any pre-existing permanent positions which have been renamed to include 'Nurse Navigator' in the position title.<br>The HHS is required to report monthly on:<br>• employed Nurse Navigator FTE;<br>• number of Nurse Navigator plans in place; and<br>• number of patients seen by Nurse Navigators.
Another 100 Midwives (Nursing)	*$726,085*<br>$744,885<br>$353,880	*0*<br>0<br>0	*2019/20*<br>2020/21<br>2021/22	The HHS will deliver the initiatives and outcomes outlined in the performance requirements as per memo C-ECTF-18/8074. Funding may be withdrawn if requirements are not met.
High risk foot patients seen/managed within 48 hours of referral to ambulatory services	$309,577 (recurrent)<br><br>$154,788 (recurrent)	*65 WAUs (Q21)*<br><br>*32 WAUs (Q22)*	*2018/19*<br><br>*2019/20*	The HHS will provide services as specified in the 2019/20 Ambulatory High-Risk Foot Services specification sheet published on QHEPS.

Service/Program/Project	Funding	Activity	Timeframe	Conditions
Oral Health Services	$6,718,557 (recurrent)	130,959 WOOS	2020/21	Delivery consistent with the Oral Health Policy Framework.<br>Funding does not include Commonwealth National Partnership Agreement funding.<br>Funding may be adjusted where the total oral health activity delivered varies from the purchased levels.<br>Oral health activity (WOOS) for the 0-15 year age group shall not be less than that achieved in 2017/18.<br>Oral health activity (WOOS) includes activity claimed under the Child Dental Benefits Schedule but excludes dental treatment delivered under general anaesthetic in public hospitals.
• National Partnership Agreement (NPA) on Adult Public Dental Services – First Nations	$46,678	849 WOOS	2020/21	Queensland is required to meet two performance targets during 2020/21, which are the 30 September 2020 target (for 1 April to 30 September 2020) and the 31 March 2021 target (for 1 October 2020 to 31 March 2021). HHSs must collectively meet these targets.<br>Funding may be adjusted where the total oral health activity delivered varies from the purchased levels.
Connecting Care to Recovery 2016-2021: A plan for Queensland's Mental Health				
• Independent Patient Rights Advisers	$178,000 (recurrent)	*0*	*2017/18*	Independent Patient Rights Advisors are to be employed or engaged in accordance with the *Mental Health Act 2016* and the Chief Psychiatrist Policy on Independent Patients' Rights Advisers.
• Adult Step Up Step Down (SUSD)	$369,321 (recurrent)	*0*	*2017/18*	Clinical staffing to support interim SUSD service.
	$582,068 (recurrent)	*0*	*2019/20*	Clinical Staffing to support operation of adult SUSD, including building maintenance.
• Co-located Service Pilot (STARR)	*$270,000*	*0*	*2019/20*	Co-located Service Pilot Program: Support Time and Rehabilitation Recovery continuation of project and evaluation of outcomes.

Service/Program/Project	Funding	Activity	Timeframe	Conditions
Rapid Results Program<br>*Delivering what matters: Advancing Kidney Care 2026 Collaborative*	$706,893 (recurrent)	*72 WAUs (Q22 - part WAU backed)*<br>72 WAUs (Q22 - fully WAU backed)	*2019/20*<br>2020/21	Funding is provided for implementation of a supported home haemodialysis model and a kidney supportive care model under the *Advancing Kidney Care 2026 Collaborative*.<br>The HHS will ensure that the reporting requirements established for this initiative are met, including the provision of quarterly progress against agreed implementation milestones and outcome measures.
Rapid Results Program<br>*Delivering what matters: Frail and Older persons initiative*<br>• Residential Aged Care Facility (RACF) support services (RaSS)<br>• Geriatric Emergency Department Intervention (GEDI)	$500,000 (recurrent)<br>$500,000 (recurrent)	*0*<br>*0*	*2019/20*<br>*2019/20*	Funding is provided to implement the core principals of a RACF Acute Care Support Service and Geriatric Emergency Department Intervention through a hybrid model of care at Mackay Hospital from 1 July 2019.<br>The HHS will:<br>• Establish the service in line with agreed timelines;<br>• Establish a Steering Committee to provide oversight of the service development and operation;<br>• Comply with the agreed reporting requirements, including progress against the identified project outcomes and performance measures; and<br>• Participate in learning sessions and statewide working group meetings.<br>If these conditions are not met or if dedicated Frail Older Persons models of care are ceased, funding may be withdrawn.

Service/Program/Project	Funding	Activity	Timeframe	Conditions
Care in the Right Setting (CaRS):<br>• Better Health North Queensland: Regional Healthcare in the home	*$715,000*	*0*	*2019/20*	Services will be provided consistent with the CaRS application(s).<br>If Service commencement does not align with the agreed implementation timeframes funding may be withdrawn on a pro-rata basis.<br>If the agreed Service levels are not provided, funding may be withdrawn.<br>Activity levels will be monitored regularly and cooperation with external evaluators is required.<br>Where the Service includes Service provision to another (receiving) HHS:<br>• If staffing is not available within the HHS to meet the agreed Service levels, the HHS will make alternate arrangements to ensure that the agreed Service levels are provided; and<br>• If the agreed Service levels are not provided, funding may be withdrawn and provided to the receiving HHS.

3.2 **National Partnership on COVID-19 Response**

(a) The Department will provide additional funding to the HHS under the State Public Health Payment component of the *National Partnership on COVID-19 Response* where actual additional cost is demonstrated.

(b) Funding will be provided where the HHS has:

(i) undertaken activity that is in-scope for the State Public Health Payment during the reporting period; and

(ii) met the reporting requirements in relation to COVID-19 expenditure, as prescribed under the *National Partnership on COVID-19 Response* and by the Department.

(c) The scope of the State Public Health Payment is defined as:

(i) additional costs that are attributable to the treatment of patients with diagnosed or suspected COVID-19; or

(ii) additional costs of activities directed at preventing the spread of COVID-19.

(d) Additional costs that are reimbursed through the State Public Health Payment will be excluded from the calculation of activity eligible for funding under the terms of the National Health Reform Agreement.

(e) There is no guarantee that the HHS will have access to additional funding under the State Public Health Payment.

(f) All funding that is provided through the State Public Health Payment will be subject to a reconciliation to be undertaken by the Administrator of the National Health Funding Pool. The HHS will be required to provide evidence to support claims for additional costs. If the HHS is unable to provide the required evidence on request, funding received may be recalled subject to reconciliation.

(g) Funding adjustments will be actioned through the process set out in clause 3.4 of Schedule 5 of this Service Agreement.

3.3 **Financial adjustments – other**

(a) The purchasing approach includes a range of funding adjustments which aim to incentivise high value care, that is care which delivers the best outcomes at an efficient cost, and dis-incentivise Low Benefit Care. This includes incentive payments for HHS who achieve quality targets in specific areas of priority. The purchasing incentives that apply to this Service Agreement are detailed in Table 2.

(b) The Department must reconcile the applicable purchasing incentives in Table 2 in line with the timeframes specified in the purchasing specification sheet included within the supporting document 'Purchasing Policy and Funding Guidelines 2020/21'. The Department must promptly provide a copy of the reconciliation statement to the HHS-SA Contact Person.

(c) Funding adjustments must be based on the requirements contained in the relevant specification sheet for that purchasing incentive.

(d) If the Parties are unable to reach agreement in relation to any funding adjustments that are identified, the provisions of clause 14 in the standard terms of this Service Agreement will apply to resolve the dispute.

(e) When the Parties have agreed on a funding adjustment, the Chief Executive (or delegate) will issue an Adjustment Notice to the HHS-SA Contact Person confirming any funding adjustment that has been made in accordance with this clause 3.3 of Schedule 2. The Adjustment Notice will be issued through the process set out in clause 3.4(c) of Schedule 5.

(f) Following receipt of an Adjustment Notice under clause 3.4(c) of Schedule 5, the Parties will comply with the Adjustment Notice and immediately take the steps necessary to give effect to the requirements of that Adjustment Notice.

(g) The Parties acknowledge that the funding adjustments may vary the Service Agreement Value recorded in Schedule 2. Where the Service Agreement Value recorded in Schedule 2 is varied, the variation will be recorded in a Deed of Amendment issued following the next available Amendment Window.

Table 2 Purchasing Incentives 2020/21 (Summary)

Incentive	Description	Scope	Status for 2020/21	Funding Adjustment
Quality Improvement Payment (QIP) – Antenatal Visits for First Nations Women	Incentive payments for achieving targets for:<br>• First Nations women attending an antenatal session during their first trimester, and attending at least 5 antenatal visits; and<br>• First Nations women stopping smoking	All HHSs (excluding Children's Health Queensland)	Continues as per 2019/20 with new targets	50% advance payments made to HHSs with balance paid retrospectively based on performance.
Quality Improvement Payment (QIP) - Smoking Cessation (Community Mental Health)	Incentive payments for achieving targets for community mental health patients clinically supported onto the Smoking Cessation Clinical Pathway	All HHSs (excluding Children's Health Queensland and Mater Public Hospitals)	Continues as per 2019/20 with new targets	Paid retrospectively
High Cost Home Support Program	Payment for high cost 24 hour home ventilated patients meeting the eligibility criteria, where funding is not available through existing sources	All HHSs	Continues as per 2019/20	Paid retrospectively based on forecast costs
Telehealth	Incentive payments for additional outpatient activity volume, provision of telehealth consultancy for Inpatients, Emergency Department and Outpatients episodes and Store and Forward assessments	Inpatients, Emergency Department, Outpatients, and Store and Forward - all HHSs	Continues as per 2019/20 with Outpatients scope expanded to include rural and remote facilities across all HHSs	Paid retrospectively
Sentinel Events	Zero payment for national sentinel events	All ABF public hospitals	Continues as per 2019/20	Retrospective adjustment

3.4 Public and private activity/Own Source Revenue

(a) Own Source Revenue comprises Grants and Contributions, User Charges and Other Revenues.

(b) Where an HHS is above its Own Source Revenue target in respect of private patients, it will be able to retain the additional Own Source Revenue with no compensating adjustments to funding from other sources.

(c) Conversely where an HHS is below its Own Source Revenue target in respect of private patients, it will experience a reduction in revenue with no compensating adjustments to funding from other sources.

(d) The HHS will make all efforts to identify and maximise revenue from appropriate third parties to ensure optimisation of available State and Commonwealth funding across the Queensland Public Sector Health System.

(e) The Own Source Revenue identified in Table 3 is an estimate generated by the HHS which allows all third-party funding sources associated with service delivery

to be identified. The HHS will ensure that this estimate is substantiated and accurate to ensure no significant variances to actual revenue generated.

(f) The HHS will routinely revise and update the estimate to ensure alignment between the Service Agreement and Queensland Treasury's reporting system (TRIDATA).

(g) Budget adjustments for changes in Own Source Revenue from private patients will be actioned through the process set out in Schedule 5 of this Service Agreement.

4. Funding sources

4.1 The four main funding sources contributing to the HHS Service Agreement value are:

(a) Commonwealth funding;

(b) State funding;

(c) Grants and Contributions; and

(d) Own Source Revenue.

4.2 Table 3 provides a summary of the funding sources for the HHS and mirrors the total value of the Service Agreement included in Table 4.

Table 3 Hospital and Health Service funding sources 2020/21

Funding Source	Value ($)
**NHRA Funding**	
Activity Based Funding	331,785,976
Clinical Education and Training[3]	-14,369,046
Own Source Revenue contribution in ABF funded services	-24,654,412
**Pool Account – ABF Funding (State and Commonwealth)[4]**	**292,762,518**
Block Funding	68,920,499
Clinical Education and Training[3]	14,369,046
**State Managed Fund – Block Funding (State and Commonwealth)[5]**	**83,289,545**
**Locally Receipted Funds (Including Grants)**	**14,093,646**
**Locally Receipted Own Source Revenue (ABF)**	**24,654,412**
**Locally Receipted Own Source Revenue (Other activities)**	**11,410,111**
**Department of Health Funding[6]**	**57,393,931**
**Total NHRA Funding**	**483,604,163**
**NPA Covid-19 Response**	
Activity Based Funding	-
**Hospital Services Payment – ABF Funding (State and Commonwealth)[7]**	**-**
Block Funding	-
Clinical Education and Training[3]	-
**Hospital Services Payment – Block Funding (State and Commonwealth)[5]**	**-**
**Public Health Funding (State and Commonwealth) [8]**	**570,000**
**Total NPA – COVID-19 Funding**	**570,000**
**TOTAL**	**484,174,163**

[3] Clinical Education and Training (CET) is classified as Teaching, Training and Research Funding under the National Model and funded as a Block Funded Service. Under the State Model, CET is included as 'Other ABF' and forms part of the ABF total. To comply with the requirements of the National Health Reform Agreement, funding must be paid as it is received, therefore from a Funding Source perspective, CET has been reclassified to Block Funding.

[4] Pool Account - ABF Funding (State and Commonwealth) includes: Inpatient; Critical Care; Emergency Department; Sub and Non Acute; Mental Health; and Outpatient activities each allocated a proportion of Other ABF Adjustments.

[5] State Managed Fund - Block Funding (State and Commonwealth) includes: block funded hospitals; standalone specialist mental health hospitals; community mental health; and teaching, training and research.

[6] Department of Health Funding represents funding by the Department for items not covered by the National Health Reform Agreement including such items as: Prevention, Promotion and Protection; Depreciation, and other Health Services.

7 Hospital Services Payment - Funding provided under the COVID-19 National Partnership Agreement for activity that is attributable to the diagnosis and treatment of Medicare eligible patients with COVID-19 or suspected of having COVID-19; elective surgeries that have been rescheduled to free up public hospital capacity to respond to the COVID-19 outbreak; and may include activities related to the care of public patients being treated in private hospitals.

[8] Public Health Payment - Funding provided under the COVID-19 National Partnership Agreement for the State public health system's activity attributable to the treatment of patients with diagnosed or suspected COVID-19 or for activities directed at preventing the spread of COVID-19.

5. Funds disbursement

5.1 The Chief Executive will direct the disbursement of both State and Commonwealth funding from the State's National Health Funding Pool Sub Account and the State Managed Fund to the HHS. The Service Agreement and State level block payments to State managed funds from Commonwealth payments into the national funding pool are stated in Table 8.

5.2 However, the State (represented by the Chief Executive) will not:

(a) redirect Commonwealth payments between HHSs;

(b) redirect Commonwealth payments between funding streams (e.g. from ABF to Block Funding); and/or

(c) adjust the payment calculations underpinning the Commonwealth's funding.

5.3 Payment of Activity Based Funding and Block Funding to the HHS will be on a fortnightly basis.

5.4 Further information on the disbursement of funds is available in the supporting document to this Service Agreement 'Purchasing Policy and Funding Guidelines 2020/21'.

Table 4 HHS Finance and Activity Schedule 2019/20 – 2021/22 – Summary by Purchasing Hierarchy

	ABF/NABF	ABF_SPLIT	Service Stream	2019/20 QWAU (Q22)	2019/20 Funding ($) (Price: $4847)	2020/21 QWAU (Q22)	2020/21 Funding) (Price: $4847)	2021/22 QWAU (QTBC)	2021/22 Funding (Price: $TBC)
Allocations excluding COVID-19	ABF	ABF	Inpatients	35,766	$158,402,265	36,771	$163,611,190		
			Outpatients	8,901	$43,328,338	9,026	$41,943,638		
			Procedures & Interventions	5,170	$26,092,514	5,283	$26,681,099		
			Emergency Department	9,254	$41,061,418	9,286	$41,291,185		
			Sub & Non-Acute	2,492	$11,328,108	2,504	$11,383,372		
			Mental Health	3,361	$15,287,879	3,378	$15,689,955		
			Prevention & Primary Care	1,713	$8,634,917	1,698	$8,697,879		
			Other ABF $	0	$6,387,087	0	$6,471,071		
		**ABF Total**		**66,657**	**$310,522,526**	**67,946**	**$315,769,389**		
		ABF Other	CET Funding	0	$13,272,977	0	$14,357,690		
			Specified Grants	0	$1,653,022	0	$1,658,897		
			PPP	0	$0	0	$0		
			EB Quarantined	0	$2,776,385	0	$0		
		**ABF Other Total**		**0**	**$17,702,385**	**0**	**$16,016,587**		
	Other Funding	Other Funding	Block Funded Services	7,531	$53,688,409	7,531	$55,549,588		
			Population Based Community Services	0	$34,859,288	0	$34,830,944		
			Other Specific Funding	0	$61,968,680	0	$59,311,275		
			PY Services moved to ABF	0	$0	0	$0		
			Prevention Services – Public Health	0	$2,191,963	0	$2,126,380		
		**Other Funding Total**		**7,531**	**$152,708,340**	**7,531**	**$151,818,187**		
	**Allocations excluding COVID-19 TOTAL**			**74,189**	**$480,933,250**	**75,477**	**$483,604,163**		

	ABF/NABF	ABF_SPLIT	Service Stream	2019/20 QWAU (Q22)	2019/20 Funding ($) (Price: $4847)	2020/21 QWAU (Q22)	2020/21 Funding) (Price: $4847)	2021/22 QWAU (QTBC)	2021/22 Funding (Price: $TBC)
COVID-19 related allocations	ABF	ABF	Inpatients	274	$1,770,000	-0	$0		
			Outpatients	0	$500,000	0	$0		
			Procedures & Interventions	0	$0	0	$0		
			Emergency Department	0	$0	0	$0		
			Sub & Non-Acute	0	$0	0	$0		
			Mental Health	0	$0	0	$0		
			Prevention & Primary Care	0	$0	0	$0		
			Other ABF $	0	$0	0	$0		
		**ABF Total**		**274**	**$2,270,000**	**-0**	**$0**		
		ABF Other	CET Funding	0	$0	0	$0		
			Specified Grants	0	$0	0	$0		
			PPP	0	$0	0	$0		
			EB Quarantined	0	$0	0	$0		
		**ABF Other Total**		**0**	**$0**	**0**	**$0**		
	Other Funding	Other Funding	Block Funded Services	0	$570,000	0	$570,000		
			Population Based Community Services	0	$0	0	$0		
			Other Specific Funding	0	$1,131,354	0	$0		
			PY Services moved to ABF	0	$0	0	$0		
			Prevention Services – Public Health	0	$0	0	$0		
		**Other Funding Total**		**0**	**$1,701,354**	**0**	**$570,000**		
	**COVID-19 Allocations TOTAL**			**274**	**$3,971,354**	**-0**	**$570,000**		
**Grand Total**				**74,463**	**$484,904,604**	**75,477**	**$484,174,163**		

Table 5 Minor Capital and Equity

	2019/20 $	2020/21 $	2021/22 $
**Minor Capital & Equity**			
**Cash**			
SA 16-17.326 - Minor Capital funding Allocation 2016-17	$1,489,000	$1,489,000	
MAC-AW2-Oct17-21 NTFEP - eyeConnect	$0	$0	
MAC-AW2-OCT19-04 Breastscreen Van for Mackay	$750,000	$0	
MAC-AW2-OCT19-36 Capital funding for a Poweredge T640 Server	$8,670	$0	
MAC-AW3-FEB20-06 Lease funding swap per changes to AASB16 - equity component	$525,725	$0	
MAC-BB2021-36 Lease funding swap per changes to AASB16 - equity component	$0	$230,183	
**Non-Cash**			
-	-	-	
**Grand Total**	**$2,773,395**	**$1,719,183**	

Table 6 HHS Finance and Activity Schedule 2019/20 – 2021/22 Other Funding Detail

Agreement		Service Stream	Category	2019/20 $	2020/21 $	2021/22 $
Allocations excluding COVID-19	Other Funding	Block Funded Services	Block Funded Services	$53,688,409	$55,549,588	
		Block Funded Services Total		$53,688,409	$55,549,588	
		Population Based Community Services	Alcohol, Tobacco & Other Drugs	$2,873,644	$2,953,483	
			Community Care Programs	$391,055	$395,203	
			Community Mental Health	$26,306,414	$26,249,635	
			Community Mental Health – Child & Youth	$4,172,645	$4,002,147	
			Other Community Services	$14,674,035	$14,757,637	
			Other Funding Subsidy/(Contribution)	-$17,596,709	-$17,596,709	
			Primary Health Care	$4,038,203	$4,069,548	
		Population Based Community Services Total		$34,859,288	$34,830,944	
		Other Specific Funding	Aged Care Assessment Program	$606,754	$455,066	
			Commercial Activities	$1,218,734	$1,218,734	
			Consumer Information Services	$10,662	$10,662	
			Depreciation	$29,385,000	$28,261,000	
			Disability Residential Care Services	$1,056	$0	
			Environmental Health	$1,838	$8,099	
			Home & Community Care (HACC) Program	$3,655,957	$3,655,957	
			Home & Community Medical Aids & Appliances	$8,773	$8,773	
			Home Care Packages	$349,424	$349,424	
			Interstate Patients	$276,878	$276,878	
			Multi-Purpose Health Services	$2,657,336	$2,657,336	
			Prisoner Health Services	$0	$0	
			Oral Health	$0	$0	
			Patient Transport	$9,711,992	$9,711,992	
			Research	$78,418	$78,418	
			Residential Aged Care	$0	$0	
			Specific Allocations	$12,339,911	$10,952,989	
			State-Wide Functions	$684,110	$684,110	
			Transition Care	$981,836	$981,836	
		Other Specific Funding Total		$61,968,680	$59,311,275	
		Prevention Services – Public Health	Environmental Health (PH)	$586,588	$596,675	
			Other Community Services (PH)	$1,605,375	$1,529,705	

Agreement		Service Stream	Category	2019/20 $	2020/21 $	2021/22 $
		**Prevention Services – Public Health Total**		**$2,191,963**	**$2,126,380**	
	**Allocations excluding COVID-19 Other Funding Total**			**$152,708,340**	**$151,818,187**	
**COVID-19 related allocations**	**Other Funding**	Block Funded Services	Block Funded Services	$570,000	$570,000	
		**Block Funded Services Total**		**$570,000**	**$570,000**	
		Population Based Community Services	Alcohol, Tobacco & Other Drugs	$0	$0	
			Community Care Programs	$0	$0	
			Community Mental Health	$0	$0	
			Community Mental Health – Child & Youth	$0	$0	
			Other Community Services	$0	$0	
			Other Funding Subsidy/(Contribution)	$0	$0	
			Primary Health Care	$0	$0	
		**Population Based Community Services Total**		**$0**	**$0**	
		Other Specific Funding	Aged Care Assessment Program	$0	$0	
			Commercial Activities	$0	$0	
			Consumer Information Services	$0	$0	
			Depreciation	$0	$0	
			Disability Residential Care Services	$0	$0	
			Environmental Health	$0	$0	
			Home & Community Care (HACC) Program	$0	$0	
			Home & Community Medical Aids & Appliances	$0	$0	
			Home Care Packages	$0	$0	
			Interstate Patients	$0	$0	
			Multi-Purpose Health Services	$0	$0	
			Prisoner Health Services	$0	$0	
			Oral Health	$0	$0	
			Patient Transport	$0	$0	
			Research	$0	$0	
			Residential Aged Care	$0	$0	
			Specific Allocations	$1,131,354	$0	
			State-Wide Functions	$0	$0	
			Transition Care	$0	$0	
		**Other Specific Funding Total**		**$1,131,354**	**$0**	
			Environmental Health (PH)	$0	$0	

Agreement		Service Stream	Category	2019/20 $	2020/21 $	2021/22 $
		Prevention Services – Public Health	Other Community Services (PH)	$0	$0	
		**Prevention Services – Public Health Total**		**$0**	**$0**	
	**COVID-19 Allocations Other Funding Total**			**$1,701,354**	**$570,000**	
**Grand Total**				**$154,409,694**	**$152,388,187**	

Table 7 Specified Grants

Program	2019/20 $	2020/21 $	2021/22 $
High Cost Outliers	$1,309,625	$1,318,134	
Limited Indication Medication Scheme	$249,965	$251,589	
18-19 Purch Initiatives (Final reconciliation) - Rewards	$93,432	$0	
20-21 QIP - Antenatal care for Indigenous women	$0	$89,174	
**Grand Total**	**$1,653,022**	**$1,658,897**	

Table 8 Hospital and Health Service Service Agreement and State Level Block Payments to state managed funds from Commonwealth payments into national funding pool

**State:**	QLD	**Service agreement for financial year:**	2020/21
**HHS**	Mackay	**Version for financial year:**	
**HHS ID**		**Version effective for payments from:**	
		**Version status:**	07.07.2020

HHS ABF payment requirements:

Expected National Weighted Activity Unit (NWAU)		National efficient price (NEP) (as set by IHPA)
**ABF Service group**	**Projected NWAU – N2021 (Draft)**	
Admitted acute public services	33,644	$5,320
Admitted acute private services	3,891	$5,320
Emergency department services	9,097	$5,320
Non-admitted services	6,231	$5,320
Mental health services	2,823	$5,320
Sub-acute services	2,327	$5,320
**LHN ABF Total – excluding COVID-19**	**58,012**	**$5,320**
**LHN ABF Total – COVID-19 NPA**	**0**	**$5,320**

Note:
NWAU estimates do not take account of cross-border activity

Reporting requirements by HHS - total block funding paid (including Commonwealth) per HHS, as set out in Service Agreement:

Amount (Commonwealth and State) for each amount of block funding from state managed fund to LHN:	
**Block funding component**	**Estimated Commonwealth and state block funding contribution (ex GST)**
Block funded hospitals	$52,190,614
Community mental health services	$16,729,886
Teaching, Training and Research	$14,369,046
Home ventilation	$0
Other block funded services	$0
**Total block funding for LHN – excluding COVID-19**	**$83,289,545**
**Total funding for LHN under COVID-19 NPA State Public Health Payment**	**$570,000**

6. Purchased services

6.1 State-funded Outreach Services

(a) The HHS forms part of a referral network with other HHSs. Where state-funded Outreach Services are currently provided the HHS will deliver these Health Services in line with the following principles:

(i) historical agreements for the provision of Outreach Services will continue as agreed between HHSs;

(ii) funding will remain part of the providing HHS's funding base;

(iii) activity should be recorded at the HHS where the Health Service is being provided; and

(iv) the Department will purchase outreach activity based on the utilisation of the Activity Based Funding (ABF) price when Outreach Services are delivered in an ABF facility.

(b) Where new or expanded state-funded Outreach Services are developed the following principles will apply:

(i) the Department will purchase outreach activity based on the utilisation of the ABF price when Outreach Services are delivered in an ABF facility;

(ii) agreements between HHSs to purchase Outreach Services will be based on a cost recovery model, which will ensure providing sites are not financially disadvantaged and annual increases will be consistent with the ABF model;

(iii) any proposed expansion or commencement of Outreach Services will be negotiated between HHSs;

(iv) the HHS is able to purchase the Outreach Service from the most appropriate provider including private providers or other HHSs. However, when a change to existing Health Services is proposed, a transition period of at least 12 months will apply during which time the HHS will be required to continue to purchase Outreach Services from the HHS currently providing the Health Service;

(v) any changes to existing levels of Outreach Services need to be agreed to by both HHSs and any proposed realignment of funding should be communicated to the Department to ensure that any necessary funding changes are actioned as part of the Service Agreement amendment process and/or the annual negotiation of the Service Agreement Value; and

(vi) the activity should be recorded at the HHS where the Health Service is being provided.

(c) In the event of a disagreement regarding the continued provision of state-funded Outreach Services:

(i) any proposed cessation of Outreach Services will be negotiated between HHSs to mitigate any potential disadvantage or risks to either HHS; and

(ii) redistribution of funding will be agreed between the HHSs and communicated to the Department to action through the Service Agreement amendment processes outlined in Schedule 5 of this Service Agreement.

6.2 **Telehealth services**

(a) The HHS will support implementation of the Department Telehealth program, including the Telehealth Emergency Support Service. The HHS will collaborate with the Department, other HHSs, relevant non-government organisations and Primary Care stakeholders to contribute to an expanded network of Telehealth services to better enable a program of scheduled and unscheduled care.

(b) The HHS will ensure dedicated Telehealth Coordinators progress the Telehealth agenda locally, driving stakeholder engagement, adoption, planning and implementation activities that will support and grow Telehealth enabled services through substitution of existing face to face services and identification of new Telehealth enabled models of care.

(c) The HHS will ensure the Medical Telehealth Lead will collaborate with the network of HHS based Telehealth Coordinators and the Telehealth Support Unit to assist in driving promotion and adoption of Telehealth across the State through intra and cross-HHS clinician led engagement and change management initiatives as well as informing the development and implementation of clinical protocols and new Telehealth enabled models of care.

6.3 **Newborn hearing screening**

(a) In line with the National Framework for Neonatal Hearing Screening the HHS will:

(i) provide newborn hearing screening in all birthing hospitals and screening facilities; and

(ii) provide where applicable, co-ordination, diagnostic audiology, family support, and childhood hearing clinic services which meet the existing screening, audiology and medical protocols available from the Healthy Hearing website.

6.4 **Statewide Services**

This clause does not apply to this HHS.

6.5 **Statewide and highly specialised clinical services**

The HHS will:

(a) participate in and contribute to the staged review of the purchasing model for identified Statewide and highly specialised clinical services; and

(b) collaborate with the Department and other HHSs in the development of Statewide Services Descriptions through the implementation of the Statewide Services Governance and Risk Management Framework. The Statewide Services Governance and Risk Management Framework guides the Department and HHSs in the strategic management, oversight and delivery of Statewide Services in order to optimise clinical safety and quality and ensure sustainability of services across Queensland.

6.6 **Regional Services**

This clause does not apply to this HHS.

6.7 **Rural and remote clinical support**

This clause does not apply to this HHS.

6.8 **Prevention Services, Primary Care and Community Health Services**

(a) The following funding arrangements will apply to the Prevention, Primary Care and Community Health Services delivered by the HHS:

(i) Department funding for Community Health Services. A pool of funding for these services is allocated to each HHS for a range of Community Health Services and must be used to meet local Primary Care and community healthcare and prevention needs including through delivery of the services identified in Table 6 and HHSs have the discretion to allocate funding across Primary Care and Community Health Services and Prevention Services according to local priorities.

(ii) Department specified funding models for consumer information services, disability, residential care, environmental health, prisoner health services, home and community medical aids, Primary Care, community mental health services, and alcohol and other drugs services. The funding specified for these programs is listed in Table 6 and Department Community Health Service grants.

(iii) Funding from other state government departments and the Commonwealth for specific programs (third party funded services).

(b) **Prevention Services**

The HHS will provide Prevention Services in line with public health related legislation and the service delivery and reporting requirements outlined in the *Public Health Practice Manual*, including:

(i) **Specialist Public Health Units**

The HHS will:

(A) provide a specialist communicable disease epidemiology and surveillance, disease prevention and control service;

(B) maintain and improve, using a public health approach, the surveillance, prevention and control of notifiable conditions, including the prevention and control of invasive and exotic mosquitos, in accordance with national and/or State guidelines and ensure clinical and provisional notification of specified notifiable conditions are reported in accordance with the *Public Health Act* and Public Health Regulations;

(C) provide a specialist environmental health service, which includes assessment and coordination of local responses to local environmental health risks;

(D) undertake regulatory monitoring, investigation, enforcement and

compliance activity on behalf of the Department; and

(E) utilise specialist public health units to support the HHS through provision of advice on prevention strategies and evidence.

(ii) **Preventive health services**

The HHS will:

(A) maintain delivery of risk factor prevention and early intervention programs and services targeting nutrition, physical activity, alcohol consumption, tobacco use, overweight and obesity and falls prevention;

(B) maintain delivery of the school-based youth nursing program throughout Queensland secondary schools; and

(C) promote brief interventions, lifestyle modification programs and other prevention, promotion or early intervention activities.

(iii) **Immunisation services**

The HHS will maintain or improve existing immunisation coverage through continuation of current immunisation services including:

(A) national immunisation program;

(B) opportunistic immunisation in healthcare facilities;

(C) special immunisation programs; and

(D) delivery of the annual school immunisation program in accordance with the Guideline for Immunisation Services (QH-GDL-955:2014).

(iv) **Sexually transmissible infections including HIV and viral hepatitis**

The HHS will maintain and improve, using a public health approach, the prevention, testing, treatment and contact tracing of blood borne viruses and sexually transmissible infections with a continued focus on relevant identified target populations such as First Nations people and culturally and linguistically diverse populations through Services including, but not limited to:

(A) public health units;

(B) sexual health services;

(C) infectious diseases services;

(D) viral hepatitis services;

(E) syphilis surveillance services;

(F) needle and syringe programs; and

(G) existing clinical outreach and support programs in place between HHS.

(v) **Tuberculosis services**

The HHS will ensure there is no financial barrier for any person to tuberculosis diagnostic and management services and will ensure that Services are available in accordance with the Tuberculosis Control health service directive (qh-hsd-040:2018) and Protocol (qh-hsdptl-040:2018)

(vi) **Public Health Events of State Significance**

The HHS will comply with the *Declaration and Management of a Public Health Event of State Significance* health service directive (qh-hsd-046:2014).

(vii) **Cancer screening services**

The HHS will:

(A) increase cervical screening rates for women in rural and remote areas and refer clients to relevant preventive health programs such as BreastScreen Queensland, QUIT line, Get Healthy and My Health for Life by maintaining the existing Mobile Women's Health Service;

(B) provide the Department with an annual report detailing the services provided by the Mobile Women's Health Service:

- across Mackay HHS excluding Collinsville and Bowen; and
- across Central Queensland HHS – Tieri only;

(C) ensure that all cervical screening services provided by the HHS are delivered in accordance with the National Competencies for Cervical Screening Providers and national cervical screening policy documents; and

(D) provide timely, appropriate, high quality and safe follow-up diagnostic services within the HHS for National Cervical Screening Program participants in accordance with the *National Cervical Screening Program Guidelines for the Management of Screen-detected Abnormalities, Screening in Specific Populations and Investigation of Abnormal Vaginal Bleeding* (2017) and national cervical screening policy documents.

(E) develop, implement and evaluate a plan to increase participation in bowel cancer screening and provide the Department with an evaluation report at the end of year 2021/22; and

(F) provide timely, appropriate, high quality and safe diagnostic assessment services for National Bowel Cancer Screening Program participants in accordance with the National Health and Medical Research Council's *Clinical Guideline for Prevention, Early Detection and Management of Colorectal Cancer* (2017). Services to be provided:

- across Mackay HHS.

(G) develop and implement a local service management plan to increase participation in and guide the delivery of accessible breast screening for women in the target age group (50-74 years) through a BreastScreen Australia accredited service. The screening and assessment services should be delivered in accordance with the BreastScreen Queensland (BSQ) Quality Standards Protocols and Procedures Manual, BreastScreen Australia National Accreditation Standards and national policies. Services to be provided:

- across Mackay HHS excluding for Bowen and Collinsville SA2s;

(H) allow the use of the HHS BSQ mobile asset by other HHSs during periods where practical to maximise utilisation of BSQ mobile fleet;

(I) schedule screening services through provision of BSQ mobile vans to increase accessibility for women living in rural and remote areas. While screening schedules are ideally finalised by HHSs six months in advance, confirmation of mobile sites is required by BreastScreen Queensland Registry eight weeks prior to commencement at each site to ensure invitations for screening are prepared and distributed to women in the catchment area; and

(J) The HHS will develop and implement infrastructure plans to manage asset lifecycle performance and replacement schedules including mobile vans. The repair and maintenance services for the BSQ mobile service fleet will be managed and administered by the Mobile Dental Clinic Workshop in Metro South HHS. The HHS will notify the Mobile Dental Clinic Workshop of any repair and maintenance issues and liaise with the Mobile Dental Clinic Workshop to arrange scheduled servicing. The Mobile Dental Clinic Workshop will meet the costs for these services subject to availability of allocated funding for this purpose in any given financial year.

6.9 **Oral health services**

The HHS will ensure that:

(a) oral health services are provided to the Eligible Population at no cost to the patient[9] and that the current range of clinical services will continue;

(b) oral health services fulfil the relevant obligations related to Commonwealth Government dental funding program/s;

[9] The HHS may provide oral health services on a fee-for-service basis to non-eligible patients in rural and remote areas where private dental services are not available.

(c) service delivery is consistent with Queensland Health's oral health policy framework; and

(d) the repair, maintenance and relocation services to the mobile dental fleet continues to be provided by the Mobile Dental Clinic Workshop in Metro South HHS.

6.10 Prisoner health services

This clause does not apply to this HHS.

6.11 Refugee health

This clause does not apply to this HHS.

6.12 Adult sexual health clinical forensic examinations

(a) The HHS will:

(i) provide 24 hour access to clinical forensic examinations for adult victims of sexual assault who present at a public hospital; and

(ii) provide the Department with a quarterly report on the number of examinations provided.

(b) The Service provided will be consistent with the principles of the Queensland Government inter-agency guidelines for responding to people who have experienced sexual assault and any standards issued pursuant to a Health Service Directive.

7. Teaching training and research

The HHS will provide the teaching, training and research programs for which funding is provided within Schedule 2 of this Service Agreement and as described below:

7.1 Clinical education and training

(a) The HHS will:

(i) continue to support and align with the current Student Placement Deed Framework which governs clinical placements from relevant tertiary education providers in Queensland HHS facilities;

(ii) comply with the obligations and responsibilities of Queensland Health under the Student Placement Deed, as appropriate, as operator of the facility at which the student placement is taking place;

(iii) comply with the terms and conditions of students from Australian education providers participating in the Student Placement Deed Framework;

(iv) only accept clinical placements of students from Australian education provides participating in the Student Placement Deed Framework;

(v) continue to provide training placements consistent with and proportionate to the capacity of the HHS. This includes, but is not limited to, planning and resourcing for clinical placement offers in collaboration with other

HHSs and the Department, and the provision of placements for the following professional groups relevant to the HHS:

(A) medical students

(B) nursing and midwifery students

(C) pre-entry clinical allied health students

(D) interns

(E) rural generalist trainees

(F) vocational medical trainees

(G) first year nurses and midwives

(H) re-entry to professional register nursing and midwifery candidates

(I) dental students

(J) allied health rural generalist training positions

(K) Aboriginal and Torres Strait Islander Health Workers and Aboriginal and Torres Strait Islander Health Practitioners

(vi) participate in vocational medical rotational training schemes, facilitate the movement of vocational trainees between HHSs and work collaboratively across HHSs to support education and training program outcomes;

(vii) report, at the intervals and in the format agreed between the Parties, to the Department on the pre-entry clinical placements provided under the Student Placement Deed Framework;

(viii) comply with the state-wide vocational medical training pathway models including:

(A) The Queensland Basic Physician Training Network;

(B) The Queensland General Medicine Advanced Training Network;

(C) The Queensland Intensive Care Training Pathway;

(D) The Queensland Basic Paediatric Training Network;

(E) The Queensland General Paediatric Advanced Training Network; and

(F) The Queensland Neonatal and Perinatal Medicine Advanced Training Network;

(ix) support the provision of placements by the Queensland Physiotherapy Placement Collaborative for physiotherapy pre-entry students via the Physiotherapy Pre-registration Clinical Placement Agreement; and

(x) provide clinical area placements for dietetics pre-entry students from additional funding provided through relevant agreements with Universities.

(b) In addition, the Health Practitioners and Dental Officers (Queensland Health) Certified Agreement (No 2) 2016 (the HP agreement) requires Hospital and Health Services to:

(i) continue to support development of allied health research capacity through continued implementation and retention of health practitioner research positions provided through the HP agreement; and

(ii) support development of allied health clinical education capacity through continued implementation and retention of clinical educator positions provided through the HP agreement, continuing to provide allied health pre-entry clinical placements and maintaining support for allied health HP 3 to 4 rural development pathway positions.

7.2 **Health and medical research**

The HHS will:

(a) Articulate an investment strategy for research (including research targets and Performance Measures) which integrates with the clinical environment to improve clinical outcomes;

(b) Develop mechanisms for completing administrative governance for the approval of research in line with a national benchmark of 25 days (Standard Operating Procedures for Queensland Health Research Governance Officers 2013);

(c) Develop mechanisms for monitoring site research activity in line with jurisdictional commitments and National Health and Medical Research Council Guidelines (*Framework for* Monitoring *Guidance for the national approach to single ethical review of multi-centre research, January 2012*); and

(d) Develop systems to capture research and development expenditure and revenue data and associated information on research.

Schedule 3 Performance Measures

1. Purpose

This Schedule 3 outlines the Performance Measures that apply to the HHS.

2. Performance Measures

2.1 The Performance and Accountability Framework uses Performance Measures to monitor the extent to which the HHS is delivering the high-level objectives set out in this Service Agreement.

2.2 Each Performance Measure is identified under one of four categories:

(a) Safety and Quality Markers which together provide timely and transparent information on the safety and quality of services provided by the HHS;

(b) Key Performance Indicators (KPIs) which are focused on the delivery of key strategic objectives and statewide targets. KPI performance will inform HHS performance assessments;

(c) Outcome Indicators which provide information on specific activities and interventions where there is a reasonable expectation or evidence that they make a positive contribution towards improving the health status and experience of patients; and

(d) supporting indicators which provide contextual information and enable an improved understanding of performance, facilitate benchmarking of performance across HHSs and provide intelligence on potential future areas of focus. Supporting indicators are available on-line as referenced in Appendix 1 to this Service Agreement.

2.3 The HHS should refer to the relevant attribute sheet for each Performance Measure for full details. These are available on-line as referenced in Appendix 1 to this Service Agreement.

2.4 The Performance Measures identified in Table 9; Table 10 and Table 11 are applicable to the HHS unless otherwise specified within the attribute sheet.

2.5 The HHS will meet the target for each KPI identified in Table 9 as specified in the attribute sheet.

2.6 The Performance Measures identified in italic text are for future development.

2.7 Further information on the performance assessment process is provided in the supporting document to this Service Agreement, Performance and Accountability Framework 2020/21 referenced at Appendix 1 to this Service Agreement.

Table 9 HHS Performance Measures – Key Performance Indicators

**Key Performance Indicators**	
**Safe**	
The health and welfare of service users is paramount	
• Minimise risk	• Avoid harm from care
• Transparency and openness	• Learn from mistakes
**Title**	
Hospital Acquired Complications	
Emergency length of stay:<br>• % of Emergency Department attendances who are admitted as an inpatient, including to a short stay unit, and whose Emergency Department length of stay is within 4 hours	
Number of Emergency Department stays greater than 24 hours	
Emergency Department wait time by triage category	
Rate of face to face community follow up within 1-7 days of discharge from an acute mental health inpatient unit	
**Timely**	
Care is provided within an appropriate timeframe	
• Treatment within clinically recommended time	
**Title**	
Patient off stretcher time:<br>• % of patients transferred from Queensland Ambulance Service into the Emergency Department within 30 minutes	
Elective surgery:<br>• % of category 1 patients treated within the clinically recommended time	
Elective surgery:<br>• Number of ready for care patients waiting longer than the clinically recommended timeframe for their category	
Specialist outpatients:<br>• % of category 1 patients who receive their initial specialist outpatient appointment within the clinically recommended time	
Specialist outpatients:<br>• Number of ready for care category 1 patients waiting longer than clinically recommended for their initial specialist outpatient appointment	
Gastrointestinal endoscopy:<br>• % of category 4 patients who are treated within the clinically recommended time	
Gastrointestinal endoscopy:<br>• Number of patients waiting longer than clinically recommended timeframe for their category	
Access to oral health services:<br>• % of patients on the general care dental wait list waiting for less than the clinically recommended time	

**Equitable**	
Consumers have access to healthcare that is responsive to need and addresses health inequalities	
• Fair access based on need	• Addresses inequalities
**Title**	
Potentially Preventable Hospitalisations – First Nations People	
Telehealth utilisation rates:<br>• Number of non-admitted telehealth service events	
**Efficient**	
Available resources are maximised to deliver sustainable, high quality healthcare	
• Avoid waste	• Minimise financial risk
• Sustainable/productive	• Maximise available resources
**Title**	
Forecast operating position:<br>• Full year<br>• Year to date	
Average sustainable Queensland Health FTE	
Capital expenditure performance	
**Patient Centred**	
Providing Healthcare that is respectful of and responsive to individual patient preferences, needs and values	
• Patient involved in care	• Patient feedback
• Respects patient/person values and preferences	• Care close to home
**Title**	
Proportion of mental health service episodes with a documented care plan	
Proportion of beds vacated by 11am	

Table 10 HHS Performance Measures - Safety and Quality Markers

**Safety and Quality Markers**	
**Safe**	
The health and welfare of service users is paramount	
• Minimise risk<br>• Transparency and openness	• Avoid harm from care<br>• Learn from mistakes
**Title**	
Sentinel Events:<br>• Number of wholly preventable sentinel events	
Hospital Standardised Mortality Ratio	
Healthcare-associated Staphylococcus Aureus (including MRSA) bacteraemia:<br>• Rate per 10,000 patient days	
Severity Assessment Code (SAC) closure rates:<br>• % of incidents closed within the prescribed timeframe	
Unplanned Readmission Rates	

Table 11 HHS Performance Measures – Outcome Indicators

Outcome Indicators	
**Safe**	
**The health and welfare of service users is paramount**	
• Minimise risk<br>• Transparency and openness	• Avoid harm from care<br>• Learn from mistakes
**Title**	
Rate of seclusion events per 1,000 acute mental health admitted patient days	
Rate of absent without approval from acute mental health inpatient care	
**Timely**	
Care is provided within an appropriate timeframe	
• Treatment within clinically recommended time	
**Title**	
Reperfusion therapy for acute ischaemic stroke:<br>• Proportion of patients treated with either IV thrombolytic drugs or endovascular clot retrieval	
Access to emergency dental care:<br>• % of emergency courses of care for adult dental patients that commence within the recommended waiting times	
**Equitable**	
Consumers have access to healthcare that is responsive to need and addresses health inequalities	
• Fair access based on need	• Addresses inequalities
**Title**	
First Nations people representation in the workforce:<br>• % of the workforce who identify as being First Nations people	
Completed general courses of oral health care for First Nations people adult patients	
Low birthweight:<br>• % of low birthweight babies born to Queensland mothers	
**Patient Centred**	
Providing Healthcare that is respectful of and responsive to individual patient preferences, needs and values	
• Patient involved in care	• Patient feedback
• Respects patient/person values and preferences	• Care close to home
**Title**	
Complaints resolved within 35 calendar days	
Advance care planning:<br>• The proportion of approaches made to people who are identified as being at risk of dying within the next 12 months, or suitable for an advance care planning discussion, and who are offered the opportunity to consider, discuss and decide their preferences for care at the end of life	

Effective	
Healthcare that delivers the best achievable outcomes through evidence-based practice	
• Evidence based practice	• Care integration
• Treatment directed to those who benefit	• Optimise Health
• Clinical Capability	

**Title**
Uptake of the smoking cessation clinical pathway for public hospital inpatients and dental clients
Potentially Preventable Hospitalisations – diabetes complications:<br>• The number and proportion of hospitalisations of people with Diabetes complications that could have potentially been prevented through the provision of appropriate non-hospital health services.
Potentially Preventable Hospitalisations – non-diabetes complications:<br>• The number and proportion of hospitalisations of people with non-Diabetes complications that could have potentially been prevented through the provision of appropriate non-hospital health services.
% of oral health activity which is preventive
Cardiac rehabilitation:<br>• Proportion of public cardiac patients that are referred to cardiac rehabilitation and complete a timely patient journey
Adolescent vaccinations administered via the statewide School Immunisation Program

Schedule 4 Data Supply Requirements

1. Purpose

1.1 *The Hospital and Health Boards Act 2011*[10] (s.16(1)(d)) provides that the Service Agreement will state the performance data and other data to be provided by an HHS to the Chief Executive, including how, and how often, the data is to be provided.

1.2 This Schedule 4 specifies the data to be provided by the HHS to the Chief Executive and the requirements for the provision of the data.

2. Principles

2.1 The following principles guide the collection, storage, transfer and disposal of data:

(a) trustworthy – data is accurate, relevant, timely, available and secure;

(b) private – personal information is protected in accordance with the law;

(c) valued – data is a core strategic asset;

(d) managed – collection of data is actively planned, managed and compliant; and

(e) quality – data provided is complete, consistent, undergoes regular validation and is of sufficient quality to enable the purposes outlined in clause 3.2 of this Schedule 4 to be fulfilled.

2.2 The Parties agree to constructively review the data supply requirements as set out in this Schedule 4 on an ongoing basis in order to:

(a) ensure data supply requirements are able to be fulfilled; and

(b) minimise regulatory burden.

3. Roles and responsibilities

3.1 **Hospital and Health Services**

(a) The HHS will:

(i) provide, including the form and manner and at the times specified, the data specified in the data supply requirements (Attachment A to this Schedule 4) in accordance with this Schedule 4;

(ii) provide data in accordance with the provisions of the *Hospital and Health Boards Act 2011, Public Health Act 2005* and *Private Health Facilities Act 1999;*

[10] Section 143(2)(a) of the *Hospital and Health Boards Act 2011* provides that the disclosure of confidential information (as defined in s.139 of the Act) to the Chief Executive by an HHS under a service agreement is a disclosure permitted by an Act.

(iii) provide other HHSs with routine access to data, that is not Patient Identifiable Data, for the purposes of benchmarking and performance improvement;

(iv) provide data as required to facilitate reporting against the Performance Measures set out in Schedule 3 of this Service Agreement;

(v) provide data as specified within the provision of a health service directive;

(vi) provide activity data that complies with the national data provision timeframes required under the Independent Hospital Pricing Authority (IHPA) data plan for Commonwealth funding. Details of the timeframes are specified in the 'Commonwealth Efficient Growth Funding and National Weighted Activity Units (NWAUs)' specification sheet included in the supporting document Purchasing Policy and Funding Guidelines 2020/21 and the clinical placement data supply requirements; and

(vii) as requested by the Chief Executive from time to time, provide to the Chief Executive data, whether or not specified in this Schedule 4 or the Service Agreement, as specified by the Chief Executive in writing to the HHS in the form and manner and at the times specified by the Chief Executive.

(b) Data that is capable of identifying patients will only be disclosed as permitted by, and in accordance with, the *Hospital and Health Boards Act 2011, Public Health Act 2005 and the Private Health Facilities Act 1999.*

3.2 **Department**

The Department will:

(a) produce a monthly performance report which includes:

(i) actual activity compared with purchased activity levels;

(ii) any variance(s) from purchased activity;

(iii) performance information as required by the Department to demonstrate HHS performance against the Performance Measures specified in Schedule 3 of this Service Agreement; and

(iv) performance information as required by the Department to demonstrate the achievement of commitments linked to specifically allocated funding included in Schedule 2 of this Service Agreement.

(b) utilise the data sets provided for a range of purposes including:

(i) to fulfil legislative requirements;

(ii) to deliver accountabilities to state and commonwealth governments;

(iii) to monitor and promote improvements in the safety and quality of Health Services;

(iv) to support clinical innovation; and

(c) advise the HHS of any updates to data supply requirements as they occur.

Attachment A Data Supply Requirements

The HHS should refer to the relevant minimum data set for full details. These are available on-line as referenced in Appendix 1.

Table 12 Clinical data

Data Set	Data Custodian
Aged Care Assessment Team data via the Aged Care Evaluation (ACE) database	Strategic Policy Unit
Alcohol Tobacco and Other Drug Treatment Services	Mental Health Alcohol and Other Drugs Branch
Alcohol and Other Drugs Establishment Collection	Mental Health Alcohol and Other Drugs Branch
Allied Health Clinical Placement Activity Data	Allied Health Professions Office of Queensland
Australian and New Zealand Intensive Care Society (ANZICS) Data Collection	Healthcare Improvement Unit
BreastScreening Clinical Data	Executive Director, Preventive Health Branch
Clinical Incident Data Set	Patient Safety and Quality Improvement Service
Clinical Placement Data (excluding Allied Health)	Workforce Strategy Branch
Consumer Feedback Data Set	Patient Safety and Quality Improvement Service
Elective Surgery Data Collection	Healthcare Improvement Unit
Emergency Data Collection	Healthcare Improvement Unit
Gastrointestinal Endoscopy Data Collection	Healthcare Improvement Unit
Hand Hygiene Compliance Data	Communicable Diseases Branch
Healthcare Infection Surveillance Data	Communicable Diseases Branch
Maternal Deaths	Queensland Maternal and Perinatal Quality Council (through Statistical Services Branch)
Mental Health Act Data	Mental Health Alcohol and Other Drugs Branch
Mental Health Activity Data Collection	Mental Health Alcohol and Other Drugs Branch
Mental Health Carer Experience Survey Collection	Mental Health Alcohol and Other Drugs Branch
Mental Health Establishments Collection	Mental Health Alcohol and Other Drugs Branch
Monthly Activity Collection (including admitted and non-admitted patient activity and bed availability data)	Statistical Services Branch
Newborn Hearing Screening	Children's Health Queensland
Notifications Data	Chief Health Officer
Patient Experience Survey Data	Patient Safety and Quality Improvement Service
Patient Level Costing and Funding Data	HHS Funding and Costing Unit
Perinatal Data Collection	Statistical Services Branch
Queensland Bedside Audit	Patient Safety and Quality Improvement Service
Queensland Health Non-Admitted Patient Data Collection	Statistical Services Branch
Queensland Hospital Admitted Patient Data Collection	Statistical Services Branch
Queensland Needle and Syringe Program (QNSP) data	Chief Health Officer
Queensland Opioid Treatment Program Admissions and Discharges	Chief Health Officer
Radiation Therapy Data Collection	Healthcare Improvement Unit

Data Set	Data Custodian
Residential Mental Health Care Collections	Mental Health Alcohol and Other Drugs Branch
Schedule 8 Dispensing data	Chief Health Officer
School Immunisation Program – Annual Outcome Report	Communicable Diseases Branch
Specialist Outpatient Data Collection	Healthcare Improvement Unit
National Notifiable Diseases Surveillance System	Chief Health Officer
Vaccination Administration data	Chief Health Officer
Variable Life Adjusted Display (VLAD) CM (collection of hospital investigations)	Patient Safety and Quality Improvement Service
Your Experience of Service (YES) Survey Collection (Mental Health)	Mental Health Alcohol and Other Drugs Branch

Table 13 Non-clinical data

Non-Clinical Data Set	Data Custodian
Asbestos management data	Capital and Asset Services Branch
Asset Management<br>• Planning<br>• Maintenance<br>• Maintenance Budget<br>• Statement of Building Portfolio Compliance<br>• Benchmarking & Performance Data	Capital and Asset Services Branch
Conduct and Performance Excellence (CaPE)	Human Resources Branch
Expenditure	Finance Branch
Financial and Residential Activity Collection (FRAC)	Statistical Services Branch
Graduate Nursing Recruitment Data Statewide using the Public Service Commission Graduate Portal System	Office of the Chief Nursing and Midwifery Officer
Hospital Car Parks (including Government Portfolio Model funding arrangements)	Capital and Asset Services Branch
Minimum Obligatory Human Resource Information (MOHRI)	Finance Branch
Minor Capital Funding Program expenditure & forecast data	Finance Branch
Recruitment Data	Human Resources Branch
Revenue	Finance Branch
Queensland Health Workforce & Work Health & Safety Data	Human Resources Branch
Queensland Integrated Safety Information Project (QISIP)Solution Minimum Data Set	Human Resources Branch
Statewide employment matters	Human Resources Branch
Sustaining Capital Reporting Requirements (other than minor capital)	Capital and Asset Services Branch
Whole of Government Asset Management Policies data	Capital and Asset Services Branch

Schedule 5 Amendments to this Service Agreement

1. Purpose

This Schedule 5 sets out the mechanisms through which this Service Agreement may be amended during its term, consistent with the requirements of the *Hospital and Health Boards Act 2011.*

2. Principles

2.1 It is acknowledged that the primary mechanism through which HHS funding adjustments are made is through the budget build process that is undertaken annually in advance of the commencement of the financial year. This approach is intended to provide clarity, certainty and transparency in relation to funding allocations.

2.2 Amendments to the clauses of this Service Agreement should be progressed for consideration as part of the annual budget build process.

2.3 It is recognised that there is a requirement to vary funding and activity in-year. The following principles will guide amendments and amendment processes:

(a) funding allocations to HHSs should occur as early as possible within a financial year if unable to be finalised in advance of a given financial year;

(b) the number of Amendment Windows each year should be minimised to reduce the administrative burden on HHSs and the Department;

(c) Amendment Proposals should be minimised wherever possible and should always be of a material nature;

(d) Amendment Windows 2 and 3 are not intended to include funding or activity variations that could have been anticipated in advance of the financial year;

(e) Amendment Windows are intended to provide a formal mechanism to transact funding or activity variations in response to emerging priorities;

(f) Extraordinary Amendment Windows are not intended to be routinely used.

2.4 The Department remains committed to the ongoing simplification and streamlining of amendment processes.

3. Process to amend this Service Agreement

3.1 The Parties recognise the following mechanisms through which an amendment to this Service Agreement can be made:

(a) Amendment Windows;

(b) Extraordinary Amendment Windows;

(c) periodic adjustments; and

(d) end of year financial adjustments.

3.2 Amendment Windows

(a) In order for the Department to manage amendments across all HHS Service Agreements and their effect on the delivery of Public Sector Health Services in Queensland, proposals to amend this Service Agreement will be negotiated and finalised during set periods of time during the year (Amendment Windows).

(b) Amendment Windows are the primary mechanism through which amendments to this Service Agreement are made.

(c) Amendment Windows occur three times within a given financial year:

(i) Amendment Window 1: Annual Budget Build;

(ii) Amendment Window 2: In-year variation; and

(iii) Amendment Window 3: In-year variation.

(d) A Party that wants to amend the terms of this Service Agreement must give an Amendment Proposal to the other party.

(e) While a Party may submit an Amendment Proposal at any time, an Amendment Proposal will only be formally negotiated and resolved during one of the Amendment Windows outlined in Table 14 (excluding Extraordinary Amendment Windows).

Table 14 Amendment Window Exchange Dates

Amendment Window	Exchange Date	Primary Focus
Amendment Window 2: In-year variation	4 October 2019	2019/20 in-year variations
Amendment Window 3: In-year variation	14 February 2020	2019/20 in-year variations
Amendment Window 1: Annual Budget Build	27 March 2020	2020/21 budget build
Amendment Window 2: In-year variation	9 October 2020	2020/21 in-year variations
Amendment Window 3: In-year variation	12 February 2021	2020/21 in-year variations
Amendment Window 1: Annual Budget Build	26 March 2021	2021/22 budget build
Amendment Window 2: In-year variation	8 October 2021	2021/22 in-year variations
Amendment Window 3: In-year variation	11 February 2022	2021/22 in-year variations

(f) An Amendment Proposal is made by:

(i) the responsible Deputy Director-General signing and providing an Amendment Proposal to the HHS-SA Contact Person through Healthcare Purchasing and System Performance Division prior to the commencement of any Amendment Window; or

(ii) the Health Service Chief Executive signing and providing an Amendment Proposal to the D-SA Contact Person prior to the commencement of any Amendment Window.

(g) A Party giving an Amendment Proposal must provide the other Party with the following information:

(i) the rationale for the proposed amendment;

(ii) the precise drafting for the proposed amendment;

(iii) any information and documents relevant to the proposed amendment; and

(iv) details and explanation of any financial, activity or service delivery impact of the amendment.

(h) Negotiation and resolution of Amendment Proposals will occur during the Negotiation Period through a tiered process, as outlined in Figure 3.

Figure 3 Amendment Proposal negotiation and resolution

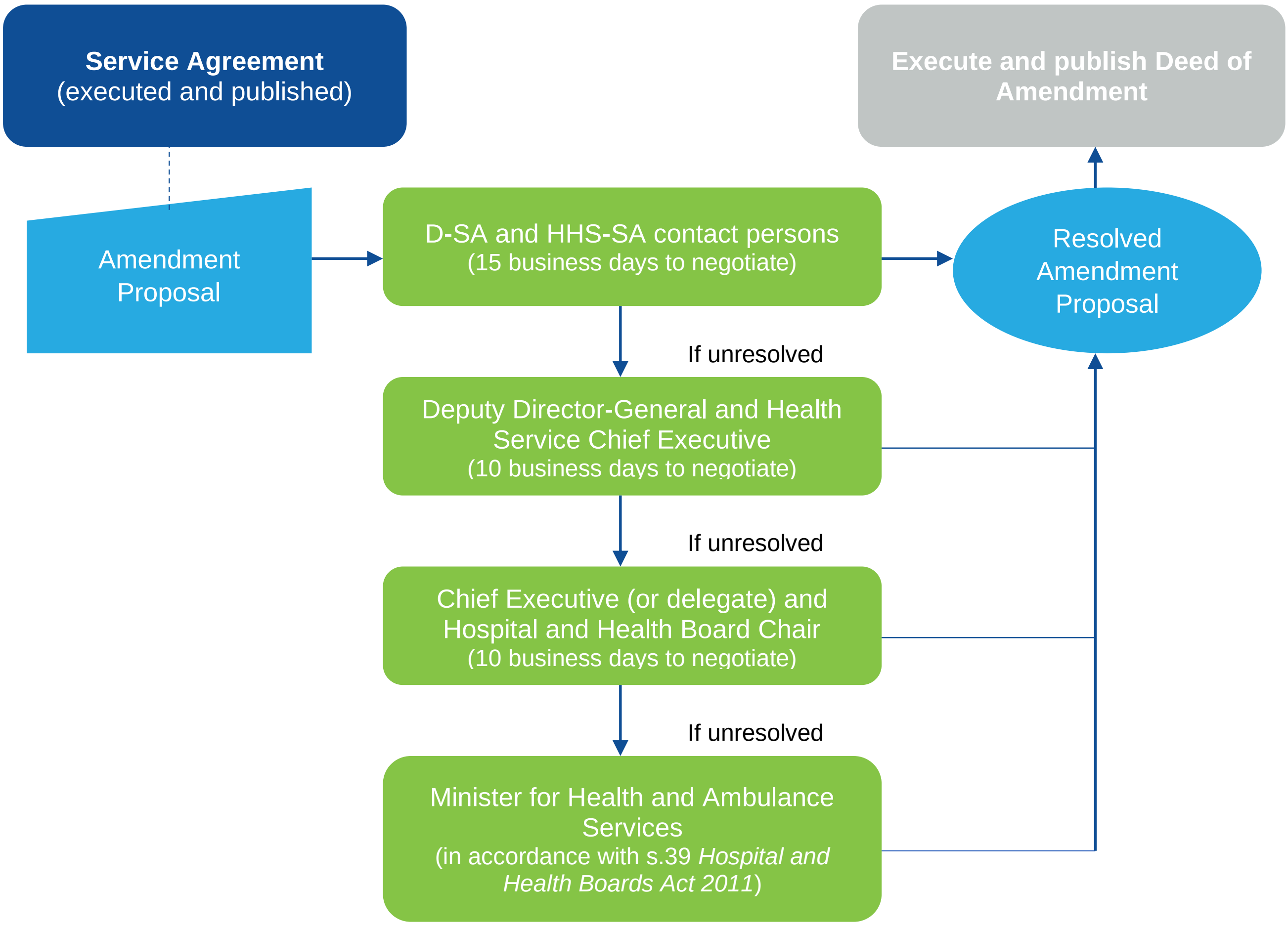

(i) The timeframes identified in Figure 3 may be reduced in the complete discretion of the Chief Executive.

(j) In accordance with section 39(5) of the *Hospital and Health Boards Act 2011*, the Parties must include any terms decided by the Minister in the Service Agreement.

(k) If the Chief Executive at any time:

(i) considers that an amendment agreed with the HHS may or will have associated impacts on other HHSs; or

(ii) considers it appropriate for any other reasons,

then the Chief Executive may:

(iii) propose further amendments to any HHS affected; and

(iv) may address the amendment and/or associated impacts of the

amendment in other ways, including through the exercise of any statutory powers and/or statutory directions under the *Hospital and Health Boards Act 2011*.

(l) Amendment Proposals that are resolved will be documented in a Deed of Amendment to this Service Agreement and executed by the Parties.

(m) Only upon execution of a Deed of Amendment by the Parties will the amendments documented by that Deed of Amendment be deemed to be an amendment to this Service Agreement.

3.3 **Extraordinary Amendment Windows**

(a) A Party that wants to amend the terms of this Service Agreement outside of an Amendment Window outlined in Table 14 must give an Extraordinary Amendment Proposal to the other Party.

(b) An Extraordinary Amendment Proposal may only be formally negotiated and resolved outside of an Amendment Window outlined in Table 14 to facilitate funding allocations where an urgent priority needs to be addressed in a timely manner and an Amendment Window is not available within an acceptable timeframe.

(c) An Extraordinary Amendment Proposal that is issued by or on behalf of the Chief Executive must be given to the HHS-SA Contact Person through Healthcare Purchasing and System Performance Division.

(d) An Extraordinary Amendment Proposal that is issued by or on behalf of the HHS must be given to the D-SA Contact Person through Healthcare Purchasing and System Performance Division.

(e) An Extraordinary Amendment Proposal may be issued by or on behalf of either Party at any time, noting the requirement that it relate to an urgent priority that necessitates timely resolution.

(f) Negotiation and resolution of Extraordinary Amendment Proposals will be through a tiered process as outlined in Figure 3.

(g) The timeframes identified in Figure 3 may be reduced in the complete discretion of the Chief Executive.

(h) In accordance with section 39(5) of the *Hospital and Health Boards Act 2011*, the Parties must include any terms decided by the Minster in the Service Agreement.

(i) Extraordinary Amendment Proposals that are resolved must be executed by both Parties.

(j) The Parties must comply with the terms of the Extraordinary Amendment Proposal from the date that the final Party executed the Extraordinary Amendment Proposal.

(k) The terms of an executed Extraordinary Amendment Proposal will be documented in a Deed of Amendment to this Service Agreement and executed by the Parties. Once executed, the Deed of Amendment will expressly exclude the application of the Extraordinary Amendment Proposal and only the terms of the Deed of Amendment will apply.

3.4 Periodic adjustments

(a) The Service Agreement Value may be adjusted outside of an Amendment Window to allow for funding variations that:

(i) occur on a periodic basis;

(ii) are referenced in the Service Agreement; and

(iii) are based on a clearly articulated formula.

(b) Adjustments to the Service Agreement Value and purchased activity that are required as a result of a periodic adjustment will be made following agreement between the Parties of the data on which the adjustment is based.

(c) The Chief Executive (or delegate) will issue an Adjustment Notice to the HHS-SA Contact Person confirming any funding adjustment that has been made.

(d) Following receipt of an Adjustment Notice, the Parties will comply with the Adjustment Notice and immediately take the steps necessary to give effect to the requirements of the Adjustment Notice.

(e) A Deed of Amendment will not be issued immediately following periodic adjustment. The HHS will be provided with a summary of all transactions made through periodic adjustment on completion.

(f) Any funding adjustments agreed through periodic adjustment which result in a variation to the Service Agreement Value, purchased activity or the requirements specified within Schedule 2 of this Service Agreement will be formalised in a Deed of Amendment issued following the next available Amendment Window.

3.5 End of financial year adjustments

(a) End of year financial adjustments may be determined after the financial year end outside of the Amendment Window process.

(b) The scope will be defined by the Department and informed by Queensland Government Central Agency requirements.

(c) The Department will provide the HHS with a reconciliation of all Service Agreement funding and purchased activity for the prior financial year. This will reflect the agreed position between the Parties following conclusion of the end of year financial adjustments process.

(d) The impact of end of year financial adjustments on subsequent year funding and activity will be incorporated in the Service Agreement through the Deed of Amendment executed following the next available Amendment Window.

(e) This clause will survive expiration of this Service Agreement.

Schedule 6 Definitions

In this Service Agreement:

Activity Based Funding (ABF) means the funding framework for publicly-funded health care services delivered across Queensland. The ABF framework applies to those Queensland public sector health service facilities which are operationally large enough and have the systems which are required to support the framework. The ABF framework allocates health funding to these hospitals based on standardised costs of health care services (referred to as 'activities') delivered. The Framework promotes smarter health care choices and better care by placing greater focus on the value of the health care delivered for the amount of money spent.

Adjustment Notice means the written notice of a proposed funding adjustment made by or on behalf of the Chief Executive in accordance with the terms of this Service Agreement.

Administrator of the National Health Funding Pool means the position established by the *National Health Reform Amendment (Administrator and National Funding Body) Act 2012* for the purposes of administering the National Health Funding Pool according to the National Health Reform Agreement.

Agreement means this Service Agreement.

Ambulatory Care means the care provided to hospital patients who are not admitted to the hospital, such as patients of emergency departments and outpatient clinics. Can also be used to refer to care provided to patients of community-based (non-hospital) healthcare services.

Amendment Proposal means the written notice of a proposed amendment to the terms of this Service Agreement as required under section 39 of the *Hospital and Health Boards Act 2011*.

Amendment Window means the period within which Amendment Proposals are negotiated and resolved. Amendment Windows commence on the relevant Exchange Date as specified in Table 14 Schedule 5 and end at the conclusion of the Negotiation Period.

Block Funding means funding for those services which are outside the scope of ABF.

Business Day means a day which is not a Saturday, Sunday or public holiday in Brisbane.

Chair means the Chair of the Hospital and Health Board.

Chief Executive means the chief executive of the Department.

Clinical Product/Consumable means a product that has been Clinically Prescribed.

Clinically Prescribed means prescribed by appropriately qualified and credentialed clinicians relative to the product.

Clinical Prioritisation Criteria means Statewide minimum criteria to determine if a referral to specialist medical or surgical outpatients is appropriate and, if so, the urgency of that referral.

Clinical Services Capability Framework means the Clinical Services Capability Framework for Public and Licensed Private Health Facilities which provides a standard set of minimum capability criteria for service delivery and planning. The Framework outlines the minimum service requirements, staffing, support services and risk considerations for both public and private health services to ensure safe and appropriately supported clinical service delivery. It applies to all public and private licensed facilities in Queensland. References to the Clinical Services Capability

Framework in this Service Agreement mean the most recent approved version unless otherwise specified.

Community Health Service means non-admitted patient Health Services, excluding hospital outpatient services, typically delivered outside of a hospital setting.

Deed of Amendment means the resolved amendment proposals.

Department means the department administering the *Hospital and Health Boards Act 2011* (Qld), which, at the date of this Service Agreement is known as 'Queensland Health'. To avoid any doubt, the term does not include the Hospital and Health Services.

D-SA Contact Person means the position nominated by the Department as the primary point of contact for all matters relating to this Service Agreement.

Effective Date means1 July 2019.

Efficient Growth means the increased in-scope activity-based services delivered by a HHS measured on a year to year basis in terms of both the Queensland efficient price for any changes in the volume of services provided and the growth in the national efficient price of providing the existing volume of services.

Eligible Population (Oral Health Services) refers to the proportion of the population for whom publicly funded oral health services is to be provided and is defined by the following criteria:

(a) adults, and their dependents, who are Queensland residents; eligible for Medicare and, where applicable, currently in receipt of benefits from at least one of the following concession cards:

 (i) Pensioner Concession Card issued by the Department of Veteran's Affairs;

 (ii) Pensioner Concession Card issued by Centrelink;

 (iii) Health Care Card (this includes Low Income Health Care Card Holders who are automatically eligible for services);

 (iv) Commonwealth Seniors Health Card;

 (v) Queensland Seniors Card.

(b) children who are Queensland residents or attend a Queensland school, are eligible for Medicare, and are:

 (i) eligible for dental program/s funded by the Commonwealth Government; or

 (ii) four years of age or older and have not completed Year 10 of secondary school; or

 (iii) dependents of current concession card holders or hold a current concession card.

Exchange Date means the date on which the Parties must provide Amendment Proposals for negotiation, as specified in Table 14 Schedule 5.

Extraordinary Amendment Window means an Amendment Window that occurs outside of the Amendment Windows specified in Table 14 Schedule 5, in accordance with the provisions of clause 3.3 of Schedule 5.

Force Majeure means an event:

(a) which is outside of the reasonable control of the Party claiming that the event has occurred; and

(b) the adverse effects of which could not have been prevented or mitigated against by that Party by reasonable diligence or precautionary measures, and includes lightning, earthquake, fire, cyclone, flood, natural disasters, health pandemics, acts of terrorism, riots, civil disturbances, industrial disputes and strikes (other than strikes involving that Party, its' agents, employees or suppliers), war (declared or undeclared), revolution, or radioactive contamination.

Formal Agreement means an agreed set of roles and responsibilities relating to the provision and receipt of services designated as Statewide or Regional:

(a) Statewide or Regional service provision

- (i) ensure equitable and timely access to entire catchment (clinical and non-clinical)
- (ii) provide training and consultation Services where this is appropriate within the agreed model of care (clinical and non-clinical)
- (iii) timely discharge or return of patients to their place of residence (clinical Services)
- (iv) adequate communication practices to enable ongoing effective local health care, including with the patient's General Practitioner where required (clinical Services)

(b) Recipient HHS

- (i) utilisation of standardised referral criteria, where they exist, to ensure appropriate use of Statewide Services (clinical services)
- (ii) timely acceptance of patients being transferred out of Statewide Services (back-transfers) (clinical Services)
- (iii) equitable access to ongoing local health care as required (clinical services)

Health Executive means a person appointed as a health executive under section 67(2) of the *Hospital and Health Boards Act 2011.*

Health Service has the same meaning as set out in section 15 of the *Hospital and Health Boards Act 2011.*

Health Service Chief Executive means a health service chief executive appointed for an HHS under section 33 of the *Hospital and Health Boards Act 2011.*

Health Service Employee means all person, appointed as a 'health service employee' for the HHS under section 67(1) of the *Hospital and Health Boards Act 2011.*

Hospital and Health Board means the hospital and health board appointed under section 23 of the *Hospital and Health Boards Act 2011*.

Hospital and Health Service or **HHS** means the Hospital and Health Service to which this Agreement applies unless otherwise specified.

HHS-SA Contact Person means the position nominated by the HHS as the primary point of contact for all matters relating to this Service Agreement.

HR Management Functions means the formal system for managing people within the HHS, including recruitment and selection; onboarding; induction and orientation; capability, learning and development; industrial and employee relations; performance management; work health and safety and well-being; workforce planning; diversity and inclusion; and workforce consultation, engagement and communication.

Industrial Instrument means an industrial instrument made under the *Industrial Relations Act 2016.*

Inter-HHS Dispute means a dispute between two or more HHSs.

Key Performance Indicator means a measure of performance that is used to evaluate the HHSs success in meeting key priorities.

Low Benefit Care means use of an intervention where evidence suggests it confers no or very little benefit on patients, or the risk of harm exceeds the likely benefit.

Minister means the Minister administering the *Hospital and Health Boards Act 2011* (Qld).

National Health Reform Agreement means the document titled *National Health Reform Agreement* made between the Council of Australian Governments (CoAG) in 2011, and incorporating all subsequent amendments agreed between the Commonwealth of Australia and the States and Territories.

Negotiation Period means a period of no less than 15 business days (or such longer period agreed in writing between the Parties) from each Exchange Date.

Notice of Dispute means the written notice of a dispute provided by the Chief Executive or the HHS to the other party or the written notice of a dispute provided by an HHS to another HHS.

Outcome Indicator means a measure of performance that provides information on specific activities and interventions where there is a reasonable expectation or evidence that they make a positive contribution towards improving the health status and experience of patients;

Outreach Service means a Health Service delivered on sites outside of the HHS area to meet or complement local service need. Outreach services include Health Services provided from one HHS to another as well as Statewide Services that may provide Health Services to multiple sites.

Own Source Revenue means, as per Section G3 of the *National Healthcare Agreement*, ‘private patients, compensable patients and ineligible persons may be charged an amount for public hospital services as determined by the state and territory’. The funding for these patients is called own source revenue and includes:

(a) Medicare ineligible patients, such as overseas visitors (not covered under reciprocal agreements), people in community detention and overseas students studying in Australia

(b) compensable patients with an alternate funding source, such as:

   (i) workers’ compensation insurers;

   (ii) motor vehicle accident insurers;

   (iii) personal injury insurers;

   (iv) Department of Defence; and/or

   (v) Department of Veterans’ Affairs; and

   Medicare eligible patients can elect to be treated as a public or private patient, allowing HHS' to recoup a portion of the healthcare service delivery cost.

Party means each of the Chief Executive and the HHS to which this Service Agreement applies.

Patient Identifiable Data means data that could lead to the identification of an individual either directly (for example by name), or through a combination of pieces of data that are unique to that individual.

Performance Review Meeting means the forum established which routinely reviews and discusses a range of aspects of HHS and system wide performance in accordance with the accountabilities contained within this Service Agreement and the Performance and Accountability Framework. Attendance at Performance Review Meetings comprises:

(a) the D-SA Contact Person and the HHS-SA Contact Person;

(b) executives nominated by the Department; and

(c) executives nominated by the HHS.

Performance Measure means a quantifiable indicator that is used to assess how effectively the HHS is meeting identified priorities and objectives.

Person Conducting a Business or Undertaking takes the meaning as defined in the *Work Health and Safety Act 2011,* section 5.

Prevention Services means programs that prevent illness and injury, promote health and wellbeing, create healthy and safe environments, reduce health inequalities and address factors in those communities whose health status is the lowest.

Primary Care means first level healthcare provided by a range of healthcare professionals in socially appropriate and accessible ways and supported by integrated referral systems. It includes health promotion, illness prevention, care of the sick, advocacy and community development.

Public Health Event of State Significance means an event where the actual or potential impact extends beyond the community service by a particular Hospital and Health Service.

Public Sector Health Service has the same meaning as set out in the *Hospital and Health Boards Act 2011.*

Public Sector Health System means the Queensland public sector health system, which is comprised of the Hospital and Health Services and the Department.

Quality Improvement Payment (QIP) means a non-recurrent payment due to the HHS for having met the goals set out in the QIP Purchasing Incentive Specification.

Queensland Government Central Agency means one or all of the Department of the Premier and Cabinet, Queensland Treasury, the Queensland Audit Office, the Public Service Commission and the Office of the Integrity Commissioner.

Regional Service means a clinical (direct or indirect patient care) or non-clinical Health Service funded and delivered, or coordinated and monitored, by an HHS with a catchment of two or more HHSs, but not on a Statewide basis as defined in this Schedule. Service delivery includes facility based, outreach and telehealth service models.

Referral Pathway means the process by which a patient is referred from one clinician to another in order to access the Health Services required to meet their healthcare needs.

Residential HHS means the HHS area, as determined by the *Hospital and Health Boards Regulation 2012*, in which the patient normally resides.

Safety and Quality Marker means a measure of performance that provides timely and transparent information on the safety and quality of Health Services provided by the HHS;

Schedule means this Schedule to the Service Agreement.

Senior Health Service Employee means a person appointed under section 67(2) of the *Hospital and Health Boards Act 2011* in a position prescribed as a 'senior health service employee position' under the *Hospital and Health Boards Regulation 2012.*

Service Agreement means this service agreement including the Schedules and annexures, as amended from time to time.

Service Agreement Value means the figure set out in Schedule 2 as the expected annual value of the services purchased by the Department through this Service Agreement.

State means the State of Queensland.

Statement of Building Portfolio Compliance means a declaration completed by the HHS stating that it has maintained compliance with all mandatory Acts, Regulations, Australian Standards and Codes of Practice applicable to the HHS' building portfolio.

Statewide Service means a service that is delivered by a lead provider to the State. A Statewide Service may be:

(a) a clinical service that is:

   (i) a low volume, highly specialised Health Service delivered from a single location;

   (ii) a highly specialised, or high risk[11], Health Service delivered in multiple locations or

   (iii) a prevention and/or health promotion service.

(b) a support service that is required to enable the delivery of specific direct clinical services; or

(c) services that have a primary role to provide clinical education services and/or training programs.

Statewide Service Description means a document that defines the Service to be provided by the HHS on a statewide basis and how the Statewide Service will be accessed and used by other HHSs across the State, including but not limited to:

(a) an overview of the Statewide Service;

(b) components of the Statewide Service;

(c) eligibility criteria;

(d) Service referrals and pathways; and

(e) governance and capability arrangements for the Statewide Service.

[11] A Health Service that, due to its nature, poses an increased threat of ongoing sustainability, efficiency and affordability.

Supporting Indicator means a measure of performance that provides contextual information to support an assessment of HHS performance.

Suspend and Suspension means to cause the temporary cessation of a service provided by the HHS under the terms of this Service Agreement. Suspension may result from, but is not exclusively due to, limitations in workforce capacity or issues regarding the safety or quality of the service provided.

Telehealth means the delivery of Health Services and information using telecommunication technology, including:

(a) live interactive video and audio links for clinical consultations and education;

(b) store and forward systems to enable the capture of digital images, video, audio and clinical data stored on a computer and forwarded to another location to be studied and reported on by specialists;

(c) teleradiology to support remote reporting and provision of clinical advice associated with diagnostic images; and

(d) telehealth services and equipment for home monitoring of health.

Terminate and Termination means the permanent cessation of a service provided by the HHS under the terms of this Service Agreement.

Treating HHS means the HHS area, as determined by the *Hospital and Health Boards Regulation 2012*, in which a patient is receiving treatment.

Value-Based Healthcare means delivering what matters most to patients in the most efficient way. Value-Based Healthcare is characterised by:

(a) the identification of clearly defined population segments of patients with similar needs around which clinically integrated teams organise and deliver care, rather than designing and organising care around medical specialities, procedures or facilities;

(b) a focus on delivering health outcomes and the experience of receiving care as defined from the patient perspective, not just the system or clinical perspective;

(c) connection between outcomes and the costs required to deliver the outcomes; and

(d) an integrated approach across the full cycle of care with a focus on the goal of health rather than just treatment.

Key Documents

Hospital and Health Services Service Agreements and supporting documents including:

(a) Hospital and Health Services Service Agreements

(b) Queensland Health System Outlook to 2026 for a sustainable health service

(c) Performance and Accountability Framework 2020/21

(d) Purchasing Policy and Funding Guidelines 2020/21

are available at: www.health.qld.gov.au/system-governance/health-system/managing/agreements-deeds

My health, Queensland's future: Advancing health 2026

www.health.qld.gov.au/__data/assets/pdf_file/0025/441655/vision-strat-healthy-qld.pdf

Queensland Health 2020-2021 System Priorities

[link to follow]

Department of Health Strategic Plan

www.health.qld.gov.au/system-governance/strategic-direction/plans/doh-plan

Guideline for Immunisation Services

https://www.health.qld.gov.au/__data/assets/pdf_file/0026/147545/qh-gdl-955.pdf

Queensland Health Statement of Action towards Closing the Gap in health outcomes

https://qheps.health.qld.gov.au/atsihb/html/statement-of-action

HHS Performance Measures and Attribute Sheets

https://qheps.health.qld.gov.au/purchasing-performance/hhs-agreements/performance-kpis

Data Supply Requirements

https://qheps.health.qld.gov.au/purchasing-performance/hhs-agreements/data-reporting-requirements

Australian Commission on Safety and Quality in Healthcare – National Safety and Quality Health Service Standards

https://www.safetyandquality.gov.au/standards/nsqhs-standards

Statewide Services Governance and Risk Management Framework

https://qheps.health.qld.gov.au/spb/html/statewide-services/statewide-services-governance-and-risk-management-framework

Public Health Practice Manual

https://qheps.health.qld.gov.au/__data/assets/pdf_file/0035/667754/public-health-prac-man.pdf

National Healthcare Agreement

http://www.federalfinancialrelations.gov.au/content/national_agreements.aspx

National Health Reform Agreement

www.federalfinancialrelations.gov.au/content/national_health_reform.aspx

Abbreviations

ACQSC	Aged Care Quality and Safety Commission
ABF	Activity Based Funding
ACSQHC	Australian Commission on Safety and Quality in Healthcare
CET	Clinical Education and Training
D-SA	Department – Service Agreement
HHS	Hospital and Health Service
HHS-SA	Hospital and Health Service – Service Agreement
HITH	Hospital in the Home
KPI	Key Performance Indicator
LAM	List of Approved Medicines
Non-ABF	Non-Activity Based Funding
NPA	National Partnership Agreement
NSQHS	National Safety and Quality Health Service Standards
NWAU	National Weighted Activity Unit
PBS	Pharmaceutical Benefits Scheme
QAS	Queensland Ambulance Service
QIP	Quality Improvement Payment
QWAU	Queensland Weighted Activity Unit
RACGP	Royal Australian College of General Practitioners
SA2	Statistical Area Level 2

This page is intentionally left blank.

Queensland Health
www.health.qld.gov.au

Queensland Government

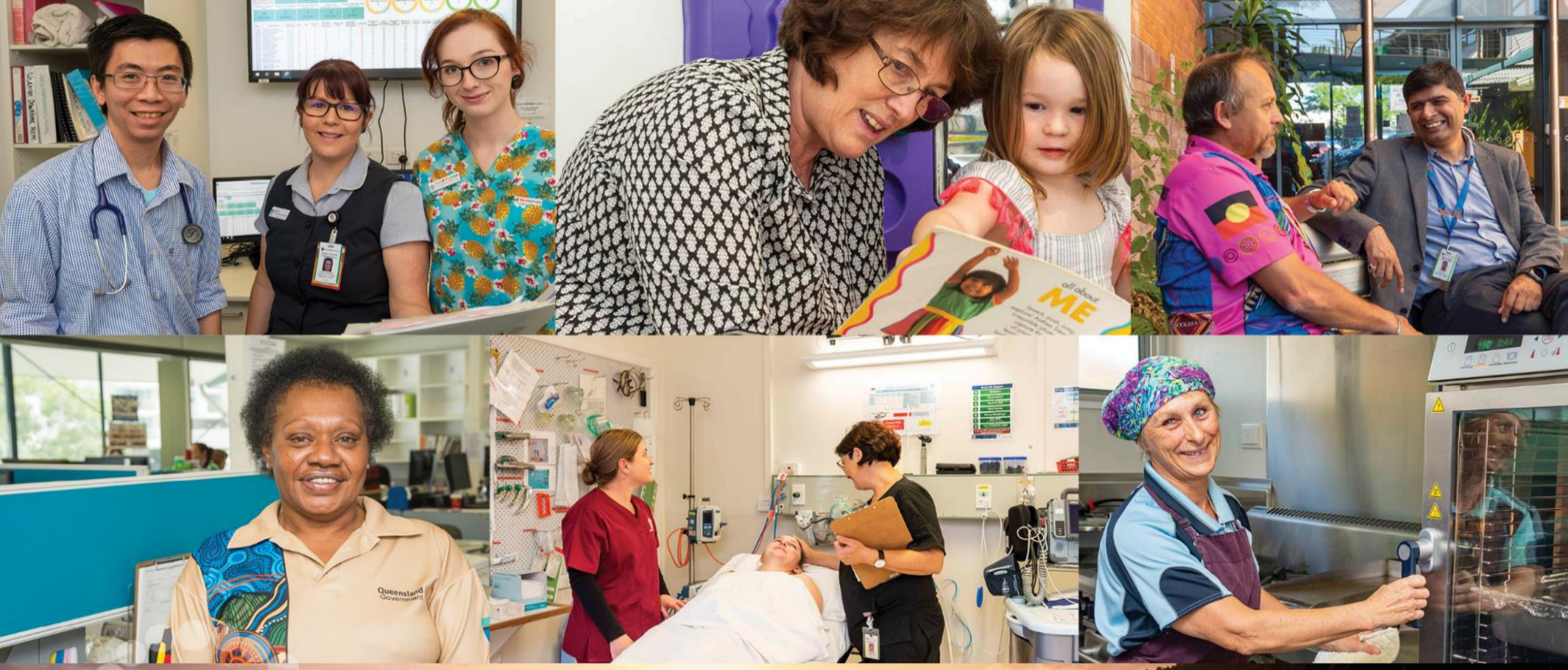

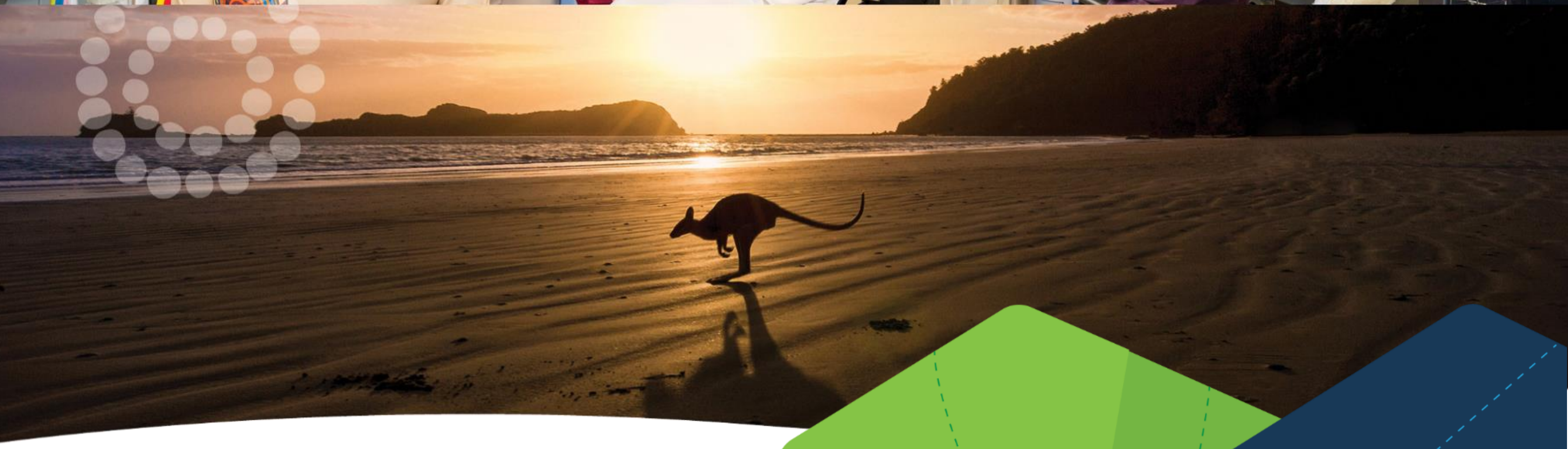

Queensland Health

Service Agreement 2022/23 – 2024/25

Mackay Hospital and Health Service

December 2024 Revision

Queensland Government

Mackay Hospital and Health Service, Service Agreement 2022/23 - 2024/25

Published by the State of Queensland, (Queensland Health), December 2024

This document is licensed under a Creative Commons Attribution 3.0 Australia licence. To view a copy of this licence, visit creativecommons.org/licenses/by/3.0/au

© State of Queensland (Queensland Health) **2024**

You are free to copy, communicate and adapt the work, as long as you attribute the State of Queensland (Queensland Health).

For more information contact:

Contracting and Performance Management Branch, Department of Health, GPO Box 48, Brisbane QLD 4001
Tel (+61) (07) 3708 5869

Email SAM@health.qld.gov.au

An electronic version of this document is available at www.health.qld.gov.au/system-governance/health-system/managing/default.asp

Disclaimer:
The content presented in this publication is distributed by the Queensland Government as an information source only. The State of Queensland makes no statements, representations or warranties about the accuracy, completeness or reliability of any information contained in this publication. The State of Queensland disclaims all responsibility and all liability (including without limitation for liability in negligence) for all expenses, losses, damages and costs you might incur as a result of the information being inaccurate or incomplete in any way, and for any reason reliance was placed on such information.

Acknowledgement

We acknowledge the Aboriginal Traditional Custodian(s) as the Cultural Custodians of the lands, waters and seas across the Mackay, Whitsunday and Isaac regions. We acknowledge and pay our respects to Aboriginal and Torres Strait Islander (First Nation) Peoples, acknowledging Elders past and present and recognise the role of current and emerging leaders in shaping our health systems.

We recognise the First Nations people in Queensland and support the cultural knowledge, determination and commitment of Aboriginal and Torres Strait Islander communities in caring for health and wellbeing for millennia. Mackay Hospital and Health Service declares our commitment to reducing inequalities in health outcomes for Aboriginal and Torres Strait Islander peoples as we move to a place of equity, justice and partnership together.

Whilst the Australian South Sea Islander people are not recognised as First Nation peoples, Mackay Hospital and Health Service acknowledges Australian South Sea Islander people, their historical relationship with First Nation peoples and the contributions they have made in the community.

We are proud to recognise and celebrate the cultural diversity of our communities and workforce at the following locations:

Facility	Traditional Owner Group	Region
Bowen Hospital	Juru People (jer-roo)	Whitsunday
Bowen Community Health Service	Juru People	Whitsunday
Clermont Multi-Purpose Health Services	Wangan Jagalingou People (wan-gan jaga-lin-goo)	Isaac
Collinsville Multi-Purpose Health Services	Birriah People (birryah)	Whitsunday
Dysart Hospital	Barada Barna People (ba-rada barn-a)	Isaac
Glendon Community Health Service	Wiri/Widi People (wirry/widdy)	Isaac
Mackay Base Hospital	Yuwi People (you-ee)	Mackay
Mackay Community Health Service	Yuwi People	Mackay
Middlemount Community Health Service	Barada Barna People	Isaac
Moranbah Hospital	Barada Barna People	Isaac
Monash Lodge Aged Care Facility (Clermont)	Wangan Jagalingou People	Isaac
Moranbah Community Health Service	Barada Barna People	Isaac
Proserpine Hospital	Gia People	Whitsunday
Sarina Hospital & Primary Health Care Centre	Yuwi People	Mackay
Whitsunday Community Health Service	Ngaro People (garo)	Whitsunday

Contents

Acknowledgement 3
Contents 4
Tables 5
1. Introduction 6
2. Scope 6
3. Services 6
4. Performance and Accountability Framework 7
5. Outcomes Framework 7
6. Data supply requirements 8
7. Hospital and Health Service accountabilities 8
8. Department accountabilities 10
9. Achieving health equity with First Nations Queenslanders 10
10. Dispute Resolution 11
11. General 11
12. Counterparts 12
Execution 13
Schedule 1 HHS profile 14
1. HHS profile 14
Schedule 2 Funding and purchased activity and services 15
1. Introduction 15
2. Purchased health services 16
3. Teaching, training, and research 18
4. Delivery of purchased activity 19
5. Delivery of purchased services 20
6. Financial adjustments 21
7. Funding sources 25
8. Funds disbursement 25
Schedule 3 Department of Health Provided Services 32
1. In scope services and service schedules 32
Schedule 4 Performance Measures 33
1. Performance Measures 33
Schedule 5 Amendments to this service agreement 37
1. Agreed process to amend this service agreement 37
Appendix 1 Reference Documents 39

Tables

Table 1 Statewide Services...16
Table 2 Financial adjustments applied on breach of activity thresholds......................................22
Table 3 Funding adjustments 2024/25...22
Table 4 HHS Funding by Outcomes Framework Care Domain...30
Table 5 HHS Total Funding Allocation by Funding Source 2024/25..30
Table 6 National Health Reform Funding 29
Table 7 Discretely Funded Programs (Non-ABF)...31
Table 8 Department of Health Provided Services and Service Schedules..............................34
Table 9 HHS Performance Measures – Key Performance Indicators..35
Table 10 HHS Performance Measures – Safety and Quality Markers..35
Table 11 HHS Performance Measures – Outcome Indicators..35
Table 12 Statewide Health Equity Key Performance Measures...36

1. Introduction

1.1 In performing this service agreement, the Department and the Hospital and Health Service (HHS) will act consistent with the object of the *Hospital and Health Boards Act 2011* (Qld) (Act).

1.2 The Department and the HHS operate as part of a networked system and agree to work collaboratively with each other, with other HHSs and with the Queensland Ambulance Service in the best interests of the Queensland public sector health system.

1.3 The parties will ensure that planning and delivery of health services is consistent with the strategies and priorities set out by government. The parties recognise the importance of the HHS' Health Equity Strategy (as defined in the Act) and the parties' commitment to improving health and wellbeing outcomes and achieving health equity for Aboriginal and Torres Strait Islander peoples.

2. Scope

2.1 This service agreement covers the period from 1 July 2022 to 30 June 2025.

2.2 The parties have identified the services to be provided by the HHS, the funding for the provision of those services, the performance measures applicable to the services and data requirements.

3. Services

3.1 In delivering services, the HHS is required to meet:

(a) the applicable conditions of all national agreements and national partnership agreements between the Queensland Government and the Commonwealth Government and commitments under any related implementation plans; and

(b) the applicable conditions of each agreement or arrangement for funding between the Department and the HHS and commitments under any related implementation plans, which conditions and commitments may be recorded separately to this service agreement but for which funding is (or becomes) provided in Schedule 2.

3.2 The HHS is required to deliver the services outlined in this service agreement for which funding is provided in Schedule 2.

3.3 Where issues arise which prevent the HHS from providing a service or necessitate a reduction in the level or scope of a service provided, prompt notification must be made to the Department and impacted HHSs, with appropriate details. The HHS must minimise any clinical risk or adverse impact to patient experience that may result from service disruption. The Department will respond to the HHS on any requirements it has concerning service delivery and any adjustments triggered by under delivery.

3.4 If the HHS wishes to terminate or reduce service levels for a service for which funding is provided in Schedule 2, this will remain subject to negotiation and agreement by the Department at its discretion.

3.5 For any new services proposed during the term of this service agreement, the parties agree:

(a) the service must meet a demonstrated clinical need and provide value for money;

(b) the commencement of a new service, including the implementation of new models of care, may occur where the service has been commissioned by the Department, a funding stream is in place and any conditions relating to the funding have already been agreed; and

(c) if the HHS wishes to commence providing a new service that has not been commissioned by the Department, this will remain subject to agreement by the Department at its discretion.

3.6 It is acknowledged that there may, from time to time, need to be service delivery changes between HHSs. Management of inter-HHS relationships should be informed by the following principles:

(a) HHSs should maintain the proportion of out of HHS work undertaken unless as a result of agreed repatriation of patients;

(b) each HHS should manage patients from its own catchment population if it is within its clinical capability to do so as specified by the Clinical Services Capability Framework; and

(c) where it is proposed that a service move from one HHS to another, the Department will consider, as part of its review under clauses 3.4 and 3.5, whether the respective Health Service Chief Executives endorse the proposed change in patient flows; and the funding required to follow the patient.

4. Performance and Accountability Framework

4.1 The Queensland Health Performance and Accountability Framework (the Performance and Accountability Framework) sets out the framework within which the Department monitors and manages the performance of public sector health services in Queensland.

4.2 The parties will act consistent with the Performance and Accountability Framework.

5. Outcomes Framework

5.1 Queensland Health is embarking on a strategic shift in funding focus from “volume” to “outcome” using the Outcomes Framework. This approach aims to link the resources and services required and delivered as part of healthcare activities, to health outcomes for individuals and the population.

5.2 The Outcomes Framework takes a three-tiered approach:

(a) The System Tier (Tier 1), which acts as a strategic tier, and includes four domains to measure the contribution of Queensland Health to the system outcomes.

(b) The Operational Tier (Tier 2) which includes nine (9) Clinical Care Domains, reflecting areas that are important to deliver change and improvement in the short to medium term, and to operationalise the Outcomes Framework.

(c) The Tactical Tier (Tier 3) provides scaffolding to select initiatives for implementation as specific pressures arise. These pressures may include areas identified for improvement through Tier 2.

5.3 In consultation with the State-wide Clinical Networks the indicators below are under further development and shadowing.

Indicator	Care Domain	Clinical Leadership
Percentage of patients who have HBA1C ordered during hospital admission	Chronic and Complex	Diabetes Network
Time to treatment for breast, colorectal and lung cancers	Cancer Care	Cancer Care Network

5.4 Schedule 4 maps existing indicators in the Performance and Accountability Framework to the care domains of the Outcomes Framework.

6. Data supply requirements

6.1 The HHS will provide the Department with all clinical and non-clinical data that is reasonably required to support the effective management of the public sector health system. This will include, but is not limited to, data that is required to:

(a) fulfil legislative obligations;

(b) deliver accountabilities and obligations to State and Commonwealth Governments; including related to the provision and reconciliation of activity data by the Administrator of the National Health Funding Pool;

(c) monitor and support performance improvement;

(d) manage this service agreement;

(e) support clinical innovation; and

(f) facilitate evaluation and audit.

6.2 The parties agree and acknowledge that:

(a) the Department will keep the HHS informed of the Department's data requirements; and

(b) data will be provided as required, or permitted, by law.

6.3 Further details on data supply requirements, including principles that guide the collection, storage, transfer and disposal of data and prescribed timeframes for data submission, are provided online as detailed in Appendix 1.

7. Hospital and Health Service accountabilities

7.1 The HHS will perform its obligations under this service agreement.

7.2 As applicable to the HHS and its services, the HHS will comply with:

(a) legislation and subordinate legislation, including the Act;

(b) cabinet decisions;

(c) Ministerial directives;

(d) agreements entered into between the Queensland and Commonwealth governments (or agreements with others in furtherance of such agreements), of which it is informed;

(e) agreements entered into between the Department and other Queensland Government entities, of which it is informed;

(f) agreements entered into with another HHS(s), including Networked Services Agreements;

(g) all industrial instruments;

(h) all health service directives and health employment directives; and

(i) all policies, guidelines, and implementation standards, including human resource policies.

7.3 As part of the commitment to achieving First Nations health equity, the HHS will prioritise the elimination of racial discrimination and institutional racism within its service and ensure that Aboriginal and Torres Strait Islander peoples have access to culturally safe and responsive health services.

7.4 The HHS will ensure that effective health service planning and delivery systems are in place, working in collaboration with the Department.

7.5 To support the achievement of the Queensland-Commonwealth Partnership's (QTP's) vision and commitment to work together to tackle health system challenges that cannot be overcome by any one organisation, HHSs are required to prepare and submit Joint Regional Needs Assessments in accordance with the framework provided online as detailed in Appendix 1.

7.6 HHSs must operate clinical service delivery consistent with the National Quality and Safety Standards. The HHS is expected to escalate any concerns that arise at the conclusion of a formalised assessment.

7.7 The HHS will ensure that health service employees employed by the Chief Executive[1] who perform work for the HHS are managed in accordance with any applicable delegations and directions from the Chief Executive. The HHS will ensure that effective asset management systems are in place (available online, as detailed in Appendix 1), that comply with the *Queensland Government Building Policy Framework and Guideline*, while working in collaboration with the Department.

7.8 The HHS will maintain accreditation to the standards required by the Department.

7.9 The HHS will appropriately perform and fulfil its functions under the Act.

[1] In this service agreement the term Chief Executive takes the meaning applied in the Act, which is the Chief Executive of Queensland Health, but which is generally referred to as the Director-General.

7.10 The HHS will provide to the Chief Executive reports of a type, and at the intervals, agreed between the parties, or as reasonably specified by the Chief Executive.

8. Department accountabilities

8.1 The Department will perform its obligations under this service agreement including, in return for the HHS performing its obligations and delivering the services, providing funding to the HHS as stipulated in this service agreement (as amended).

8.2 The Department will:

(a) comply with applicable legislation and subordinate legislation, including the Act, as relates to this service agreement;

(b) perform the system manager role (as defined in the Act) through the Chief Executive; and

(c) provide a range of services to the HHS as set out in Schedule 3.

8.3 The Chief Executive will appoint health service employees to perform work for the HHS for the purpose of enabling the HHS to perform its functions under the Act.

8.4 The Chief Executive will consult, cooperate, and coordinate with the HHS to ensure legal compliance with the *Work Health and Safety Act 2011* and other legislation as it applies to the scope, nature and location of operations associated with this service agreement.

8.5 The Chief Executive will appropriately perform and fulfil their functions under the Act.

9. Achieving health equity with First Nations Queenslanders

9.1 Through legislative amendments to the Act and the Hospital and Health Boards Regulation 2012, and the release of *Making Tracks Together: Queensland's Aboriginal and Torres Strait Islander Health Equity Framework* (2021), Queensland Health has strengthened its commitment to improving health and wellbeing outcomes and achieving health equity with First Nations peoples.

9.2 The HHS will develop and resource a First Nations Health Equity Strategy, compliant with legislative requirements. An implementation plan, accompanying the strategy, demonstrates the HHS's activities and key performance measures to achieve health equity with First Nations peoples. The Health Equity Strategy will act as the principal accountability mechanism between the Aboriginal and Torres Strait Islander community and the HHS in achieving health equity with First Nations Queenslanders and forms a key part of the Queensland Government's commitment to the *National Agreement on Closing the Gap* (2020).

9.3 The HHS is required to review the Health Equity Strategy at least once every three years and will publish the Health Equity Strategy in a way that allows it to be accessed by members of the public.

9.4 The HHS will ensure that commitment and leadership is demonstrated through implementing the actions and achieving the key performance measures outlined in the Health Equity Strategy.

9.5 The HHS will report publicly every year on progress against the Health Equity Strategy.

9.6 The HHS will support the implementation of other supplementary policies and strategies to drive health equity across the public health system, including relevant election commitments.

9.7 The HHS will participate as a partner in the implementation and achievement of Queensland's *HealthQ32 First Nations First Strategy 2032* in addition to HHS commitments within their Health Equity Strategy.

10. Dispute Resolution

10.1 Where a dispute arises in connection to this agreement, either between the department and one or more HHSs or between HHSs, every effort should be made to resolve the dispute at the local level. If local resolution cannot be achieved, the dispute resolution processes, accessible through Appendix 1, must be followed.

11. General

11.1 **Sub-contracting**

(a) The HHS must have appropriate systems in place to ensure that any subcontractor is accredited (as applicable), qualified, and otherwise fit to perform any services for which it is contracted.

(b) The HHS must ensure that any sub-contractor who has access to confidential information (as defined in the Act) or personal information (as defined in the Information Privacy Act 2009 (Qld)) complies with obligations no less onerous than those imposed on the HHS.

11.2 **Insurance**

The HHS must:

(a) hold and maintain the types and levels of insurances that the HHS considers appropriate according to its functions and obligations; and

(b) comply with reasonable requests or directions from the Department in this regard.

11.3 **Amendment**

The process for amending this service agreement is provided for under the Act and further outlined in Schedule 5.

12. Counterparts

12.1 This service agreement may be executed in two or more identical copy counterparts, each of which together will be deemed an original, but all of which together will constitute one and the same instrument.

12.2 In the event that any signature executing this service agreement or any part of this service agreement is delivered by facsimile transmission or by scanned e-mail delivery of a '.pdf' format data file or equivalent of the entire agreement, the signature will create a valid and binding obligation of the party executing (or on whose behalf the signature is executed) with the same force and effect as if the signature page were an original. For the avoidance of doubt, this service agreement may be in the form of an electronic document and may be electronically signed.

12.3 For execution under this clause 12 to be valid, the entire service agreement upon execution by each individual party must be delivered to the remaining parties.

Execution

The terms of this Service Agreement were agreed under the provisions set out in the *Hospital and* Health *Boards Act,* section 35 on 29 June 2022, and were subsequently amended by the Deeds of Amendment entered into pursuant to section 39 of the *Hospital and Health Boards Act 2011* and executed on 10 January 2023, 28 April 2023, 3 August 2023, 22 December 2023, 5 April 2024, 17 July 2024 and 11 December 2024.

This revised Service Agreement consolidates amendments arising from:

- Periodic Adjustment – COVID-19 Funding Transfer September 2022
- Periodic Adjustment – COVID-19 Funding Transfer October 2022
- 2022/23 Amendment Window 2 (in year variation)
- Periodic Adjustment – COVID-19 Funding Transfer December 2022
- 2022/23 Amendment Window 3 (in year variation)
- Periodic Adjustment – COVID-19 Funding Transfer April 2023
- Extraordinary Amendment Window – May 2023
- 2023/24 Amendment Window 1 (Budget Build)
- 2023/24 Amendment Window 2 (in year variation)
- 2023/24 Amendment Window 3 (in year variation)
- 2024/25 Amendment Window 1 (Budget Build)
- 2024/25 Amendment Window 2 (in year variation)

Schedule 1 HHS profile

1. HHS profile

This Schedule does not apply to this HHS.

Schedule 2 Funding and purchased activity and services

This Schedule 2 sets out:

(a) the services which are to be provided by the HHS;

(b) the activity purchased by the Department from the HHS;

(c) the funding provided for delivery of the purchased activity;

(d) the allocation of funding provided against the care domains of the Outcomes Framework;

(e) the criteria and processes for financial adjustments associated with the delivery of purchased activity and specific funding allocations; and

(f) the sources of funding that this service agreement is based on and the way these funds will be provided to the HHS.

1. Introduction

1.1 The HHS will deliver the services for which funding is provided in this Schedule 2. In providing these services, the HHS will ensure that:

(a) all statewide and national policy frameworks, guidelines, protocols and implementation standards applicable to the service provided are followed;

(b) participation in national programs is facilitated and supported, including where these programs are provided by the Commonwealth Government;

(c) service delivery partnerships, including with other HHSs, primary care organisations and non-government organisations, are maintained and operate effectively;

(d) collaboration and engagement with other service providers and stakeholders is initiated and maintained to ensure that an integrated system of treatment, care and support is in place and to facilitate the delivery of comprehensive and effective services. This may include but is not limited to:

(i) other HHSs;

(ii) non-government organisations;

(iii) Aboriginal and Torres Strait Islander community-controlled health organisations;

(iv) Queensland Ambulance Service;

(v) services provided through the Department of Health (for example, Pathology Queensland);

(vi) primary care providers;

(vii) other government departments and agencies; and

(viii) private providers;

(e) models of care and service delivery arrangements are consistent with evidence-based practice and offer value for money;

(f) services are provided on an equitable basis to the community, and processes are in place to ensure that services reach identified target populations, high risk groups and hard to reach communities;

(g) referral networks and pathways continue to operate effectively; and

(h) innovation and continuous improvement are supported.

2. Purchased health services

2.1 Table 4 shows the allocation of funding from the Department to the HHS across the care domains of the Outcomes Framework. Table 5, Table 6, and Table 7 outline the activity and service streams which the Department agrees to purchase from the HHS pursuant to this service agreement.

2.2 More generally, this will include the following:

2.3 **Statewide Services**

(a) The designation of a service as a statewide service (either a clinical statewide service or a clinical support statewide service) will be determined by the Department, consistent with the stipulated governance arrangements for such services.

(b) The HHS will:

(i) collaborate with the Department and other HHSs in the implementation of, and adherence to, the governance and oversight arrangements for statewide services;

(ii) participate in, and contribute to, the staged review of the purchasing model for identified statewide services; and

(iii) ensure that referral pathways in and out of each statewide service are followed.

2.4 **Clinical Statewide Services and Clinical Support Statewide Services provided**

This clause does not apply to this HHS.

Table 1 Statewide Services

This table does not apply to this HHS.

2.5 **Regional services**

This clause does not apply to this HHS.

2.6 Prevention services and public health services

(a) The HHS will provide a range of prevention and public health services to promote and protect health, prevent illness and disease, and manage risk, including:

(i) Specialist Public Health Units

(ii) environmental health services, including risk assessment, regulation and enforcement in relation to environmental hazards, food safety, medicines and therapeutic goods, mosquitos and other vectors, pest management, poisons, radiation safety, chemical safety and water quality;

(iii) communicable disease services including immunisation, blood-borne viruses, sexually transmissible infections, infection control, notifiable conditions, mosquito-borne disease and tuberculosis;

(iv) management of incidents, emergencies and disasters, and disease outbreak readiness and response services;

(v) preventive health services;

(vi) population health screening including, but not limited to, cancer screening services and newborn blood spot screening;

(vii) public health epidemiology and surveillance;

(viii) mitigation and adaptation in response to climate risks.

(b) Services will be provided in line with public health related legislation and the service delivery and reporting requirements outlined in the Public Health Service Schedule and supported by the *Public Health Practice Manual*, as these relate to the services provided.

(c) Delivery of these services may be coordinated through specialist public health units, sexual health services, tuberculosis services, other areas of the HHS, or a combination of these.

2.7 Aboriginal and Torres Strait Islander health services

The HHS will provide Aboriginal and Torres Strait Islander specific health services and initiatives consistent with the principles and objectives of the Queensland Government's *Making Tracks toward closing the gap in health outcomes for Indigenous Queenslanders by 2033 – Policy and Accountability Framework* and the priorities committed to in the HHS's Health Equity Strategy. These services and initiatives will be delivered in line with guidance from the First Nations Health Office and the *First Nations First Strategy 2032.*

2.8 Mental health, alcohol, and other drugs services

The HHS will provide treatment, care and support for individuals who are, or may be, experiencing substance use disorders and/or other mental health disorders, mental health crisis and suicidal distress, and their families and carers. Services will be delivered in line with guidance from the Mental Health, Alcohol and Other Drugs Strategy and Planning Branch.

2.9 **Oral health services**

The HHS will provide oral health services to people who meet the eligibility criteria for accessing public dental services in Queensland. Services will be delivered in line with guidance from the Office of the Chief Dental Officer.

2.10 **Prisoner health services**

This clause does not apply to this HHS.

2.11 **Youth detention services**

This clause does not apply to this HHS.

2.12 **Refugee health**

This clause does not apply to this HHS.

2.13 **State-funded outreach services**

(a) Where state-funded outreach services are provided:

(i) funding for the service will remain part of the providing HHS's funding allocation; and

(ii) the activity must be recorded at the HHS where the outreach service is being provided.

(b) Any changes to the provision of outreach services will follow the requirements set out in clause 3 of this service agreement.

3. Teaching, training, and research

The HHS will provide the teaching, training and research programs for which funding is provided within Schedule 2 and as described below.

3.1 **Clinical education and training**

(a) The HHS will provide education and training placements for the following professional groups consistent with and proportionate to the capacity of the HHS and will support and align with stipulated placement terms governing clinical placements in Queensland Health facilities:

(i) medical students;

(ii) nursing and midwifery students;

(iii) pre-entry clinical allied health students;

(iv) interns;

(v) rural generalist trainees;

(vi) vocational medical trainees;

(vii) first year nurses and midwives;

(viii) re-entry to professional register nursing and midwifery candidates;

(ix) dental students;

(x) allied health rural generalist training positions;

(xi) Aboriginal and Torres Strait Islander Health Workers and Aboriginal and Torres Strait Islander Health Practitioners.

(b) The HHS will comply with the state-wide vocational medical training pathways.

(c) The HHS will support profession specific and inter-profession statewide allied health clinical education programs.

(d) The HHS will continue to implement and retain the following positions provided through clauses to the *Health Practitioners and Dental Officers (Queensland Health) Certified Agreement (No 4) 2022*:

(i) health practitioner research positions provided through the Research Package for Health Practitioners; and

(ii) clinical educator positions provided through the Clinical Education Management Initiative for Health Practitioners.

(e) The HHS will maintain or increase its contribution of staff to the Queensland Country Relieving Doctors and the receiving HHS will be responsible for wages, clinical governance, and appropriate supervision of the junior medical relievers.

3.2 **Statewide training, education, and research**

This clause does not apply to this HHS.

3.3 **Health and medical research**

The HHS will:

(a) develop and implement a strategy to drive increased research activity and its translation into clinical practice;

(b) support increased and equitable access to clinical trials for patients;

(c) support clinicians to undertake research linked to their practice; and

(d) ensure high quality and timely research governance approval processes.

4. Delivery of purchased activity

4.1 The HHS is required to maintain accurate activity forecasts in the purchased target module of the Decision Support System (DSS) at all times. This information is imperative to the Department's assessment of State performance against the national Soft Cap and for outer-year planning. Activity forecasts must accurately reflect financial forecasts reported to the Finance Branch monthly.

4.2 The Department and the HHS will monitor actual activity against purchased levels and will act as necessary to ensure delivery of purchased levels is achieved. The HHS has a responsibility to only recognise revenue that is linked with actual activity delivered against purchased volumes.

4.3 The HHS will actively monitor variances from purchased activity levels and notify the Department as soon as the HHS becomes aware of significant variances.

4.4 The HHS will also notify the Department of deliberate changes to the consistent recording of activity within year that would result in additional activity being recorded for existing health services.

4.5 The HHS will undertake regular quality audits. The HHS is encouraged to publish its data quality framework describing audits undertaken and results achieved. For further information, refer to the Delivery of Purchased Activity – Requirement for Quality Audits specification sheet as detailed in Appendix 1.

4.6 If the HHS wishes to convert activity between purchased activity types, programs, and levels the HHS must negotiate this with the Department based on a sound needs based rationale.

4.7 The Department will reconcile in-scope activity, as defined in the Activity Reconciliation specification sheet (available online, as detailed in Appendix 1), delivered by the HHS against the purchased in-scope activity targets outlined in Table 5.

4.8 Activity reconciliations will be undertaken in the applicable End of Year Technical Amendment Window and subsequent Amendment Window 2 and will be derived through application of the methodology which is documented in the Activity Reconciliation specification sheet.

4.9 Should the HHS be unable to deliver the activity that has been funded a financial adjustment will be applied.

4.10 Funding and corresponding activity that is withdrawn from the HHS may be reallocated to an alternate provider that can undertake the activity.

4.11 Any resulting changes to funding and purchased activity levels will be actioned through the processes outlined in Schedules 2 and 5 of this service agreement.

4.12 The HHS will maintain its efforts to accurately record the delivered activity and submit this data to the Department in accordance with the agreed requirements.

5. Delivery of purchased services

5.1 As part of the service agreement, the Department purchases a range of services (or deliverables) from the HHS for the delivery of certain programs and projects.

5.2 These program or project services may be the subject of separately agreed conditions tied to that funding and the focus of detailed monitoring by the Department.

5.3 Conditions may include, but are not limited to:

(a) establishment and/or commencement of services;

(b) delivery of activity;

(c) workforce obligations;

(d) establishment of oversight committees;

(e) opening or upgrades to facilities;

(f) program evaluation;

(g) program management;

(h) reporting or notification obligations; and

(i) attainment of performance standards.

5.4 The HHS will ensure that the conditions are achieved within the stipulated time period.

5.5 The HHS will notify the Department if the HHS forecasts an inability to achieve program or project objectives or the conditions.

5.6 The Department may withdraw allocated funding pro rata to the level of under delivery if the services for a specified program or project are not being fully delivered according to the objectives or conditions.

5.7 Any resulting changes to funding and purchased activity levels will be actioned through the processes outlined in Schedules 2 and 5 of this service agreement.

6. Financial adjustments

6.1 Activity targets

(a) The Department will initiate a joint process with the HHS to determine whether a financial adjustment should be applied in relation to any purchased activity which has breached the thresholds identified bi-annually. This process will take into account any relevant matters that have been identified in a review/analysis of the breach as well as the outcomes of the activity plan implemented to address the activity breach.

(b) Activity will be monitored at the purchasing hierarchy level. Providing the HHS meets all relevant KPIs and specific funding allocations, the HHS has the ability to negotiate the transfer of activity across the purchasing hierarchy with the Department.

(c) Table 2 demonstrates the financial adjustment that will be applied when activity thresholds have been breached.

(d) The HHS may not utilise the provisions within AASB15 Revenue from Contracts with Customers to override the application of any financial adjustment made by the Department in line with Table 2.

Table 2 Financial adjustments applied on breach of activity thresholds

Example of Breach	Description	Financial Adjustment
Over performance	Activity exceeds that specified in the service agreement value for in-scope activity as shown in Table 5.	Purchasing contracts are capped and an HHS will not be paid for additional activity apart from activity that is in scope for the identified purchasing incentives as set out in Table 3 (where applicable.)
Under performance	Activity is below that specified for in-scope activity as shown in Table 5.	Purchased activity and the related funding will be withdrawn at 100% of the Queensland Efficient Price and reallocated to an alternate provider that can undertake the activity. The reconciliation will be undertaken as outlined in the Activity Reconciliation Specification. Refer to Table 5 for the HHS QWAU target.
Failure to deliver on service commitments linked to specific funding allocations	Specific funding allocations<br>National Partnership Agreements.	It is at the discretion of the Department to withdraw allocated funding pro rata to the level of under delivery.
For all other types of activity variance, any financial adjustment will be made at the discretion of the Department.		

6.2 Purchasing approach

(a) The purchasing approach includes a range of funding adjustments (purchasing incentives and ABF model localisations) that aim to incentivise high quality and high priority activity, support innovation and evidence-based practice, deliver additional capacity through clinically and cost-effective models of care and dis-incentivise care providing insufficient or no benefit for patients. This includes incentive payments for HHSs who achieve targets in specific priority areas. The funding adjustments are detailed in Table 3. The Department must reconcile the applicable funding adjustments in Table 3 in line with the timeframes specified in the relevant purchasing specification sheet. The Department must provide a copy of the reconciliation statement to the HHS.

(b) Funding adjustments must be based on the requirements contained in the relevant purchasing specification sheet.

(c) Any resulting changes to funding and purchased activity levels will be actioned through the processes outlined in Schedules 2 and 5 of this service agreement.

Table 3 Funding adjustments 2024/25

Funding adjustment	
**Purchasing incentives**	
Models of care/workforce	This program includes a range of initiatives focusing on incentivising:<br>• specific models of care; and<br>• the use of workforce operating at top of scope where there may be long wait lists and staff have not been available in a traditional model of care.
**ABF model localisations**	
Child Health Checks	QWAU loading for every in-scope check performed.
Unqualified neonate funding	Reduced Diagnosis Related Group (DRG) QWAU for all maternal delivery episodes with a liveborn outcome, discounted by the Diagnosis Related Group (DRG), with QWAUs re-allocated for unqualified neonates.
Maternity care for First Nations women	QWAUs to incentivise maternity care provided to First Nations mothers during pregnancy and to incentivise smoking cessation during pregnancy.
Sentinel events	Payment withdrawn for sentinel events as per the national ABF model.
Advance Care Planning (ACP)	QWAUs for HHSs offering ACP discussions to admitted patients, non-admitted outpatients, community health patients and Emergency Department patients.
Emergency Department Did Not Wait (DNW)	Zero QWAUs for DNWs.
Fractured neck of femur	QWAUs reduced by 20% for non-timely surgical treatment of fractured neck of femur.
Hospital in The Home (HITH)	QWAUs increased by 12.5% for Hospital in the Home (HITH) admissions of Residential Aged Care Facility residents.
Out-of-scope services	Nil QWAUs for out-of-scope procedures.
Pre-operative bed days	QWAUs for long stay days above the upper trim point less ICU days reduced equivalent to pre-operative days, up to a maximum of 3 days, for elective episodes.
Smoking cessation (community mental health)	QWAUs for smoking cessation activity for community mental health patients.
Smoking cessation (inpatients)	QWAUs for smoking cessation activity for publicly funded inpatients.
Stroke care	10% QWAU loading for acute stroke patients admitted to Queensland Stroke Clinical Network-endorsed stroke unit care.
Telehealth (admitted patients)	QWAUs for provider-end of in-scope admitted patient telehealth activity.
Allied Health Led Workforce for Pelvic Health and Gastroenterology	QWAU loading for an in-scope service event for Pelvic Health and Gastroenterology recorded against an Other Health Professional.
Remote Patient Monitoring	QWAU loading for an in-scope non-admitted remote patient monitoring encounter per month per patient.

Surgery Connect reimbursements

(a) The HHS will reimburse the Department for the actual costs of activity for nominated patient referrals to the Surgery Connect program where:

(i) The HHS has nominated the patient referral as HHS funded or HHS Direct on entry of the referral in the Surgery Connect Activity Navigator (SCAN); and

(ii) The HHS Chief Finance Officer has recorded approval of the nomination in SCAN;

or

(iii) The HHS has obtained the Department's agreement to retrospectively convert a defined patient cohort to HHS funded status in SCAN.

(b) The HHS may only request retrospective conversion of activity to HHS funded within the financial year in which the activity has taken place.

(c) Any resulting changes to funding and purchased activity levels will be actioned through the processes outlined in Schedules 2 and 5 of this service agreement.

6.3 **Financial adjustments – other**

(a) Notwithstanding the provisions regarding the recognition of revenue as stipulated in AASB1058 Income of Not-for-Profit Entities and/or AASB15 Revenue from Contracts with Customers, the Department may seek to recover funding from an HHS that was provided through this service agreement which has:

(i) not been utilised in accordance with its intended purpose; and/or

(ii) not been utilised within the prescribed time period to deliver the agreed outcomes/services.

(b) If the Chief Executive (or delegate) determines that previously allocated funding is to be recovered, any resulting changes to funding and purchased activity levels will be actioned through the processes outlined in Schedules 2 and 5 of this service agreement.

6.4 **Public and private activity/own source revenue**

(a) Own source revenue comprises grants and contributions, user charges and other revenues.

(b) Where an HHS is above its own source revenue target, it will be able to retain the additional own source revenue with no compensating adjustments to funding from other sources where the additional revenue is not attributable to a private patient

consistent with clauses A9 to A13 and A44 of the *National Health Reform Agreement.*

(c) Where an HHS is below its own source revenue target in respect of private patients, it will experience a reduction in revenue with no compensating adjustments to funding from other sources.

(d) The HHS will make all efforts to identify and maximise revenue from appropriate third parties to ensure optimisation of available State and Commonwealth funding across the Queensland public sector health system.

(e) The own source revenue identified in Table 5 is an estimate generated by the HHS which allows all third-party funding sources associated with service delivery to be identified. The HHS will ensure that this estimate is substantiated and accurate to ensure no significant variances to actual revenue generated.

(f) The HHS will routinely revise and update the estimate to ensure alignment between the service agreement and Queensland Treasury's reporting system (TRIDATA).

(g) Budget adjustments for changes in own source revenue from private patients will be actioned through the processes set out in Schedule 5 of this service agreement.

7. Funding sources

7.1 The four main funding sources contributing to the HHS service agreement value are:

(a) Commonwealth funding;

(b) State funding;

(c) grants and contributions; and

(d) own source revenue.

7.2 Table 5 provides a summary of the funding sources for the HHS and the total value of the service agreement.

7.3 The HHS must undertake regular quality audits to check for potential duplicates in funding source, in particular the National Health Reform Agreement and Medicare given the Commonwealth's contribution to both funding sources. The HHS should take active steps to remedy areas of concern. A consumer's choice of funding arrangement should be reflected on a patient election form.

8. Funds disbursement

8.1 The Department agrees to pay the HHS the amount described in Table 5 of Schedule 2, subject to:

(a) parliamentary appropriation and adequate funds being allocated to the Department; and

(b) the terms of this service agreement.

8.2 All payments under this service agreement will be made in accordance with the requirements of the *National Health Reform Agreement* and the Act.

8.3 The Chief Executive will direct the disbursement of both State and Commonwealth funding from the State Pool Account, the State Managed Fund, and the Department of Health Expenditure account to the HHS.

8.4 However, the State (represented by the Chief Executive) will not:

(a) redirect Commonwealth payments between HHSs;

(b) redirect Commonwealth payments between funding streams (e.g., from ABF to block funding); and/or

(c) adjust the payment calculations underpinning the Commonwealth's funding.

8.5 The Department will pay state-funded activity-based funding, block funding and system manager funding to the HHS on a fortnightly basis in line with receipt of the State Appropriation Payment. The payment made will be equal to 1/26th of the state-funded component of the service agreement value described in Table 5.

8.6 The Department will pay Commonwealth-funded activity-based funding and block funding monthly in line with receipt of the Commonwealth payment. The payment made will be equal to 1/12th of the Commonwealth funded component of the service agreement value described in Table 5.

8.7 Where the parties have agreed to amend the service agreement value or the Commonwealth contribution amount changes, the fortnightly and/or monthly payments will be adjusted to reflect the amended service agreement value.

8.8 Service agreement payments may be made outside of these timeframes with the approval of the Department's Chief Finance Officer.

Table 4 HHS Funding by Outcomes Framework Care Domain 2024/25

Care Domain	Funding $	QWAU (Q27)
Prevention, early intervention, and primary health care	$84,180,239	6,372
Trauma and illness	$204,470,989	31,685
Mental health and alcohol and other drugs	$52,926,868	7,242
Cancer	$39,433,430	6,038
Planned care	$92,723,970	14,499
Maternity and neonates	$37,687,034	5,991
Chronic and complex	$158,308,185	22,924
Statewide services	$2,253,709	45
Depreciation	$35,198,000	0
**TOTAL**	**$707,182,424**	**94,796**

Table 5 HHS Total Funding Allocation by Funding Source 2024/25

Funding Source	24-25 NWAU (N2425)	24-25 QWAU (Q27)	24-25 Agreed ($)
**NHRA Funding**			
**ABF Pool**			
**ABF Funding (In scope NHRA)**[2]			
Commonwealth	75,894		$190,524,137
State		75,848	$241,571,332
State Specified Grants			$8,174,787
State-wide Services			$0
Restoring Planned Care	824	830	$4,880,000
Long Stay Patient Recovery Funding	492	493	$6,560,000
**Total ABF Funding (in scope NHRA)**	**77,209**	**77,171**	**$451,710,256**
**State Managed Fund**			
**Block Funding (State and Commonwealth)**			
Small rural hospital		5,670	$42,951,231
Teaching, Training and Research			$16,673,699
Other Mental Health	3,118	3,118	$23,360,053
Non-Admitted Home Ventilation			$0
Residential Mental Health Services		288	$0
Other Non-admitted Service			$0
Highly Specialised Therapies			$0
Other Public Hospital Programs			$0
**Total NHRA Funding**	**77,209**	**86,247**	**$534,695,238**
**Out of Scope NHRA**			
**Queensland ABF Model**			
DVA		477	$2,800,879
NIISQ/MAIC		89	$524,516
Oral Health		1,155	$7,750,626
Oral Health – FFA		0	$0
BreastScreen		303	$2,330,258
Child Health Checks		43	$801,317
**Total Queensland ABF Funding**		**2,067**	**$14,207,597**
**Discretely Funded Programs**[3]			
Department of Health			$61,979,157
Locally Receipted Funds			$5,893,409
Research (Other OSR)			$4,745
**Total Discretely Funded Programs**			**$67,877,311**

[2] The split between Commonwealth and State NHRA funding will change during the year as the purchased activity targets are updated and the National Health Funding Board updates their payment advice accordingly.

[3] Includes all other (non-ABF) State and Commonwealth funded health services including, but not limited to, Prisoner Health, Public Health and Prevention Services.

Funding Source	24-25 NWAU (N2425)	24-25 QWAU (Q27)	24-25 Agreed ($)
**Own Source Revenue**			
Private Patient Admitted Revenue[4]	2,025	1,997	$11,737,108
Pharmaceuticals Benefits Scheme		1,949	$18,635,149
Non-Admitted Services		1,796	$3,684,550
Other Activities[5]		740	$9,664,011
Oral Health – CDBS		0	$761,493
**Total Own Source Revenue**	**-**	**6,482**	**$44,482,311**
Locally Receipted Funds (exc. Discretely Funded Programs)[6]			$10,721,968
Depreciation			$35,198,000
**GRAND TOTAL**	**77,209**	**94,796**	**$707,182,424**

Pool Accounts			
ABF Pool (National Health Funding Pool)[7]			$465,917,852
State Managed Fund[8]			$82,984,983
System Manager			$61,979,157

[4] The estimated value of the revenue earned from private patients, based on OSR estimates provided by the HHS.
[5] Incorporates all OSR which is not identified elsewhere in Table 5.
[6] Includes items such as training programs and donations. Does not include locally receipted funds associated with discretely funded programs, e.g.Transition Care.
[7] Articulates the financial payment made to support in-scope ABF services under the NHRA including DVA, NIISQ, MAIC and BreastScreen Services. Applies to all HHSs except Central West HHS and Torres and Cape HHS.
[8] Articulates the payment made for block funded services under the NHRA, DVA, NIISQ and MAIC services.

Table 6 National Health Reform Funding

NHRA Funding Type	No. of In-scope services (NWAU)	No. of Out-of-scope services (QWAU)	Total Services (WAU)	ABF NEP ($)	State Price ($)	Funding for In-scope services at NEP ($)	Cwlth contribution for In-scope services ($)	State contribution for In-scope services ($)	State contribution for Out-of-scope services ($)	Total Cwlth and State contribution ($)
National Efficient Price (NEP)		*a,b*		*c*	*d*			*e*		
**ABF Allocation (NWAU)**										
Emergency Department	14,365	105	14,470	$6,465	$5,878	92,867,951	36,061,094	47,270,222	799,191	84,130,508
Acute Admitted	43,772	1,054	44,827	$6,465	$5,878	282,987,264	109,885,384	144,041,844	8,008,868	261,936,096
Admitted Mental Health	3,676	0	3,676	$6,465	$5,878	23,766,351	9,228,594	12,097,184	0	21,325,778
Sub-Acute	4,742	592	5,334	$6,465	$5,878	30,658,654	11,904,910	15,605,398	4,498,180	32,008,488
Non-Admitted	9,339	1,625	10,964	$6,465	$5,878	60,375,611	23,444,155	30,731,469	12,341,357	66,516,981
**Total ABF Allocation**	**75,894**	**3,377**	**79,271**			**490,655,831**	**190,524,137**	**249,746,119**	**25,647,597**	**465,917,852**
**Block Allocation**										
Teaching, Training, and Research						0	4,430,438	12,243,261	0	16,673,699
Small and Rural Hospitals[9]						0	12,340,010	30,611,221	0	42,951,231

[9] Incorporating small regional and rural public hospitals, four specialist mental health facilities (Baillie Henderson Hospital, Jacaranda Place – Queensland Adolescent Extended Treatment Centre, The Park – Centre for Mental Health and Kirwan Rehabilitation Unit) and the Ellen Barron Family Centre.

NHRA Funding Type	No. of In-scope services (NWAU)	No. of Out-of-scope services (QWAU)	Total Services (WAU)	ABF NEP ($)	State Price ($)	Funding for In-scope services at NEP ($)	Cwlth contribution for In-scope services ($)	State contribution for In-scope services ($)	State contribution for Out-of-scope services ($)	Total Cwlth and State contribution ($)
Other Mental Health						0	8,707,016	14,653,037	0	23,360,053
Non-Admitted Home Ventilation						0	0	0	0	0
Other Non-Admitted Services						0	0	0	0	0
Other Public Hospital Programs						0	0	0	0	0
Highly Specialised Therapies						0	0	0	0	0
**Total Block Allocation**						**0**	**25,477,464**	**57,507,519**	**0**	**82,984,983**
**Grand Total Funding Allocation**										**548,902,835**

Notes

a. QWAU refers to Queensland Weighted Activity Units in Q27 phase (built on N2425)
b. DVA, NIISQ/MAIC, Oral Health, Child Health Checks and BreastScreen
c. Queensland Efficient Price used to Purchase growth QWAUs
d. NWAU x NEP
e. State funding transacted through the Pool/State Managed Fund Account; not covered under the NHRA
- NWAU estimates do not take account of cross-border activity.

Table 7 Discretely Funded Programs (Non-ABF)

Discretely Funded Programs	Revenue Models	$
Aged Care Assessment Program	Commonwealth	$649,074
Alcohol, Tobacco and Other Drugs	State	$4,424,701
Community Health Programs	State	$32,914,485
Interstate Patients (QLD Residents)	State	$1,886,944
Other State Funding	State	$0
Patient Transport: PTSS	State	$11,046,505
Patient Transport: Aeromedical Retrieval	State	$1,763,939
Patient Transport	State	$0
Prevention Services and Public Health	Commonwealth	$2,033,543
	State	$298,173
Prisoner Primary Health Services	State	$0
Disability Residential Care Services	State	$0
Multi-Purpose Health Services	Commonwealth	$4,668,172
Residential Aged Care Services	Commonwealth	$140,420
	Locally Receipted Funds	$0
	State	$1,458,456
Transition Care	Locally Receipted Funds	$1,383,700
	State	$694,745
Research	Commonwealth	$0
	OSR	$4,745
Home and Community Care (HACC) Program	Locally Receipted Funds	$4,509,709
**Discretely Funded Programs Total**		**$67,877,311**
**TOTAL**		**$67,877,311**

Schedule 3 Department of Health Provided Services

1. In scope services and service schedules

Table 8 Department of Health provided services and service schedules

Provider	Service provided	Link to Service Statement
Corporate Services Division (CSD)	• Corporate Enterprise Solutions<br>• Finance Branch:<br>o Accounts Payable Service Provision<br>o Banking and Payment Services<br>• Central Pharmacy<br>• Group Linen Services<br>• Transport and Logistic Services<br>• Supply Chain Services	CSD Service Schedules
eHealth Queensland (eHQ)	• ICT Service	eHQ Service Schedule
Queensland Public Health and Scientific Services Division (QPHaSS)	• Pathology Queensland<br>• Biomedical Technical Services<br>• Public Health Services	QPHaSS Service Schedules

Schedule 4 Performance Measures

1. Performance Measures

1.1 The performance of the HHS will be measured according to the assessment criteria and processes described in the Performance and Accountability Framework.

1.2 Existing performance indicators are mapped to the care domains of the Outcomes Framework.

1.3 The detailed specification for each of the performance measures listed in this service agreement are provided through performance measure attribute sheets.

1.4 The performance measures identified in this service agreement are applicable to the HHS unless otherwise specified within the attribute sheet.

1.5 HHSs are also required to report against the agreed Statewide Health Equity Key Performance Measures (Table 12).

Table 9 HHS Performance Measures – Key Performance Indicators

Outcomes Framework Care Domain	Key Performance Indicators	Indicator Number
Chronic and complex	Hospital Acquired Complications (IHACPA code 8, 11, 13, 14)	31
Chronic and complex	Potentially Preventable Hospitalisations – First Nations Peoples:<br>• Diabetes complications<br>• Selected conditions	 37a<br>37b
Chronic and complex	Potentially avoidable deaths – First Nations Peoples	70
Maternity and neonates	Hospital Acquired Complications (IHACPA code 15,16)	31
Maternity and neonates	Proportion of healthy birthweight babies (2,500 grams to 4,499 grams at birth) – Aboriginal and Torres Strait Islander babies	71
Mental health, alcohol, and other drugs	Face to face community follow up within 1-7 days of discharge from an acute mental health inpatient unit	26
Mental health, alcohol, and other drugs	Proportion of mental health and alcohol and other drug service episodes with a documented care plan	27
Mental health, alcohol, and other drugs	Suicide count and rate – First Nations Peoples	72
Other	Average sustainable Queensland Health FTE	50
Other	Capital expenditure performance	51
Other	Forecast operating position:<br>• Full year<br>• Year to date	 48<br>49
Planned care	Category 1 elective surgery patients treated within the clinically recommended timeframe	7
Planned care	Elective surgery patients waiting longer than the clinically recommended timeframe	9
Planned care	Proportion of overnight inpatients discharged by 10am	12

Outcomes Framework Care Domain	Key Performance Indicators	Indicator Number
Planned care	Category 4 gastrointestinal endoscopy patients treated within the clinically recommended timeframe	13
Planned care	Gastrointestinal endoscopy patients waiting longer than the clinically recommended timeframe	16
Planned care	Category 1 patients who receive their initial specialist outpatient appointment within the clinically recommended timeframe	17
Planned care	Patients waiting longer than clinically recommended for their initial specialist outpatient appointment	19
Planned care	Telehealth utilisation rates:<br>Number of non-admitted telehealth service events	20
Planned care	Hospital Acquired Complications (IHACPA code 1,2,3,4,6,7,9,10,12)	31
Planned care	Missed Opportunity to Treat – Outpatients	73
Prevention, early intervention, and primary health care	Access to oral health services (adults)	21
Prevention, early intervention, and primary health care	Access to oral health services (children)	67
Prevention, early intervention, and primary health care	Potentially avoidable deaths – First Nations Peoples	70
Prevention, early intervention, and primary health care	Suicide count and rate – First Nations Peoples	72
Trauma and illness	Hospital Access Target (Admitted patients)<br>• % of emergency stays within 4 hours	1
Trauma and illness	Hospital Access Target (All patients)<br>• % of emergency stays within 4 hours	3
Trauma and illness	Emergency Department wait time by triage category	4
Trauma and illness	Emergency Department stays greater than 24 hours	5
Trauma and illness	Patient off stretcher time	6
Trauma and illness	Lost Minutes	61
Trauma and illness	Emergency Surgery patients treated in hours	62
Trauma and illness	Emergency Surgery patients treated in time	63
Trauma and illness	Transfer of care	69

Table 10 HHS Performance Measures - Safety and Quality Markers

Outcomes Framework Care Domain	Safety and Quality Markers	Indicator Number
Maternity and neonates	Sentinel Events	32
Planned care	Sentinel Events	32
Planned care	Hospital Standardised Mortality Ratio	33
Planned care	Severity Assessment Code (SAC1) analysis completion rates	34
Planned care	Healthcare-associated Staphylococcus Aureus (including MRSA) bacteraemia	35
Planned care	Patient Reported Experience	68

Table 11 HHS Performance Measures – Outcome Indicators

Outcomes Framework Care Domain	Outcome Indicators	Indicator Number
Chronic and complex	Potentially Preventable Hospitalisations (diabetes complications)	38
Chronic and complex	Potentially Preventable Hospitalisations (non-diabetes complications)	39
Chronic and complex	Advance care planning	43
Chronic and complex	Cardiac rehabilitation	44
Maternity and neonates	% of low birthweight babies born to Queensland mothers	41
Mental health, alcohol, and other drugs	Rate of seclusion events	28
Mental health, alcohol, and other drugs	Rate of absent without approval from acute mental health inpatient care	29
Mental health, alcohol, and other drugs	Smoking cessation clinical pathway	42
Other	First Nations peoples' representation in the workforce	47
Planned care	Complaints resolved within 35 calendar days	36
Planned care	Smoking cessation clinical pathway	42
Prevention, early intervention, and primary health care	General oral health care for First Nations peoples	22
Prevention, early intervention, and primary health care	The percentage of oral health activity which is preventive	23
Prevention, early intervention, and primary health care	Access to emergency dental care	24
Prevention, early intervention, and primary health care	Smoking cessation clinical pathway	42
Prevention, early intervention, and primary health care	Adolescent vaccinations administered via the statewide School Immunisation Program	45

Table 12 Statewide Health Equity Key Performance Measures

Outcomes Framework Care Domain	Key Performance Measures	Indicator Number
Chronic and complex	Advance care planning	43
Chronic and complex	Integrated care pathways - Rural and Remote HHSs:<br>• Care pathway in place for patients with identified co-morbidities	60
Maternity and neonates	Proportion of healthy birthweight babies (2,500 grams to 4,499 grams at birth) – Aboriginal and Torres Strait Islander babies	71
Mental health, alcohol, and other drugs	Face to face community follow up within 1-7 days of discharge from an acute mental health inpatient unit	26
Mental health, alcohol, and other drugs<br>Chronic and complex	Suicide count and rate – First Nations People	72
Other	First Nations peoples' representation in the workforce	47
Planned care	Category 1 elective surgery patients treated within the clinically recommended timeframe	7
Planned care	Category 2 and 3 elective surgery patients treated within the clinically recommended timeframe	8
Planned care	Category 1 patients who receive their initial specialist outpatient appointment within the clinically recommended timeframe	17
Planned care	Category 2 and 3 patients who receive their initial specialist outpatient appointment within the clinically recommended timeframe	18
Prevention, early intervention, and primary health care	General oral health care for First Nations peoples	22
Prevention, early intervention, and primary health care	Potentially avoidable deaths – First Nations peoples	70

Schedule 5 Amendments to this service agreement

1. Agreed process to amend this service agreement

1.1 The parties acknowledge that this service agreement is subject to amendment, which will generally occur through:

(a) amendment windows;

(b) extraordinary amendment;

(c) periodic adjustments; and

(d) end of financial year reconciliation.

1.2 **Amendment windows**

(a) There will be set periods of time nominated by the Department during the year (amendment windows) in which a party may propose an amendment and the parties will endeavour to negotiate and finalise proposals to amend this service agreement.

(b) Amendment proposals that are agreed will be documented in a deed of amendment to this service agreement.

(c) Further details on the amendment window process, including the timing of amendment windows, is provided online as detailed in Appendix 1.

1.3 **Extraordinary Amendment**

(a) Outside an amendment window, the Department and the HHS agree to limit any proposal to amend the terms of this service agreement to those where there is an urgent priority need to facilitate a funding allocation (extraordinary amendment). The parties will endeavour to negotiate and finalise any such proposal urgently.

(b) The process for submitting, negotiating, and resolving an extraordinary amendment is available online as detailed at Appendix 1.

(c) Agreed extraordinary amendments will be reflected in a notice issued by the Chief Executive and countersigned as accepted by the HHS. The notice will be replaced when the extraordinary amendment is subsequently formalised in a deed of amendment issued following the next amendment window.

1.4 **Periodic adjustments**

(a) The service agreement value (and corresponding purchased activity) may be adjusted at any time to reflect funding variations that:

(i) occur on a periodic basis or in line with adjustments permitted for specific funding allocations;

(ii) are referenced in the service agreement; and

(iii) are based on a clearly articulated formula, an agreed basis or such other reasonably substantiated basis tied to performance.

(b) Periodic adjustments will be reflected in an adjustment notice issued by the Chief Executive (or delegate) to the HHS, based on relevant data, and subsequently formalised in a deed of amendment issued following the next amendment window.

1.5 **End of financial year reconciliation**

(a) There will be an end of financial year reconciliation process, with the scope defined by the Department and informed by Queensland Government Central Agency requirements.

(b) The Department will provide the HHS with a reconciliation of all service agreement funding and purchased activity for the prior financial year. This will reflect the position following conclusion of the end of financial year adjustments process.

(c) The impact of end of financial year adjustments on subsequent year funding and activity will be incorporated in the service agreement through the deed of amendment executed following the next available amendment window.

(d) This clause will survive expiration of this service agreement.

Appendix 1 Reference Documents

Service Agreement:

- Data supply requirements
- Delivery of Purchased Activity – Requirement for Quality Audits specification sheet
- Dispute resolution process - current
- First Nations First Strategy 2032
- Funding Outcomes Framework
- Hospital and Health Boards Act 2011
- Joint Regional Needs Assessment Framework
- Making Tracks Together: Queensland's Aboriginal and Torres Strait Islander Health Equity Framework
- Making Tracks toward closing the gap in health outcomes for Indigenous Queenslanders by 2033 - Policy and Accountability Framework
- National Agreement on Closing the Gap
- National Health Reform Agreement (NHRA) 2020-25
- Performance Measures Attribute Sheets
- Public Health Practice Manual
- Queensland Government Building Policy Framework and Guideline
- Queensland Health Performance and Accountability Framework
- Service agreement amendment processes
- Specifications supporting the Healthcare Purchasing Model
- Statewide services reference material

Supporting Policy documents

- Aboriginal and Torres Strait Islander Health Workforce Strategic Framework 2016-2026
- Department of Health Strategic Plan 2021-2025

- HEALTHQ32: A vision for Queensland's health system
- My health, Queensland's future: Advancing health 2026
- Queensland Health Equity, Diversity, and Inclusion Statement of Commitment
- System Outlook to 2026 - for a sustainable health service

Contracting and Performance Management Branch
GPO Box 48
Brisbane QLD 4001 Australia

Tel (+61) (07) 3708 5869

www.health.qld.gov.au

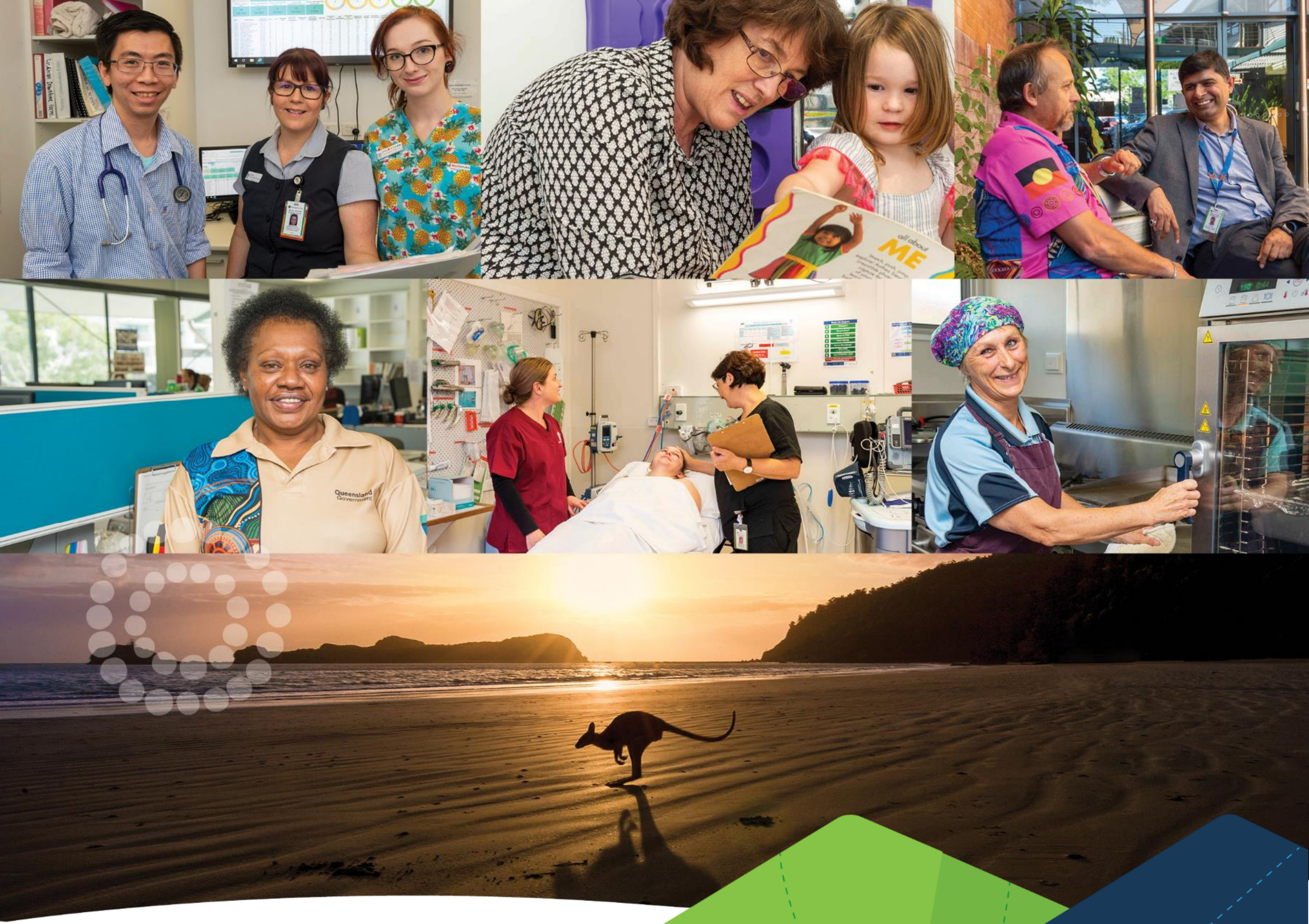

Queensland Health

Service Agreement 2022/23 – 2024/25

Mackay Hospital and Health Service

Queensland Government

Mackay Hospital and Health Service, Service Agreement 2022/23 - 2024/25

Published by the State of Queensland, (Queensland Health), July 2022

This document is licensed under a Creative Commons Attribution 3.0 Australia licence. To view a copy of this licence, visit creativecommons.org/licenses/by/3.0/au

© State of Queensland (Queensland Health) **2022**

You are free to copy, communicate and adapt the work, as long as you attribute the State of Queensland (Queensland Health).

For more information contact:

Contracting and Performance Management Branch, Department of Health, GPO Box 48, Brisbane QLD 4001
Tel (+61) (07) 3708 5869

Email SAM@health.qld.gov.au

An electronic version of this document is available at www.health.qld.gov.au/system-governance/health-system/managing/default.asp

Disclaimer:
The content presented in this publication is distributed by the Queensland Government as an information source only. The State of Queensland makes no statements, representations or warranties about the accuracy, completeness or reliability of any information contained in this publication. The State of Queensland disclaims all responsibility and all liability (including without limitation for liability in negligence) for all expenses, losses, damages and costs you might incur as a result of the information being inaccurate or incomplete in any way, and for any reason reliance was placed on such information.

Acknowledgement

We acknowledge the Aboriginal Traditional Custodian(s) as the Cultural Custodians of the lands, waters and seas across the Mackay, Whitsunday and Isaac regions. We acknowledge and pay our respects to Aboriginal and Torres Strait Islander (First Nation) Peoples, acknowledging Elders past and present and recognise the role of current and emerging leaders in shaping our health systems.

We acknowledge the First Nations people in Queensland and support the cultural knowledge, determination and commitment of Aboriginal and Torres Strait Islander communities in caring for health and wellbeing for millennia. Mackay Hospital and Health Service declares our commitment to reducing inequalities in health outcomes for Aboriginal and Torres Strait Islander peoples as we move to a place of equity, justice and partnership together.

Whilst the Australian South Sea Islander people are not recognised as First Nation peoples, Mackay Hospital and Health Service acknowledges Australian South Sea Islander people, their historical relationship with First Nation peoples and the contributions they have made in the community.

We are proud to recognise and celebrate the cultural diversity of our communities and workforce at the following locations:

Facility	Traditional Owner Group	Region
Bowen Hospital	Juru People (jer-roo)	Whitsunday
Bowen Community Health Service	Juru People	Whitsunday
Clermont Multi-Purpose Health Services	Wangan Jagalingou People (wan-gan jaga-lin-goo)	Isaac
Collinsville Multi-Purpose Health Services	Birriah People (birryah)	Whitsunday
Dysart Hospital	Barada Barna People (ba-rada barn-a)	Isaac
Glendon Community Health Service	Wiri/Widi People (wirry/widdy)	Isaac
Mackay Base Hospital	Yuwi People (you-ee)	Mackay
Mackay Community Health Service	Yuwi People	Mackay
Middlemount Community Health Service	Barada Barna People	Isaac
Moranbah Hospital	Barada Barna People	Isaac
Monash Lodge Aged Care Facility (Clermont)	Wangan Jagalingou People	Isaac
Moranbah Community Health Service	Barada Barna People	Isaac
Proserpine Hospital	Gia People	Whitsunday
Sarina Hospital & Primary Health Care Centre	Yuwi People	Mackay
Whitsunday Community Health Service	Ngaro People (garo)	Whitsunday

Contents

Acknowledgement ... 3

Contents ... 4

Tables ... 5

- **1.** Introduction ... 6
- **2.** Scope ... 6
- **3.** Services ... 6
- **4.** Performance and Accountability Framework ... 7
- **5.** Data supply requirements ... 7
- **6.** Hospital and Health Service accountabilities ... 8
- **7.** Department accountabilities ... 9
- **8.** Achieving health equity with First Nations Queenslanders ... 9
- **9.** General ... 10
- **10.** Counterparts ... 11

Execution ... 12

Schedule 1 HHS profile ... 13

- **1.** HHS profile ... 13

Schedule 2 Funding and purchased activity and services ... 14

- **1.** Introduction ... 14
- **2.** Purchased health services ... 15
- **3.** Teaching training and research ... 17
- **4.** Delivery of purchased activity ... 18
- **5.** Delivery of purchased services ... 19
- **6.** Financial adjustments ... 19
- **7.** Funding sources ... 24
- **8.** Funds disbursement ... 24

Schedule 3 Performance Measures ... 29

- **1.** Performance Measures ... 29

Schedule 4 Amendments to this service agreement ... 31

- **1.** Agreed process to amend this service agreement ... 31

Appendix 1 Reference Documents ... 33

Tables

Table 1 Statewide Services 15
Table 2 Financial adjustments applied on breach of activity thresholds 20
Table 3 Purchasing Incentives 2022/23 21
Table 4 Mackay HHS Total Funding Allocation by Funding Source 2022/23........ 25
Table 5 National Health Reform Funding 27
Table 6 Discretely Funded Programs (Non-ABF)........ 28
Table 7 HHS Performance Measures – Key Performance Indicators 29
Table 8 HHS Performance Measures - Safety and Quality Markers........ 30
Table 9 HHS Performance Measures – Outcome Indicators........ 30

1. Introduction

1.1 In performing this service agreement, the Department and the Hospital and Health Service (HHS) will act consistently with the object of the *Hospital and Health Boards Act 2011* (Qld) (Act).

1.2 The Department and the HHS operate as part of a networked system and agree to work collaboratively with each other, with other HHSs and with the Queensland Ambulance Service in the best interests of the Queensland public sector health system.

1.3 The parties will ensure that planning and delivery of health services is consistent with the strategies and priorities set out by government. The parties recognise the importance of the HHS' Health Equity Strategy (as defined in the Act) and the parties commitment to improving health and wellbeing outcomes and achieving health equity for Aboriginal and Torres Strait Islander peoples.

2. Scope

2.1 This service agreement covers the period from 1 July 2022 to 30 June 2025.

2.2 The parties have identified the services to be provided by the HHS, the funding for the provision of those services, the performance measures applicable to the services and data requirements.

3. Services

3.1 In delivering services, the HHS is required to meet:

(a) the applicable conditions of all national agreements and national partnership agreements between the Queensland Government and the Commonwealth Government and commitments under any related implementation plans; and

(b) the applicable conditions of each agreement or arrangement for funding between the Department and the HHS and commitments under any related implementation plans, which conditions and commitments may be recorded separately to this service agreement but for which funding is (or becomes) provided in Schedule 2.

3.2 The HHS is required to deliver the services outlined in this service agreement for which funding is provided in Schedule 2.

3.3 Where issues arise which prevent the HHS from providing a service or necessitate a reduction in the level or scope of a service provided, prompt notification must be made to the Department and impacted HHSs, with appropriate details. The HHS must minimise any clinical risk or adverse impact to patient experience that may result from service disruption. The Department will respond to the HHS on any requirements it has concerning service delivery and any adjustments triggered by under delivery.

3.4 If the HHS wishes to terminate or reduce service levels for a service for which funding is provided in Schedule 2, this will remain subject to negotiation and agreement by the Department at its discretion.

3.5 For any new services proposed during the term of this service agreement, the parties agree:

(a) the service must meet a demonstrated clinical need and provide value for money;

(b) the commencement of a new service, including the implementation of new models of care, may occur where the service has been commissioned by the Department, a funding stream is in place and any conditions relating to the funding have already been agreed; and

(c) if the HHS wishes to commence providing a new service that has not been commissioned by the Department, this will remain subject to agreement by the Department at its discretion.

3.6 It is acknowledged that there may from time to time need to be service delivery changes between HHSs. Management of inter-HHS relationships should be informed by the following principles:

(a) HHSs should maintain the proportion of out of HHS work undertaken unless as a result of agreed repatriation of patients;

(b) each HHS should manage patients from its own catchment population if it is within its clinical capability to do so as specified by the Clinical Services Capability Framework; and

(c) where it is proposed that a service move from one HHS to another, the Department will consider, as part of its review under clause 3.4 and 3.5, whether the respective Health Service Chief Executives endorse the proposed change in patient flows; and the funding required to follow the patient.

4. Performance and Accountability Framework

4.1 The Queensland Health Performance and Accountability Framework (the Performance and Accountability Framework) sets out the framework within which the Department monitors and manages the performance of public sector health services in Queensland.

4.2 The parties will act consistently with the Performance and Accountability Framework.

5. Data supply requirements

5.1 The HHS will provide the Department with all clinical and non-clinical data that is reasonably required to support the effective management of the public sector health system. This will include, but is not limited to, data that is required to:

(a) fulfil legislative obligations;

(b) deliver accountabilities and obligations to State and Commonwealth Governments; including related to the provision and reconciliation of activity data by the Administrator of the National Health Funding Pool;

(c) monitor and support performance improvement;

(d) manage this service agreement;

(e) support clinical innovation; and

(f) facilitate evaluation and audit.

5.2 The parties agree and acknowledge that:

(a) the Department will keep the HHS informed of the Department's data requirements; and

(b) data will be provided as required, or permitted, by law.

6. Hospital and Health Service accountabilities

6.1 The HHS will perform its obligations under this service agreement.

6.2 As applicable to the HHS and its services, the HHS will comply with:

(a) legislation and subordinate legislation, including the Act;

(b) cabinet decisions;

(c) Ministerial directives;

(d) agreements entered into between the Queensland and Commonwealth governments (or agreements with others in furtherance of such agreements), of which it is informed;

(e) agreements entered into between the Department and other Queensland Government entities, of which it is informed;

(f) all industrial instruments;

(g) all health service directives and health employment directives; and

(h) all policies, guidelines and implementation standards, including human resource policies.

6.3 As part of the commitment to achieving First Nations health equity, the HHS will prioritise the elimination of racial discrimination and institutional racism within its service and ensure that Aboriginal and Torres Strait Islander peoples have access to culturally safe and responsive health services.

6.4 The HHS will ensure that effective health service planning and delivery systems are in place, working in collaboration with the Department.

6.5 The HHS will ensure that health service employees employed by the Chief Executive[1] who perform work for the HHS are managed in accordance with any applicable delegations and directions from the Chief Executive.

6.6 The HHS will ensure that effective asset management systems are in place, working in collaboration with the Department.

6.7 The HHS will maintain accreditation to the standards required by the Department.

6.8 The HHS will appropriately perform and fulfil its functions under the Act.

6.9 The HHS will provide to the Chief Executive reports of a type, and at the intervals, agreed between the parties, or as reasonably specified by the Chief Executive.

7. Department accountabilities

7.1 The Department will perform its obligations under this service agreement including, in return for the HHS performing its obligations and delivering the services, providing funding to the HHS as stipulated in this service agreement (as amended).

7.2 The Department will:

(a) comply with applicable legislation and subordinate legislation, including the Act, as relates to this service agreement; and

(b) perform the system manager role (as defined in the Act) through the Chief Executive;

7.3 The Chief Executive will appoint health service employees to perform work for the HHS for the purpose of enabling the HHS to perform its functions under the Act.

7.4 The Chief Executive will consult, cooperate, and coordinate with the HHS to ensure legal compliance with the *Work Health and Safety Act 2011* and other legislation as it applies to the scope, nature and location of operations associated with this service agreement.

7.5 The Chief Executive will appropriately perform and fulfil their functions under the Act.

8. Achieving health equity with First Nations Queenslanders

8.1 Through legislative amendments to the Act and the Hospital and Health Boards Regulation 2012, and the release of *Making Tracks Together: Queensland's Aboriginal and Torres Strait Islander Health Equity Framework* (2021), Queensland Health has strengthened its commitment to improving health and wellbeing outcomes and achieving health equity for First Nations peoples.

8.2 The HHS will develop a Health Equity Strategy to demonstrate the HHS's activities and key performance measures to achieve health equity with First Nations peoples that is compliant with legislative requirements. The Health Equity Strategy will act as the principal

[1] In this service agreement the term Chief Executive takes the meaning applied in the Act, which is the Chief Executive of Queensland Health, but which is generally referred to as the Director-General.

accountability mechanism between community and the HHS in achieving health equity for First Nations Queenslanders and forms a key part of the Queensland Government's commitment to the *National Agreement on Closing the Gap* (2020).

8.3 The HHS will review the Health Equity Strategy at least once every three years and will publish the Health Equity Strategy in a way that allows it to be accessed by members of the public.

8.4 The HHS will ensure that commitment and leadership is demonstrated through implementing the actions and achieving the key performance measures outlined in the Health Equity Strategy.

8.5 The HHS will report publicly on progress against the Health Equity Strategy.

8.6 The HHS will support the implementation of other supplementary policies and strategies to drive health equity across the public health system, including relevant election commitments.

8.7 The HHS will participate as a partner in the design, development and implementation of the new *Queensland First Nations Health Workforce Strategy for Action.*

9. General

9.1 Sub-contracting

(a) The HHS must have appropriate systems in place to ensure that any subcontractor is accredited (as applicable), qualified, and otherwise fit to perform any services for which it is contracted.

(b) The HHS must ensure that any sub-contractor who has access to confidential information (as defined in the Act) or personal information (as defined in the *Information Privacy Act 2009* (Qld)) complies with obligations no less onerous than those imposed on the HHS.

9.2 Insurance

The HHS must:

(a) hold and maintain the types and levels of insurances that the HHS considers appropriate according to its functions and obligations; and

(b) comply with reasonable requests or directions from the Department in this regard.

9.3 Amendment

The process for amending this service agreement is provided for under the Act and further outlined in Schedule 4.

10. Counterparts

10.1 This service agreement may be executed in two or more identical copy counterparts, each of which together will be deemed an original, but all of which together will constitute one and the same instrument.

10.2 In the event that any signature executing this service agreement or any part of this service agreement is delivered by facsimile transmission or by scanned e-mail delivery of a '.pdf' format data file or equivalent of the entire agreement, the signature will create a valid and binding obligation of the party executing (or on whose behalf the signature is executed) with the same force and effect as if the signature page were an original. For the avoidance of doubt, this service agreement may be in the form of an electronic document and may be electronically signed.

10.3 For execution under this clause 10 to be valid the entire service agreement upon execution by each individual party must be delivered to the remaining parties.

Execution

Executed as an agreement in Queensland

Signed by the Chief Executive, Queensland Health:)
)

....

Signature of Chief Executive

SHAUN DRUMMOND

....

Name of Chief Executive (print)

....

(date)

Signed for and on behalf of the Mackay Hospital and Health Service:)
)

....

Signature of Hospital and Health Board Chair

Darryl Camilleri

....

Name of Hospital and Health Board Chair (print)

17 June 2022

....

(date)

Execution

Executed as an agreement in Queensland

Signed by the Chief Executive, Queensland Health:)

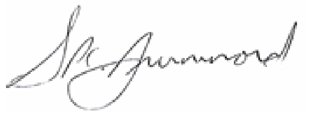

....

Signature of Chief Executive

SHAUN DRUMMOND

....

Name of Chief Executive (print)

29 June 2022

....

(date)

Signed for and on behalf of the Mackay Hospital and Health Service:)

....

Signature of Hospital and Health Board Chair

....

Name of Hospital and Health Board Chair (print)

....

(date)

Schedule 1 HHS profile

1. HHS profile

This Schedule does not apply to this HHS.

Schedule 2 Funding and purchased activity and services

This Schedule 2 sets out:

(a) the services which are to be provided by the HHS;

(b) the activity purchased by the Department from the HHS;

(c) the funding provided for delivery of the purchased activity;

(d) the criteria and processes for financial adjustments associated with the delivery of purchased activity and specific funding allocations;

(e) the sources of funding that this service agreement is based on and the manner in which these funds will be provided to the HHS.

1. Introduction

1.1 The HHS will deliver the services for which funding is provided in this Schedule 2. In providing these services, the HHS will ensure that:

(a) all statewide and national policy frameworks, guidelines, protocols and implementation standards applicable to the service provided are followed;

(b) participation in national programs is facilitated and supported, including where these programs are provided by the Commonwealth Government;

(c) service delivery partnerships, including with other HHSs, primary care organisations and non-government organisations, are maintained and operate effectively;

(d) collaboration and engagement with other service providers and stakeholders is initiated and maintained to ensure that an integrated system of treatment, care and support is in place and to facilitate the delivery of comprehensive and effective services. This may include but is not limited to:

(i) other HHSs;

(ii) non-government organisations;

(iii) Aboriginal and Torres Strait Islander community-controlled health organisations;

(iv) Queensland Ambulance Service;

(v) services provided through the Department of Health (for example, Pathology Queensland);

(vi) primary care providers;

(vii) other government departments and agencies; and

(viii) private providers;

(e) models of care and service delivery arrangements are consistent with evidence-based practice and offer value for money;

(f) services are provided on an equitable basis to the community, and processes are in place to ensure that services reach identified target populations, high risk groups and hard to reach communities;

(g) referral networks and pathways continue to operate effectively; and

(h) innovation and continuous improvement is supported.

2. Purchased health services

2.1 Table 4, Table 5 and Table 6 outline the activity and service streams which the Department agrees to purchase from the HHS pursuant to this service agreement.

2.2 More generally, this will include the following:

2.3 **Statewide Services**

(a) The designation of a service as a statewide service (either a clinical statewide service or a clinical support statewide service) will be determined by the Department, consistent with the stipulated governance arrangements for such services.

(b) The HHS will:

(i) collaborate with the Department and other HHSs in the implementation of, and adherence to, the governance and oversight arrangements for statewide services;

(ii) participate in, and contribute to, the staged review of the purchasing model for identified statewide services; and

(iii) ensure that referral pathways in and out of each statewide service are followed.

2.4 **Clinical Statewide Services and Clinical Support Statewide Services provided**

This clause does not apply to this HHS.

Table 1 Statewide Services

This table does not apply to this HHS.

2.5 **Regional services**

This clause does not apply to this HHS.

2.6 **Prevention services and population health services**

(a) The HHS will provide a range of services with a focus on the prevention of ill-health and disease, including:

(i) Specialist Public Health Units;

(ii) preventive health services;

(iii) immunisation services;

(iv) sexually transmissible infections including HIV and viral hepatitis;

(v) tuberculosis services; and

(vi) population health screening including, but not limited to, cancer screening services and newborn blood spot screening.

(b) Services will be provided in line with public health related legislation and the service delivery and reporting requirements outlined in the *Public Health Practice Manual*, as these relate to the services provided.

2.7 Aboriginal and Torres Strait Islander health services

The HHS will provide Aboriginal and Torres Strait Islander specific health services and initiatives consistent with the principles and objectives of the Queensland Government's *Making Tracks towards closing the gap in health outcomes for Indigenous Queenslanders by 2022 – Policy and Accountability Framework*. These service and initiatives will be delivered in line with guidance from the Aboriginal and Torres Strait Islander Health Division.

2.8 Mental health alcohol and other drugs services

The HHS will provide treatment, care and support for individuals who are, or may be, experiencing substance use disorders and/or other mental health disorders, mental health crisis and suicidal distress, and their families and carers. Services will be delivered in line with guidance from the Mental Health Alcohol and Other Drugs Branch:

2.9 Oral health services

The HHS will provide oral health services to people who meet the eligibility criteria for accessing public dental services in Queensland. Services will be delivered in line with guidance from the Office of the Chief Dental Officer.

2.10 Prisoner health services

This clause does not apply to this HHS.

2.11 Youth detention services

This clause does not apply to this HHS.

2.12 Refugee health

This clause does not apply to this HHS.

2.13 State-funded outreach services

(a) Where state-funded outreach services are provided;

(i) funding for the service will remain part of the providing HHS's funding allocation; and

(ii) the activity must be recorded at the HHS where the outreach service is being provided

(b) Any changes to the provision of outreach services will follow the requirements set out in clause 3 of this service agreement.

3. Teaching training and research

The HHS will provide the teaching, training and research programs for which funding is provided within Schedule 2 and as described below.

3.1 Clinical education and training

(a) The HHS will provide education and training placements for the following professional groups consistent with and proportionate to the capacity of the HHS and will support and align with stipulated placement terms governing clinical placements in Queensland Health facilities;

(i) medical students;

(ii) nursing and midwifery students;

(iii) pre-entry clinical allied health students;

(iv) interns;

(v) rural generalist trainees;

(vi) vocational medical trainees;

(vii) first year nurses and midwives;

(viii) re-entry to professional register nursing and midwifery candidates;

(ix) dental students;

(x) allied health rural generalist training positions;

(xi) Aboriginal and Torres Strait Islander Health Workers and Aboriginal and Torres Strait Islander Health Practitioners.

(b) The HHS will comply with the state-wide vocational medical training pathways.

(c) The HHS will support profession specific and inter-profession statewide allied health clinical education programs.

(d) The HHS will continue to implement and retain the following positions provided through clauses to the *Health Practitioners and Dental Officers (Queensland Health) Certified Agreement (No 3)*:

(i) health practitioner research positions provided through the Research Package for Health Practitioners; and

(ii) clinical educator positions provided through the Clinical Education Management Initiative for Health Practitioners.

(e) The HHS will maintain or increase its contribution of staff to the Queensland Country Relieving doctors program and the receiving HHS will be responsible for wages, clinical governance and appropriate supervision of the junior medical relievers.

3.2 Statewide training, education and research

This clause does not apply to this HHS.

3.3 **Health and medical research**

The HHS will:

(a) develop and implement a strategy to drive increased research activity and its translation into clinical practice;

(b) support increased and equitable access to clinical trials for patients;

(c) support clinicians to undertake research linked to their practice; and

(d) ensure high quality and timely research governance approval processes.

4. Delivery of purchased activity

4.1 The Department and the HHS will monitor actual activity against purchased levels and will take action as necessary to ensure delivery of purchased levels is achieved. The HHS has a responsibility to only recognise revenue that is linked with actual activity delivered against purchased volumes.

4.2 The HHS will actively monitor variances from purchased activity levels and notify the Department as soon as the HHS becomes aware of significant variances.

4.3 The HHS will also notify the Department of deliberate changes to the consistent recording of activity within year that would result in additional activity being recorded for existing health services.

4.4 If the HHS wishes to convert activity between purchased activity types, programs and levels the HHS must negotiate this with the Department based on a sound needs based rationale.

4.5 The Department will reconcile in-scope activity, as defined in the Activity Reconciliation specification sheet (available online, as detailed in Appendix 1), delivered by the HHS against the purchased in-scope activity targets outlined in Table 4.

4.6 Activity reconciliation will be undertaken in February (for the July to December period) and August (for the January to June period) each year and will be derived through application of the methodology which is documented in the Activity Reconciliation specification sheet.

4.7 Should the HHS be unable to deliver the activity that has been funded a financial adjustment will be applied.

4.8 Under delivery of in-scope activity, as defined in the Activity Reconciliation specification sheet, will be withdrawn from the HHS at 100% of the Queensland Efficient Price (QEP).

4.9 Funding and corresponding activity that is withdrawn from the HHS may be reallocated to an alternate provider that can undertake the activity.

4.10 Any resulting changes to funding and purchased activity levels will be actioned through the processes outlined in Schedules 2 and 4 of this service agreement.

4.11 The HHS will maintain its efforts to accurately record the delivered activity and submit this data to the Department in accordance with the agreed requirements.

5. Delivery of purchased services

5.1 As part of the service agreement, the Department purchases a range of services (or deliverables) from the HHS for the delivery of certain programs and projects.

5.2 These program or project services may be the subject of separately agreed conditions tied to that funding and the focus of detailed monitoring by the Department.

5.3 Conditions may include, but are not limited to:

(a) establishment and/or commencement of services;

(b) delivery of activity;

(c) workforce obligations;

(d) establishment of oversight committees;

(e) opening or upgrades to facilities;

(f) program evaluation;

(g) program management;

(h) reporting or notification obligations; and

(i) attainment of performance standards.

5.4 The HHS will ensure that the conditions are achieved within the stipulated time period.

5.5 The HHS will notify the Department if the HHS forecasts an inability to achieve program or project objectives or the conditions.

5.6 The Department may withdraw allocated funding pro rata to the level of under delivery if the services for a specified program or project are not being fully delivered according to the objectives or conditions.

5.7 Any resulting changes to funding and purchased activity levels will be actioned through the processes outlined in Schedules 2 and 4 of this service agreement.

6. Financial adjustments

6.1 **Activity targets**

(a) The Department will initiate a joint process with the HHS to determine whether a financial adjustment should be applied in relation to any purchased activity which has breached the thresholds identified bi-annually. This process will take into account any relevant matters that have been identified in a review/analysis of the breach as well as the outcomes of the activity plan implemented to address the activity breach.

(b) Activity will be monitored at the purchasing hierarchy level. Providing the HHS meets all relevant KPIs and specific funding allocations, the HHS has the ability to negotiate the transfer of activity across the purchasing hierarchy with the Department.

(c) Table 2 demonstrates the financial adjustment that will be applied when activity thresholds have been breached.

(d) The HHS may not utilise the provisions within AASB15 *Revenue from Contracts with Customers* to override the application of any financial adjustment made by the Department in line with Table 2.

Table 2 Financial adjustments applied on breach of activity thresholds

Example of Breach	Description	Financial Adjustment
Over performance	Activity exceeds that specified in the service agreement value for in-scope activity as shown in Table 4.	Purchasing contracts are capped and an HHS will not be paid for additional activity with the exception of activity that is in scope for the identified purchasing incentives as set out in Table 3.
Under performance	Activity is below that specified for in-scope activity as shown in Table 4.	Purchased activity and the related funding will be withdrawn at 100% of the Queensland Efficient Price and reallocated to an alternate provider that can undertake the activity.<br>Refer to Table 4 for the HHS QWAU target.
Failure to deliver on service commitments linked to specific funding allocations	Specific funding allocations<br>National Partnership Agreements.	It is at the discretion of the Department to withdraw allocated funding pro rata to the level of under delivery.
For all other types of activity variance, any financial adjustment will be made at the discretion of the Department.		

6.2 National Partnership on COVID-19 Response

(a) The Department will provide additional funding to the HHS under the *National Partnership on COVID-19 Response* where actual additional cost is demonstrated.

(b) Funding will be provided where the HHS has:

(i) undertaken activity that is in-scope for the State Public Health Payment, as defined under the *National Partnership on COVID-19 Response*, during the reporting period*;* and/or

(ii) undertaken activity that is in-scope for the Hospital Services Payment, as defined under the *National Partnership on COVID-19 Response*, during the reporting period; and

(iii) met the reporting requirements in relation to COVID-19 expenditure, as prescribed under the *National Partnership on COVID-19 Response* and by the Department.

(c) Additional costs that are reimbursed through the State Public Health Payment and the Hospital Services Payment will be excluded from the calculation of activity eligible for funding under the terms of the *National Health Reform Agreement*.

(d) There is no guarantee that the HHS will have access to additional funding under the State Public Health Payment or the Hospital Services Payment.

(e) All funding that is provided through the State Public Health Payment and the Hospital Services Payment will be subject to a reconciliation to be undertaken by the Administrator of the National Health Funding Pool. The HHS will be required to provide evidence to support claims for additional costs. If the HHS is unable to provide the required evidence with their expenditure claim, funding received may be recalled subject to reconciliation.

(f) Funding adjustments will be actioned through the processes outlined in Schedules 2 and 4 of this service agreement.

6.3 Purchasing incentives

(a) The purchasing approach includes a range of funding adjustments which aim to incentivise high quality and high priority activity, support innovation and evidence-based practice, deliver additional capacity through clinically and cost effective models of care and dis-incentivise care which provides insufficient or no benefit for patients. This includes incentive payments for HHSs who achieve targets in specific priority areas. The purchasing incentives are detailed in Table 3. The Department must reconcile the applicable purchasing incentives in Table 3 in line with the timeframes specified in the relevant purchasing specification sheet. The Department must provide a copy of the reconciliation statement to the HHS.

(b) Funding adjustments must be based on the requirements contained in the relevant purchasing specification sheet for that purchasing incentive.

(c) Any resulting changes to funding and purchased activity levels will be actioned through the processes outlined in Schedules 2 and 4 of this service agreement.

Table 3 Purchasing Incentives 2022/23

Incentive	
**Quality Improvement Payment (QIP)**	
Antenatal care for First Nations Women	Payments for achieving two Closing the Gap targets for First Nations women:<br>• to attend five or more antenatal visits with their first antenatal first taking place in the first trimester; and<br>• to stop smoking by 20 weeks gestation.
**Purchasing incentives**	
Virtual care incentive	Incentive funding to increase the number of specialist outpatient services which are provided in virtual settings.
Own source revenue growth	Incentivise the recognition of own source revenue through matching growth in own source revenue with public activity growth funding.
**ABF model localisations**	
Sentinel events	Payment withdrawn for sentinel events as per the national ABF model.
Advance Care Planning (ACP)	QWAUs for HHSs who offer ACP discussions to admitted patients, non-admitted outpatients, community health patients and Emergency Department patients.
Emergency Department Did Not Wait (DNW)	Zero QWAUs for DNWs.
Fractured neck of femur	QWAUs reduced by 20% for non-timely surgical treatment of fractured neck of femur.
Hospital in The Home (HITH)	QWAUs increased for Hospital in the Home (HITH) admissions of Residential Aged Care Facility residents.
Out-of-scope services	Nil QWAUs for out-of-scope procedures.
Pre-operative bed days	QWAUs for long stay days above the upper trim point less ICU days reduced equivalent to pre-operative days, up to a maximum of 3 days, for elective episodes.
Smoking cessation (community mental health)	QWAUs for smoking cessation activity for community mental health patients.

Incentive	
Smoking cessation (inpatients)	QWAUs for smoking cessation activity for publicly funded inpatients.
Stroke care	10% QWAU loading for acute stroke patients admitted to Statewide Stroke Clinical Network-endorsed stroke unit care.
Telehealth (admitted patients)	QWAUs for provider-end of in-scope admitted patient telehealth activity.
**Commissioning mechanisms**	
High-cost home support	Funding for approved individuals requiring 24-hour home ventilation.
Patient flow initiative	Provision of non-recurrent WAU-backed funding to participating HHS who successfully implement agreed recommendations.
Rapid access clinics	Recurrent WAU-backed funding to support the implementation of rapid access clinics to reduce pressure on emergency departments.
Expansion of sub-acute and long stay care	Additional funding to increase the availability of and access to care for sub-acute and long stay patients, thereby improving access to care in a range of settings and releasing capacity within acute facilities.
Connected Community Pathways	Funding to incentivise evidence-based and innovative models of care which promote the delivery of care outside acute facilities and support shared-care partnership arrangements.

6.4 Surgery Connect reimbursements

(a) The HHS will reimburse the Department for the actual costs of activity for nominated patient referrals to the Surgery Connect program where:

(i) The HHS has nominated the patient referral as HHS funded on entry of the referral in the Surgery Connect Activity Navigator (SCAN); and

(ii) The HHS Chief Finance Officer has recorded approval of the nomination in SCAN;

or

(iii) The HHS has obtained the Department’s agreement to retrospectively convert a defined patient cohort to HHS funded status in SCAN.

(b) The HHS may only request retrospective conversion of activity to HHS funded within the financial year in which the activity has taken place.

(c) Any resulting changes to funding and purchased activity levels will be actioned through the processes outlined in Schedules 2 and 4 of this service agreement.

6.5 Financial adjustments – other

(a) Notwithstanding the provisions regarding the recognition of revenue as stipulated in AASB1058 *Income of Not-for-Profit Entities* and/or AASB15 *Revenue from Contracts with Customers*, the Department may seek to recover funding from an HHS that was provided through this service agreement which has:

(i) not been utilised in accordance with its intended purpose; and/or

(ii) not been utilised within the prescribed time period to deliver the agreed outcomes/services.

(b) If the Chief Executive (or delegate) determines that previously allocated funding is to be recovered, any resulting changes to funding and purchased activity levels will be actioned through the processes outlined in Schedules 2 and 4 of this service agreement.

6.6 Public and private activity/own source revenue

(a) Own source revenue comprises grants and contributions, user charges and other revenues.

(b) Where an HHS is above its own source revenue target, it will be able to retain the additional own source revenue with no compensating adjustments to funding from other sources where the additional revenue is not attributable to a private patient consistent with clauses A9 to A13 and A44 of the *National Health Reform Agreement.*

(c) Where an HHS is below its own source revenue target in respect of private patients, it will experience a reduction in revenue with no compensating adjustments to funding from other sources.

(d) The HHS will make all efforts to identify and maximise revenue from appropriate third parties to ensure optimisation of available State and Commonwealth funding across the Queensland public sector health system.

(e) The own source revenue identified in Table 4 is an estimate generated by the HHS which allows all third-party funding sources associated with service delivery to be identified. The HHS will ensure that this estimate is substantiated and accurate to ensure no significant variances to actual revenue generated.

(f) The HHS will routinely revise and update the estimate to ensure alignment between the service agreement and Queensland Treasury's reporting system (TRIDATA).

(g) Budget adjustments for changes in own source revenue from private patients will be actioned through the processes set out in Schedule 4 of this service agreement.

7. Funding sources

7.1 The four main funding sources contributing to the HHS service agreement value are:

(a) Commonwealth funding;

(b) State funding;

(c) grants and contributions; and

(d) own source revenue.

7.2 Table 4 provides a summary of the funding sources for the HHS and the total value of the service agreement.

8. Funds disbursement

8.1 The Department agrees to pay the HHS the amount described in Table 4 of Schedule 2, subject to:

(a) parliamentary appropriation and adequate funds being allocated to the Department; and

(b) the terms of this service agreement.

8.2 All payments under this service agreement will be made in accordance with the requirements of the *National Health Reform Agreement* and the Act.

8.3 The Chief Executive will direct the disbursement of both State and Commonwealth funding from the State Pool Account, the State Managed Fund and the Department of Health Expenditure account to the HHS.

8.4 However, the State (represented by the Chief Executive) will not:

(a) redirect Commonwealth payments between HHSs;

(b) redirect Commonwealth payments between funding streams (e.g., from ABF to block funding); and/or

(c) adjust the payment calculations underpinning the Commonwealth's funding.

8.5 The Department will pay state-funded activity-based funding, block funding and system manager funding to the HHS on a fortnightly basis in line with receipt of the State Appropriation Payment. The payment made will be equal to 1/26th of the state-funded component of the service agreement value described in Table 4.

8.6 The Department will pay Commonwealth-funded activity-based funding and block funding on a monthly basis in line with receipt of the Commonwealth payment. The payment made will be equal to 1/12th of the Commonwealth funded component of the service agreement value described in Table 4.

8.7 Where the parties have agreed to amend the service agreement value or the Commonwealth contribution amount changes, the fortnightly and/or monthly payments will be adjusted to reflect the amended service agreement value.

8.8 Service agreement payments may be made outside of these timeframes with the approval of the Department's Chief Finance Officer.

Table 4 Mackay HHS Total Funding Allocation by Funding Source 2022/23

Funding Source	NWAU (N2122)	QWAU (Q24)	Value ($)
**NHRA Funding**			
**ABF Pool**			
ABF Funding (in scope NHRA)[2]			
Commonwealth[2]	65,860		$155,325,761
State		65,647	$174,539,216
State Specified Grants			$8,645,301
State-wide Services			$0
**State Managed Fund**			
Block Funding			
Small Rural Hospitals		7,635	$53,001,982
Teaching, Training & Research			$14,819,020
Non-Admitted Child & Youth Mental Health			$3,628,218
Non-Admitted Home Ventilation			$0
Non-Admitted Mental Health			$16,307,850
Other Non-Admitted Service			$0
Highly Specialised Therapies			$0
**Total NHRA Funding**	**65,860**	**73,282**	**$426,267,349**
**Out of Scope NHRA**			
**Queensland ABF Model**			
DVA		499	$2,697,185
NIISQ/MAIC		209	$583,656
Oral Health		1,326	$7,188,444
BreastScreen		332	$2,129,770
**Total Queensland ABF Funding**	**-**	**2,365**	**$12,599,055**
**Discretely Funded Programs[3]**			
Department of Health			$37,367,800
Locally receipted funds			$5,380,817
**Total Discretely Funded Programs**	**-**	**-**	**$42,748,617**
**Own Source Revenue**			
Private Patient Admitted Revenue[4]	*1,911*	2,112	$10,697,109
Non-Admitted Services		2,173	$3,645,515
Pharmaceuticals Benefits Scheme		1,927	$13,395,149
Other Activities[5]		566	$11,483,298
**Total Own Source Revenue**	**-**	**6,778**	**$39,221,071**

[2] The split between Commonwealth and State NHRA funding will change during the year as the purchased activity targets are updated and the National Health Funding Board updates their payment advice accordingly.

[3] Includes all other (non-ABF) State and Commonwealth funded health services including, but not limited to, Prisoner Health, Public Health and Prevention Services.

[4] The estimated value of the revenue earned from private patients, based on OSR estimates provided by the HHS.

[5] Incorporates all OSR which is not identified elsewhere in Table 4.

Funding Source	NWAU (N2122)	QWAU (Q24)	Value ($)
Locally Receipted Funds (exc. Discretely Funded Programs)[6]			$9,732,875
Depreciation			$31,599,000
**NPA COVID-19 Response**			
Hospital Services Payment			$0
State Public Health Payment			$0
COVID-19 Vaccine Payment			$0
**Total NPA COVID-19 Response Funding**	**-**	**-**	**$0**
**GRAND TOTAL**	**65,860**	**82,425**	**$562,167,967**

Pool Accounts			
ABF Pool (National Health Funding Pool)[7]			$351,109,334
State Managed Fund[8]			$87,757,070
System Manager			$37,367,800

[6] Includes items such as training programs and donations. Does not include locally receipted funds associated with discretely funded programs, e.g.Transition Care.
[7] Articulates the financial payment made to support in-scope ABF services under the NHRA including DVA, NIISQ, MAIC and Breastscreen Services. Applies to all HHSs except Central West HHS and Torres and Cape HHS.
[8] Articulates the payment made for block funded services under the NHRA, DVA, NIISQ and MAIC services.

Table 5 National Health Reform Funding

NHRA Funding Type	NWAU (N2122)	Commonwealth ($)	State ($)	Other State funding[9] DVA/MAIC/Oral Health/BreastScreen ($)	Total ($)
**National Efficient Price (NEP)**					**$5,597**
**ABF Allocation (NWAU)**					
Emergency Department	11,194	$26,399,113	$31,133,977	$1,948,594	$59,481,684
Acute Admitted	40,380	$95,233,305	$112,314,061	$0	$207,547,366
Admitted Mental Health	3,387	$7,987,553	$9,420,176	$0	$17,407,729
Sub-Acute	3,247	$7,656,798	$9,030,098	$0	$16,686,897
Non-Admitted	7,653	$18,048,992	$21,286,204	$10,650,462	$49,985,658
**Total ABF Pool Allocation**	**65,860**	**$155,325,761**	**$183,184,517**	**$12,599,055**	**$351,109,334**
**Block Allocation**					
Teaching Training and Research	-	$3,055,518	$11,763,503	-	$14,819,020
Small and Rural Hospitals[10]	-	$19,123,284	$33,878,698	-	$53,001,982
Non-Admitted Mental Health	-	$6,210,049	$10,097,801	-	$16,307,850
Non-Admitted Child & Youth Mental Health	-	$370,123	$3,258,094	-	$3,628,218
Non-Admitted Home Ventilation	-	$0	$0	-	$0
Other Non-Admitted Services	-	$0	$0	-	$0
Other Public Hospital Programs	-	$0	$0	-	$0
Highly Specialised Therapies	-	$0	$0	-	$0
**Total Block Allocation**	**-**	**$28,758,974**	**$58,998,096**	**-**	**$87,757,070**
**Grand Total Funding Allocation**					**$438,866,404**

[9] State funding transacted through the Pool Account; not covered under the NHRA

[10] Incorporating small regional and rural public hospitals, five specialist mental health facilities (Baillie Henderson Hospital, Jacaranda Place – Queensland Adolescent Extended Treatment Centre, The Park – Centre for Mental Health, Kirwan Rehabilitation Unit and Charters Towers Rehabilitation Unit) and the Ellen Barron Family Centre.

Table 6 Discretely Funded Programs (Non-ABF)

Discretely Funded Programs	$
Aged Care Assessment Program	$636,347
Alcohol, Tobacco and Other Drugs	$2,993,517
Community Health Programs	$18,163,900
Disability Residential Aged Care Services	$0
Home and Community Care Program (HACC)	$3,997,117
Interstate Patients (QLD residents)	$276,878
Multi-purpose Health Services	$3,646,137
Other State Funding	-$1,389,560
Patient transport	$9,711,992
Prevention Services and Public Health	$2,060,171
Prisoner Health Services	$0
Research	$78,418
Transition Care	$1,383,700
Residential Aged Care Services	$1,190,000
**TOTAL**	**$42,748,617**

Schedule 3 Performance Measures

1. Performance Measures

1.1 The performance of the HHS will be measured according to the assessment criteria and processes described in the Performance and Accountability Framework.

1.2 The detailed specification for each of the performance measures listed in this service agreement are provided through performance measure attribute sheets.

1.3 The performance measures identified in this service agreement are applicable to the HHS unless otherwise specified within the attribute sheet.

1.4 HHSs are also required to report against the agreed key performance measures in their Health Equity Strategy.

Table 7 HHS Performance Measures – Key Performance Indicators

Key Performance Indicators
Hospital Acquired Complications
Hospital Access Target (admitted patients)<br>• % of emergency stays within 4 hours
Emergency Department stays greater than 24 hours
Emergency Department wait time by triage category
Face to face community follow up within 1-7 days of discharge from an acute mental health inpatient unit
Patient off stretcher time
Lost minutes per ambulance (in development)
Patient flow target: time between the decision to admit and patient leaving the Emergency Department (in development)
Category 1 elective surgery patients treated within the clinically recommended timeframe
Elective surgery patients waiting longer than the clinically recommended timeframe
Emergency Surgery (placeholder - measure to be determined)
Category 1 patients who receive their initial specialist outpatient appointment within the clinically recommended timeframe
Patients waiting longer than clinically recommended for their initial specialist outpatient appointment
Category 4 gastrointestinal endoscopy patients treated within the clinically recommended timeframe
Gastrointestinal endoscopy patients waiting longer than the clinically recommended timeframe
Access to oral health services (adults)
Access to oral health services (children)
Potentially Preventable Hospitalisations – First Nations peoples:<br>• Diabetes complications<br>• Selected conditions
Reduction in the proportion of Aboriginal and Torres Strait Islander failure to attend appointments
Telehealth utilisation rates:<br>• Number of non-admitted telehealth service events
Forecast operating position:<br>• Full year<br>• Year to date

Average sustainable Queensland Health FTE
Capital expenditure performance
Proportion of mental health and alcohol and other drug service episodes with a documented care plan
Proportion of overnight inpatients discharged by 10am

Table 8 HHS Performance Measures - Safety and Quality Markers

Safety and Quality Markers
Sentinel Events
Hospital Standardised Mortality Ratio
Healthcare-associated Staphylococcus Aureus (including MRSA) bacteraemia
Severity Assessment Code (SAC) analysis completion rates
Patient Reported Experience

Table 9 HHS Performance Measures – Outcome Indicators

Outcome Indicators
Rate of seclusion events
Rate of absent without approval from acute mental health inpatient care
Reperfusion therapy for acute ischaemic stroke
Access to emergency dental care
First Nations peoples representation in the workforce
General oral health care for First Nations peoples
% of low birthweight babies born to Queensland mothers
Complaints resolved within 35 calendar days
Advance care planning
Smoking cessation clinical pathway
Potentially Preventable Hospitalisations (diabetes complications)
Potentially Preventable Hospitalisations (non-diabetes complications)
The percentage of oral health activity which is preventive
Cardiac rehabilitation
Adolescent vaccinations administered via the statewide School Immunisation Program

Schedule 4 Amendments to this service agreement

1. Agreed process to amend this service agreement

1.1 The parties acknowledge that this service agreement is subject to amendment, which will generally occur through:

(a) amendment windows;

(b) extraordinary amendment;

(c) periodic adjustments; and

(d) end of financial year reconciliation.

1.2 **Amendment windows**

(a) There will be set periods of time nominated by the Department during the year (amendment windows) in which a party may propose an amendment and the parties will endeavour to negotiate and finalise proposals to amend this service agreement.

(b) Amendment proposals that are agreed will be documented in a deed of amendment to this service agreement.

(c) Further details on the amendment window process, including the timing of amendment windows, is provided online, as detailed in Appendix 1.

1.3 **Extraordinary Amendment**

(a) Outside an amendment window, the Department and the HHS agree to limit any proposal to amend the terms of this service agreement to those where there is an urgent priority need to facilitate a funding allocation (extraordinary amendment). The parties will endeavour to negotiate and finalise any such proposal urgently.

(b) The process for submitting, negotiating and resolving an extraordinary amendment is available online, as detailed at Appendix 1.

(c) Agreed extraordinary amendments will be reflected in a notice issued by the Chief Executive countersigned as accepted by the HHS, which notice will be replaced when the extraordinary amendment is subsequently formalised in a deed of amendment issued following the next amendment window.

1.4 **Periodic adjustments**

(a) The service agreement value (and corresponding purchased activity) may be adjusted at any time to reflect funding variations that:

(i) occur on a periodic basis or in line with adjustments permitted for specific funding allocations;

(ii) are referenced in the service agreement; and

(iii) are based on a clearly articulated formula, an agreed basis or such other reasonably substantiated basis tied to performance.

(periodic adjustment).

(b) Periodic adjustments will be reflected in an adjustment notice issued by the Chief Executive (or delegate) to the HHS, based on relevant data, and subsequently formalised in a deed of amendment issued following the next amendment window.

1.5 **End of financial year reconciliation**

(a) There will be an end of financial year reconciliation process, with the scope defined by the Department and informed by Queensland Government Central Agency requirements.

(b) The Department will provide the HHS with a reconciliation of all service agreement funding and purchased activity for the prior financial year. This will reflect the position following conclusion of the end of financial year adjustments process.

(c) The impact of end of financial year adjustments on subsequent year funding and activity will be incorporated in the service agreement through the deed of amendment executed following the next available amendment window.

(d) This clause will survive expiration of this service agreement.

Appendix 1 Reference Documents

Hospital and Health Boards Act 2011

National Health Reform Agreement (NHRA) 2020-25

System Outlook to 2026 - for a sustainable health service

Queensland Health Performance and Accountability Framework

My health, Queensland's future: Advancing health 2026

Department of Health Strategic Plan 2021-2025

Local Area Needs Assessment (LANA) Framework

Making Tracks Together: Queensland's Aboriginal and Torres Strait Islander Health Equity Framework

Making Tracks toward closing the gap in health outcomes for Indigenous Queenslanders by 2033 - Policy and Accountability Framework

Aboriginal and Torres Strait Islander Health Workforce Strategic Framework 2016-2026

National Agreement on Closing the Gap

Queensland Health Workforce Diversity and Inclusion Strategy 2017 to 2022

Performance Measures Attribute Sheets

Purchasing Initiatives and Funding Specifications

Public Health Practice Manual

National Partnership on COVID-19 Response

Statewide services reference material

Service agreement amendment processes

Data supply requirements

Contracting and Performance Management Branch
GPO Box 48
Brisbane QLD 4001 Australia

Tel (+61) (07) 3708 5869

www.health.qld.gov.au

Service Agreement

2019/20 – 2021/22

North West Hospital and Health Service

July 2020 Revision

Queensland Government

North West Hospital and Health Service

Service Agreement 2019/20 - 2021/22, July 2020 Revision

Published by the State of Queensland (Queensland Health), July 2020

This document is licensed under a Creative Commons Attribution 3.0 Australia licence. To view a copy of this licence, visit creativecommons.org/licenses/by/3.0/au

© State of Queensland (Queensland Health) **2020**

You are free to copy, communicate and adapt the work, as long as you attribute the State of Queensland (Queensland Health).

For more information contact:
Contracting and Performance Management Branch, Department of Health, GPO Box 48, Brisbane QLD 4001, email SAM@health.qld.gov.au, phone (07) 3708 5869.

An electronic version of this document is available at www.health.qld.gov.au/system-governance/health-system/managing/default.asp

Disclaimer:
The content presented in this publication is distributed by the Queensland Government as an information source only. The State of Queensland makes no statements, representations or warranties about the accuracy, completeness or reliability of any information contained in this publication. The State of Queensland disclaims all responsibility and all liability (including without limitation for liability in negligence) for all expenses, losses, damages and costs you might incur as a result of the information being inaccurate or incomplete in any way, and for any reason reliance was placed on such information.

Contents

1. Introduction ... 6
2. Interpretation ... 6
3. Legislative and regulatory framework ... 7
4. Health system priorities ... 7
5. Objectives of the Service Agreement ... 9
6. Scope ... 9
7. Performance and Accountability Framework ... 9
8. Period of this Service Agreement ... 10
9. Amendments to this Service Agreement ... 10
10. Publication of amendments ... 10
11. Cessation of service delivery ... 10
12. Commencement of a new service ... 11
13. Provision of data to the Chief Executive ... 12
14. Dispute resolution ... 12
15. Force Majeure ... 15
16. Hospital and Health Service accountabilities ... 16
17. Department accountabilities ... 16
18. Insurance ... 18
19. Indemnity ... 18
20. Indemnity arrangements for officers, employees and agents ... 19
21. Legal proceedings ... 19
22. Sub-contracting ... 19
23. Counterparts ... 20

Execution ... 21

Schedule 1 HHS Accountabilities ... 22

1. Purpose ... 22
2. Registration, credentialing and scope of clinical practice ... 22
3. Clinical Services Capability Framework ... 22
4. Clinical Prioritisation Criteria ... 23
5. Service delivery ... 23
6. Accreditation ... 24
7. Responsive regulatory process for accreditation ... 25
8. Achieving health equity for First Nations Queenslanders ... 26
9. Provision of Clinical Products/Consumables in outpatient settings ... 27
10. Capital, land, buildings, equipment and maintenance ... 28
11. Occupational health and safety ... 30
12. Workforce management ... 30
13. Medically authorised ambulance transports ... 31

Schedule 2 Funding, purchased activity and services ... 32

1. Purpose ... 32
2. Delivery of purchased activity ... 32
3. Financial adjustments ... 33

4. Funding sources 46
5. Funds disbursement 48
6. Purchased services 57
7. Teaching training and research 62
Schedule 3 Performance Measures 65
1. Purpose 65
2. Performance Measures 65
Schedule 4 Data Supply Requirements 69
1. Purpose 69
2. Principles 69
3. Roles and responsibilities 69
Attachment A Data Supply Requirements 71
Schedule 5 Amendments to this Service Agreement 73
1. Purpose 73
2. Principles 73
3. Process to amend this Service Agreement 73
Schedule 6 Definitions 78
Key Documents 85
Abbreviations 87

Figures

Figure 1 Dispute resolution process 13
Figure 2 Inter-HHS dispute resolution process 15
Figure 3 Amendment Proposal negotiation and resolution 75

Tables

Table 1 Specific Funding Commitments 34
Table 2 Purchasing Incentives 2020/21 (Summary) 45
Table 3 Hospital and Health Service funding sources 2020/21 47
Table 4 HHS Finance and Activity Schedule 2019/20 – 2021/22 – Summary by Purchasing Hierarchy 49
Table 5 Minor Capital and Equity 51
Table 6 HHS Finance and Activity Schedule 2019/20 – 2021/22 Other Funding Detail 52
Table 7 Specified Grants 55
Table 8 Hospital and Health Service Service Agreement and State Level Block Payments to state managed funds from Commonwealth payments into national funding pool 56
Table 9 HHS Performance Measures – Key Performance Indicators 66
Table 10 HHS Performance Measures - Safety and Quality Markers 67
Table 11 HHS Performance Measures – Outcome Indicators 68
Table 12 Clinical data 71
Table 13 Non-clinical data 72
Table 14 Amendment Window Exchange Dates 74

1. Introduction

1.1 The Queensland Public Sector Health System is committed to strengthening performance and improving services and programs in order to meet the needs of the community and deliver improved health outcomes to all Queenslanders.

1.2 The development of Service Agreements between the Chief Executive and Hospital and Health Services (HHSs), assists this process by formally assigning accountability for the high-level outcomes and targets to be met during the period to which the Service Agreement relates.

1.3 The content and process for the preparation of this Service Agreement is consistent with the requirements of the *Hospital and Health Boards Act 2011*. As such this Service Agreement specifies:

(a) the Health Services and other services to be provided by the HHS;

(b) the funding which is provided to the HHS for the provision of these services and the way in which the funding is to be provided;

(c) the Performance Measures that the HHS will meet for the services provided;

(d) data supply requirements; and

(e) other obligations of the Parties.

1.4 Fundamental to the success of this Service Agreement is a strong collaboration between the HHS and its Board and the Department. This collaboration is supported through regular Performance Review Meetings attended by representatives from both the HHS and the Department which provide a forum within which a range of aspects of HHS and system wide performance are discussed and jointly addressed.

2. Interpretation

Unless expressed to the contrary, in this Service Agreement:

(a) words in the singular include the plural and vice versa;

(b) any gender includes the other genders;

(c) if a word or phrase is defined its other grammatical forms have corresponding meanings;

(d) “includes” and “including” are not terms of limitation;

(e) no rule of construction will apply to a clause to the disadvantage of a Party merely because that Party put forward the clause or would otherwise benefit from it;

(f) a reference to:

(i) a Party is a reference to a Party to this Service Agreement;

(ii) a person includes a partnership, joint venture, unincorporated association, corporation and a government or statutory body or authority;

(iii) a person includes the person's legal personal representatives, successors, assigns and persons substituted by novation;

(g) any legislation includes subordinate legislation under it and includes that legislation and subordinate legislation as modified or replaced;

(h) a reference to a role, function or organisational unit is deemed to transfer to an equivalent successor role, function or organisational unit in the event of organisational change or restructure in either Party;

(i) an obligation includes a warranty or representation and a reference to a failure to comply with an obligation includes a breach of warranty or representation;

(j) headings do not affect the interpretation of this Service Agreement;

(k) unless the contrary intention appears, a reference to a Schedule, annexure or attachment is a reference to a Schedule, annexure or attachment to this Service Agreement; and

(l) unless the contrary intention appears, words in the Service Agreement that are defined in Schedule 6 'Definitions' have the meaning given to them in that Schedule.

3. Legislative and regulatory framework

3.1 This Service Agreement is regulated by the National Health Reform Agreement and the provisions of the *Hospital and Health Boards Act 2011.*

3.2 The National Health Reform Agreement requires the State of Queensland to establish Service Agreements with each HHS for the purchasing of health services and to implement a performance and accountability framework including processes for remediation of poor performance. The *Hospital and Health Boards Act 2011* states under section 35(3) that the Service Agreement executed between the Chief Executive and the Hospital and Health Board Chair binds each of them.

3.3 The *Hospital and Health Boards Act 2011* recognises and gives effect to the principles and objectives of the national health system agreed by the commonwealth, state and territory governments, including the Medicare principles and health system principles set out in section 4. Section 5 of the *Hospital and Health Boards Act 2011* states that the object of the Act is to establish a Public Sector Health System that delivers high-quality hospital and other Health Services to persons in Queensland having regard to the principles and objectives of the national health system. This Service Agreement is an integral part of implementing these objectives and principles.

4. Health system priorities

4.1 Ensuring the provision of Public Sector Health Services across Queensland requires clear priorities, supportive leadership and staff who work together and across each level of the Public Sector Health System. The Parties recognise that they each have a mutual and reciprocal obligation to work collaboratively with each other, with other Hospital and Health

Services (HHS) and with the Queensland Ambulance Service in the best interests of the Queensland Public Sector Health System.

4.2 The priorities, goals and outcomes for the Queensland Public Sector Health System are defined through:

(a) *Our Future State: Advancing Queensland's Priorities* - the Queensland Government's objectives for the community; and

(b) *My health, Queensland's future: Advancing health 2026* – the vision and strategy for Queensland's health system.

4.3 The Parties will also work collaboratively to deliver the *Queensland Health 2020/21 System Priorities.* The *Queensland Health 2020/21 System Priorities* establishes a tactical framework which will ensure that the Queensland Public Sector Health System delivers sustainable, high quality and timely Health Services during 2020/21, whilst remaining positioned to respond effectively to the COVID-19 pandemic.

4.4 Additionally, the Queensland Government, Premier or the Minister for Health and Minister for Ambulance Services (The Minister) may articulate key priorities, themes and issues from time to time.

4.5 HHSs have a responsibility to ensure that the delivery of Public Sector Health Services in Queensland is consistent with these strategic directions and priorities.

4.6 The Parties will collectively identify, develop, implement and evaluate strategies that support the delivery of priorities identified by the Minister, and which align with a Value-Based Healthcare approach to the delivery of Health Services.

4.7 In accordance with section 9 of the *Financial and Performance Management Standard 2009*, HHSs are required to develop a strategic plan. The HHS's strategic plan will reflect local priorities and will be developed considering the shared Queensland priorities outlined in the Queensland Government's objectives for the community, the Ministers' articulated priorities and *My health, Queensland's future: Advancing health 2026*.

4.8 The Parties have a collective responsibility to contribute to a sustainable Public Sector Health System in Queensland. Planning and delivery of Health Services will be aligned with the system planning agenda set out in *Queensland Health System Outlook to 2026 for a sustainable health service* in order to ensure a coordinated, system-wide response to growing demand for Health Services.

4.9 In delivering Health Services, HHSs are required to meet the applicable conditions of all national agreements and national partnership agreements between the Queensland Government and the Commonwealth Government and commitments under any related implementation plans.

4.10 This Service Agreement is underpinned by and is to be managed in line with the following supporting documents:

(a) Queensland Health System Outlook to 2026 for a sustainable health service;

(b) Performance and Accountability Framework 2020/21; and

(c) Purchasing Policy and Funding Guidelines 2020/21.

5. Objectives of the Service Agreement

This Service Agreement is designed to:

(a) specify the Health Services, teaching, research and other services to be provided by the HHS;

(b) specify the funding to be provided to the HHS for the provision of the services;

(c) specify the Performance Measures for the provision of the services;

(d) specify the performance and other data to be provided by the HHS to the Chief Executive;

(e) provide a platform for greater public accountability; and

(f) facilitate the achievement of State and Commonwealth Government priorities, services, outputs and outcomes while ensuring local input.

6. Scope

6.1 This Service Agreement outlines the services that the Department will purchase from the HHS during the period of this Service Agreement.

6.2 This Service Agreement does not cover the provision of clinical and non-clinical services by the Department, including the Queensland Ambulance Service, to the HHS. Separate arrangements will be established for those services provided by Health Support Queensland and eHealth Queensland.

7. Performance and Accountability Framework

7.1 The Performance and Accountability Framework sets out the framework within which the Department, as the overall manager of Public Health System Performance, monitors and assesses the performance of Public Sector Health Services in Queensland. The systems and processes employed for this purpose include, but are not limited to, assessing and monitoring HHS performance, reporting on HHS performance and, as required, intervening to manage identified performance issues.

7.2 During 2020/21 the Performance and Accountability Framework will support delivery of the *Queensland Health System Priorities 2020/21* which focus on realising positive changes to the Public Sector Health System through providing sustainable, timely, safe and high-quality Health Services in the right setting whilst remaining ready to respond to the COVID-19 pandemic.

7.3 The Performance and Accountability Framework uses Performance Measures to monitor the extent to which HHSs are delivering the high-level objectives set out in this Service Agreement. The Key Performance Indicators and other measures of performance against which the HHS will be assessed and benchmarked are detailed in Schedule 3 of this Service Agreement.

7.4 The Parties agree to constructively implement the Performance and Accountability Framework.

8. Period of this Service Agreement

8.1 This Service Agreement commences on the Effective Date and expires on 30 June 2022. The Service Agreement framework is in place for three years in order to provide HHSs with a level of guidance regarding funding and purchased activity for the outer years.

8.2 In this Service Agreement, references to years are references to the period commencing on 1 July and ending on 30 June unless otherwise stated.

8.3 Using the provisions of the *Hospital and Health Boards Act 2011* as a guide, the Parties will enter into funding and purchased activity negotiations for the following year six months before the end of the current year.

8.4 In accordance with the *Hospital and Health Boards Act 2011* the Parties will enter negotiations for the next Service Agreement at least six months before the expiry of the existing Service Agreement.

9. Amendments to this Service Agreement

9.1 Section 39 of the *Hospital and Health Boards Act 2011* requires that, if the Chief Executive or the HHS wish to amend the terms of a Service Agreement, the Party wishing to amend the Service Agreement must give written notice of the proposed amendment to the other Party.

9.2 The process for amending this Service Agreement is set out in Schedule 5 of this Service Agreement.

10. Publication of amendments

The Department will publish each executed Deed of Amendment within 14 days of the date of execution on www.health.qld.gov.au/system-governance/health-system/managing/default.asp.

11. Cessation of service delivery

11.1 The HHS is required to deliver the Health Services and other services outlined in this Service Agreement for which funding is provided in Schedule 2. Any changes to service delivery must ensure maintenance of care and minimise disruptions to patients.

11.2 The Department and HHS may Terminate or temporarily Suspend a Health Service or other service by mutual agreement having regard to the following obligations:

(a) any proposed Termination or Suspension must be made in writing to the other Party;

(b) where it is proposed to Terminate or Suspend a Statewide Service, or a Regional Service, the HHSs which are in receipt of that service must also be consulted;

(c) the Parties must agree on a reasonable notice period following which Termination, or Suspension, will take effect; and

(d) patient needs, workforce implications, relevant government policy and HHS sustainability are to be considered.

11.3 The Department, in its role as the Queensland Public Health System manager:

(a) may, in its unfettered discretion, not support a requested Termination or Suspension and require the HHS to maintain the service; and

(b) will reallocate existing funding and activity for the Terminated or Suspended service inclusive of baseline Service Agreement funding and in-year growth funding on a pro-rata basis.

11.4 The HHS will:

(a) work with the Department to ensure continuity of care and a smooth transfer of the service to an alternative provider where this is necessary; and

(b) minimise any risk or inconvenience to patients associated with service Termination, Suspension or transfer.

11.5 In the event that a sustainable alternative provider cannot be identified, and this is required, the service and associated patient cohort will continue to remain the responsibility of the HHS.

12. Commencement of a new service

12.1 In the event that the HHS wishes to commence providing a new Health Service, the HHS will notify the Department in writing in advance of commencement.

12.2 The Department will provide a formal response regarding the proposed new Health Service to the HHS in writing. The Department may not agree to purchase the new Health Service or to provide funding on either a recurrent or non-recurrent basis.

12.3 In the event that a change to an established Referral Pathway is proposed which would result in the direction of patient referrals to an alternative HHS on a temporary or a permanent basis:

(a) the new Referral Pathway must be agreed by all impacted HHSs prior to its implementation; and

(b) following agreement of the new Referral Pathway, if there is an identifiable and agreed impact to funding the Department will redistribute funding and activity between HHSs in alignment with new Referral Pathway.

13. Provision of data to the Chief Executive

The HHS will provide to the Chief Executive the performance data and other data, including data pursuant to ad hoc requests, set out in Schedule 4 of this Service Agreement in accordance with the Schedule, including in relation to the form, manner and the times required for the provision of data.

14. Dispute resolution

14.1 The dispute resolution process set out below is designed to resolve disputes which may arise between the Parties to this Service Agreement in a final and binding manner.

14.2 These procedures and any disputes addressed or to be addressed by them are subject to the provisions of the *Hospital and Health Boards Act 2011*, including in respect of any directions issued under that legislation or by Government in respect of any dispute.

14.3 Resolution of disputes will be through a tiered process commencing with the Performance Review Meeting and culminating, if required, with the Minister, as illustrated in Figure 1.

14.4 Use of the dispute resolution process set out in this clause should only occur following the best endeavours of both Parties to agree a resolution to an issue at the local level. The dispute resolution process is not intended for the resolution of ongoing issues or performance related issues. At each stage of the dispute resolution process, the Parties agree to cooperate.

14.5 If a dispute arises in connection with this Service Agreement (including in respect of interpretation of the terms of this Service Agreement), then either Party may give the other a written Notice of Dispute.

14.6 The Notice of Dispute must be provided to the D-SA Contact Person if the notice is being given by the HHS and to the HHS-SA Contact Person if the notice is being given by the Department.

14.7 The Notice of Dispute must contain the following information:

(a) a summary of the matter in dispute;

(b) an explanation of how the Party giving the Notice of Dispute believes the dispute should be resolved and reasons to support that belief;

(c) any information or documents to support the Notice of Dispute; and

(d) a definition and explanation of any financial or Service delivery impact of the dispute.

Figure 1 Dispute resolution process

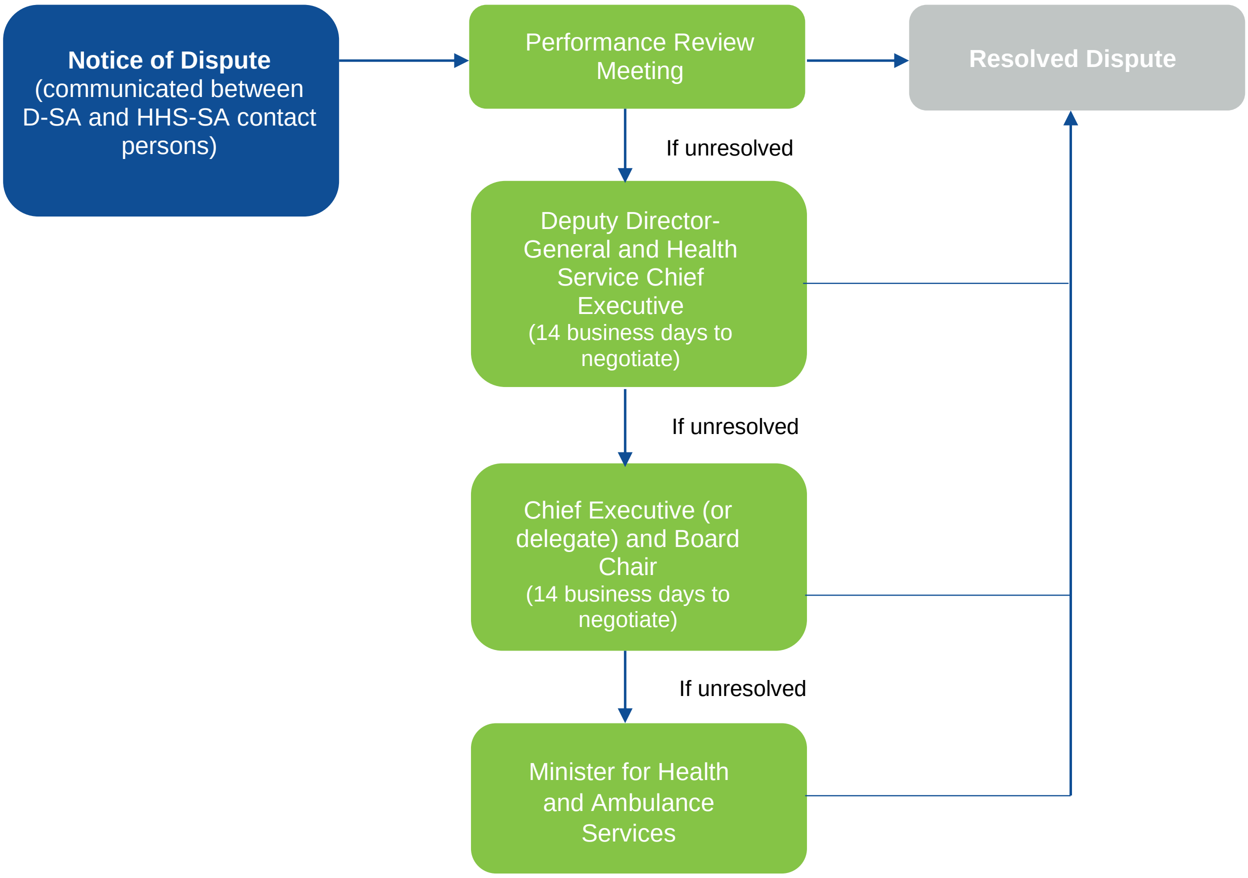

14.8 Resolution of a dispute

(a) Resolution of a dispute at any level is final. The resolution of the dispute is binding on the Parties but does not set a precedent to be adopted in similar disputes between other Parties.

(b) The Parties agree that each dispute (including the existence and contents of each Notice of Dispute) and any exchange of information or documents between the Parties in connection with the dispute is confidential and must not be disclosed to any third party without the prior written consent of the other Party, other than if required by law and only to the extent required by law.

14.9 Continued performance

Notwithstanding the existence of one or more disputes, the HHS must continue to perform and comply with this Service Agreement to the best of their abilities given the circumstances.

14.10 Disputes arising between Hospital and Health Services

(a) In the event of a dispute arising between two or more HHSs (an Inter-HHS Dispute), the process set out in Figure 2 will be initiated. Resolution of Inter-HHS Disputes will be through a tiered process, commencing with local resolution and culminating if required with formal and binding arbitration by the Minister under the provisions of the *Hospital and Health Boards Act 2011*, section 44.

(b) If the HHS wishes to escalate a dispute, the HHS will be expected to demonstrate that best endeavours (including Chair and Board involvement) to resolve the dispute between all parties at an informal and local level have taken place.

(c) Management of inter-HHS relationships should be informed by the following principles:

(i) HHSs should maintain (for both the base level of funding and growth) the proportion of out of HHS work undertaken unless as a result of agreed repatriation of patients.

(ii) All HHSs manage patients from their own catchment population if it is within their clinical capability to do so as specified by the Clinical Services Capability Framework.

(iii) Where it is proposed that a Health Service move from one HHS to another, agreement between the respective Health Service Chief Executives will be secured prior to any change in patient flows. Once agreed, funding will follow the patient.

(iv) All HHSs abide by the agreed dispute resolution process.

(v) All HHSs operate in a manner which is consistent with the health system principles and objectives as set out in the National Health Reform Agreement and the *Hospital and Health Boards Act 2011*.

Figure 2 Inter-HHS dispute resolution process

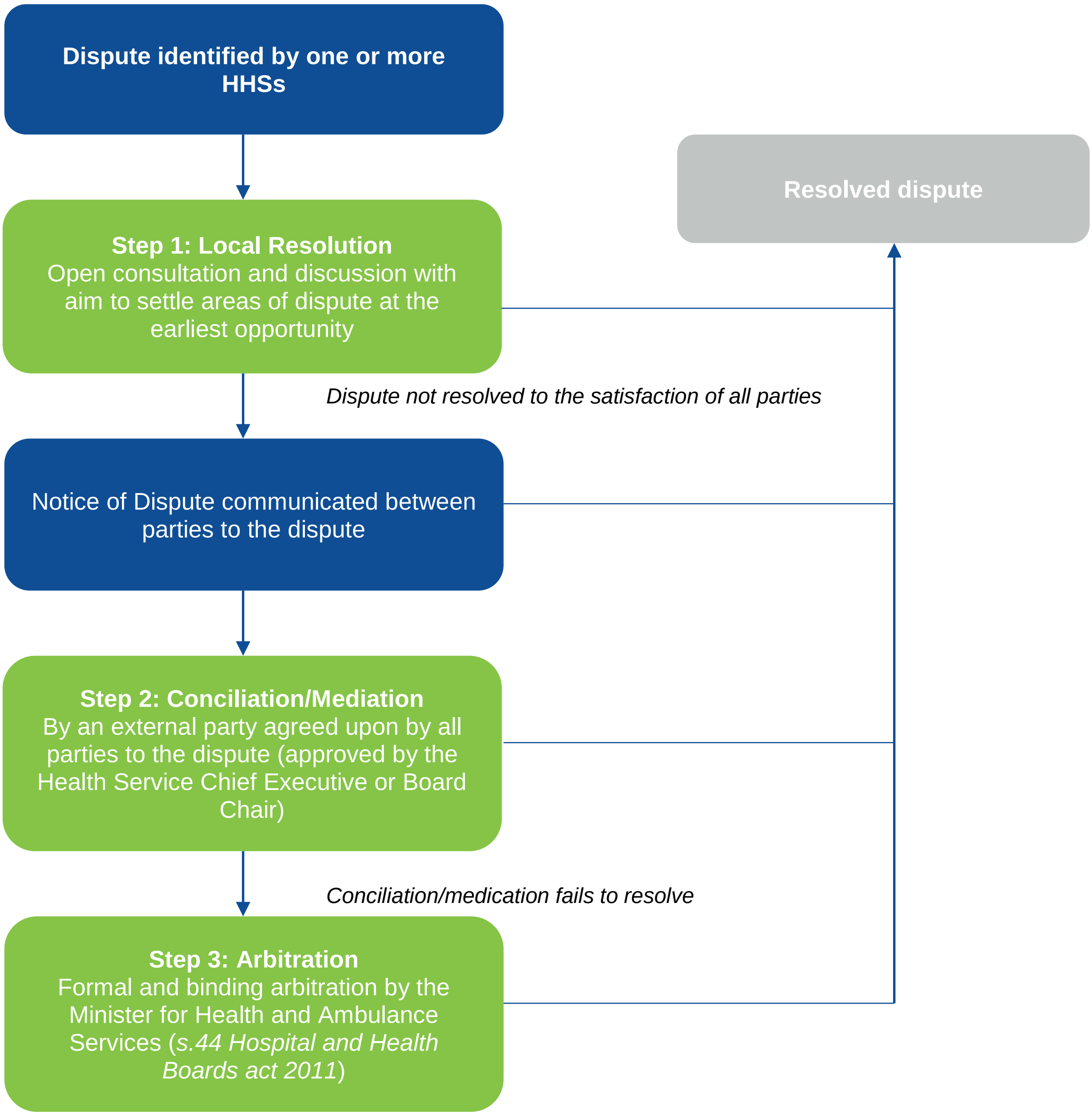

15. Force Majeure

15.1 If a Party (Affected Party) is prevented or hindered by Force Majeure from fully or partly complying with any obligation under this Service Agreement, that obligation may (subject to the terms of this Force Majeure clause) be suspended, provided that if the Affected Party wishes to claim the benefit of this Force Majeure clause, it must:

(a) give prompt written notice of the Force Majeure to the other Party of:

(i) the occurrence and nature of the Force Majeure;

(ii) the anticipated duration of the Force Majeure;

(iii) the effect the Force Majeure has had (if any) and the likely effect the Force Majeure will have on the performance of the Affected Party's

obligations under this Service Agreement; and

(iv) any disaster management plan that applies to the party in respect of the Force Majeure.

(b) use its best endeavours to resume fulfilling its obligations under this Service Agreement as promptly as possible; and

(c) give written notice to the other Party within five days of the cessation of the Force Majeure.

15.2 Without limiting any other powers, rights or remedies of the Chief Executive, if the Affected Party is the HHS and the delay caused by the Force Majeure continues for more than 14 days from the date that the Chief Executive determines that the Force Majeure commenced, the Chief Executive may give directions to the HHS regarding the HHS's performance or non-performance of this Service Agreement during the Force Majeure and the HHS must comply with that direction.

15.3 Neither Party may terminate this Service Agreement due to a Force Majeure event.

16. Hospital and Health Service accountabilities

16.1 Without limiting any other obligations of the HHS, it must comply with:

(a) the terms of this Service Agreement;

(b) all legislation applicable to the HHS, including the *Hospital and Health Boards Act 2011*;

(c) all Cabinet decisions applicable to the HHS;

(d) all Ministerial directives applicable to the HHS;

(e) all agreements entered into between the Queensland and Commonwealth governments applicable to the HHS;

(f) all regulations made under the *Hospital and Health Boards Act 2011*;

(g) all Industrial Instruments applicable to the HHS; and

(h) all health service directives applicable to the HHS.

16.2 The HHS will ensure that the accountabilities set out in Schedule 1 of this Service Agreement are met.

17. Department accountabilities

17.1 Without limiting any other obligations of the Department, it must comply with:

(a) the terms of this Service Agreement;

(b) the legislative requirements as set out within the *Hospital and Health Boards Act 2011*;

(c) all regulations made under the *Hospital and Health Boards Act 2011*; and

(d) all Cabinet decisions applicable to the Department.

17.2 The Department will work in collaboration with HHSs to ensure the Public Sector Health System delivers high quality hospital and other Health Services to persons in Queensland having regard to the principles and objectives of the national health system. In support of realising this objective, in accordance with section 5 of the *Hospital and Health Boards Act 2011* the Department will:

(a) provide overall management of the Queensland Public Sector Health System including health system planning, coordination and standard setting;

(b) provide the HHS with funding specified under Schedule 2 of this Service Agreement;

(c) provide and maintain payroll and rostering systems to the HHS unless agreed otherwise between the Parties;

(d) operate 13 HEALTH as a first point of contact for health advice with timely HHS advice and information where appropriate to local issues; and

(e) balance the benefits of a local and system-wide approach.

17.3 The Department will endeavour to purchase services in line with Clinical Prioritisation Criteria, where these have been developed, in order to improve equity of access and reflect the scope of publicly funded services.

17.4 The Department will maintain a public record of the Clinical Service Capability Framework levels for all public facilities based on the information provided by HHSs.

17.5 **Workforce management**

The Chief Executive agrees to appoint Health Service Employees to:

(a) perform work for the HHS for the purpose of enabling the HHS to perform its functions and exercise powers under the *Hospital and Health Boards Act 2011;* and

(b) deliver the services specified in this Service Agreement.

17.6 The Chief Executive, whether in the capacity of a Person Conducting a Business or Undertaking as an employer or as a building owner or occupier, will consult, cooperate and coordinate with the HHS to ensure legal compliance with the *Work Health and Safety Act 2011* and other legislation as it applies to the scope, nature and location of operations associated with this Service Agreement. This includes, but is not limited to:

(a) codes of practice;

(b) electrical safety legislation;

(c) building and fire safety legislation; and

(d) workers' compensation legislation.

18. Insurance

18.1 The HHS must hold and maintain for the period of this Service Agreement the types and levels of insurances that the HHS considers appropriate to cover its obligations under this Service Agreement.

18.2 Without limiting the types and levels of insurances that the HHS considers appropriate, any insurance policies taken out by the HHS under this clause must include appropriate coverage for the following:

(a) public and product liability insurance;

(b) professional indemnity insurance; and

(c) workers' compensation insurance in accordance with the *Workers' Compensation and Rehabilitation Act 2003* (Qld).

18.3 The HHS will be deemed to comply with its requirements under clauses 18.1 and 18.2(a) and 18.2(b) if it takes out and maintains a current insurance policy with the Queensland Government Insurance Fund.

18.4 Any insurance policies held by the HHS pursuant to this clause must be effected with an insurer that is authorised and licensed to operate in Australia.

18.5 The HHS must maintain a current register of all third-party guarantees.

18.6 The HHS must, if requested by the Department, promptly provide a sufficiently detailed certificate of currency and/or insurance and policy documents for each insurance policy held by the HHS pursuant to this clause.

18.7 The HHS warrants that any exclusions and deductibles that may be applicable under the insurance policies held pursuant to this clause will not impact on the HHS's ability to meet any claim, action or demand or otherwise prejudice the Department's rights under this Service Agreement.

18.8 The HHS must immediately advise the Department if any insurance policy, as required by this clause, is materially modified or cancelled.

19. Indemnity

19.1 The HHS indemnifies the Department against all and any liabilities, claims, actions, demands, costs and expenses made by any person which may be brought against or made upon or incurred by the Department arising directly or indirectly from or in connection with any of the following:

(a) any wilful, unlawful or negligent act or omission of the HHS, a Health Service Employee, Health Executive, Senior Health Service Employee or an officer, employee or agent working for the HHS in the course of the performance or attempted or purported performance of this Service Agreement;

(b) any penalty imposed for breach of any applicable law in relation to the HHS's performance of this Service Agreement; and

(c) a breach of this Service Agreement,

except to the extent that any act or omission by the Department caused or contributed to the liability, claim, action, demand, cost or expense.

19.2 The indemnity referred to in this clause will survive the expiration or termination of this Service Agreement.

20. Indemnity arrangements for officers, employees and agents

20.1 Indemnity arrangements for officers, employees or agents working for the Public Sector Health System are administered in accordance with the following policy documents, as amended from time to time:

(a) Indemnity for Queensland Health Medical Practitioners HR Policy I2 (QH-POL-153:2014); and

(b) Queensland Government Indemnity Guideline.

20.2 The costs of indemnity arrangements provided for Health Service Employees, Health Executives, Senior Health Service Employees, or officers, employees or agents working for the HHS are payable by the HHS.

21. Legal proceedings

21.1 This clause applies if there is any demand, claim, liability or legal proceeding relating to assets, contracts, agreements or instruments relating to the HHS, whether or not they are:

(a) transferred to an HHS under section 307 of the *Hospital and Health Boards Act 2011*; or

(b) retained by the Department.

21.2 Subject to any law, each party must (at its own cost) do all things, execute such documents and share such information in its possession and control that is relevant, and which is reasonably necessary, to enable the other party to institute or defend (as the case may be) any demand, claim, liability or legal proceeding.

22. Sub-contracting

22.1 The Parties acknowledge that the HHS may sub-contract the provision of Health Services and other services that are required to be performed by the HHS under this Service Agreement.

22.2 The HHS must ensure that any sub-contractor who has access to confidential information (as defined in section 139 of the *Hospital and Health Boards Act 2011*) and/or personal information (as defined in section 12 of the *Information Privacy Act 2009*) complies with obligations no less onerous than those imposed on the HHS.

22.3 The HHS agrees that the sub-contracting of services:

(a) will not transfer responsibility for provision of the services to the sub-contractor; and

(b) will not relieve the HHS from any of its liabilities or obligations under this Service Agreement, including but not limited to obligations concerning the provision of data in accordance with Schedule 4 (Data Supply Requirements).

23. Counterparts

23.1 This Service Agreement may be executed in two or more identical copy counterparts, each of which together will be deemed an original, but all of which together will constitute one and the same instrument.

23.2 In the event that any signature executing this Service Agreement or any part of this Service Agreement is delivered by facsimile transmission or by scanned e-mail delivery of a '.pdf' format data file or equivalent, the signature will create a valid and binding obligation of the Party executing (or on whose behalf the signature is executed) with the same force and effect as if the signature page were an original.

23.3 For execution under this clause 23 to be valid the entire Service Agreement upon execution by each individual Party must be delivered to the remaining parties.

Execution

A. The terms of this Service Agreement were agreed under the provisions set out in the *Hospital and* Health *Boards Act,* section 35 on 27 June 2019, and were subsequently amended by the Deed of Amendment entered into pursuant to section 39 of the *Hospital and Health Boards Act 2011* and executed on 10 January 2020; 15 May 2020; 2 June 2020; 25 June 2020 and 2 September 2020.

B. This revised Service Agreement consolidates amendments arising from:

- 2019/20 Amendment Window 2 (in-year variation);
- 2019/20 Amendment Window 3 (in-year variation);
- 2020/21 Amendment Window 1 (annual budget build);
- April 2020 Extra-ordinary Amendment Window; and
- May 2020 Extra-ordinary Amendment Window.

C. Execution source documents are available on the service agreement website https://www.health.qld.gov.au/system-governance/health-system/managing/agreements-deeds.

Schedule 1 HHS Accountabilities

1. Purpose

Without limiting any other obligations of the HHS, this Schedule 1 sets out the key accountabilities that the HHS is required to meet under the terms of this Service Agreement.

2. Registration, credentialing and scope of clinical practice

2.1 The HHS must ensure that:

(a) all persons (including off site reporting radiologists) who provide a clinical service for which there is a national or Queensland legal requirement for registration, have and maintain current registration throughout their employment and only practise within the scope of that registration;

(b) all persons who perform roles for which eligibility for membership of a professional association is a mandatory requirement, have and maintain current eligibility of membership of the relevant professional association throughout their employment in the role; and

(c) all persons who provide a clinical service, and who fall within the scope of current credentialing policies (i.e. including medical, dental, nursing, midwifery and allied health), have a current scope of clinical practice and practise within that scope of clinical practice (which includes practising within their registration conditions and within the scope of the Clinical Services Capability Framework of the facility/s at which the service is provided).

2.2 Confirmation of registration and/or professional memberships is to be undertaken in accordance with the processes outlined in 'Health Professionals Registration: medical officers, nurses, midwives and other health professionals HR Policy B14 (QH-POL-147:2016)', as amended from time to time.

3. Clinical Services Capability Framework

3.1 The HHS must ensure that:

(a) all facilities have undertaken a baseline self-assessment against the Clinical Services Capability Framework (version 3.2);

(b) the Department is notified when a change to the Clinical Services Capability Framework baseline self-assessment occurs through the established public hospital Clinical Services Capability Framework notification process; and

(c) in the event that a Clinical Services Capability Framework module is updated or a new module is introduced, a self-assessment is undertaken against the relevant module and submitted to the Department.

3.2 The HHS is accountable for attesting to the accuracy of the information contained in any Clinical Services Capability Framework self-assessment submitted to the Department.

4. Clinical Prioritisation Criteria

4.1 The HHS must ensure that:

(a) processes for access to specialist surgical and medical services in line with Clinical Prioritisation Criteria are implemented, where these have been developed, in order to improve equity of access to specialist services; and

(b) General Practice Liaison Officer and Business Practice Improvement Officer programs are maintained in order to deliver improved access to specialist outpatient services, including through (but not limited to) their contribution to the development and implementation of Statewide Clinical Prioritisation Criteria.

5. Service delivery

5.1 The HHS will work with collaboratively with other healthcare service providers to ensure that an integrated pathway of care is in place for patients. This will include, but is not limited to:

(a) other HHSs;

(b) Primary Care providers;

(c) non-government organisations; and

(d) private providers.

5.2 The HHS must ensure that:

(a) the Health Services and other outlined in this Service Agreement, for which funding is provided in Schedule 2 ‘Funding and Purchased Activity and Services’ continue to be provided;

(b) the obligations regarding the payment and planning for blood and blood products and best practice as set out under the National Blood Agreement are fulfilled for the facilities for which funding is provided; and

(c) the *Queensland Organ Donation Strategy 2018-2020* is implemented in order to support an increase in organ donation rates in Queensland.

5.3 Through accepting the funding levels defined in Schedule 2 of this Service Agreement, the HHS accepts responsibility for the delivery of the associated programs and reporting requirements to State and Commonwealth bodies as defined by the Department.

6. Accreditation

6.1 All Queensland public hospitals, day procedure services and health care centres (howsoever titled) managed within the framework of HHSs are to maintain accreditation under the Australian Health Service Safety and Quality Accreditation Scheme[1].

6.2 Accreditation will be assessed against the National Safety and Quality Health Service standards[2] (NSQHS) second edition.

6.3 Residential aged care facilities will maintain accreditation by the Aged Care Quality and Safety Commission (ACQSC).

6.4 General practices owned or managed by the HHS are to be externally accredited in accordance with the current edition of the Royal Australian College of General Practitioners (RACGP) accreditation standards and in line with the National General Practice Accreditation Scheme.

6.5 For the purpose of accreditation, the performance of the HHS against the NSQHS and the performance of general practices owned or managed by the HHS against the RACGP accreditation standards can only be assessed by accrediting agencies that are approved by the Australian Commission on Safety and Quality in Healthcare (ACSQHC).

6.6 The HHS will select their accrediting agency from among the approved accrediting agencies. The ACSQHC and the RACGP provide a list of approved accrediting agencies which are published on their respective websites (www.safetyandquality.gov.au and www.racgp.org.au.

6.7 If the HHS does not meet the NSQHS standards accreditation requirements, the HHS has 60 days to address any not met actions. If the HHS does not meet the other accreditation standards requirements (RACGP and ACQSC), a remediation period will be defined by the accrediting agency.

6.8 Following assessment against NSQHS, ACQSC and RACGP standards, the HHS will provide to the Executive Director, Patient Safety and Quality Improvement Service, Department.

(a) immediate advice if a significant patient risk (one where there is a high probability of a substantial and demonstrable adverse impact for patients) is identified during an onsite visit, also identifying the plan of action and timeframe to remedy the issue as negotiated between the surveyors/assessors and/or the respective accrediting agency and the HHS;

(b) a copy of any 'not met' reports within two days of receipt of the report by the HHS;

(c) the accreditation report within seven days of receipt of the report by the HHS; and

(d) immediate advice should any action be rated not-met by the accrediting agency following the remediation period of an accreditation event, resulting in the facility or service not being accredited. Responsive regulatory processes may be enacted under clause 7 below.

---

[1] www.safetyandquality.gov.au/our-work/assessment-to-the-nsqhs-standards/australian-health-service-safety-and-quality-accreditation-scheme/
[2] www.safetyandquality.gov.au/our-work/assessment-to-the-nsqhs-standards/

6.9 The award recognising that the facility or service has met the required accreditation standards will be issued by the assessing accrediting agency for the period determined by their respective accreditation scheme.

6.10 The HHS will apply to an approved accrediting agency for a re-accreditation assessment prior to the expiry of their current accreditation period.

6.11 Where the HHS funds non-government organisations to deliver health and human services the HHS will ensure, from the Effective Date of this Service Agreement, that:

(a) within 12 months HHS procurement processes and service agreements with contracted non-government organisations specify the quality accreditation requirements for mental health services as determined by the Department; and

(b) as the quality accreditation requirements for subsequent funded service types are determined by the Department, procurement processes and service agreements with contracted non-government organisations reflect these requirements within 12 months of their formal communication by the Department to HHSs.

7. Responsive regulatory process for accreditation

7.1 A responsive regulatory process is utilised in the following circumstances:

(a) where a significant patient risk is identified by a certified accrediting agency during an accreditation process; and/or

(b) where an HHS has failed to address ‘not met’ actions of the specified standards within required timeframes.

7.2 An initial regulatory response will begin with a process of verifying the scope, scale and implications of the reported issues, a review of documentation, and may include one or more site visits by nominated specialty experts.

7.3 The regulatory process may include one or a combination of the following actions:

(a) seek further information from the HHS;

(b) request a progress report for the implementation of an action plan;

(c) escalate non-compliance and/or risk to the Performance Review Meeting;

(d) provide advice, information on options or strategies that could be used to address the non-met actions within a designated time frame; and/or

(e) connect the hospital to other hospitals that have addressed similar deficits or have exemplar practice in this area.

7.4 In the case of serious or persistent non-compliance and where required action is not taken by the HHS the response may be escalated. The Department may undertake one or a combination of the following actions:

(a) restrict specified practices/activities in areas/units or services of the HHS where the specified standards have not been met;

(b) suspend particular services at the HHS until the area/s of concern are resolved; and

(c) suspend all service delivery at a facility within an HHS for a period of time.

8. Achieving health equity for First Nations Queenslanders

8.1 The *Queensland Health Statement of Action towards Closing the Gap in Health Outcomes* is a commitment to addressing systemic barriers that may in any way contribute to preventing the achievement of health equity for all First Nations people. The statement is expected to mobilise renewed efforts and prompt new strategies for achieving health equity for First Nations Queenslanders.

8.2 The HHS will develop a Health Equity Strategy (previously referred to as the Closing the Gap Health Plan) to demonstrate the HHS's activities towards achieving health equity for First Nations people. The Health Equity Strategy will supersede the existing Closing the Gap Health Plan and act as the principal accountability mechanism between community and Government in the pursuit of Health Equity for First Nations Queenslanders.

8.3 The Health Equity Strategy will:

(a) be co-designed, co-developed and co-implemented by the First Nations community and the HHS; and

(b) demonstrate an evidence-based approach to priority setting.

8.4 The HHS will review the Health Equity Strategy at least once every three years and will publish the Health Equity Strategy in a way that allows it to be accessed by members of the public.

8.5 In line with the *Queensland Health Statement of Action towards Closing the Gap in Health Outcomes,* the HHS will ensure that commitment and leadership is demonstrated through implementing actions outlined in the Health Equity Strategy. The actions will, at a minimum:

(a) promote and provide opportunities to embed the representation of First Nations people in leadership, governance and the workforce;

(b) improve local engagement and partnerships between the HHS and First Nations people, communities and organisations to enable co-design, co-development and co-implementation;

(c) improve transparency, reporting and accountability in Closing the Gap progress; and

(d) demonstrate co-design, co-development, co-implementation and co-leadership of health programs and strategies.

8.6 The HHS will:

(a) establish and maintain an ongoing dialogue with the Department to progress and report on key actions taken and Health Service initiatives aligned to the *Queensland Health Statement of Action towards Closing the Gap in Health Outcomes*;

(b) establish and maintain an ongoing dialogue with the Department to progress and report on key actions taken and health service initiatives and strategies to attract,

recruit, support and retain a First Nations people workforce and workforce models commensurate to the HHS population and aligned to the benchmarks prescribed in the Workforce Diversity and Inclusion Strategy 2017-2022; and

(c) report publicly on progress against the Health Equity Strategy. Progress will be reported on an annual basis as a minimum.

9. Provision of Clinical Products/Consumables in outpatient settings

9.1 Upon discharge as an inpatient or outpatient, and where products/consumables are provided free of charge or at a subsidised charge, the Treating HHS will bear the initial costs of products/consumables provided to the patient/consumer as part of their care. These costs will be met by the Treating HHS for a sufficient period of time to ensure the patient/consumer incurs no disruption to their access to the Clinical Products/Consumables.

9.2 Unless otherwise determined by the HHS providing the Clinical Products/Consumables, ongoing direct costs (beyond an initial period following discharge as an inpatient) of the provided products/consumables will be borne by the Residential HHS of the outpatient/consumer.

9.3 Where guidelines exist (e.g. Guideline for Compression Garments for Adults with Lymphoedema: Eligibility, Supply and Costing and Guideline for Home Enteral Nutrition Services for Outpatients: Eligibility, Supply and Costing), standardised eligibility criteria and charges should apply.

9.4 Where a patient is supplied with medicines on discharge, or consequent to an outpatient appointment, that are being introduced to a patient’s treatment, the Treating HHS will provide prescription(s) and an adequate initial supply. This will comprise:

(a) for medicines reimbursable under the Pharmaceutical Benefits Scheme (PBS), including the Section 100 Highly Specialised Drugs Program – the quantity that has been clinically-appropriately prescribed or the maximum PBS supply, whichever is the lesser; or

(b) for non-reimbursable medicines, one month’s supply or a complete course of treatment, whichever is the lesser.

9.5 For medicines that are non-reimbursable under the PBS, and which are not included in the Queensland Health List of Approved Medicines (LAM), the Residential HHS will be responsible for ongoing supply, provided that the Treating HHS has provided the Residential HHS with documentary evidence of the gatekeeping approval at the Treating HHS for the non-LAM medicine. This evidence may be:

(a) a copy of the individual patient approval; or

(b) where the medicine is subject to a ‘blanket approval’ at the Treating HHS, a copy of the blanket approval, and a statement that the patient meets the criteria to be included under that approval.

9.6 This evidence is to be provided pro-actively to the Director of Pharmacy (or, for non-Pharmacist sites, the Director of Nursing and the HHS Director of Pharmacy) for the hospital nominated under clause 9.8 below.

9.7 For non-reimbursable medicines listed on the LAM for the condition being treated, the Residential HHS is responsible for ongoing supplies.

9.8 The Treating HHS will inform the patient about the ongoing supply arrangements and agree which hospital, within the patient's Residential HHS, they should attend for repeat supplies. The patient will be advised to contact the pharmacy at the nominated hospital regarding their requirements at least a week before attending for repeat supply.

9.9 PBS-reimbursable prescriptions issued by a public hospital may be dispensed at any other public hospital that has the ability to claim reimbursement. Patients may, in accordance with hospital policy, be encouraged to have their PBS prescriptions dispensed at a private pharmacy of their choice.

10. Capital, land, buildings, equipment and maintenance

10.1 **Capital**

(a) The HHS will:

(i) achieve annual capital expenditure within an acceptable variance to its allocation in the State's published Budget Paper 3 – Capital Statement, as specified in the capital expenditure performance KPI target.

(ii) record capital expenditure data in the capital intelligence portal each month. Data will be published through the System Performance Reporting (SPR) platform.

(iii) achieve all Priority Capital and Health Technology Equipment Replacement Program capital expenditure requirements and associated delivery milestones, as funded, and undertake all capital expenditure performance reporting requirements in the capital intelligence portal on a monthly basis.

(iv) comply with all other capital program reporting requirements, as identified in Schedule 4, Table 13.

10.2 **Asset Management**

(a) The Service Agreement includes funding provision for regular maintenance of the HHS's building portfolio.

(b) The Department has determined that a total sustainable budget allocation that equates to a minimum of 2.81% of the un-depreciated asset replacement value of the Queensland Public Health System's building portfolio is required to sustain the building assets to achieve expected life-cycles. The sustainable budget allocation is a combination of operational and capital maintenance funding.

(c) The HHS will conduct a comprehensive assessment of the maintenance demand for the HHS's building portfolio to ascertain the total maintenance funding

requirements of that portfolio. The assessment must identify the following for the portfolio:

(i) regulatory requirements;

(ii) best practice requirements;

(iii) condition-based requirements;

(iv) lifecycle planning requirements; and

(v) reactive maintenance estimates based on historical information, including backlog maintenance liabilities and risk mitigation strategies.

(d) The HHS will allocate an annual maintenance budget that reasonably takes into account the maintenance demand identified by the assessment in its reasonable considerations, without limiting the scope of such reasonable considerations including financial affordability linked to risk assessment. The annual maintenance budget will equate to either:

(i) 2.81% of the un-depreciated asset replacement value of the HHS's building portfolio; or

(ii) an alternative percentage amount determined by the HHSs as a result of its considerations.

(e) The HHS will submit an annual asset management and maintenance plan, approved by the Health Service Chief Executive, to the Department that:

(i) outlines the maintenance demand assessment undertaken by the HHS under Schedule 1, clause 10.2(c)

(ii) confirms the annual maintenance budget determined by the HHS under Schedule 1, clause 10.2(d)

(f) The HHS will submit an annual Statement of Building Portfolio Compliance to the Department for each year of the Term of this Service Agreement.

(g) The HHS will continue to proactively develop and address the recommendations within the final Asset Management Capability Report that was issued to the HHS as part of the transfer notice process.

10.3 **Property**

(a) The HHS will ensure building and infrastructure assets are managed in accordance with the specifications of any relevant transfer notices published as a gazette notice by the Minister under section 273A of the *Hospital and Health Boards Act 2011.*

(b) For land, buildings and parts of buildings where the Department is, or is intended to be, the exclusive occupier under specific occupancy or ground leases implemented pursuant to clauses 1.7 (c) and 1.8 respectively (where applicable) of a transfer notice, the Department is deemed to be in control of that land, building or part of a building for the purpose of work health and safety law.

10.4 Nothing in clause 10.3(b) of Schedule 1:

(a) removes any work health and safety responsibilities shared with another party or parties in accordance with work health and safety law; or

(b) limits the arrangements for the provision of work health and safety services provided in clause 11.

11. Occupational health and safety

11.1 The HHS, whether in the capacity of a Person Conducting a Business or Undertaking as an employer or as a building owner or occupier, will consult, cooperate and coordinate to ensure legal compliance with the *Work Health and Safety Act 2011* and other legislation as it applies to the scope, nature and location of operations associated with this Service Agreement. This includes, but is not limited to:

(a) codes of practice;

(b) electrical safety legislation;

(c) building and fire safety legislation; and

(d) workers' compensation legislation.

11.2 The HHS will establish, implement and maintain a health and safety management system which conforms to recognised health and safety management system standard AS/NZS 4801 *Occupational Health and Safety Management System* or ISO45001 *Occupational Health and Safety Management Systems* or another standard as agreed with the Chief Executive.

11.3 The HHS will monitor health and safety performance and will provide to the Chief Executive reports of a type, and at the intervals, agreed between the Parties, or as reasonably specified by the Chief Executive.

11.4 The Chief Executive will monitor health and safety performance at the system level. Where significant health and safety risks are identified, or performance against targets is identified as being outside tolerable levels, the Chief Executive may request further information from the HHS to address the issue(s) and/or make recommendations for action.

12. Workforce management

12.1 Subject to a delegation by the Chief Executive under section 46 of the *Hospital and Health Boards Act 2011*, the HHS is responsible for the day-to-day management (the HR Management Functions) of the Health Service Employees provided by the Chief Executive to perform work for the HHS under this Service Agreement.

(a) The HHS will exercise its decision-making power in relation to all HR Management Functions which may be delegated to it by the Chief Executive under section 46 of the *Hospital and Health Boards Act 2011*, in respect of the Health Service Employees, in a lawful and reasonable manner and with due diligence, and in accordance with:

(i) terms and conditions of employment specified by the Department in accordance with section 66 of the *Hospital and Health Boards Act 2011;*

(ii) health service directives, issued by the Chief Executive under section 47 of the *Hospital and Health Boards Act 2011*;

(iii) health employment directives, issued by the Chief Executive under section 51A of the *Hospital and Health Boards Act 2011;*

(iv) any policy document that applies to the Health Service Employee;

(v) any Industrial Instrument that applies to the Health Service Employee;

(vi) the relevant HR delegations manual; and

(vii) any other relevant legislation.

12.2 The HHS must ensure that Health Service Employees are suitably qualified to perform their required functions.

12.3 Persons appointed in an HHS as a Health Executive or Senior Health Service Employees are employees of the HHS

12.4 All HHSs will provide to the Chief Executive human resource, workforce, and health and safety reports of a type, and at the intervals, agreed between the Parties, or as reasonably specified by the Chief Executive.

13. Medically authorised ambulance transports

13.1 The HHS will:

(a) utilise the Queensland Ambulance Service (QAS) for all road ambulance services not provided by the HHS. This includes both paramedic level and patient transport level services where the patient requires clinical care;

(b) follow the *Medically Authorised Ambulance Transports Operational Standards* when utilising QAS services; and

(c) ensure that performance data for ambulance services authorised by the HHS is collected and provided to the Department in line with agreed data supply requirements.

Schedule 2 Funding, purchased activity and services

1. Purpose

This Schedule 2 sets out:

(a) The activity purchased by the Department from the HHS (Table 4, Table 6 and Table 8);

(b) The funding provided for delivery of the purchased activity (Table 4; Table 5; Table 6; and Table 7);

(c) Specific funding commitments (Table 1);

(d) The criteria and processes for financial adjustments associated with the delivery of purchased activity and specific funding commitments;

(e) The sources of funding that this Service Agreement is based on and the manner in which these funds will be provided to the HHS (Table 3); and

(f) An overview of the purchased Health Services and other services which the HHS is required to provide throughout the period of this Service Agreement.

2. Delivery of purchased activity

2.1 The Department and the HHS will monitor actual activity against purchased levels.

2.2 The HHS has a responsibility to actively monitor variances from purchased activity levels and will notify the Department immediately via the D-SA Contact Person as soon as the HHS becomes aware of significant variances.

2.3 The HHS will also notify the Department of deliberate changes to the consistent recording of activity within year that would result in additional activity being recorded for existing Health Services.

2.4 If the HHS wishes to move activity between purchased activity types and levels, for example, activity moving from outpatients to inpatients or from one inpatient Service Related Group (SRG) to another, the HHS must negotiate this with the Department based on a sound needs based rationale.

2.5 With the exception of the programs, services and projects that are specified in Table 1, during 2020/21 no financial adjustment will be applied where the HHS is unable to deliver or exceeds the activity that has been funded, in recognition of the Commonwealth Government's treatment of the National Health Reform Agreement to support the response to the COVID-19 pandemic.

2.6 The activity purchased through this Service Agreement for 2020/21 is based on the activity purchased recurrently in 2019/20 and includes the productivity dividend.

2.7 The activity purchased in the Service Agreement for 2021/22 will be based on the activity purchased recurrently in 2020/21 including the productivity dividend.

2.8 Any resulting changes to funding and purchased activity levels will be actioned through the processes outlined in Schedules 2 and 5 of this Service Agreement.

2.9 The Department is required to report HHS activity data to the Independent Hospital Pricing Authority and the Administrator of the National Health Funding Pool. The HHS will maintain its efforts to accurately record the delivered activity and submit this data to the Department in accordance with the requirements set out in Schedule 4.

2.10 The HHS should refer to the supporting document to this Service Agreement 'Healthcare Purchasing Policy and Funding Guidelines 2020/21' for details regarding the calculation of Weighted Activity Units. Supporting documents are available on-line as detailed in Appendix 1.

3. Financial adjustments

3.1 **Specific funding commitments**

(a) As part of the Service Agreement Value, the services, programs and projects set out in Table 1 have been purchased by the Department from the HHS. These services will be the focus of detailed monitoring by the Department.

(b) The HHS will promptly notify the D-SA Contact Person if the HHS forecasts an inability to achieve commitments linked to the specific funding commitments included in Table 1.

(c) On receipt of any notice under clause 3.1(b) of Schedule 2, it is at the discretion of the Chief Executive (or delegate) to withdraw allocated funding pro rata to the level of under delivery if the program is not being delivered according to the program objective or is not being delivered in full.

(d) If the Chief Executive (or delegate) decides to withdraw allocated funding, the Chief Executive (or delegate) will immediately issue an Adjustment Notice to the HHS-SA Contact Person confirming any adjustment that has been made in accordance with this clause 3.1 of Schedule 2. The Adjustment Notice will be issued through the process set out in clause 3.4 or 3.5 of Schedule 5.

(e) Following receipt of an Adjustment Notice under clause 3.1(d) of Schedule 2, the Parties will comply with the Adjustment Notice and immediately take steps necessary to give effect to the requirements of that Adjustment Notice.

(f) The Parties acknowledge that adjustments made under this clause 3.1 of Schedule 2 may vary the Service Agreement Value and/or a specific value recorded in Table 1.

(g) Where the Service Agreement Value and/or a specific value recorded in Table 1 is varied, the variation will be recorded in a Deed of Amendment issued following the next available Amendment Window.

Table 1 Specific Funding Commitments

Service/Program/Project	Funding	Activity	Timeframe	Conditions
Making Tracks towards closing the gap in health outcomes for Indigenous Queenslanders by 2033 – Investment Strategy 2018-21 including the *Queensland Aboriginal and Torres Strait Islander Rheumatic Heart Disease Action Plan 2018-2021*	*$3,722,254 ($3,640,040+ $82,214*)*<br>$2,609,177	*0*<br>0	*2019/20*<br>2020/21	The HHS will deliver the initiatives and outcomes outlined in the performance requirements issued by the Aboriginal and Torres Strait Islander Health Division in memorandum C-ECTF-19/5767.<br>Funding may be adjusted, and/or unspent funds redirected or recovered where project performance requirements are not met.<br>* Funding of $82,214 for the Discharge Against Medical Advice initiative deferred from 2018/19 to 2019/20.
North Queensland Aboriginal and Torres Strait Islander Sexually Transmissible Infections Action Plan 2016-2021	*$878,920* ($830,668+ $48,252)*<br>$830,668	*0*<br>0	*2019/20*<br>2020/21	The HHS will implement and support the required actions under the *North Queensland Aboriginal and Torres Strait Islander Sexually Transmissible Infections Action Plan 2016-2021* (the Plan) including the delivery of initiatives and outcomes outlined in memorandum CECTF- 19/5767.<br>Funding may be adjusted and/or unspent funds redirected or recovered where project performance requirements are not met.<br>*$48,252 - Transferred from Cairns and Hinterland Hospital and Health Service 2019/20 for Director Program management role.
• Contact Tracing Support Officer position	*$73,800*<br>$75,720	*0*<br>0	*2019/20*<br>2020/21	Progress with contact tracing is to be reported through the Plan.<br>If the position is not fully operational between 1 November 2018 and 30 June 2021, the HHS is to return unspent labour costs to the Communicable Diseases Branch.
High risk foot patients seen/managed within 48 hours of referral to ambulatory services	$81,721 (recurrent)	*17 WAUs (Q21)*	*2018/19*	The HHS will provide services as specified in the 2019/20 Ambulatory High-Risk Foot Services specification sheet published on QHEPS.
Connecting Care to Recovery 2016-2021: A plan for Queensland's Mental Health				
• Ed-LinQ Program	$162,500 (recurrent)	*0*	*2019/20*	Implement a new Ed-LinQ program in North West HHS.

Service/Program/Project	Funding	Activity	Timeframe	Conditions
Community Mental Health Growth Allocation	$221,400 (recurrent)	*0*	*2018/19*	Provision of funding to employ 2 additional Full Time Equivalents (FTEs) in support of the HHSs initiatives to enhance Community FTEs across Older Persons Mental Health Services and Child and Youth Mental Health Services.<br>Recruitment of FTEs to be monitored by the Mental Health Alcohol and Other Drugs Branch on a regular basis using the Mental Health Establishment Collection, with adjustments to be made in-year if FTEs not all recruited permanently.
Nurse Navigators	*$1,774,434*<br>$1,887,786<br>$1,887,786<br>$1,887,786 (recurrent)	*0*<br>0<br>0<br>0	*2019/20*<br>2020/21<br>2021/22<br>2022/23	The total Nurse Navigator Program allocation (2015/16 – 2019/20) is 6 NG7 and 3 NG8. All Nurse Navigator Program FTE is required to be appointed to a position ID that has 'Nurse Navigator' within the position title.<br>The HHS is ineligible to appoint Nurse Navigator Program FTE to any pre-existing permanent positions which have been renamed to include 'Nurse Navigator' in the position title.<br>The HHS is required to report monthly on:<br>• Employed Nurse Navigator FTE;<br>• Number of Nurse Navigator plans in place; and<br>• Number of patients seen by Nurse Navigators.
Nurse Graduates Reprovision	*$904,588*<br>$904,588	*0*<br>0	*2019/20*<br>2020/21	Non-recurrent reprovision of funding to provide continued support for the graduate nurse/midwife program.
Another 100 Midwives (Nursing)	*$870,406*<br>$890,695<br>$425,320	*0*<br>0<br>0	*2019/20*<br>2020/21<br>2021/22	The HHS will deliver the initiatives and outcomes outlined in the performance requirements as per memo C-ECTF-18/8074. Funding may be withdrawn if requirements are not met.

Service/Program/Project	Funding	Activity	Timeframe	Conditions
Evolve Therapeutic Services (ETS)	*$145,270*	*0*	*2019/20*	Provision of ETS within allocated resources and in line with the state-wide ETS Manual, noting variation in local contexts.<br>Reporting requirements as defined by the Mental Health, Alcohol and Other Drugs Branch.<br>If program performance requirements are not met in-year funding may be adjusted proportional to the under delivery against the agreed target.
Strength with Immersion Model (SwIM)<br>• Rural Generalist Skills Program, Clinical Immersions and Exchanges	*$110,000*<br>$144,987	*0*<br>0	*2019/20*<br>2020/21	The HHS will deliver the initiatives and outcomes outlined in the performance requirements as per memo C-ECTF-19/10193.
COVID-19 First Nations Response	$3,100,000	0	2020/21	The HHS will implement and deliver the required actions under the HHS First Nations COVID-19 response including the delivery of initiatives and outcomes outlined in memorandum C-ECTF-20-9652.<br>Funding is one-off in nature for discrete and time-limited activities that are directly attributable to managing the impacts of COVID-19 for First Nations peoples and must be in-scope under the existing financial guidelines for COVID-19 expenditure.<br>HHSs are to retain appropriate supporting documentation to substantiate all expenditure under the National Partnership Agreement.

Service/Program/Project	Funding	Activity	Timeframe	Conditions
Enterprise Bargaining (EB)	*$4,252,700*<br>$1,133,359<br>$479<br>(comprises both recurrent and non-recurrent funding)	*0*<br>0<br>0	*2019/20*<br>2020/21<br>2021/22	Funding has been allocated in full for the following EB agreements:<br>• *Nurses and Midwives (Queensland Health and Department of Education and Training) Certified Agreement (EB10) 2018* (Base wages and certain entitlements)*;* and<br>• *Medical Officers (Queensland Health) Certified Agreement (MOCA5) 2018.*<br>Legislative amendments have been introduced under the *Industrial Relations Act 2016* to give effect to a 2.5% increases under the following agreements as new agreements are yet to be certified:<br>• *Queensland Public Health Sector Certified Agreement (No.9) 2016;*<br>• *Queensland Health, Building, Engineering & Maintenance Services Certified Agreement (No.6) 2016;* and<br>• *Health Practitioners' and Dental Officers (Queensland Health) Certified agreement (No. 2) 2016.*<br>Funding which has been allocated recurrently in previous years has been recalled for the following streams as wage increase are not yet approved:<br>• HES-DSO;<br>• SES-SO; and<br>• VMO.<br>Subject to the terms and conditions of the agreements once executed a funding adjustment may be required. Full details can be found on the Budget and Analysis SharePoint platform.
Nurses and Midwives EB10 Innovation Fund:<br>• Digital Back to Country Project<br>• Implementation of InterRAI acute care assessment tool	*$172,595*<br>$37,826	*0*<br>0	*2019/20*<br>2020/21	Funding has been provided under the *Nurses and Midwives (Queensland Health and Department of Education) Certified Agreement (EB10) 2018,* clause 44.6, Innovation Fund.<br>The HHS will deliver the project, evaluation and reporting as outlined in memos:<br>• C-ECTF-19/8760; and<br>• C-ECTF-19/8764<br>Funding may be withdrawn if project requirements are not met.

Service/Program/Project	Funding	Activity	Timeframe	Conditions
Specialist Outpatient Strategy<br>• Telehealth	*$215,000*<br><br>$215,000 (recurrent)	*45 WAUs (Q22)*<br><br>45 WAUs (Q22)	*2019/20*<br><br>2020/21	Funding may be withdrawn if requirements are not met.<br>Funding is provided for Telehealth (tele-ophthalmology and antenatal telehealth services).
Rapid Results Program<br>*Delivering what matters: Networked Cardiac Services*	*$206,070*<br><br>$408,231 (recurrent)	*18 WAUs (Q22)*<br><br>36 WAUs (Q22)	*2019/20*<br><br>2020/21	Funding is provided for the provision of Networked Cardiac Services.<br>Services will be provided consistent with the agreed business case.<br>If service commencement does not align with the agreed implementation timeframes funding may be withdrawn on a pro-rata basis.<br>If the agreed service levels are not provided, funding may be withdrawn.<br>The HHS will utilise the Queensland Cardiac Outcomes Registry Outreach data module for all data capture and reporting.<br>Activity levels will be monitored quarterly.
Rapid Results Program<br>*Delivering what matters: Advancing Kidney Care 2026 Collaborative*	$565,472 (recurrent)	*62 WAUs (Q22 part WAU backed)*<br><br>117 WAUs (Q22 fully WAU backed)	*2019/20*<br><br>2020/21	Funding is provided for implementation of a vascular access coordination model, supported home haemodialysis model and transplant coordination model under the *Advancing Kidney Care 2026 Collaborative.*<br>The HHS will ensure that the reporting requirements established for this initiative are met, including the provision of quarterly progress against agreed implementation milestones and outcome measures.

Service/Program/Project	Funding	Activity	Timeframe	Conditions
Rapid Results Program<br>*Delivering what matters: Frail and Older persons initiative*<br>• Geriatric Emergency Department Intervention (GEDI)	$200,000 (recurrent)	*0*	*2019/20*	Funding is provided for the establishment of a rural older person's model of care by 1 July 2019.<br>The HHS will:<br>• Establish the service in line with agreed timelines;<br>• Establish a Steering Committee to provide oversight of the service development and operation;<br>• Comply with the agreed reporting requirements, including progress against the identified project outcomes and performance measures; and<br>• Participate in learning sessions and statewide working group meetings.<br>If these conditions are not met or if dedicated Frail Older Persons models of care are ceased, funding may be withdrawn.
Care in the Right Setting (CaRS):<br>• Better Health North Queensland: Regional Healthcare in the Home	*$431,000*	*0*	*2019/20*	Services will be provided consistent with the CaRS application(s).<br>If Service commencement does not align with the agreed implementation timeframes funding may be withdrawn on a pro-rata basis.<br>If the agreed Service levels are not provided, funding may be withdrawn.<br>Activity levels will be monitored regularly and cooperation with external evaluators is required.<br>Where the Service includes Service provision to another (receiving) HHS:<br>• If staffing is not available within the HHS to meet the agreed Service levels, the HHS will make alternate arrangements to ensure that the agreed Service levels are provided; and<br>• If the agreed Service levels are not provided, funding may be withdrawn and provided to the receiving HHS.

Service/Program/Project	Funding	Activity	Timeframe	Conditions
Planned Care Volume Targets – Elective Surgery	$3,699,130 (Funding in existing service agreement)	733 elective surgery separations aligned with the elective surgery data collection, as reported on SPR and any outsourced elective surgery activity. 763 WAUs (Q22).	2020/21	Funding has been quarantined to support the ongoing delivery of planned care volumes during 2020/21. Quarantined funds have been determined based on agreed average Q22 WAUs for 733 Elective Surgery Separations (volume target).<br>Volume targets to be reviewed and adjusted through the amendment window process to include any additional planned care investments.<br>Performance will be monitored quarterly with reconciliation of the agreed planned care activity targets (number of day case and overnight treated patients).<br>Should the agreed volume targets not be met, funding will be withdrawn at the calculated HHS average Q22 WAUs **per separation not delivered**:<br>Example:<br>1 Case = 1.04 Q22 WAUs or<br>$5,045 (QEP)<br>There will not be claw back of under delivery against Q22 WAUs target if the volume of elective day case and overnight separations has been delivered.

Service/Program/Project	Funding	Activity	Timeframe	Conditions
Planned Care Volume Targets – Gastrointestinal Endoscopy (GIE)	$1,302,518 (Funding in existing service agreement value)	478 Gastrointestinal Endoscopies aligned with the Gastrointestinal Endoscopy data collection, as reported on SPR and any outsourced GIE activity. 269 WAUs (Q22).	2020/21	Funding has been quarantined to support the ongoing delivery of planned care volumes during 2020/21. Quarantined funds have been determined based on agreed average Q22 WAUs for 478 Gastrointestinal Endoscopy Separations (volume target).<br>Volume targets to be reviewed and adjusted through the amendment window process to include any additional planned care investments.<br>Performance will be monitored quarterly with reconciliation of the agreed planned care activity targets (number treated GIE patients).<br>Should the agreed volume targets not be met, funding will be withdrawn at the calculated HHS average Q22 WAUs per separation not delivered:<br>Example:<br>1 Case = 0.56 Q22 WAUs or<br>$2,726 (QEP)<br>There will not be claw back of under delivery against Q22 WAUs target if the volume of GIE day case and overnight separations has been delivered.

Service/Program/Project	Funding	Activity	Timeframe	Conditions
Planned Care Volume Targets – Specialist Outpatients	$787,945 (Funding in existing service agreement value)	5,748 Specialist Outpatient initial service events as per the funding specification, and outsourced activity. 163 WAUs (Q22).	2020/21	Funding has been quarantined to support the ongoing delivery of planned care volumes during 2020/21. Quarantined funds have been determined based on agreed average Q22 WAUs for 5,748 Initial Service Events (volume target).<br>Volume targets to be reviewed and adjusted through the amendment window process to include any additional planned care investments.<br>Performance will be monitored quarterly with reconciliation of the agreed planned care activity targets (number of initial service events).<br>Should the agreed volume targets not be met, funding will be withdrawn at the calculated HHS average Q22 WAUs per initial service event not delivered:<br>Example:<br>1 Case = 0.028 Q22 WAUs or<br>$137 (QEP)<br>There will not be claw back of under delivery against Q22 WAUs target if the volume of initial service events has been delivered.
Oral Health Services	$1,679,000 (recurrent)	27,738 WOOS	2020/21	Delivery consistent with the Oral Health Policy Framework.<br>Funding may be adjusted where the total oral health activity delivered varies from the purchased levels.<br>Oral health activity (WOOS) for the 0-15 year age group shall not be less than that achieved in 2017/18.<br>Oral health activity (WOOS) includes activity claimed under the Child Dental Benefits Schedule but excludes dental treatment delivered under general anaesthetic in public hospitals.
• National Partnership Agreement (NPA) on Adult Public Dental Services<br>• National Partnership Agreement (NPA) on Adult Public Dental Services – First Nations	$70,000<br>$88,000	921 WOOS<br>1,158 WOOS	2020/21<br>2020/21	Queensland is required to meet two performance targets during 2020/21, which are the 30 September 2020 target (for 1 April to 30 September 2020) and the 31 March 2021 target (for 1 October 2020 to 31 March 2021). HHSs must collectively meet these targets.<br>Funding may be adjusted where the total oral health activity delivered varies from the purchased levels.

3.2 **National Partnership on COVID-19 Response**

(a) The Department will provide additional funding to the HHS under the State Public Health Payment component of the *National Partnership on COVID-19 Response* where actual additional cost is demonstrated.

(b) Funding will be provided where the HHS has:

(i) undertaken activity that is in-scope for the State Public Health Payment during the reporting period; and

(ii) met the reporting requirements in relation to COVID-19 expenditure, as prescribed under the *National Partnership on COVID-19 Response* and by the Department.

(c) The scope of the State Public Health Payment is defined as:

(i) additional costs that are attributable to the treatment of patients with diagnosed or suspected COVID-19; or

(ii) additional costs of activities directed at preventing the spread of COVID-19.

(d) Additional costs that are reimbursed through the State Public Health Payment will be excluded from the calculation of activity eligible for funding under the terms of the National Health Reform Agreement.

(e) There is no guarantee that the HHS will have access to additional funding under the State Public Health Payment.

(f) All funding that is provided through the State Public Health Payment will be subject to a reconciliation to be undertaken by the Administrator of the National Health Funding Pool. The HHS will be required to provide evidence to support claims for additional costs. If the HHS is unable to provide the required evidence on request, funding received may be recalled subject to reconciliation.

(g) Funding adjustments will be actioned through the process set out in clause 3.4 of Schedule 5 of this Service Agreement.

3.3 **Financial adjustments – other**

(a) The purchasing approach includes a range of funding adjustments which aim to incentivise high value care, that is care which delivers the best outcomes at an efficient cost, and dis-incentivise Low Benefit Care. This includes incentive payments for HHS who achieve quality targets in specific areas of priority. The purchasing incentives that apply to this Service Agreement are detailed in Table 2.

(b) The Department must reconcile the applicable purchasing incentives in Table 2 in line with the timeframes specified in the purchasing specification sheet included within the supporting document 'Purchasing Policy and Funding Guidelines 2020/21'. The Department must promptly provide a copy of the reconciliation statement to the HHS-SA Contact Person.

(c) Funding adjustments must be based on the requirements contained in the relevant specification sheet for that purchasing incentive.

(d) If the Parties are unable to reach agreement in relation to any funding adjustments that are identified, the provisions of clause 14 in the standard terms of this Service Agreement will apply to resolve the dispute.

(e) When the Parties have agreed on a funding adjustment, the Chief Executive (or delegate) will issue an Adjustment Notice to the HHS-SA Contact Person confirming any funding adjustment that has been made in accordance with this clause 3.3 of Schedule 2. The Adjustment Notice will be issued through the process set out in clause 3.4(c) of Schedule 5.

(f) Following receipt of an Adjustment Notice under clause 3.4(c) of Schedule 5, the Parties will comply with the Adjustment Notice and immediately take the steps necessary to give effect to the requirements of that Adjustment Notice.

(g) The Parties acknowledge that the funding adjustments may vary the Service Agreement Value recorded in Schedule 2. Where the Service Agreement Value recorded in Schedule 2 is varied, the variation will be recorded in a Deed of Amendment issued following the next available Amendment Window.

Table 2 Purchasing Incentives 2020/21 (Summary)

Incentive	Description	Scope	Status for 2020/21	Funding Adjustment
Quality Improvement Payment (QIP) – Antenatal Visits for First Nations Women	Incentive payments for achieving targets for:<br>• First Nations women attending an antenatal session during their first trimester, and attending at least 5 antenatal visits; and<br>• First Nations women stopping smoking	All HHSs (excluding Children's Health Queensland)	Continues as per 2019/20 with new targets	50% advance payments made to HHSs with balance paid retrospectively based on performance.
Quality Improvement Payment (QIP) - Smoking Cessation (Community Mental Health)	Incentive payments for achieving targets for community mental health patients clinically supported onto the Smoking Cessation Clinical Pathway	All HHSs (excluding Children's Health Queensland and Mater Public Hospitals)	Continues as per 2019/20 with new targets	Paid retrospectively
High Cost Home Support Program	Payment for high cost 24 hour home ventilated patients meeting the eligibility criteria, where funding is not available through existing sources	All HHSs	Continues as per 2019/20	Paid retrospectively based on forecast costs
Telehealth	Incentive payments for additional outpatient activity volume, provision of telehealth consultancy for Inpatients, Emergency Department and Outpatients episodes and Store and Forward assessments	Inpatients, Emergency Department, Outpatients, and Store and Forward - all HHSs	Continues as per 2019/20 with Outpatients scope expanded to include rural and remote facilities across all HHSs	Paid retrospectively
Sentinel Events	Zero payment for national sentinel events	All ABF public hospitals	Continues as per 2019/20	Retrospective adjustment

3.4 Public and private activity/Own Source Revenue

(a) Own Source Revenue comprises Grants and Contributions, User Charges and Other Revenues.

(b) Where an HHS is above its Own Source Revenue target in respect of private patients, it will be able to retain the additional Own Source Revenue with no compensating adjustments to funding from other sources.

(c) Conversely where an HHS is below its Own Source Revenue target in respect of private patients, it will experience a reduction in revenue with no compensating adjustments to funding from other sources.

(d) The HHS will make all efforts to identify and maximise revenue from appropriate third parties to ensure optimisation of available State and Commonwealth funding across the Queensland Public Sector Health System.

(e) The Own Source Revenue identified in Table 3 is an estimate generated by the HHS which allows all third-party funding sources associated with service delivery

to be identified. The HHS will ensure that this estimate is substantiated and accurate to ensure no significant variances to actual revenue generated.

(f) The HHS will routinely revise and update the estimate to ensure alignment between the Service Agreement and Queensland Treasury's reporting system (TRIDATA).

(g) Budget adjustments for changes in Own Source Revenue from private patients will be actioned through the process set out in Schedule 5 of this Service Agreement.

4. Funding sources

4.1 The four main funding sources contributing to the HHS Service Agreement value are:

(a) Commonwealth funding;

(b) State funding;

(c) Grants and Contributions; and

(d) Own Source Revenue.

4.2 Table 3 provides a summary of the funding sources for the HHS and mirrors the total value of the Service Agreement included in Table 4.

Table 3 Hospital and Health Service funding sources 2020/21

Funding Source	Value ($)
**NHRA Funding**	
Activity Based Funding	93,011,898
Clinical Education and Training[3]	-4,090,477
Own Source Revenue contribution in ABF funded services	-1,684,841
**Pool Account – ABF Funding (State and Commonwealth)[4]**	**87,236,580**
Block Funding	47,141,930
Clinical Education and Training[3]	4,090,477
**State Managed Fund – Block Funding (State and Commonwealth)[5]**	**51,232,407**
**Locally Receipted Funds (Including Grants)**	**4,282,792**
**Locally Receipted Own Source Revenue (ABF)**	**1,684,841**
**Locally Receipted Own Source Revenue (Other activities)**	**4,558,538**
**Department of Health Funding[6]**	**45,654,290**
**Total NHRA Funding**	**194,649,448**
**NPA Covid-19 Response**	
Activity Based Funding	-
**Hospital Services Payment – ABF Funding (State and Commonwealth)[7]**	**-**
Block Funding	-
Clinical Education and Training[3]	-
**Hospital Services Payment – Block Funding (State and Commonwealth)[5]**	**-**
**Public Health Funding (State and Commonwealth) [8]**	**3,100,000**
**Total NPA – COVID-19 Funding**	**3,100,000**
**TOTAL**	**197,749,448**

[3] Clinical Education and Training (CET) is classified as Teaching, Training and Research Funding under the National Model and funded as a Block Funded Service. Under the State Model, CET is included as 'Other ABF' and forms part of the ABF total. To comply with the requirements of the National Health Reform Agreement, funding must be paid as it is received, therefore from a Funding Source perspective, CET has been reclassified to Block Funding.

[4] Pool Account - ABF Funding (State and Commonwealth) includes: Inpatient; Critical Care; Emergency Department; Sub and Non Acute; Mental Health; and Outpatient activities each allocated a proportion of Other ABF Adjustments.

[5] State Managed Fund - Block Funding (State and Commonwealth) includes: block funded hospitals; standalone specialist mental health hospitals; community mental health; and teaching, training and research.

[6] Department of Health Funding represents funding by the Department for items not covered by the National Health Reform Agreement including such items as: Prevention, Promotion and Protection; Depreciation, and other Health Services.

7 Hospital Services Payment - Funding provided under the COVID-19 National Partnership Agreement for activity that is attributable to the diagnosis and treatment of Medicare eligible patients with COVID-19 or suspected of having COVID-19; elective surgeries that have been rescheduled to free up public hospital capacity to respond to the COVID-19 outbreak; and may include activities related to the care of public patients being treated in private hospitals.

[8] Public Health Payment - Funding provided under the COVID-19 National Partnership Agreement for the State public health system's activity attributable to the treatment of patients with diagnosed or suspected COVID-19 or for activities directed at preventing the spread of COVID-19.

5. Funds disbursement

5.1 The Chief Executive will direct the disbursement of both State and Commonwealth funding from the State's National Health Funding Pool Sub Account and the State Managed Fund to the HHS. The Service Agreement and State level block payments to State managed funds from Commonwealth payments into the national funding pool are stated in Table 8.

5.2 However, the State (represented by the Chief Executive) will not:

(a) redirect Commonwealth payments between HHSs;

(b) redirect Commonwealth payments between funding streams (e.g. from ABF to Block Funding); and/or

(c) adjust the payment calculations underpinning the Commonwealth's funding.

5.3 Payment of Activity Based Funding and Block Funding to the HHS will be on a fortnightly basis.

5.4 Further information on the disbursement of funds is available in the supporting document to this Service Agreement 'Purchasing Policy and Funding Guidelines 2020/21'.

Table 4 HHS Finance and Activity Schedule 2019/20 – 2021/22 – Summary by Purchasing Hierarchy

	ABF/NABF	ABF_SPLIT	Service Stream	2019/20 QWAU (Q22)	2019/20 Funding ($) (Price: $4847)	2020/21 QWAU (Q22)	2020/21 Funding) (Price: $4847)	2021/22 QWAU (QTBC)	2021/22 Funding (Price: $TBC)
**Allocations excluding COVID-19**	**ABF**	ABF	Inpatients	8,962	$41,899,280	9,182	$40,971,810		
			Outpatients	2,389	$11,363,021	2,496	$10,538,444		
			Procedures & Interventions	1,009	$4,752,477	1,029	$4,243,364		
			Emergency Department	3,995	$19,118,606	4,082	$18,592,269		
			Sub & Non-Acute	728	$3,469,936	744	$3,365,039		
			Mental Health	116	$556,135	118	$548,786		
			Prevention & Primary Care	358	$1,735,406	344	$1,837,000		
			Other ABF $	0	$7,404,498	0	$7,319,712		
		**ABF Total**		**17,556**	**$90,299,359**	**17,994**	**$87,416,424**		
		ABF Other	CET Funding	0	$4,097,365	0	$4,931,531		
			Specified Grants	0	$595,129	0	$663,943		
			PPP	0	$0	0	$0		
			EB Quarantined	0	$822,859	0	$0		
		**ABF Other Total**		**0**	**$5,515,353**	**0**	**$5,595,474**		
	**Other Funding**	Other Funding	Block Funded Services	3,753	$45,853,171	3,753	$45,824,644		
			Population Based Community Services	0	$19,688,360	0	$19,437,828		
			Other Specific Funding	0	$36,502,695	0	$36,344,504		
			PY Services moved to ABF	0	$0	0	$0		
			Prevention Services – Public Health	0	$100,000	0	$30,574		
		**Other Funding Total**		**3,753**	**$102,144,225**	**3,753**	**$101,637,550**		
	**Allocations excluding COVID-19 TOTAL**			**21,308**	**$197,958,937**	**21,747**	**$194,649,448**		

	ABF/NABF	ABF_SPLIT	Service Stream	2019/20 QWAU (Q22)	2019/20 Funding ($) (Price: $4847)	2020/21 QWAU (Q22)	2020/21 Funding) (Price: $4847)	2021/22 QWAU (QTBC)	2021/22 Funding (Price: $TBC)
**COVID-19 related allocations**	**ABF**	ABF	Inpatients	0	$0	0	$0		
			Outpatients	0	$0	0	$0		
			Procedures & Interventions	0	$0	0	$0		
			Emergency Department	0	$0	0	$0		
			Sub & Non-Acute	0	$0	0	$0		
			Mental Health	0	$0	0	$0		
			Prevention & Primary Care	0	$0	0	$0		
			Other ABF $	0	$0	0	$0		
		**ABF Total**		**0**	**$0**	**0**	**$0**		
		ABF Other	CET Funding	0	$0	0	$0		
			Specified Grants	0	$0	0	$0		
			PPP	0	$0	0	$0		
			EB Quarantined	0	$0	0	$0		
		**ABF Other Total**		**0**	**$0**	**0**	**$0**		
	**Other Funding**	Other Funding	Block Funded Services	0	$0	0	$0		
			Population Based Community Services	0	$0	0	$0		
			Other Specific Funding	0	$1,890,683	0	$3,100,000		
			PY Services moved to ABF	0	$0	0	$0		
			Prevention Services – Public Health	0	$0	0	$0		
		**Other Funding Total**		**0**	**$1,890,683**	**0**	**$3,100,000**		
	**COVID-19 Allocations TOTAL**			**0**	**$1,890,683**	**0**	**$3,100,000**		
**Grand Total**				**21,308**	**$199,849,620**	**21,747**	**$197,749,448**		

Table 5 Minor Capital and Equity

	2019/20 $	2020/21 $	2021/22 $
**Minor Capital & Equity**			
**Cash**			
SA 16-17.329 - Minor Capital funding Allocation 2016-17	$869,000	$869,000	
NTW-EoY1718-03 Indigenous Family Room Funding Swap (Capex)	$0	$0	
NTW-AW3-Feb19-03 Making Tracks – Mornington Island initiative	$0	$0	
NTW-AW3-FEB20-06 Lease funding swap per changes to AASB16 - equity component	$280,014	$0	
NTW-BB2021-28 Lease funding swap per changes to AASB16 - equity component	$0	$279,000	
**Non-Cash**			
-	-	-	
**Grand Total**	**$1,149,014**	**$1,148,000**	

Table 6 HHS Finance and Activity Schedule 2019/20 – 2021/22 Other Funding Detail

Agreement		Service Stream	Category	2019/20 $	2020/21 $	2021/22 $
Allocations excluding COVID-19	Other Funding	Block Funded Services	Block Funded Services	$45,853,171	$45,824,644	
		**Block Funded Services Total**		**$45,853,171**	**$45,824,644**	
		Population Based Community Services	Alcohol, Tobacco & Other Drugs	$3,711,844	$3,849,267	
			Community Care Programs	$0	$0	
			Community Mental Health	$2,766,816	$2,869,063	
			Community Mental Health – Child & Youth	$489,030	$357,746	
			Other Community Services	$8,224,897	$8,258,187	
			Other Funding Subsidy/(Contribution)	-$3,288,687	-$3,288,687	
			Primary Health Care	$7,784,459	$7,392,254	
		**Population Based Community Services Total**		**$19,688,360**	**$19,437,828**	
		Other Specific Funding	Aged Care Assessment Program	$217,160	$162,870	
			Commercial Activities	$188,169	$188,169	
			Consumer Information Services	$0	$0	
			Depreciation	$8,854,000	$9,049,000	
			Disability Residential Care Services	$0	$0	
			Environmental Health	$780,050	$808,929	
			Home & Community Care (HACC) Program	$0	$0	
			Home & Community Medical Aids & Appliances	$4,037	$4,037	
			Home Care Packages	$39,305	$39,305	
			Interstate Patients	$184,039	$184,039	
			Multi-Purpose Health Services	$1,198,544	$1,198,544	
			Prisoner Health Services	$0	$0	
			Oral Health	$0	$0	
			Patient Transport	$12,828,842	$12,828,842	
			Research	$0	$0	
			Residential Aged Care	$0	$0	
			Specific Allocations	$12,203,775	$11,875,993	
			State-Wide Functions	$0	$0	
			Transition Care	$4,774	$4,774	
		**Other Specific Funding Total**		**$36,502,695**	**$36,344,504**	
		Prevention Services – Public Health	Environmental Health (PH)	$0	$0	
			Other Community Services (PH)	$100,000	$30,574	

Agreement		Service Stream	Category	2019/20 $	2020/21 $	2021/22 $
		**Prevention Services – Public Health Total**		**$100,000**	**$30,574**	
	**Allocations excluding COVID-19 Other Funding Total**			**$102,144,225**	**$101,637,550**	
**COVID-19 related allocations**	**Other Funding**	Block Funded Services	Block Funded Services	$0	$0	
		**Block Funded Services Total**		**$0**	**$0**	
		Population Based Community Services	Alcohol, Tobacco & Other Drugs	$0	$0	
			Community Care Programs	$0	$0	
			Community Mental Health	$0	$0	
			Community Mental Health – Child & Youth	$0	$0	
			Other Community Services	$0	$0	
			Other Funding Subsidy/(Contribution)	$0	$0	
			Primary Health Care	$0	$0	
		**Population Based Community Services Total**		**$0**	**$0**	
		Other Specific Funding	Aged Care Assessment Program	$0	$0	
			Commercial Activities	$0	$0	
			Consumer Information Services	$0	$0	
			Depreciation	$0	$0	
			Disability Residential Care Services	$0	$0	
			Environmental Health	$0	$0	
			Home & Community Care (HACC) Program	$0	$0	
			Home & Community Medical Aids & Appliances	$0	$0	
			Home Care Packages	$0	$0	
			Interstate Patients	$0	$0	
			Multi-Purpose Health Services	$0	$0	
			Prisoner Health Services	$0	$0	
			Oral Health	$0	$0	
			Patient Transport	$0	$0	
			Research	$0	$0	
			Residential Aged Care	$0	$0	
			Specific Allocations	$1,890,683	$3,100,000	
			State-Wide Functions	$0	$0	
			Transition Care	$0	$0	
		**Other Specific Funding Total**		**$1,890,683**	**$3,100,000**	
			Environmental Health (PH)	$0	$0	

Agreement		Service Stream	Category	2019/20 $	2020/21 $	2021/22 $
		Prevention Services – Public Health	Other Community Services (PH)	$0	$0	
		**Prevention Services – Public Health Total**		**$0**	**$0**	
	**COVID-19 Allocations Other Funding Total**			**$1,890,683**	**$3,100,000**	
**Grand Total**				**$104,034,909**	**$104,737,550**	

Table 7 Specified Grants

Program	2019/20 $	2020/21 $	2021/22 $
High Cost Outliers	$399,470	$402,731	
Limited Indication Medication Scheme	$76,246	$76,868	
18-19 Purch Initiatives (Final reconciliation) - Rewards	$119,413	$0	
20-21 QIP - Antenatal care for Indigenous women	$0	$184,344	
**Grand Total**	**$595,129**	**$663,943**	

Table 8 Hospital and Health Service Service Agreement and State Level Block Payments to state managed funds from Commonwealth payments into national funding pool

**State:**	QLD	**Service agreement for financial year:**	2020/21
**HHS**	North West	**Version for financial year:**	
**HHS ID**		**Version effective for payments from:**	
		**Version status:**	07.07.2020

HHS ABF payment requirements:

**Expected National Weighted Activity Unit (NWAU)**		**National efficient price (NEP) (as set by IHPA)**
**ABF Service group**	**Projected NWAU – N2021 (Draft)**	
Admitted acute public services	9,743	$5,320
Admitted acute private services	85	$5,320
Emergency department services	4,202	$5,320
Non-admitted services	1,849	$5,320
Mental health services	143	$5,320
Sub-acute services	730	$5,320
**LHN ABF Total – excluding COVID-19**	**16,753**	**$5,320**
**LHN ABF Total – COVID-19 NPA**	**0**	**$5,320**

Note:

NWAU estimates do not take account of cross-border activity

Reporting requirements by HHS - total block funding paid (including Commonwealth) per HHS, as set out in Service Agreement:

**Amount (Commonwealth and State) for each amount of block funding from state managed fund to LHN:**	
**Block funding component**	**Estimated Commonwealth and state block funding contribution (ex GST)**
Block funded hospitals	$43,943,670
Community mental health services	$3,198,260
Teaching, Training and Research	$4,090,477
Home ventilation	$0
Other block funded services	$0
**Total block funding for LHN – excluding COVID-19**	**$51,232,407**
**Total funding for LHN under COVID-19 NPA**<br>**State Public Health Payment**	**$3,100,000**

6. Purchased services

6.1 State-funded Outreach Services

(a) The HHS forms part of a referral network with other HHSs. Where state-funded Outreach Services are currently provided the HHS will deliver these Health Services in line with the following principles:

(i) historical agreements for the provision of Outreach Services will continue as agreed between HHSs;

(ii) funding will remain part of the providing HHS's funding base;

(iii) activity should be recorded at the HHS where the Health Service is being provided; and

(iv) the Department will purchase outreach activity based on the utilisation of the Activity Based Funding (ABF) price when Outreach Services are delivered in an ABF facility.

(b) Where new or expanded state-funded Outreach Services are developed the following principles will apply:

(i) the Department will purchase outreach activity based on the utilisation of the ABF price when Outreach Services are delivered in an ABF facility;

(ii) agreements between HHSs to purchase Outreach Services will be based on a cost recovery model, which will ensure providing sites are not financially disadvantaged and annual increases will be consistent with the ABF model;

(iii) any proposed expansion or commencement of Outreach Services will be negotiated between HHSs;

(iv) the HHS is able to purchase the Outreach Service from the most appropriate provider including private providers or other HHSs. However, when a change to existing Health Services is proposed, a transition period of at least 12 months will apply during which time the HHS will be required to continue to purchase Outreach Services from the HHS currently providing the Health Service;

(v) any changes to existing levels of Outreach Services need to be agreed to by both HHSs and any proposed realignment of funding should be communicated to the Department to ensure that any necessary funding changes are actioned as part of the Service Agreement amendment process and/or the annual negotiation of the Service Agreement Value; and

(vi) the activity should be recorded at the HHS where the Health Service is being provided.

(c) In the event of a disagreement regarding the continued provision of state-funded Outreach Services:

(i) any proposed cessation of Outreach Services will be negotiated between HHSs to mitigate any potential disadvantage or risks to either HHS; and

(ii) redistribution of funding will be agreed between the HHSs and communicated to the Department to action through the Service Agreement amendment processes outlined in Schedule 5 of this Service Agreement.

6.2 **Telehealth services**

(a) The HHS will support implementation of the Department Telehealth program, including the Telehealth Emergency Support Service. The HHS will collaborate with the Department, other HHSs, relevant non-government organisations and Primary Care stakeholders to contribute to an expanded network of Telehealth services to better enable a program of scheduled and unscheduled care.

(b) The HHS will ensure dedicated Telehealth Coordinators progress the Telehealth agenda locally, driving stakeholder engagement, adoption, planning and implementation activities that will support and grow Telehealth enabled services through substitution of existing face to face services and identification of new Telehealth enabled models of care.

(c) The HHS will ensure the Medical Telehealth Lead will collaborate with the network of HHS based Telehealth Coordinators and the Telehealth Support Unit to assist in driving promotion and adoption of Telehealth across the State through intra and cross-HHS clinician led engagement and change management initiatives as well as informing the development and implementation of clinical protocols and new Telehealth enabled models of care.

6.3 **Newborn hearing screening**

(a) In line with the National Framework for Neonatal Hearing Screening the HHS will:

(i) provide newborn hearing screening in all birthing hospitals and screening facilities; and

(ii) provide where applicable, co-ordination, diagnostic audiology, family support, and childhood hearing clinic services which meet the existing screening, audiology and medical protocols available from the Healthy Hearing website.

6.4 **Statewide Services**

This clause does not apply to this HHS.

6.5 **Statewide and highly specialised clinical services**

The HHS will:

(a) participate in and contribute to the staged review of the purchasing model for identified Statewide and highly specialised clinical services; and

(b) collaborate with the Department and other HHSs in the development of Statewide Services Descriptions through the implementation of the Statewide Services Governance and Risk Management Framework. The Statewide Services Governance and Risk Management Framework guides the Department and HHSs in the strategic management, oversight and delivery of Statewide Services in order to optimise clinical safety and quality and ensure sustainability of services across Queensland.

6.6 **Regional Services**

This clause does not apply to this HHS.

6.7 **Rural and remote clinical support**

This clause does not apply to this HHS.

6.8 **Prevention Services, Primary Care and Community Health Services**

(a) The following funding arrangements will apply to the Prevention, Primary Care and Community Health Services delivered by the HHS:

(i) Department funding for Community Health Services. A pool of funding for these services is allocated to each HHS for a range of Community Health Services and must be used to meet local Primary Care and community healthcare and prevention needs including through delivery of the services identified in Table 6 and HHSs have the discretion to allocate funding across Primary Care and Community Health Services and Prevention Services according to local priorities.

(ii) Department specified funding models for consumer information services, disability, residential care, environmental health, prisoner health services, home and community medical aids, Primary Care, community mental health services, and alcohol and other drugs services. The funding specified for these programs is listed in Table 6 and Department Community Health Service grants.

(iii) Funding from other state government departments and the Commonwealth for specific programs (third party funded services).

(b) **Prevention Services**

The HHS will provide Prevention Services in line with public health related legislation and the service delivery and reporting requirements outlined in the *Public Health Practice Manual*, including:

(i) **Specialist Public Health Units**

The HHS will:

(A) maintain and improve, using a public health approach, the surveillance, prevention and control of notifiable conditions, including the prevention and control of invasive and exotic mosquitos, in accordance with national and/or State guidelines and ensure clinical and provisional notification of specified notifiable conditions are reported in accordance with the *Public Health Act* and Public Health Regulations;

(B) provide office accommodation and related support to those staff of the Townsville Public Health Unit (PHU) located at Mount Isa. The Townsville PHU will provide specialist communicable disease epidemiology and surveillance, disease prevention and control and environmental health services to North West HHS. Where this includes visits to Mount Isa by Townsville based PHU staff, office accommodation and related support will be made

available for those staff during their visit.

(ii) **Preventive health services**

The HHS will:

(A) maintain delivery of risk factor prevention and early intervention programs and services targeting nutrition, physical activity, alcohol consumption, tobacco use, overweight and obesity and falls prevention;

(B) maintain delivery of the school-based youth nursing program throughout Queensland secondary schools; and

(C) promote brief interventions, lifestyle modification programs and other prevention, promotion or early intervention activities.

(iii) **Immunisation services**

The HHS will maintain or improve existing immunisation coverage through continuation of current immunisation services including:

(A) national immunisation program;

(B) opportunistic immunisation in healthcare facilities;

(C) special immunisation programs; and

(D) delivery of the annual school immunisation program in accordance with the Guideline for Immunisation Services (QH-GDL-955:2014).

(iv) **Sexually transmissible infections including HIV and viral hepatitis**

The HHS will maintain and improve, using a public health approach, the prevention, testing, treatment and contact tracing of blood borne viruses and sexually transmissible infections with a continued focus on relevant identified target populations such as First Nations people and culturally and linguistically diverse populations through Services including, but not limited to:

(A) public health units:

(B) sexual health services;

(C) infectious diseases services;

(D) viral hepatitis services;

(E) syphilis surveillance services;

(F) needle and syringe programs; and

(G) existing clinical outreach and support programs in place between HHSs.

(v) **Tuberculosis services**

The HHS will ensure there is no financial barrier for any person to tuberculosis diagnostic and management services and will ensure that

services are available in accordance with the Tuberculosis Control health service directive (qh-hsd-040:2018) and Protocol (qh-hsdptl-040-1:2018).

(vi) **Public Health Events of State Significance**

The HHS will comply with the *Declaration and Management of a Public Health Event of State Significance* health service directive (qh-hsd-046:2014).

(vii) **Cancer screening services**

The HHS will:

(A) increase cervical screening rates for women in rural and remote areas and refer clients to relevant preventive health programs such as BreastScreen Queensland, QUIT line, Get Healthy and My Health for Life by maintaining the existing Mobile Women's Health Service;

(B) provide the Department with an annual report detailing the services provided by the Mobile Women's Health Service;

(C) ensure that all cervical screening services provided by the HHS are delivered in accordance with the National Competencies for Cervical Screening Providers and national cervical screening policy documents. Services to be provided:

- across North West HHS;

(D) maintain the existing Healthy Women's Initiative in accordance with the Principles of Practice, Standards and Guidelines for Providers of Cervical Screening Services for First Nations women and national cervical screening policy documents;

(E) provide timely, appropriate, high quality and safe follow-up diagnostic services within the HHS for National Cervical Screening Program participants in accordance with the *National Cervical Screening Program Guidelines for the Management of Screen-detected Abnormalities, Screening in Specific Populations and Investigation of Abnormal Vaginal Bleeding* (2017) and national cervical screening policy documents; and

(F) provide timely, appropriate, high quality and safe diagnostic assessment services for National Bowel Cancer Screening Program participants in accordance with the National Health and Medical Research Council's *Clinical Guidelines for Prevention, Early Detection and Management of Colorectal Cancer* (2017).

6.9 **Oral health services**

The HHS will ensure that:

(a) oral health services are provided to the Eligible Population at no cost to the patient[9] and that the current range of clinical services will continue;

(b) oral health services fulfil the relevant obligations related to Commonwealth Government dental funding program/s;

(c) service delivery is consistent with Queensland Health's oral health policy framework; and

(d) the repair maintenance and relocation service for the mobile dental fleet continues to be provided by the Mobile Dental Clinic Workshop in Metro South HHS.

6.10 **Prisoner health services**

This clause does not apply to this HHS.

6.11 **Refugee health**

This clause does not apply to this HHS.

6.12 **Adult sexual health clinical forensic examinations**

(a) The HHS will:

(i) provide 24-hour access to clinical forensic examinations for adult victims of sexual assault who present at a public hospital; and

(ii) provide the Department with a quarterly report on the number of examinations provided.

(b) The Service provided will be consistent with the principles of the Queensland Government inter-agency guidelines for responding to people who have experienced sexual assault and any standards issued pursuant to a Health Service Directive.

7. Teaching training and research

The HHS will provide the teaching, training and research programs for which funding is provided within Schedule 2 of this Service Agreement and as described below:

7.1 **Clinical education and training**

(a) The HHS will:

(i) continue to support and align with the current Student Placement Deed Framework which governs clinical placements from relevant tertiary education providers in Queensland HHS facilities;

(ii) comply with the obligations and responsibilities of Queensland Health under the Student Placement Deed, as appropriate, as operator of the facility at which the student placement is taking place;

(iii) comply with the terms and conditions of students from Australian

[9] The HHS may provide oral health services on a fee-for-service basis to non-eligible patients in rural and remote areas where private dental services are not available.

education providers participating in the Student Placement Deed Framework;

(iv) only accept clinical placements of students from Australian education provides participating in the Student Placement Deed Framework;

(v) continue to provide training placements consistent with and proportionate to the capacity of the HHS. This includes, but is not limited to, planning and resourcing for clinical placement offers in collaboration with other HHSs and the Department, and the provision of placements for the following professional groups relevant to the HHS:

(A) medical students

(B) nursing and midwifery students

(C) pre-entry clinical allied health students

(D) interns

(E) rural generalist trainees

(F) vocational medical trainees

(G) first year nurses and midwives

(H) re-entry to professional register nursing and midwifery candidates

(I) dental students

(J) allied health rural generalist training positions

(K) Aboriginal and Torres Strait Islander Health Workers and Aboriginal and Torres Strait Islander Health Practitioners

(vi) participate in vocational medical rotational training schemes, facilitate the movement of vocational trainees between HHSs and work collaboratively across HHSs to support education and training program outcomes;

(vii) report, at the intervals and in the format agreed between the Parties, to the Department on the pre-entry clinical placements provided under the Student Placement Deed Framework;

(viii) comply with the state-wide vocational medical training pathway models including:

(A) The Queensland Basic Physician Training Network;

(B) The Queensland General Medicine Advanced Training Network;

(C) The Queensland Intensive Care Training Pathway;

(D) The Queensland Basic Paediatric Training Network;

(E) The Queensland General Paediatric Advanced Training Network; and

(F) The Queensland Neonatal and Perinatal Medicine Advanced Training Network;

(ix) support the provision of placements by the Queensland Physiotherapy Placement Collaborative for physiotherapy pre-entry students via the Physiotherapy Pre-registration Clinical Placement Agreement;

(x) provide clinical area placements for dietetics pre-entry students from additional funding provided through relevant agreements with Universities;

(xi) participate in the Voluntary Oral Health Therapist Graduate Scheme; and

(xii) participate in the Voluntary Dental Graduate Scheme.

(b) In addition, the Health Practitioners and Dental Officers (Queensland Health) Certified Agreement (No 2) 2016 (the HP agreement) requires Hospital and Health Services to:

(i) continue to support development of allied health research capacity through continued implementation and retention of health practitioner research positions provided through the HP agreement; and

(ii) support development of allied health clinical education capacity through continued implementation and retention of clinical educator positions provided through the HP agreement, continuing to provide allied health pre-entry clinical placements and maintaining support for allied health HP 3 to 4 rural development pathway positions.

7.2 **Health and medical research**

The HHS will:

(a) Articulate an investment strategy for research (including research targets and Performance Measures) which integrates with the clinical environment to improve clinical outcomes;

(b) Develop mechanisms for completing administrative governance for the approval of research in line with a national benchmark of 25 days (Standard Operating Procedures for Queensland Health Research Governance Officers 2013);

(c) Develop mechanisms for monitoring site research activity in line with jurisdictional commitments and National Health and Medical Research Council Guidelines (*Framework for* Monitoring *Guidance for the national approach to single ethical review of multi-centre research, January 2012*); and

(d) Develop systems to capture research and development expenditure and revenue data and associated information on research.

Schedule 3 Performance Measures

1. Purpose

This Schedule 3 outlines the Performance Measures that apply to the HHS.

2. Performance Measures

2.1 The Performance and Accountability Framework uses Performance Measures to monitor the extent to which the HHS is delivering the high-level objectives set out in this Service Agreement.

2.2 Each Performance Measure is identified under one of four categories:

(a) Safety and Quality Markers which together provide timely and transparent information on the safety and quality of services provided by the HHS;

(b) Key Performance Indicators (KPIs) which are focused on the delivery of key strategic objectives and statewide targets. KPI performance will inform HHS performance assessments;

(c) Outcome Indicators which provide information on specific activities and interventions where there is a reasonable expectation or evidence that they make a positive contribution towards improving the health status and experience of patients; and

(d) supporting indicators which provide contextual information and enable an improved understanding of performance, facilitate benchmarking of performance across HHSs and provide intelligence on potential future areas of focus. Supporting indicators are available on-line as referenced in Appendix 1 to this Service Agreement.

2.3 The HHS should refer to the relevant attribute sheet for each Performance Measure for full details. These are available on-line as referenced in Appendix 1 to this Service Agreement.

2.4 The Performance Measures identified in Table 9; Table 10 and Table 11 are applicable to the HHS unless otherwise specified within the attribute sheet.

2.5 The HHS will meet the target for each KPI identified in Table 9 as specified in the attribute sheet.

2.6 The Performance Measures identified in italic text are for future development.

2.7 Further information on the performance assessment process is provided in the supporting document to this Service Agreement, Performance and Accountability Framework 2020/21 referenced at Appendix 1 to this Service Agreement.

Table 9 HHS Performance Measures – Key Performance Indicators

Key Performance Indicators	
**Safe**	
The health and welfare of service users is paramount	
• Minimise risk	• Avoid harm from care
• Transparency and openness	• Learn from mistakes
**Title**	
Hospital Acquired Complications	
**Timely**	
Care is provided within an appropriate timeframe	
• Treatment within clinically recommended time	
**Title**	
Telehealth utilisation rates:<br>• Number of non-admitted telehealth service events	
Access to oral health services:<br>• % of patients on the general care dental wait list waiting for less than the clinically recommended time	
**Equitable**	
Consumers have access to healthcare that is responsive to need and addresses health inequalities	
• Fair access based on need	• Addresses inequalities
Potentially Preventable Hospitalisations – First Nations People	
Low birthweight:<br>• % of low birthweight babies born to Queensland mothers	
**Efficient**	
Available resources are maximised to deliver sustainable, high quality healthcare	
• Avoid waste	• Minimise financial risk
• Sustainable/productive	• Maximise available resources
**Title**	
Forecast operating position:<br>• Full year<br>• Year to date	
Average sustainable Queensland Health FTE	
Capital expenditure performance	
Increase participation in Practice Incentive Program	
**Patient Centred**	
Providing Healthcare that is respectful of and responsive to individual patient preferences, needs and values	
• Patient involved in care	• Patient feedback
• Respects patient/person values and preferences	• Care close to home
**Title**	
Proportion of mental health service episodes with a documented care plan	
Travel rates for admitted patients – reduction target	

Effective	
Healthcare that delivers the best achievable outcomes through evidence-based practice	
• Evidence based practice	• Care integration
• Treatment directed to those who benefit	• Optimise Health
• Clinical Capability	
**Title**	
Potentially Preventable Hospitalisations – non-diabetes complications:<br>• The number and proportion of hospitalisations of people with non-diabetes complications that could have potentially been prevented through the provision of appropriate non-hospital health services	
% of oral health activity which is preventive	
Diabetes control:<br>• *Type 2 diabetes patients with HbA1c result within a specified level*	
Smoking Status:<br>• *% of patients with a respiratory diagnosis whose smoking status has been recorded*	
**People and Culture**	
**Title**	
Workforce:<br>• *Specific measure/s to be determined*	

Table 10 HHS Performance Measures - Safety and Quality Markers

Safety and Quality Markers	
**Safe**	
The health and welfare of service users is paramount	
• Minimise risk<br>• Transparency and openness	• Avoid harm from care<br>• Learn from mistakes
**Title**	
Sentinel Events:<br>• Number of wholly preventable sentinel events	
Hospital Standardised Mortality Ratio	
Severity Assessment Code (SAC) closure rates:<br>• % of incidents closed within the prescribed timeframe	
Transfer of care for mental health patients:<br>• *Time from arrival in the Emergency Department to admission to an acute mental health bed*	

Table 11 HHS Performance Measures – Outcome Indicators

Outcome Indicators	
**Timely**	
Care is provided within an appropriate timeframe	
• Treatment within clinically recommended time	
**Title**	
Telehealth:<br>• *Expansion to rural and remote communities*	
Access to emergency dental care:<br>• % of emergency courses of care for adult dental patients that commence within the recommended waiting times	
**Equitable**	
Consumers have access to healthcare that is responsive to need and addresses health inequalities	
• Fair access based on need	• Addresses inequalities
**Title**	
First Nations people representation in the workforce:<br>• % of the workforce who identify as being First Nations people	
Completed general courses of oral health care for First Nations people adult patients	
**Patient Centred**	
Providing Healthcare that is respectful of and responsive to individual patient preferences, needs and values	
• Patient involved in care	• Patient feedback
• Respects patient/person values and preferences	• Care close to home
**Title**	
Complaints resolved within 35 calendar days	
Advance care planning:<br>• The proportion of approaches made to people who are identified as being at risk of dying within the next 12 months, or suitable for an advance care planning discussion, and who are offered the opportunity to consider, discuss and decide their preferences for care at the end of life	
Outpatient appointments delivered outside the HHS area	
Integrated care pathway in place for patients with identified co-morbidities	
**Effective**	
Healthcare that delivers the best achievable outcomes through evidence-based practice	
• Evidence based practice	• Care integration
• Treatment directed to those who benefit	• Optimise Health
• Clinical Capability	
**Title**	
Uptake of the smoking cessation clinical pathway for public hospital inpatients and dental clients	
Adolescent vaccinations administered via the statewide School Immunisation Program	

Schedule 4 Data Supply Requirements

1. Purpose

1.1 *The Hospital and Health Boards Act 2011*[10] (s.16(1)(d)) provides that the Service Agreement will state the performance data and other data to be provided by an HHS to the Chief Executive, including how, and how often, the data is to be provided.

1.2 This Schedule 4 specifies the data to be provided by the HHS to the Chief Executive and the requirements for the provision of the data.

2. Principles

2.1 The following principles guide the collection, storage, transfer and disposal of data:

(a) trustworthy – data is accurate, relevant, timely, available and secure;

(b) private – personal information is protected in accordance with the law;

(c) valued – data is a core strategic asset;

(d) managed – collection of data is actively planned, managed and compliant; and

(e) quality – data provided is complete, consistent, undergoes regular validation and is of sufficient quality to enable the purposes outlined in clause 3.2 of this Schedule 4 to be fulfilled.

2.2 The Parties agree to constructively review the data supply requirements as set out in this Schedule 4 on an ongoing basis in order to:

(a) ensure data supply requirements are able to be fulfilled; and

(b) minimise regulatory burden.

3. Roles and responsibilities

3.1 **Hospital and Health Services**

(a) The HHS will:

(i) provide, including the form and manner and at the times specified, the data specified in the data supply requirements (Attachment A to this Schedule 4) in accordance with this Schedule 4;

(ii) provide data in accordance with the provisions of the *Hospital and Health Boards Act 2011, Public Health Act 2005* and *Private Health Facilities Act 1999;*

---

[10] Section 143(2)(a) of the *Hospital and Health Boards Act 2011* provides that the disclosure of confidential information (as defined in s.139 of the Act) to the Chief Executive by an HHS under a service agreement is a disclosure permitted by an Act.

(iii) provide other HHSs with routine access to data, that is not Patient Identifiable Data, for the purposes of benchmarking and performance improvement;

(iv) provide data as required to facilitate reporting against the Performance Measures set out in Schedule 3 of this Service Agreement;

(v) provide data as specified within the provision of a health service directive;

(vi) provide activity data that complies with the national data provision timeframes required under the Independent Hospital Pricing Authority (IHPA) data plan for Commonwealth funding. Details of the timeframes are specified in the 'Commonwealth Efficient Growth Funding and National Weighted Activity Units (NWAUs)' specification sheet included in the supporting document Purchasing Policy and Funding Guidelines 2020/21 and the clinical placement data supply requirements; and

(vii) as requested by the Chief Executive from time to time, provide to the Chief Executive data, whether or not specified in this Schedule 4 or the Service Agreement, as specified by the Chief Executive in writing to the HHS in the form and manner and at the times specified by the Chief Executive.

(b) Data that is capable of identifying patients will only be disclosed as permitted by, and in accordance with, the *Hospital and Health Boards Act 2011, Public Health Act 2005 and the Private Health Facilities Act 1999.*

3.2 **Department**

The Department will:

(a) produce a monthly performance report which includes:

(i) actual activity compared with purchased activity levels;

(ii) any variance(s) from purchased activity;

(iii) performance information as required by the Department to demonstrate HHS performance against the Performance Measures specified in Schedule 3 of this Service Agreement; and

(iv) performance information as required by the Department to demonstrate the achievement of commitments linked to specifically allocated funding included in Schedule 2 of this Service Agreement.

(b) utilise the data sets provided for a range of purposes including:

(i) to fulfil legislative requirements;

(ii) to deliver accountabilities to state and commonwealth governments;

(iii) to monitor and promote improvements in the safety and quality of Health Services;

(iv) to support clinical innovation; and

(c) advise the HHS of any updates to data supply requirements as they occur.

Attachment A Data Supply Requirements

The HHS should refer to the relevant minimum data set for full details. These are available on-line as referenced in Appendix 1.

Table 12   Clinical data

Data Set	Data Custodian
Aged Care Assessment Team data via the Aged Care Evaluation (ACE) database	Strategic Policy Unit
Alcohol Tobacco and Other Drug Treatment Services	Mental Health Alcohol and Other Drugs Branch
Alcohol and Other Drugs Establishment Collection	Mental Health Alcohol and Other Drugs Branch
Allied Health Clinical Placement Activity Data	Allied Health Professions Office of Queensland
Australian and New Zealand Intensive Care Society (ANZICS) Data Collection	Healthcare Improvement Unit
BreastScreening Clinical Data	Executive Director, Preventive Health Branch
Clinical Incident Data Set	Patient Safety and Quality Improvement Service
Clinical Placement Data (excluding Allied Health)	Workforce Strategy Branch
Consumer Feedback Data Set	Patient Safety and Quality Improvement Service
Elective Surgery Data Collection	Healthcare Improvement Unit
Emergency Data Collection	Healthcare Improvement Unit
Gastrointestinal Endoscopy Data Collection	Healthcare Improvement Unit
Hand Hygiene Compliance Data	Communicable Diseases Branch
Healthcare Infection Surveillance Data	Communicable Diseases Branch
Maternal Deaths	Queensland Maternal and Perinatal Quality Council (through Statistical Services Branch)
Mental Health Act Data	Mental Health Alcohol and Other Drugs Branch
Mental Health Activity Data Collection	Mental Health Alcohol and Other Drugs Branch
Mental Health Carer Experience Survey Collection	Mental Health Alcohol and Other Drugs Branch
Mental Health Establishments Collection	Mental Health Alcohol and Other Drugs Branch
Monthly Activity Collection (including admitted and non-admitted patient activity and bed availability data)	Statistical Services Branch
Newborn Hearing Screening	Children's Health Queensland
Notifications Data	Chief Health Officer
Patient Experience Survey Data	Patient Safety and Quality Improvement Service
Patient Level Costing and Funding Data	HHS Funding and Costing Unit
Perinatal Data Collection	Statistical Services Branch
Queensland Bedside Audit	Patient Safety and Quality Improvement Service
Queensland Health Non-Admitted Patient Data Collection	Statistical Services Branch
Queensland Hospital Admitted Patient Data Collection	Statistical Services Branch
Queensland Needle and Syringe Program (QNSP) data	Chief Health Officer
Queensland Opioid Treatment Program Admissions and Discharges	Chief Health Officer
Radiation Therapy Data Collection	Healthcare Improvement Unit

Data Set	Data Custodian
Residential Mental Health Care Collections	Mental Health Alcohol and Other Drugs Branch
Schedule 8 Dispensing data	Chief Health Officer
School Immunisation Program – Annual Outcome Report	Communicable Diseases Branch
Specialist Outpatient Data Collection	Healthcare Improvement Unit
National Notifiable Diseases Surveillance System	Chief Health Officer
Vaccination Administration data	Chief Health Officer
Variable Life Adjusted Display (VLAD) CM (collection of hospital investigations)	Patient Safety and Quality Improvement Service
Your Experience of Service (YES) Survey Collection (Mental Health)	Mental Health Alcohol and Other Drugs Branch

Table 13 Non-clinical data

Non-Clinical Data Set	Data Custodian
Asbestos management data	Capital and Asset Services Branch
Asset Management<br>• Planning<br>• Maintenance<br>• Maintenance Budget<br>• Statement of Building Portfolio Compliance<br>• Benchmarking & Performance Data	Capital and Asset Services Branch
Conduct and Performance Excellence (CaPE)	Human Resources Branch
Expenditure	Finance Branch
Financial and Residential Activity Collection (FRAC)	Statistical Services Branch
Graduate Nursing Recruitment Data Statewide using the Public Service Commission Graduate Portal System	Office of the Chief Nursing and Midwifery Officer
Hospital Car Parks (including Government Portfolio Model funding arrangements)	Capital and Asset Services Branch
Minimum Obligatory Human Resource Information (MOHRI)	Finance Branch
Minor Capital Funding Program expenditure & forecast data	Finance Branch
Recruitment Data	Human Resources Branch
Revenue	Finance Branch
Queensland Health Workforce & Work Health & Safety Data	Human Resources Branch
Queensland Integrated Safety Information Project (QISIP)Solution Minimum Data Set	Human Resources Branch
Statewide employment matters	Human Resources Branch
Sustaining Capital Reporting Requirements (other than minor capital)	Capital and Asset Services Branch
Whole of Government Asset Management Policies data	Capital and Asset Services Branch

Schedule 5 Amendments to this Service Agreement

1. Purpose

This Schedule 5 sets out the mechanisms through which this Service Agreement may be amended during its term, consistent with the requirements of the *Hospital and Health Boards Act 2011.*

2. Principles

2.1 It is acknowledged that the primary mechanism through which HHS funding adjustments are made is through the budget build process that is undertaken annually in advance of the commencement of the financial year. This approach is intended to provide clarity, certainty and transparency in relation to funding allocations.

2.2 Amendments to the clauses of this Service Agreement should be progressed for consideration as part of the annual budget build process.

2.3 It is recognised that there is a requirement to vary funding and activity in-year. The following principles will guide amendments and amendment processes:

(a) funding allocations to HHSs should occur as early as possible within a financial year if unable to be finalised in advance of a given financial year;

(b) the number of Amendment Windows each year should be minimised to reduce the administrative burden on HHSs and the Department;

(c) Amendment Proposals should be minimised wherever possible and should always be of a material nature;

(d) Amendment Windows 2 and 3 are not intended to include funding or activity variations that could have been anticipated in advance of the financial year;

(e) Amendment Windows are intended to provide a formal mechanism to transact funding or activity variations in response to emerging priorities;

(f) Extraordinary Amendment Windows are not intended to be routinely used.

2.4 The Department remains committed to the ongoing simplification and streamlining of amendment processes.

3. Process to amend this Service Agreement

3.1 The Parties recognise the following mechanisms through which an amendment to this Service Agreement can be made:

(a) Amendment Windows;

(b) Extraordinary Amendment Windows;

(c) periodic adjustments; and

(d) end of year financial adjustments.

3.2 Amendment Windows

(a) In order for the Department to manage amendments across all HHS Service Agreements and their effect on the delivery of Public Sector Health Services in Queensland, proposals to amend this Service Agreement will be negotiated and finalised during set periods of time during the year (Amendment Windows).

(b) Amendment Windows are the primary mechanism through which amendments to this Service Agreement are made.

(c) Amendment Windows occur three times within a given financial year:

(i) Amendment Window 1: Annual Budget Build;

(ii) Amendment Window 2: In-year variation; and

(iii) Amendment Window 3: In-year variation.

(d) A Party that wants to amend the terms of this Service Agreement must give an Amendment Proposal to the other party.

(e) While a Party may submit an Amendment Proposal at any time, an Amendment Proposal will only be formally negotiated and resolved during one of the Amendment Windows outlined in Table 14 (excluding Extraordinary Amendment Windows).

Table 14 Amendment Window Exchange Dates

Amendment Window	Exchange Date	Primary Focus
Amendment Window 2: In-year variation	4 October 2019	2019/20 in-year variations
Amendment Window 3: In-year variation	14 February 2020	2019/20 in-year variations
Amendment Window 1: Annual Budget Build	27 March 2020	2020/21 budget build
Amendment Window 2: In-year variation	9 October 2020	2020/21 in-year variations
Amendment Window 3: In-year variation	12 February 2021	2020/21 in-year variations
Amendment Window 1: Annual Budget Build	26 March 2021	2021/22 budget build
Amendment Window 2: In-year variation	8 October 2021	2021/22 in-year variations
Amendment Window 3: In-year variation	11 February 2022	2021/22 in-year variations

(f) An Amendment Proposal is made by:

(i) the responsible Deputy Director-General signing and providing an Amendment Proposal to the HHS-SA Contact Person through Healthcare Purchasing and System Performance Division prior to the commencement of any Amendment Window; or

(ii) the Health Service Chief Executive signing and providing an Amendment Proposal to the D-SA Contact Person prior to the commencement of any Amendment Window.

(g) A Party giving an Amendment Proposal must provide the other Party with the following information:

(i) the rationale for the proposed amendment;

(ii) the precise drafting for the proposed amendment;

(iii) any information and documents relevant to the proposed amendment; and

(iv) details and explanation of any financial, activity or service delivery impact of the amendment.

(h) Negotiation and resolution of Amendment Proposals will occur during the Negotiation Period through a tiered process, as outlined in Figure 3.

Figure 3 Amendment Proposal negotiation and resolution

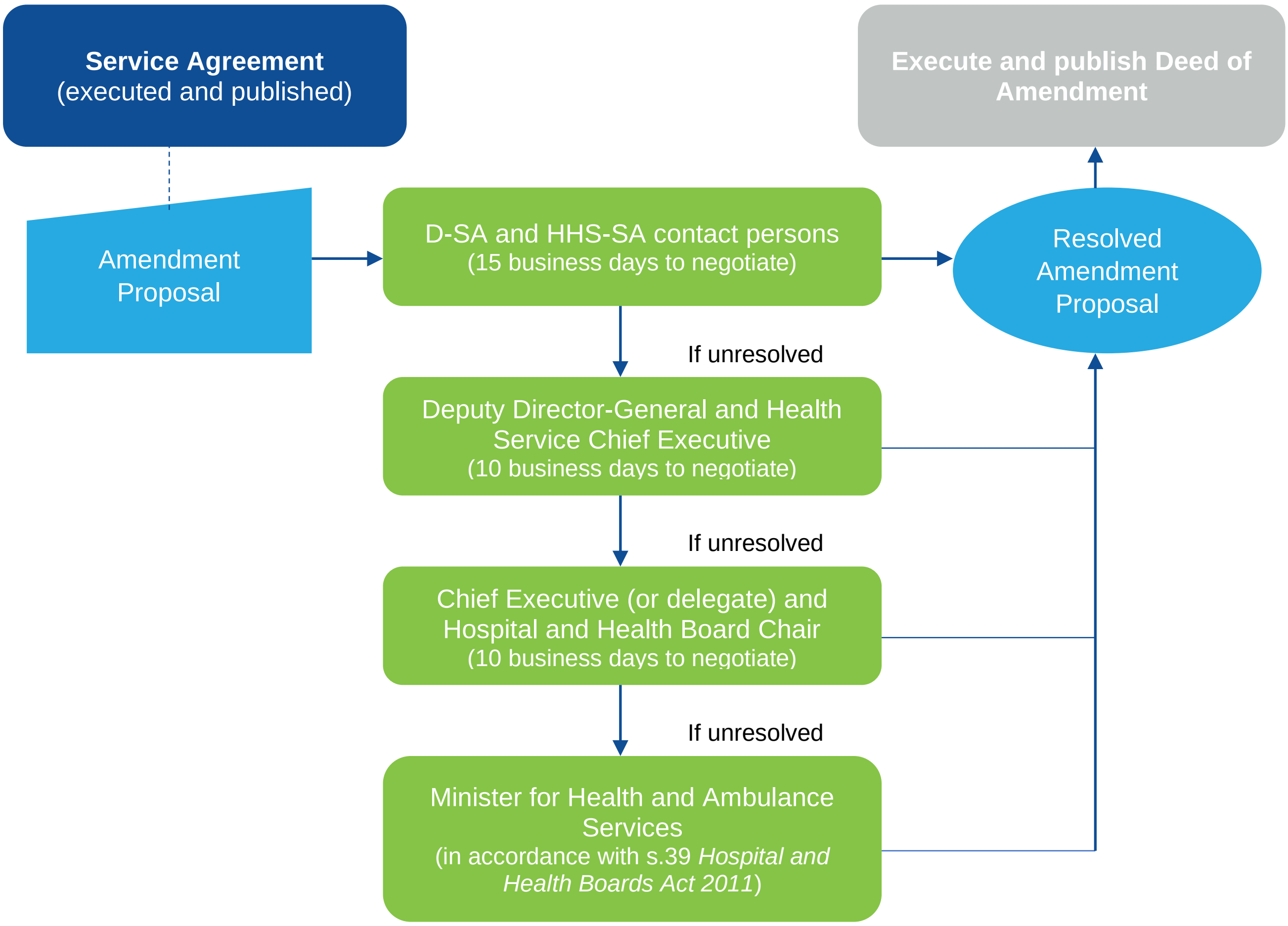

(i) The timeframes identified in Figure 3 may be reduced in the complete discretion of the Chief Executive.

(j) In accordance with section 39(5) of the *Hospital and Health Boards Act 2011*, the Parties must include any terms decided by the Minister in the Service Agreement.

(k) If the Chief Executive at any time:

(i) considers that an amendment agreed with the HHS may or will have associated impacts on other HHSs; or

(ii) considers it appropriate for any other reasons,

then the Chief Executive may:

(iii) propose further amendments to any HHS affected; and

(iv) may address the amendment and/or associated impacts of the

amendment in other ways, including through the exercise of any statutory powers and/or statutory directions under the *Hospital and Health Boards Act 2011*.

(l) Amendment Proposals that are resolved will be documented in a Deed of Amendment to this Service Agreement and executed by the Parties.

(m) Only upon execution of a Deed of Amendment by the Parties will the amendments documented by that Deed of Amendment be deemed to be an amendment to this Service Agreement.

3.3 **Extraordinary Amendment Windows**

(a) A Party that wants to amend the terms of this Service Agreement outside of an Amendment Window outlined in Table 14 must give an Extraordinary Amendment Proposal to the other Party.

(b) An Extraordinary Amendment Proposal may only be formally negotiated and resolved outside of an Amendment Window outlined in Table 14 to facilitate funding allocations where an urgent priority needs to be addressed in a timely manner and an Amendment Window is not available within an acceptable timeframe.

(c) An Extraordinary Amendment Proposal that is issued by or on behalf of the Chief Executive must be given to the HHS-SA Contact Person through Healthcare Purchasing and System Performance Division.

(d) An Extraordinary Amendment Proposal that is issued by or on behalf of the HHS must be given to the D-SA Contact Person through Healthcare Purchasing and System Performance Division.

(e) An Extraordinary Amendment Proposal may be issued by or on behalf of either Party at any time, noting the requirement that it relate to an urgent priority that necessitates timely resolution.

(f) Negotiation and resolution of Extraordinary Amendment Proposals will be through a tiered process as outlined in Figure 3.

(g) The timeframes identified in Figure 3 may be reduced in the complete discretion of the Chief Executive.

(h) In accordance with section 39(5) of the *Hospital and Health Boards Act 2011*, the Parties must include any terms decided by the Minster in the Service Agreement.

(i) Extraordinary Amendment Proposals that are resolved must be executed by both Parties.

(j) The Parties must comply with the terms of the Extraordinary Amendment Proposal from the date that the final Party executed the Extraordinary Amendment Proposal.

(k) The terms of an executed Extraordinary Amendment Proposal will be documented in a Deed of Amendment to this Service Agreement and executed by the Parties. Once executed, the Deed of Amendment will expressly exclude the application of the Extraordinary Amendment Proposal and only the terms of the Deed of Amendment will apply.

3.4 Periodic adjustments

(a) The Service Agreement Value may be adjusted outside of an Amendment Window to allow for funding variations that:

(i) occur on a periodic basis;

(ii) are referenced in the Service Agreement; and

(iii) are based on a clearly articulated formula.

(b) Adjustments to the Service Agreement Value and purchased activity that are required as a result of a periodic adjustment will be made following agreement between the Parties of the data on which the adjustment is based.

(c) The Chief Executive (or delegate) will issue an Adjustment Notice to the HHS-SA Contact Person confirming any funding adjustment that has been made.

(d) Following receipt of an Adjustment Notice, the Parties will comply with the Adjustment Notice and immediately take the steps necessary to give effect to the requirements of the Adjustment Notice.

(e) A Deed of Amendment will not be issued immediately following periodic adjustment. The HHS will be provided with a summary of all transactions made through periodic adjustment on completion.

(f) Any funding adjustments agreed through periodic adjustment which result in a variation to the Service Agreement Value, purchased activity or the requirements specified within Schedule 2 of this Service Agreement will be formalised in a Deed of Amendment issued following the next available Amendment Window.

3.5 End of financial year adjustments

(a) End of year financial adjustments may be determined after the financial year end outside of the Amendment Window process.

(b) The scope will be defined by the Department and informed by Queensland Government Central Agency requirements.

(c) The Department will provide the HHS with a reconciliation of all Service Agreement funding and purchased activity for the prior financial year. This will reflect the agreed position between the Parties following conclusion of the end of year financial adjustments process.

(d) The impact of end of year financial adjustments on subsequent year funding and activity will be incorporated in the Service Agreement through the Deed of Amendment executed following the next available Amendment Window.

(e) This clause will survive expiration of this Service Agreement.

Schedule 6 Definitions

In this Service Agreement:

Activity Based Funding (ABF) means the funding framework for publicly-funded health care services delivered across Queensland. The ABF framework applies to those Queensland public sector health service facilities which are operationally large enough and have the systems which are required to support the framework. The ABF framework allocates health funding to these hospitals based on standardised costs of health care services (referred to as 'activities') delivered. The Framework promotes smarter health care choices and better care by placing greater focus on the value of the health care delivered for the amount of money spent.

Adjustment Notice means the written notice of a proposed funding adjustment made by or on behalf of the Chief Executive in accordance with the terms of this Service Agreement.

Administrator of the National Health Funding Pool means the position established by the *National Health Reform Amendment (Administrator and National Funding Body) Act 2012* for the purposes of administering the National Health Funding Pool according to the National Health Reform Agreement.

Agreement means this Service Agreement.

Ambulatory Care means the care provided to hospital patients who are not admitted to the hospital, such as patients of emergency departments and outpatient clinics. Can also be used to refer to care provided to patients of community-based (non-hospital) healthcare services.

Amendment Proposal means the written notice of a proposed amendment to the terms of this Service Agreement as required under section 39 of the *Hospital and Health Boards Act 2011*.

Amendment Window means the period within which Amendment Proposals are negotiated and resolved. Amendment Windows commence on the relevant Exchange Date as specified in Table 14 Schedule 5 and end at the conclusion of the Negotiation Period.

Block Funding means funding for those services which are outside the scope of ABF.

Business Day means a day which is not a Saturday, Sunday or public holiday in Brisbane.

Chair means the Chair of the Hospital and Health Board.

Chief Executive means the chief executive of the Department.

Clinical Product/Consumable means a product that has been Clinically Prescribed.

Clinically Prescribed means prescribed by appropriately qualified and credentialed clinicians relative to the product.

Clinical Prioritisation Criteria means Statewide minimum criteria to determine if a referral to specialist medical or surgical outpatients is appropriate and, if so, the urgency of that referral.

Clinical Services Capability Framework means the Clinical Services Capability Framework for Public and Licensed Private Health Facilities which provides a standard set of minimum capability criteria for service delivery and planning. The Framework outlines the minimum service requirements, staffing, support services and risk considerations for both public and private health services to ensure safe and appropriately supported clinical service delivery. It applies to all public and private licensed facilities in Queensland. References to the Clinical Services Capability

Framework in this Service Agreement mean the most recent approved version unless otherwise specified.

Community Health Service means non-admitted patient Health Services, excluding hospital outpatient services, typically delivered outside of a hospital setting.

Deed of Amendment means the resolved amendment proposals.

Department means the department administering the *Hospital and Health Boards Act 2011* (Qld), which, at the date of this Service Agreement is known as 'Queensland Health'. To avoid any doubt, the term does not include the Hospital and Health Services.

D-SA Contact Person means the position nominated by the Department as the primary point of contact for all matters relating to this Service Agreement.

Effective Date means1 July 2019.

Efficient Growth means the increased in-scope activity-based services delivered by a HHS measured on a year to year basis in terms of both the Queensland efficient price for any changes in the volume of services provided and the growth in the national efficient price of providing the existing volume of services.

Eligible Population (Oral Health Services) refers to the proportion of the population for whom publicly funded oral health services is to be provided and is defined by the following criteria:

(a) adults, and their dependents, who are Queensland residents; eligible for Medicare and, where applicable, currently in receipt of benefits from at least one of the following concession cards:

  (i) Pensioner Concession Card issued by the Department of Veteran's Affairs;

  (ii) Pensioner Concession Card issued by Centrelink;

  (iii) Health Care Card (this includes Low Income Health Care Card Holders who are automatically eligible for services);

  (iv) Commonwealth Seniors Health Card;

  (v) Queensland Seniors Card.

(b) children who are Queensland residents or attend a Queensland school, are eligible for Medicare, and are:

  (i) eligible for dental program/s funded by the Commonwealth Government; or

  (ii) four years of age or older and have not completed Year 10 of secondary school; or

  (iii) dependents of current concession card holders or hold a current concession card.

Exchange Date means the date on which the Parties must provide Amendment Proposals for negotiation, as specified in Table 14 Schedule 5.

Extraordinary Amendment Window means an Amendment Window that occurs outside of the Amendment Windows specified in Table 14 Schedule 5, in accordance with the provisions of clause 3.3 of Schedule 5.

Force Majeure means an event:

(a) which is outside of the reasonable control of the Party claiming that the event has occurred; and

(b) the adverse effects of which could not have been prevented or mitigated against by that Party by reasonable diligence or precautionary measures, and includes lightning, earthquake, fire, cyclone, flood, natural disasters, health pandemics, acts of terrorism, riots, civil disturbances, industrial disputes and strikes (other than strikes involving that Party, its' agents, employees or suppliers), war (declared or undeclared), revolution, or radioactive contamination.

Formal Agreement means an agreed set of roles and responsibilities relating to the provision and receipt of services designated as Statewide or Regional:

(a) Statewide or Regional service provision

- (i) ensure equitable and timely access to entire catchment (clinical and non-clinical)
- (ii) provide training and consultation Services where this is appropriate within the agreed model of care (clinical and non-clinical)
- (iii) timely discharge or return of patients to their place of residence (clinical Services)
- (iv) adequate communication practices to enable ongoing effective local health care, including with the patient's General Practitioner where required (clinical Services)

(b) Recipient HHS

- (i) utilisation of standardised referral criteria, where they exist, to ensure appropriate use of Statewide Services (clinical services)
- (ii) timely acceptance of patients being transferred out of Statewide Services (back-transfers) (clinical Services)
- (iii) equitable access to ongoing local health care as required (clinical services)

Health Executive means a person appointed as a health executive under section 67(2) of the *Hospital and Health Boards Act 2011.*

Health Service has the same meaning as set out in section 15 of the *Hospital and Health Boards Act 2011.*

Health Service Chief Executive means a health service chief executive appointed for an HHS under section 33 of the *Hospital and Health Boards Act 2011.*

Health Service Employee means all person, appointed as a 'health service employee' for the HHS under section 67(1) of the *Hospital and Health Boards Act 2011.*

Hospital and Health Board means the hospital and health board appointed under section 23 of the *Hospital and Health Boards Act 2011*.

Hospital and Health Service or **HHS** means the Hospital and Health Service to which this Agreement applies unless otherwise specified.

HHS-SA Contact Person means the position nominated by the HHS as the primary point of contact for all matters relating to this Service Agreement.

HR Management Functions means the formal system for managing people within the HHS, including recruitment and selection; onboarding; induction and orientation; capability, learning and development; industrial and employee relations; performance management; work health and safety and well-being; workforce planning; diversity and inclusion; and workforce consultation, engagement and communication.

Industrial Instrument means an industrial instrument made under the *Industrial Relations Act 2016.*

Inter-HHS Dispute means a dispute between two or more HHSs.

Key Performance Indicator means a measure of performance that is used to evaluate the HHSs success in meeting key priorities.

Low Benefit Care means use of an intervention where evidence suggests it confers no or very little benefit on patients, or the risk of harm exceeds the likely benefit.

Minister means the Minister administering the *Hospital and Health Boards Act 2011* (Qld).

National Health Reform Agreement means the document titled *National Health Reform Agreement* made between the Council of Australian Governments (CoAG) in 2011, and incorporating all subsequent amendments agreed between the Commonwealth of Australia and the States and Territories.

Negotiation Period means a period of no less than 15 business days (or such longer period agreed in writing between the Parties) from each Exchange Date.

Notice of Dispute means the written notice of a dispute provided by the Chief Executive or the HHS to the other party or the written notice of a dispute provided by an HHS to another HHS.

Outcome Indicator means a measure of performance that provides information on specific activities and interventions where there is a reasonable expectation or evidence that they make a positive contribution towards improving the health status and experience of patients;

Outreach Service means a Health Service delivered on sites outside of the HHS area to meet or complement local service need. Outreach services include Health Services provided from one HHS to another as well as Statewide Services that may provide Health Services to multiple sites.

Own Source Revenue means, as per Section G3 of the *National Healthcare Agreement*, ‘private patients, compensable patients and ineligible persons may be charged an amount for public hospital services as determined by the state and territory’. The funding for these patients is called own source revenue and includes:

(a) Medicare ineligible patients, such as overseas visitors (not covered under reciprocal agreements), people in community detention and overseas students studying in Australia

(b) compensable patients with an alternate funding source, such as:

  (i) workers’ compensation insurers;

  (ii) motor vehicle accident insurers;

  (iii) personal injury insurers;

  (iv) Department of Defence; and/or

  (v) Department of Veterans’ Affairs; and

  Medicare eligible patients can elect to be treated as a public or private patient, allowing HHS' to recoup a portion of the healthcare service delivery cost.

Party means each of the Chief Executive and the HHS to which this Service Agreement applies.

Patient Identifiable Data means data that could lead to the identification of an individual either directly (for example by name), or through a combination of pieces of data that are unique to that individual.

Performance Review Meeting means the forum established which routinely reviews and discusses a range of aspects of HHS and system wide performance in accordance with the accountabilities contained within this Service Agreement and the Performance and Accountability Framework. Attendance at Performance Review Meetings comprises:

(a) the D-SA Contact Person and the HHS-SA Contact Person;

(b) executives nominated by the Department; and

(c) executives nominated by the HHS.

Performance Measure means a quantifiable indicator that is used to assess how effectively the HHS is meeting identified priorities and objectives.

Person Conducting a Business or Undertaking takes the meaning as defined in the *Work Health and Safety Act 2011,* section 5.

Prevention Services means programs that prevent illness and injury, promote health and wellbeing, create healthy and safe environments, reduce health inequalities and address factors in those communities whose health status is the lowest.

Primary Care means first level healthcare provided by a range of healthcare professionals in socially appropriate and accessible ways and supported by integrated referral systems. It includes health promotion, illness prevention, care of the sick, advocacy and community development.

Public Health Event of State Significance means an event where the actual or potential impact extends beyond the community service by a particular Hospital and Health Service.

Public Sector Health Service has the same meaning as set out in the *Hospital and Health Boards Act 2011.*

Public Sector Health System means the Queensland public sector health system, which is comprised of the Hospital and Health Services and the Department.

Quality Improvement Payment (QIP) means a non-recurrent payment due to the HHS for having met the goals set out in the QIP Purchasing Incentive Specification.

Queensland Government Central Agency means one or all of the Department of the Premier and Cabinet, Queensland Treasury, the Queensland Audit Office, the Public Service Commission and the Office of the Integrity Commissioner.

Regional Service means a clinical (direct or indirect patient care) or non-clinical Health Service funded and delivered, or coordinated and monitored, by an HHS with a catchment of two or more HHSs, but not on a Statewide basis as defined in this Schedule. Service delivery includes facility based, outreach and telehealth service models.

Referral Pathway means the process by which a patient is referred from one clinician to another in order to access the Health Services required to meet their healthcare needs.

Residential HHS means the HHS area, as determined by the *Hospital and Health Boards Regulation 2012*, in which the patient normally resides.

Safety and Quality Marker means a measure of performance that provides timely and transparent information on the safety and quality of Health Services provided by the HHS;

Schedule means this Schedule to the Service Agreement.

Senior Health Service Employee means a person appointed under section 67(2) of the *Hospital and Health Boards Act 2011* in a position prescribed as a 'senior health service employee position' under the *Hospital and Health Boards Regulation 2012.*

Service Agreement means this service agreement including the Schedules and annexures, as amended from time to time.

Service Agreement Value means the figure set out in Schedule 2 as the expected annual value of the services purchased by the Department through this Service Agreement.

State means the State of Queensland.

Statement of Building Portfolio Compliance means a declaration completed by the HHS stating that it has maintained compliance with all mandatory Acts, Regulations, Australian Standards and Codes of Practice applicable to the HHS' building portfolio.

Statewide Service means a service that is delivered by a lead provider to the State. A Statewide Service may be:

(a) a clinical service that is:

 (i) a low volume, highly specialised Health Service delivered from a single location;

 (ii) a highly specialised, or high risk[11], Health Service delivered in multiple locations or

 (iii) a prevention and/or health promotion service.

(b) a support service that is required to enable the delivery of specific direct clinical services; or

(c) services that have a primary role to provide clinical education services and/or training programs.

Statewide Service Description means a document that defines the Service to be provided by the HHS on a statewide basis and how the Statewide Service will be accessed and used by other HHSs across the State, including but not limited to:

(a) an overview of the Statewide Service;

(b) components of the Statewide Service;

(c) eligibility criteria;

(d) Service referrals and pathways; and

(e) governance and capability arrangements for the Statewide Service.

---

[11] A Health Service that, due to its nature, poses an increased threat of ongoing sustainability, efficiency and affordability.

Supporting Indicator means a measure of performance that provides contextual information to support an assessment of HHS performance.

Suspend and Suspension means to cause the temporary cessation of a service provided by the HHS under the terms of this Service Agreement. Suspension may result from, but is not exclusively due to, limitations in workforce capacity or issues regarding the safety or quality of the service provided.

Telehealth means the delivery of Health Services and information using telecommunication technology, including:

(a) live interactive video and audio links for clinical consultations and education;

(b) store and forward systems to enable the capture of digital images, video, audio and clinical data stored on a computer and forwarded to another location to be studied and reported on by specialists;

(c) teleradiology to support remote reporting and provision of clinical advice associated with diagnostic images; and

(d) telehealth services and equipment for home monitoring of health.

Terminate and Termination means the permanent cessation of a service provided by the HHS under the terms of this Service Agreement.

Treating HHS means the HHS area, as determined by the *Hospital and Health Boards Regulation 2012*, in which a patient is receiving treatment.

Value-Based Healthcare means delivering what matters most to patients in the most efficient way. Value-Based Healthcare is characterised by:

(a) the identification of clearly defined population segments of patients with similar needs around which clinically integrated teams organise and deliver care, rather than designing and organising care around medical specialities, procedures or facilities;

(b) a focus on delivering health outcomes and the experience of receiving care as defined from the patient perspective, not just the system or clinical perspective;

(c) connection between outcomes and the costs required to deliver the outcomes; and

(d) an integrated approach across the full cycle of care with a focus on the goal of health rather than just treatment.

Key Documents

Hospital and Health Services Service Agreements and supporting documents including:

(a) Hospital and Health Services Service Agreements

(b) Queensland Health System Outlook to 2026 for a sustainable health service

(c) Performance and Accountability Framework 2020/21

(d) Purchasing Policy and Funding Guidelines 2020/21

are available at: www.health.qld.gov.au/system-governance/health-system/managing/agreements-deeds

My health, Queensland's future: Advancing health 2026

www.health.qld.gov.au/__data/assets/pdf_file/0025/441655/vision-strat-healthy-qld.pdf

Queensland Health 2020-2021 System Priorities

[link to follow]

Department of Health Strategic Plan

www.health.qld.gov.au/system-governance/strategic-direction/plans/doh-plan

Guideline for Immunisation Services

https://www.health.qld.gov.au/__data/assets/pdf_file/0026/147545/qh-gdl-955.pdf

Queensland Health Statement of Action towards Closing the Gap in health outcomes

https://qheps.health.qld.gov.au/atsihb/html/statement-of-action

HHS Performance Measures and Attribute Sheets

https://qheps.health.qld.gov.au/purchasing-performance/hhs-agreements/performance-kpis

Data Supply Requirements

https://qheps.health.qld.gov.au/purchasing-performance/hhs-agreements/data-reporting-requirements

Australian Commission on Safety and Quality in Healthcare – National Safety and Quality Health Service Standards

https://www.safetyandquality.gov.au/standards/nsqhs-standards

Statewide Services Governance and Risk Management Framework

https://qheps.health.qld.gov.au/spb/html/statewide-services/statewide-services-governance-and-risk-management-framework

Public Health Practice Manual

https://qheps.health.qld.gov.au/__data/assets/pdf_file/0035/667754/public-health-prac-man.pdf

National Healthcare Agreement

http://www.federalfinancialrelations.gov.au/content/national_agreements.aspx

National Health Reform Agreement

www.federalfinancialrelations.gov.au/content/national_health_reform.aspx

Abbreviations

ACQSC	Aged Care Quality and Safety Commission
ABF	Activity Based Funding
ACSQHC	Australian Commission on Safety and Quality in Healthcare
CET	Clinical Education and Training
D-SA	Department – Service Agreement
HHS	Hospital and Health Service
HHS-SA	Hospital and Health Service – Service Agreement
HITH	Hospital in the Home
KPI	Key Performance Indicator
LAM	List of Approved Medicines
Non-ABF	Non-Activity Based Funding
NPA	National Partnership Agreement
NSQHS	National Safety and Quality Health Service Standards
NWAU	National Weighted Activity Unit
PBS	Pharmaceutical Benefits Scheme
QAS	Queensland Ambulance Service
QIP	Quality Improvement Payment
QWAU	Queensland Weighted Activity Unit
RACGP	Royal Australian College of General Practitioners
SA2	Statistical Area Level 2

This page is intentionally left blank.

Queensland Health
www.health.qld.gov.au

Queensland Government

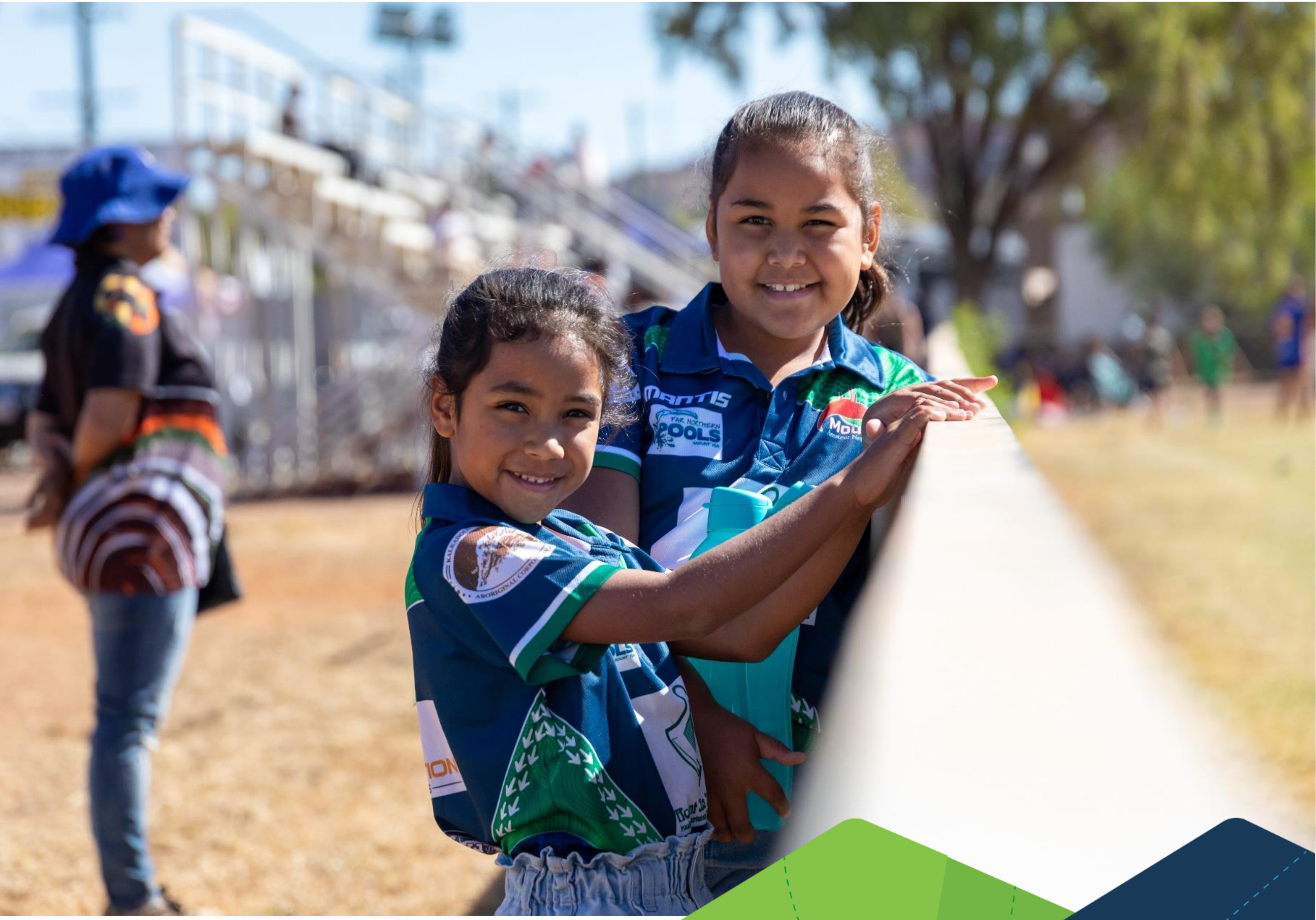

Queensland Health

Service Agreement 2022/23 – 2024/25

North West Hospital and Health Service

Queensland Government

North West Hospital and Health Service, Service Agreement 2022/23 - 2024/25

Published by the State of Queensland, (Queensland Health), July 2022

This document is licensed under a Creative Commons Attribution 3.0 Australia licence. To view a copy of this licence, visit creativecommons.org/licenses/by/3.0/au

© State of Queensland (Queensland Health) **2022**

You are free to copy, communicate and adapt the work, as long as you attribute the State of Queensland (Queensland Health).

For more information contact:

Contracting and Performance Management Branch, Department of Health, GPO Box 48, Brisbane QLD 4001
Tel (+61) (07) 3708 5869

Email SAM@health.qld.gov.au

An electronic version of this document is available at www.health.qld.gov.au/system-governance/health-system/managing/default.asp

Disclaimer:
The content presented in this publication is distributed by the Queensland Government as an information source only. The State of Queensland makes no statements, representations or warranties about the accuracy, completeness or reliability of any information contained in this publication. The State of Queensland disclaims all responsibility and all liability (including without limitation for liability in negligence) for all expenses, losses, damages and costs you might incur as a result of the information being inaccurate or incomplete in any way, and for any reason reliance was placed on such information.

Acknowledgement

We acknowledge the Traditional and Cultural Custodians of the lands, waters and seas across Queensland, pay our respects to Elders past and present, and recognise the role of current and emerging leaders in shaping a better health system.

We acknowledge the First Nations people in Queensland are both Aboriginal Peoples and Torres Strait Islander Peoples, and support the cultural knowledge, determination and commitment of Aboriginal and Torres Strait Islander communities in caring for health and wellbeing for millennia.

North West Hospital and Health Service is committed to reducing inequalities between Indigenous and non-Indigenous health outcomes in line with the Australian Government's Closing the Gap initiative.

North West Hospital and Health Service is proud to recognise and celebrate the cultural diversity of our communities and workforce at the following locations:

**Location**	**Traditional Custodians**
Burketown	Gangalidda and Garawa
Camooweal	Indjalandji / Dhidhnu
Cloncurry	Mitakoodi
Dajarra	Waluwarra and Yulluna
Doomadgee	Gangalidda and Waanyi
Mornington Island	Lardil (other historical groups from surrounding islands include Yangkal and Kaiadilt
Normanton	Kurtijar, Gkuthaarn, Kukatj
Mount Isa	Kalkadoon

Contents

Acknowledgement 3

Contents 4

Tables 5

1. Introduction 6
2. Scope 6
3. Services 6
4. Performance and Accountability Framework 7
5. Data supply requirements 7
6. Hospital and Health Service accountabilities 8
7. Department accountabilities 9
8. Achieving health equity with First Nations Queenslanders 9
9. General 10
10. Counterparts 11

Execution 12

Schedule 1 HHS profile 13
1. HHS profile 13

Schedule 2 Funding and purchased activity and services 14
1. Introduction 14
2. Purchased health services 15
3. Teaching training and research 17
4. Delivery of purchased activity 18
5. Delivery of purchased services 19
6. Financial adjustments 19
7. Funding sources 23
8. Funds disbursement 24

Schedule 3 Performance Measures 29
1. Performance Measures 29

Schedule 4 Amendments to this service agreement 31
1. Agreed process to amend this service agreement 31

Appendix 1 Reference Documents 33

Tables

Table 1 Statewide Services 15
Table 2 Financial adjustments applied on breach of activity thresholds 20
Table 3 Purchasing Incentives 2022/23 21
Table 4 North West HHS Total Funding Allocation by Funding Source 2022/23 25
Table 5 National Health Reform Funding 27
Table 6 Discretely Funded Programs (Non-ABF) 28
Table 7 HHS Performance Measures – Key Performance Indicators 29
Table 8 HHS Performance Measures - Safety and Quality Markers 30
Table 9 HHS Performance Measures – Outcome Indicators 30

1. Introduction

1.1 In performing this service agreement, the Department and the Hospital and Health Service (HHS) will act consistently with the object of the *Hospital and Health Boards Act 2011* (Qld) (Act).

1.2 The Department and the HHS operate as part of a networked system and agree to work collaboratively with each other, with other HHSs and with the Queensland Ambulance Service in the best interests of the Queensland public sector health system.

1.3 The parties will ensure that planning and delivery of health services is consistent with the strategies and priorities set out by government. The parties recognise the importance of the HHS' Health Equity Strategy (as defined in the Act) and the parties commitment to improving health and wellbeing outcomes and achieving health equity for Aboriginal and Torres Strait Islander peoples.

2. Scope

2.1 This service agreement covers the period from 1 July 2022 to 30 June 2025.

2.2 The parties have identified the services to be provided by the HHS, the funding for the provision of those services, the performance measures applicable to the services and data requirements.

3. Services

3.1 In delivering services, the HHS is required to meet:

(a) the applicable conditions of all national agreements and national partnership agreements between the Queensland Government and the Commonwealth Government and commitments under any related implementation plans; and

(b) the applicable conditions of each agreement or arrangement for funding between the Department and the HHS and commitments under any related implementation plans, which conditions and commitments may be recorded separately to this service agreement but for which funding is (or becomes) provided in Schedule 2.

3.2 The HHS is required to deliver the services outlined in this service agreement for which funding is provided in Schedule 2.

3.3 Where issues arise which prevent the HHS from providing a service or necessitate a reduction in the level or scope of a service provided, prompt notification must be made to the Department and impacted HHSs, with appropriate details. The HHS must minimise any clinical risk or adverse impact to patient experience that may result from service disruption. The Department will respond to the HHS on any requirements it has concerning service delivery and any adjustments triggered by under delivery.

3.4 If the HHS wishes to terminate or reduce service levels for a service for which funding is provided in Schedule 2, this will remain subject to negotiation and agreement by the Department at its discretion.

3.5 For any new services proposed during the term of this service agreement, the parties agree:

(a) the service must meet a demonstrated clinical need and provide value for money;

(b) the commencement of a new service, including the implementation of new models of care, may occur where the service has been commissioned by the Department, a funding stream is in place and any conditions relating to the funding have already been agreed; and

(c) if the HHS wishes to commence providing a new service that has not been commissioned by the Department, this will remain subject to agreement by the Department at its discretion.

3.6 It is acknowledged that there may from time to time need to be service delivery changes between HHSs. Management of inter-HHS relationships should be informed by the following principles:

(a) HHSs should maintain the proportion of out of HHS work undertaken unless as a result of agreed repatriation of patients;

(b) each HHS should manage patients from its own catchment population if it is within its clinical capability to do so as specified by the Clinical Services Capability Framework; and

(c) where it is proposed that a service move from one HHS to another, the Department will consider, as part of its review under clause 3.4 and 3.5, whether the respective Health Service Chief Executives endorse the proposed change in patient flows; and the funding required to follow the patient.

4. Performance and Accountability Framework

4.1 The Queensland Health Performance and Accountability Framework (the Performance and Accountability Framework) sets out the framework within which the Department monitors and manages the performance of public sector health services in Queensland.

4.2 The parties will act consistently with the Performance and Accountability Framework.

5. Data supply requirements

5.1 The HHS will provide the Department with all clinical and non-clinical data that is reasonably required to support the effective management of the public sector health system. This will include, but is not limited to, data that is required to:

(a) fulfil legislative obligations;

(b) deliver accountabilities and obligations to State and Commonwealth Governments; including related to the provision and reconciliation of activity data by the Administrator of the National Health Funding Pool;

(c) monitor and support performance improvement;

(d) manage this service agreement;

(e) support clinical innovation; and

(f) facilitate evaluation and audit.

5.2 The parties agree and acknowledge that:

(a) the Department will keep the HHS informed of the Department's data requirements; and

(b) data will be provided as required, or permitted, by law.

6. Hospital and Health Service accountabilities

6.1 The HHS will perform its obligations under this service agreement.

6.2 As applicable to the HHS and its services, the HHS will comply with:

(a) legislation and subordinate legislation, including the Act;

(b) cabinet decisions;

(c) Ministerial directives;

(d) agreements entered into between the Queensland and Commonwealth governments (or agreements with others in furtherance of such agreements), of which it is informed;

(e) agreements entered into between the Department and other Queensland Government entities, of which it is informed;

(f) all industrial instruments;

(g) all health service directives and health employment directives; and

(h) all policies, guidelines and implementation standards, including human resource policies.

6.3 As part of the commitment to achieving First Nations health equity, the HHS will prioritise the elimination of racial discrimination and institutional racism within its service and ensure that Aboriginal and Torres Strait Islander peoples have access to culturally safe and responsive health services.

6.4 The HHS will ensure that effective health service planning and delivery systems are in place, working in collaboration with the Department.

6.5 The HHS will ensure that health service employees employed by the Chief Executive[1] who perform work for the HHS are managed in accordance with any applicable delegations and directions from the Chief Executive.

6.6 The HHS will ensure that effective asset management systems are in place, working in collaboration with the Department.

6.7 The HHS will maintain accreditation to the standards required by the Department.

6.8 The HHS will appropriately perform and fulfil its functions under the Act.

6.9 The HHS will provide to the Chief Executive reports of a type, and at the intervals, agreed between the parties, or as reasonably specified by the Chief Executive.

7. Department accountabilities

7.1 The Department will perform its obligations under this service agreement including, in return for the HHS performing its obligations and delivering the services, providing funding to the HHS as stipulated in this service agreement (as amended).

7.2 The Department will:

(a) comply with applicable legislation and subordinate legislation, including the Act, as relates to this service agreement; and

(b) perform the system manager role (as defined in the Act) through the Chief Executive;

7.3 The Chief Executive will appoint health service employees to perform work for the HHS for the purpose of enabling the HHS to perform its functions under the Act.

7.4 The Chief Executive will consult, cooperate, and coordinate with the HHS to ensure legal compliance with the *Work Health and Safety Act 2011* and other legislation as it applies to the scope, nature and location of operations associated with this service agreement.

7.5 The Chief Executive will appropriately perform and fulfil their functions under the Act.

8. Achieving health equity with First Nations Queenslanders

8.1 Through legislative amendments to the Act and the Hospital and Health Boards Regulation 2012, and the release of *Making Tracks Together: Queensland's Aboriginal and Torres Strait Islander Health Equity Framework* (2021), Queensland Health has strengthened its commitment to improving health and wellbeing outcomes and achieving health equity for First Nations peoples.

8.2 The HHS will develop a Health Equity Strategy to demonstrate the HHS's activities and key performance measures to achieve health equity with First Nations peoples that is compliant with legislative requirements. The Health Equity Strategy will act as the principal

[1] In this service agreement the term Chief Executive takes the meaning applied in the Act, which is the Chief Executive of Queensland Health, but which is generally referred to as the Director-General.

accountability mechanism between community and the HHS in achieving health equity for First Nations Queenslanders and forms a key part of the Queensland Government's commitment to the *National Agreement on Closing the Gap* (2020).

8.3 The HHS will review the Health Equity Strategy at least once every three years and will publish the Health Equity Strategy in a way that allows it to be accessed by members of the public.

8.4 The HHS will ensure that commitment and leadership is demonstrated through implementing the actions and achieving the key performance measures outlined in the Health Equity Strategy.

8.5 The HHS will report publicly on progress against the Health Equity Strategy.

8.6 The HHS will support the implementation of other supplementary policies and strategies to drive health equity across the public health system, including relevant election commitments.

8.7 The HHS will participate as a partner in the design, development and implementation of the new *Queensland First Nations Health Workforce Strategy for Action.*

9. General

9.1 Sub-contracting

(a) The HHS must have appropriate systems in place to ensure that any subcontractor is accredited (as applicable), qualified, and otherwise fit to perform any services for which it is contracted.

(b) The HHS must ensure that any sub-contractor who has access to confidential information (as defined in the Act) or personal information (as defined in the *Information Privacy Act 2009* (Qld)) complies with obligations no less onerous than those imposed on the HHS.

9.2 Insurance

The HHS must:

(a) hold and maintain the types and levels of insurances that the HHS considers appropriate according to its functions and obligations; and

(b) comply with reasonable requests or directions from the Department in this regard.

9.3 Amendment

The process for amending this service agreement is provided for under the Act and further outlined in Schedule 4.

10. Counterparts

10.1 This service agreement may be executed in two or more identical copy counterparts, each of which together will be deemed an original, but all of which together will constitute one and the same instrument.

10.2 In the event that any signature executing this service agreement or any part of this service agreement is delivered by facsimile transmission or by scanned e-mail delivery of a '.pdf' format data file or equivalent of the entire agreement, the signature will create a valid and binding obligation of the party executing (or on whose behalf the signature is executed) with the same force and effect as if the signature page were an original. For the avoidance of doubt, this service agreement may be in the form of an electronic document and may be electronically signed.

10.3 For execution under this clause 10 to be valid the entire service agreement upon execution by each individual party must be delivered to the remaining parties.

Execution

Executed as an agreement in Queensland

Signed by the Chief Executive, Queensland Health:)

....

Signature of Chief Executive

SHAUN DRUMMOND

....

Name of Chief Executive (print)

....

(date)

Signed for and on behalf of the North West Hospital and Health Service:)

....

Signature of Hospital and Health Board Chair

Cheryl Vardon

....

Name of Hospital and Health Board Chair (print)

20 June 2022

....

(date)

Execution

Executed as an agreement in Queensland

Signed by the Chief Executive, Queensland)
Health:)

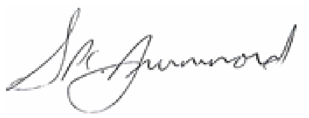

....

Signature of Chief Executive

SHAUN DRUMMOND

....

Name of Chief Executive (print)

29 June 2022

....

(date)

Signed for and on behalf of the North West)
Hospital and Health Service:)

....

Signature of Hospital and Health Board Chair

....

Name of Hospital and Health Board Chair (print)

....

(date)

Schedule 1 HHS profile

1. HHS profile

North West Hospital and Health Service delivers health services to the communities of North West Queensland, serving a resident population of around 30,000 people that significantly increases with visitors and tourists annually, with one regional hospital, two multi-purpose health services, four primary health clinics and five community health centres. Mount Isa Hospital, as the regional hospital, is the primary referral centre within North West HHS.

With one of the highest proportions of Aboriginal and Torres Strait Islander populations in Queensland, North West HHS is committed to improving health outcomes for First Nations people with the Making Tracks program which focuses on chronic diseases, sexual health, discharge against medical advice, and maternal and infant health.

The North West HHS region’s population is slowly declining with an increasing proportion of aged community members and an overall lower socio-economic population where high levels of acute and chronic disease are evident. These demographics place an increasing demand on public heath services delivered across a geographical area of 300,000 kilometres stretching across north western Queensland and the Gulf of Carpentaria.

Increasing the use of and available infrastructure for communication technologies is essential for North West HHS to continue to deliver contemporary models of care to the community closer to home.

North West HHS partners with a broad range of organisations and service providers to enhance the services available to communities in the region, including:

- Gidgee Healing, the regional Aboriginal Controlled Health Service for North West Queensland
- Royal Flying Doctor Services
- Western Queensland Primary health Network
- Queensland Ambulance Service and Queensland Police Service
- Seven City, Shire and Aboriginal Shire Councils
- Outreach allied and medical health services including CheckUp, the Deadly Ears and Indigenous Respiratory Outreach Care (IROC) programs
- Centracare, Headspace and other charitable not for profit enterprises
- James Cook University’s Centre for Rural and Remote Health

Schedule 2 Funding and purchased activity and services

This Schedule 2 sets out:

(a) the services which are to be provided by the HHS;

(b) the activity purchased by the Department from the HHS;

(c) the funding provided for delivery of the purchased activity;

(d) the criteria and processes for financial adjustments associated with the delivery of purchased activity and specific funding allocations;

(e) the sources of funding that this service agreement is based on and the manner in which these funds will be provided to the HHS.

1. Introduction

1.1 The HHS will deliver the services for which funding is provided in this Schedule 2. In providing these services, the HHS will ensure that:

(a) all statewide and national policy frameworks, guidelines, protocols and implementation standards applicable to the service provided are followed;

(b) participation in national programs is facilitated and supported, including where these programs are provided by the Commonwealth Government;

(c) service delivery partnerships, including with other HHSs, primary care organisations and non-government organisations, are maintained and operate effectively;

(d) collaboration and engagement with other service providers and stakeholders is initiated and maintained to ensure that an integrated system of treatment, care and support is in place and to facilitate the delivery of comprehensive and effective services. This may include but is not limited to:

(i) other HHSs;

(ii) non-government organisations;

(iii) Aboriginal and Torres Strait Islander community-controlled health organisations;

(iv) Queensland Ambulance Service;

(v) services provided through the Department of Health (for example, Pathology Queensland);

(vi) primary care providers;

(vii) other government departments and agencies; and

(viii) private providers;

(e) models of care and service delivery arrangements are consistent with evidence-based practice and offer value for money;

(f) services are provided on an equitable basis to the community, and processes are in place to ensure that services reach identified target populations, high risk groups and hard to reach communities;

(g) referral networks and pathways continue to operate effectively; and

(h) innovation and continuous improvement is supported.

2. Purchased health services

2.1 Table 4, Table 5 and Table 6 outline the activity and service streams which the Department agrees to purchase from the HHS pursuant to this service agreement.

2.2 More generally, this will include the following:

2.3 **Statewide Services**

(a) The designation of a service as a statewide service (either a clinical statewide service or a clinical support statewide service) will be determined by the Department, consistent with the stipulated governance arrangements for such services.

(b) The HHS will:

(i) collaborate with the Department and other HHSs in the implementation of, and adherence to, the governance and oversight arrangements for statewide services;

(ii) participate in, and contribute to, the staged review of the purchasing model for identified statewide services; and

(iii) ensure that referral pathways in and out of each statewide service are followed.

2.4 **Clinical Statewide Services and Clinical Support Statewide Services provided**

This clause does not apply to this HHS.

Table 1 Statewide Services

This table does not apply to this HHS.

2.5 **Regional services**

This clause does not apply to this HHS.

2.6 **Prevention services and population health services**

(a) The HHS will provide a range of services with a focus on the prevention of ill-health and disease, including:

(i) Specialist Public Health Units;

(ii) preventive health services;

(iii) immunisation services;

(iv) sexually transmissible infections including HIV and viral hepatitis;

(v) tuberculosis services; and

(vi) population health screening including, but not limited to, cancer screening services and newborn blood spot screening.

(b) Services will be provided in line with public health related legislation and the service delivery and reporting requirements outlined in the *Public Health Practice Manual*, as these relate to the services provided.

2.7 Aboriginal and Torres Strait Islander health services

The HHS will provide Aboriginal and Torres Strait Islander specific health services and initiatives consistent with the principles and objectives of the Queensland Government's *Making Tracks towards closing the gap in health outcomes for Indigenous Queenslanders by 2022 – Policy and Accountability Framework*. These service and initiatives will be delivered in line with guidance from the Aboriginal and Torres Strait Islander Health Division.

2.8 Mental health alcohol and other drugs services

The HHS will provide treatment, care and support for individuals who are, or may be, experiencing substance use disorders and/or other mental health disorders, mental health crisis and suicidal distress, and their families and carers. Services will be delivered in line with guidance from the Mental Health Alcohol and Other Drugs Branch:

2.9 Oral health services

The HHS will provide oral health services to people who meet the eligibility criteria for accessing public dental services in Queensland. Services will be delivered in line with guidance from the Office of the Chief Dental Officer.

2.10 Prisoner health services

This clause does not apply to this HHS.

2.11 Youth detention services

This clause does not apply to this HHS.

2.12 Refugee health

This clause does not apply to this HHS.

2.13 State-funded outreach services

(a) Where state-funded outreach services are provided;

(i) funding for the service will remain part of the providing HHS's funding allocation; and

(ii) the activity must be recorded at the HHS where the outreach service is being provided

(b) Any changes to the provision of outreach services will follow the requirements set out in clause 3 of this service agreement.

3. Teaching training and research

The HHS will provide the teaching, training and research programs for which funding is provided within Schedule 2 and as described below.

3.1 Clinical education and training

(a) The HHS will provide education and training placements for the following professional groups consistent with and proportionate to the capacity of the HHS and will support and align with stipulated placement terms governing clinical placements in Queensland Health facilities;

- (i) medical students;
- (ii) nursing and midwifery students;
- (iii) pre-entry clinical allied health students;
- (iv) interns;
- (v) rural generalist trainees;
- (vi) vocational medical trainees;
- (vii) first year nurses and midwives;
- (viii) re-entry to professional register nursing and midwifery candidates;
- (ix) dental students;
- (x) allied health rural generalist training positions;
- (xi) Aboriginal and Torres Strait Islander Health Workers and Aboriginal and Torres Strait Islander Health Practitioners.

(b) The HHS will comply with the state-wide vocational medical training pathways.

(c) The HHS will support profession specific and inter-profession statewide allied health clinical education programs.

(d) The HHS will continue to implement and retain the following positions provided through clauses to the *Health Practitioners and Dental Officers (Queensland Health) Certified Agreement (No 3)*:

- (i) health practitioner research positions provided through the Research Package for Health Practitioners; and
- (ii) clinical educator positions provided through the Clinical Education Management Initiative for Health Practitioners.

(e) The HHS will maintain or increase its contribution of staff to the Queensland Country Relieving doctors program and the receiving HHS will be responsible for wages, clinical governance and appropriate supervision of the junior medical relievers.

3.2 Statewide training, education and research

This clause does not apply to this HHS.

3.3 **Health and medical research**

The HHS will:

(a) develop and implement a strategy to drive increased research activity and its translation into clinical practice;

(b) support increased and equitable access to clinical trials for patients;

(c) support clinicians to undertake research linked to their practice; and

(d) ensure high quality and timely research governance approval processes.

4. Delivery of purchased activity

4.1 The Department and the HHS will monitor actual activity against purchased levels and will take action as necessary to ensure delivery of purchased levels is achieved. The HHS has a responsibility to only recognise revenue that is linked with actual activity delivered against purchased volumes.

4.2 The HHS will actively monitor variances from purchased activity levels and notify the Department as soon as the HHS becomes aware of significant variances.

4.3 The HHS will also notify the Department of deliberate changes to the consistent recording of activity within year that would result in additional activity being recorded for existing health services.

4.4 If the HHS wishes to convert activity between purchased activity types, programs and levels the HHS must negotiate this with the Department based on a sound needs based rationale.

4.5 The Department will reconcile in-scope activity, as defined in the Activity Reconciliation specification sheet (available online, as detailed in Appendix 1), delivered by the HHS against the purchased in-scope activity targets outlined in Table 4.

4.6 Activity reconciliation will be undertaken in February (for the July to December period) and August (for the January to June period) each year and will be derived through application of the methodology which is documented in the Activity Reconciliation specification sheet.

4.7 Should the HHS be unable to deliver the activity that has been funded a financial adjustment will be applied.

4.8 Under delivery of in-scope activity, as defined in the Activity Reconciliation specification sheet, will be withdrawn from the HHS at 100% of the Queensland Efficient Price (QEP).

4.9 Funding and corresponding activity that is withdrawn from the HHS may be reallocated to an alternate provider that can undertake the activity.

4.10 Any resulting changes to funding and purchased activity levels will be actioned through the processes outlined in Schedules 2 and 4 of this service agreement.

4.11 The HHS will maintain its efforts to accurately record the delivered activity and submit this data to the Department in accordance with the agreed requirements.

5. Delivery of purchased services

5.1 As part of the service agreement, the Department purchases a range of services (or deliverables) from the HHS for the delivery of certain programs and projects.

5.2 These program or project services may be the subject of separately agreed conditions tied to that funding and the focus of detailed monitoring by the Department.

5.3 Conditions may include, but are not limited to:

(a) establishment and/or commencement of services;

(b) delivery of activity;

(c) workforce obligations;

(d) establishment of oversight committees;

(e) opening or upgrades to facilities;

(f) program evaluation;

(g) program management;

(h) reporting or notification obligations; and

(i) attainment of performance standards.

5.4 The HHS will ensure that the conditions are achieved within the stipulated time period.

5.5 The HHS will notify the Department if the HHS forecasts an inability to achieve program or project objectives or the conditions.

5.6 The Department may withdraw allocated funding pro rata to the level of under delivery if the services for a specified program or project are not being fully delivered according to the objectives or conditions.

5.7 Any resulting changes to funding and purchased activity levels will be actioned through the processes outlined in Schedules 2 and 4 of this service agreement.

6. Financial adjustments

6.1 **Activity targets**

(a) The Department will initiate a joint process with the HHS to determine whether a financial adjustment should be applied in relation to any purchased activity which has breached the thresholds identified bi-annually. This process will take into account any relevant matters that have been identified in a review/analysis of the breach as well as the outcomes of the activity plan implemented to address the activity breach.

(b) Activity will be monitored at the purchasing hierarchy level. Providing the HHS meets all relevant KPIs and specific funding allocations, the HHS has the ability to negotiate the transfer of activity across the purchasing hierarchy with the Department.

(c) Table 2 demonstrates the financial adjustment that will be applied when activity thresholds have been breached.

(d) The HHS may not utilise the provisions within AASB15 *Revenue from Contracts with Customers* to override the application of any financial adjustment made by the Department in line with Table 2.

Table 2 Financial adjustments applied on breach of activity thresholds

Example of Breach	Description	Financial Adjustment
Over performance	Activity exceeds that specified in the service agreement value for in-scope activity as shown in Table 4.	Purchasing contracts are capped and an HHS will not be paid for additional activity with the exception of activity that is in scope for the identified purchasing incentives as set out in Table 3.
Under performance	Activity is below that specified for in-scope activity as shown in Table 4.	Purchased activity and the related funding will be withdrawn at 100% of the Queensland Efficient Price and reallocated to an alternate provider that can undertake the activity.<br>Refer to Table 4 for the HHS QWAU target.
Failure to deliver on service commitments linked to specific funding allocations	Specific funding allocations<br>National Partnership Agreements.	It is at the discretion of the Department to withdraw allocated funding pro rata to the level of under delivery.
For all other types of activity variance, any financial adjustment will be made at the discretion of the Department.		

6.2 National Partnership on COVID-19 Response

(a) The Department will provide additional funding to the HHS under the *National Partnership on COVID-19 Response* where actual additional cost is demonstrated.

(b) Funding will be provided where the HHS has:

(i) undertaken activity that is in-scope for the State Public Health Payment, as defined under the *National Partnership on COVID-19 Response*, during the reporting period; and/or

(ii) undertaken activity that is in-scope for the Hospital Services Payment, as defined under the *National Partnership on COVID-19 Response*, during the reporting period; and

(iii) met the reporting requirements in relation to COVID-19 expenditure, as prescribed under the *National Partnership on COVID-19 Response* and by the Department.

(c) Additional costs that are reimbursed through the State Public Health Payment and the Hospital Services Payment will be excluded from the calculation of activity eligible for funding under the terms of the *National Health Reform Agreement*.

(d) There is no guarantee that the HHS will have access to additional funding under the State Public Health Payment or the Hospital Services Payment.

(e) All funding that is provided through the State Public Health Payment and the Hospital Services Payment will be subject to a reconciliation to be undertaken by the Administrator of the National Health Funding Pool. The HHS will be required to provide evidence to support claims for additional costs. If the HHS is unable to provide the required evidence with their expenditure claim, funding received may be recalled subject to reconciliation.

(f) Funding adjustments will be actioned through the processes outlined in Schedules 2 and 4 of this service agreement.

6.3 **Purchasing incentives**

(a) The purchasing approach includes a range of funding adjustments which aim to incentivise high quality and high priority activity, support innovation and evidence-based practice, deliver additional capacity through clinically and cost effective models of care and dis-incentivise care which provides insufficient or no benefit for patients. This includes incentive payments for HHSs who achieve targets in specific priority areas. The purchasing incentives are detailed in Table 3. The Department must reconcile the applicable purchasing incentives in Table 3 in line with the timeframes specified in the relevant purchasing specification sheet. The Department must provide a copy of the reconciliation statement to the HHS.

(b) Funding adjustments must be based on the requirements contained in the relevant purchasing specification sheet for that purchasing incentive.

(c) Any resulting changes to funding and purchased activity levels will be actioned through the processes outlined in Schedules 2 and 4 of this service agreement.

Table 3 Purchasing Incentives 2022/23

Incentive	
**Quality Improvement Payment (QIP)**	
Antenatal care for First Nations Women	Payments for achieving two Closing the Gap targets for First Nations women:<br>• to attend five or more antenatal visits with their first antenatal first taking place in the first trimester; and<br>• to stop smoking by 20 weeks gestation.
**Purchasing incentives**	
Virtual care incentive	Incentive funding to increase the number of specialist outpatient services which are provided in virtual settings.
Own source revenue growth	Incentivise the recognition of own source revenue through matching growth in own source revenue with public activity growth funding.
**ABF model localisations**	
Sentinel events	Payment withdrawn for sentinel events as per the national ABF model.
Advance Care Planning (ACP)	QWAUs for HHSs who offer ACP discussions to admitted patients, non-admitted outpatients, community health patients and Emergency Department patients.
Emergency Department Did Not Wait (DNW)	Zero QWAUs for DNWs.
Fractured neck of femur	QWAUs reduced by 20% for non-timely surgical treatment of fractured neck of femur.
Hospital in The Home (HITH)	QWAUs increased for Hospital in the Home (HITH) admissions of Residential Aged Care Facility residents.
Out-of-scope services	Nil QWAUs for out-of-scope procedures.
Pre-operative bed days	QWAUs for long stay days above the upper trim point less ICU days reduced equivalent to pre-operative days, up to a maximum of 3 days, for elective episodes.
Smoking cessation (community mental health)	QWAUs for smoking cessation activity for community mental health patients.

Incentive	
Smoking cessation (inpatients)	QWAUs for smoking cessation activity for publicly funded inpatients.
Stroke care	10% QWAU loading for acute stroke patients admitted to Statewide Stroke Clinical Network-endorsed stroke unit care.
Telehealth (admitted patients)	QWAUs for provider-end of in-scope admitted patient telehealth activity.
**Commissioning mechanisms**	
High-cost home support	Funding for approved individuals requiring 24-hour home ventilation.
Patient flow initiative	Provision of non-recurrent WAU-backed funding to participating HHS who successfully implement agreed recommendations.
Rapid access clinics	Recurrent WAU-backed funding to support the implementation of rapid access clinics to reduce pressure on emergency departments.
Expansion of sub-acute and long stay care	Additional funding to increase the availability of and access to care for sub-acute and long stay patients, thereby improving access to care in a range of settings and releasing capacity within acute facilities.
Connected Community Pathways	Funding to incentivise evidence-based and innovative models of care which promote the delivery of care outside acute facilities and support shared-care partnership arrangements.

6.4 Surgery Connect reimbursements

(a) The HHS will reimburse the Department for the actual costs of activity for nominated patient referrals to the Surgery Connect program where:

(i) The HHS has nominated the patient referral as HHS funded on entry of the referral in the Surgery Connect Activity Navigator (SCAN); and

(ii) The HHS Chief Finance Officer has recorded approval of the nomination in SCAN;

or

(iii) The HHS has obtained the Department's agreement to retrospectively convert a defined patient cohort to HHS funded status in SCAN.

(b) The HHS may only request retrospective conversion of activity to HHS funded within the financial year in which the activity has taken place.

(c) Any resulting changes to funding and purchased activity levels will be actioned through the processes outlined in Schedules 2 and 4 of this service agreement.

6.5 Financial adjustments – other

(a) Notwithstanding the provisions regarding the recognition of revenue as stipulated in AASB1058 *Income of Not-for-Profit Entities* and/or AASB15 *Revenue from Contracts with Customers*, the Department may seek to recover funding from an HHS that was provided through this service agreement which has:

(i) not been utilised in accordance with its intended purpose; and/or

(ii) not been utilised within the prescribed time period to deliver the agreed

outcomes/services.

(b) If the Chief Executive (or delegate) determines that previously allocated funding is to be recovered, any resulting changes to funding and purchased activity levels will be actioned through the processes outlined in Schedules 2 and 4 of this service agreement.

6.6 **Public and private activity/own source revenue**

(a) Own source revenue comprises grants and contributions, user charges and other revenues.

(b) Where an HHS is above its own source revenue target, it will be able to retain the additional own source revenue with no compensating adjustments to funding from other sources where the additional revenue is not attributable to a private patient consistent with clauses A9 to A13 and A44 of the *National Health Reform Agreement.*

(c) Where an HHS is below its own source revenue target in respect of private patients, it will experience a reduction in revenue with no compensating adjustments to funding from other sources.

(d) The HHS will make all efforts to identify and maximise revenue from appropriate third parties to ensure optimisation of available State and Commonwealth funding across the Queensland public sector health system.

(e) The own source revenue identified in Table 4 is an estimate generated by the HHS which allows all third-party funding sources associated with service delivery to be identified. The HHS will ensure that this estimate is substantiated and accurate to ensure no significant variances to actual revenue generated.

(f) The HHS will routinely revise and update the estimate to ensure alignment between the service agreement and Queensland Treasury's reporting system (TRIDATA).

(g) Budget adjustments for changes in own source revenue from private patients will be actioned through the processes set out in Schedule 4 of this service agreement.

7. Funding sources

7.1 The four main funding sources contributing to the HHS service agreement value are:

(a) Commonwealth funding;

(b) State funding;

(c) grants and contributions; and

(d) own source revenue.

7.2 Table 4 provides a summary of the funding sources for the HHS and the total value of the service agreement.

8. Funds disbursement

8.1 The Department agrees to pay the HHS the amount described in Table 4 of Schedule 2, subject to:

(a) parliamentary appropriation and adequate funds being allocated to the Department; and

(b) the terms of this service agreement.

8.2 All payments under this service agreement will be made in accordance with the requirements of the *National Health Reform Agreement* and the Act.

8.3 The Chief Executive will direct the disbursement of both State and Commonwealth funding from the State Pool Account, the State Managed Fund and the Department of Health Expenditure account to the HHS.

8.4 However, the State (represented by the Chief Executive) will not:

(a) redirect Commonwealth payments between HHSs;

(b) redirect Commonwealth payments between funding streams (e.g., from ABF to block funding); and/or

(c) adjust the payment calculations underpinning the Commonwealth's funding.

8.5 The Department will pay state-funded activity-based funding, block funding and system manager funding to the HHS on a fortnightly basis in line with receipt of the State Appropriation Payment. The payment made will be equal to 1/26th of the state-funded component of the service agreement value described in Table 4.

8.6 The Department will pay Commonwealth-funded activity-based funding and block funding on a monthly basis in line with receipt of the Commonwealth payment. The payment made will be equal to 1/12th of the Commonwealth funded component of the service agreement value described in Table 4.

8.7 Where the parties have agreed to amend the service agreement value or the Commonwealth contribution amount changes, the fortnightly and/or monthly payments will be adjusted to reflect the amended service agreement value.

8.8 Service agreement payments may be made outside of these timeframes with the approval of the Department's Chief Finance Officer.

Table 4 North West HHS Total Funding Allocation by Funding Source 2022/23

Funding Source	NWAU (N2122)	QWAU (Q24)	Value ($)
**NHRA Funding**			
**ABF Pool**			
ABF Funding (in scope NHRA)[2]			
Commonwealth[2]	18,050		$43,660,414
State		18,034	$54,280,789
State Specified Grants			$3,380,823
State-wide Services			$0
**State Managed Fund**			
Block Funding			
Small Rural Hospitals		3,944	$21,701,505
Teaching, Training & Research			$3,444,084
Non-Admitted Child & Youth Mental Health			$372,828
Non-Admitted Home Ventilation			$0
Non-Admitted Mental Health			$3,056,191
Other Non-Admitted Service			$0
Highly Specialised Therapies			$0
**Total NHRA Funding**	**18,050**	**21,978**	**$129,896,634**
**Out of Scope NHRA**			
**Queensland ABF Model**			
DVA		61	$138,148
NIISQ/MAIC		92	$316,559
Oral Health		636	$2,563,460
BreastScreen		0	$0
**Total Queensland ABF Funding**	**-**	**789**	**$3,018,167**
**Discretely Funded Programs[3]**			
Department of Health			$64,157,454
Locally receipted funds			$0
**Total Discretely Funded Programs**	**-**	**-**	**$64,157,454**
**Own Source Revenue**			
Private Patient Admitted Revenue[4]	*66*	73	$367,851
Non-Admitted Services		75	$186,132
Pharmaceuticals Benefits Scheme		493	$3,439,610
Other Activities[5]		287	$4,265,424
**Total Own Source Revenue**	**-**	**928**	**$8,259,017**

[2] The split between Commonwealth and State NHRA funding will change during the year as the purchased activity targets are updated and the National Health Funding Board updates their payment advice accordingly.

[3] Includes all other (non-ABF) State and Commonwealth funded health services including, but not limited to, Prisoner Health, Public Health and Prevention Services.

[4] The estimated value of the revenue earned from private patients, based on OSR estimates provided by the HHS.

[5] Incorporates all OSR which is not identified elsewhere in Table 4.

Funding Source	NWAU (N2122)	QWAU (Q24)	Value ($)
Locally Receipted Funds (exc. Discretely Funded Programs)[6]			$3,662,893
Depreciation			$11,159,000
**NPA COVID-19 Response**			
Hospital Services Payment			$0
State Public Health Payment			$0
COVID-19 Vaccine Payment			$0
**Total NPA COVID-19 Response Funding**	**-**	**-**	**$0**
**GRAND TOTAL**	**18,050**	**23,695**	**$220,153,165**

**Pool Accounts**			
ABF Pool (National Health Funding Pool)[7]			$104,340,193
State Managed Fund[8]			$28,574,609
System Manager			$64,157,454

[6] Includes items such as training programs and donations. Does not include locally receipted funds associated with discretely funded programs, e.g.Transition Care.

[7] Articulates the financial payment made to support in-scope ABF services under the NHRA including DVA, NIISQ, MAIC and Breastscreen Services. Applies to all HHSs except Central West HHS and Torres and Cape HHS.

[8] Articulates the payment made for block funded services under the NHRA, DVA, NIISQ and MAIC services.

Table 5 National Health Reform Funding

NHRA Funding Type	NWAU (N2122)	Commonwealth ($)	State ($)	Other State funding[9] DVA/MAIC/Oral Health/BreastScreen ($)	Total ($)
**National Efficient Price (NEP)**					**$5,597**
**ABF Allocation (NWAU)**					
Emergency Department	4,329	$10,471,391	$13,829,398	$307,085	$24,607,874
Acute Admitted	10,136	$24,517,182	$32,379,451	$0	$56,896,633
Admitted Mental Health	246	$595,982	$787,104	$0	$1,383,086
Sub-Acute	1,258	$3,042,042	$4,017,576	$0	$7,059,618
Non-Admitted	2,081	$5,033,817	$6,648,082	$2,711,082	$14,392,982
**Total ABF Pool Allocation**	**18,050**	**$43,660,414**	**$57,661,612**	**$3,018,167**	**$104,340,193**
**Block Allocation**					
Teaching Training and Research	-	$710,132	$2,733,952	-	$3,444,084
Small and Rural Hospitals[10]	-	$7,829,972	$13,871,533	-	$21,701,505
Non-Admitted Mental Health	-	$1,163,801	$1,892,390	-	$3,056,191
Non-Admitted Child & Youth Mental Health	-	$38,033	$334,795	-	$372,828
Non-Admitted Home Ventilation	-	$0	$0	-	$0
Other Non-Admitted Services	-	$0	$0	-	$0
Other Public Hospital Programs	-	$0	$0	-	$0
Highly Specialised Therapies	-	$0	$0	-	$0
**Total Block Allocation**	**-**	**$9,741,939**	**$18,832,670**	**-**	**$28,574,609**
**Grand Total Funding Allocation**					**$132,914,802**

[9] State funding transacted through the Pool Account; not covered under the NHRA

[10] Incorporating small regional and rural public hospitals, five specialist mental health facilities (Baillie Henderson Hospital, Jacaranda Place – Queensland Adolescent Extended Treatment Centre, The Park – Centre for Mental Health, Kirwan Rehabilitation Unit and Charters Towers Rehabilitation Unit) and the Ellen Barron Family Centre.

Table 6 Discretely Funded Programs (Non-ABF)

Discretely Funded Programs	$
Aged Care Assessment Program	$227,751
Alcohol, Tobacco and Other Drugs	$4,079,886
Community Health Programs	$10,842,261
Disability Residential Aged Care Services	$0
Home and Community Care Program (HACC)	$0
Interstate Patients (QLD residents)	$184,039
Multi-purpose Health Services	$1,317,843
Other State Funding	$33,616,406
Patient transport	$12,828,842
Prevention Services and Public Health	$1,060,425
Prisoner Health Services	$0
Research	$0
Transition Care	$0
Residential Aged Care Services	$0
**TOTAL**	**$64,157,454**

Schedule 3 Performance Measures

1. Performance Measures

1.1 The performance of the HHS will be measured according to the assessment criteria and processes described in the Performance and Accountability Framework.

1.2 The detailed specification for each of the performance measures listed in this service agreement are provided through performance measure attribute sheets.

1.3 The performance measures identified in this service agreement are applicable to the HHS unless otherwise specified within the attribute sheet.

1.4 HHSs are also required to report against the agreed key performance measures in their Health Equity Strategy.

Table 7 HHS Performance Measures – Key Performance Indicators

Key Performance Indicators
Hospital Acquired Complications
Elective surgery patients waiting longer than the clinically recommended timeframe
Gastrointestinal endoscopy patients waiting longer than the clinically recommended timeframe
Access to oral health services (adults)
Access to oral health services (children)
The percentage of oral health activity which is preventive
Proportion of diabetic patients with a current HbA1c result
Patients whose smoking status has been recorded
Potentially Preventable Hospitalisations – First Nations peoples:<br>• Diabetes complications<br>• Selected conditions
Potentially Preventable Hospitalisations (non-diabetes complications)
Telehealth utilisation rates:<br>• Number of non-admitted telehealth service events
% of low birthweight babies born to Queensland mothers
Forecast operating position:<br>• Full year<br>• Year to date
Average sustainable Queensland Health FTE
Capital expenditure performance
Care Closer to Home:<br>• *Self-sufficiency: admitted patients*<br>• *Self-sufficiency: non-admitted patients*
Participation in the Practice Incentive Program
Proportion of mental health and alcohol and other drug service episodes with a documented care plan

Table 8 HHS Performance Measures - Safety and Quality Markers

Safety and Quality Markers
Sentinel Events
Hospital Standardised Mortality Ratio
Severity Assessment Code (SAC) analysis completion rates
Patient Reported Experience

Table 9 HHS Performance Measures – Outcome Indicators

Outcome Indicators
Access to emergency dental care
First Nations peoples representation in the workforce
General oral health care for First Nations peoples
Complaints resolved within 35 calendar days
Advance care planning
Smoking cessation clinical pathway
Adolescent vaccinations administered via the statewide School Immunisation Program
Integrated care pathways:<br>• Care pathway in place for patients with identified co-morbidities

Schedule 4 Amendments to this service agreement

1. Agreed process to amend this service agreement

1.1 The parties acknowledge that this service agreement is subject to amendment, which will generally occur through:

(a) amendment windows;

(b) extraordinary amendment;

(c) periodic adjustments; and

(d) end of financial year reconciliation.

1.2 **Amendment windows**

(a) There will be set periods of time nominated by the Department during the year (amendment windows) in which a party may propose an amendment and the parties will endeavour to negotiate and finalise proposals to amend this service agreement.

(b) Amendment proposals that are agreed will be documented in a deed of amendment to this service agreement.

(c) Further details on the amendment window process, including the timing of amendment windows, is provided online, as detailed in Appendix 1.

1.3 **Extraordinary Amendment**

(a) Outside an amendment window, the Department and the HHS agree to limit any proposal to amend the terms of this service agreement to those where there is an urgent priority need to facilitate a funding allocation (extraordinary amendment). The parties will endeavour to negotiate and finalise any such proposal urgently.

(b) The process for submitting, negotiating and resolving an extraordinary amendment is available online, as detailed at Appendix 1.

(c) Agreed extraordinary amendments will be reflected in a notice issued by the Chief Executive countersigned as accepted by the HHS, which notice will be replaced when the extraordinary amendment is subsequently formalised in a deed of amendment issued following the next amendment window.

1.4 **Periodic adjustments**

(a) The service agreement value (and corresponding purchased activity) may be adjusted at any time to reflect funding variations that:

(i) occur on a periodic basis or in line with adjustments permitted for specific funding allocations;

(ii) are referenced in the service agreement; and

(iii) are based on a clearly articulated formula, an agreed basis or such other reasonably substantiated basis tied to performance.

(periodic adjustment).

(b) Periodic adjustments will be reflected in an adjustment notice issued by the Chief Executive (or delegate) to the HHS, based on relevant data, and subsequently formalised in a deed of amendment issued following the next amendment window.

1.5 **End of financial year reconciliation**

(a) There will be an end of financial year reconciliation process, with the scope defined by the Department and informed by Queensland Government Central Agency requirements.

(b) The Department will provide the HHS with a reconciliation of all service agreement funding and purchased activity for the prior financial year. This will reflect the position following conclusion of the end of financial year adjustments process.

(c) The impact of end of financial year adjustments on subsequent year funding and activity will be incorporated in the service agreement through the deed of amendment executed following the next available amendment window.

(d) This clause will survive expiration of this service agreement.

Appendix 1 Reference Documents

Hospital and Health Boards Act 2011

National Health Reform Agreement (NHRA) 2020-25

System Outlook to 2026 - for a sustainable health service

Queensland Health Performance and Accountability Framework

My health, Queensland's future: Advancing health 2026

Department of Health Strategic Plan 2021-2025

Local Area Needs Assessment (LANA) Framework

Making Tracks Together: Queensland's Aboriginal and Torres Strait Islander Health Equity Framework

Making Tracks toward closing the gap in health outcomes for Indigenous Queenslanders by 2033 - Policy and Accountability Framework

Aboriginal and Torres Strait Islander Health Workforce Strategic Framework 2016-2026

National Agreement on Closing the Gap

Queensland Health Workforce Diversity and Inclusion Strategy 2017 to 2022

Performance Measures Attribute Sheets

Purchasing Initiatives and Funding Specifications

Public Health Practice Manual

National Partnership on COVID-19 Response

Statewide services reference material

Service agreement amendment processes

Data supply requirements

Contracting and Performance Management Branch
GPO Box 48
Brisbane QLD 4001 Australia

Tel (+61) (07) 3708 5869

www.health.qld.gov.au

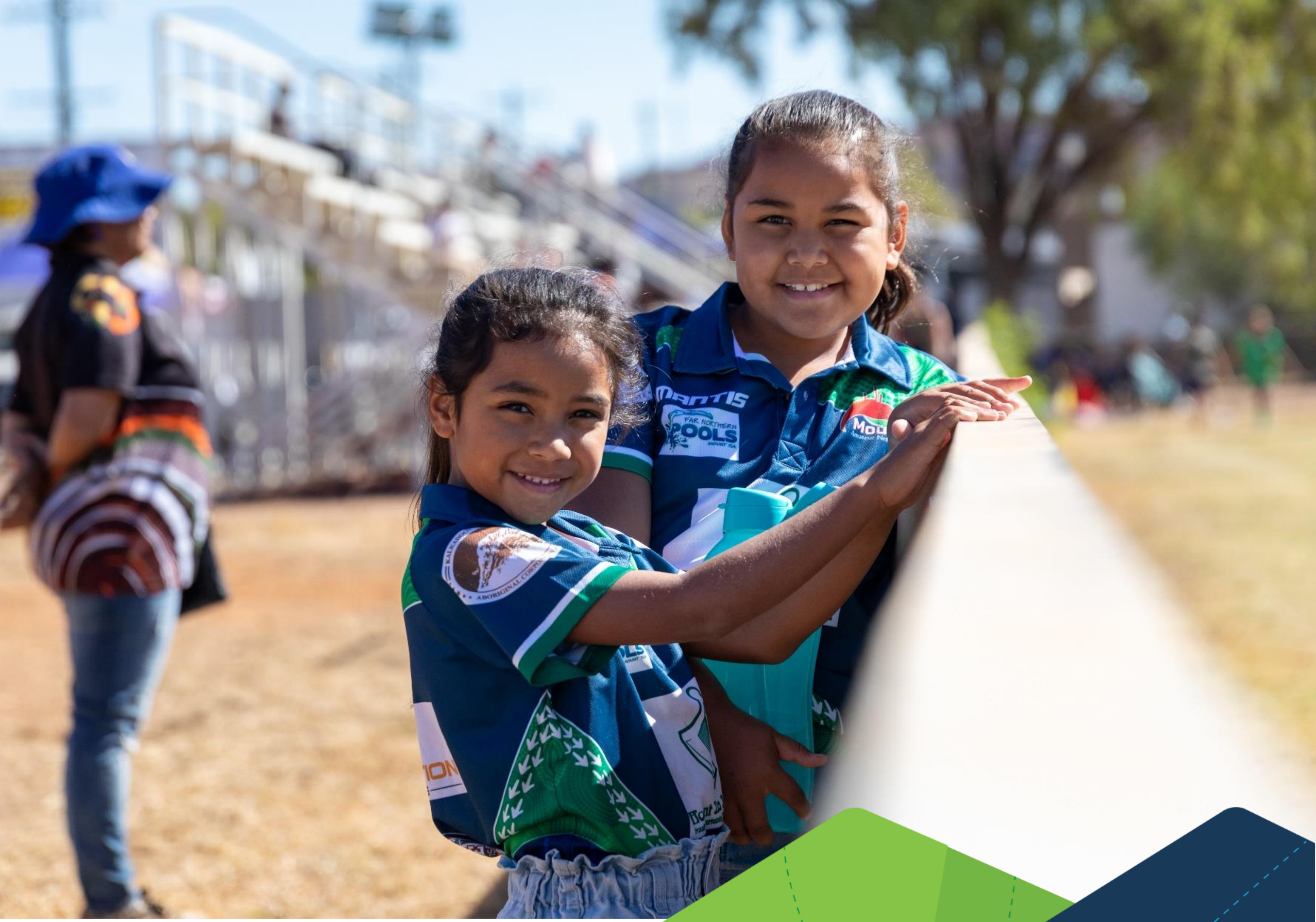

Queensland Health

Service Agreement 2022/23 – 2024/25

North West Hospital and Health Service

December 2024 Revision

Queensland Government

North West Hospital and Health Service, Service Agreement 2022/23 - 2024/25

Published by the State of Queensland, (Queensland Health), December 2024

This document is licensed under a Creative Commons Attribution 3.0 Australia licence. To view a copy of this licence, visit creativecommons.org/licenses/by/3.0/au

© State of Queensland (Queensland Health) **2024**

You are free to copy, communicate and adapt the work, as long as you attribute the State of Queensland (Queensland Health).

For more information contact:

Contracting and Performance Management Branch, Department of Health, GPO Box 48, Brisbane QLD 4001
Tel (+61) (07) 3708 5869

Email SAM@health.qld.gov.au

An electronic version of this document is available at www.health.qld.gov.au/system-governance/health-system/managing/default.asp

Disclaimer:
The content presented in this publication is distributed by the Queensland Government as an information source only. The State of Queensland makes no statements, representations or warranties about the accuracy, completeness or reliability of any information contained in this publication. The State of Queensland disclaims all responsibility and all liability (including without limitation for liability in negligence) for all expenses, losses, damages and costs you might incur as a result of the information being inaccurate or incomplete in any way, and for any reason reliance was placed on such information.

Acknowledgement

We acknowledge the Traditional and Cultural Custodians of the lands, waters, and seas across Queensland, pay our respects to Elders past and present, and recognise the role of current and emerging leaders in shaping a better health system.

We recognise the First Nations peoples in Queensland are both Aboriginal Peoples and Torres Strait Islander Peoples, and support the cultural knowledge, determination, and commitment of Aboriginal and Torres Strait Islander communities in caring for health and wellbeing for millennia.

North West Hospital and Health Service is committed to reducing inequalities between Indigenous and non-Indigenous health outcomes in line with the Australian Government's Closing the Gap initiative.

North West Hospital and Health Service is proud to recognise and celebrate the cultural diversity of our communities and workforce at the following locations:

**Location**	**Traditional Custodians**
Burketown	Gangalidda and Garawa
Camooweal	Indjalandji / Dhidhnu
Cloncurry	Mitakoodi
Dajarra	Waluwarra and Yulluna
Doomadgee	Gangalidda and Waanyi
Mornington Island	Lardil (other historical groups from surrounding islands include Yangkal and Kaiadilt
Normanton	Kurtijar, Gkuthaarn, Kukatj
Mount Isa	Kalkadoon

Contents

Acknowledgement 3

Contents 4

Tables 5

- **1.** Introduction 6
- **2.** Scope 6
- **3.** Services 6
- **4.** Performance and Accountability Framework 7
- **5.** Outcomes Framework 7
- **6.** Data supply requirements 8
- **7.** Hospital and Health Service accountabilities 8
- **8.** Department accountabilities 10
- **9.** Achieving health equity with First Nations Queenslanders 10
- **10.** Dispute Resolution 11
- **11.** General 11
- **12.** Counterparts 12

Execution 13

Schedule 1 HHS profile 14

- **1.** HHS profile 14

Schedule 2 Funding and purchased activity and services 15

- **1.** Introduction 15
- **2.** Purchased health services 16
- **3.** Teaching, training, and research 18
- **4.** Delivery of purchased activity 19
- **5.** Delivery of purchased services 20
- **6.** Financial adjustments 21
- **7.** Funding sources 25
- **8.** Funds disbursement 25

Schedule 3 Department of Health Provided Services 32

- **1.** In scope services and service schedules 32

Schedule 4 Performance Measures 33

- **1.** Performance Measures 33

Schedule 5 Amendments to this service agreement 37

- **1.** Agreed process to amend this service agreement 37

Appendix 1 Reference Documents 39

Tables

Table 1 Statewide Services....16
Table 2 Financial adjustments applied on breach of activity thresholds....22
Table 3 Funding adjustments 2024/25....22
Table 4 HHS Funding by Outcomes Framework Care Domain....30
Table 5 HHS Total Funding Allocation by Funding Source 2024/25....30
Table 6 National Health Reform Funding....29
Table 7 Discretely Funded Programs (Non-ABF)....31
Table 8 Department of Health Provided Services and Service Schedules....34
Table 9 HHS Performance Measures – Key Performance Indicators....35
Table 10 HHS Performance Measures – Safety and Quality Markers....34
Table 11 HHS Performance Measures – Outcome Indicators....35
Table 12 Statewide Health Equity Key Performance Measures....35

1. Introduction

1.1 In performing this service agreement, the Department and the Hospital and Health Service (HHS) will act consistent with the object of the *Hospital and Health Boards Act 2011* (Qld) (Act).

1.2 The Department and the HHS operate as part of a networked system and agree to work collaboratively with each other, with other HHSs and with the Queensland Ambulance Service in the best interests of the Queensland public sector health system.

1.3 The parties will ensure that planning and delivery of health services is consistent with the strategies and priorities set out by government. The parties recognise the importance of the HHS' Health Equity Strategy (as defined in the Act) and the parties' commitment to improving health and wellbeing outcomes and achieving health equity for Aboriginal and Torres Strait Islander peoples.

2. Scope

2.1 This service agreement covers the period from 1 July 2022 to 30 June 2025.

2.2 The parties have identified the services to be provided by the HHS, the funding for the provision of those services, the performance measures applicable to the services and data requirements.

3. Services

3.1 In delivering services, the HHS is required to meet:

(a) the applicable conditions of all national agreements and national partnership agreements between the Queensland Government and the Commonwealth Government and commitments under any related implementation plans; and

(b) the applicable conditions of each agreement or arrangement for funding between the Department and the HHS and commitments under any related implementation plans, which conditions and commitments may be recorded separately to this service agreement but for which funding is (or becomes) provided in Schedule 2.

3.2 The HHS is required to deliver the services outlined in this service agreement for which funding is provided in Schedule 2.

3.3 Where issues arise which prevent the HHS from providing a service or necessitate a reduction in the level or scope of a service provided, prompt notification must be made to the Department and impacted HHSs, with appropriate details. The HHS must minimise any clinical risk or adverse impact to patient experience that may result from service disruption. The Department will respond to the HHS on any requirements it has concerning service delivery and any adjustments triggered by under delivery.

3.4 If the HHS wishes to terminate or reduce service levels for a service for which funding is provided in Schedule 2, this will remain subject to negotiation and agreement by the Department at its discretion.

3.5 For any new services proposed during the term of this service agreement, the parties agree:

(a) the service must meet a demonstrated clinical need and provide value for money;

(b) the commencement of a new service, including the implementation of new models of care, may occur where the service has been commissioned by the Department, a funding stream is in place and any conditions relating to the funding have already been agreed; and

(c) if the HHS wishes to commence providing a new service that has not been commissioned by the Department, this will remain subject to agreement by the Department at its discretion.

3.6 It is acknowledged that there may, from time to time, need to be service delivery changes between HHSs. Management of inter-HHS relationships should be informed by the following principles:

(a) HHSs should maintain the proportion of out of HHS work undertaken unless as a result of agreed repatriation of patients;

(b) each HHS should manage patients from its own catchment population if it is within its clinical capability to do so as specified by the Clinical Services Capability Framework; and

(c) where it is proposed that a service move from one HHS to another, the Department will consider, as part of its review under clauses 3.4 and 3.5, whether the respective Health Service Chief Executives endorse the proposed change in patient flows; and the funding required to follow the patient.

4. Performance and Accountability Framework

4.1 The Queensland Health Performance and Accountability Framework (the Performance and Accountability Framework) sets out the framework within which the Department monitors and manages the performance of public sector health services in Queensland.

4.2 The parties will act consistent with the Performance and Accountability Framework.

5. Outcomes Framework

5.1 Queensland Health is embarking on a strategic shift in funding focus from “volume” to “outcome” using the Outcomes Framework. This approach aims to link the resources and services required and delivered as part of healthcare activities, to health outcomes for individuals and the population.

5.2 The Outcomes Framework takes a three-tiered approach:

(a) The System Tier (Tier 1), which acts as a strategic tier, and includes four domains to measure the contribution of Queensland Health to the system outcomes.

(b) The Operational Tier (Tier 2) which includes nine (9) Clinical Care Domains, reflecting areas that are important to deliver change and improvement in the short to medium term, and to operationalise the Outcomes Framework.

(c) The Tactical Tier (Tier 3) provides scaffolding to select initiatives for implementation as specific pressures arise. These pressures may include areas identified for improvement through Tier 2.

5.3 In consultation with the State-wide Clinical Networks the indicators below are under further development and shadowing.

Indicator	Care Domain	Clinical Leadership
Percentage of patients who have HBA1C ordered during hospital admission	Chronic and Complex	Diabetes Network
Time to treatment for breast, colorectal and lung cancers	Cancer Care	Cancer Care Network

5.4 Schedule 4 maps existing indicators in the Performance and Accountability Framework to the care domains of the Outcomes Framework.

6. Data supply requirements

6.1 The HHS will provide the Department with all clinical and non-clinical data that is reasonably required to support the effective management of the public sector health system. This will include, but is not limited to, data that is required to:

(a) fulfil legislative obligations;

(b) deliver accountabilities and obligations to State and Commonwealth Governments; including related to the provision and reconciliation of activity data by the Administrator of the National Health Funding Pool;

(c) monitor and support performance improvement;

(d) manage this service agreement;

(e) support clinical innovation; and

(f) facilitate evaluation and audit.

6.2 The parties agree and acknowledge that:

(a) the Department will keep the HHS informed of the Department's data requirements; and

(b) data will be provided as required, or permitted, by law.

6.3 Further details on data supply requirements, including principles that guide the collection, storage, transfer and disposal of data and prescribed timeframes for data submission, are provided online as detailed in Appendix 1.

7. Hospital and Health Service accountabilities

7.1 The HHS will perform its obligations under this service agreement.

7.2 As applicable to the HHS and its services, the HHS will comply with:

(a) legislation and subordinate legislation, including the Act;

(b) cabinet decisions;

(c) Ministerial directives;

(d) agreements entered into between the Queensland and Commonwealth governments (or agreements with others in furtherance of such agreements), of which it is informed;

(e) agreements entered into between the Department and other Queensland Government entities, of which it is informed;

(f) agreements entered into with another HHS(s), including Networked Services Agreements;

(g) all industrial instruments;

(h) all health service directives and health employment directives; and

(i) all policies, guidelines, and implementation standards, including human resource policies.

7.3 As part of the commitment to achieving First Nations health equity, the HHS will prioritise the elimination of racial discrimination and institutional racism within its service and ensure that Aboriginal and Torres Strait Islander peoples have access to culturally safe and responsive health services.

7.4 The HHS will ensure that effective health service planning and delivery systems are in place, working in collaboration with the Department.

7.5 To support the achievement of the Queensland-Commonwealth Partnership's (QTP's) vision and commitment to work together to tackle health system challenges that cannot be overcome by any one organisation, HHSs are required to prepare and submit Joint Regional Needs Assessments in accordance with the framework provided online as detailed in Appendix 1.

7.6 HHSs must operate clinical service delivery consistent with the National Quality and Safety Standards. The HHS is expected to escalate any concerns that arise at the conclusion of a formalised assessment.

7.7 The HHS will ensure that health service employees employed by the Chief Executive[1] who perform work for the HHS are managed in accordance with any applicable delegations and directions from the Chief Executive. The HHS will ensure that effective asset management systems are in place (available online, as detailed in Appendix 1), that comply with the *Queensland Government Building Policy Framework and Guideline*, while working in collaboration with the Department.

7.8 The HHS will maintain accreditation to the standards required by the Department.

7.9 The HHS will appropriately perform and fulfil its functions under the Act.

[1] In this service agreement the term Chief Executive takes the meaning applied in the Act, which is the Chief Executive of Queensland Health, but which is generally referred to as the Director-General.

7.10 The HHS will provide to the Chief Executive reports of a type, and at the intervals, agreed between the parties, or as reasonably specified by the Chief Executive.

8. Department accountabilities

8.1 The Department will perform its obligations under this service agreement including, in return for the HHS performing its obligations and delivering the services, providing funding to the HHS as stipulated in this service agreement (as amended).

8.2 The Department will:

(a) comply with applicable legislation and subordinate legislation, including the Act, as relates to this service agreement;

(b) perform the system manager role (as defined in the Act) through the Chief Executive; and

(c) provide a range of services to the HHS as set out in Schedule 3.

8.3 The Chief Executive will appoint health service employees to perform work for the HHS for the purpose of enabling the HHS to perform its functions under the Act.

8.4 The Chief Executive will consult, cooperate, and coordinate with the HHS to ensure legal compliance with the *Work Health and Safety Act 2011* and other legislation as it applies to the scope, nature and location of operations associated with this service agreement.

8.5 The Chief Executive will appropriately perform and fulfil their functions under the Act.

9. Achieving health equity with First Nations Queenslanders

9.1 Through legislative amendments to the Act and the Hospital and Health Boards Regulation 2012, and the release of *Making Tracks Together: Queensland's Aboriginal and Torres Strait Islander Health Equity Framework* (2021), Queensland Health has strengthened its commitment to improving health and wellbeing outcomes and achieving health equity with First Nations peoples.

9.2 The HHS will develop and resource a First Nations Health Equity Strategy, compliant with legislative requirements. An implementation plan, accompanying the strategy, demonstrates the HHS's activities and key performance measures to achieve health equity with First Nations peoples. The Health Equity Strategy will act as the principal accountability mechanism between the Aboriginal and Torres Strait Islander community and the HHS in achieving health equity with First Nations Queenslanders and forms a key part of the Queensland Government's commitment to the *National Agreement on Closing the Gap* (2020).

9.3 The HHS is required to review the Health Equity Strategy at least once every three years and will publish the Health Equity Strategy in a way that allows it to be accessed by members of the public.

9.4 The HHS will ensure that commitment and leadership is demonstrated through implementing the actions and achieving the key performance measures outlined in the Health Equity Strategy.

9.5 The HHS will report publicly every year on progress against the Health Equity Strategy.

9.6 The HHS will support the implementation of other supplementary policies and strategies to drive health equity across the public health system, including relevant election commitments.

9.7 The HHS will participate as a partner in the implementation and achievement of Queensland's *HealthQ32 First Nations First Strategy 2032* in addition to HHS commitments within their Health Equity Strategy.

10. Dispute Resolution

10.1 Where a dispute arises in connection to this agreement, either between the department and one or more HHSs or between HHSs, every effort should be made to resolve the dispute at the local level. If local resolution cannot be achieved, the dispute resolution processes, accessible through Appendix 1, must be followed.

11. General

11.1 **Sub-contracting**

(a) The HHS must have appropriate systems in place to ensure that any subcontractor is accredited (as applicable), qualified, and otherwise fit to perform any services for which it is contracted.

(b) The HHS must ensure that any sub-contractor who has access to confidential information (as defined in the Act) or personal information (as defined in the Information Privacy Act 2009 (Qld)) complies with obligations no less onerous than those imposed on the HHS.

11.2 **Insurance**

The HHS must:

(a) hold and maintain the types and levels of insurances that the HHS considers appropriate according to its functions and obligations; and

(b) comply with reasonable requests or directions from the Department in this regard.

11.3 **Amendment**

The process for amending this service agreement is provided for under the Act and further outlined in Schedule 5.

12. Counterparts

12.1 This service agreement may be executed in two or more identical copy counterparts, each of which together will be deemed an original, but all of which together will constitute one and the same instrument.

12.2 In the event that any signature executing this service agreement or any part of this service agreement is delivered by facsimile transmission or by scanned e-mail delivery of a '.pdf' format data file or equivalent of the entire agreement, the signature will create a valid and binding obligation of the party executing (or on whose behalf the signature is executed) with the same force and effect as if the signature page were an original. For the avoidance of doubt, this service agreement may be in the form of an electronic document and may be electronically signed.

12.3 For execution under this clause 12 to be valid, the entire service agreement upon execution by each individual party must be delivered to the remaining parties.

Execution

The terms of this Service Agreement were agreed under the provisions set out in the *Hospital and* Health *Boards Act,* section 35 on 29 June 2022, and were subsequently amended by the Deeds of Amendment entered into pursuant to section 39 of the *Hospital and Health Boards Act 2011* and executed on 22 December 2022, 3 May 2023, 20 July 2023, 15 January 2024, 5 April 2024, 17 July 2024 and 4 December 2024.

This revised Service Agreement consolidates amendments arising from:

- Periodic Adjustment – COVID-19 Funding Transfer September 2022
- Periodic Adjustment – COVID-19 Funding Transfer October 2022
- 2022/23 Amendment Window 2 (in year variation)
- Periodic Adjustment – COVID-19 Funding Transfer December 2022
- 2022/23 Amendment Window 3 (in year variation)
- Periodic Adjustment – COVID-19 Funding Transfer April 2023
- Extraordinary Amendment Window – May 2023
- 2023/24 Amendment Window 1 (Budget Build)
- 2023/24 Amendment Window 2 (in year variation)
- 2023/24 Amendment Window 3 (in year variation)
- Extraordinary Amendment Window – June 2024
- 2024/25 Amendment Window 1 (Budget Build)
- 2024/25 Amendment Window 2 (in year variation)

Schedule 1 HHS profile

1. HHS profile

North West Hospital and Health Service delivers health services to the communities of North West Queensland, serving a resident population of around 30,000 people that significantly increases with visitors and tourists annually, with one regional hospital, two multi-purpose health services, four primary health clinics and five community health centres. Mount Isa Hospital, as the regional hospital, is the primary referral centre within North West HHS.

With one of the highest proportions of Aboriginal and Torres Strait Islander populations in Queensland, North West HHS is committed to improving health outcomes for First Nations people with the Making Tracks program which focuses on chronic diseases, sexual health, discharge against medical advice, and maternal and infant health.

The North West HHS region's population is slowly declining with an increasing proportion of aged community members and an overall lower socio-economic population where high levels of acute and chronic disease are evident. These demographics place an increasing demand on public heath services delivered across a geographical area of 300,000 kilometres stretching across north western Queensland and the Gulf of Carpentaria.

Increasing the use of and available infrastructure for communication technologies is essential for North West HHS to continue to deliver contemporary models of care to the community closer to home.

North West HHS partners with a broad range of organisations and service providers to enhance the services available to communities in the region, including:

- Gidgee Healing, the regional Aboriginal Controlled Health Service for North West Queensland
- Royal Flying Doctor Services
- Western Queensland Primary health Network
- Queensland Ambulance Service and Queensland Police Service
- Seven City, Shire and Aboriginal Shire Councils
- Outreach allied and medical health services including CheckUp, the Deadly Ears and Indigenous Respiratory Outreach Care (IROC) programs
- Centracare, Headspace and other charitable not for profit enterprises
- James Cook University's Centre for Rural and Remote Health.

Schedule 2 Funding and purchased activity and services

This Schedule 2 sets out:

(a) the services which are to be provided by the HHS;

(b) the activity purchased by the Department from the HHS;

(c) the funding provided for delivery of the purchased activity;

(d) the allocation of funding provided against the care domains of the Outcomes Framework;

(e) the criteria and processes for financial adjustments associated with the delivery of purchased activity and specific funding allocations; and

(f) the sources of funding that this service agreement is based on and the way these funds will be provided to the HHS.

1. Introduction

1.1 The HHS will deliver the services for which funding is provided in this Schedule 2. In providing these services, the HHS will ensure that:

(a) all statewide and national policy frameworks, guidelines, protocols and implementation standards applicable to the service provided are followed;

(b) participation in national programs is facilitated and supported, including where these programs are provided by the Commonwealth Government;

(c) service delivery partnerships, including with other HHSs, primary care organisations and non-government organisations, are maintained and operate effectively;

(d) collaboration and engagement with other service providers and stakeholders is initiated and maintained to ensure that an integrated system of treatment, care and support is in place and to facilitate the delivery of comprehensive and effective services. This may include but is not limited to:

(i) other HHSs;

(ii) non-government organisations;

(iii) Aboriginal and Torres Strait Islander community-controlled health organisations;

(iv) Queensland Ambulance Service;

(v) services provided through the Department of Health (for example, Pathology Queensland);

(vi) primary care providers;

(vii) other government departments and agencies; and

(viii) private providers;

(e) models of care and service delivery arrangements are consistent with evidence-based practice and offer value for money;

(f) services are provided on an equitable basis to the community, and processes are in place to ensure that services reach identified target populations, high risk groups and hard to reach communities;

(g) referral networks and pathways continue to operate effectively; and

(h) innovation and continuous improvement are supported.

2. Purchased health services

2.1 Table 4 shows the allocation of funding from the Department to the HHS across the care domains of the Outcomes Framework. Table 5, Table 6, and Table 7 outline the activity and service streams which the Department agrees to purchase from the HHS pursuant to this service agreement.

2.2 More generally, this will include the following:

2.3 **Statewide Services**

(a) The designation of a service as a statewide service (either a clinical statewide service or a clinical support statewide service) will be determined by the Department, consistent with the stipulated governance arrangements for such services.

(b) The HHS will:

(i) collaborate with the Department and other HHSs in the implementation of, and adherence to, the governance and oversight arrangements for statewide services;

(ii) participate in, and contribute to, the staged review of the purchasing model for identified statewide services; and

(iii) ensure that referral pathways in and out of each statewide service are followed.

2.4 **Clinical Statewide Services and Clinical Support Statewide Services provided**

This clause does not apply to this HHS.

Table 1 Statewide Services

This table does not apply to this HHS.

2.5 **Regional services**

This clause does not apply to this HHS.

2.6 Prevention services and public health services

(a) The HHS will provide a range of prevention and public health services to promote and protect health, prevent illness and disease, and manage risk, including:

(i) Specialist Public Health Units

(ii) environmental health services, including risk assessment, regulation and enforcement in relation to environmental hazards, food safety, medicines and therapeutic goods, mosquitos and other vectors, pest management, poisons, radiation safety, chemical safety and water quality;

(iii) communicable disease services including immunisation, blood-borne viruses, sexually transmissible infections, infection control, notifiable conditions, mosquito-borne disease and tuberculosis;

(iv) management of incidents, emergencies and disasters, and disease outbreak readiness and response services;

(v) preventive health services;

(vi) population health screening including, but not limited to, cancer screening services and newborn blood spot screening;

(vii) public health epidemiology and surveillance;

(viii) mitigation and adaptation in response to climate risks.

(b) Services will be provided in line with public health related legislation and the service delivery and reporting requirements outlined in the Public Health Service Schedule and supported by the *Public Health Practice Manual*, as these relate to the services provided.

(c) Delivery of these services may be coordinated through specialist public health units, sexual health services, tuberculosis services, other areas of the HHS, or a combination of these.

2.7 Aboriginal and Torres Strait Islander health services

The HHS will provide Aboriginal and Torres Strait Islander specific health services and initiatives consistent with the principles and objectives of the Queensland Government's *Making Tracks toward closing the gap in health outcomes for Indigenous Queenslanders by 2033 – Policy and Accountability Framework* and the priorities committed to in the HHS's Health Equity Strategy. These services and initiatives will be delivered in line with guidance from the First Nations Health Office and the *First Nations First Strategy 2032.*

2.8 Mental health, alcohol, and other drugs services

The HHS will provide treatment, care and support for individuals who are, or may be, experiencing substance use disorders and/or other mental health disorders, mental health crisis and suicidal distress, and their families and carers. Services will be delivered in line with guidance from the Mental Health, Alcohol and Other Drugs Strategy and Planning Branch.

2.9 **Oral health services**

The HHS will provide oral health services to people who meet the eligibility criteria for accessing public dental services in Queensland. Services will be delivered in line with guidance from the Office of the Chief Dental Officer.

2.10 **Prisoner health services**

This clause does not apply to this HHS.

2.11 **Youth detention services**

This clause does not apply to this HHS.

2.12 **Refugee health**

This clause does not apply to this HHS.

2.13 **State-funded outreach services**

(a) Where state-funded outreach services are provided:

(i) funding for the service will remain part of the providing HHS's funding allocation; and

(ii) the activity must be recorded at the HHS where the outreach service is being provided.

(b) Any changes to the provision of outreach services will follow the requirements set out in clause 3 of this service agreement.

3. Teaching, training, and research

The HHS will provide the teaching, training and research programs for which funding is provided within Schedule 2 and as described below.

3.1 **Clinical education and training**

(a) The HHS will provide education and training placements for the following professional groups consistent with and proportionate to the capacity of the HHS and will support and align with stipulated placement terms governing clinical placements in Queensland Health facilities:

(i) medical students;

(ii) nursing and midwifery students;

(iii) pre-entry clinical allied health students;

(iv) interns;

(v) rural generalist trainees;

(vi) vocational medical trainees;

(vii) first year nurses and midwives;

(viii) re-entry to professional register nursing and midwifery candidates;

(ix) dental students;

(x) allied health rural generalist training positions;

(xi) Aboriginal and Torres Strait Islander Health Workers and Aboriginal and Torres Strait Islander Health Practitioners.

(b) The HHS will comply with the state-wide vocational medical training pathways.

(c) The HHS will support profession specific and inter-profession statewide allied health clinical education programs.

(d) The HHS will continue to implement and retain the following positions provided through clauses to the *Health Practitioners and Dental Officers (Queensland Health) Certified Agreement (No 4) 2022*:

(i) health practitioner research positions provided through the Research Package for Health Practitioners; and

(ii) clinical educator positions provided through the Clinical Education Management Initiative for Health Practitioners.

(e) The HHS will maintain or increase its contribution of staff to the Queensland Country Relieving Doctors and the receiving HHS will be responsible for wages, clinical governance, and appropriate supervision of the junior medical relievers.

3.2 **Statewide training, education, and research**

This clause does not apply to this HHS.

3.3 **Health and medical research**

The HHS will:

(a) develop and implement a strategy to drive increased research activity and its translation into clinical practice;

(b) support increased and equitable access to clinical trials for patients;

(c) support clinicians to undertake research linked to their practice; and

(d) ensure high quality and timely research governance approval processes.

4. Delivery of purchased activity

4.1 The HHS is required to maintain accurate activity forecasts in the purchased target module of the Decision Support System (DSS) at all times. This information is imperative to the Department's assessment of State performance against the national Soft Cap and for outer-year planning. Activity forecasts must accurately reflect financial forecasts reported to the Finance Branch monthly.

4.2 The Department and the HHS will monitor actual activity against purchased levels and will act as necessary to ensure delivery of purchased levels is achieved. The HHS has a responsibility to only recognise revenue that is linked with actual activity delivered against purchased volumes.

4.3 The HHS will actively monitor variances from purchased activity levels and notify the Department as soon as the HHS becomes aware of significant variances.

4.4 The HHS will also notify the Department of deliberate changes to the consistent recording of activity within year that would result in additional activity being recorded for existing health services.

4.5 The HHS will undertake regular quality audits. The HHS is encouraged to publish its data quality framework describing audits undertaken and results achieved. For further information, refer to the Delivery of Purchased Activity – Requirement for Quality Audits specification sheet as detailed in Appendix 1.

4.6 If the HHS wishes to convert activity between purchased activity types, programs, and levels the HHS must negotiate this with the Department based on a sound needs based rationale.

4.7 The Department will reconcile in-scope activity, as defined in the Activity Reconciliation specification sheet (available online, as detailed in Appendix 1), delivered by the HHS against the purchased in-scope activity targets outlined in Table 5.

4.8 Activity reconciliations will be undertaken in the applicable End of Year Technical Amendment Window and subsequent Amendment Window 2 and will be derived through application of the methodology which is documented in the Activity Reconciliation specification sheet.

4.9 Should the HHS be unable to deliver the activity that has been funded a financial adjustment will be applied.

4.10 Funding and corresponding activity that is withdrawn from the HHS may be reallocated to an alternate provider that can undertake the activity.

4.11 Any resulting changes to funding and purchased activity levels will be actioned through the processes outlined in Schedules 2 and 5 of this service agreement.

4.12 The HHS will maintain its efforts to accurately record the delivered activity and submit this data to the Department in accordance with the agreed requirements.

5. Delivery of purchased services

5.1 As part of the service agreement, the Department purchases a range of services (or deliverables) from the HHS for the delivery of certain programs and projects.

5.2 These program or project services may be the subject of separately agreed conditions tied to that funding and the focus of detailed monitoring by the Department.

5.3 Conditions may include, but are not limited to:

(a) establishment and/or commencement of services;

(b) delivery of activity;

(c) workforce obligations;

(d) establishment of oversight committees;

(e) opening or upgrades to facilities;

(f) program evaluation;

(g) program management;

(h) reporting or notification obligations; and

(i) attainment of performance standards.

5.4 The HHS will ensure that the conditions are achieved within the stipulated time period.

5.5 The HHS will notify the Department if the HHS forecasts an inability to achieve program or project objectives or the conditions.

5.6 The Department may withdraw allocated funding pro rata to the level of under delivery if the services for a specified program or project are not being fully delivered according to the objectives or conditions.

5.7 Any resulting changes to funding and purchased activity levels will be actioned through the processes outlined in Schedules 2 and 5 of this service agreement.

6. Financial adjustments

6.1 **Activity targets**

(a) The Department will initiate a joint process with the HHS to determine whether a financial adjustment should be applied in relation to any purchased activity which has breached the thresholds identified bi-annually. This process will take into account any relevant matters that have been identified in a review/analysis of the breach as well as the outcomes of the activity plan implemented to address the activity breach.

(b) Activity will be monitored at the purchasing hierarchy level. Providing the HHS meets all relevant KPIs and specific funding allocations, the HHS has the ability to negotiate the transfer of activity across the purchasing hierarchy with the Department.

(c) Table 2 demonstrates the financial adjustment that will be applied when activity thresholds have been breached.

(d) The HHS may not utilise the provisions within AASB15 Revenue from Contracts with Customers to override the application of any financial adjustment made by the Department in line with Table 2.

Table 2 Financial adjustments applied on breach of activity thresholds

Example of Breach	Description	Financial Adjustment
Over performance	Activity exceeds that specified in the service agreement value for in-scope activity as shown in Table 5.	Purchasing contracts are capped and an HHS will not be paid for additional activity apart from activity that is in scope for the identified purchasing incentives as set out in Table 3 (where applicable.)
Under performance	Activity is below that specified for in-scope activity as shown in Table 5.	Purchased activity and the related funding will be withdrawn at 100% of the Queensland Efficient Price and reallocated to an alternate provider that can undertake the activity. The reconciliation will be undertaken as outlined in the Activity Reconciliation Specification. Refer to Table 5 for the HHS QWAU target.
Failure to deliver on service commitments linked to specific funding allocations	Specific funding allocations<br>National Partnership Agreements.	It is at the discretion of the Department to withdraw allocated funding pro rata to the level of under delivery.
For all other types of activity variance, any financial adjustment will be made at the discretion of the Department.		

6.2 Purchasing approach

(a) The purchasing approach includes a range of funding adjustments (purchasing incentives and ABF model localisations) that aim to incentivise high quality and high priority activity, support innovation and evidence-based practice, deliver additional capacity through clinically and cost-effective models of care and dis-incentivise care providing insufficient or no benefit for patients. This includes incentive payments for HHSs who achieve targets in specific priority areas. The funding adjustments are detailed in Table 3. The Department must reconcile the applicable funding adjustments in Table 3 in line with the timeframes specified in the relevant purchasing specification sheet. The Department must provide a copy of the reconciliation statement to the HHS.

(b) Funding adjustments must be based on the requirements contained in the relevant purchasing specification sheet.

(c) Any resulting changes to funding and purchased activity levels will be actioned through the processes outlined in Schedules 2 and 5 of this service agreement.

Table 3 Funding adjustments 2024/25

Funding adjustment	
**Purchasing incentives**	
Models of care/workforce	This program includes a range of initiatives focusing on incentivising:<br>• specific models of care; and<br>• the use of workforce operating at top of scope where there may be long wait lists and staff have not been available in a traditional model of care.
**ABF model localisations**	
Child Health Checks	QWAU loading for every in-scope check performed.
Unqualified neonate funding	Reduced Diagnosis Related Group (DRG) QWAU for all maternal delivery episodes with a liveborn outcome, discounted by the Diagnosis Related Group (DRG), with QWAUs re-allocated for unqualified neonates.
Maternity care for First Nations women	QWAUs to incentivise maternity care provided to First Nations mothers during pregnancy and to incentivise smoking cessation during pregnancy.
Sentinel events	Payment withdrawn for sentinel events as per the national ABF model.
Advance Care Planning (ACP)	QWAUs for HHSs offering ACP discussions to admitted patients, non-admitted outpatients, community health patients and Emergency Department patients.
Emergency Department Did Not Wait (DNW)	Zero QWAUs for DNWs.
Fractured neck of femur	QWAUs reduced by 20% for non-timely surgical treatment of fractured neck of femur.
Hospital in The Home (HITH)	QWAUs increased by 12.5% for Hospital in the Home (HITH) admissions of Residential Aged Care Facility residents.
Out-of-scope services	Nil QWAUs for out-of-scope procedures.
Pre-operative bed days	QWAUs for long stay days above the upper trim point less ICU days reduced equivalent to pre-operative days, up to a maximum of 3 days, for elective episodes.
Smoking cessation (community mental health)	QWAUs for smoking cessation activity for community mental health patients.
Smoking cessation (inpatients)	QWAUs for smoking cessation activity for publicly funded inpatients.
Stroke care	10% QWAU loading for acute stroke patients admitted to Queensland Stroke Clinical Network-endorsed stroke unit care.
Telehealth (admitted patients)	QWAUs for provider-end of in-scope admitted patient telehealth activity.
Allied Health Led Workforce for Pelvic Health and Gastroenterology	QWAU loading for an in-scope service event for Pelvic Health and Gastroenterology recorded against an Other Health Professional.
Remote Patient Monitoring	QWAU loading for an in-scope non-admitted remote patient monitoring encounter per month per patient.

Surgery Connect reimbursements

(a) The HHS will reimburse the Department for the actual costs of activity for nominated patient referrals to the Surgery Connect program where:

(i) The HHS has nominated the patient referral as HHS funded or HHS Direct on entry of the referral in the Surgery Connect Activity Navigator (SCAN); and

(ii) The HHS Chief Finance Officer has recorded approval of the nomination in SCAN;

or

(iii) The HHS has obtained the Department's agreement to retrospectively convert a defined patient cohort to HHS funded status in SCAN.

(b) The HHS may only request retrospective conversion of activity to HHS funded within the financial year in which the activity has taken place.

(c) Any resulting changes to funding and purchased activity levels will be actioned through the processes outlined in Schedules 2 and 5 of this service agreement.

6.3 Financial adjustments – other

(a) Notwithstanding the provisions regarding the recognition of revenue as stipulated in AASB1058 Income of Not-for-Profit Entities and/or AASB15 Revenue from Contracts with Customers, the Department may seek to recover funding from an HHS that was provided through this service agreement which has:

(i) not been utilised in accordance with its intended purpose; and/or

(ii) not been utilised within the prescribed time period to deliver the agreed outcomes/services.

(b) If the Chief Executive (or delegate) determines that previously allocated funding is to be recovered, any resulting changes to funding and purchased activity levels will be actioned through the processes outlined in Schedules 2 and 5 of this service agreement.

6.4 Public and private activity/own source revenue

(a) Own source revenue comprises grants and contributions, user charges and other revenues.

(b) Where an HHS is above its own source revenue target, it will be able to retain the additional own source revenue with no compensating adjustments to funding from other sources where the additional revenue is not attributable to a private patient

consistent with clauses A9 to A13 and A44 of the *National Health Reform Agreement.*

(c) Where an HHS is below its own source revenue target in respect of private patients, it will experience a reduction in revenue with no compensating adjustments to funding from other sources.

(d) The HHS will make all efforts to identify and maximise revenue from appropriate third parties to ensure optimisation of available State and Commonwealth funding across the Queensland public sector health system.

(e) The own source revenue identified in Table 5 is an estimate generated by the HHS which allows all third-party funding sources associated with service delivery to be identified. The HHS will ensure that this estimate is substantiated and accurate to ensure no significant variances to actual revenue generated.

(f) The HHS will routinely revise and update the estimate to ensure alignment between the service agreement and Queensland Treasury's reporting system (TRIDATA).

(g) Budget adjustments for changes in own source revenue from private patients will be actioned through the processes set out in Schedule 5 of this service agreement.

7. Funding sources

7.1 The four main funding sources contributing to the HHS service agreement value are:

(a) Commonwealth funding;

(b) State funding;

(c) grants and contributions; and

(d) own source revenue.

7.2 Table 5 provides a summary of the funding sources for the HHS and the total value of the service agreement.

7.3 The HHS must undertake regular quality audits to check for potential duplicates in funding source, in particular the National Health Reform Agreement and Medicare given the Commonwealth's contribution to both funding sources. The HHS should take active steps to remedy areas of concern. A consumer's choice of funding arrangement should be reflected on a patient election form.

8. Funds disbursement

8.1 The Department agrees to pay the HHS the amount described in Table 5 of Schedule 2, subject to:

(a) parliamentary appropriation and adequate funds being allocated to the Department; and

(b) the terms of this service agreement.

8.2 All payments under this service agreement will be made in accordance with the requirements of the *National Health Reform Agreement* and the Act.

8.3 The Chief Executive will direct the disbursement of both State and Commonwealth funding from the State Pool Account, the State Managed Fund, and the Department of Health Expenditure account to the HHS.

8.4 However, the State (represented by the Chief Executive) will not:

(a) redirect Commonwealth payments between HHSs;

(b) redirect Commonwealth payments between funding streams (e.g., from ABF to block funding); and/or

(c) adjust the payment calculations underpinning the Commonwealth's funding.

8.5 The Department will pay state-funded activity-based funding, block funding and system manager funding to the HHS on a fortnightly basis in line with receipt of the State Appropriation Payment. The payment made will be equal to 1/26th of the state-funded component of the service agreement value described in Table 5.

8.6 The Department will pay Commonwealth-funded activity-based funding and block funding monthly in line with receipt of the Commonwealth payment. The payment made will be equal to 1/12th of the Commonwealth funded component of the service agreement value described in Table 5.

8.7 Where the parties have agreed to amend the service agreement value or the Commonwealth contribution amount changes, the fortnightly and/or monthly payments will be adjusted to reflect the amended service agreement value.

8.8 Service agreement payments may be made outside of these timeframes with the approval of the Department's Chief Finance Officer.

Table 4 HHS Funding by Outcomes Framework Care Domain 2024/25

Care Domain	Funding $	QWAU (Q27)
Prevention, early intervention, and primary health care	$49,447,111	2,157
Trauma and illness	$106,180,737	11,397
Mental health and alcohol and other drugs	$14,274,973	1,152
Cancer	$8,541,161	938
Planned care	$18,421,652	2,149
Maternity and neonates	$17,111,835	1,889
Chronic and complex	$53,680,386	5,710
Statewide services	$569,112	21
Depreciation	$16,444,000	0
**TOTAL**	**$284,670,967**	**25,413**

Table 5 HHS Total Funding Allocation by Funding Source 2024/25

Funding Source	24-25 NWAU (N2425)	24-25 QWAU (Q27)	24-25 Agreed ($)
**NHRA Funding**			
**ABF Pool**			
**ABF Funding (In scope NHRA)**[2]			
Commonwealth	18,823		$45,990,079
State		18,383	$78,181,624
State Specified Grants			$9,320,055
State-wide Services			$0
Restoring Planned Care	32	31	$180,000
Long Stay Patient Recovery Funding	0	0	$0
**Total ABF Funding (in scope NHRA)**	**18,855**	**18,414**	**$133,671,759**
**State Managed Fund**			
**Block Funding (State and Commonwealth)**			
Small rural hospital		4,664	$44,192,466
Teaching, Training and Research			$4,606,968
Other Mental Health	629	629	$4,370,124
Non-Admitted Home Ventilation			$0
Residential Mental Health Services		0	$0
Other Non-admitted Service			$0
Highly Specialised Therapies			$0
Other Public Hospital Programs			$0
**Total NHRA Funding**	**18,855**	**23,707**	**$186,841,317**
**Out of Scope NHRA**			
**Queensland ABF Model**			
DVA		34	$202,156
NIISQ/MAIC		45	$266,509
Oral Health		294	$2,016,452
Oral Health – FFA		131	$770,000
BreastScreen		0	$0
Child Health Checks		66	$1,227,064
**Total Queensland ABF Funding**		**571**	**$4,482,181**
**Discretely Funded Programs**[3]			
Department of Health			$62,753,313
Locally Receipted Funds			$0
Research (Other OSR)			$0
**Total Discretely Funded Programs**			**$62,753,313**

[2] The split between Commonwealth and State NHRA funding will change during the year as the purchased activity targets are updated and the National Health Funding Board updates their payment advice accordingly.

[3] Includes all other (non-ABF) State and Commonwealth funded health services including, but not limited to, Prisoner Health, Public Health and Prevention Services.

Funding Source	24-25 NWAU (N2425)	24-25 QWAU (Q27)	24-25 Agreed ($)
**Own Source Revenue**			
Private Patient Admitted Revenue[4]	55	54	$320,108
Pharmaceuticals Benefits Scheme		543	$4,353,030
Non-Admitted Services		329	$155,314
Other Activities[5]		209	$4,183,915
Oral Health – CDBS		0	$100,000
**Total Own Source Revenue**	**-**	**1,135**	**$9,112,367**
Locally Receipted Funds (exc. Discretely Funded Programs)[6]			$5,037,789
Depreciation			$16,444,000
**GRAND TOTAL**	**18,855**	**25,413**	**$284,670,967**

Pool Accounts			
ABF Pool (National Health Funding Pool)[7]			$138,153,939
State Managed Fund[8]			$53,169,558
System Manager			$62,753,313

[4] The estimated value of the revenue earned from private patients, based on OSR estimates provided by the HHS.
[5] Incorporates all OSR which is not identified elsewhere in Table 5.
[6] Includes items such as training programs and donations. Does not include locally receipted funds associated with discretely funded programs, e.g.Transition Care.
[7] Articulates the financial payment made to support in-scope ABF services under the NHRA including DVA, NIISQ, MAIC and Breastscreen Services. Applies to all HHSs except Central West HHS and Torres and Cape HHS.
[8] Articulates the payment made for block funded services under the NHRA, DVA, NIISQ and MAIC services.

Table 6 National Health Reform Funding

NHRA Funding Type	No. of In-scope services (NWAU)	No. of Out-of-scope services (QWAU)	Total Services (WAU)	ABF NEP ($)	State Price ($)	Funding for In-scope services at NEP ($)	Cwlth contribution for In-scope services ($)	State contribution for In-scope services ($)	State contribution for Out-of-scope services ($)	Total Cwlth and State contribution ($)
National Efficient Price (NEP)		*a,b*		*c*	*d*			*e*		
**ABF Allocation (NWAU)**										
Emergency Department	4,962	28	4,990	$6,465	$5,878	32,080,905	12,124,307	23,067,957	213,196	35,405,460
Acute Admitted	9,813	37	9,850	$6,465	$5,878	63,441,045	23,976,215	45,617,645	282,984	69,876,845
Admitted Mental Health	332	31	363	$6,465	$5,878	2,146,009	811,039	1,543,100	238,648	2,592,787
Sub-Acute	1,370	12	1,382	$6,465	$5,878	8,860,048	3,348,470	6,370,868	92,871	9,812,209
Non-Admitted	2,345	495	2,840	$6,465	$5,878	15,161,703	5,730,049	10,902,109	3,834,481	20,466,639
**Total ABF Allocation**	**18,823**	**602**	**19,425**			**121,689,709**	**45,990,079**	**87,501,679**	**4,662,181**	**138,153,939**
**Block Allocation**										
Teaching, Training, and Research						0	1,224,137	3,382,831	0	4,606,968
Small and Rural Hospitals[9]						0	12,696,620	31,495,846	0	44,192,466

[9] Incorporating small regional and rural public hospitals, four specialist mental health facilities (Baillie Henderson Hospital, Jacaranda Place – Queensland Adolescent Extended Treatment Centre, The Park – Centre for Mental Health and Kirwan Rehabilitation Unit) and the Ellen Barron Family Centre.

NHRA Funding Type	No. of In-scope services (NWAU)	No. of Out-of-scope services (QWAU)	Total Services (WAU)	ABF NEP ($)	State Price ($)	Funding for In-scope services at NEP ($)	Cwlth contribution for In-scope services ($)	State contribution for In-scope services ($)	State contribution for Out-of-scope services ($)	Total Cwlth and State contribution ($)
Other Mental Health						0	1,628,881	2,741,243	0	4,370,124
Non-Admitted Home Ventilation						0	0	0	0	0
Other Non-Admitted Services						0	0	0	0	0
Other Public Hospital Programs						0	0	0	0	0
Highly Specialised Therapies						0	0	0	0	0
**Total Block Allocation**						**0**	**15,549,638**	**37,619,921**	**0**	**53,169,558**
**Grand Total Funding Allocation**										**191,323,497**

Notes

a. QWAU refers to Queensland Weighted Activity Units in Q27 phase (built on N2425)
b. DVA, NIISQ/MAIC, Oral Health, Child Health Checks and BreastScreen
c. Queensland Efficient Price used to Purchase growth QWAUs
d. NWAU x NEP
e. State funding transacted through the Pool/State Managed Fund Account; not covered under the NHRA
-NWAU estimates do not take account of cross-border activity.

Table 7 Discretely Funded Programs (Non-ABF)

Discretely Funded Programs	Revenue Models	$
Aged Care Assessment Program	Commonwealth	$232,306
Alcohol, Tobacco and Other Drugs	State	$4,465,612
Community Health Programs	State	$18,784,411
Interstate Patients (QLD Residents)	State	$518,589
Other State Funding	State	$14,304,915
Patient Transport: PTSS	State	$15,222,080
Patient Transport: Aeromedical Retrieval	State	$5,954,117
Patient Transport	State	$0
Prevention Services and Public Health	Commonwealth	$812,600
	State	$604,107
Prisoner Primary Health Services	State	$0
Disability Residential Care Services	State	$0
Multi-Purpose Health Services	Commonwealth	$1,648,035
Residential Aged Care Services	Commonwealth	$93,550
	Locally Receipted Funds	$0
	State	$112,991
Transition Care	Locally Receipted Funds	$0
Research	Commonwealth	$0
	OSR	$0
Home and Community Care (HACC) Program	Locally Receipted Funds	$0
**Discretely Funded Programs Total**		**$62,753,313**
**TOTAL**		**$62,753,313**

Schedule 3 Department of Health Provided Services

1. In scope services and service schedules

Table 8 Department of Health provided services and service schedules

Provider	Service provided	Link to Service Statement
Corporate Services Division (CSD)	• Corporate Enterprise Solutions<br>• Finance Branch:<br>o Accounts Payable Service Provision<br>o Banking and Payment Services<br>• Central Pharmacy<br>• Group Linen Services<br>• Transport and Logistic Services<br>• Supply Chain Services	CSD Service Schedules
eHealth Queensland (eHQ)	• ICT Service	eHQ Service Schedule
Queensland Public Health and Scientific Services Division (QPHaSS)	• Pathology Queensland<br>• Biomedical Technical Services<br>• Public Health Services	QPHaSS Service Schedules

Schedule 4 Performance Measures

1. Performance Measures

1.1 The performance of the HHS will be measured according to the assessment criteria and processes described in the Performance and Accountability Framework.

1.2 Existing performance indicators are mapped to the care domains of the Outcomes Framework.

1.3 The detailed specification for each of the performance measures listed in this service agreement are provided through performance measure attribute sheets.

1.4 The performance measures identified in this service agreement are applicable to the HHS unless otherwise specified within the attribute sheet.

1.5 HHSs are also required to report against the agreed Statewide Health Equity Key Performance Measures (Table 12).

Table 9 HHS Performance Measures – Key Performance Indicators

Outcomes Framework Care Domain	Key Performance Indicators	Indicator Number
Chronic and complex	Hospital Acquired Complications (IHACPA code 8, 11, 13, 14)	31
Chronic and complex	Potentially Preventable Hospitalisations – First Nations peoples:<br>• Diabetes complications<br>• Selected conditions	37a<br>37b
Chronic and complex	Potentially Preventable Hospitalisations (non-diabetes complications)	39
Chronic and complex	Proportion of diabetic patients with a current HbA1c result	59
Chronic and complex	Potentially avoidable deaths - First Nations Peoples	70
Maternity and neonates	Hospital Acquired Complications (IHACPA code 15,16)	31
Maternity and neonates	% of low birthweight babies born to Queensland mothers	41
Maternity and neonates	Proportion of healthy birthweight babies (2,500 grams to 4,499 grams at birth) – Aboriginal and Torres Strait Islander babies	71
Mental health, alcohol, and other drugs	Proportion of mental health and alcohol and other drug service episodes with a documented care plan	27
Mental health, alcohol, and other drugs	Suicide count and rate – First Nations Peoples	72
Other	Forecast operating position:<br>• Full year<br>• Year to date	48<br>49
Other	Average sustainable Queensland Health FTE	50
Other	Capital expenditure performance	51

Outcomes Framework Care Domain	Key Performance Indicators	Indicator Number
Planned care	Elective surgery patients waiting longer than the clinically recommended timeframe	9
Planned care	Gastrointestinal endoscopy patients waiting longer than the clinically recommended timeframe	16
Planned care	Telehealth utilisation rates:<br>• Number of non-admitted telehealth service events	20
Planned care	Hospital Acquired Complications (IHACPA code 1,2,3,4,6,7,9,10,12)	31
Planned Care	Missed Opportunity to Treat – Outpatients	73
Prevention, early intervention, and primary health care	Access to oral health services (adults)	21
Prevention, early intervention, and primary health care	The percentage of oral health activity which is preventive	23
Prevention, early intervention, and primary health care	Patients whose smoking status has been recorded	58
Prevention, early intervention, and primary health care	Access to oral health services (children)	67
Prevention, early intervention, and primary health care	Potentially avoidable deaths - First Nations Peoples	70
Prevention, early intervention, and primary health care	Suicide count and rate – First Nations Peoples	72
Prevention, early intervention, and primary health care	*Care Closer to Home (placeholder):*<br>• *Self-sufficiency: admitted patients*<br>• *Self-sufficiency: non-admitted patients*	*NA*
Prevention, early intervention, and primary health care	*Participation in the Practice Incentive Program (placeholder)*	*NA*

Table 10 HHS Performance Measures - Safety and Quality Markers

Outcomes Framework Care Domain	Safety and Quality Markers	Indicator Number
Maternity and neonates	Sentinel Events	32
Planned care	Sentinel Events	32
Planned care	Hospital Standardised Mortality Ratio	33
Planned care	Severity Assessment Code (SAC1) analysis completion rates	34
Planned care	Patient Reported Experience	68

Table 11 HHS Performance Measures – Outcome Indicators

Outcomes Framework Care Domain	Outcome Indicators	Indicator Number
Chronic and complex	Advance care planning	43
Chronic and complex	Integrated care pathways:<br>• Care pathway in place for patients with identified co-morbidities	60
Mental health, alcohol and other drugs	Smoking cessation clinical pathway (community mental health)	42
Other	First Nations peoples' representation in the workforce	47
Planned care	Complaints resolved within 35 calendar days	36
Prevention, early intervention, and primary health care	General oral health care for First Nations peoples	22
Prevention, early intervention, and primary health care	Access to emergency dental care	24
Prevention, early intervention, and primary health care	Smoking cessation clinical pathway (admitted episodes and dental)	42
Prevention, early intervention, and primary health care	Adolescent vaccinations administered via the statewide School Immunisation Program	45

Table 12 Statewide Health Equity Key Performance Measures

Outcomes Framework Care Domain	Key Performance Measures	Indicator Number
Chronic and complex	Advance care planning	43
Chronic and complex	Integrated care pathways - Rural and Remote HHSs:<br>• Care pathway in place for patients with identified co-morbidities	60
Maternity and neonates	Proportion of healthy birthweight babies (2,500 grams to 4,499 grams at birth) – Aboriginal and Torres Strait Islander babies	71
Mental health, alcohol, and other drugs	Face to face community follow up within 1-7 days of discharge from an acute mental health inpatient unit	26
Mental health, alcohol, and other drugs<br>Chronic and complex	Suicide count and rate – First Nations People	72
Other	First Nations peoples' representation in the workforce	47
Planned care	Category 1 elective surgery patients treated within the clinically recommended timeframe	7
Planned care	Category 2 and 3 elective surgery patients treated within the clinically recommended timeframe	8
Planned care	Category 1 patients who receive their initial specialist outpatient appointment within the clinically recommended timeframe	17
Planned care	Category 2 and 3 patients who receive their initial specialist outpatient appointment within the clinically recommended timeframe	18

Outcomes Framework Care Domain	Key Performance Measures	Indicator Number
Prevention, early intervention, and primary health care	General oral health care for First Nations peoples	22
Prevention, early intervention, and primary health care	Potentially avoidable deaths – First Nations peoples	70

Schedule 5 Amendments to this service agreement

1. Agreed process to amend this service agreement

1.1 The parties acknowledge that this service agreement is subject to amendment, which will generally occur through:

(a) amendment windows;

(b) extraordinary amendment;

(c) periodic adjustments; and

(d) end of financial year reconciliation.

1.2 **Amendment windows**

(a) There will be set periods of time nominated by the Department during the year (amendment windows) in which a party may propose an amendment and the parties will endeavour to negotiate and finalise proposals to amend this service agreement.

(b) Amendment proposals that are agreed will be documented in a deed of amendment to this service agreement.

(c) Further details on the amendment window process, including the timing of amendment windows, is provided online as detailed in Appendix 1.

1.3 **Extraordinary Amendment**

(a) Outside an amendment window, the Department and the HHS agree to limit any proposal to amend the terms of this service agreement to those where there is an urgent priority need to facilitate a funding allocation (extraordinary amendment). The parties will endeavour to negotiate and finalise any such proposal urgently.

(b) The process for submitting, negotiating, and resolving an extraordinary amendment is available online as detailed at Appendix 1.

(c) Agreed extraordinary amendments will be reflected in a notice issued by the Chief Executive and countersigned as accepted by the HHS. The notice will be replaced when the extraordinary amendment is subsequently formalised in a deed of amendment issued following the next amendment window.

1.4 **Periodic adjustments**

(a) The service agreement value (and corresponding purchased activity) may be adjusted at any time to reflect funding variations that:

(i) occur on a periodic basis or in line with adjustments permitted for specific funding allocations;

(ii) are referenced in the service agreement; and

(iii) are based on a clearly articulated formula, an agreed basis or such other reasonably substantiated basis tied to performance.

(b) Periodic adjustments will be reflected in an adjustment notice issued by the Chief Executive (or delegate) to the HHS, based on relevant data, and subsequently formalised in a deed of amendment issued following the next amendment window.

1.5 End of financial year reconciliation

(a) There will be an end of financial year reconciliation process, with the scope defined by the Department and informed by Queensland Government Central Agency requirements.

(b) The Department will provide the HHS with a reconciliation of all service agreement funding and purchased activity for the prior financial year. This will reflect the position following conclusion of the end of financial year adjustments process.

(c) The impact of end of financial year adjustments on subsequent year funding and activity will be incorporated in the service agreement through the deed of amendment executed following the next available amendment window.

(d) This clause will survive expiration of this service agreement.

Appendix 1 Reference Documents

Service Agreement:

- Data supply requirements
- Delivery of Purchased Activity – Requirement for Quality Audits specification sheet
- Dispute resolution process - current
- First Nations First Strategy 2032
- Funding Outcomes Framework
- Hospital and Health Boards Act 2011
- Joint Regional Needs Assessment Framework
- Making Tracks Together: Queensland's Aboriginal and Torres Strait Islander Health Equity Framework
- Making Tracks toward closing the gap in health outcomes for Indigenous Queenslanders by 2033 - Policy and Accountability Framework
- National Agreement on Closing the Gap
- National Health Reform Agreement (NHRA) 2020-25
- Performance Measures Attribute Sheets
- Public Health Practice Manual
- Queensland Government Building Policy Framework and Guideline
- Queensland Health Performance and Accountability Framework
- Service agreement amendment processes
- Specifications supporting the Healthcare Purchasing Model
- Statewide services reference material

Supporting Policy documents

- Aboriginal and Torres Strait Islander Health Workforce Strategic Framework 2016-2026
- Department of Health Strategic Plan 2021-2025

- HEALTHQ32: A vision for Queensland's health system
- My health, Queensland's future: Advancing health 2026
- Queensland Health Equity, Diversity, and Inclusion Statement of Commitment
- System Outlook to 2026 - for a sustainable health service

Contracting and Performance Management Branch
GPO Box 48
Brisbane QLD 4001 Australia

Tel (+61) (07) 3708 5869

www.health.qld.gov.au

Service Agreement
2019/20 – 2021/22
Torres and Cape Hospital and Health Service

July 2020 Revision

Queensland Government

Torres and Cape Hospital and Health Service

Service Agreement 2019/20 - 2021/22, July 2020 Revision

Published by the State of Queensland (Queensland Health), July 2020

This document is licensed under a Creative Commons Attribution 3.0 Australia licence. To view a copy of this licence, visit creativecommons.org/licenses/by/3.0/au

© State of Queensland (Queensland Health) **2020**

You are free to copy, communicate and adapt the work, as long as you attribute the State of Queensland (Queensland Health).

For more information contact:
Contracting and Performance Management Branch, Department of Health, GPO Box 48, Brisbane QLD 4001, email SAM@health.qld.gov.au, phone (07) 3708 5869.

An electronic version of this document is available at
www.health.qld.gov.au/system-governance/health-system/managing/default.asp

Disclaimer:
The content presented in this publication is distributed by the Queensland Government as an information source only. The State of Queensland makes no statements, representations or warranties about the accuracy, completeness or reliability of any information contained in this publication. The State of Queensland disclaims all responsibility and all liability (including without limitation for liability in negligence) for all expenses, losses, damages and costs you might incur as a result of the information being inaccurate or incomplete in any way, and for any reason reliance was placed on such information.

Contents

1. Introduction ... 6
2. Interpretation ... 6
3. Legislative and regulatory framework ... 7
4. Health system priorities ... 7
5. Objectives of the Service Agreement ... 9
6. Scope ... 9
7. Performance and Accountability Framework ... 9
8. Period of this Service Agreement ... 10
9. Amendments to this Service Agreement ... 10
10. Publication of amendments ... 10
11. Cessation of service delivery ... 10
12. Commencement of a new service ... 11
13. Provision of data to the Chief Executive ... 12
14. Dispute resolution ... 12
15. Force Majeure ... 15
16. Hospital and Health Service accountabilities ... 16
17. Department accountabilities ... 16
18. Insurance ... 18
19. Indemnity ... 18
20. Indemnity arrangements for officers, employees and agents ... 19
21. Legal proceedings ... 19
22. Sub-contracting ... 19
23. Counterparts ... 20

Execution ... 21

Schedule 1 HHS Accountabilities ... 22

1. Purpose ... 22
2. Registration, credentialing and scope of clinical practice ... 22
3. Clinical Services Capability Framework ... 22
4. Clinical Prioritisation Criteria ... 23
5. Service delivery ... 23
6. Accreditation ... 24
7. Responsive regulatory process for accreditation ... 25
8. Achieving health equity for First Nations Queenslanders ... 26
9. Provision of Clinical Products/Consumables in outpatient settings ... 27
10. Capital, land, buildings, equipment and maintenance ... 28
11. Occupational health and safety ... 30
12. Workforce management ... 30
13. Medically authorised ambulance transports ... 31

Schedule 2 Funding, purchased activity and services ... 32

1. Purpose ... 32
2. Delivery of purchased activity ... 32
3. Financial adjustments ... 33

4. Funding sources 43
5. Funds disbursement 45
6. Purchased services 54
7. Teaching training and research 61
Schedule 3 Performance Measures 64
1. Purpose 64
2. Performance Measures 64
Schedule 4 Data Supply Requirements 68
1. Purpose 68
2. Principles 68
3. Roles and responsibilities 68
Attachment A Data Supply Requirements 70
Schedule 5 Amendments to this Service Agreement 72
1. Purpose 72
2. Principles 72
3. Process to amend this Service Agreement 72
Schedule 6 Definitions 77
Key Documents 84
Abbreviations 86

Figures

Figure 1 Dispute resolution process ... 13
Figure 2 Inter-HHS dispute resolution process ... 15
Figure 3 Amendment Proposal negotiation and resolution ... 74

Tables

Table 1 Specific Funding Commitments ... 34
Table 2 Purchasing Incentives 2020/21 (Summary) ... 42
Table 3 Hospital and Health Service funding sources 2020/21 ... 44
Table 4 HHS Finance and Activity Schedule 2019/20 – 2021/22 – Summary by Purchasing Hierarchy ... 46
Table 5 Minor Capital and Equity ... 48
Table 6 HHS Finance and Activity Schedule 2019/20 – 2021/22 Other Funding Detail ... 49
Table 7 Specified Grants ... 52
Table 8 Hospital and Health Service Service Agreement and State Level Block Payments to state managed funds from Commonwealth payments into national funding pool ... 53
Table 9 HHS Performance Measures – Key Performance Indicators ... 65
Table 10 HHS Performance Measures - Safety and Quality Markers ... 66
Table 11 HHS Performance Measures – Outcome Indicators ... 67
Table 12 Clinical data ... 70
Table 13 Non-clinical data ... 71
Table 14 Amendment Window Exchange Dates ... 73

1. Introduction

1.1 The Queensland Public Sector Health System is committed to strengthening performance and improving services and programs in order to meet the needs of the community and deliver improved health outcomes to all Queenslanders.

1.2 The development of Service Agreements between the Chief Executive and Hospital and Health Services (HHSs), assists this process by formally assigning accountability for the high-level outcomes and targets to be met during the period to which the Service Agreement relates.

1.3 The content and process for the preparation of this Service Agreement is consistent with the requirements of the *Hospital and Health Boards Act 2011*. As such this Service Agreement specifies:

(a) the Health Services and other services to be provided by the HHS;

(b) the funding which is provided to the HHS for the provision of these services and the way in which the funding is to be provided;

(c) the Performance Measures that the HHS will meet for the services provided;

(d) data supply requirements; and

(e) other obligations of the Parties.

1.4 Fundamental to the success of this Service Agreement is a strong collaboration between the HHS and its Board and the Department. This collaboration is supported through regular Performance Review Meetings attended by representatives from both the HHS and the Department which provide a forum within which a range of aspects of HHS and system wide performance are discussed and jointly addressed.

2. Interpretation

Unless expressed to the contrary, in this Service Agreement:

(a) words in the singular include the plural and vice versa;

(b) any gender includes the other genders;

(c) if a word or phrase is defined its other grammatical forms have corresponding meanings;

(d) “includes” and “including” are not terms of limitation;

(e) no rule of construction will apply to a clause to the disadvantage of a Party merely because that Party put forward the clause or would otherwise benefit from it;

(f) a reference to:

(i) a Party is a reference to a Party to this Service Agreement;

(ii) a person includes a partnership, joint venture, unincorporated association, corporation and a government or statutory body or authority;

(iii) a person includes the person's legal personal representatives, successors, assigns and persons substituted by novation;

(g) any legislation includes subordinate legislation under it and includes that legislation and subordinate legislation as modified or replaced;

(h) a reference to a role, function or organisational unit is deemed to transfer to an equivalent successor role, function or organisational unit in the event of organisational change or restructure in either Party;

(i) an obligation includes a warranty or representation and a reference to a failure to comply with an obligation includes a breach of warranty or representation;

(j) headings do not affect the interpretation of this Service Agreement;

(k) unless the contrary intention appears, a reference to a Schedule, annexure or attachment is a reference to a Schedule, annexure or attachment to this Service Agreement; and

(l) unless the contrary intention appears, words in the Service Agreement that are defined in Schedule 6 'Definitions' have the meaning given to them in that Schedule.

3. Legislative and regulatory framework

3.1 This Service Agreement is regulated by the National Health Reform Agreement and the provisions of the *Hospital and Health Boards Act 2011.*

3.2 The National Health Reform Agreement requires the State of Queensland to establish Service Agreements with each HHS for the purchasing of health services and to implement a performance and accountability framework including processes for remediation of poor performance. The *Hospital and Health Boards Act 2011* states under section 35(3) that the Service Agreement executed between the Chief Executive and the Hospital and Health Board Chair binds each of them.

3.3 The *Hospital and Health Boards Act 2011* recognises and gives effect to the principles and objectives of the national health system agreed by the commonwealth, state and territory governments, including the Medicare principles and health system principles set out in section 4. Section 5 of the *Hospital and Health Boards Act 2011* states that the object of the Act is to establish a Public Sector Health System that delivers high-quality hospital and other Health Services to persons in Queensland having regard to the principles and objectives of the national health system. This Service Agreement is an integral part of implementing these objectives and principles.

4. Health system priorities

4.1 Ensuring the provision of Public Sector Health Services across Queensland requires clear priorities, supportive leadership and staff who work together and across each level of the Public Sector Health System. The Parties recognise that they each have a mutual and reciprocal obligation to work collaboratively with each other, with other Hospital and Health

Services (HHS) and with the Queensland Ambulance Service in the best interests of the Queensland Public Sector Health System.

4.2 The priorities, goals and outcomes for the Queensland Public Sector Health System are defined through:

(a) *Our Future State: Advancing Queensland's Priorities* - the Queensland Government's objectives for the community; and

(b) *My health, Queensland's future: Advancing health 2026* – the vision and strategy for Queensland's health system.

4.3 The Parties will also work collaboratively to deliver the *Queensland Health 2020/21 System Priorities.* The *Queensland Health 2020/21 System Priorities* establishes a tactical framework which will ensure that the Queensland Public Sector Health System delivers sustainable, high quality and timely Health Services during 2020/21, whilst remaining positioned to respond effectively to the COVID-19 pandemic.

4.4 Additionally, the Queensland Government, Premier or the Minister for Health and Minister for Ambulance Services (The Minister) may articulate key priorities, themes and issues from time to time.

4.5 HHSs have a responsibility to ensure that the delivery of Public Sector Health Services in Queensland is consistent with these strategic directions and priorities.

4.6 The Parties will collectively identify, develop, implement and evaluate strategies that support the delivery of priorities identified by the Minister, and which align with a Value-Based Healthcare approach to the delivery of Health Services.

4.7 In accordance with section 9 of the *Financial and Performance Management Standard 2009*, HHSs are required to develop a strategic plan. The HHS's strategic plan will reflect local priorities and will be developed considering the shared Queensland priorities outlined in the Queensland Government's objectives for the community, the Ministers' articulated priorities and *My health, Queensland's future: Advancing health 2026*.

4.8 The Parties have a collective responsibility to contribute to a sustainable Public Sector Health System in Queensland. Planning and delivery of Health Services will be aligned with the system planning agenda set out in *Queensland Health System Outlook to 2026 for a sustainable health service* in order to ensure a coordinated, system-wide response to growing demand for Health Services.

4.9 In delivering Health Services, HHSs are required to meet the applicable conditions of all national agreements and national partnership agreements between the Queensland Government and the Commonwealth Government and commitments under any related implementation plans.

4.10 This Service Agreement is underpinned by and is to be managed in line with the following supporting documents:

(a) Queensland Health System Outlook to 2026 for a sustainable health service;

(b) Performance and Accountability Framework 2020/21; and

(c) Purchasing Policy and Funding Guidelines 2020/21.

5. Objectives of the Service Agreement

This Service Agreement is designed to:

(a) specify the Health Services, teaching, research and other services to be provided by the HHS;

(b) specify the funding to be provided to the HHS for the provision of the services;

(c) specify the Performance Measures for the provision of the services;

(d) specify the performance and other data to be provided by the HHS to the Chief Executive;

(e) provide a platform for greater public accountability; and

(f) facilitate the achievement of State and Commonwealth Government priorities, services, outputs and outcomes while ensuring local input.

6. Scope

6.1 This Service Agreement outlines the services that the Department will purchase from the HHS during the period of this Service Agreement.

6.2 This Service Agreement does not cover the provision of clinical and non-clinical services by the Department, including the Queensland Ambulance Service, to the HHS. Separate arrangements will be established for those services provided by Health Support Queensland and eHealth Queensland.

7. Performance and Accountability Framework

7.1 The Performance and Accountability Framework sets out the framework within which the Department, as the overall manager of Public Health System Performance, monitors and assesses the performance of Public Sector Health Services in Queensland. The systems and processes employed for this purpose include, but are not limited to, assessing and monitoring HHS performance, reporting on HHS performance and, as required, intervening to manage identified performance issues.

7.2 During 2020/21 the Performance and Accountability Framework will support delivery of the *Queensland Health System Priorities 2020/21* which focus on realising positive changes to the Public Sector Health System through providing sustainable, timely, safe and high-quality Health Services in the right setting whilst remaining ready to respond to the COVID-19 pandemic.

7.3 The Performance and Accountability Framework uses Performance Measures to monitor the extent to which HHSs are delivering the high-level objectives set out in this Service Agreement. The Key Performance Indicators and other measures of performance against which the HHS will be assessed and benchmarked are detailed in Schedule 3 of this Service Agreement.

7.4 The Parties agree to constructively implement the Performance and Accountability Framework.

8. Period of this Service Agreement

8.1 This Service Agreement commences on the Effective Date and expires on 30 June 2022. The Service Agreement framework is in place for three years in order to provide HHSs with a level of guidance regarding funding and purchased activity for the outer years.

8.2 In this Service Agreement, references to years are references to the period commencing on 1 July and ending on 30 June unless otherwise stated.

8.3 Using the provisions of the *Hospital and Health Boards Act 2011* as a guide, the Parties will enter into funding and purchased activity negotiations for the following year six months before the end of the current year.

8.4 In accordance with the *Hospital and Health Boards Act 2011* the Parties will enter negotiations for the next Service Agreement at least six months before the expiry of the existing Service Agreement.

9. Amendments to this Service Agreement

9.1 Section 39 of the *Hospital and Health Boards Act 2011* requires that, if the Chief Executive or the HHS wish to amend the terms of a Service Agreement, the Party wishing to amend the Service Agreement must give written notice of the proposed amendment to the other Party.

9.2 The process for amending this Service Agreement is set out in Schedule 5 of this Service Agreement.

10. Publication of amendments

The Department will publish each executed Deed of Amendment within 14 days of the date of execution on www.health.qld.gov.au/system-governance/health-system/managing/default.asp.

11. Cessation of service delivery

11.1 The HHS is required to deliver the Health Services and other services outlined in this Service Agreement for which funding is provided in Schedule 2. Any changes to service delivery must ensure maintenance of care and minimise disruptions to patients.

11.2 The Department and HHS may Terminate or temporarily Suspend a Health Service or other service by mutual agreement having regard to the following obligations:

(a) any proposed Termination or Suspension must be made in writing to the other Party;

(b) where it is proposed to Terminate or Suspend a Statewide Service, or a Regional Service, the HHSs which are in receipt of that service must also be consulted;

(c) the Parties must agree on a reasonable notice period following which Termination, or Suspension, will take effect; and

(d) patient needs, workforce implications, relevant government policy and HHS sustainability are to be considered.

11.3 The Department, in its role as the Queensland Public Health System manager:

(a) may, in its unfettered discretion, not support a requested Termination or Suspension and require the HHS to maintain the service; and

(b) will reallocate existing funding and activity for the Terminated or Suspended service inclusive of baseline Service Agreement funding and in-year growth funding on a pro-rata basis.

11.4 The HHS will:

(a) work with the Department to ensure continuity of care and a smooth transfer of the service to an alternative provider where this is necessary; and

(b) minimise any risk or inconvenience to patients associated with service Termination, Suspension or transfer.

11.5 In the event that a sustainable alternative provider cannot be identified, and this is required, the service and associated patient cohort will continue to remain the responsibility of the HHS.

12. Commencement of a new service

12.1 In the event that the HHS wishes to commence providing a new Health Service, the HHS will notify the Department in writing in advance of commencement.

12.2 The Department will provide a formal response regarding the proposed new Health Service to the HHS in writing. The Department may not agree to purchase the new Health Service or to provide funding on either a recurrent or non-recurrent basis.

12.3 In the event that a change to an established Referral Pathway is proposed which would result in the direction of patient referrals to an alternative HHS on a temporary or a permanent basis:

(a) the new Referral Pathway must be agreed by all impacted HHSs prior to its implementation; and

(b) following agreement of the new Referral Pathway, if there is an identifiable and agreed impact to funding the Department will redistribute funding and activity between HHSs in alignment with new Referral Pathway.

13. Provision of data to the Chief Executive

The HHS will provide to the Chief Executive the performance data and other data, including data pursuant to ad hoc requests, set out in Schedule 4 of this Service Agreement in accordance with the Schedule, including in relation to the form, manner and the times required for the provision of data.

14. Dispute resolution

14.1 The dispute resolution process set out below is designed to resolve disputes which may arise between the Parties to this Service Agreement in a final and binding manner.

14.2 These procedures and any disputes addressed or to be addressed by them are subject to the provisions of the *Hospital and Health Boards Act 2011*, including in respect of any directions issued under that legislation or by Government in respect of any dispute.

14.3 Resolution of disputes will be through a tiered process commencing with the Performance Review Meeting and culminating, if required, with the Minister, as illustrated in Figure 1.

14.4 Use of the dispute resolution process set out in this clause should only occur following the best endeavours of both Parties to agree a resolution to an issue at the local level. The dispute resolution process is not intended for the resolution of ongoing issues or performance related issues. At each stage of the dispute resolution process, the Parties agree to cooperate.

14.5 If a dispute arises in connection with this Service Agreement (including in respect of interpretation of the terms of this Service Agreement), then either Party may give the other a written Notice of Dispute.

14.6 The Notice of Dispute must be provided to the D-SA Contact Person if the notice is being given by the HHS and to the HHS-SA Contact Person if the notice is being given by the Department.

14.7 The Notice of Dispute must contain the following information:

(a) a summary of the matter in dispute;

(b) an explanation of how the Party giving the Notice of Dispute believes the dispute should be resolved and reasons to support that belief;

(c) any information or documents to support the Notice of Dispute; and

(d) a definition and explanation of any financial or Service delivery impact of the dispute.

Figure 1 Dispute resolution process

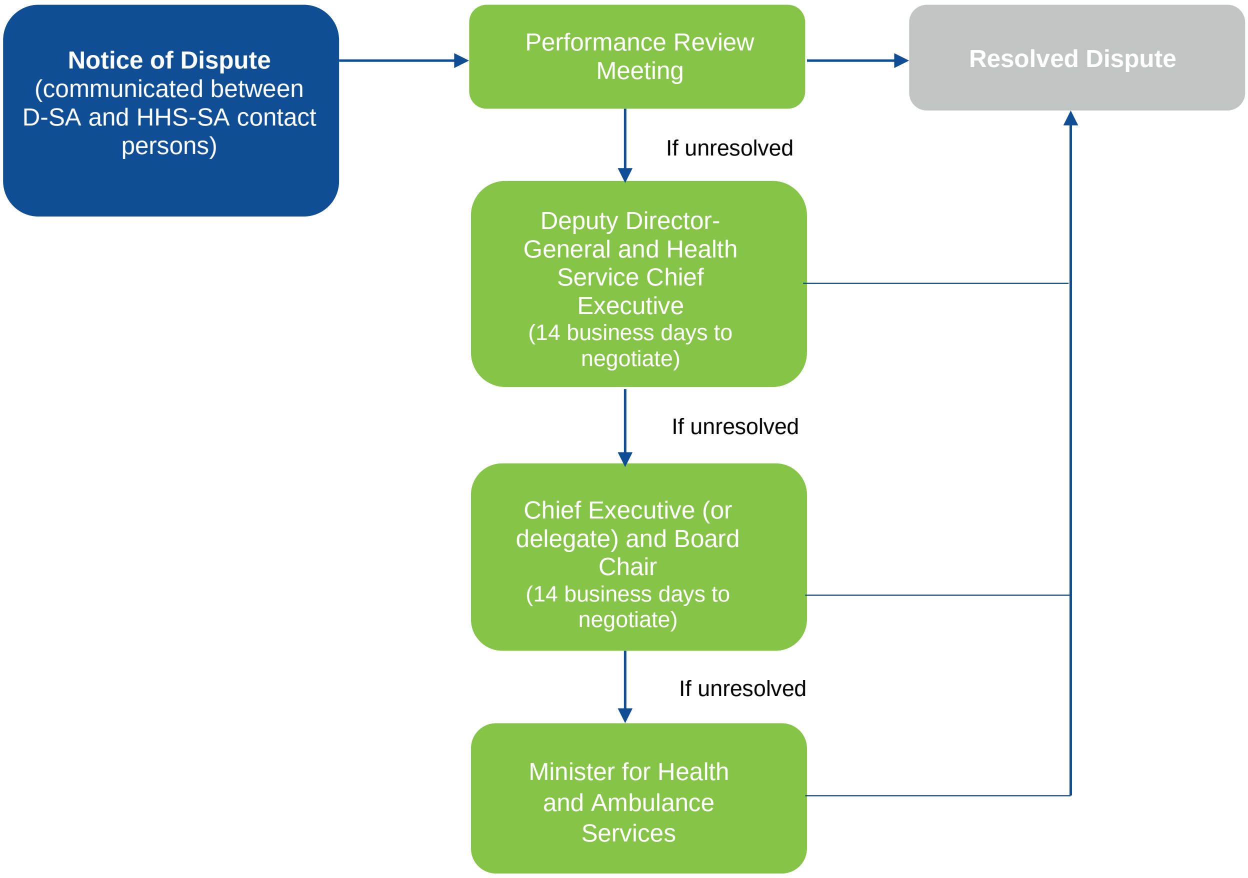

14.8 Resolution of a dispute

(a) Resolution of a dispute at any level is final. The resolution of the dispute is binding on the Parties but does not set a precedent to be adopted in similar disputes between other Parties.

(b) The Parties agree that each dispute (including the existence and contents of each Notice of Dispute) and any exchange of information or documents between the Parties in connection with the dispute is confidential and must not be disclosed to any third party without the prior written consent of the other Party, other than if required by law and only to the extent required by law.

14.9 Continued performance

Notwithstanding the existence of one or more disputes, the HHS must continue to perform and comply with this Service Agreement to the best of their abilities given the circumstances.

14.10 Disputes arising between Hospital and Health Services

(a) In the event of a dispute arising between two or more HHSs (an Inter-HHS Dispute), the process set out in Figure 2 will be initiated. Resolution of Inter-HHS Disputes will be through a tiered process, commencing with local resolution and culminating if required with formal and binding arbitration by the Minister under the provisions of the *Hospital and Health Boards Act 2011*, section 44.

(b) If the HHS wishes to escalate a dispute, the HHS will be expected to demonstrate that best endeavours (including Chair and Board involvement) to resolve the dispute between all parties at an informal and local level have taken place.

(c) Management of inter-HHS relationships should be informed by the following principles:

(i) HHSs should maintain (for both the base level of funding and growth) the proportion of out of HHS work undertaken unless as a result of agreed repatriation of patients.

(ii) All HHSs manage patients from their own catchment population if it is within their clinical capability to do so as specified by the Clinical Services Capability Framework.

(iii) Where it is proposed that a Health Service move from one HHS to another, agreement between the respective Health Service Chief Executives will be secured prior to any change in patient flows. Once agreed, funding will follow the patient.

(iv) All HHSs abide by the agreed dispute resolution process.

(v) All HHSs operate in a manner which is consistent with the health system principles and objectives as set out in the National Health Reform Agreement and the *Hospital and Health Boards Act 2011*.

Figure 2 Inter-HHS dispute resolution process

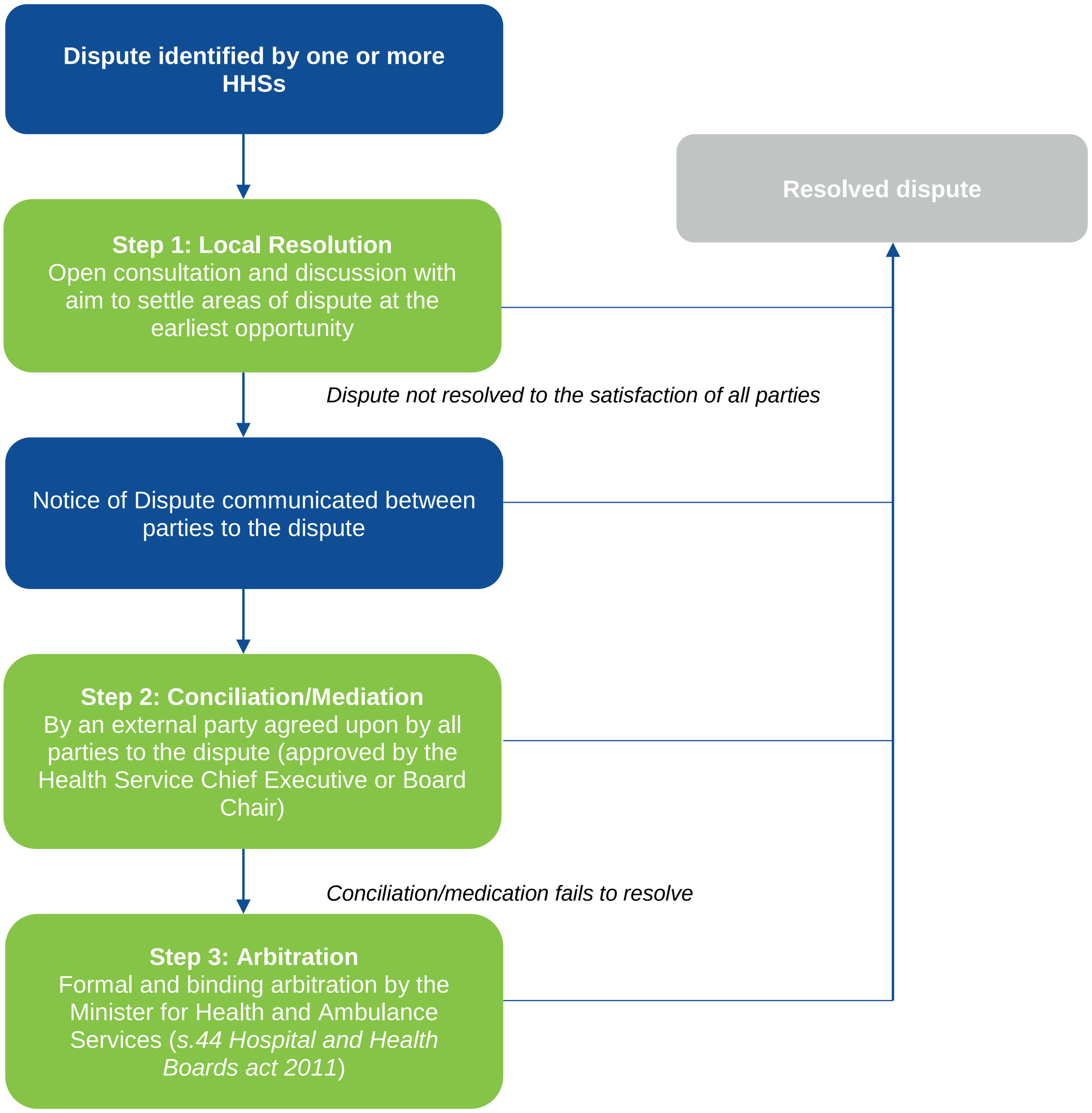

15. Force Majeure

15.1 If a Party (Affected Party) is prevented or hindered by Force Majeure from fully or partly complying with any obligation under this Service Agreement, that obligation may (subject to the terms of this Force Majeure clause) be suspended, provided that if the Affected Party wishes to claim the benefit of this Force Majeure clause, it must:

(a) give prompt written notice of the Force Majeure to the other Party of:

(i) the occurrence and nature of the Force Majeure;

(ii) the anticipated duration of the Force Majeure;

(iii) the effect the Force Majeure has had (if any) and the likely effect the Force Majeure will have on the performance of the Affected Party's

obligations under this Service Agreement; and

(iv) any disaster management plan that applies to the party in respect of the Force Majeure.

(b) use its best endeavours to resume fulfilling its obligations under this Service Agreement as promptly as possible; and

(c) give written notice to the other Party within five days of the cessation of the Force Majeure.

15.2 Without limiting any other powers, rights or remedies of the Chief Executive, if the Affected Party is the HHS and the delay caused by the Force Majeure continues for more than 14 days from the date that the Chief Executive determines that the Force Majeure commenced, the Chief Executive may give directions to the HHS regarding the HHS's performance or non-performance of this Service Agreement during the Force Majeure and the HHS must comply with that direction.

15.3 Neither Party may terminate this Service Agreement due to a Force Majeure event.

16. Hospital and Health Service accountabilities

16.1 Without limiting any other obligations of the HHS, it must comply with:

(a) the terms of this Service Agreement;

(b) all legislation applicable to the HHS, including the *Hospital and Health Boards Act 2011*;

(c) all Cabinet decisions applicable to the HHS;

(d) all Ministerial directives applicable to the HHS;

(e) all agreements entered into between the Queensland and Commonwealth governments applicable to the HHS;

(f) all regulations made under the *Hospital and Health Boards Act 2011*;

(g) all Industrial Instruments applicable to the HHS; and

(h) all health service directives applicable to the HHS.

16.2 The HHS will ensure that the accountabilities set out in Schedule 1 of this Service Agreement are met.

17. Department accountabilities

17.1 Without limiting any other obligations of the Department, it must comply with:

(a) the terms of this Service Agreement;

(b) the legislative requirements as set out within the *Hospital and Health Boards Act 2011*;

(c) all regulations made under the *Hospital and Health Boards Act 2011*; and

(d) all Cabinet decisions applicable to the Department.

17.2 The Department will work in collaboration with HHSs to ensure the Public Sector Health System delivers high quality hospital and other Health Services to persons in Queensland having regard to the principles and objectives of the national health system. In support of realising this objective, in accordance with section 5 of the *Hospital and Health Boards Act 2011* the Department will:

(a) provide overall management of the Queensland Public Sector Health System including health system planning, coordination and standard setting;

(b) provide the HHS with funding specified under Schedule 2 of this Service Agreement;

(c) provide and maintain payroll and rostering systems to the HHS unless agreed otherwise between the Parties;

(d) operate 13 HEALTH as a first point of contact for health advice with timely HHS advice and information where appropriate to local issues; and

(e) balance the benefits of a local and system-wide approach.

17.3 The Department will endeavour to purchase services in line with Clinical Prioritisation Criteria, where these have been developed, in order to improve equity of access and reflect the scope of publicly funded services.

17.4 The Department will maintain a public record of the Clinical Service Capability Framework levels for all public facilities based on the information provided by HHSs.

17.5 **Workforce management**

The Chief Executive agrees to appoint Health Service Employees to:

(a) perform work for the HHS for the purpose of enabling the HHS to perform its functions and exercise powers under the *Hospital and Health Boards Act 2011;* and

(b) deliver the services specified in this Service Agreement.

17.6 The Chief Executive, whether in the capacity of a Person Conducting a Business or Undertaking as an employer or as a building owner or occupier, will consult, cooperate and coordinate with the HHS to ensure legal compliance with the *Work Health and Safety Act 2011* and other legislation as it applies to the scope, nature and location of operations associated with this Service Agreement. This includes, but is not limited to:

(a) codes of practice;

(b) electrical safety legislation;

(c) building and fire safety legislation; and

(d) workers' compensation legislation.

18. Insurance

18.1 The HHS must hold and maintain for the period of this Service Agreement the types and levels of insurances that the HHS considers appropriate to cover its obligations under this Service Agreement.

18.2 Without limiting the types and levels of insurances that the HHS considers appropriate, any insurance policies taken out by the HHS under this clause must include appropriate coverage for the following:

(a) public and product liability insurance;

(b) professional indemnity insurance; and

(c) workers' compensation insurance in accordance with the *Workers' Compensation and Rehabilitation Act 2003* (Qld).

18.3 The HHS will be deemed to comply with its requirements under clauses 18.1 and 18.2(a) and 18.2(b) if it takes out and maintains a current insurance policy with the Queensland Government Insurance Fund.

18.4 Any insurance policies held by the HHS pursuant to this clause must be effected with an insurer that is authorised and licensed to operate in Australia.

18.5 The HHS must maintain a current register of all third-party guarantees.

18.6 The HHS must, if requested by the Department, promptly provide a sufficiently detailed certificate of currency and/or insurance and policy documents for each insurance policy held by the HHS pursuant to this clause.

18.7 The HHS warrants that any exclusions and deductibles that may be applicable under the insurance policies held pursuant to this clause will not impact on the HHS's ability to meet any claim, action or demand or otherwise prejudice the Department's rights under this Service Agreement.

18.8 The HHS must immediately advise the Department if any insurance policy, as required by this clause, is materially modified or cancelled.

19. Indemnity

19.1 The HHS indemnifies the Department against all and any liabilities, claims, actions, demands, costs and expenses made by any person which may be brought against or made upon or incurred by the Department arising directly or indirectly from or in connection with any of the following:

(a) any wilful, unlawful or negligent act or omission of the HHS, a Health Service Employee, Health Executive, Senior Health Service Employee or an officer, employee or agent working for the HHS in the course of the performance or attempted or purported performance of this Service Agreement;

(b) any penalty imposed for breach of any applicable law in relation to the HHS's performance of this Service Agreement; and

(c) a breach of this Service Agreement,

except to the extent that any act or omission by the Department caused or contributed to the liability, claim, action, demand, cost or expense.

19.2 The indemnity referred to in this clause will survive the expiration or termination of this Service Agreement.

20. Indemnity arrangements for officers, employees and agents

20.1 Indemnity arrangements for officers, employees or agents working for the Public Sector Health System are administered in accordance with the following policy documents, as amended from time to time:

(a) Indemnity for Queensland Health Medical Practitioners HR Policy I2 (QH-POL-153:2014); and

(b) Queensland Government Indemnity Guideline.

20.2 The costs of indemnity arrangements provided for Health Service Employees, Health Executives, Senior Health Service Employees, or officers, employees or agents working for the HHS are payable by the HHS.

21. Legal proceedings

21.1 This clause applies if there is any demand, claim, liability or legal proceeding relating to assets, contracts, agreements or instruments relating to the HHS, whether or not they are:

(a) transferred to an HHS under section 307 of the *Hospital and Health Boards Act 2011*; or

(b) retained by the Department.

21.2 Subject to any law, each party must (at its own cost) do all things, execute such documents and share such information in its possession and control that is relevant, and which is reasonably necessary, to enable the other party to institute or defend (as the case may be) any demand, claim, liability or legal proceeding.

22. Sub-contracting

22.1 The Parties acknowledge that the HHS may sub-contract the provision of Health Services and other services that are required to be performed by the HHS under this Service Agreement.

22.2 The HHS must ensure that any sub-contractor who has access to confidential information (as defined in section 139 of the *Hospital and Health Boards Act 2011*) and/or personal information (as defined in section 12 of the *Information Privacy Act 2009*) complies with obligations no less onerous than those imposed on the HHS.

22.3 The HHS agrees that the sub-contracting of services:

(a) will not transfer responsibility for provision of the services to the sub-contractor; and

(b) will not relieve the HHS from any of its liabilities or obligations under this Service Agreement, including but not limited to obligations concerning the provision of data in accordance with Schedule 4 (Data Supply Requirements).

23. Counterparts

23.1 This Service Agreement may be executed in two or more identical copy counterparts, each of which together will be deemed an original, but all of which together will constitute one and the same instrument.

23.2 In the event that any signature executing this Service Agreement or any part of this Service Agreement is delivered by facsimile transmission or by scanned e-mail delivery of a '.pdf' format data file or equivalent, the signature will create a valid and binding obligation of the Party executing (or on whose behalf the signature is executed) with the same force and effect as if the signature page were an original.

23.3 For execution under this clause 23 to be valid the entire Service Agreement upon execution by each individual Party must be delivered to the remaining parties.

Execution

A. The terms of this Service Agreement were agreed under the provisions set out in the *Hospital and* Health *Boards Act,* section 35 on 27 June 2019, and were subsequently amended by the Deed of Amendment entered into pursuant to section 39 of the *Hospital and Health Boards Act 2011* and executed on 10 January 2020; 22 May 2020; 25 June 2020 and 29 July 2020.

B. This revised Service Agreement consolidates amendments arising from:

- 2019/20 Amendment Window 2 (in-year variation);
- 2019/20 Amendment Window 3 (in-year variation);
- 2020/21 Amendment Window 1 (annual budget build); and
- May 2020 Extra-ordinary Amendment Window.

C. Execution source documents are available on the service agreement website https://www.health.qld.gov.au/system-governance/health-system/managing/agreements-deeds.

Schedule 1 HHS Accountabilities

1. Purpose

Without limiting any other obligations of the HHS, this Schedule 1 sets out the key accountabilities that the HHS is required to meet under the terms of this Service Agreement.

2. Registration, credentialing and scope of clinical practice

2.1 The HHS must ensure that:

(a) all persons (including off site reporting radiologists) who provide a clinical service for which there is a national or Queensland legal requirement for registration, have and maintain current registration throughout their employment and only practise within the scope of that registration;

(b) all persons who perform roles for which eligibility for membership of a professional association is a mandatory requirement, have and maintain current eligibility of membership of the relevant professional association throughout their employment in the role; and

(c) all persons who provide a clinical service, and who fall within the scope of current credentialing policies (i.e. including medical, dental, nursing, midwifery and allied health), have a current scope of clinical practice and practise within that scope of clinical practice (which includes practising within their registration conditions and within the scope of the Clinical Services Capability Framework of the facility/s at which the service is provided).

2.2 Confirmation of registration and/or professional memberships is to be undertaken in accordance with the processes outlined in 'Health Professionals Registration: medical officers, nurses, midwives and other health professionals HR Policy B14 (QH-POL-147:2016)', as amended from time to time.

3. Clinical Services Capability Framework

3.1 The HHS must ensure that:

(a) all facilities have undertaken a baseline self-assessment against the Clinical Services Capability Framework (version 3.2);

(b) the Department is notified when a change to the Clinical Services Capability Framework baseline self-assessment occurs through the established public hospital Clinical Services Capability Framework notification process; and

(c) in the event that a Clinical Services Capability Framework module is updated or a new module is introduced, a self-assessment is undertaken against the relevant module and submitted to the Department.

3.2 The HHS is accountable for attesting to the accuracy of the information contained in any Clinical Services Capability Framework self-assessment submitted to the Department.

4. Clinical Prioritisation Criteria

4.1 The HHS must ensure that:

(a) processes for access to specialist surgical and medical services in line with Clinical Prioritisation Criteria are implemented, where these have been developed, in order to improve equity of access to specialist services; and

(b) General Practice Liaison Officer and Business Practice Improvement Officer programs are maintained in order to deliver improved access to specialist outpatient services, including through (but not limited to) their contribution to the development and implementation of Statewide Clinical Prioritisation Criteria.

5. Service delivery

5.1 The HHS will work with collaboratively with other healthcare service providers to ensure that an integrated pathway of care is in place for patients. This will include, but is not limited to:

(a) other HHSs;

(b) Primary Care providers;

(c) non-government organisations; and

(d) private providers.

5.2 The HHS must ensure that:

(a) the Health Services and other outlined in this Service Agreement, for which funding is provided in Schedule 2 ‘Funding and Purchased Activity and Services’ continue to be provided;

(b) the obligations regarding the payment and planning for blood and blood products and best practice as set out under the National Blood Agreement are fulfilled for the facilities for which funding is provided; and

(c) the *Queensland Organ Donation Strategy 2018-2020* is implemented in order to support an increase in organ donation rates in Queensland.

5.3 Through accepting the funding levels defined in Schedule 2 of this Service Agreement, the HHS accepts responsibility for the delivery of the associated programs and reporting requirements to State and Commonwealth bodies as defined by the Department.

6. Accreditation

6.1 All Queensland public hospitals, day procedure services and health care centres (howsoever titled) managed within the framework of HHSs are to maintain accreditation under the Australian Health Service Safety and Quality Accreditation Scheme[1].

6.2 Accreditation will be assessed against the National Safety and Quality Health Service standards[2] (NSQHS) second edition.

6.3 Residential aged care facilities will maintain accreditation by the Aged Care Quality and Safety Commission (ACQSC).

6.4 General practices owned or managed by the HHS are to be externally accredited in accordance with the current edition of the Royal Australian College of General Practitioners (RACGP) accreditation standards and in line with the National General Practice Accreditation Scheme.

6.5 For the purpose of accreditation, the performance of the HHS against the NSQHS and the performance of general practices owned or managed by the HHS against the RACGP accreditation standards can only be assessed by accrediting agencies that are approved by the Australian Commission on Safety and Quality in Healthcare (ACSQHC).

6.6 The HHS will select their accrediting agency from among the approved accrediting agencies. The ACSQHC and the RACGP provide a list of approved accrediting agencies which are published on their respective websites (www.safetyandquality.gov.au and www.racgp.org.au.

6.7 If the HHS does not meet the NSQHS standards accreditation requirements, the HHS has 60 days to address any not met actions. If the HHS does not meet the other accreditation standards requirements (RACGP and ACQSC), a remediation period will be defined by the accrediting agency.

6.8 Following assessment against NSQHS, ACQSC and RACGP standards, the HHS will provide to the Executive Director, Patient Safety and Quality Improvement Service, Department.

(a) immediate advice if a significant patient risk (one where there is a high probability of a substantial and demonstrable adverse impact for patients) is identified during an onsite visit, also identifying the plan of action and timeframe to remedy the issue as negotiated between the surveyors/assessors and/or the respective accrediting agency and the HHS;

(b) a copy of any ‘not met’ reports within two days of receipt of the report by the HHS;

(c) the accreditation report within seven days of receipt of the report by the HHS; and

(d) immediate advice should any action be rated not-met by the accrediting agency following the remediation period of an accreditation event, resulting in the facility or service not being accredited. Responsive regulatory processes may be enacted under clause 7 below.

[1] www.safetyandquality.gov.au/our-work/assessment-to-the-nsqhs-standards/australian-health-service-safety-and-quality-accreditation-scheme/
[2] www.safetyandquality.gov.au/our-work/assessment-to-the-nsqhs-standards/

6.9 The award recognising that the facility or service has met the required accreditation standards will be issued by the assessing accrediting agency for the period determined by their respective accreditation scheme.

6.10 The HHS will apply to an approved accrediting agency for a re-accreditation assessment prior to the expiry of their current accreditation period.

6.11 Where the HHS funds non-government organisations to deliver health and human services the HHS will ensure, from the Effective Date of this Service Agreement, that:

(a) within 12 months HHS procurement processes and service agreements with contracted non-government organisations specify the quality accreditation requirements for mental health services as determined by the Department; and

(b) as the quality accreditation requirements for subsequent funded service types are determined by the Department, procurement processes and service agreements with contracted non-government organisations reflect these requirements within 12 months of their formal communication by the Department to HHSs.

7. Responsive regulatory process for accreditation

7.1 A responsive regulatory process is utilised in the following circumstances:

(a) where a significant patient risk is identified by a certified accrediting agency during an accreditation process; and/or

(b) where an HHS has failed to address ‘not met’ actions of the specified standards within required timeframes.

7.2 An initial regulatory response will begin with a process of verifying the scope, scale and implications of the reported issues, a review of documentation, and may include one or more site visits by nominated specialty experts.

7.3 The regulatory process may include one or a combination of the following actions:

(a) seek further information from the HHS;

(b) request a progress report for the implementation of an action plan;

(c) escalate non-compliance and/or risk to the Performance Review Meeting;

(d) provide advice, information on options or strategies that could be used to address the non-met actions within a designated time frame; and/or

(e) connect the hospital to other hospitals that have addressed similar deficits or have exemplar practice in this area.

7.4 In the case of serious or persistent non-compliance and where required action is not taken by the HHS the response may be escalated. The Department may undertake one or a combination of the following actions:

(a) restrict specified practices/activities in areas/units or services of the HHS where the specified standards have not been met;

(b) suspend particular services at the HHS until the area/s of concern are resolved; and

(c) suspend all service delivery at a facility within an HHS for a period of time.

8. Achieving health equity for First Nations Queenslanders

8.1 The *Queensland Health Statement of Action towards Closing the Gap in Health Outcomes* is a commitment to addressing systemic barriers that may in any way contribute to preventing the achievement of health equity for all First Nations people. The statement is expected to mobilise renewed efforts and prompt new strategies for achieving health equity for First Nations Queenslanders.

8.2 The HHS will develop a Health Equity Strategy (previously referred to as the Closing the Gap Health Plan) to demonstrate the HHS's activities towards achieving health equity for First Nations people. The Health Equity Strategy will supersede the existing Closing the Gap Health Plan and act as the principal accountability mechanism between community and Government in the pursuit of Health Equity for First Nations Queenslanders.

8.3 The Health Equity Strategy will:

(a) be co-designed, co-developed and co-implemented by the First Nations community and the HHS; and

(b) demonstrate an evidence-based approach to priority setting.

8.4 The HHS will review the Health Equity Strategy at least once every three years and will publish the Health Equity Strategy in a way that allows it to be accessed by members of the public.

8.5 In line with the *Queensland Health Statement of Action towards Closing the Gap in Health Outcomes,* the HHS will ensure that commitment and leadership is demonstrated through implementing actions outlined in the Health Equity Strategy. The actions will, at a minimum:

(a) promote and provide opportunities to embed the representation of First Nations people in leadership, governance and the workforce;

(b) improve local engagement and partnerships between the HHS and First Nations people, communities and organisations to enable co-design, co-development and co-implementation;

(c) improve transparency, reporting and accountability in Closing the Gap progress; and

(d) demonstrate co-design, co-development, co-implementation and co-leadership of health programs and strategies.

8.6 The HHS will:

(a) establish and maintain an ongoing dialogue with the Department to progress and report on key actions taken and Health Service initiatives aligned to the *Queensland Health Statement of Action towards Closing the Gap in Health Outcomes*;

(b) establish and maintain an ongoing dialogue with the Department to progress and report on key actions taken and health service initiatives and strategies to attract,

recruit, support and retain a First Nations people workforce and workforce models commensurate to the HHS population and aligned to the benchmarks prescribed in the Workforce Diversity and Inclusion Strategy 2017-2022; and

(c) report publicly on progress against the Health Equity Strategy. Progress will be reported on an annual basis as a minimum.

9. Provision of Clinical Products/Consumables in outpatient settings

9.1 Upon discharge as an inpatient or outpatient, and where products/consumables are provided free of charge or at a subsidised charge, the Treating HHS will bear the initial costs of products/consumables provided to the patient/consumer as part of their care. These costs will be met by the Treating HHS for a sufficient period of time to ensure the patient/consumer incurs no disruption to their access to the Clinical Products/Consumables.

9.2 Unless otherwise determined by the HHS providing the Clinical Products/Consumables, ongoing direct costs (beyond an initial period following discharge as an inpatient) of the provided products/consumables will be borne by the Residential HHS of the outpatient/consumer.

9.3 Where guidelines exist (e.g. Guideline for Compression Garments for Adults with Lymphoedema: Eligibility, Supply and Costing and Guideline for Home Enteral Nutrition Services for Outpatients: Eligibility, Supply and Costing), standardised eligibility criteria and charges should apply.

9.4 Where a patient is supplied with medicines on discharge, or consequent to an outpatient appointment, that are being introduced to a patient's treatment, the Treating HHS will provide prescription(s) and an adequate initial supply. This will comprise:

(a) for medicines reimbursable under the Pharmaceutical Benefits Scheme (PBS), including the Section 100 Highly Specialised Drugs Program – the quantity that has been clinically-appropriately prescribed or the maximum PBS supply, whichever is the lesser; or

(b) for non-reimbursable medicines, one month's supply or a complete course of treatment, whichever is the lesser.

9.5 For medicines that are non-reimbursable under the PBS, and which are not included in the Queensland Health List of Approved Medicines (LAM), the Residential HHS will be responsible for ongoing supply, provided that the Treating HHS has provided the Residential HHS with documentary evidence of the gatekeeping approval at the Treating HHS for the non-LAM medicine. This evidence may be:

(a) a copy of the individual patient approval; or

(b) where the medicine is subject to a 'blanket approval' at the Treating HHS, a copy of the blanket approval, and a statement that the patient meets the criteria to be included under that approval.

9.6 This evidence is to be provided pro-actively to the Director of Pharmacy (or, for non-Pharmacist sites, the Director of Nursing and the HHS Director of Pharmacy) for the hospital nominated under clause 9.8 below.

9.7 For non-reimbursable medicines listed on the LAM for the condition being treated, the Residential HHS is responsible for ongoing supplies.

9.8 The Treating HHS will inform the patient about the ongoing supply arrangements and agree which hospital, within the patient's Residential HHS, they should attend for repeat supplies. The patient will be advised to contact the pharmacy at the nominated hospital regarding their requirements at least a week before attending for repeat supply.

9.9 PBS-reimbursable prescriptions issued by a public hospital may be dispensed at any other public hospital that has the ability to claim reimbursement. Patients may, in accordance with hospital policy, be encouraged to have their PBS prescriptions dispensed at a private pharmacy of their choice.

10. Capital, land, buildings, equipment and maintenance

10.1 **Capital**

(a) The HHS will:

(i) achieve annual capital expenditure within an acceptable variance to its allocation in the State's published Budget Paper 3 – Capital Statement, as specified in the capital expenditure performance KPI target.

(ii) record capital expenditure data in the capital intelligence portal each month. Data will be published through the System Performance Reporting (SPR) platform.

(iii) achieve all Priority Capital and Health Technology Equipment Replacement Program capital expenditure requirements and associated delivery milestones, as funded, and undertake all capital expenditure performance reporting requirements in the capital intelligence portal on a monthly basis.

(iv) comply with all other capital program reporting requirements, as identified in Schedule 4, Table 13.

10.2 **Asset Management**

(a) The Service Agreement includes funding provision for regular maintenance of the HHS's building portfolio.

(b) The Department has determined that a total sustainable budget allocation that equates to a minimum of 2.81% of the un-depreciated asset replacement value of the Queensland Public Health System's building portfolio is required to sustain the building assets to achieve expected life-cycles. The sustainable budget allocation is a combination of operational and capital maintenance funding.

(c) The HHS will conduct a comprehensive assessment of the maintenance demand for the HHS's building portfolio to ascertain the total maintenance funding

requirements of that portfolio. The assessment must identify the following for the portfolio:

(i) regulatory requirements;

(ii) best practice requirements;

(iii) condition-based requirements;

(iv) lifecycle planning requirements; and

(v) reactive maintenance estimates based on historical information, including backlog maintenance liabilities and risk mitigation strategies.

(d) The HHS will allocate an annual maintenance budget that reasonably takes into account the maintenance demand identified by the assessment in its reasonable considerations, without limiting the scope of such reasonable considerations including financial affordability linked to risk assessment. The annual maintenance budget will equate to either:

(i) 2.81% of the un-depreciated asset replacement value of the HHS's building portfolio; or

(ii) an alternative percentage amount determined by the HHSs as a result of its considerations.

(e) The HHS will submit an annual asset management and maintenance plan, approved by the Health Service Chief Executive, to the Department that:

(i) outlines the maintenance demand assessment undertaken by the HHS under Schedule 1, clause 10.2(c)

(ii) confirms the annual maintenance budget determined by the HHS under Schedule 1, clause 10.2(d)

(f) The HHS will submit an annual Statement of Building Portfolio Compliance to the Department for each year of the Term of this Service Agreement.

(g) The HHS will continue to proactively develop and address the recommendations within the final Asset Management Capability Report that was issued to the HHS as part of the transfer notice process.

10.3 **Property**

(a) The HHS will ensure building and infrastructure assets are managed in accordance with the specifications of any relevant transfer notices published as a gazette notice by the Minister under section 273A of the *Hospital and Health Boards Act 2011.*

(b) For land, buildings and parts of buildings where the Department is, or is intended to be, the exclusive occupier under specific occupancy or ground leases implemented pursuant to clauses 1.7 (c) and 1.8 respectively (where applicable) of a transfer notice, the Department is deemed to be in control of that land, building or part of a building for the purpose of work health and safety law.

10.4 Nothing in clause 10.3(b) of Schedule 1:

(a) removes any work health and safety responsibilities shared with another party or parties in accordance with work health and safety law; or

(b) limits the arrangements for the provision of work health and safety services provided in clause 11.

11. Occupational health and safety

11.1 The HHS, whether in the capacity of a Person Conducting a Business or Undertaking as an employer or as a building owner or occupier, will consult, cooperate and coordinate to ensure legal compliance with the *Work Health and Safety Act 2011* and other legislation as it applies to the scope, nature and location of operations associated with this Service Agreement. This includes, but is not limited to:

(a) codes of practice;

(b) electrical safety legislation;

(c) building and fire safety legislation; and

(d) workers' compensation legislation.

11.2 The HHS will establish, implement and maintain a health and safety management system which conforms to recognised health and safety management system standard AS/NZS 4801 *Occupational Health and Safety Management System* or ISO45001 *Occupational Health and Safety Management Systems* or another standard as agreed with the Chief Executive.

11.3 The HHS will monitor health and safety performance and will provide to the Chief Executive reports of a type, and at the intervals, agreed between the Parties, or as reasonably specified by the Chief Executive.

11.4 The Chief Executive will monitor health and safety performance at the system level. Where significant health and safety risks are identified, or performance against targets is identified as being outside tolerable levels, the Chief Executive may request further information from the HHS to address the issue(s) and/or make recommendations for action.

12. Workforce management

12.1 Subject to a delegation by the Chief Executive under section 46 of the *Hospital and Health Boards Act 2011*, the HHS is responsible for the day-to-day management (the HR Management Functions) of the Health Service Employees provided by the Chief Executive to perform work for the HHS under this Service Agreement.

(a) The HHS will exercise its decision-making power in relation to all HR Management Functions which may be delegated to it by the Chief Executive under section 46 of the *Hospital and Health Boards Act 2011*, in respect of the Health Service Employees, in a lawful and reasonable manner and with due diligence, and in accordance with:

(i) terms and conditions of employment specified by the Department in accordance with section 66 of the *Hospital and Health Boards Act 2011;*

(ii) health service directives, issued by the Chief Executive under section 47 of the *Hospital and Health Boards Act 2011*;

(iii) health employment directives, issued by the Chief Executive under section 51A of the *Hospital and Health Boards Act 2011;*

(iv) any policy document that applies to the Health Service Employee;

(v) any Industrial Instrument that applies to the Health Service Employee;

(vi) the relevant HR delegations manual; and

(vii) any other relevant legislation.

12.2 The HHS must ensure that Health Service Employees are suitably qualified to perform their required functions.

12.3 Persons appointed in an HHS as a Health Executive or Senior Health Service Employees are employees of the HHS

12.4 All HHSs will provide to the Chief Executive human resource, workforce, and health and safety reports of a type, and at the intervals, agreed between the Parties, or as reasonably specified by the Chief Executive.

13. Medically authorised ambulance transports

13.1 The HHS will:

(a) utilise the Queensland Ambulance Service (QAS) for all road ambulance services not provided by the HHS. This includes both paramedic level and patient transport level services where the patient requires clinical care;

(b) follow the *Medically Authorised Ambulance Transports Operational Standards* when utilising QAS services; and

(c) ensure that performance data for ambulance services authorised by the HHS is collected and provided to the Department in line with agreed data supply requirements.

Schedule 2 Funding, purchased activity and services

1. Purpose

This Schedule 2 sets out:

(a) The activity purchased by the Department from the HHS (Table 4, Table 6 and Table 8);

(b) The funding provided for delivery of the purchased activity (Table 4; Table 5; Table 6; and Table 7);

(c) Specific funding commitments (Table 1);

(d) The criteria and processes for financial adjustments associated with the delivery of purchased activity and specific funding commitments;

(e) The sources of funding that this Service Agreement is based on and the manner in which these funds will be provided to the HHS (Table 3); and

(f) An overview of the purchased Health Services and other services which the HHS is required to provide throughout the period of this Service Agreement.

2. Delivery of purchased activity

2.1 The Department and the HHS will monitor actual activity against purchased levels.

2.2 The HHS has a responsibility to actively monitor variances from purchased activity levels and will notify the Department immediately via the D-SA Contact Person as soon as the HHS becomes aware of significant variances.

2.3 The HHS will also notify the Department of deliberate changes to the consistent recording of activity within year that would result in additional activity being recorded for existing Health Services.

2.4 If the HHS wishes to move activity between purchased activity types and levels, for example, activity moving from outpatients to inpatients or from one inpatient Service Related Group (SRG) to another, the HHS must negotiate this with the Department based on a sound needs based rationale.

2.5 With the exception of the programs, services and projects that are specified in Table 1, during 2020/21 no financial adjustment will be applied where the HHS is unable to deliver or exceeds the activity that has been funded, in recognition of the Commonwealth Government's treatment of the National Health Reform Agreement to support the response to the COVID-19 pandemic.

2.6 The activity purchased through this Service Agreement for 2020/21 is based on the activity purchased recurrently in 2019/20 and includes the productivity dividend.

2.7 The activity purchased in the Service Agreement for 2021/22 will be based on the activity purchased recurrently in 2020/21 including the productivity dividend.

2.8 Any resulting changes to funding and purchased activity levels will be actioned through the processes outlined in Schedules 2 and 5 of this Service Agreement.

2.9 The Department is required to report HHS activity data to the Independent Hospital Pricing Authority and the Administrator of the National Health Funding Pool. The HHS will maintain its efforts to accurately record the delivered activity and submit this data to the Department in accordance with the requirements set out in Schedule 4.

2.10 The HHS should refer to the supporting document to this Service Agreement 'Healthcare Purchasing Policy and Funding Guidelines 2020/21' for details regarding the calculation of Weighted Activity Units. Supporting documents are available on-line as detailed in Appendix 1.

3. Financial adjustments

3.1 **Specific funding commitments**

(a) As part of the Service Agreement Value, the services, programs and projects set out in Table 1 have been purchased by the Department from the HHS. These services will be the focus of detailed monitoring by the Department.

(b) The HHS will promptly notify the D-SA Contact Person if the HHS forecasts an inability to achieve commitments linked to the specific funding commitments included in Table 1.

(c) On receipt of any notice under clause 3.1(b) of Schedule 2, it is at the discretion of the Chief Executive (or delegate) to withdraw allocated funding pro rata to the level of under delivery if the program is not being delivered according to the program objective or is not being delivered in full.

(d) If the Chief Executive (or delegate) decides to withdraw allocated funding, the Chief Executive (or delegate) will immediately issue an Adjustment Notice to the HHS-SA Contact Person confirming any adjustment that has been made in accordance with this clause 3.1 of Schedule 2. The Adjustment Notice will be issued through the process set out in clause 3.4 or 3.5 of Schedule 5.

(e) Following receipt of an Adjustment Notice under clause 3.1(d) of Schedule 2, the Parties will comply with the Adjustment Notice and immediately take steps necessary to give effect to the requirements of that Adjustment Notice.

(f) The Parties acknowledge that adjustments made under this clause 3.1 of Schedule 2 may vary the Service Agreement Value and/or a specific value recorded in Table 1.

(g) Where the Service Agreement Value and/or a specific value recorded in Table 1 is varied, the variation will be recorded in a Deed of Amendment issued following the next available Amendment Window.

Table 1 Specific Funding Commitments

Service/Program/Project	Funding	Activity	Timeframe	Conditions
Making Tracks towards closing the gap in health outcomes for Indigenous Queenslanders by 2033 - Investment Strategy 2018-21	*$2,173,466*<br>$1,805,193	*0*<br>0	*2019/20*<br>2020/21	The HHS will deliver the initiatives and outcomes outlined in the performance requirements issued by the Aboriginal and Torres Strait Islander Health Division in memorandum C-ECTF-19/5767.<br>Funding may be adjusted and/or unspent funds redirected or recovered where project performance requirements are not met.
• Making Tracks: Collaboration in Health (MaTCH)	*$30,000*	*0*	*2019/20*	The HHS will deliver the initiatives and outcomes outlined in the performance requirements issued by the Aboriginal and Torres Strait Islander Health Division in memorandum C-ECTF-20/1314.<br>Funding may be adjusted and/or unspent funds redirected or recovered where project performance requirements are not met.
North Queensland Aboriginal and Torres Strait Islander Sexually Transmissible Infections Action Plan 2016-2021	*$1,489,326*<br>$1,489,326	*0*<br>0	*2019/20*<br>2020/21	The HHS will implement and support the required actions under the *North Queensland Aboriginal and Torres Strait Islander Sexually Transmissible Infections Action Plan 2016-2021* including the delivery of initiatives and outcomes outlined in memorandum C-ECTF-19/5767.<br>Funding may be adjusted and/or unspent funds redirected or recovered where project performance requirements are not met.
North Queensland Renal Funding				
• Weipa Renal Dialysis	*$287,000*<br>$287,000<br>$287,000	*0*<br>0<br>0	*2019/20*<br>2020/21<br>2021/22	Weipa - funds may be withdrawn on a pro rata basis should the satellite unit not transition from a self-care facility to a semi supported satellite unit.
• Bamaga Renal Dialysis	*$571,000*<br><br>*$310,000*	*120 WAUs (Q21)*<br><br>*64 WAUs (Q22)*	*2018/19*<br><br>*2019/20*	Bamaga – funds may be withdrawn on a pro rata basis should the service not be fully operational throughout the year. This includes any revenue received in advance funding deferred to 2019/20.

Service/Program/Project	Funding	Activity	Timeframe	Conditions
Care in the Right Setting (CaRS):<br>• Better Health North Queensland: Regional Healthcare in the Home<br>• Pop-Up Palliative Care	 *$431,000*<br>$833,975	 *0*<br>0	 *2019/20*<br>2020/21	Services will be provided consistent with the CaRS application(s).<br>If Service commencement does not align with the agreed implementation timeframes funding may be withdrawn on a pro-rata basis.<br>If the agreed Service levels are not provided, funding may be withdrawn.<br>Activity levels will be monitored regularly and cooperation with external evaluators is required.<br>Where the Service includes Service provision to another (receiving) HHS:<br>• If staffing is not available within the HHS to meet the agreed Service levels, the HHS will make alternate arrangements to ensure that the agreed Service levels are provided; and<br>• If the agreed Service levels are not provided, funding may be withdrawn and provided to the receiving HHS.
COVID-19 First Nations Response	$3,031,375	0	2020/21	The HHS will implement and deliver the required actions under the HHS First Nations COVID-19 response including the delivery of initiatives and outcomes outlined in memorandum C-ECTF-20-9652.<br>Funding is one-off in nature for discrete and time-limited activities that are directly attributable to managing the impacts of COVID-19 for First Nations peoples and must be in-scope under the existing financial guidelines for COVID-19 expenditure.<br>HHSs are to retain appropriate supporting documentation to substantiate all expenditure under the National Partnership Agreement.

Service/Program/Project	Funding	Activity	Timeframe	Conditions
Enterprise Bargaining (EB)	*$3,827,164*<br>$1,400,529<br>$3,827 (comprises both recurrent and non-recurrent funding)	*0*<br>0<br>0	*2019/20*<br>2020/21<br>2021/22	Funding has been allocated in full for the following EB agreements:<br>• *Nurses and Midwives (Queensland Health and Department of Education and Training) Certified Agreement (EB10) 2018* (Base wages and certain entitlements)*;* and<br>• *Medical Officers (Queensland Health) Certified Agreement (MOCA5) 2018.*<br>Legislative amendments have been introduced under the *Industrial Relations Act 2016* to give effect to a 2.5% increases under the following agreements as new agreements are yet to be certified:<br>• *Queensland Public Health Sector Certified Agreement (No.9) 2016;*<br>• *Queensland Health, Building, Engineering & Maintenance Services Certified Agreement (No.6) 2016;* and<br>• *Health Practitioners' and Dental Officers (Queensland Health) Certified agreement (No. 2) 2016.*<br>Funding which has been allocated recurrently in previous years has been recalled for the following streams as wage increase are not yet approved:<br>• HES-DSO;<br>• SES-SO; and<br>• VMO.<br>Subject to the terms and conditions of the agreements once executed a funding adjustment may be required. Full details can be found on the Budget and Analysis SharePoint platform.
• Nurses and Midwives EB10 Innovation Fund – Clinical Nurse Consultant to Nurse Practitioner Clinical Excellence Project	*$554,542*<br>$211,290	*0*<br>0	*2019/20*<br>2020/21	Funding has been provided under the *Nurses and Midwives (Queensland Health and Department of Education) Certified Agreement (EB10) 2018,* clause 44.6, Innovation Fund.<br>The HHS will deliver the project, evaluation and reporting as outlined in memo C-ECTF-19/8771.<br>Funding may be withdrawn if project requirements are not met.

Service/Program/Project	Funding	Activity	Timeframe	Conditions
Community Mental Health Growth Allocation	$350,550 (recurrent)	*0*	*2018/19*	Provision of funding to employ 2 additional Full Time Equivalents (FTEs) in support of the HHS's initiatives to enhance the Torres and Cape Child and Youth Mental Health Service.<br>Recruitment of FTEs to be monitored by the Mental Health Alcohol and Other Drugs Branch on a regular basis using the Mental Health Establishment Collection, with adjustments to be made in-year if FTEs not all recruited permanently.
Connecting Care to Recovery 2016-2021: A plan for Queensland's Mental Health<br>• Ed-LinQ Program	$195,800 (recurrent)	*0*	*2019/20*	Implement a new Ed-LinQ program in Torres and Cape HHS.
Nurse Navigators (includes Rheumatic Heart Disease Nurse Navigator)	*$2,265,301*<br>$2,474,785<br>$2,474,785<br>$2,474,785 (recurrent)	*0*<br>0<br>0<br>0	*2019/20*<br>2020/21<br>2021/22<br>2022/23	The total Nurse Navigator Program allocation (2015/16 – 2019/20) is 9 NG7 and 3 NG8. All Nurse Navigator Program Full Time Equivalent (FTE) is required to be appointed to a position ID that has 'Nurse Navigator' within the position title.<br>The HHS is ineligible to appoint Nurse Navigator Program FTE to any pre-existing permanent positions which have been renamed to include 'Nurse Navigator' in the position title.<br>The HHS is required to report monthly on:<br>• Employed Nurse Navigator FTE;<br>• Number of Nurse Navigator plans in place; and<br>• Number of patients seen by Nurse Navigators.
Another 100 Midwives (Nursing)	*$412,026*<br>$421,596<br>$201,354	*0*<br>0<br>0	*2019/20*<br>2020/21<br>2021/22	The HHS will deliver the initiatives and outcomes outlined in the performance requirements as per memo C-ECTF-18/8074. Funding may be withdrawn if requirements are not met.
Nurse Graduates Reprovision	*$419,987*<br>$419,987	*0*<br>0	*2019/20*<br>2020/21	Non-recurrent reprovision of funding to provide continued support for the graduate nurse/midwife program.

Service/Program/Project	Funding	Activity	Timeframe	Conditions
Strength with Immersion Model (SwIM)<br>• Program Management and Governance Hub	*$680,000*	*0*	*2019/20*	The HHS will deliver the initiatives and outcomes outlined in the performance requirements as per memo C-ECTF-19/10267.
Oral Health Services	$4,148,705 (recurrent)	44,485 WOOS	2020/21	Delivery consistent with the Oral Health Policy Framework.<br>Funding may be adjusted where the total oral health activity delivered varies from the purchased levels.<br>Oral health activity (WOOS) for the 0-15 year age group shall not be less than that achieved in 2017/18.<br>Oral health activity (WOOS) includes activity claimed under the Child Dental Benefits Schedule but excludes dental treatment delivered under general anaesthetic in public hospitals.
• National Partnership Agreement (NPA) on Adult Public Dental Services<br>• National Partnership Agreement (NPA) on Adult Public Dental Services – First Nations	$70,000<br>$91,936	660 WOOS<br>867 WOOS	2020/21<br>2020/21	Queensland is required to meet two performance targets during 2020/21, which are the 30 September 2020 target (for 1 April to 30 September 2020) and the 31 March 2021 target (for 1 October 2020 to 31 March 2021). HHSs must collectively meet these targets.<br>Funding may be adjusted where the total oral health activity delivered varies from the purchased levels.
High risk foot patients seen/managed within 48 hours of referral to ambulatory services	$81,721 (recurrent)	*17 WAUs (Q21)*	*2018/19*	The HHS will provide services as specified in the 2019/20 Ambulatory High Risk Foot Services specification sheet published on QHEPS.
Specialist Outpatient Strategy<br>• Telehealth	*$65,000*<br>$65,000 (recurrent)	*0*<br>13 WAUs (Q22)	*2019/20*<br>2020/21	Funding may be withdrawn if requirements are not met.<br>Funding is provided for Telehealth (antenatal telehealth services). The HHS will deliver the initiatives and outcomes outlined in memo C-ECTF-19/6694
Rapid Results Program<br>*Delivering what matters: Advancing Kidney Care 2026 Collaborative*	$1,088,902 (recurrent)	*62 WAUs (Q22 part WAU backed)*<br>96 WAUs (Q22 part WAU backed)	*2019/20*<br>2020/21	Funding is provided for implementation of a chronic kidney disease model of care under the *Advancing Kidney Care 2026 Collaborative.*<br>The HHS will ensure that the reporting requirements established for this initiative are met, including the provision of quarterly progress against agreed implementation milestones and outcome measures.

Service/Program/Project	Funding	Activity	Timeframe	Conditions
Rapid Results Program<br>*Delivering what matters: Networked Cardiac Services*	$278,694 (recurrent)	*27 WAUs (Q22)*	*2019/20*	Funding is provided for the provision of Networked Cardiac Services.<br>Services will be provided consistent with the agreed business case.<br>If service commencement does not align with the agreed implementation timeframes funding may be withdrawn on a pro-rata basis.<br>If the agreed service levels are not provided, funding may be withdrawn.<br>The HHS will utilise the Queensland Cardiac Outcomes Registry Outreach data module for all data capture and reporting.<br>Activity levels will be monitored quarterly.
Planned Care Volume Targets – Elective Surgery	$1,325,052 (Funding in existing service agreement)	304 elective surgery separations aligned with the elective surgery data collection, as reported on SPR and any outsourced elective surgery activity.<br>273 WAUs (Q22).	2020/21	Funding has been quarantined to support the ongoing delivery of planned care volumes during 2020/21. Quarantined funds have been determined based on agreed average Q22 WAUs for 304 Elective Surgery Separations (volume target).<br>Volume targets to be reviewed and adjusted through the amendment window process to include any additional planned care investments.<br>Performance will be monitored quarterly with reconciliation of the agreed planned care activity targets (number of day case and overnight treated patients).<br>Should the agreed volume targets not be met, funding will be withdrawn at the calculated HHS average Q22 WAUs **per separation not delivered**:<br>Example:<br>1 Case = 0.90 Q22 WAUs or<br>$4,359 (QEP)<br>There will not be claw back of under delivery against Q22 WAUs target if the volume of elective day case and overnight separations has been delivered.

Service/Program/Project	Funding	Activity	Timeframe	Conditions
Planned Care Volume Targets – Gastrointestinal Endoscopy (GIE)	$479,549 (Funding in existing service agreement value)	174 Gastrointestinal Endoscopies aligned with the Gastrointestinal Endoscopy data collection, as reported on SPR and any outsourced GIE activity. 99 WAUs (Q22).	2020/21	Funding has been quarantined to support the ongoing delivery of planned care volumes during 2020/21. Quarantined funds have been determined based on agreed average Q22 WAUs for 174 Gastrointestinal Endoscopy Separations (volume target).<br>Volume targets to be reviewed and adjusted through the amendment window process to include any additional planned care investments.<br>Performance will be monitored quarterly with reconciliation of the agreed planned care activity targets (number treated GIE patients).<br>Should the agreed volume targets not be met, funding will be withdrawn at the calculated HHS average Q22 WAUs per separation not delivered:<br>Example:<br>1 Case = 0.57 Q22 WAUs or<br>$2,756 (QEP)<br>There will not be claw back of under delivery against Q22 WAUs target if the volume of GIE day case and overnight separations has been delivered.

3.2 **National Partnership on COVID-19 Response**

(a) The Department will provide additional funding to the HHS under the State Public Health Payment component of the *National Partnership on COVID-19 Response* where actual additional cost is demonstrated.

(b) Funding will be provided where the HHS has:

(i) undertaken activity that is in-scope for the State Public Health Payment during the reporting period; and

(ii) met the reporting requirements in relation to COVID-19 expenditure, as prescribed under the *National Partnership on COVID-19 Response* and by the Department.

(c) The scope of the State Public Health Payment is defined as:

(i) additional costs that are attributable to the treatment of patients with diagnosed or suspected COVID-19; or

(ii) additional costs of activities directed at preventing the spread of COVID-19.

(d) Additional costs that are reimbursed through the State Public Health Payment will be excluded from the calculation of activity eligible for funding under the terms of the National Health Reform Agreement.

(e) There is no guarantee that the HHS will have access to additional funding under the State Public Health Payment.

(f) All funding that is provided through the State Public Health Payment will be subject to a reconciliation to be undertaken by the Administrator of the National Health Funding Pool. The HHS will be required to provide evidence to support claims for additional costs. If the HHS is unable to provide the required evidence on request, funding received may be recalled subject to reconciliation.

(g) Funding adjustments will be actioned through the process set out in clause 3.4 of Schedule 5 of this Service Agreement.

3.3 **Financial adjustments – other**

(a) The purchasing approach includes a range of funding adjustments which aim to incentivise high value care, that is care which delivers the best outcomes at an efficient cost, and dis-incentivise Low Benefit Care. This includes incentive payments for HHS who achieve quality targets in specific areas of priority. The purchasing incentives that apply to this Service Agreement are detailed in Table 2.

(b) The Department must reconcile the applicable purchasing incentives in Table 2 in line with the timeframes specified in the purchasing specification sheet included within the supporting document ‘Purchasing Policy and Funding Guidelines 2020/21’. The Department must promptly provide a copy of the reconciliation statement to the HHS-SA Contact Person.

(c) Funding adjustments must be based on the requirements contained in the relevant specification sheet for that purchasing incentive.

(d) If the Parties are unable to reach agreement in relation to any funding adjustments that are identified, the provisions of clause 14 in the standard terms of this Service Agreement will apply to resolve the dispute.

(e) When the Parties have agreed on a funding adjustment, the Chief Executive (or delegate) will issue an Adjustment Notice to the HHS-SA Contact Person confirming any funding adjustment that has been made in accordance with this clause 3.3 of Schedule 2. The Adjustment Notice will be issued through the process set out in clause 3.4(c) of Schedule 5.

(f) Following receipt of an Adjustment Notice under clause 3.4(c) of Schedule 5, the Parties will comply with the Adjustment Notice and immediately take the steps necessary to give effect to the requirements of that Adjustment Notice.

(g) The Parties acknowledge that the funding adjustments may vary the Service Agreement Value recorded in Schedule 2. Where the Service Agreement Value recorded in Schedule 2 is varied, the variation will be recorded in a Deed of Amendment issued following the next available Amendment Window.

Table 2 Purchasing Incentives 2020/21 (Summary)

Incentive	Description	Scope	Status for 2020/21	Funding Adjustment
Quality Improvement Payment (QIP) – Antenatal Visits for First Nations Women	Incentive payments for achieving targets for:<br>• First Nations women attending an antenatal session during their first trimester, and attending at least 5 antenatal visits; and<br>• First Nations women stopping smoking	All HHSs (excluding Children's Health Queensland)	Continues as per 2019/20 with new targets	50% advance payments made to HHSs with balance paid retrospectively based on performance.
Quality Improvement Payment (QIP) - Smoking Cessation (Community Mental Health)	Incentive payments for achieving targets for community mental health patients clinically supported onto the Smoking Cessation Clinical Pathway	All HHSs (excluding Children's Health Queensland and Mater Public Hospitals)	Continues as per 2019/20 with new targets	Paid retrospectively
High Cost Home Support Program	Payment for high cost 24 hour home ventilated patients meeting the eligibility criteria, where funding is not available through existing sources	All HHSs	Continues as per 2019/20	Paid retrospectively based on forecast costs
Telehealth	Incentive payments for additional outpatient activity volume, provision of telehealth consultancy for Inpatients, Emergency Department and Outpatients episodes and Store and Forward assessments	Inpatients, Emergency Department, Outpatients, and Store and Forward - all HHSs	Continues as per 2019/20 with Outpatients scope expanded to include rural and remote facilities across all HHSs	Paid retrospectively
Sentinel Events	Zero payment for national sentinel events	All ABF public hospitals	Continues as per 2019/20	Retrospective adjustment

3.4 Public and private activity/Own Source Revenue

(a) Own Source Revenue comprises Grants and Contributions, User Charges and Other Revenues.

(b) Where an HHS is above its Own Source Revenue target in respect of private patients, it will be able to retain the additional Own Source Revenue with no compensating adjustments to funding from other sources.

(c) Conversely where an HHS is below its Own Source Revenue target in respect of private patients, it will experience a reduction in revenue with no compensating adjustments to funding from other sources.

(d) The HHS will make all efforts to identify and maximise revenue from appropriate third parties to ensure optimisation of available State and Commonwealth funding across the Queensland Public Sector Health System.

(e) The Own Source Revenue identified in Table 3 is an estimate generated by the HHS which allows all third-party funding sources associated with service delivery

to be identified. The HHS will ensure that this estimate is substantiated and accurate to ensure no significant variances to actual revenue generated.

(f) The HHS will routinely revise and update the estimate to ensure alignment between the Service Agreement and Queensland Treasury's reporting system (TRIDATA).

(g) Budget adjustments for changes in Own Source Revenue from private patients will be actioned through the process set out in Schedule 5 of this Service Agreement.

4. Funding sources

4.1 The four main funding sources contributing to the HHS Service Agreement value are:

(a) Commonwealth funding;

(b) State funding;

(c) Grants and Contributions; and

(d) Own Source Revenue.

4.2 Table 3 provides a summary of the funding sources for the HHS and mirrors the total value of the Service Agreement included in Table 4.

Table 3 Hospital and Health Service funding sources 2020/21

Funding Source	Value ($)
**NHRA Funding**	
Activity Based Funding	-
Clinical Education and Training[3]	-
Own Source Revenue contribution in ABF funded services	-
**Pool Account – ABF Funding (State and Commonwealth)[4]**	**-**
Block Funding	109,395,924
Clinical Education and Training[3]	-
**State Managed Fund – Block Funding (State and Commonwealth)[5]**	**109,395,924**
**Locally Receipted Funds (Including Grants)**	**18,491,654**
**Locally Receipted Own Source Revenue (ABF)**	**-**
**Locally Receipted Own Source Revenue (Other activities)**	**5,358,730**
**Department of Health Funding[6]**	**102,464,743**
**Total NHRA Funding**	**235,711,051**
**NPA Covid-19 Response**	
Activity Based Funding	-
**Hospital Services Payment – ABF Funding (State and Commonwealth)[7]**	**-**
Block Funding	-
Clinical Education and Training[3]	-
**Hospital Services Payment – Block Funding (State and Commonwealth)[5]**	**-**
**Public Health Funding (State and Commonwealth) [8]**	**3,031,376**
**Total NPA – COVID-19 Funding**	**3,031,376**
**TOTAL**	**238,742,427**

[3] Clinical Education and Training (CET) is classified as Teaching, Training and Research Funding under the National Model and funded as a Block Funded Service. Under the State Model, CET is included as 'Other ABF' and forms part of the ABF total. To comply with the requirements of the National Health Reform Agreement, funding must be paid as it is received, therefore from a Funding Source perspective, CET has been reclassified to Block Funding.

[4] Pool Account - ABF Funding (State and Commonwealth) includes: Inpatient; Critical Care; Emergency Department; Sub and Non Acute; Mental Health; and Outpatient activities each allocated a proportion of Other ABF Adjustments.

[5] State Managed Fund - Block Funding (State and Commonwealth) includes: block funded hospitals; standalone specialist mental health hospitals; community mental health; and teaching, training and research.

[6] Department of Health Funding represents funding by the Department for items not covered by the National Health Reform Agreement including such items as: Prevention, Promotion and Protection; Depreciation, and other Health Services.

7 Hospital Services Payment - Funding provided under the COVID-19 National Partnership Agreement for activity that is attributable to the diagnosis and treatment of Medicare eligible patients with COVID-19 or suspected of having COVID-19; elective surgeries that have been rescheduled to free up public hospital capacity to respond to the COVID-19 outbreak; and may include activities related to the care of public patients being treated in private hospitals.

[8] Public Health Payment - Funding provided under the COVID-19 National Partnership Agreement for the State public health system's activity attributable to the treatment of patients with diagnosed or suspected COVID-19 or for activities directed at preventing the spread of COVID-19.

5. Funds disbursement

5.1 The Chief Executive will direct the disbursement of both State and Commonwealth funding from the State's National Health Funding Pool Sub Account and the State Managed Fund to the HHS. The Service Agreement and State level block payments to State managed funds from Commonwealth payments into the national funding pool are stated in Table 8.

5.2 However, the State (represented by the Chief Executive) will not:

(a) redirect Commonwealth payments between HHSs;

(b) redirect Commonwealth payments between funding streams (e.g. from ABF to Block Funding); and/or

(c) adjust the payment calculations underpinning the Commonwealth's funding.

5.3 Payment of Activity Based Funding and Block Funding to the HHS will be on a fortnightly basis.

5.4 Further information on the disbursement of funds is available in the supporting document to this Service Agreement 'Purchasing Policy and Funding Guidelines 2020/21'.

Table 4 HHS Finance and Activity Schedule 2019/20 – 2021/22 – Summary by Purchasing Hierarchy

	ABF/NABF	ABF_SPLIT	Service Stream	2019/20 QWAU (Q22)	2019/20 Funding ($) (Price: $4847)	2020/21 QWAU (Q22)	2020/21 Funding) (Price: $4847)	2021/22 QWAU (QTBC)	2021/22 Funding (Price: $TBC)
Allocations excluding COVID-19	ABF	ABF	Inpatients	0	$0	0	$0		
			Outpatients	0	$0	0	$0		
			Procedures & Interventions	0	$0	0	$0		
			Emergency Department	0	$0	0	$0		
			Sub & Non-Acute	0	$0	0	$0		
			Mental Health	0	$0	0	$0		
			Prevention & Primary Care	0	$0	0	$0		
			Other ABF $	0	$0	0	$0		
		**ABF Total**		**0**	**$0**	**0**	**$0**		
		ABF Other	CET Funding	0	$0	0	$0		
			Specified Grants	0	$0	0	$0		
			PPP	0	$0	0	$0		
			EB Quarantined	0	$0	0	$0		
		**ABF Other Total**		**0**	**$0**	**0**	**$0**		
	Other Funding	Other Funding	Block Funded Services	11,778	$107,716,954	11,763	$108,345,284		
			Population Based Community Services	0	$73,206,974	0	$72,224,341		
			Other Specific Funding	0	$55,079,740	0	$54,004,151		
			PY Services moved to ABF	0	$0	0	$0		
			Prevention Services – Public Health	0	$985,560	0	$1,137,275		
		**Other Funding Total**		**11,778**	**$236,989,228**	**11,763**	**$235,711,052**		
	**Allocations excluding COVID-19 TOTAL**			**11,778**	**$236,989,228**	**11,763**	**$235,711,052**		

	ABF/NABF	ABF_SPLIT	Service Stream	2019/20 QWAU (Q22)	2019/20 Funding ($) (Price: $4847)	2020/21 QWAU (Q22)	2020/21 Funding) (Price: $4847)	2021/22 QWAU (QTBC)	2021/22 Funding (Price: $TBC)
**COVID-19 related allocations**	**ABF**	ABF	Inpatients	0	$0	0	$0		
			Outpatients	0	$0	0	$0		
			Procedures & Interventions	0	$0	0	$0		
			Emergency Department	0	$0	0	$0		
			Sub & Non-Acute	0	$0	0	$0		
			Mental Health	0	$0	0	$0		
			Prevention & Primary Care	0	$0	0	$0		
			Other ABF $	0	$0	0	$0		
		**ABF Total**		**0**	**$0**	**0**	**$0**		
		ABF Other	CET Funding	0	$0	0	$0		
			Specified Grants	0	$0	0	$0		
			PPP	0	$0	0	$0		
			EB Quarantined	0	$0	0	$0		
		**ABF Other Total**		**0**	**$0**	**0**	**$0**		
	**Other Funding**	Other Funding	Block Funded Services	0	$0	0	$0		
			Population Based Community Services	0	$0	0	$0		
			Other Specific Funding	0	$2,532,176	0	$3,031,375		
			PY Services moved to ABF	0	$0	0	$0		
			Prevention Services – Public Health	0	$0	0	$0		
		**Other Funding Total**		**0**	**$2,532,176**	**0**	**$3,031,375**		
	**COVID-19 Allocations TOTAL**			**0**	**$2,532,176**	**0**	**$3,031,375**		
**Grand Total**				**11,778**	**$239,521,404**	**11,763**	**$238,742,427**		

Table 5 Minor Capital and Equity

	2019/20 $	2020/21 $	2021/22 $
**Minor Capital & Equity**			
**Cash**			
SA 16-17.332 - Minor Capital funding Allocation 2016-17	$1,434,000	$1,434,000	
TAC-Apr17-06 BMRP Output to Equity Swap	$0	$0	
TAC-Apr17-08 BMRP Equity Swap	$0	$0	
TAC-AW2-OCT19-26 TAC - Leases operating to equity swap	$2,830,520	$0	
TAC-AW3-FEB20-06 Lease funding swap per changes to AASB16 - equity component	$157,287	$0	
TAC-AW3-FEB20-08 2019-20 Capital Swaps - equity component	$35,700	$0	
TAC-BB2021-23 Lease funding swap per changes to AASB16 - equity component	$0	$2,111,542	
**Non-Cash**			
-	-	-	
**Grand Total**	**$4,457,507**	**$3,545,542**	

Table 6 HHS Finance and Activity Schedule 2019/20 – 2021/22 Other Funding Detail

Agreement		Service Stream	Category	2019/20 $	2020/21 $	2021/22 $
Allocations excluding COVID-19	Other Funding	Block Funded Services	Block Funded Services	$107,716,954	$108,345,284	
		**Block Funded Services Total**		**$107,716,954**	**$108,345,284**	
		Population Based Community Services	Alcohol, Tobacco & Other Drugs	$3,642,651	$3,674,102	
			Community Care Programs	$226,976	$230,331	
			Community Mental Health	$5,320,894	$5,368,443	
			Community Mental Health – Child & Youth	$587,067	$592,196	
			Other Community Services	$21,094,989	$20,018,729	
			Other Funding Subsidy/(Contribution)	-$4,851,488	-$4,851,488	
			Primary Health Care	$47,185,885	$47,192,028	
		**Population Based Community Services Total**		**$73,206,974**	**$72,224,341**	
		Other Specific Funding	Aged Care Assessment Program	$0	$0	
			Commercial Activities	$0	$0	
			Consumer Information Services	$0	$0	
			Depreciation	$18,343,000	$18,649,000	
			Disability Residential Care Services	$30,042	$0	
			Environmental Health	$177,404	$178,954	
			Home & Community Care (HACC) Program	$784,463	$784,463	
			Home & Community Medical Aids & Appliances	$44,696	$44,696	
			Home Care Packages	$0	$0	
			Interstate Patients	$329,859	$329,859	
			Multi-Purpose Health Services	$2,210,958	$2,210,958	
			Prisoner Health Services	$0	$0	
			Oral Health	$0	$0	
			Patient Transport	$12,962,085	$12,962,085	
			Research	$0	$0	
			Residential Aged Care	$0	$0	
			Specific Allocations	$15,866,666	$14,513,570	
			State-Wide Functions	$4,330,567	$4,330,567	
			Transition Care	$0	$0	
		**Other Specific Funding Total**		**$55,079,740**	**$54,004,152**	
		Prevention Services – Public Health	Environmental Health (PH)	$93,102	-$263	
			Other Community Services (PH)	$892,458	$1,137,538	

Agreement		Service Stream	Category	2019/20 $	2020/21 $	2021/22 $
		**Prevention Services – Public Health Total**		**$985,560**	**$1,137,275**	
	**Allocations excluding COVID-19 Other Funding Total**			**$236,989,228**	**$235,711,052**	
**COVID-19 related allocations**	**Other Funding**	Block Funded Services	Block Funded Services	$0	$0	
		**Block Funded Services Total**		**$0**	**$0**	
		Population Based Community Services	Alcohol, Tobacco & Other Drugs	$0	$0	
			Community Care Programs	$0	$0	
			Community Mental Health	$0	$0	
			Community Mental Health – Child & Youth	$0	$0	
			Other Community Services	$0	$0	
			Other Funding Subsidy/(Contribution)	$0	$0	
			Primary Health Care	$0	$0	
		**Population Based Community Services Total**		**$0**	**$0**	
		Other Specific Funding	Aged Care Assessment Program	$0	$0	
			Commercial Activities	$0	$0	
			Consumer Information Services	$0	$0	
			Depreciation	$0	$0	
			Disability Residential Care Services	$0	$0	
			Environmental Health	$0	$0	
			Home & Community Care (HACC) Program	$0	$0	
			Home & Community Medical Aids & Appliances	$0	$0	
			Home Care Packages	$0	$0	
			Interstate Patients	$0	$0	
			Multi-Purpose Health Services	$0	$0	
			Prisoner Health Services	$0	$0	
			Oral Health	$0	$0	
			Patient Transport	$0	$0	
			Research	$0	$0	
			Residential Aged Care	$0	$0	
			Specific Allocations	$2,532,176	$3,031,375	
			State-Wide Functions	$0	$0	
			Transition Care	$0	$0	
		**Other Specific Funding Total**		**$2,532,176**	**$3,031,375**	
			Environmental Health (PH)	$0	$0	

Agreement		Service Stream	Category	2019/20 $	2020/21 $	2021/22 $
		Prevention Services – Public Health	Other Community Services (PH)	$0	$0	
		**Prevention Services – Public Health Total**		**$0**	**$0**	
	**COVID-19 Allocations Other Funding Total**			**$2,532,176**	**$3,031,375**	
**Grand Total**				**$239,521,404**	**$238,742,427**	

Table 7 Specified Grants

This table does not apply to this HHS.

Table 8 Hospital and Health Service Service Agreement and State Level Block Payments to state managed funds from Commonwealth payments into national funding pool

**State:**	QLD	**Service agreement for financial year:**	2020/21
**HHS**	Torres and Cape	**Version for financial year:**	
**HHS ID**		**Version effective for payments from:**	
		**Version status:**	07.07.2020

HHS ABF payment requirements:

Expected National Weighted Activity Unit (NWAU)		National efficient price (NEP) (as set by IHPA)
**ABF Service group**	**Projected NWAU – N2021 (Draft)**	
Admitted acute public services	0	$5,320
Admitted acute private services	0	$5,320
Emergency department services	0	$5,320
Non-admitted services	0	$5,320
Mental health services	0	$5,320
Sub-acute services	0	$5,320
**LHN ABF Total – excluding COVID-19**	**0**	**$5,320**
**LHN ABF Total – COVID-19 NPA**	**0**	**$5,320**

Note:

NWAU estimates do not take account of cross-border activity

Reporting requirements by HHS - total block funding paid (including Commonwealth) per HHS, as set out in Service Agreement:

**Amount (Commonwealth and State) for each amount of block funding from state managed fund to LHN:**	
**Block funding component**	**Estimated Commonwealth and state block funding contribution (ex GST)**
Block funded hospitals	$100,545,155
Community mental health services	$8,850,770
Teaching, Training and Research	$0
Home ventilation	$0
Other block funded services	$0
**Total block funding for LHN – excluding COVID-19**	**$109,395,924**
**Total funding for LHN under COVID-19 NPA State Public Health Payment**	**$3,031,375**

6. Purchased services

6.1 State-funded Outreach Services

(a) The HHS forms part of a referral network with other HHSs. Where state-funded Outreach Services are currently provided the HHS will deliver these Health Services in line with the following principles:

(i) historical agreements for the provision of Outreach Services will continue as agreed between HHSs;

(ii) funding will remain part of the providing HHS's funding base;

(iii) activity should be recorded at the HHS where the Health Service is being provided; and

(iv) the Department will purchase outreach activity based on the utilisation of the Activity Based Funding (ABF) price when Outreach Services are delivered in an ABF facility.

(b) Where new or expanded state-funded Outreach Services are developed the following principles will apply:

(i) the Department will purchase outreach activity based on the utilisation of the ABF price when Outreach Services are delivered in an ABF facility;

(ii) agreements between HHSs to purchase Outreach Services will be based on a cost recovery model, which will ensure providing sites are not financially disadvantaged and annual increases will be consistent with the ABF model;

(iii) any proposed expansion or commencement of Outreach Services will be negotiated between HHSs;

(iv) the HHS is able to purchase the Outreach Service from the most appropriate provider including private providers or other HHSs. However, when a change to existing Health Services is proposed, a transition period of at least 12 months will apply during which time the HHS will be required to continue to purchase Outreach Services from the HHS currently providing the Health Service;

(v) any changes to existing levels of Outreach Services need to be agreed to by both HHSs and any proposed realignment of funding should be communicated to the Department to ensure that any necessary funding changes are actioned as part of the Service Agreement amendment process and/or the annual negotiation of the Service Agreement Value; and

(vi) the activity should be recorded at the HHS where the Health Service is being provided.

(c) In the event of a disagreement regarding the continued provision of state-funded Outreach Services:

(i) any proposed cessation of Outreach Services will be negotiated between HHSs to mitigate any potential disadvantage or risks to either HHS; and

(ii) redistribution of funding will be agreed between the HHSs and communicated to the Department to action through the Service Agreement amendment processes outlined in Schedule 5 of this Service Agreement.

6.2 Telehealth services

(a) The HHS will support implementation of the Department Telehealth program, including the Telehealth Emergency Support Service. The HHS will collaborate with the Department, other HHSs, relevant non-government organisations and Primary Care stakeholders to contribute to an expanded network of Telehealth services to better enable a program of scheduled and unscheduled care.

(b) The HHS will ensure dedicated Telehealth Coordinators progress the Telehealth agenda locally, driving stakeholder engagement, adoption, planning and implementation activities that will support and grow Telehealth enabled services through substitution of existing face to face services and identification of new Telehealth enabled models of care.

(c) The HHS will ensure the Medical Telehealth Lead will collaborate with the network of HHS based Telehealth Coordinators and the Telehealth Support Unit to assist in driving promotion and adoption of Telehealth across the State through intra and cross-HHS clinician led engagement and change management initiatives as well as informing the development and implementation of clinical protocols and new Telehealth enabled models of care.

6.3 Newborn hearing screening

(a) In line with the National Framework for Neonatal Hearing Screening the HHS will:

(i) provide newborn hearing screening in all birthing hospitals and screening facilities; and

(ii) provide where applicable, co-ordination, diagnostic audiology, family support, and childhood hearing clinic services which meet the existing screening, audiology and medical protocols available from the Healthy Hearing website.

6.4 Statewide Services

This clause does not apply to this HHS.

6.5 Statewide and highly specialised clinical services

The HHS will:

(a) participate in and contribute to the staged review of the purchasing model for identified Statewide and highly specialised clinical services; and

(b) collaborate with the Department and other HHSs in the development of Statewide Services Descriptions through the implementation of the Statewide Services Governance and Risk Management Framework. The Statewide Services Governance and Risk Management Framework guides the Department and HHSs in the strategic management, oversight and delivery of Statewide Services in order to optimise clinical safety and quality and ensure sustainability of services across Queensland.

6.6 **Regional Services**

This clause does not apply to this HHS.

6.7 **Rural and remote clinical support**

(a) Torres and Cape HHS will host the functions provided by the Rural and Remote Clinical Support Unit as detailed below on behalf of the following identified HHSs:

(i) Central West HHS;

(ii) North West HHS;

(iii) South West HHS; and

(iv) Torres and Cape HHS.

(b) The functions to be provided by the Rural and Remote Clinical Support Unit will include:

(i) provision of high quality, consistent credentialing, credentialing support and credentialing database services which comply with any relevant health service directive(s) and Department statewide credentialing guidelines as required by the Client Engagement Committee;

(ii) provision of a medical support advisory service;

(iii) provision of a medical employment advisory service;

(iv) production, maintenance, review and delivery of the Blended and online orientation and education resources;

(v) production, maintenance, review and publishing of the Chronic Conditions Manual (CCM);

(vi) production, maintenance, review and publishing of the Primary Clinical Care Manual (PCCM) in accordance with the requirements of the prevailing drugs and poisons legislation within the State of Queensland; and

(vii) delivery of special projects and support as required by the Client Engagement Committee.

(c) Services will be delivered according to the following principles:

(i) functions may be accessed by the identified HHSs

(ii) a Client Engagement Committee with representatives from the identified HHSs will:

(A) endorse the operational plans and Performance Indicators for the unit;

(B) on (at least) an annual basis, review the hosted functions to determine the level of utilisation, satisfaction and the relevance of hosted functions; and

(C) endorse changes to functions and reinvestment of existing resources to develop services relevant to HHS requirements.

6.8 **Prevention Services, Primary Care and Community Health Services**

(a) The following funding arrangements will apply to the Prevention, Primary Care and Community Health Services delivered by the HHS:

(i) Department funding for Community Health Services. A pool of funding for these services is allocated to each HHS for a range of Community Health Services and must be used to meet local Primary Care and community healthcare and prevention needs including through delivery of the services identified in Table 6 and HHSs have the discretion to allocate funding across Primary Care and Community Health Services and Prevention Services according to local priorities.

(ii) Department specified funding models for consumer information services, disability, residential care, environmental health, prisoner health services, home and community medical aids, Primary Care, community mental health services, and alcohol and other drugs services. The funding specified for these programs is listed in Table 6 and Department Community Health Service grants.

(iii) Funding from other state government departments and the Commonwealth for specific programs (third party funded services).

(b) **Prevention Services**

The HHS will provide Prevention Services in line with public health related legislation and the service delivery and reporting requirements outlined in the *Public Health Practice Manual*, including:

(i) **Specialist Public Health Units**

The HHS will:

(A) maintain the sexual health services and will deliver these services at a minimum to those levels delivered in 2012/13, including:

- provision of clinical sexual and reproductive health service for residents on Thursday Island and other inner islands;
- provision of sexual health orientation and workforce development for all generalist clinical staff who work either in the primary health care centre or at Thursday Island hospital; and
- progression of the implementation of school-based sexuality and relationships education in Tagai Collage on Thursday Island and oversee condom distribution and monitoring;

(B) maintain and improve, using a public health approach, the surveillance, prevention and control of notifiable conditions, including the prevention and control of invasive and exotic mosquitos, in accordance with national and/or State guidelines and ensure clinical and provisional notification of specified notifiable conditions are reported in accordance with the *Public*

Health Act and Public Health Regulations;

(C) provide environmental health worker support to First Nations communities; and

(D) provide office accommodation and related support to Cairns and Hinterland Public Health Unit (PHU) staff who are required to visit Cape York. The Cairns and Hinterland PHU will provide specialist communicable disease epidemiology and surveillance, disease prevention and control and environmental health services to Torres and Cape HHS.

(ii) **Preventive health services**

The HHS will:

(A) maintain delivery of risk factor prevention and early intervention programs and services targeting nutrition, physical activity, alcohol consumption, tobacco use, overweight and obesity and falls prevention;

(B) maintain delivery of the school-based youth nursing program throughout Queensland secondary schools; and

(C) promote brief interventions, lifestyle modification programs and other prevention, promotion or early intervention activities.

(iii) **Immunisation services**

The HHS will maintain or improve existing immunisation coverage through continuation of current immunisation services including:

(A) national immunisation program;

(B) opportunistic immunisation in healthcare facilities;

(C) special immunisation programs; and

(D) delivery of the annual school immunisation program in accordance with the Guideline for Immunisation Services (QH-GDL-955:2014).

(iv) **Sexually transmissible infections including HIV and viral hepatitis**

The HHS will:

(A) maintain and improve, using a public health approach, the monitoring, surveillance and evaluation of sexual health services as guided by the *North Queensland Aboriginal and Torres Strait Islander STI Action Plan 2016-2021* and *Schedule A: Addressing Blood Borne Viruses and Sexually Transmissible* Infections in *the Torres Strait, Project Agreement on Healthcare and disease Prevention in the Torres Strait Islands.*

(B) maintain and improve, using a primary healthcare health approach, the prevention, testing, treatment and contact tracing of blood borne viruses and sexually transmissible infections with

a continued focus on relevant identified target populations such as First Nations people and culturally and linguistically diverse populations through Services including, but not limited to:

- public health units;
- Primary Care services;
- sexual health services;
- men's and women's health programs;
- infectious diseases services;
- viral hepatitis services;
- syphilis surveillance services;
- needle and syringe programs; and
- existing clinical outreach and support programs in place between HHSs.

(v) **Tuberculosis services**

(A) The HHS will ensure there is no financial barrier for any person to tuberculosis diagnostic and management services and will ensure that services are available in accordance with the Tuberculosis Control health service directive (qh-hsd-040:2018) and Protocol (qh-hsdptl-040-1:2018).

(B) The Torres and Cape Tuberculosis Control Unit will provide the following services to Torres and Cape HHS:

- patient assessment and management of mycobacterial diseases;
- outpatient services onsite and clinics offsite;
- clinics as required at private health facilities, educational facilities and public areas and workplaces;
- screening services; and
- BCG vaccination services.

(vi) **Public Health Events of State Significance**

The HHS will comply with the *Declaration and Management of a Public Health Event of State Significance* health service directive (qh-hsd-046:2014).

(vii) **Cancer screening services**

The HHS will:

(A) increase cervical screening rates for women in rural and remote areas and refer clients to relevant preventive health programs such as BreastScreen Queensland, QUIT line, Get Healthy and

My Health for Life by maintaining the existing Mobile Women's Health Service;

(B) provide the Department with an annual report detailing the services provided by the Mobile Women's Health Service;

(C) ensure that all cervical screening services provided by the HHS are delivered in accordance with the National Competencies for Cervical Screening Providers and national cervical screening policy documents;

(D) maintain the existing Healthy Women's Initiative in accordance with the Principles of Practice, Standards and Guidelines for Providers of Cervical Screening Services for First Nations women and national cervical screening policy documents;

(E) provide timely, appropriate, high quality and safe follow-up diagnostic services within the HHS for National Cervical Screening Program participants in accordance with the *National Cervical Screening Program Guidelines for the Management of Screen-detected Abnormalities, Screening in Specific Populations and Investigation of Abnormal Vaginal Bleeding* (2017) and national cervical screening policy documents; and

(F) provide primary healthcare medical services with an emphasis on cervical screening and health promotion to women in rural and remote communities of the Torres Strait, including all 15 islands, and Northern Cape and provide the Department with an annual report detailing the services provided.

(G) Provide timely, appropriate, high quality and safe diagnostic assessment services for national bowel cancer screening program participants in accordance with the National Health and Medical Research Council's *Clinical Guidelines for Prevention, Early Detection and Management of Colorectal Cancer* (2017).

6.9 **Oral health services**

The HHS will ensure that:

(a) oral health services are provided to the Eligible Population at no cost to the patient[9] and that the current range of clinical services will continue;

(b) oral health services fulfil the relevant obligations related to Commonwealth Government dental funding program/s;

(c) service delivery is consistent with Queensland Health's oral health policy framework; and

(d) the repair maintenance and relocation service for the mobile dental fleet continues to be provided by the Mobile Dental Clinic Workshop in Metro South HHS.

---

[9] The HHS may provide oral health services on a fee-for-service basis to non-eligible patients in rural and remote areas where private dental services are not available.

6.10 **Prisoner health services**

This clause does not apply to this HHS.

6.11 **Refugee health**

This clause does not apply to this HHS.

6.12 **Adult sexual health clinical forensic examinations**

This clause does not apply to this HHS.

7. Teaching training and research

The HHS will provide the teaching, training and research programs for which funding is provided within Schedule 2 of this Service Agreement and as described below:

7.1 **Clinical education and training**

(a) The HHS will:

(i) continue to support and align with the current Student Placement Deed Framework which governs clinical placements from relevant tertiary education providers in Queensland HHS facilities;

(ii) comply with the obligations and responsibilities of Queensland Health under the Student Placement Deed, as appropriate, as operator of the facility at which the student placement is taking place;

(iii) comply with the terms and conditions of students from Australian education providers participating in the Student Placement Deed Framework;

(iv) only accept clinical placements of students from Australian education providers participating in the Student Placement Deed Framework;

(v) continue to provide training placements consistent with and proportionate to the capacity of the HHS. This includes, but is not limited to, planning and resourcing for clinical placement offers in collaboration with other HHSs and the Department, and the provision of placements for the following professional groups relevant to the HHS:

(A) medical students

(B) nursing

(C) pre-entry clinical placement for allied health students

(D) interns

(E) rural generalist trainees

(F) vocational medical trainees

(G) first year nurses and midwives

(H) oral health students

(I) allied health rural generalist training positions

(J) Aboriginal and Torres Strait Islander Health Workers and Aboriginal and Torres Strait Islander Health Practitioners

(vi) participate in vocational medical rotational training schemes and facilitate the movement of vocational trainees between HHSs;

(vii) report, at the intervals and in the format agreed between the Parties, to the Department on the pre-entry clinical placements provided under the Student Placement Deed Framework;

(viii) comply with the state-wide vocational medical training pathway models including:

(A) The Queensland Basic Physician Training Network;

(B) The Queensland General Medicine Advanced Training Network;

(C) The Queensland Intensive Care Training Pathway;

(D) The Queensland Basic Paediatric Training Network;

(E) The Queensland General Paediatric Advanced Training Network; and

(F) The Queensland Neonatal and Perinatal Medicine Advanced Training Network;

(ix) support the provision of placements by the Queensland Physiotherapy Placement Collaborative for physiotherapy pre-entry students via the Physiotherapy Pre-registration Clinical Placement Agreement; and

(x) provide clinical area placements for dietetics pre-entry students from additional funding provided through relevant agreements with Universities.

(b) In addition, the Health Practitioners and Dental Officers (Queensland Health) Certified Agreement (No 2) 2016 (the HP agreement) requires Hospital and Health Services to:

(i) continue to support development of allied health research capacity through continued implementation and retention of health practitioner research positions provided through the HP agreement; and

(ii) support development of allied health clinical education capacity through continued implementation and retention of clinical educator positions provided through the HP agreement, continuing to provide allied health pre-entry clinical placements and maintaining support for allied health HP 3 to 4 rural development pathway positions.

7.2 **Health and medical research**

The HHS will:

(a) Articulate an investment strategy for research (including research targets and Performance Measures) which integrates with the clinical environment to improve clinical outcomes;

(b) Develop mechanisms for completing administrative governance for the approval of research in line with a national benchmark of 25 days (Standard Operating Procedures for Queensland Health Research Governance Officers 2013);

(c) Develop mechanisms for monitoring site research activity in line with jurisdictional commitments and National Health and Medical Research Council Guidelines (*Framework for* Monitoring *Guidance for the national approach to single ethical review of multi-centre research, January 2012*); and

(d) Develop systems to capture research and development expenditure and revenue data and associated information on research.

Schedule 3 Performance Measures

1. Purpose

This Schedule 3 outlines the Performance Measures that apply to the HHS.

2. Performance Measures

2.1 The Performance and Accountability Framework uses Performance Measures to monitor the extent to which the HHS is delivering the high-level objectives set out in this Service Agreement.

2.2 Each Performance Measure is identified under one of four categories:

(a) Safety and Quality Markers which together provide timely and transparent information on the safety and quality of services provided by the HHS;

(b) Key Performance Indicators (KPIs) which are focused on the delivery of key strategic objectives and statewide targets. KPI performance will inform HHS performance assessments;

(c) Outcome Indicators which provide information on specific activities and interventions where there is a reasonable expectation or evidence that they make a positive contribution towards improving the health status and experience of patients; and

(d) supporting indicators which provide contextual information and enable an improved understanding of performance, facilitate benchmarking of performance across HHSs and provide intelligence on potential future areas of focus. Supporting indicators are available on-line as referenced in Appendix 1 to this Service Agreement.

2.3 The HHS should refer to the relevant attribute sheet for each Performance Measure for full details. These are available on-line as referenced in Appendix 1 to this Service Agreement.

2.4 The Performance Measures identified in Table 9; Table 10 and Table 11 are applicable to the HHS unless otherwise specified within the attribute sheet.

2.5 The HHS will meet the target for each KPI identified in Table 9 as specified in the attribute sheet.

2.6 The Performance Measures identified in italic text are for future development.

2.7 Further information on the performance assessment process is provided in the supporting document to this Service Agreement, Performance and Accountability Framework 2020/21 referenced at Appendix 1 to this Service Agreement.

Table 9 HHS Performance Measures – Key Performance Indicators

**Key Performance Indicators**	
**Safe**	
The health and welfare of service users is paramount	
• Minimise risk	• Avoid harm from care
• Transparency and openness	• Learn from mistakes
**Title**	
Hospital Acquired Complications	
**Timely**	
Care is provided within an appropriate timeframe	
• Treatment within clinically recommended time	
**Title**	
Telehealth utilisation rates:<br>• Number of non-admitted telehealth service events	
Access to oral health services:<br>• % of patients on the general care dental wait list waiting for less than the clinically recommended time	
**Equitable**	
Consumers have access to healthcare that is responsive to need and addresses health inequalities	
• Fair access based on need	• Addresses inequalities
Potentially Preventable Hospitalisations – First Nations People	
Low birthweight:<br>• % of low birthweight babies born to Queensland mothers	
**Efficient**	
Available resources are maximised to deliver sustainable, high quality healthcare	
• Avoid waste	• Minimise financial risk
• Sustainable/productive	• Maximise available resources
**Title**	
Forecast operating position:<br>• Full year<br>• Year to date	
Average sustainable Queensland Health FTE	
Capital expenditure performance	
Increase participation in Practice Incentive Program	
**Patient Centred**	
Providing Healthcare that is respectful of and responsive to individual patient preferences, needs and values	
• Patient involved in care	• Patient feedback
• Respects patient/person values and preferences	• Care close to home
**Title**	
Proportion of mental health service episodes with a documented care plan	
Travel rates for admitted patients – reduction target	

Effective	
Healthcare that delivers the best achievable outcomes through evidence-based practice	
• Evidence based practice	• Care integration
• Treatment directed to those who benefit	• Optimise Health
• Clinical Capability	
**Title**	
Potentially Preventable Hospitalisations – non-diabetes complications:<br>• The number and proportion of hospitalisations of people with non-diabetes complications that could have potentially been prevented through the provision of appropriate non-hospital health services	
% of oral health activity which is preventive	
Diabetes control:<br>• *Type 2 diabetes patients with HbA1c result within a specified level*	
Smoking Status:<br>• *% of patients with a respiratory diagnosis whose smoking status has been recorded*	
**People and Culture**	
**Title**	
Workforce:<br>• *Specific measure/s to be determined*	

Table 10 HHS Performance Measures - Safety and Quality Markers

Safety and Quality Markers	
**Safe**	
The health and welfare of service users is paramount	
• Minimise risk<br>• Transparency and openness	• Avoid harm from care<br>• Learn from mistakes
**Title**	
Sentinel Events:<br>• Number of wholly preventable sentinel events	
Hospital Standardised Mortality Ratio	
Severity Assessment Code (SAC) closure rates:<br>• % of incidents closed within the prescribed timeframe	
Transfer of care for mental health patients:<br>• *Time from arrival in the Emergency Department to admission to an acute mental health bed*	

Table 11 HHS Performance Measures – Outcome Indicators

Outcome Indicators	
**Timely**	
Care is provided within an appropriate timeframe	
• Treatment within clinically recommended time	
**Title**	
Telehealth:<br>• *Expansion to rural and remote communities*	
Access to emergency dental care:<br>• % of emergency courses of care for adult dental patients that commence within the recommended waiting times	
**Equitable**	
Consumers have access to healthcare that is responsive to need and addresses health inequalities	
• Fair access based on need	• Addresses inequalities
**Title**	
First Nations people representation in the workforce:<br>• % of the workforce who identify as being First Nations people	
Completed general courses of oral health care for First Nations people adult patients	
**Patient Centred**	
Providing Healthcare that is respectful of and responsive to individual patient preferences, needs and values	
• Patient involved in care	• Patient feedback
• Respects patient/person values and preferences	• Care close to home
**Title**	
Complaints resolved within 35 calendar days	
Advance care planning:<br>• The proportion of approaches made to people who are identified as being at risk of dying within the next 12 months, or suitable for an advance care planning discussion, and who are offered the opportunity to consider, discuss and decide their preferences for care at the end of life	
Outpatient appointments delivered outside the HHS area	
Integrated care pathway in place for patients with identified co-morbidities	
**Effective**	
Healthcare that delivers the best achievable outcomes through evidence-based practice	
• Evidence based practice	• Care integration
• Treatment directed to those who benefit	• Optimise Health
• Clinical Capability	
**Title**	
Uptake of the smoking cessation clinical pathway for public hospital inpatients and dental clients	
Adolescent vaccinations administered via the statewide School Immunisation Program	

Schedule 4 Data Supply Requirements

1. Purpose

1.1 *The Hospital and Health Boards Act 2011*[10] (s.16(1)(d)) provides that the Service Agreement will state the performance data and other data to be provided by an HHS to the Chief Executive, including how, and how often, the data is to be provided.

1.2 This Schedule 4 specifies the data to be provided by the HHS to the Chief Executive and the requirements for the provision of the data.

2. Principles

2.1 The following principles guide the collection, storage, transfer and disposal of data:

(a) trustworthy – data is accurate, relevant, timely, available and secure;

(b) private – personal information is protected in accordance with the law;

(c) valued – data is a core strategic asset;

(d) managed – collection of data is actively planned, managed and compliant; and

(e) quality – data provided is complete, consistent, undergoes regular validation and is of sufficient quality to enable the purposes outlined in clause 3.2 of this Schedule 4 to be fulfilled.

2.2 The Parties agree to constructively review the data supply requirements as set out in this Schedule 4 on an ongoing basis in order to:

(a) ensure data supply requirements are able to be fulfilled; and

(b) minimise regulatory burden.

3. Roles and responsibilities

3.1 **Hospital and Health Services**

(a) The HHS will:

(i) provide, including the form and manner and at the times specified, the data specified in the data supply requirements (Attachment A to this Schedule 4) in accordance with this Schedule 4;

(ii) provide data in accordance with the provisions of the *Hospital and Health Boards Act 2011, Public Health Act 2005* and *Private Health Facilities Act 1999;*

[10] Section 143(2)(a) of the *Hospital and Health Boards Act 2011* provides that the disclosure of confidential information (as defined in s.139 of the Act) to the Chief Executive by an HHS under a service agreement is a disclosure permitted by an Act.

(iii) provide other HHSs with routine access to data, that is not Patient Identifiable Data, for the purposes of benchmarking and performance improvement;

(iv) provide data as required to facilitate reporting against the Performance Measures set out in Schedule 3 of this Service Agreement;

(v) provide data as specified within the provision of a health service directive;

(vi) provide activity data that complies with the national data provision timeframes required under the Independent Hospital Pricing Authority (IHPA) data plan for Commonwealth funding. Details of the timeframes are specified in the ‘Commonwealth Efficient Growth Funding and National Weighted Activity Units (NWAUs)’ specification sheet included in the supporting document Purchasing Policy and Funding Guidelines 2020/21 and the clinical placement data supply requirements; and

(vii) as requested by the Chief Executive from time to time, provide to the Chief Executive data, whether or not specified in this Schedule 4 or the Service Agreement, as specified by the Chief Executive in writing to the HHS in the form and manner and at the times specified by the Chief Executive.

(b) Data that is capable of identifying patients will only be disclosed as permitted by, and in accordance with, the *Hospital and Health Boards Act 2011, Public Health Act 2005 and the Private Health Facilities Act 1999.*

3.2 **Department**

The Department will:

(a) produce a monthly performance report which includes:

(i) actual activity compared with purchased activity levels;

(ii) any variance(s) from purchased activity;

(iii) performance information as required by the Department to demonstrate HHS performance against the Performance Measures specified in Schedule 3 of this Service Agreement; and

(iv) performance information as required by the Department to demonstrate the achievement of commitments linked to specifically allocated funding included in Schedule 2 of this Service Agreement.

(b) utilise the data sets provided for a range of purposes including:

(i) to fulfil legislative requirements;

(ii) to deliver accountabilities to state and commonwealth governments;

(iii) to monitor and promote improvements in the safety and quality of Health Services;

(iv) to support clinical innovation; and

(c) advise the HHS of any updates to data supply requirements as they occur.

Attachment A Data Supply Requirements

The HHS should refer to the relevant minimum data set for full details. These are available on-line as referenced in Appendix 1.

Table 12 Clinical data

Data Set	Data Custodian
Aged Care Assessment Team data via the Aged Care Evaluation (ACE) database	Strategic Policy Unit
Alcohol Tobacco and Other Drug Treatment Services	Mental Health Alcohol and Other Drugs Branch
Alcohol and Other Drugs Establishment Collection	Mental Health Alcohol and Other Drugs Branch
Allied Health Clinical Placement Activity Data	Allied Health Professions Office of Queensland
Australian and New Zealand Intensive Care Society (ANZICS) Data Collection	Healthcare Improvement Unit
BreastScreening Clinical Data	Executive Director, Preventive Health Branch
Clinical Incident Data Set	Patient Safety and Quality Improvement Service
Clinical Placement Data (excluding Allied Health)	Workforce Strategy Branch
Consumer Feedback Data Set	Patient Safety and Quality Improvement Service
Elective Surgery Data Collection	Healthcare Improvement Unit
Emergency Data Collection	Healthcare Improvement Unit
Gastrointestinal Endoscopy Data Collection	Healthcare Improvement Unit
Hand Hygiene Compliance Data	Communicable Diseases Branch
Healthcare Infection Surveillance Data	Communicable Diseases Branch
Maternal Deaths	Queensland Maternal and Perinatal Quality Council (through Statistical Services Branch)
Mental Health Act Data	Mental Health Alcohol and Other Drugs Branch
Mental Health Activity Data Collection	Mental Health Alcohol and Other Drugs Branch
Mental Health Carer Experience Survey Collection	Mental Health Alcohol and Other Drugs Branch
Mental Health Establishments Collection	Mental Health Alcohol and Other Drugs Branch
Monthly Activity Collection (including admitted and non-admitted patient activity and bed availability data)	Statistical Services Branch
Newborn Hearing Screening	Children's Health Queensland
Notifications Data	Chief Health Officer
Patient Experience Survey Data	Patient Safety and Quality Improvement Service
Patient Level Costing and Funding Data	HHS Funding and Costing Unit
Perinatal Data Collection	Statistical Services Branch
Queensland Bedside Audit	Patient Safety and Quality Improvement Service
Queensland Health Non-Admitted Patient Data Collection	Statistical Services Branch
Queensland Hospital Admitted Patient Data Collection	Statistical Services Branch
Queensland Needle and Syringe Program (QNSP) data	Chief Health Officer
Queensland Opioid Treatment Program Admissions and Discharges	Chief Health Officer
Radiation Therapy Data Collection	Healthcare Improvement Unit

Data Set	Data Custodian
Residential Mental Health Care Collections	Mental Health Alcohol and Other Drugs Branch
Schedule 8 Dispensing data	Chief Health Officer
School Immunisation Program – Annual Outcome Report	Communicable Diseases Branch
Specialist Outpatient Data Collection	Healthcare Improvement Unit
National Notifiable Diseases Surveillance System	Chief Health Officer
Vaccination Administration data	Chief Health Officer
Variable Life Adjusted Display (VLAD) CM (collection of hospital investigations)	Patient Safety and Quality Improvement Service
Your Experience of Service (YES) Survey Collection (Mental Health)	Mental Health Alcohol and Other Drugs Branch

Table 13 Non-clinical data

Non-Clinical Data Set	Data Custodian
Asbestos management data	Capital and Asset Services Branch
Asset Management<br>• Planning<br>• Maintenance<br>• Maintenance Budget<br>• Statement of Building Portfolio Compliance<br>• Benchmarking & Performance Data	Capital and Asset Services Branch
Conduct and Performance Excellence (CaPE)	Human Resources Branch
Expenditure	Finance Branch
Financial and Residential Activity Collection (FRAC)	Statistical Services Branch
Graduate Nursing Recruitment Data Statewide using the Public Service Commission Graduate Portal System	Office of the Chief Nursing and Midwifery Officer
Hospital Car Parks (including Government Portfolio Model funding arrangements)	Capital and Asset Services Branch
Minimum Obligatory Human Resource Information (MOHRI)	Finance Branch
Minor Capital Funding Program expenditure & forecast data	Finance Branch
Recruitment Data	Human Resources Branch
Revenue	Finance Branch
Queensland Health Workforce & Work Health & Safety Data	Human Resources Branch
Queensland Integrated Safety Information Project (QISIP)Solution Minimum Data Set	Human Resources Branch
Statewide employment matters	Human Resources Branch
Sustaining Capital Reporting Requirements (other than minor capital)	Capital and Asset Services Branch
Whole of Government Asset Management Policies data	Capital and Asset Services Branch

Schedule 5 Amendments to this Service Agreement

1. Purpose

This Schedule 5 sets out the mechanisms through which this Service Agreement may be amended during its term, consistent with the requirements of the *Hospital and Health Boards Act 2011.*

2. Principles

2.1 It is acknowledged that the primary mechanism through which HHS funding adjustments are made is through the budget build process that is undertaken annually in advance of the commencement of the financial year. This approach is intended to provide clarity, certainty and transparency in relation to funding allocations.

2.2 Amendments to the clauses of this Service Agreement should be progressed for consideration as part of the annual budget build process.

2.3 It is recognised that there is a requirement to vary funding and activity in-year. The following principles will guide amendments and amendment processes:

(a) funding allocations to HHSs should occur as early as possible within a financial year if unable to be finalised in advance of a given financial year;

(b) the number of Amendment Windows each year should be minimised to reduce the administrative burden on HHSs and the Department;

(c) Amendment Proposals should be minimised wherever possible and should always be of a material nature;

(d) Amendment Windows 2 and 3 are not intended to include funding or activity variations that could have been anticipated in advance of the financial year;

(e) Amendment Windows are intended to provide a formal mechanism to transact funding or activity variations in response to emerging priorities;

(f) Extraordinary Amendment Windows are not intended to be routinely used.

2.4 The Department remains committed to the ongoing simplification and streamlining of amendment processes.

3. Process to amend this Service Agreement

3.1 The Parties recognise the following mechanisms through which an amendment to this Service Agreement can be made:

(a) Amendment Windows;

(b) Extraordinary Amendment Windows;

(c) periodic adjustments; and

(d) end of year financial adjustments.

3.2 Amendment Windows

(a) In order for the Department to manage amendments across all HHS Service Agreements and their effect on the delivery of Public Sector Health Services in Queensland, proposals to amend this Service Agreement will be negotiated and finalised during set periods of time during the year (Amendment Windows).

(b) Amendment Windows are the primary mechanism through which amendments to this Service Agreement are made.

(c) Amendment Windows occur three times within a given financial year:

(i) Amendment Window 1: Annual Budget Build;

(ii) Amendment Window 2: In-year variation; and

(iii) Amendment Window 3: In-year variation.

(d) A Party that wants to amend the terms of this Service Agreement must give an Amendment Proposal to the other party.

(e) While a Party may submit an Amendment Proposal at any time, an Amendment Proposal will only be formally negotiated and resolved during one of the Amendment Windows outlined in Table 14 (excluding Extraordinary Amendment Windows).

Table 14 Amendment Window Exchange Dates

Amendment Window	Exchange Date	Primary Focus
Amendment Window 2: In-year variation	4 October 2019	2019/20 in-year variations
Amendment Window 3: In-year variation	14 February 2020	2019/20 in-year variations
Amendment Window 1: Annual Budget Build	27 March 2020	2020/21 budget build
Amendment Window 2: In-year variation	9 October 2020	2020/21 in-year variations
Amendment Window 3: In-year variation	12 February 2021	2020/21 in-year variations
Amendment Window 1: Annual Budget Build	26 March 2021	2021/22 budget build
Amendment Window 2: In-year variation	8 October 2021	2021/22 in-year variations
Amendment Window 3: In-year variation	11 February 2022	2021/22 in-year variations

(f) An Amendment Proposal is made by:

(i) the responsible Deputy Director-General signing and providing an Amendment Proposal to the HHS-SA Contact Person through Healthcare Purchasing and System Performance Division prior to the commencement of any Amendment Window; or

(ii) the Health Service Chief Executive signing and providing an Amendment Proposal to the D-SA Contact Person prior to the commencement of any Amendment Window.

(g) A Party giving an Amendment Proposal must provide the other Party with the following information:

(i) the rationale for the proposed amendment;

(ii) the precise drafting for the proposed amendment;

(iii) any information and documents relevant to the proposed amendment; and

(iv) details and explanation of any financial, activity or service delivery impact of the amendment.

(h) Negotiation and resolution of Amendment Proposals will occur during the Negotiation Period through a tiered process, as outlined in Figure 3.

Figure 3 Amendment Proposal negotiation and resolution

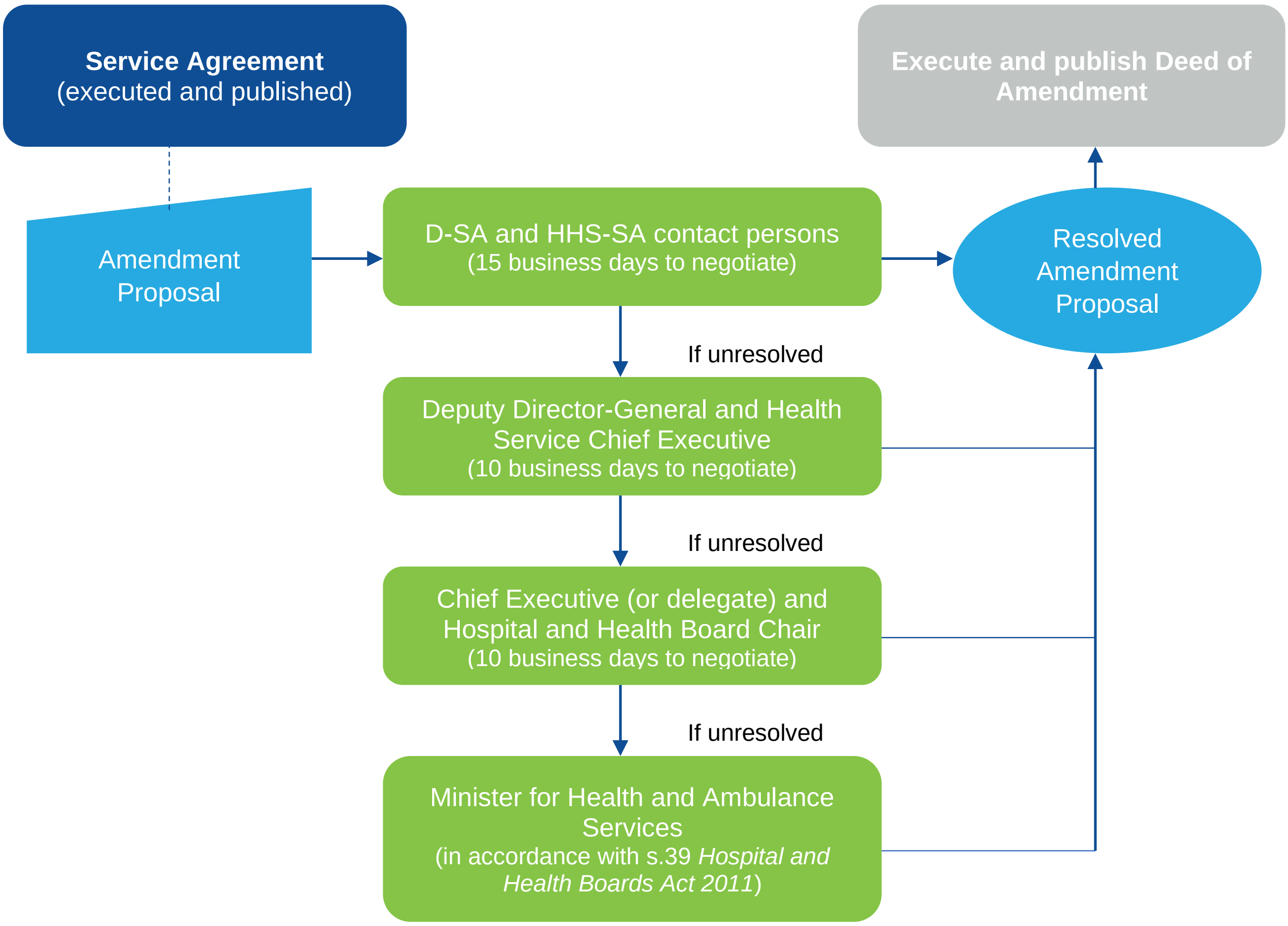

(i) The timeframes identified in Figure 3 may be reduced in the complete discretion of the Chief Executive.

(j) In accordance with section 39(5) of the *Hospital and Health Boards Act 2011*, the Parties must include any terms decided by the Minister in the Service Agreement.

(k) If the Chief Executive at any time:

(i) considers that an amendment agreed with the HHS may or will have associated impacts on other HHSs; or

(ii) considers it appropriate for any other reasons,

then the Chief Executive may:

(iii) propose further amendments to any HHS affected; and

(iv) may address the amendment and/or associated impacts of the

amendment in other ways, including through the exercise of any statutory powers and/or statutory directions under the *Hospital and Health Boards Act 2011*.

(l) Amendment Proposals that are resolved will be documented in a Deed of Amendment to this Service Agreement and executed by the Parties.

(m) Only upon execution of a Deed of Amendment by the Parties will the amendments documented by that Deed of Amendment be deemed to be an amendment to this Service Agreement.

3.3 **Extraordinary Amendment Windows**

(a) A Party that wants to amend the terms of this Service Agreement outside of an Amendment Window outlined in Table 14 must give an Extraordinary Amendment Proposal to the other Party.

(b) An Extraordinary Amendment Proposal may only be formally negotiated and resolved outside of an Amendment Window outlined in Table 14 to facilitate funding allocations where an urgent priority needs to be addressed in a timely manner and an Amendment Window is not available within an acceptable timeframe.

(c) An Extraordinary Amendment Proposal that is issued by or on behalf of the Chief Executive must be given to the HHS-SA Contact Person through Healthcare Purchasing and System Performance Division.

(d) An Extraordinary Amendment Proposal that is issued by or on behalf of the HHS must be given to the D-SA Contact Person through Healthcare Purchasing and System Performance Division.

(e) An Extraordinary Amendment Proposal may be issued by or on behalf of either Party at any time, noting the requirement that it relate to an urgent priority that necessitates timely resolution.

(f) Negotiation and resolution of Extraordinary Amendment Proposals will be through a tiered process as outlined in Figure 3.

(g) The timeframes identified in Figure 3 may be reduced in the complete discretion of the Chief Executive.

(h) In accordance with section 39(5) of the *Hospital and Health Boards Act 2011*, the Parties must include any terms decided by the Minster in the Service Agreement.

(i) Extraordinary Amendment Proposals that are resolved must be executed by both Parties.

(j) The Parties must comply with the terms of the Extraordinary Amendment Proposal from the date that the final Party executed the Extraordinary Amendment Proposal.

(k) The terms of an executed Extraordinary Amendment Proposal will be documented in a Deed of Amendment to this Service Agreement and executed by the Parties. Once executed, the Deed of Amendment will expressly exclude the application of the Extraordinary Amendment Proposal and only the terms of the Deed of Amendment will apply.

3.4 Periodic adjustments

(a) The Service Agreement Value may be adjusted outside of an Amendment Window to allow for funding variations that:

(i) occur on a periodic basis;

(ii) are referenced in the Service Agreement; and

(iii) are based on a clearly articulated formula.

(b) Adjustments to the Service Agreement Value and purchased activity that are required as a result of a periodic adjustment will be made following agreement between the Parties of the data on which the adjustment is based.

(c) The Chief Executive (or delegate) will issue an Adjustment Notice to the HHS-SA Contact Person confirming any funding adjustment that has been made.

(d) Following receipt of an Adjustment Notice, the Parties will comply with the Adjustment Notice and immediately take the steps necessary to give effect to the requirements of the Adjustment Notice.

(e) A Deed of Amendment will not be issued immediately following periodic adjustment. The HHS will be provided with a summary of all transactions made through periodic adjustment on completion.

(f) Any funding adjustments agreed through periodic adjustment which result in a variation to the Service Agreement Value, purchased activity or the requirements specified within Schedule 2 of this Service Agreement will be formalised in a Deed of Amendment issued following the next available Amendment Window.

3.5 End of financial year adjustments

(a) End of year financial adjustments may be determined after the financial year end outside of the Amendment Window process.

(b) The scope will be defined by the Department and informed by Queensland Government Central Agency requirements.

(c) The Department will provide the HHS with a reconciliation of all Service Agreement funding and purchased activity for the prior financial year. This will reflect the agreed position between the Parties following conclusion of the end of year financial adjustments process.

(d) The impact of end of year financial adjustments on subsequent year funding and activity will be incorporated in the Service Agreement through the Deed of Amendment executed following the next available Amendment Window.

(e) This clause will survive expiration of this Service Agreement.

Schedule 6 Definitions

In this Service Agreement:

Activity Based Funding (ABF) means the funding framework for publicly-funded health care services delivered across Queensland. The ABF framework applies to those Queensland public sector health service facilities which are operationally large enough and have the systems which are required to support the framework. The ABF framework allocates health funding to these hospitals based on standardised costs of health care services (referred to as 'activities') delivered. The Framework promotes smarter health care choices and better care by placing greater focus on the value of the health care delivered for the amount of money spent.

Adjustment Notice means the written notice of a proposed funding adjustment made by or on behalf of the Chief Executive in accordance with the terms of this Service Agreement.

Administrator of the National Health Funding Pool means the position established by the *National Health Reform Amendment (Administrator and National Funding Body) Act 2012* for the purposes of administering the National Health Funding Pool according to the National Health Reform Agreement.

Agreement means this Service Agreement.

Ambulatory Care means the care provided to hospital patients who are not admitted to the hospital, such as patients of emergency departments and outpatient clinics. Can also be used to refer to care provided to patients of community-based (non-hospital) healthcare services.

Amendment Proposal means the written notice of a proposed amendment to the terms of this Service Agreement as required under section 39 of the *Hospital and Health Boards Act 2011*.

Amendment Window means the period within which Amendment Proposals are negotiated and resolved. Amendment Windows commence on the relevant Exchange Date as specified in Table 14 Schedule 5 and end at the conclusion of the Negotiation Period.

Block Funding means funding for those services which are outside the scope of ABF.

Business Day means a day which is not a Saturday, Sunday or public holiday in Brisbane.

Chair means the Chair of the Hospital and Health Board.

Chief Executive means the chief executive of the Department.

Clinical Product/Consumable means a product that has been Clinically Prescribed.

Clinically Prescribed means prescribed by appropriately qualified and credentialed clinicians relative to the product.

Clinical Prioritisation Criteria means Statewide minimum criteria to determine if a referral to specialist medical or surgical outpatients is appropriate and, if so, the urgency of that referral.

Clinical Services Capability Framework means the Clinical Services Capability Framework for Public and Licensed Private Health Facilities which provides a standard set of minimum capability criteria for service delivery and planning. The Framework outlines the minimum service requirements, staffing, support services and risk considerations for both public and private health services to ensure safe and appropriately supported clinical service delivery. It applies to all public and private licensed facilities in Queensland. References to the Clinical Services Capability

Framework in this Service Agreement mean the most recent approved version unless otherwise specified.

Community Health Service means non-admitted patient Health Services, excluding hospital outpatient services, typically delivered outside of a hospital setting.

Deed of Amendment means the resolved amendment proposals.

Department means the department administering the *Hospital and Health Boards Act 2011* (Qld), which, at the date of this Service Agreement is known as 'Queensland Health'. To avoid any doubt, the term does not include the Hospital and Health Services.

D-SA Contact Person means the position nominated by the Department as the primary point of contact for all matters relating to this Service Agreement.

Effective Date means1 July 2019.

Efficient Growth means the increased in-scope activity-based services delivered by a HHS measured on a year to year basis in terms of both the Queensland efficient price for any changes in the volume of services provided and the growth in the national efficient price of providing the existing volume of services.

Eligible Population (Oral Health Services) refers to the proportion of the population for whom publicly funded oral health services is to be provided and is defined by the following criteria:

(a) adults, and their dependents, who are Queensland residents; eligible for Medicare and, where applicable, currently in receipt of benefits from at least one of the following concession cards:

    (i) Pensioner Concession Card issued by the Department of Veteran's Affairs;

    (ii) Pensioner Concession Card issued by Centrelink;

    (iii) Health Care Card (this includes Low Income Health Care Card Holders who are automatically eligible for services);

    (iv) Commonwealth Seniors Health Card;

    (v) Queensland Seniors Card.

(b) children who are Queensland residents or attend a Queensland school, are eligible for Medicare, and are:

    (i) eligible for dental program/s funded by the Commonwealth Government; or

    (ii) four years of age or older and have not completed Year 10 of secondary school; or

    (iii) dependents of current concession card holders or hold a current concession card.

Exchange Date means the date on which the Parties must provide Amendment Proposals for negotiation, as specified in Table 14 Schedule 5.

Extraordinary Amendment Window means an Amendment Window that occurs outside of the Amendment Windows specified in Table 14 Schedule 5, in accordance with the provisions of clause 3.3 of Schedule 5.

Force Majeure means an event:

(a) which is outside of the reasonable control of the Party claiming that the event has occurred; and

(b) the adverse effects of which could not have been prevented or mitigated against by that Party by reasonable diligence or precautionary measures, and includes lightning, earthquake, fire, cyclone, flood, natural disasters, health pandemics, acts of terrorism, riots, civil disturbances, industrial disputes and strikes (other than strikes involving that Party, its' agents, employees or suppliers), war (declared or undeclared), revolution, or radioactive contamination.

Formal Agreement means an agreed set of roles and responsibilities relating to the provision and receipt of services designated as Statewide or Regional:

(a) Statewide or Regional service provision

- (i) ensure equitable and timely access to entire catchment (clinical and non-clinical)
- (ii) provide training and consultation Services where this is appropriate within the agreed model of care (clinical and non-clinical)
- (iii) timely discharge or return of patients to their place of residence (clinical Services)
- (iv) adequate communication practices to enable ongoing effective local health care, including with the patient's General Practitioner where required (clinical Services)

(b) Recipient HHS

- (i) utilisation of standardised referral criteria, where they exist, to ensure appropriate use of Statewide Services (clinical services)
- (ii) timely acceptance of patients being transferred out of Statewide Services (back-transfers) (clinical Services)
- (iii) equitable access to ongoing local health care as required (clinical services)

Health Executive means a person appointed as a health executive under section 67(2) of the *Hospital and Health Boards Act 2011.*

Health Service has the same meaning as set out in section 15 of the *Hospital and Health Boards Act 2011.*

Health Service Chief Executive means a health service chief executive appointed for an HHS under section 33 of the *Hospital and Health Boards Act 2011.*

Health Service Employee means all person, appointed as a 'health service employee' for the HHS under section 67(1) of the *Hospital and Health Boards Act 2011.*

Hospital and Health Board means the hospital and health board appointed under section 23 of the *Hospital and Health Boards Act 2011*.

Hospital and Health Service or **HHS** means the Hospital and Health Service to which this Agreement applies unless otherwise specified.

HHS-SA Contact Person means the position nominated by the HHS as the primary point of contact for all matters relating to this Service Agreement.

HR Management Functions means the formal system for managing people within the HHS, including recruitment and selection; onboarding; induction and orientation; capability, learning and development; industrial and employee relations; performance management; work health and safety and well-being; workforce planning; diversity and inclusion; and workforce consultation, engagement and communication.

Industrial Instrument means an industrial instrument made under the *Industrial Relations Act 2016.*

Inter-HHS Dispute means a dispute between two or more HHSs.

Key Performance Indicator means a measure of performance that is used to evaluate the HHSs success in meeting key priorities.

Low Benefit Care means use of an intervention where evidence suggests it confers no or very little benefit on patients, or the risk of harm exceeds the likely benefit.

Minister means the Minister administering the *Hospital and Health Boards Act 2011* (Qld).

National Health Reform Agreement means the document titled *National Health Reform Agreement* made between the Council of Australian Governments (CoAG) in 2011, and incorporating all subsequent amendments agreed between the Commonwealth of Australia and the States and Territories.

Negotiation Period means a period of no less than 15 business days (or such longer period agreed in writing between the Parties) from each Exchange Date.

Notice of Dispute means the written notice of a dispute provided by the Chief Executive or the HHS to the other party or the written notice of a dispute provided by an HHS to another HHS.

Outcome Indicator means a measure of performance that provides information on specific activities and interventions where there is a reasonable expectation or evidence that they make a positive contribution towards improving the health status and experience of patients;

Outreach Service means a Health Service delivered on sites outside of the HHS area to meet or complement local service need. Outreach services include Health Services provided from one HHS to another as well as Statewide Services that may provide Health Services to multiple sites.

Own Source Revenue means, as per Section G3 of the *National Healthcare Agreement*, ‘private patients, compensable patients and ineligible persons may be charged an amount for public hospital services as determined by the state and territory’. The funding for these patients is called own source revenue and includes:

(a) Medicare ineligible patients, such as overseas visitors (not covered under reciprocal agreements), people in community detention and overseas students studying in Australia

(b) compensable patients with an alternate funding source, such as:

- (i) workers’ compensation insurers;
- (ii) motor vehicle accident insurers;
- (iii) personal injury insurers;
- (iv) Department of Defence; and/or
- (v) Department of Veterans’ Affairs; and

Medicare eligible patients can elect to be treated as a public or private patient, allowing HHS' to recoup a portion of the healthcare service delivery cost.

Party means each of the Chief Executive and the HHS to which this Service Agreement applies.

Patient Identifiable Data means data that could lead to the identification of an individual either directly (for example by name), or through a combination of pieces of data that are unique to that individual.

Performance Review Meeting means the forum established which routinely reviews and discusses a range of aspects of HHS and system wide performance in accordance with the accountabilities contained within this Service Agreement and the Performance and Accountability Framework. Attendance at Performance Review Meetings comprises:

(a) the D-SA Contact Person and the HHS-SA Contact Person;

(b) executives nominated by the Department; and

(c) executives nominated by the HHS.

Performance Measure means a quantifiable indicator that is used to assess how effectively the HHS is meeting identified priorities and objectives.

Person Conducting a Business or Undertaking takes the meaning as defined in the *Work Health and Safety Act 2011,* section 5.

Prevention Services means programs that prevent illness and injury, promote health and wellbeing, create healthy and safe environments, reduce health inequalities and address factors in those communities whose health status is the lowest.

Primary Care means first level healthcare provided by a range of healthcare professionals in socially appropriate and accessible ways and supported by integrated referral systems. It includes health promotion, illness prevention, care of the sick, advocacy and community development.

Public Health Event of State Significance means an event where the actual or potential impact extends beyond the community service by a particular Hospital and Health Service.

Public Sector Health Service has the same meaning as set out in the *Hospital and Health Boards Act 2011.*

Public Sector Health System means the Queensland public sector health system, which is comprised of the Hospital and Health Services and the Department.

Quality Improvement Payment (QIP) means a non-recurrent payment due to the HHS for having met the goals set out in the QIP Purchasing Incentive Specification.

Queensland Government Central Agency means one or all of the Department of the Premier and Cabinet, Queensland Treasury, the Queensland Audit Office, the Public Service Commission and the Office of the Integrity Commissioner.

Regional Service means a clinical (direct or indirect patient care) or non-clinical Health Service funded and delivered, or coordinated and monitored, by an HHS with a catchment of two or more HHSs, but not on a Statewide basis as defined in this Schedule. Service delivery includes facility based, outreach and telehealth service models.

Referral Pathway means the process by which a patient is referred from one clinician to another in order to access the Health Services required to meet their healthcare needs.

Residential HHS means the HHS area, as determined by the *Hospital and Health Boards Regulation 2012*, in which the patient normally resides.

Safety and Quality Marker means a measure of performance that provides timely and transparent information on the safety and quality of Health Services provided by the HHS;

Schedule means this Schedule to the Service Agreement.

Senior Health Service Employee means a person appointed under section 67(2) of the *Hospital and Health Boards Act 2011* in a position prescribed as a 'senior health service employee position' under the *Hospital and Health Boards Regulation 2012.*

Service Agreement means this service agreement including the Schedules and annexures, as amended from time to time.

Service Agreement Value means the figure set out in Schedule 2 as the expected annual value of the services purchased by the Department through this Service Agreement.

State means the State of Queensland.

Statement of Building Portfolio Compliance means a declaration completed by the HHS stating that it has maintained compliance with all mandatory Acts, Regulations, Australian Standards and Codes of Practice applicable to the HHS' building portfolio.

Statewide Service means a service that is delivered by a lead provider to the State. A Statewide Service may be:

(a) a clinical service that is:

(i) a low volume, highly specialised Health Service delivered from a single location;

(ii) a highly specialised, or high risk[11], Health Service delivered in multiple locations or

(iii) a prevention and/or health promotion service.

(b) a support service that is required to enable the delivery of specific direct clinical services; or

(c) services that have a primary role to provide clinical education services and/or training programs.

Statewide Service Description means a document that defines the Service to be provided by the HHS on a statewide basis and how the Statewide Service will be accessed and used by other HHSs across the State, including but not limited to:

(a) an overview of the Statewide Service;

(b) components of the Statewide Service;

(c) eligibility criteria;

(d) Service referrals and pathways; and

(e) governance and capability arrangements for the Statewide Service.

---

[11] A Health Service that, due to its nature, poses an increased threat of ongoing sustainability, efficiency and affordability.

Supporting Indicator means a measure of performance that provides contextual information to support an assessment of HHS performance.

Suspend and Suspension means to cause the temporary cessation of a service provided by the HHS under the terms of this Service Agreement. Suspension may result from, but is not exclusively due to, limitations in workforce capacity or issues regarding the safety or quality of the service provided.

Telehealth means the delivery of Health Services and information using telecommunication technology, including:

(a) live interactive video and audio links for clinical consultations and education;

(b) store and forward systems to enable the capture of digital images, video, audio and clinical data stored on a computer and forwarded to another location to be studied and reported on by specialists;

(c) teleradiology to support remote reporting and provision of clinical advice associated with diagnostic images; and

(d) telehealth services and equipment for home monitoring of health.

Terminate and Termination means the permanent cessation of a service provided by the HHS under the terms of this Service Agreement.

Treating HHS means the HHS area, as determined by the *Hospital and Health Boards Regulation 2012*, in which a patient is receiving treatment.

Value-Based Healthcare means delivering what matters most to patients in the most efficient way. Value-Based Healthcare is characterised by:

(a) the identification of clearly defined population segments of patients with similar needs around which clinically integrated teams organise and deliver care, rather than designing and organising care around medical specialities, procedures or facilities;

(b) a focus on delivering health outcomes and the experience of receiving care as defined from the patient perspective, not just the system or clinical perspective;

(c) connection between outcomes and the costs required to deliver the outcomes; and

(d) an integrated approach across the full cycle of care with a focus on the goal of health rather than just treatment.

Key Documents

Hospital and Health Services Service Agreements and supporting documents including:

(a) Hospital and Health Services Service Agreements

(b) Queensland Health System Outlook to 2026 for a sustainable health service

(c) Performance and Accountability Framework 2020/21

(d) Purchasing Policy and Funding Guidelines 2020/21

are available at: www.health.qld.gov.au/system-governance/health-system/managing/agreements-deeds

My health, Queensland's future: Advancing health 2026

www.health.qld.gov.au/__data/assets/pdf_file/0025/441655/vision-strat-healthy-qld.pdf

Queensland Health 2020-2021 System Priorities

[link to follow]

Department of Health Strategic Plan

www.health.qld.gov.au/system-governance/strategic-direction/plans/doh-plan

Guideline for Immunisation Services

https://www.health.qld.gov.au/__data/assets/pdf_file/0026/147545/qh-gdl-955.pdf

Queensland Health Statement of Action towards Closing the Gap in health outcomes

https://qheps.health.qld.gov.au/atsihb/html/statement-of-action

HHS Performance Measures and Attribute Sheets

https://qheps.health.qld.gov.au/purchasing-performance/hhs-agreements/performance-kpis

Data Supply Requirements

https://qheps.health.qld.gov.au/purchasing-performance/hhs-agreements/data-reporting-requirements

Australian Commission on Safety and Quality in Healthcare – National Safety and Quality Health Service Standards

https://www.safetyandquality.gov.au/standards/nsqhs-standards

Statewide Services Governance and Risk Management Framework

https://qheps.health.qld.gov.au/spb/html/statewide-services/statewide-services-governance-and-risk-management-framework

Public Health Practice Manual

https://qheps.health.qld.gov.au/__data/assets/pdf_file/0035/667754/public-health-prac-man.pdf

National Healthcare Agreement

http://www.federalfinancialrelations.gov.au/content/national_agreements.aspx

National Health Reform Agreement

www.federalfinancialrelations.gov.au/content/national_health_reform.aspx

Abbreviations

ACQSC	Aged Care Quality and Safety Commission
ABF	Activity Based Funding
ACSQHC	Australian Commission on Safety and Quality in Healthcare
CET	Clinical Education and Training
D-SA	Department – Service Agreement
HHS	Hospital and Health Service
HHS-SA	Hospital and Health Service – Service Agreement
HITH	Hospital in the Home
KPI	Key Performance Indicator
LAM	List of Approved Medicines
Non-ABF	Non-Activity Based Funding
NPA	National Partnership Agreement
NSQHS	National Safety and Quality Health Service Standards
NWAU	National Weighted Activity Unit
PBS	Pharmaceutical Benefits Scheme
QAS	Queensland Ambulance Service
QIP	Quality Improvement Payment
QWAU	Queensland Weighted Activity Unit
RACGP	Royal Australian College of General Practitioners
SA2	Statistical Area Level 2

This page is intentionally left blank.

Queensland Health
www.health.qld.gov.au

Queensland Government

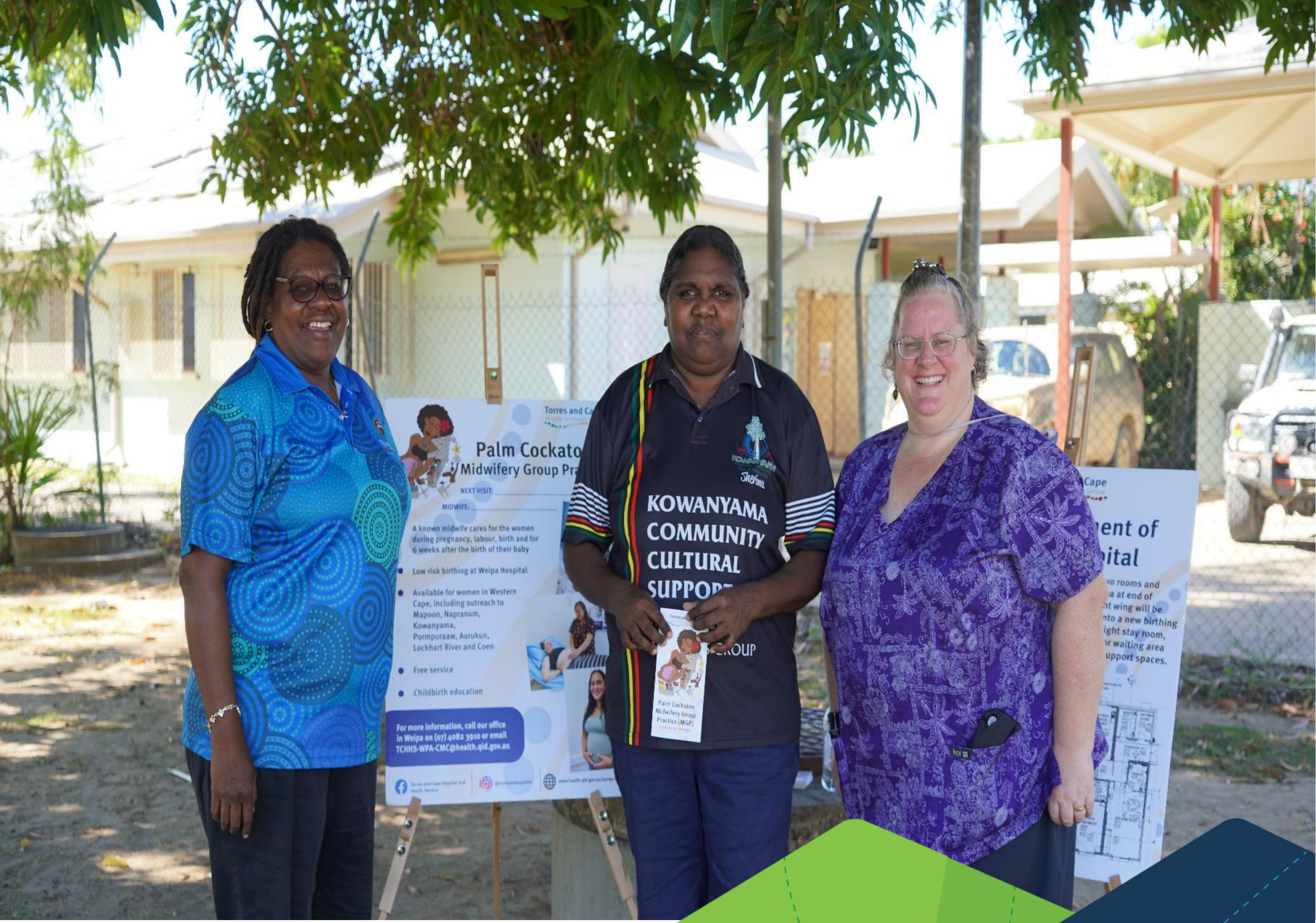

Queensland Health

Service Agreement 2022/23 – 2024/25

Torres and Cape Hospital and Health Service

Queensland Government

Torres and Cape Hospital and Health Service, Service Agreement 2022/23 - 2024/25

Published by the State of Queensland, (Queensland Health), July 2022

This document is licensed under a Creative Commons Attribution 3.0 Australia licence. To view a copy of this licence, visit creativecommons.org/licenses/by/3.0/au

© State of Queensland (Queensland Health) **2022**

You are free to copy, communicate and adapt the work, as long as you attribute the State of Queensland (Queensland Health).

For more information contact:

Contracting and Performance Management Branch, Department of Health, GPO Box 48, Brisbane QLD 4001
Tel (+61) (07) 3708 5869

Email SAM@health.qld.gov.au

An electronic version of this document is available at www.health.qld.gov.au/system-governance/health-system/managing/default.asp

Disclaimer:
The content presented in this publication is distributed by the Queensland Government as an information source only. The State of Queensland makes no statements, representations or warranties about the accuracy, completeness or reliability of any information contained in this publication. The State of Queensland disclaims all responsibility and all liability (including without limitation for liability in negligence) for all expenses, losses, damages and costs you might incur as a result of the information being inaccurate or incomplete in any way, and for any reason reliance was placed on such information.

Acknowledgement

Torres and Cape Hospital and Health Service acknowledges and respects the Traditional Owners of the land on which we live and work and acknowledges their continuing connection to the land and community which we serve. We pay respect to them, their culture, and their Elders past, present and future.

We acknowledge the First Nations people in Queensland are both Aboriginal Peoples and Torres Strait Islander Peoples, and support the cultural knowledge, determination and commitment of Aboriginal and Torres Strait Islander communities in caring for health and wellbeing for millennia.

Torres and Cape Hospital and Health Service is proud to recognise and celebrate the cultural diversity of our communities and workforce and we acknowledge the following Traditional Owners and Custodians of the lands within the Torres and Cape:

- Ayabadhu
- Alngith
- Anathangayth
- Anggamudi
- Apalech
- Atambaya
- Angkamuthi
- Binthi
- Burunga
- Dingaal
- Girramay
- Gulaal
- Gugu Muminh
- Guugu-Yimidhirr
- Gudang
- Gudamaluilgal
- Kaantju
- Koko-bera
- Kokomini
- Kuku Taypan
- Kuku Yalanji
- Kunjen/Olkol
- Kuuku – Yani
- Kaiwalagal
- Kulkalgal
- Wik
- Wik Mungkan
- Wimarangga
- Yadhaykenu
- Lama Lama
- Mpalitjanh
- Munghan
- Maluilgal
- Meriam
- Ngaatha
- Ngayimburr
- Ngurrumungu
- Nugal
- Oolkoloo
- Oompala
- Peppan
- Puutch
- Sara
- Teppathiggi
- Thaayorre
- Thanakwithi
- Thiitharr
- Thuubi
- Tjungundji
- Uutaalnganu
- Wanam
- Warrangku
- Wathayn
- Waya
- Winchanam
- Wuthathi
- Yupungathi

Contents

Acknowledgement 3
Contents 4
Tables 5
1. Introduction 6
2. Scope 6
3. Services 6
4. Performance and Accountability Framework 7
5. Data supply requirements 7
6. Hospital and Health Service accountabilities 8
7. Department accountabilities 9
8. Achieving health equity with First Nations Queenslanders 9
9. General 10
10. Counterparts 11
Execution 12
Schedule 1 HHS profile 13
1. HHS profile 13
Schedule 2 Funding and purchased activity and services 14
1. Introduction 14
2. Purchased health services 15
3. Teaching training and research 17
4. Delivery of purchased activity 18
5. Delivery of purchased services 19
6. Financial adjustments 19
7. Funding sources 23
8. Funds disbursement 24
Schedule 3 Performance Measures 29
1. Performance Measures 29
Schedule 4 Amendments to this service agreement 31
1. Agreed process to amend this service agreement 31
Appendix 1 Reference Documents 33

Tables

Table 1 Statewide Services 15
Table 2 Financial adjustments applied on breach of activity thresholds 20
Table 3 Purchasing Incentives 2022/23 21
Table 4 Torres and Cape HHS Total Funding Allocation by Funding Source 2022/23 25
Table 5 National Health Reform Funding 27
Table 6 Discretely Funded Programs (Non-ABF) 28
Table 7 HHS Performance Measures – Key Performance Indicators 29
Table 8 HHS Performance Measures - Safety and Quality Markers 30
Table 9 HHS Performance Measures – Outcome Indicators 30

1. Introduction

1.1 In performing this service agreement, the Department and the Hospital and Health Service (HHS) will act consistently with the object of the *Hospital and Health Boards Act 2011* (Qld) (Act).

1.2 The Department and the HHS operate as part of a networked system and agree to work collaboratively with each other, with other HHSs and with the Queensland Ambulance Service in the best interests of the Queensland public sector health system.

1.3 The parties will ensure that planning and delivery of health services is consistent with the strategies and priorities set out by government. The parties recognise the importance of the HHS' Health Equity Strategy (as defined in the Act) and the parties commitment to improving health and wellbeing outcomes and achieving health equity for Aboriginal and Torres Strait Islander peoples.

2. Scope

2.1 This service agreement covers the period from 1 July 2022 to 30 June 2025.

2.2 The parties have identified the services to be provided by the HHS, the funding for the provision of those services, the performance measures applicable to the services and data requirements.

3. Services

3.1 In delivering services, the HHS is required to meet:

(a) the applicable conditions of all national agreements and national partnership agreements between the Queensland Government and the Commonwealth Government and commitments under any related implementation plans; and

(b) the applicable conditions of each agreement or arrangement for funding between the Department and the HHS and commitments under any related implementation plans, which conditions and commitments may be recorded separately to this service agreement but for which funding is (or becomes) provided in Schedule 2.

3.2 The HHS is required to deliver the services outlined in this service agreement for which funding is provided in Schedule 2.

3.3 Where issues arise which prevent the HHS from providing a service or necessitate a reduction in the level or scope of a service provided, prompt notification must be made to the Department and impacted HHSs, with appropriate details. The HHS must minimise any clinical risk or adverse impact to patient experience that may result from service disruption. The Department will respond to the HHS on any requirements it has concerning service delivery and any adjustments triggered by under delivery.

3.4 If the HHS wishes to terminate or reduce service levels for a service for which funding is provided in Schedule 2, this will remain subject to negotiation and agreement by the Department at its discretion.

3.5 For any new services proposed during the term of this service agreement, the parties agree:

(a) the service must meet a demonstrated clinical need and provide value for money;

(b) the commencement of a new service, including the implementation of new models of care, may occur where the service has been commissioned by the Department, a funding stream is in place and any conditions relating to the funding have already been agreed; and

(c) if the HHS wishes to commence providing a new service that has not been commissioned by the Department, this will remain subject to agreement by the Department at its discretion.

3.6 It is acknowledged that there may from time to time need to be service delivery changes between HHSs. Management of inter-HHS relationships should be informed by the following principles:

(a) HHSs should maintain the proportion of out of HHS work undertaken unless as a result of agreed repatriation of patients;

(b) each HHS should manage patients from its own catchment population if it is within its clinical capability to do so as specified by the Clinical Services Capability Framework; and

(c) where it is proposed that a service move from one HHS to another, the Department will consider, as part of its review under clause 3.4 and 3.5, whether the respective Health Service Chief Executives endorse the proposed change in patient flows; and the funding required to follow the patient.

4. Performance and Accountability Framework

4.1 The Queensland Health Performance and Accountability Framework (the Performance and Accountability Framework) sets out the framework within which the Department monitors and manages the performance of public sector health services in Queensland.

4.2 The parties will act consistently with the Performance and Accountability Framework.

5. Data supply requirements

5.1 The HHS will provide the Department with all clinical and non-clinical data that is reasonably required to support the effective management of the public sector health system. This will include, but is not limited to, data that is required to:

(a) fulfil legislative obligations;

(b) deliver accountabilities and obligations to State and Commonwealth Governments; including related to the provision and reconciliation of activity data by the Administrator of the National Health Funding Pool;

(c) monitor and support performance improvement;

(d) manage this service agreement;

(e) support clinical innovation; and

(f) facilitate evaluation and audit.

5.2 The parties agree and acknowledge that:

(a) the Department will keep the HHS informed of the Department's data requirements; and

(b) data will be provided as required, or permitted, by law.

6. Hospital and Health Service accountabilities

6.1 The HHS will perform its obligations under this service agreement.

6.2 As applicable to the HHS and its services, the HHS will comply with:

(a) legislation and subordinate legislation, including the Act;

(b) cabinet decisions;

(c) Ministerial directives;

(d) agreements entered into between the Queensland and Commonwealth governments (or agreements with others in furtherance of such agreements), of which it is informed;

(e) agreements entered into between the Department and other Queensland Government entities, of which it is informed;

(f) all industrial instruments;

(g) all health service directives and health employment directives; and

(h) all policies, guidelines and implementation standards, including human resource policies.

6.3 As part of the commitment to achieving First Nations health equity, the HHS will prioritise the elimination of racial discrimination and institutional racism within its service and ensure that Aboriginal and Torres Strait Islander peoples have access to culturally safe and responsive health services.

6.4 The HHS will ensure that effective health service planning and delivery systems are in place, working in collaboration with the Department.

6.5 The HHS will ensure that health service employees employed by the Chief Executive[1] who perform work for the HHS are managed in accordance with any applicable delegations and directions from the Chief Executive.

6.6 The HHS will ensure that effective asset management systems are in place, working in collaboration with the Department.

6.7 The HHS will maintain accreditation to the standards required by the Department.

6.8 The HHS will appropriately perform and fulfil its functions under the Act.

6.9 The HHS will provide to the Chief Executive reports of a type, and at the intervals, agreed between the parties, or as reasonably specified by the Chief Executive.

7. Department accountabilities

7.1 The Department will perform its obligations under this service agreement including, in return for the HHS performing its obligations and delivering the services, providing funding to the HHS as stipulated in this service agreement (as amended).

7.2 The Department will:

(a) comply with applicable legislation and subordinate legislation, including the Act, as relates to this service agreement; and

(b) perform the system manager role (as defined in the Act) through the Chief Executive;

7.3 The Chief Executive will appoint health service employees to perform work for the HHS for the purpose of enabling the HHS to perform its functions under the Act.

7.4 The Chief Executive will consult, cooperate, and coordinate with the HHS to ensure legal compliance with the *Work Health and Safety Act 2011* and other legislation as it applies to the scope, nature and location of operations associated with this service agreement.

7.5 The Chief Executive will appropriately perform and fulfil their functions under the Act.

8. Achieving health equity with First Nations Queenslanders

8.1 Through legislative amendments to the Act and the Hospital and Health Boards Regulation 2012, and the release of *Making Tracks Together: Queensland's Aboriginal and Torres Strait Islander Health Equity Framework* (2021), Queensland Health has strengthened its commitment to improving health and wellbeing outcomes and achieving health equity for First Nations peoples.

8.2 The HHS will develop a Health Equity Strategy to demonstrate the HHS's activities and key performance measures to achieve health equity with First Nations peoples that is compliant with legislative requirements. The Health Equity Strategy will act as the principal

[1] In this service agreement the term Chief Executive takes the meaning applied in the Act, which is the Chief Executive of Queensland Health, but which is generally referred to as the Director-General.

accountability mechanism between community and the HHS in achieving health equity for First Nations Queenslanders and forms a key part of the Queensland Government's commitment to the *National Agreement on Closing the Gap* (2020).

8.3 The HHS will review the Health Equity Strategy at least once every three years and will publish the Health Equity Strategy in a way that allows it to be accessed by members of the public.

8.4 The HHS will ensure that commitment and leadership is demonstrated through implementing the actions and achieving the key performance measures outlined in the Health Equity Strategy.

8.5 The HHS will report publicly on progress against the Health Equity Strategy.

8.6 The HHS will support the implementation of other supplementary policies and strategies to drive health equity across the public health system, including relevant election commitments.

8.7 The HHS will participate as a partner in the design, development and implementation of the new *Queensland First Nations Health Workforce Strategy for Action.*

9. General

9.1 Sub-contracting

(a) The HHS must have appropriate systems in place to ensure that any subcontractor is accredited (as applicable), qualified, and otherwise fit to perform any services for which it is contracted.

(b) The HHS must ensure that any sub-contractor who has access to confidential information (as defined in the Act) or personal information (as defined in the *Information Privacy Act 2009* (Qld)) complies with obligations no less onerous than those imposed on the HHS.

9.2 Insurance

The HHS must:

(a) hold and maintain the types and levels of insurances that the HHS considers appropriate according to its functions and obligations; and

(b) comply with reasonable requests or directions from the Department in this regard.

9.3 Amendment

The process for amending this service agreement is provided for under the Act and further outlined in Schedule 4.

10. Counterparts

10.1 This service agreement may be executed in two or more identical copy counterparts, each of which together will be deemed an original, but all of which together will constitute one and the same instrument.

10.2 In the event that any signature executing this service agreement or any part of this service agreement is delivered by facsimile transmission or by scanned e-mail delivery of a '.pdf' format data file or equivalent of the entire agreement, the signature will create a valid and binding obligation of the party executing (or on whose behalf the signature is executed) with the same force and effect as if the signature page were an original. For the avoidance of doubt, this service agreement may be in the form of an electronic document and may be electronically signed.

10.3 For execution under this clause 10 to be valid the entire service agreement upon execution by each individual party must be delivered to the remaining parties.

Execution

Executed as an agreement in Queensland

Signed by the Chief Executive, Queensland Health:)
)

....

Signature of Chief Executive

SHAUN DRUMMOND

....

Name of Chief Executive (print)

....

(date)

Signed for and on behalf of the Torres and Cape Hospital and Health Service:)
)

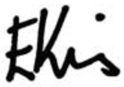

Elthies Kris
cn=Elthies Kris, o=TCHHS, ou=Board Chair, email=TCHHS-Board-Chair@health.qld.gov.au, c=AU

....

Signature of Hospital and Health Board Chair

Elthies Kris

....

Name of Hospital and Health Board Chair (print)

20 June 2022

....

(date)

Execution

Executed as an agreement in Queensland

Signed by the Chief Executive, Queensland Health:)

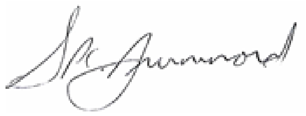

Signature of Chief Executive

SHAUN DRUMMOND

Name of Chief Executive (print)

29 June 2022

(date)

Signed for and on behalf of the Torres and Cape Hospital and Health Service:)

Signature of Hospital and Health Board Chair

Name of Hospital and Health Board Chair (print)

(date)

Schedule 1 HHS profile

1. HHS profile

Bordering with Papua New Guinea, Torres and Cape HHS provides public healthcare services to approximately 28,000 people spread widely across Cape York, the Northern Peninsula Area and the Torres Strait Islands. Sixty-seven percent of the population identify as Aboriginal and/or Torres Strait Islander (source: *Torres and Cape Hospital and Health Service Strategic Plan 2019 -2023 (version 2021)*

Services are provided from a wide range of locations, many with a primary care focus. The HHS also supports outreach teams and visiting specialist services from other HHSs along with non-government providers.

Close partnerships between the HHS and a range of local organisations are instrumental to supporting the shared goal of delivering safe, sustainable and innovative services closer to home and improving health outcomes in the region. The HHSs key local partners include:

- Apunipima Cape York Health Council
- NPA Family & Community Services (Aboriginal and Torres Strait Islander Corporation)
- Torres Health Indigenous Corporation
- Northern Queensland Primary Health Network
- Royal Flying Doctor Service
- CheckUp
- Northern Aboriginal and Torres Strait Islander Health Alliance (NATSIHA)
- Queensland Aboriginal and Torres Strait Islander Health Council (QAIHC)
- Queensland Ambulance Service

Schedule 2 Funding and purchased activity and services

This Schedule 2 sets out:

(a) the services which are to be provided by the HHS;

(b) the activity purchased by the Department from the HHS;

(c) the funding provided for delivery of the purchased activity;

(d) the criteria and processes for financial adjustments associated with the delivery of purchased activity and specific funding allocations;

(e) the sources of funding that this service agreement is based on and the manner in which these funds will be provided to the HHS.

1. Introduction

1.1 The HHS will deliver the services for which funding is provided in this Schedule 2. In providing these services, the HHS will ensure that:

(a) all statewide and national policy frameworks, guidelines, protocols and implementation standards applicable to the service provided are followed;

(b) participation in national programs is facilitated and supported, including where these programs are provided by the Commonwealth Government;

(c) service delivery partnerships, including with other HHSs, primary care organisations and non-government organisations, are maintained and operate effectively;

(d) collaboration and engagement with other service providers and stakeholders is initiated and maintained to ensure that an integrated system of treatment, care and support is in place and to facilitate the delivery of comprehensive and effective services. This may include but is not limited to:

(i) other HHSs;

(ii) non-government organisations;

(iii) Aboriginal and Torres Strait Islander community-controlled health organisations;

(iv) Queensland Ambulance Service;

(v) services provided through the Department of Health (for example, Pathology Queensland);

(vi) primary care providers;

(vii) other government departments and agencies; and

(viii) private providers;

(e) models of care and service delivery arrangements are consistent with evidence-based practice and offer value for money;

(f) services are provided on an equitable basis to the community, and processes are in place to ensure that services reach identified target populations, high risk groups and hard to reach communities;

(g) referral networks and pathways continue to operate effectively; and

(h) innovation and continuous improvement is supported.

2. Purchased health services

2.1 Table 4, Table 5 and Table 6 outline the activity and service streams which the Department agrees to purchase from the HHS pursuant to this service agreement.

2.2 More generally, this will include the following:

2.3 **Statewide Services**

(a) The designation of a service as a statewide service (either a clinical statewide service or a clinical support statewide service) will be determined by the Department, consistent with the stipulated governance arrangements for such services.

(b) The HHS will:

(i) collaborate with the Department and other HHSs in the implementation of, and adherence to, the governance and oversight arrangements for statewide services;

(ii) participate in, and contribute to, the staged review of the purchasing model for identified statewide services; and

(iii) ensure that referral pathways in and out of each statewide service are followed.

2.4 **Clinical Statewide Services and Clinical Support Statewide Services provided**

This clause does not apply to this HHS.

Table 1 Statewide Services

This table does not apply to this HHS.

2.5 **Regional services**

This clause does not apply to this HHS.

2.6 **Prevention services and population health services**

(a) The HHS will provide a range of services with a focus on the prevention of ill-health and disease, including:

(i) Specialist Public Health Units;

(ii) preventive health services;

(iii) immunisation services;

(iv) sexually transmissible infections including HIV and viral hepatitis;

(v) tuberculosis services; and

(vi) population health screening including, but not limited to, cancer screening services and newborn blood spot screening.

(b) Services will be provided in line with public health related legislation and the service delivery and reporting requirements outlined in the *Public Health Practice Manual*, as these relate to the services provided.

2.7 Aboriginal and Torres Strait Islander health services

The HHS will provide Aboriginal and Torres Strait Islander specific health services and initiatives consistent with the principles and objectives of the Queensland Government's *Making Tracks towards closing the gap in health outcomes for Indigenous Queenslanders by 2022 – Policy and Accountability Framework*. These service and initiatives will be delivered in line with guidance from the Aboriginal and Torres Strait Islander Health Division.

2.8 Mental health alcohol and other drugs services

The HHS will provide treatment, care and support for individuals who are, or may be, experiencing substance use disorders and/or other mental health disorders, mental health crisis and suicidal distress, and their families and carers. Services will be delivered in line with guidance from the Mental Health Alcohol and Other Drugs Branch:

2.9 Oral health services

The HHS will provide oral health services to people who meet the eligibility criteria for accessing public dental services in Queensland. Services will be delivered in line with guidance from the Office of the Chief Dental Officer.

2.10 Prisoner health services

This clause does not apply to this HHS.

2.11 Youth detention services

This clause does not apply to this HHS.

2.12 Refugee health

This clause does not apply to this HHS.

2.13 State-funded outreach services

(a) Where state-funded outreach services are provided;

(i) funding for the service will remain part of the providing HHS's funding allocation; and

(ii) the activity must be recorded at the HHS where the outreach service is being provided

(b) Any changes to the provision of outreach services will follow the requirements set out in clause 3 of this service agreement.

3. Teaching training and research

The HHS will provide the teaching, training and research programs for which funding is provided within Schedule 2 and as described below.

3.1 Clinical education and training

(a) The HHS will provide education and training placements for the following professional groups consistent with and proportionate to the capacity of the HHS and will support and align with stipulated placement terms governing clinical placements in Queensland Health facilities;

(i) medical students;

(ii) nursing and midwifery students;

(iii) pre-entry clinical allied health students;

(iv) interns;

(v) rural generalist trainees;

(vi) vocational medical trainees;

(vii) first year nurses and midwives;

(viii) re-entry to professional register nursing and midwifery candidates;

(ix) dental students;

(x) allied health rural generalist training positions;

(xi) Aboriginal and Torres Strait Islander Health Workers and Aboriginal and Torres Strait Islander Health Practitioners.

(b) The HHS will comply with the state-wide vocational medical training pathways.

(c) The HHS will support profession specific and inter-profession statewide allied health clinical education programs.

(d) The HHS will continue to implement and retain the following positions provided through clauses to the *Health Practitioners and Dental Officers (Queensland Health) Certified Agreement (No 3)*:

(i) health practitioner research positions provided through the Research Package for Health Practitioners; and

(ii) clinical educator positions provided through the Clinical Education Management Initiative for Health Practitioners.

(e) The HHS will maintain or increase its contribution of staff to the Queensland Country Relieving doctors program and the receiving HHS will be responsible for wages, clinical governance and appropriate supervision of the junior medical relievers.

3.2 Statewide training, education and research

This clause does not apply to this HHS.

3.3 **Health and medical research**

The HHS will:

(a) develop and implement a strategy to drive increased research activity and its translation into clinical practice;

(b) support increased and equitable access to clinical trials for patients;

(c) support clinicians to undertake research linked to their practice; and

(d) ensure high quality and timely research governance approval processes.

4. Delivery of purchased activity

4.1 The Department and the HHS will monitor actual activity against purchased levels and will take action as necessary to ensure delivery of purchased levels is achieved. The HHS has a responsibility to only recognise revenue that is linked with actual activity delivered against purchased volumes.

4.2 The HHS will actively monitor variances from purchased activity levels and notify the Department as soon as the HHS becomes aware of significant variances.

4.3 The HHS will also notify the Department of deliberate changes to the consistent recording of activity within year that would result in additional activity being recorded for existing health services.

4.4 If the HHS wishes to convert activity between purchased activity types, programs and levels the HHS must negotiate this with the Department based on a sound needs based rationale.

4.5 The Department will reconcile in-scope activity, as defined in the Activity Reconciliation specification sheet (available online, as detailed in Appendix 1), delivered by the HHS against the purchased in-scope activity targets outlined in Table 4.

4.6 Activity reconciliation will be undertaken in February (for the July to December period) and August (for the January to June period) each year and will be derived through application of the methodology which is documented in the Activity Reconciliation specification sheet.

4.7 Should the HHS be unable to deliver the activity that has been funded a financial adjustment will be applied.

4.8 Under delivery of in-scope activity, as defined in the Activity Reconciliation specification sheet, will be withdrawn from the HHS at 100% of the Queensland Efficient Price (QEP).

4.9 Funding and corresponding activity that is withdrawn from the HHS may be reallocated to an alternate provider that can undertake the activity.

4.10 Any resulting changes to funding and purchased activity levels will be actioned through the processes outlined in Schedules 2 and 4 of this service agreement.

4.11 The HHS will maintain its efforts to accurately record the delivered activity and submit this data to the Department in accordance with the agreed requirements.

5. Delivery of purchased services

5.1 As part of the service agreement, the Department purchases a range of services (or deliverables) from the HHS for the delivery of certain programs and projects.

5.2 These program or project services may be the subject of separately agreed conditions tied to that funding and the focus of detailed monitoring by the Department.

5.3 Conditions may include, but are not limited to:

(a) establishment and/or commencement of services;

(b) delivery of activity;

(c) workforce obligations;

(d) establishment of oversight committees;

(e) opening or upgrades to facilities;

(f) program evaluation;

(g) program management;

(h) reporting or notification obligations; and

(i) attainment of performance standards.

5.4 The HHS will ensure that the conditions are achieved within the stipulated time period.

5.5 The HHS will notify the Department if the HHS forecasts an inability to achieve program or project objectives or the conditions.

5.6 The Department may withdraw allocated funding pro rata to the level of under delivery if the services for a specified program or project are not being fully delivered according to the objectives or conditions.

5.7 Any resulting changes to funding and purchased activity levels will be actioned through the processes outlined in Schedules 2 and 4 of this service agreement.

6. Financial adjustments

6.1 **Activity targets**

(a) The Department will initiate a joint process with the HHS to determine whether a financial adjustment should be applied in relation to any purchased activity which has breached the thresholds identified bi-annually. This process will take into account any relevant matters that have been identified in a review/analysis of the breach as well as the outcomes of the activity plan implemented to address the activity breach.

(b) Activity will be monitored at the purchasing hierarchy level. Providing the HHS meets all relevant KPIs and specific funding allocations, the HHS has the ability to negotiate the transfer of activity across the purchasing hierarchy with the Department.

(c) Table 2 demonstrates the financial adjustment that will be applied when activity thresholds have been breached.

(d) The HHS may not utilise the provisions within AASB15 *Revenue from Contracts with Customers* to override the application of any financial adjustment made by the Department in line with Table 2.

Table 2 Financial adjustments applied on breach of activity thresholds

Example of Breach	Description	Financial Adjustment
Over performance	Activity exceeds that specified in the service agreement value for in-scope activity as shown in Table 4.	Purchasing contracts are capped and an HHS will not be paid for additional activity with the exception of activity that is in scope for the identified purchasing incentives as set out in Table 3.
Under performance	Activity is below that specified for in-scope activity as shown in Table 4.	Purchased activity and the related funding will be withdrawn at 100% of the Queensland Efficient Price and reallocated to an alternate provider that can undertake the activity.<br>Refer to Table 4 for the HHS QWAU target.
Failure to deliver on service commitments linked to specific funding allocations	Specific funding allocations<br>National Partnership Agreements.	It is at the discretion of the Department to withdraw allocated funding pro rata to the level of under delivery.
For all other types of activity variance, any financial adjustment will be made at the discretion of the Department.		

6.2 National Partnership on COVID-19 Response

(a) The Department will provide additional funding to the HHS under the *National Partnership on COVID-19 Response* where actual additional cost is demonstrated.

(b) Funding will be provided where the HHS has:

(i) undertaken activity that is in-scope for the State Public Health Payment, as defined under the *National Partnership on COVID-19 Response*, during the reporting period*;* and/or

(ii) undertaken activity that is in-scope for the Hospital Services Payment, as defined under the *National Partnership on COVID-19 Response*, during the reporting period; and

(iii) met the reporting requirements in relation to COVID-19 expenditure, as prescribed under the *National Partnership on COVID-19 Response* and by the Department.

(c) Additional costs that are reimbursed through the State Public Health Payment and the Hospital Services Payment will be excluded from the calculation of activity eligible for funding under the terms of the *National Health Reform Agreement*.

(d) There is no guarantee that the HHS will have access to additional funding under the State Public Health Payment or the Hospital Services Payment.

(e) All funding that is provided through the State Public Health Payment and the Hospital Services Payment will be subject to a reconciliation to be undertaken by the Administrator of the National Health Funding Pool. The HHS will be required to provide evidence to support claims for additional costs. If the HHS is unable to provide the required evidence with their expenditure claim, funding received may be recalled subject to reconciliation.

(f) Funding adjustments will be actioned through the processes outlined in Schedules 2 and 4 of this service agreement.

6.3 Purchasing incentives

(a) The purchasing approach includes a range of funding adjustments which aim to incentivise high quality and high priority activity, support innovation and evidence-based practice, deliver additional capacity through clinically and cost effective models of care and dis-incentivise care which provides insufficient or no benefit for patients. This includes incentive payments for HHSs who achieve targets in specific priority areas. The purchasing incentives are detailed in Table 3. The Department must reconcile the applicable purchasing incentives in Table 3 in line with the timeframes specified in the relevant purchasing specification sheet. The Department must provide a copy of the reconciliation statement to the HHS.

(b) Funding adjustments must be based on the requirements contained in the relevant purchasing specification sheet for that purchasing incentive.

(c) Any resulting changes to funding and purchased activity levels will be actioned through the processes outlined in Schedules 2 and 4 of this service agreement.

Table 3 Purchasing Incentives 2022/23

Incentive	
**Quality Improvement Payment (QIP)**	
Antenatal care for First Nations Women	Payments for achieving two Closing the Gap targets for First Nations women:<br>• to attend five or more antenatal visits with their first antenatal first taking place in the first trimester; and<br>• to stop smoking by 20 weeks gestation.
**Purchasing incentives**	
Virtual care incentive	Incentive funding to increase the number of specialist outpatient services which are provided in virtual settings.
Own source revenue growth	Incentivise the recognition of own source revenue through matching growth in own source revenue with public activity growth funding.
**ABF model localisations**	
Sentinel events	Payment withdrawn for sentinel events as per the national ABF model.
Advance Care Planning (ACP)	QWAUs for HHSs who offer ACP discussions to admitted patients, non-admitted outpatients, community health patients and Emergency Department patients.
Emergency Department Did Not Wait (DNW)	Zero QWAUs for DNWs.
Fractured neck of femur	QWAUs reduced by 20% for non-timely surgical treatment of fractured neck of femur.
Hospital in The Home (HITH)	QWAUs increased for Hospital in the Home (HITH) admissions of Residential Aged Care Facility residents.
Out-of-scope services	Nil QWAUs for out-of-scope procedures.
Pre-operative bed days	QWAUs for long stay days above the upper trim point less ICU days reduced equivalent to pre-operative days, up to a maximum of 3 days, for elective episodes.
Smoking cessation (community mental health)	QWAUs for smoking cessation activity for community mental health patients.

Incentive	
Smoking cessation (inpatients)	QWAUs for smoking cessation activity for publicly funded inpatients.
Stroke care	10% QWAU loading for acute stroke patients admitted to Statewide Stroke Clinical Network-endorsed stroke unit care.
Telehealth (admitted patients)	QWAUs for provider-end of in-scope admitted patient telehealth activity.
**Commissioning mechanisms**	
High-cost home support	Funding for approved individuals requiring 24-hour home ventilation.
Patient flow initiative	Provision of non-recurrent WAU-backed funding to participating HHS who successfully implement agreed recommendations.
Rapid access clinics	Recurrent WAU-backed funding to support the implementation of rapid access clinics to reduce pressure on emergency departments.
Expansion of sub-acute and long stay care	Additional funding to increase the availability of and access to care for sub-acute and long stay patients, thereby improving access to care in a range of settings and releasing capacity within acute facilities.
Connected Community Pathways	Funding to incentivise evidence-based and innovative models of care which promote the delivery of care outside acute facilities and support shared-care partnership arrangements.

6.4 Surgery Connect reimbursements

(a) The HHS will reimburse the Department for the actual costs of activity for nominated patient referrals to the Surgery Connect program where:

(i) The HHS has nominated the patient referral as HHS funded on entry of the referral in the Surgery Connect Activity Navigator (SCAN); and

(ii) The HHS Chief Finance Officer has recorded approval of the nomination in SCAN;

or

(iii) The HHS has obtained the Department's agreement to retrospectively convert a defined patient cohort to HHS funded status in SCAN.

(b) The HHS may only request retrospective conversion of activity to HHS funded within the financial year in which the activity has taken place.

(c) Any resulting changes to funding and purchased activity levels will be actioned through the processes outlined in Schedules 2 and 4 of this service agreement.

6.5 Financial adjustments – other

(a) Notwithstanding the provisions regarding the recognition of revenue as stipulated in AASB1058 *Income of Not-for-Profit Entities* and/or AASB15 *Revenue from Contracts with Customers*, the Department may seek to recover funding from an HHS that was provided through this service agreement which has:

(i) not been utilised in accordance with its intended purpose; and/or

(ii) not been utilised within the prescribed time period to deliver the agreed

outcomes/services.

(b) If the Chief Executive (or delegate) determines that previously allocated funding is to be recovered, any resulting changes to funding and purchased activity levels will be actioned through the processes outlined in Schedules 2 and 4 of this service agreement.

6.6 **Public and private activity/own source revenue**

(a) Own source revenue comprises grants and contributions, user charges and other revenues.

(b) Where an HHS is above its own source revenue target, it will be able to retain the additional own source revenue with no compensating adjustments to funding from other sources where the additional revenue is not attributable to a private patient consistent with clauses A9 to A13 and A44 of the *National Health Reform Agreement.*

(c) Where an HHS is below its own source revenue target in respect of private patients, it will experience a reduction in revenue with no compensating adjustments to funding from other sources.

(d) The HHS will make all efforts to identify and maximise revenue from appropriate third parties to ensure optimisation of available State and Commonwealth funding across the Queensland public sector health system.

(e) The own source revenue identified in Table 4 is an estimate generated by the HHS which allows all third-party funding sources associated with service delivery to be identified. The HHS will ensure that this estimate is substantiated and accurate to ensure no significant variances to actual revenue generated.

(f) The HHS will routinely revise and update the estimate to ensure alignment between the service agreement and Queensland Treasury's reporting system (TRIDATA).

(g) Budget adjustments for changes in own source revenue from private patients will be actioned through the processes set out in Schedule 4 of this service agreement.

7. Funding sources

7.1 The four main funding sources contributing to the HHS service agreement value are:

(a) Commonwealth funding;

(b) State funding;

(c) grants and contributions; and

(d) own source revenue.

7.2 Table 4 provides a summary of the funding sources for the HHS and the total value of the service agreement.

8. Funds disbursement

8.1 The Department agrees to pay the HHS the amount described in Table 4 of Schedule 2, subject to:

(a) parliamentary appropriation and adequate funds being allocated to the Department; and

(b) the terms of this service agreement.

8.2 All payments under this service agreement will be made in accordance with the requirements of the *National Health Reform Agreement* and the Act.

8.3 The Chief Executive will direct the disbursement of both State and Commonwealth funding from the State Pool Account, the State Managed Fund and the Department of Health Expenditure account to the HHS.

8.4 However, the State (represented by the Chief Executive) will not:

(a) redirect Commonwealth payments between HHSs;

(b) redirect Commonwealth payments between funding streams (e.g., from ABF to block funding); and/or

(c) adjust the payment calculations underpinning the Commonwealth's funding.

8.5 The Department will pay state-funded activity-based funding, block funding and system manager funding to the HHS on a fortnightly basis in line with receipt of the State Appropriation Payment. The payment made will be equal to 1/26th of the state-funded component of the service agreement value described in Table 4.

8.6 The Department will pay Commonwealth-funded activity-based funding and block funding on a monthly basis in line with receipt of the Commonwealth payment. The payment made will be equal to 1/12th of the Commonwealth funded component of the service agreement value described in Table 4.

8.7 Where the parties have agreed to amend the service agreement value or the Commonwealth contribution amount changes, the fortnightly and/or monthly payments will be adjusted to reflect the amended service agreement value.

8.8 Service agreement payments may be made outside of these timeframes with the approval of the Department's Chief Finance Officer.

Table 4 Torres and Cape HHS Total Funding Allocation by Funding Source 2022/23

Funding Source	NWAU (N2122)	QWAU (Q24)	Value ($)
**NHRA Funding**			
**ABF Pool**			
ABF Funding (in scope NHRA)[2]			
Commonwealth[2]	0		$0
State		0	$0
State Specified Grants			$0
State-wide Services			$0
**State Managed Fund**			
Block Funding			
Small Rural Hospitals		11,646	$75,902,142
Teaching, Training & Research			$40,000
Non-Admitted Child & Youth Mental Health			$2,254,280
Non-Admitted Home Ventilation			$0
Non-Admitted Mental Health			$5,038,032
Other Non-Admitted Service			$0
Highly Specialised Therapies			$0
**Total NHRA Funding**	**-**	**11,646**	**$83,234,455**
**Out of Scope NHRA**			
**Queensland ABF Model**			
DVA		25	$85,520
NIISQ/MAIC		0	$0
Oral Health		883	$4,591,335
BreastScreen		0	$0
**Total Queensland ABF Funding**	**-**	**908**	**$4,676,855**
**Discretely Funded Programs[3]**			
Department of Health			$141,820,472
Locally receipted funds			$1,412,878
**Total Discretely Funded Programs**	**-**	**-**	**$143,233,350**
**Own Source Revenue**			
Private Patient Admitted Revenue[4]	*16*	18	$90,568
Non-Admitted Services		318	$16,702
Pharmaceuticals Benefits Scheme		266	$671,332
Other Activities[5]		25	$5,930,095
**Total Own Source Revenue**	**-**	**628**	**$6,708,697**

[2] The split between Commonwealth and State NHRA funding will change during the year as the purchased activity targets are updated and the National Health Funding Board updates their payment advice accordingly.

[3] Includes all other (non-ABF) State and Commonwealth funded health services including, but not limited to, Prisoner Health, Public Health and Prevention Services.

[4] The estimated value of the revenue earned from private patients, based on OSR estimates provided by the HHS.

[5] Incorporates all OSR which is not identified elsewhere in Table 4.

Funding Source	NWAU (N2122)	QWAU (Q24)	Value ($)
Locally Receipted Funds (exc. Discretely Funded Programs)[6]			$18,762,321
Depreciation			$20,273,000
**NPA COVID-19 Response**			
Hospital Services Payment			$0
State Public Health Payment			$0
COVID-19 Vaccine Payment			$0
**Total NPA COVID-19 Response Funding**	**-**	**-**	**$0**
**GRAND TOTAL**	**0**	**13,182**	**$276,888,677**

Pool Accounts			
ABF Pool (National Health Funding Pool)[7]			$0
State Managed Fund[8]			$83,234,455
System Manager			$146,497,326

[6] Includes items such as training programs and donations. Does not include locally receipted funds associated with discretely funded programs, e.g.Transition Care.

[7] Articulates the financial payment made to support in-scope ABF services under the NHRA including DVA, NIISQ, MAIC and Breastscreen Services. Applies to all HHSs except Central West HHS and Torres and Cape HHS.

[8] Articulates the payment made for block funded services under the NHRA, DVA, NIISQ and MAIC services.

Table 5 National Health Reform Funding

NHRA Funding Type	NWAU (N2122)	Commonwealth ($)	State ($)	Other State funding[9] DVA/MAIC/Oral Health/BreastScreen ($)	Total ($)
**National Efficient Price (NEP)**					**$5,597**
**ABF Allocation (NWAU)**					
Emergency Department	0	$0	$0	$0	$0
Acute Admitted	0	$0	$0	$0	$0
Admitted Mental Health	0	$0	$0	$0	$0
Sub-Acute	0	$0	$0	$0	$0
Non-Admitted	0	$0	$0	$0	$0
**Total ABF Pool Allocation**	**0**	**$0**	**$0**	**$0**	**$0**
**Block Allocation**					
Teaching Training and Research	-	$8,248	$31,752	-	$40,000
Small and Rural Hospitals[10]	-	$27,385,735	$48,516,407	-	$75,902,142
Non-Admitted Mental Health	-	$1,918,489	$3,119,544	-	$5,038,032
Non-Admitted Child & Youth Mental Health	-	$229,965	$2,024,316	-	$2,254,280
Non-Admitted Home Ventilation	-	$0	$0	-	$0
Other Non-Admitted Services	-	$0	$0	-	$0
Other Public Hospital Programs	-	$0	$0	-	$0
Highly Specialised Therapies	-	$0	$0	-	$0
**Total Block Allocation**	**-**	**$29,542,436**	**$53,692,019**	**-**	**$83,234,455**
**Grand Total Funding Allocation**					**$83,234,455**

[9] State funding transacted through the Pool Account; not covered under the NHRA

[10] Incorporating small regional and rural public hospitals, five specialist mental health facilities (Baillie Henderson Hospital, Jacaranda Place – Queensland Adolescent Extended Treatment Centre, The Park – Centre for Mental Health, Kirwan Rehabilitation Unit and Charters Towers Rehabilitation Unit) and the Ellen Barron Family Centre.

Table 6 Discretely Funded Programs (Non-ABF)

Discretely Funded Programs	$
Aged Care Assessment Program	$0
Alcohol, Tobacco and Other Drugs	$3,883,058
Community Health Programs	$26,002,727
Disability Residential Aged Care Services	$0
Home and Community Care Program (HACC)	$1,412,878
Interstate Patients (QLD residents)	$329,859
Multi-purpose Health Services	$2,869,418
Other State Funding	$92,876,945
Patient transport	$12,962,085
Prevention Services and Public Health	$2,896,381
Prisoner Health Services	$0
Research	$0
Transition Care	$0
Residential Aged Care Services	$0
**TOTAL**	**$143,233,350**

Schedule 3 Performance Measures

1. Performance Measures

1.1 The performance of the HHS will be measured according to the assessment criteria and processes described in the Performance and Accountability Framework.

1.2 The detailed specification for each of the performance measures listed in this service agreement are provided through performance measure attribute sheets.

1.3 The performance measures identified in this service agreement are applicable to the HHS unless otherwise specified within the attribute sheet.

1.4 HHSs are also required to report against the agreed key performance measures in their Health Equity Strategy.

Table 7 HHS Performance Measures – Key Performance Indicators

Key Performance Indicators
Hospital Acquired Complications
Elective surgery patients waiting longer than the clinically recommended timeframe
Gastrointestinal endoscopy patients waiting longer than the clinically recommended timeframe
Access to oral health services (adults)
Access to oral health services (children)
The percentage of oral health activity which is preventive
Proportion of diabetic patients with a current HbA1c result
Patients whose smoking status has been recorded
Potentially Preventable Hospitalisations – First Nations peoples:<br>• Diabetes complications<br>• Selected conditions
Potentially Preventable Hospitalisations (non-diabetes complications)
Telehealth utilisation rates:<br>• Number of non-admitted telehealth service events
% of low birthweight babies born to Queensland mothers
Forecast operating position:<br>• Full year<br>• Year to date
Average sustainable Queensland Health FTE
Capital expenditure performance
Care Closer to Home:<br>• *Self-sufficiency: admitted patients*<br>• *Self-sufficiency: non-admitted patients*
Participation in the Practice Incentive Program
Proportion of mental health and alcohol and other drug service episodes with a documented care plan

Table 8 HHS Performance Measures - Safety and Quality Markers

Safety and Quality Markers
Sentinel Events
Hospital Standardised Mortality Ratio
Severity Assessment Code (SAC) analysis completion rates
Patient Reported Experience

Table 9 HHS Performance Measures – Outcome Indicators

Outcome Indicators
Access to emergency dental care
First Nations peoples representation in the workforce
General oral health care for First Nations peoples
Complaints resolved within 35 calendar days
Advance care planning
Smoking cessation clinical pathway
Adolescent vaccinations administered via the statewide School Immunisation Program
Integrated care pathways:<br>• Care pathway in place for patients with identified co-morbidities

Schedule 4 Amendments to this service agreement

1. Agreed process to amend this service agreement

1.1 The parties acknowledge that this service agreement is subject to amendment, which will generally occur through:

(a) amendment windows;

(b) extraordinary amendment;

(c) periodic adjustments; and

(d) end of financial year reconciliation.

1.2 **Amendment windows**

(a) There will be set periods of time nominated by the Department during the year (amendment windows) in which a party may propose an amendment and the parties will endeavour to negotiate and finalise proposals to amend this service agreement.

(b) Amendment proposals that are agreed will be documented in a deed of amendment to this service agreement.

(c) Further details on the amendment window process, including the timing of amendment windows, is provided online, as detailed in Appendix 1.

1.3 **Extraordinary Amendment**

(a) Outside an amendment window, the Department and the HHS agree to limit any proposal to amend the terms of this service agreement to those where there is an urgent priority need to facilitate a funding allocation (extraordinary amendment). The parties will endeavour to negotiate and finalise any such proposal urgently.

(b) The process for submitting, negotiating and resolving an extraordinary amendment is available online, as detailed at Appendix 1.

(c) Agreed extraordinary amendments will be reflected in a notice issued by the Chief Executive countersigned as accepted by the HHS, which notice will be replaced when the extraordinary amendment is subsequently formalised in a deed of amendment issued following the next amendment window.

1.4 **Periodic adjustments**

(a) The service agreement value (and corresponding purchased activity) may be adjusted at any time to reflect funding variations that:

(i) occur on a periodic basis or in line with adjustments permitted for specific funding allocations;

(ii) are referenced in the service agreement; and

(iii) are based on a clearly articulated formula, an agreed basis or such other reasonably substantiated basis tied to performance.

(periodic adjustment).

(b) Periodic adjustments will be reflected in an adjustment notice issued by the Chief Executive (or delegate) to the HHS, based on relevant data, and subsequently formalised in a deed of amendment issued following the next amendment window.

1.5 End of financial year reconciliation

(a) There will be an end of financial year reconciliation process, with the scope defined by the Department and informed by Queensland Government Central Agency requirements.

(b) The Department will provide the HHS with a reconciliation of all service agreement funding and purchased activity for the prior financial year. This will reflect the position following conclusion of the end of financial year adjustments process.

(c) The impact of end of financial year adjustments on subsequent year funding and activity will be incorporated in the service agreement through the deed of amendment executed following the next available amendment window.

(d) This clause will survive expiration of this service agreement.

Appendix 1 Reference Documents

Hospital and Health Boards Act 2011

National Health Reform Agreement (NHRA) 2020-25

System Outlook to 2026 - for a sustainable health service

Queensland Health Performance and Accountability Framework

My health, Queensland's future: Advancing health 2026

Department of Health Strategic Plan 2021-2025

Local Area Needs Assessment (LANA) Framework

Making Tracks Together: Queensland's Aboriginal and Torres Strait Islander Health Equity Framework

Making Tracks toward closing the gap in health outcomes for Indigenous Queenslanders by 2033 - Policy and Accountability Framework

Aboriginal and Torres Strait Islander Health Workforce Strategic Framework 2016-2026

National Agreement on Closing the Gap

Queensland Health Workforce Diversity and Inclusion Strategy 2017 to 2022

Performance Measures Attribute Sheets

Purchasing Initiatives and Funding Specifications

Public Health Practice Manual

National Partnership on COVID-19 Response

Statewide services reference material

Service agreement amendment processes

Data supply requirements

Contracting and Performance Management Branch
GPO Box 48
Brisbane QLD 4001 Australia

Tel (+61) (07) 3708 5869

www.health.qld.gov.au

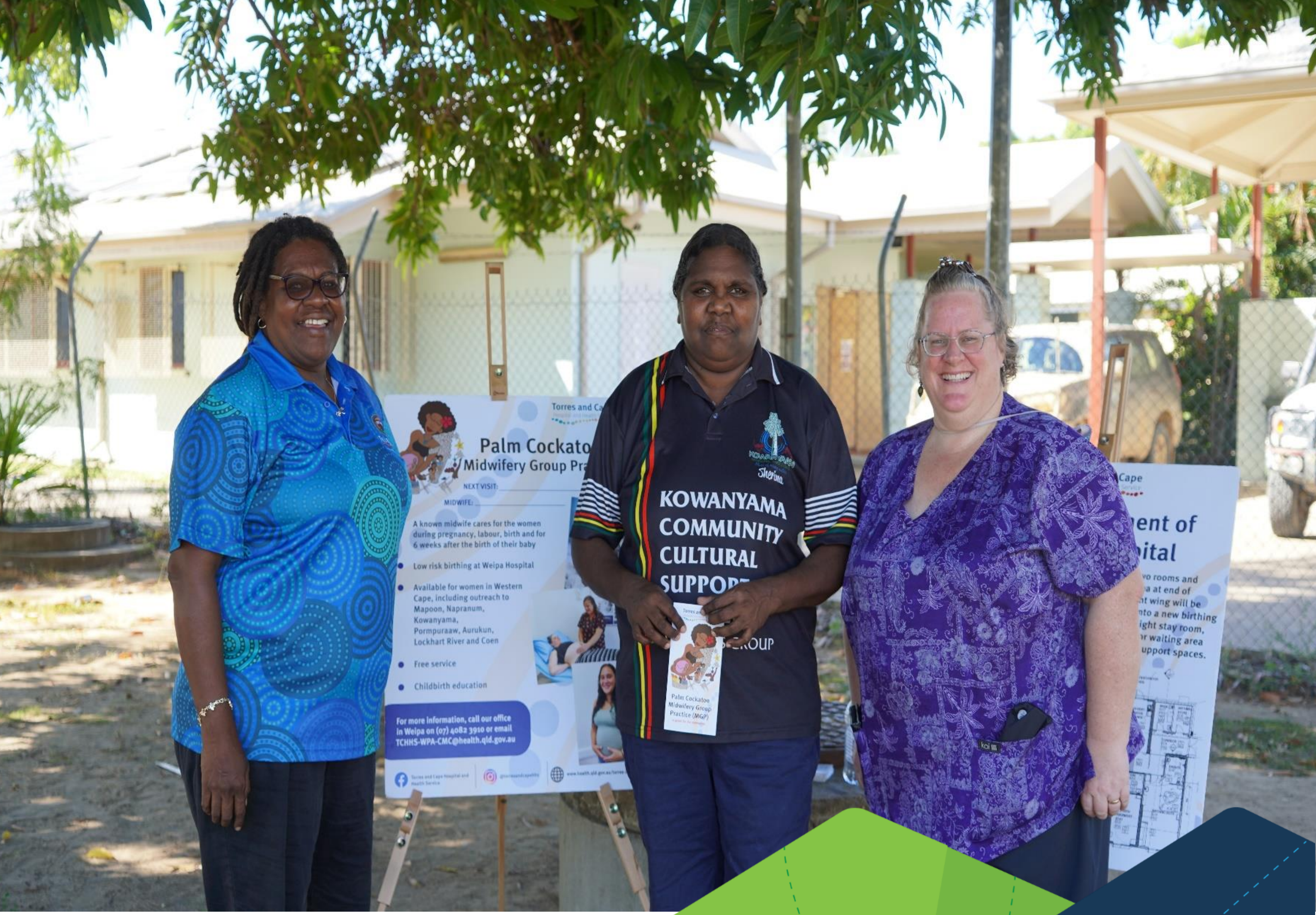

Queensland Health

Service Agreement 2022/23 – 2024/25

Torres and Cape Hospital and Health Service

December 2024 Revision

Queensland Government

Torres and Cape Hospital and Health Service, Service Agreement 2022/23 - 2024/25

Published by the State of Queensland, (Queensland Health), December 2024

This document is licensed under a Creative Commons Attribution 3.0 Australia licence. To view a copy of this licence, visit creativecommons.org/licenses/by/3.0/au

© State of Queensland (Queensland Health) **2024**

You are free to copy, communicate and adapt the work, as long as you attribute the State of Queensland (Queensland Health).

For more information contact:

Contracting and Performance Management Branch, Department of Health, GPO Box 48, Brisbane QLD 4001
Tel (+61) (07) 3708 5869

Email SAM@health.qld.gov.au

An electronic version of this document is available at www.health.qld.gov.au/system-governance/health-system/managing/default.asp

Disclaimer:
The content presented in this publication is distributed by the Queensland Government as an information source only. The State of Queensland makes no statements, representations or warranties about the accuracy, completeness or reliability of any information contained in this publication. The State of Queensland disclaims all responsibility and all liability (including without limitation for liability in negligence) for all expenses, losses, damages and costs you might incur as a result of the information being inaccurate or incomplete in any way, and for any reason reliance was placed on such information.

Acknowledgement

Torres and Cape Hospital and Health Service acknowledges and respects the Traditional Owners of the land on which we live and work and acknowledges their continuing connection to the land and community which we serve. We pay respect to them, their culture, and their Elders past, present and future.

We recognise the First Nations peoples in Queensland are both Aboriginal Peoples and Torres Strait Islander Peoples, and support the cultural knowledge, determination, and commitment of Aboriginal and Torres Strait Islander communities in caring for health and wellbeing for millennia.

Torres and Cape Hospital and Health Service is proud to recognise and celebrate the cultural diversity of our communities and workforce and we acknowledge the following Traditional Owners and Custodians of the lands within the Torres and Cape:

- Ayabadhu
- Alngith
- Anathangayth
- Anggamudi
- Apalech
- Atambaya
- Angkamuthi
- Binthi
- Burunga
- Dingaal
- Girramay
- Gulaal
- Gugu Muminh
- Guugu-Yimidhirr
- Gudang
- Gudamaluilgal
- Kaantju
- Koko-bera
- Kokomini
- Kuku Taypan
- Kuku Yalanji
- Kunjen/Olkol
- Kuuku – Yani
- Kaiwalagal
- Kulkalgal
- Wik
- Wik Mungkan
- Wimarangga
- Yadhaykenu
- Lama Lama
- Mpalitjanh
- Munghan
- Maluilgal
- Meriam
- Ngaatha
- Ngayimburr
- Ngurrumungu
- Nugal
- Oolkoloo
- Oompala
- Peppan
- Puutch
- Sara
- Teppathiggi
- Thaayorre
- Thanakwithi
- Thiitharr
- Thuubi
- Tjungundji
- Uutaalnganu
- Wanam
- Warrangku
- Wathayn
- Waya
- Winchanam
- Wuthathi
- Yupungathi

Contents

Acknowledgement 3
Contents 4
Tables 5
1. Introduction 6
2. Scope 6
3. Services 6
4. Performance and Accountability Framework 7
5. Outcomes Framework 7
6. Data supply requirements 8
7. Hospital and Health Service accountabilities 8
8. Department accountabilities 10
9. Achieving health equity with First Nations Queenslanders 10
10. Dispute Resolution 11
11. General 11
12. Counterparts 12
Execution 13
Schedule 1 HHS profile 14
1. HHS profile 14
Schedule 2 Funding and purchased activity and services 15
1. Introduction 15
2. Purchased health services 16
3. Teaching, training, and research 18
4. Delivery of purchased activity 19
5. Delivery of purchased services 20
6. Financial adjustments 21
7. Funding sources 25
8. Funds disbursement 25
Schedule 3 Department of Health Provided Services 32
1. In scope services and service schedules 32
Schedule 4 Performance Measures 33
1. Performance Measures 33
Schedule 5 Amendments to this service agreement 37
1. Agreed process to amend this service agreement 37
Appendix 1 Reference Documents 39

Tables

Table 1 Statewide Services 16
Table 2 Financial adjustments applied on breach of activity thresholds 22
Table 3 Funding adjustments 2024/25 22
Table 4 HHS Funding by Outcomes Framework Care Domain 30
Table 5 HHS Total Funding Allocation by Funding Source 2024/25 30
Table 6 National Health Reform Funding 29
Table 7 Discretely Funded Programs (Non-ABF) 31
Table 8 Department of Health Provided Services and Service Schedules 34
Table 9 HHS Performance Measures – Key Performance Indicators 35
Table 10 HHS Performance Measures – Safety and Quality Markers 34
Table 11 HHS Performance Measures – Outcome Indicators 35
Table 12 Statewide Health Equity Key Performance Measures 35

1. Introduction

1.1 In performing this service agreement, the Department and the Hospital and Health Service (HHS) will act consistent with the object of the *Hospital and Health Boards Act 2011* (Qld) (Act).

1.2 The Department and the HHS operate as part of a networked system and agree to work collaboratively with each other, with other HHSs and with the Queensland Ambulance Service in the best interests of the Queensland public sector health system.

1.3 The parties will ensure that planning and delivery of health services is consistent with the strategies and priorities set out by government. The parties recognise the importance of the HHS' Health Equity Strategy (as defined in the Act) and the parties' commitment to improving health and wellbeing outcomes and achieving health equity for Aboriginal and Torres Strait Islander peoples.

2. Scope

2.1 This service agreement covers the period from 1 July 2022 to 30 June 2025.

2.2 The parties have identified the services to be provided by the HHS, the funding for the provision of those services, the performance measures applicable to the services and data requirements.

3. Services

3.1 In delivering services, the HHS is required to meet:

(a) the applicable conditions of all national agreements and national partnership agreements between the Queensland Government and the Commonwealth Government and commitments under any related implementation plans; and

(b) the applicable conditions of each agreement or arrangement for funding between the Department and the HHS and commitments under any related implementation plans, which conditions and commitments may be recorded separately to this service agreement but for which funding is (or becomes) provided in Schedule 2.

3.2 The HHS is required to deliver the services outlined in this service agreement for which funding is provided in Schedule 2.

3.3 Where issues arise which prevent the HHS from providing a service or necessitate a reduction in the level or scope of a service provided, prompt notification must be made to the Department and impacted HHSs, with appropriate details. The HHS must minimise any clinical risk or adverse impact to patient experience that may result from service disruption. The Department will respond to the HHS on any requirements it has concerning service delivery and any adjustments triggered by under delivery.

3.4 If the HHS wishes to terminate or reduce service levels for a service for which funding is provided in Schedule 2, this will remain subject to negotiation and agreement by the Department at its discretion.

3.5 For any new services proposed during the term of this service agreement, the parties agree:

(a) the service must meet a demonstrated clinical need and provide value for money;

(b) the commencement of a new service, including the implementation of new models of care, may occur where the service has been commissioned by the Department, a funding stream is in place and any conditions relating to the funding have already been agreed; and

(c) if the HHS wishes to commence providing a new service that has not been commissioned by the Department, this will remain subject to agreement by the Department at its discretion.

3.6 It is acknowledged that there may, from time to time, need to be service delivery changes between HHSs. Management of inter-HHS relationships should be informed by the following principles:

(a) HHSs should maintain the proportion of out of HHS work undertaken unless as a result of agreed repatriation of patients;

(b) each HHS should manage patients from its own catchment population if it is within its clinical capability to do so as specified by the Clinical Services Capability Framework; and

(c) where it is proposed that a service move from one HHS to another, the Department will consider, as part of its review under clauses 3.4 and 3.5, whether the respective Health Service Chief Executives endorse the proposed change in patient flows; and the funding required to follow the patient.

4. Performance and Accountability Framework

4.1 The Queensland Health Performance and Accountability Framework (the Performance and Accountability Framework) sets out the framework within which the Department monitors and manages the performance of public sector health services in Queensland.

4.2 The parties will act consistent with the Performance and Accountability Framework.

5. Outcomes Framework

5.1 Queensland Health is embarking on a strategic shift in funding focus from “volume” to “outcome” using the Outcomes Framework. This approach aims to link the resources and services required and delivered as part of healthcare activities, to health outcomes for individuals and the population.

5.2 The Outcomes Framework takes a three-tiered approach:

(a) The System Tier (Tier 1), which acts as a strategic tier, and includes four domains to measure the contribution of Queensland Health to the system outcomes.

(b) The Operational Tier (Tier 2) which includes nine (9) Clinical Care Domains, reflecting areas that are important to deliver change and improvement in the short to medium term, and to operationalise the Outcomes Framework.

(c) The Tactical Tier (Tier 3) provides scaffolding to select initiatives for implementation as specific pressures arise. These pressures may include areas identified for improvement through Tier 2.

5.3 In consultation with the State-wide Clinical Networks the indicators below are under further development and shadowing.

Indicator	Care Domain	Clinical Leadership
Percentage of patients who have HBA1C ordered during hospital admission	Chronic and Complex	Diabetes Network
Time to treatment for breast, colorectal and lung cancers	Cancer Care	Cancer Care Network

5.4 Schedule 4 maps existing indicators in the Performance and Accountability Framework to the care domains of the Outcomes Framework.

6. Data supply requirements

6.1 The HHS will provide the Department with all clinical and non-clinical data that is reasonably required to support the effective management of the public sector health system. This will include, but is not limited to, data that is required to:

(a) fulfil legislative obligations;

(b) deliver accountabilities and obligations to State and Commonwealth Governments; including related to the provision and reconciliation of activity data by the Administrator of the National Health Funding Pool;

(c) monitor and support performance improvement;

(d) manage this service agreement;

(e) support clinical innovation; and

(f) facilitate evaluation and audit.

6.2 The parties agree and acknowledge that:

(a) the Department will keep the HHS informed of the Department's data requirements; and

(b) data will be provided as required, or permitted, by law.

6.3 Further details on data supply requirements, including principles that guide the collection, storage, transfer and disposal of data and prescribed timeframes for data submission, are provided online as detailed in Appendix 1.

7. Hospital and Health Service accountabilities

7.1 The HHS will perform its obligations under this service agreement.

7.2 As applicable to the HHS and its services, the HHS will comply with:

(a) legislation and subordinate legislation, including the Act;

(b) cabinet decisions;

(c) Ministerial directives;

(d) agreements entered into between the Queensland and Commonwealth governments (or agreements with others in furtherance of such agreements), of which it is informed;

(e) agreements entered into between the Department and other Queensland Government entities, of which it is informed;

(f) agreements entered into with another HHS(s), including Networked Services Agreements;

(g) all industrial instruments;

(h) all health service directives and health employment directives; and

(i) all policies, guidelines, and implementation standards, including human resource policies.

7.3 As part of the commitment to achieving First Nations health equity, the HHS will prioritise the elimination of racial discrimination and institutional racism within its service and ensure that Aboriginal and Torres Strait Islander peoples have access to culturally safe and responsive health services.

7.4 The HHS will ensure that effective health service planning and delivery systems are in place, working in collaboration with the Department.

7.5 To support the achievement of the Queensland-Commonwealth Partnership's (QTP's) vision and commitment to work together to tackle health system challenges that cannot be overcome by any one organisation, HHSs are required to prepare and submit Joint Regional Needs Assessments in accordance with the framework provided online as detailed in Appendix 1.

7.6 HHSs must operate clinical service delivery consistent with the National Quality and Safety Standards. The HHS is expected to escalate any concerns that arise at the conclusion of a formalised assessment.

7.7 The HHS will ensure that health service employees employed by the Chief Executive[1] who perform work for the HHS are managed in accordance with any applicable delegations and directions from the Chief Executive. The HHS will ensure that effective asset management systems are in place (available online, as detailed in Appendix 1), that comply with the *Queensland Government Building Policy Framework and Guideline*, while working in collaboration with the Department.

7.8 The HHS will maintain accreditation to the standards required by the Department.

7.9 The HHS will appropriately perform and fulfil its functions under the Act.

[1] In this service agreement the term Chief Executive takes the meaning applied in the Act, which is the Chief Executive of Queensland Health, but which is generally referred to as the Director-General.

7.10 The HHS will provide to the Chief Executive reports of a type, and at the intervals, agreed between the parties, or as reasonably specified by the Chief Executive.

8. Department accountabilities

8.1 The Department will perform its obligations under this service agreement including, in return for the HHS performing its obligations and delivering the services, providing funding to the HHS as stipulated in this service agreement (as amended).

8.2 The Department will:

(a) comply with applicable legislation and subordinate legislation, including the Act, as relates to this service agreement;

(b) perform the system manager role (as defined in the Act) through the Chief Executive; and

(c) provide a range of services to the HHS as set out in Schedule 3.

8.3 The Chief Executive will appoint health service employees to perform work for the HHS for the purpose of enabling the HHS to perform its functions under the Act.

8.4 The Chief Executive will consult, cooperate, and coordinate with the HHS to ensure legal compliance with the *Work Health and Safety Act 2011* and other legislation as it applies to the scope, nature and location of operations associated with this service agreement.

8.5 The Chief Executive will appropriately perform and fulfil their functions under the Act.

9. Achieving health equity with First Nations Queenslanders

9.1 Through legislative amendments to the Act and the Hospital and Health Boards Regulation 2012, and the release of *Making Tracks Together: Queensland's Aboriginal and Torres Strait Islander Health Equity Framework* (2021), Queensland Health has strengthened its commitment to improving health and wellbeing outcomes and achieving health equity with First Nations peoples.

9.2 The HHS will develop and resource a First Nations Health Equity Strategy, compliant with legislative requirements. An implementation plan, accompanying the strategy, demonstrates the HHS's activities and key performance measures to achieve health equity with First Nations peoples. The Health Equity Strategy will act as the principal accountability mechanism between the Aboriginal and Torres Strait Islander community and the HHS in achieving health equity with First Nations Queenslanders and forms a key part of the Queensland Government's commitment to the *National Agreement on Closing the Gap* (2020).

9.3 The HHS is required to review the Health Equity Strategy at least once every three years and will publish the Health Equity Strategy in a way that allows it to be accessed by members of the public.

9.4 The HHS will ensure that commitment and leadership is demonstrated through implementing the actions and achieving the key performance measures outlined in the Health Equity Strategy.

9.5 The HHS will report publicly every year on progress against the Health Equity Strategy.

9.6 The HHS will support the implementation of other supplementary policies and strategies to drive health equity across the public health system, including relevant election commitments.

9.7 The HHS will participate as a partner in the implementation and achievement of Queensland's *HealthQ32 First Nations First Strategy 2032* in addition to HHS commitments within their Health Equity Strategy.

10. Dispute Resolution

10.1 Where a dispute arises in connection to this agreement, either between the department and one or more HHSs or between HHSs, every effort should be made to resolve the dispute at the local level. If local resolution cannot be achieved, the dispute resolution processes, accessible through Appendix 1, must be followed.

11. General

11.1 **Sub-contracting**

(a) The HHS must have appropriate systems in place to ensure that any subcontractor is accredited (as applicable), qualified, and otherwise fit to perform any services for which it is contracted.

(b) The HHS must ensure that any sub-contractor who has access to confidential information (as defined in the Act) or personal information (as defined in the Information Privacy Act 2009 (Qld)) complies with obligations no less onerous than those imposed on the HHS.

11.2 **Insurance**

The HHS must:

(a) hold and maintain the types and levels of insurances that the HHS considers appropriate according to its functions and obligations; and

(b) comply with reasonable requests or directions from the Department in this regard.

11.3 **Amendment**

The process for amending this service agreement is provided for under the Act and further outlined in Schedule 5.

12. Counterparts

12.1 This service agreement may be executed in two or more identical copy counterparts, each of which together will be deemed an original, but all of which together will constitute one and the same instrument.

12.2 In the event that any signature executing this service agreement or any part of this service agreement is delivered by facsimile transmission or by scanned e-mail delivery of a '.pdf' format data file or equivalent of the entire agreement, the signature will create a valid and binding obligation of the party executing (or on whose behalf the signature is executed) with the same force and effect as if the signature page were an original. For the avoidance of doubt, this service agreement may be in the form of an electronic document and may be electronically signed.

12.3 For execution under this clause 12 to be valid, the entire service agreement upon execution by each individual party must be delivered to the remaining parties.

Execution

The terms of this Service Agreement were agreed under the provisions set out in the *Hospital and* Health *Boards Act,* section 35 on 29 June 2022, and were subsequently amended by the Deeds of Amendment entered into pursuant to section 39 of the *Hospital and Health Boards Act 2011* and executed on 16 January 2023, 12 May 2023, 13 July 2023, 22 December 2023, 5 April 2024, 17 July 2024 and 18 December 2024.

This revised Service Agreement consolidates amendments arising from:

- Periodic Adjustment – COVID-19 Funding Transfer September 2022
- Periodic Adjustment – COVID-19 Funding Transfer October 2022
- 2022/23 Amendment Window 2 (in year variation)
- Periodic Adjustment – COVID-19 Funding Transfer December 2022
- 2022/23 Amendment Window 3 (in year variation)
- Periodic Adjustment – COVID-19 Funding Transfer April 2023
- Extraordinary Amendment Window – May 2023
- 2023/24 Amendment Window 1 (Budget Build)
- 2023/24 Amendment Window 2 (in year variation)
- 2023/24 Amendment Window 3 (in year variation)
- 2024/25 Amendment Window 1 (Budget Build)
- 2024/25 Amendment Window 2 (in year variation)

Schedule 1 HHS profile

1. HHS profile

Bordering with Papua New Guinea, Torres and Cape HHS provides public healthcare services to approximately 28,000 people spread widely across Cape York, the Northern Peninsula Area and the Torres Strait Islands. Sixty-seven percent of the population identify as Aboriginal and/or Torres Strait Islander (source: *Torres and Cape Hospital and Health Service Strategic Plan 2019 -2023 (version 2021)*

Services are provided from a wide range of locations, many with a primary care focus. The HHS also supports outreach teams and visiting specialist services from other HHSs along with non-government providers.

Close partnerships between the HHS and a range of local organisations are instrumental to supporting the shared goal of delivering safe, sustainable and innovative services closer to home and improving health outcomes in the region. The HHSs key local partners include:

- Apunipima Cape York Health Council
- NPA Family & Community Services (Aboriginal and Torres Strait Islander Corporation)
- Torres Health Indigenous Corporation
- Northern Queensland Primary Health Network
- Royal Flying Doctor Service
- CheckUp
- Northern Aboriginal and Torres Strait Islander Health Alliance (NATSIHA)
- Queensland Aboriginal and Torres Strait Islander Health Council (QAIHC)
- Queensland Ambulance Service

Schedule 2 Funding and purchased activity and services

This Schedule 2 sets out:

(a) the services which are to be provided by the HHS;

(b) the activity purchased by the Department from the HHS;

(c) the funding provided for delivery of the purchased activity;

(d) the allocation of funding provided against the care domains of the Outcomes Framework;

(e) the criteria and processes for financial adjustments associated with the delivery of purchased activity and specific funding allocations; and

(f) the sources of funding that this service agreement is based on and the way these funds will be provided to the HHS.

1. Introduction

1.1 The HHS will deliver the services for which funding is provided in this Schedule 2. In providing these services, the HHS will ensure that:

(a) all statewide and national policy frameworks, guidelines, protocols and implementation standards applicable to the service provided are followed;

(b) participation in national programs is facilitated and supported, including where these programs are provided by the Commonwealth Government;

(c) service delivery partnerships, including with other HHSs, primary care organisations and non-government organisations, are maintained and operate effectively;

(d) collaboration and engagement with other service providers and stakeholders is initiated and maintained to ensure that an integrated system of treatment, care and support is in place and to facilitate the delivery of comprehensive and effective services. This may include but is not limited to:

(i) other HHSs;

(ii) non-government organisations;

(iii) Aboriginal and Torres Strait Islander community-controlled health organisations;

(iv) Queensland Ambulance Service;

(v) services provided through the Department of Health (for example, Pathology Queensland);

(vi) primary care providers;

(vii) other government departments and agencies; and

(viii) private providers;

(e) models of care and service delivery arrangements are consistent with evidence-based practice and offer value for money;

(f) services are provided on an equitable basis to the community, and processes are in place to ensure that services reach identified target populations, high risk groups and hard to reach communities;

(g) referral networks and pathways continue to operate effectively; and

(h) innovation and continuous improvement are supported.

2. Purchased health services

2.1 Table 4 shows the allocation of funding from the Department to the HHS across the care domains of the Outcomes Framework. Table 5, Table 6, and Table 7 outline the activity and service streams which the Department agrees to purchase from the HHS pursuant to this service agreement.

2.2 More generally, this will include the following:

2.3 **Statewide Services**

(a) The designation of a service as a statewide service (either a clinical statewide service or a clinical support statewide service) will be determined by the Department, consistent with the stipulated governance arrangements for such services.

(b) The HHS will:

(i) collaborate with the Department and other HHSs in the implementation of, and adherence to, the governance and oversight arrangements for statewide services;

(ii) participate in, and contribute to, the staged review of the purchasing model for identified statewide services; and

(iii) ensure that referral pathways in and out of each statewide service are followed.

2.4 **Clinical Statewide Services and Clinical Support Statewide Services provided**

This clause does not apply to this HHS.

Table 1 Statewide Services

This table does not apply to this HHS.

2.5 **Regional services**

This clause does not apply to this HHS.

2.6 Prevention services and public health services

(a) The HHS will provide a range of prevention and public health services to promote and protect health, prevent illness and disease, and manage risk, including:

(i) Specialist Public Health Units

(ii) environmental health services, including risk assessment, regulation and enforcement in relation to environmental hazards, food safety, medicines and therapeutic goods, mosquitos and other vectors, pest management, poisons, radiation safety, chemical safety and water quality;

(iii) communicable disease services including immunisation, blood-borne viruses, sexually transmissible infections, infection control, notifiable conditions, mosquito-borne disease and tuberculosis;

(iv) management of incidents, emergencies and disasters, and disease outbreak readiness and response services;

(v) preventive health services;

(vi) population health screening including, but not limited to, cancer screening services and newborn blood spot screening;

(vii) public health epidemiology and surveillance;

(viii) mitigation and adaptation in response to climate risks.

(b) Services will be provided in line with public health related legislation and the service delivery and reporting requirements outlined in the Public Health Service Schedule and supported by the *Public Health Practice Manual*, as these relate to the services provided.

(c) Delivery of these services may be coordinated through specialist public health units, sexual health services, tuberculosis services, other areas of the HHS, or a combination of these.

2.7 Aboriginal and Torres Strait Islander health services

The HHS will provide Aboriginal and Torres Strait Islander specific health services and initiatives consistent with the principles and objectives of the Queensland Government's *Making Tracks toward closing the gap in health outcomes for Indigenous Queenslanders by 2033 – Policy and Accountability Framework* and the priorities committed to in the HHS's Health Equity Strategy. These services and initiatives will be delivered in line with guidance from the First Nations Health Office and the *First Nations First Strategy 2032.*

2.8 Mental health, alcohol, and other drugs services

The HHS will provide treatment, care and support for individuals who are, or may be, experiencing substance use disorders and/or other mental health disorders, mental health crisis and suicidal distress, and their families and carers. Services will be delivered in line with guidance from the Mental Health, Alcohol and Other Drugs Strategy and Planning Branch.

2.9 Oral health services

The HHS will provide oral health services to people who meet the eligibility criteria for accessing public dental services in Queensland. Services will be delivered in line with guidance from the Office of the Chief Dental Officer.

2.10 Prisoner health services

This clause does not apply to this HHS.

2.11 Youth detention services

This clause does not apply to this HHS.

2.12 Refugee health

This clause does not apply to this HHS.

2.13 State-funded outreach services

(a) Where state-funded outreach services are provided:

(i) funding for the service will remain part of the providing HHS's funding allocation; and

(ii) the activity must be recorded at the HHS where the outreach service is being provided.

(b) Any changes to the provision of outreach services will follow the requirements set out in clause 3 of this service agreement.

3. Teaching, training, and research

The HHS will provide the teaching, training and research programs for which funding is provided within Schedule 2 and as described below.

3.1 Clinical education and training

(a) The HHS will provide education and training placements for the following professional groups consistent with and proportionate to the capacity of the HHS and will support and align with stipulated placement terms governing clinical placements in Queensland Health facilities:

(i) medical students;

(ii) nursing and midwifery students;

(iii) pre-entry clinical allied health students;

(iv) interns;

(v) rural generalist trainees;

(vi) vocational medical trainees;

(vii) first year nurses and midwives;

(viii) re-entry to professional register nursing and midwifery candidates;

(ix) dental students;

(x) allied health rural generalist training positions;

(xi) Aboriginal and Torres Strait Islander Health Workers and Aboriginal and Torres Strait Islander Health Practitioners.

(b) The HHS will comply with the state-wide vocational medical training pathways.

(c) The HHS will support profession specific and inter-profession statewide allied health clinical education programs.

(d) The HHS will continue to implement and retain the following positions provided through clauses to the *Health Practitioners and Dental Officers (Queensland Health) Certified Agreement (No 4) 2022*:

(i) health practitioner research positions provided through the Research Package for Health Practitioners; and

(ii) clinical educator positions provided through the Clinical Education Management Initiative for Health Practitioners.

(e) The HHS will maintain or increase its contribution of staff to the Queensland Country Relieving Doctors and the receiving HHS will be responsible for wages, clinical governance, and appropriate supervision of the junior medical relievers.

3.2 Statewide training, education, and research

This clause does not apply to this HHS.

3.3 Health and medical research

The HHS will:

(a) develop and implement a strategy to drive increased research activity and its translation into clinical practice;

(b) support increased and equitable access to clinical trials for patients;

(c) support clinicians to undertake research linked to their practice; and

(d) ensure high quality and timely research governance approval processes.

4. Delivery of purchased activity

4.1 The HHS is required to maintain accurate activity forecasts in the purchased target module of the Decision Support System (DSS) at all times. This information is imperative to the Department's assessment of State performance against the national Soft Cap and for outer-year planning. Activity forecasts must accurately reflect financial forecasts reported to the Finance Branch monthly.

4.2 The Department and the HHS will monitor actual activity against purchased levels and will act as necessary to ensure delivery of purchased levels is achieved. The HHS has a responsibility to only recognise revenue that is linked with actual activity delivered against purchased volumes.

4.3 The HHS will actively monitor variances from purchased activity levels and notify the Department as soon as the HHS becomes aware of significant variances.

4.4 The HHS will also notify the Department of deliberate changes to the consistent recording of activity within year that would result in additional activity being recorded for existing health services.

4.5 The HHS will undertake regular quality audits. The HHS is encouraged to publish its data quality framework describing audits undertaken and results achieved. For further information, refer to the Delivery of Purchased Activity – Requirement for Quality Audits specification sheet as detailed in Appendix 1.

4.6 If the HHS wishes to convert activity between purchased activity types, programs, and levels the HHS must negotiate this with the Department based on a sound needs based rationale.

4.7 The Department will reconcile in-scope activity, as defined in the Activity Reconciliation specification sheet (available online, as detailed in Appendix 1), delivered by the HHS against the purchased in-scope activity targets outlined in Table 5.

4.8 Activity reconciliations will be undertaken in the applicable End of Year Technical Amendment Window and subsequent Amendment Window 2 and will be derived through application of the methodology which is documented in the Activity Reconciliation specification sheet.

4.9 Should the HHS be unable to deliver the activity that has been funded a financial adjustment will be applied.

4.10 Funding and corresponding activity that is withdrawn from the HHS may be reallocated to an alternate provider that can undertake the activity.

4.11 Any resulting changes to funding and purchased activity levels will be actioned through the processes outlined in Schedules 2 and 5 of this service agreement.

4.12 The HHS will maintain its efforts to accurately record the delivered activity and submit this data to the Department in accordance with the agreed requirements.

5. Delivery of purchased services

5.1 As part of the service agreement, the Department purchases a range of services (or deliverables) from the HHS for the delivery of certain programs and projects.

5.2 These program or project services may be the subject of separately agreed conditions tied to that funding and the focus of detailed monitoring by the Department.

5.3 Conditions may include, but are not limited to:

(a) establishment and/or commencement of services;

(b) delivery of activity;

(c) workforce obligations;

(d) establishment of oversight committees;

(e) opening or upgrades to facilities;

(f) program evaluation;

(g) program management;

(h) reporting or notification obligations; and

(i) attainment of performance standards.

5.4 The HHS will ensure that the conditions are achieved within the stipulated time period.

5.5 The HHS will notify the Department if the HHS forecasts an inability to achieve program or project objectives or the conditions.

5.6 The Department may withdraw allocated funding pro rata to the level of under delivery if the services for a specified program or project are not being fully delivered according to the objectives or conditions.

5.7 Any resulting changes to funding and purchased activity levels will be actioned through the processes outlined in Schedules 2 and 5 of this service agreement.

6. Financial adjustments

6.1 **Activity targets**

(a) The Department will initiate a joint process with the HHS to determine whether a financial adjustment should be applied in relation to any purchased activity which has breached the thresholds identified bi-annually. This process will take into account any relevant matters that have been identified in a review/analysis of the breach as well as the outcomes of the activity plan implemented to address the activity breach.

(b) Activity will be monitored at the purchasing hierarchy level. Providing the HHS meets all relevant KPIs and specific funding allocations, the HHS has the ability to negotiate the transfer of activity across the purchasing hierarchy with the Department.

(c) Table 2 demonstrates the financial adjustment that will be applied when activity thresholds have been breached.

(d) The HHS may not utilise the provisions within AASB15 Revenue from Contracts with Customers to override the application of any financial adjustment made by the Department in line with Table 2.

Table 2 Financial adjustments applied on breach of activity thresholds

Example of Breach	Description	Financial Adjustment
Over performance	Activity exceeds that specified in the service agreement value for in-scope activity as shown in Table 5.	Purchasing contracts are capped and an HHS will not be paid for additional activity apart from activity that is in scope for the identified purchasing incentives as set out in Table 3 (where applicable.)
Under performance	Activity is below that specified for in-scope activity as shown in Table 5.	Purchased activity and the related funding will be withdrawn at 100% of the Queensland Efficient Price and reallocated to an alternate provider that can undertake the activity. The reconciliation will be undertaken as outlined in the Activity Reconciliation Specification. Refer to Table 5 for the HHS QWAU target.
Failure to deliver on service commitments linked to specific funding allocations	Specific funding allocations<br>National Partnership Agreements.	It is at the discretion of the Department to withdraw allocated funding pro rata to the level of under delivery.
For all other types of activity variance, any financial adjustment will be made at the discretion of the Department.		

6.2 Purchasing approach

(a) The purchasing approach includes a range of funding adjustments (purchasing incentives and ABF model localisations) that aim to incentivise high quality and high priority activity, support innovation and evidence-based practice, deliver additional capacity through clinically and cost-effective models of care and dis-incentivise care providing insufficient or no benefit for patients. This includes incentive payments for HHSs who achieve targets in specific priority areas. The funding adjustments are detailed in Table 3. The Department must reconcile the applicable funding adjustments in Table 3 in line with the timeframes specified in the relevant purchasing specification sheet. The Department must provide a copy of the reconciliation statement to the HHS.

(b) Funding adjustments must be based on the requirements contained in the relevant purchasing specification sheet.

(c) Any resulting changes to funding and purchased activity levels will be actioned through the processes outlined in Schedules 2 and 5 of this service agreement.

Table 3 Funding adjustments 2024/25

Funding adjustment	
**Purchasing incentives**	
Models of care/workforce	This program includes a range of initiatives focusing on incentivising:<br>• specific models of care; and<br>• the use of workforce operating at top of scope where there may be long wait lists and staff have not been available in a traditional model of care.
**ABF model localisations**	
Child Health Checks	QWAU loading for every in-scope check performed.
Unqualified neonate funding	Reduced Diagnosis Related Group (DRG) QWAU for all maternal delivery episodes with a liveborn outcome, discounted by the Diagnosis Related Group (DRG), with QWAUs re-allocated for unqualified neonates.
Maternity care for First Nations women	QWAUs to incentivise maternity care provided to First Nations mothers during pregnancy and to incentivise smoking cessation during pregnancy.
Sentinel events	Payment withdrawn for sentinel events as per the national ABF model.
Advance Care Planning (ACP)	QWAUs for HHSs offering ACP discussions to admitted patients, non-admitted outpatients, community health patients and Emergency Department patients.
Emergency Department Did Not Wait (DNW)	Zero QWAUs for DNWs.
Fractured neck of femur	QWAUs reduced by 20% for non-timely surgical treatment of fractured neck of femur.
Hospital in The Home (HITH)	QWAUs increased by 12.5% for Hospital in the Home (HITH) admissions of Residential Aged Care Facility residents.
Out-of-scope services	Nil QWAUs for out-of-scope procedures.
Pre-operative bed days	QWAUs for long stay days above the upper trim point less ICU days reduced equivalent to pre-operative days, up to a maximum of 3 days, for elective episodes.
Smoking cessation (community mental health)	QWAUs for smoking cessation activity for community mental health patients.
Smoking cessation (inpatients)	QWAUs for smoking cessation activity for publicly funded inpatients.
Stroke care	10% QWAU loading for acute stroke patients admitted to Queensland Stroke Clinical Network-endorsed stroke unit care.
Telehealth (admitted patients)	QWAUs for provider-end of in-scope admitted patient telehealth activity.
Allied Health Led Workforce for Pelvic Health and Gastroenterology	QWAU loading for an in-scope service event for Pelvic Health and Gastroenterology recorded against an Other Health Professional.
Remote Patient Monitoring	QWAU loading for an in-scope non-admitted remote patient monitoring encounter per month per patient.

Surgery Connect reimbursements

(a) The HHS will reimburse the Department for the actual costs of activity for nominated patient referrals to the Surgery Connect program where:

(i) The HHS has nominated the patient referral as HHS funded or HHS Direct on entry of the referral in the Surgery Connect Activity Navigator (SCAN); and

(ii) The HHS Chief Finance Officer has recorded approval of the nomination in SCAN;

or

(iii) The HHS has obtained the Department's agreement to retrospectively convert a defined patient cohort to HHS funded status in SCAN.

(b) The HHS may only request retrospective conversion of activity to HHS funded within the financial year in which the activity has taken place.

(c) Any resulting changes to funding and purchased activity levels will be actioned through the processes outlined in Schedules 2 and 5 of this service agreement.

6.3 Financial adjustments – other

(a) Notwithstanding the provisions regarding the recognition of revenue as stipulated in AASB1058 Income of Not-for-Profit Entities and/or AASB15 Revenue from Contracts with Customers, the Department may seek to recover funding from an HHS that was provided through this service agreement which has:

(i) not been utilised in accordance with its intended purpose; and/or

(ii) not been utilised within the prescribed time period to deliver the agreed outcomes/services.

(b) If the Chief Executive (or delegate) determines that previously allocated funding is to be recovered, any resulting changes to funding and purchased activity levels will be actioned through the processes outlined in Schedules 2 and 5 of this service agreement.

6.4 Public and private activity/own source revenue

(a) Own source revenue comprises grants and contributions, user charges and other revenues.

(b) Where an HHS is above its own source revenue target, it will be able to retain the additional own source revenue with no compensating adjustments to funding from other sources where the additional revenue is not attributable to a private patient consistent with clauses A9 to A13 and A44 of the *National Health Reform Agreement.*

(c) Where an HHS is below its own source revenue target in respect of private patients, it will experience a reduction in revenue with no compensating adjustments to funding from other sources.

(d) The HHS will make all efforts to identify and maximise revenue from appropriate third parties to ensure optimisation of available State and Commonwealth funding across the Queensland public sector health system.

(e) The own source revenue identified in Table 5 is an estimate generated by the HHS which allows all third-party funding sources associated with service delivery

to be identified. The HHS will ensure that this estimate is substantiated and accurate to ensure no significant variances to actual revenue generated.

(f) The HHS will routinely revise and update the estimate to ensure alignment between the service agreement and Queensland Treasury’s reporting system (TRIDATA).

(g) Budget adjustments for changes in own source revenue from private patients will be actioned through the processes set out in Schedule 5 of this service agreement.

7. Funding sources

7.1 The four main funding sources contributing to the HHS service agreement value are:

(a) Commonwealth funding;

(b) State funding;

(c) grants and contributions; and

(d) own source revenue.

7.2 Table 5 provides a summary of the funding sources for the HHS and the total value of the service agreement.

7.3 The HHS must undertake regular quality audits to check for potential duplicates in funding source, in particular the National Health Reform Agreement and Medicare given the Commonwealth’s contribution to both funding sources. The HHS should take active steps to remedy areas of concern. A consumer’s choice of funding arrangement should be reflected on a patient election form.

8. Funds disbursement

8.1 The Department agrees to pay the HHS the amount described in Table 5 of Schedule 2, subject to:

(a) parliamentary appropriation and adequate funds being allocated to the Department; and

(b) the terms of this service agreement.

8.2 All payments under this service agreement will be made in accordance with the requirements of the *National Health Reform Agreement* and the Act.

8.3 The Chief Executive will direct the disbursement of both State and Commonwealth funding from the State Pool Account, the State Managed Fund, and the Department of Health Expenditure account to the HHS.

8.4 However, the State (represented by the Chief Executive) will not:

(a) redirect Commonwealth payments between HHSs;

(b) redirect Commonwealth payments between funding streams (e.g., from ABF to block funding); and/or

(c) adjust the payment calculations underpinning the Commonwealth's funding.

8.5 The Department will pay state-funded activity-based funding, block funding and system manager funding to the HHS on a fortnightly basis in line with receipt of the State Appropriation Payment. The payment made will be equal to 1/26th of the state-funded component of the service agreement value described in Table 5.

8.6 The Department will pay Commonwealth-funded activity-based funding and block funding monthly in line with receipt of the Commonwealth payment. The payment made will be equal to 1/12th of the Commonwealth funded component of the service agreement value described in Table 5.

8.7 Where the parties have agreed to amend the service agreement value or the Commonwealth contribution amount changes, the fortnightly and/or monthly payments will be adjusted to reflect the amended service agreement value.

8.8 Service agreement payments may be made outside of these timeframes with the approval of the Department's Chief Finance Officer.

Table 4 HHS Funding by Outcomes Framework Care Domain 2024/25

Care Domain	Funding $	QWAU (Q27)
Prevention, early intervention, and primary health care	$101,299,717	2,670
Trauma and illness	$103,275,899	4,024
Mental health and alcohol and other drugs	$19,721,992	1,392
Cancer	$4,198,393	200
Planned care	$26,213,097	1,436
Maternity and neonates	$22,326,850	855
Chronic and complex	$70,247,161	2,739
Statewide services	$352,542	23
Depreciation	$26,293,000	0
**TOTAL**	**$373,928,651**	**13,339**

Table 5 HHS Total Funding Allocation by Funding Source 2024/25

Funding Source	24-25 NWAU (N2425)	24-25 QWAU (Q27)	24-25 Agreed ($)
**NHRA Funding**			
**ABF Pool**			
**ABF Funding (In scope NHRA)[2]**			
Commonwealth	0		$0
State		0	$0
State Specified Grants			$0
State-wide Services			$0
Restoring Planned Care	0	0	$0
Long Stay Patient Recovery Funding	0	0	$0
**Total ABF Funding (in scope NHRA)**	**0**	**0**	**$0**
**State Managed Fund**			
**Block Funding (State and Commonwealth)**			
Small rural hospital		10,506	$161,558,745
Teaching, Training and Research			$654,330
Other Mental Health	1,283	1,283	$9,052,827
Non-Admitted Home Ventilation			$0
Residential Mental Health Services		0	$0
Other Non-admitted Service			$0
Highly Specialised Therapies			$0
Other Public Hospital Programs			$0
**Total NHRA Funding**	**-**	**11,789**	**$171,265,902**
**Out of Scope NHRA**			
**Queensland ABF Model**			
DVA		16	$96,329
NIISQ/MAIC		0	$10,173
Oral Health		706	$5,103,869
Oral Health – FFA		28	$161,936
BreastScreen		0	$0
Child Health Checks		52	$965,570
**Total Queensland ABF Funding**		**802**	**$6,337,877**
**Discretely Funded Programs[3]**			
Department of Health			$127,134,174
Locally Receipted Funds			$1,603,359
Research (Other OSR)			$0
**Total Discretely Funded Programs**			**$128,737,533**

[2] The split between Commonwealth and State NHRA funding will change during the year as the purchased activity targets are updated and the National Health Funding Board updates their payment advice accordingly.

[3] Includes all other (non-ABF) State and Commonwealth funded health services including, but not limited to, Prisoner Health, Public Health and Prevention Services.

Funding Source	24-25 NWAU (N2425)	24-25 QWAU (Q27)	24-25 Agreed ($)
**Own Source Revenue**			
Private Patient Admitted Revenue[4]	16	16	$95,504
Pharmaceuticals Benefits Scheme		357	$1,229,406
Non-Admitted Services		329	$15,846
Other Activities[5]		45	$14,690,688
Oral Health – CDBS		0	$177,114
**Total Own Source Revenue**	**-**	**747**	**$16,208,557**
Locally Receipted Funds (exc. Discretely Funded Programs)[6]			$25,085,781
Depreciation			$26,293,000
**GRAND TOTAL**	**0**	**13,338**	**$373,928,650**

Pool Accounts			
ABF Pool (National Health Funding Pool)[7]			$0
State Managed Fund[8]			$171,265,902
System Manager			$133,472,051

[4] The estimated value of the revenue earned from private patients, based on OSR estimates provided by the HHS.

[5] Incorporates all OSR which is not identified elsewhere in Table 5.

[6] Includes items such as training programs and donations. Does not include locally receipted funds associated with discretely funded programs, e.g.Transition Care.

[7] Articulates the financial payment made to support in-scope ABF services under the NHRA including DVA, NIISQ, MAIC and Breastscreen Services. Applies to all HHSs except Central West HHS and Torres and Cape HHS.

[8] Articulates the payment made for block funded services under the NHRA, DVA, NIISQ and MAIC services.

Table 6 National Health Reform Funding

NHRA Funding Type	No. of In-scope services (NWAU)	No. of Out-of-scope services (QWAU)	Total Services (WAU)	ABF NEP ($)	State Price ($)	Funding for In-scope services at NEP ($)	Cwlth contribution for In-scope services ($)	State contribution for In-scope services ($)	State contribution for Out-of-scope services ($)	Total Cwlth and State contribution ($)
National Efficient Price (NEP)		*a,b*		*c*	*d*			*e*		
**ABF Allocation (NWAU)**										
Emergency Department	0	0	0	$6,465	$5,878	0	0	0	0	0
Acute Admitted	0	0	0	$6,465	$5,878	0	0	0	0	0
Admitted Mental Health	0	0	0	$6,465	$5,878	0	0	0	0	0
Sub-Acute	0	0	0	$6,465	$5,878	0	0	0	0	0
Non-Admitted	0	0	0	$6,465	$5,878	0	0	0	0	0
**Total ABF Allocation**	**0**	**0**	**0**			**0**	**0**	**0**	**0**	**0**
**Block Allocation**										
Teaching, Training, and Research						0	173,865	480,465	0	654,330
Small and Rural Hospitals[9]						0	46,416,284	115,142,461	6,337,877	167,896,622

[9] Incorporating small regional and rural public hospitals, four specialist mental health facilities (Baillie Henderson Hospital, Jacaranda Place – Queensland Adolescent Extended Treatment Centre, The Park – Centre for Mental Health and Kirwan Rehabilitation Unit) and the Ellen Barron Family Centre.

NHRA Funding Type	No. of In-scope services (NWAU)	No. of Out-of-scope services (QWAU)	Total Services (WAU)	ABF NEP ($)	State Price ($)	Funding for In-scope services at NEP ($)	Cwlth contribution for In-scope services ($)	State contribution for In-scope services ($)	State contribution for Out-of-scope services ($)	Total Cwlth and State contribution ($)
Other Mental Health						0	3,374,269	5,678,558	0	9,052,827
Non-Admitted Home Ventilation						0	0	0	0	0
Other Non-Admitted Services						0	0	0	0	0
Other Public Hospital Programs						0	0	0	0	0
Highly Specialised Therapies						0	0	0	0	0
**Total Block Allocation**						**0**	**49,964,418**	**121,301,484**	**6,337,877**	**177,603,779**
**Grand Total Funding Allocation**										**177,603,779**

Notes

a. QWAU refers to Queensland Weighted Activity Units in Q27 phase (built on N2425)
b. DVA, NIISQ/MAIC, Oral Health, Child Health Checks and BreastScreen
c. Queensland Efficient Price used to Purchase growth QWAUs
d. NWAU x NEP
e. State funding transacted through the Pool/State Managed Fund Account; not covered under the NHRA

- NWAU estimates do not take account of cross-border activity.

Table 7 Discretely Funded Programs (Non-ABF)

Discretely Funded Programs	Revenue Models	$
Aged Care Assessment Program	Commonwealth	$0
Alcohol, Tobacco and Other Drugs	State	$3,965,350
Community Health Programs	State	$46,018,838
Interstate Patients (QLD Residents)	State	$179,516
Other State Funding	State	$43,844,496
Patient Transport: PTSS	State	$22,282,504
Patient Transport: Aeromedical Retrieval	State	$567,943
Patient Transport	State	$0
Prevention Services and Public Health	Commonwealth	$3,997,419
	State	$224,351
Prisoner Primary Health Services	State	$0
Disability Residential Care Services	State	$0
Torres Strait Treaty	Commonwealth	$0
Multi-Purpose Health Services	Commonwealth	$5,813,142
Residential Aged Care Services	Commonwealth	$127,625
	Locally Receipted Funds	$0
	State	$112,990
Transition Care	Locally Receipted Funds	$0
Research	Commonwealth	$0
	OSR	$0
Home and Community Care (HACC) Program	Locally Receipted Funds	$1,603,359
**Discretely Funded Programs Total**		**$128,737,533**
**TOTAL**		**$128,737,533**

Schedule 3 Department of Health Provided Services

1. In scope services and service schedules

Table 8 Department of Health provided services and service schedules

Provider	Service provided	Link to Service Statement
Corporate Services Division (CSD)	• Corporate Enterprise Solutions<br>• Finance Branch:<br>○ Accounts Payable Service Provision<br>○ Banking and Payment Services<br>• Central Pharmacy<br>• Group Linen Services<br>• Transport and Logistic Services<br>• Supply Chain Services	CSD Service Schedules
eHealth Queensland (eHQ)	• ICT Service	eHQ Service Schedule
Queensland Public Health and Scientific Services Division (QPHaSS)	• Pathology Queensland<br>• Biomedical Technical Services<br>• Public Health Services	QPHaSS Service Schedules

Schedule 4 Performance Measures

1. Performance Measures

1.1 The performance of the HHS will be measured according to the assessment criteria and processes described in the Performance and Accountability Framework.

1.2 Existing performance indicators are mapped to the care domains of the Outcomes Framework.

1.3 The detailed specification for each of the performance measures listed in this service agreement are provided through performance measure attribute sheets.

1.4 The performance measures identified in this service agreement are applicable to the HHS unless otherwise specified within the attribute sheet.

1.5 HHSs are also required to report against the agreed Statewide Health Equity Key Performance Measures (Table 12).

Table 9 HHS Performance Measures – Key Performance Indicators

Outcomes Framework Care Domain	Key Performance Indicators	Indicator Number
Chronic and complex	Hospital Acquired Complications (IHACPA code 8, 11, 13, 14)	31
Chronic and complex	Potentially Preventable Hospitalisations – First Nations peoples:<br>• Diabetes complications<br>• Selected conditions	37a<br>37b
Chronic and complex	Potentially Preventable Hospitalisations (non-diabetes complications)	39
Chronic and complex	Proportion of diabetic patients with a current HbA1c result	59
Chronic and complex	Potentially avoidable deaths - First Nations Peoples	70
Maternity and neonates	Hospital Acquired Complications (IHACPA code 15,16)	31
Maternity and neonates	% of low birthweight babies born to Queensland mothers	41
Maternity and neonates	Proportion of healthy birthweight babies (2,500 grams to 4,499 grams at birth) – Aboriginal and Torres Strait Islander babies	71
Mental health, alcohol, and other drugs	Proportion of mental health and alcohol and other drug service episodes with a documented care plan	27
Mental health, alcohol, and other drugs	Suicide count and rate – First Nations Peoples	72
Other	Forecast operating position:<br>• Full year<br>• Year to date	48<br>49
Other	Average sustainable Queensland Health FTE	50
Other	Capital expenditure performance	51

Outcomes Framework Care Domain	Key Performance Indicators	Indicator Number
Planned care	Elective surgery patients waiting longer than the clinically recommended timeframe	9
Planned care	Gastrointestinal endoscopy patients waiting longer than the clinically recommended timeframe	16
Planned care	Telehealth utilisation rates:<br>• Number of non-admitted telehealth service events	20
Planned care	Hospital Acquired Complications (IHACPA code 1,2,3,4,6,7,9,10,12)	31
Planned Care	Missed Opportunity to Treat – Outpatients	73
Prevention, early intervention, and primary health care	Access to oral health services (adults)	21
Prevention, early intervention, and primary health care	The percentage of oral health activity which is preventive	23
Prevention, early intervention, and primary health care	Patients whose smoking status has been recorded	58
Prevention, early intervention, and primary health care	Access to oral health services (children)	67
Prevention, early intervention, and primary health care	Potentially avoidable deaths - First Nations Peoples	70
Prevention, early intervention, and primary health care	Suicide count and rate – First Nations Peoples	72
Prevention, early intervention, and primary health care	*Care Closer to Home (placeholder):*<br>• *Self-sufficiency: admitted patients*<br>• *Self-sufficiency: non-admitted patients*	*NA*
Prevention, early intervention, and primary health care	*Participation in the Practice Incentive Program (placeholder)*	*NA*

Table 10 HHS Performance Measures - Safety and Quality Markers

Outcomes Framework Care Domain	Safety and Quality Markers	Indicator Number
Maternity and neonates	Sentinel Events	32
Planned care	Sentinel Events	32
Planned care	Hospital Standardised Mortality Ratio	33
Planned care	Severity Assessment Code (SAC1) analysis completion rates	34
Planned care	Patient Reported Experience	68

Table 11 HHS Performance Measures – Outcome Indicators

Outcomes Framework Care Domain	Outcome Indicators	Indicator Number
Chronic and complex	Advance care planning	43
Chronic and complex	Integrated care pathways:<br>• Care pathway in place for patients with identified co-morbidities	60
Mental health, alcohol and other drugs	Smoking cessation clinical pathway (community mental health)	42
Other	First Nations peoples' representation in the workforce	47
Planned care	Complaints resolved within 35 calendar days	36
Prevention, early intervention, and primary health care	General oral health care for First Nations peoples	22
Prevention, early intervention, and primary health care	Access to emergency dental care	24
Prevention, early intervention, and primary health care	Smoking cessation clinical pathway (admitted episodes and dental)	42
Prevention, early intervention, and primary health care	Adolescent vaccinations administered via the statewide School Immunisation Program	45

Table 12 Statewide Health Equity Key Performance Measures

Outcomes Framework Care Domain	Key Performance Measures	Indicator Number
Chronic and complex	Advance care planning	43
Chronic and complex	Integrated care pathways - Rural and Remote HHSs:<br>• Care pathway in place for patients with identified co-morbidities	60
Maternity and neonates	Proportion of healthy birthweight babies (2,500 grams to 4,499 grams at birth) – Aboriginal and Torres Strait Islander babies	71
Mental health, alcohol, and other drugs	Face to face community follow up within 1-7 days of discharge from an acute mental health inpatient unit	26
Mental health, alcohol, and other drugs<br>Chronic and complex	Suicide count and rate – First Nations People	72
Other	First Nations peoples' representation in the workforce	47
Planned care	Category 1 elective surgery patients treated within the clinically recommended timeframe	7
Planned care	Category 2 and 3 elective surgery patients treated within the clinically recommended timeframe	8
Planned care	Category 1 patients who receive their initial specialist outpatient appointment within the clinically recommended timeframe	17
Planned care	Category 2 and 3 patients who receive their initial specialist outpatient appointment within the clinically recommended timeframe	18

Outcomes Framework Care Domain	Key Performance Measures	Indicator Number
Prevention, early intervention, and primary health care	General oral health care for First Nations peoples	22
Prevention, early intervention, and primary health care	Potentially avoidable deaths – First Nations peoples	70

Schedule 5 Amendments to this service agreement

1. Agreed process to amend this service agreement

1.1 The parties acknowledge that this service agreement is subject to amendment, which will generally occur through:

(a) amendment windows;

(b) extraordinary amendment;

(c) periodic adjustments; and

(d) end of financial year reconciliation.

1.2 **Amendment windows**

(a) There will be set periods of time nominated by the Department during the year (amendment windows) in which a party may propose an amendment and the parties will endeavour to negotiate and finalise proposals to amend this service agreement.

(b) Amendment proposals that are agreed will be documented in a deed of amendment to this service agreement.

(c) Further details on the amendment window process, including the timing of amendment windows, is provided online as detailed in Appendix 1.

1.3 **Extraordinary Amendment**

(a) Outside an amendment window, the Department and the HHS agree to limit any proposal to amend the terms of this service agreement to those where there is an urgent priority need to facilitate a funding allocation (extraordinary amendment). The parties will endeavour to negotiate and finalise any such proposal urgently.

(b) The process for submitting, negotiating, and resolving an extraordinary amendment is available online as detailed at Appendix 1.

(c) Agreed extraordinary amendments will be reflected in a notice issued by the Chief Executive and countersigned as accepted by the HHS. The notice will be replaced when the extraordinary amendment is subsequently formalised in a deed of amendment issued following the next amendment window.

1.4 **Periodic adjustments**

(a) The service agreement value (and corresponding purchased activity) may be adjusted at any time to reflect funding variations that:

(i) occur on a periodic basis or in line with adjustments permitted for specific funding allocations;

(ii) are referenced in the service agreement; and

(iii) are based on a clearly articulated formula, an agreed basis or such other reasonably substantiated basis tied to performance.

(b) Periodic adjustments will be reflected in an adjustment notice issued by the Chief Executive (or delegate) to the HHS, based on relevant data, and subsequently formalised in a deed of amendment issued following the next amendment window.

1.5 **End of financial year reconciliation**

(a) There will be an end of financial year reconciliation process, with the scope defined by the Department and informed by Queensland Government Central Agency requirements.

(b) The Department will provide the HHS with a reconciliation of all service agreement funding and purchased activity for the prior financial year. This will reflect the position following conclusion of the end of financial year adjustments process.

(c) The impact of end of financial year adjustments on subsequent year funding and activity will be incorporated in the service agreement through the deed of amendment executed following the next available amendment window.

(d) This clause will survive expiration of this service agreement.

Appendix 1 Reference Documents

Service Agreement:

- Data supply requirements
- Delivery of Purchased Activity – Requirement for Quality Audits specification sheet
- Dispute resolution process - current
- First Nations First Strategy 2032
- Funding Outcomes Framework
- Hospital and Health Boards Act 2011
- Joint Regional Needs Assessment Framework
- Making Tracks Together: Queensland's Aboriginal and Torres Strait Islander Health Equity Framework
- Making Tracks toward closing the gap in health outcomes for Indigenous Queenslanders by 2033 - Policy and Accountability Framework
- National Agreement on Closing the Gap
- National Health Reform Agreement (NHRA) 2020-25
- Performance Measures Attribute Sheets
- Public Health Practice Manual
- Queensland Government Building Policy Framework and Guideline
- Queensland Health Performance and Accountability Framework
- Service agreement amendment processes
- Specifications supporting the Healthcare Purchasing Model
- Statewide services reference material

Supporting Policy documents

- Aboriginal and Torres Strait Islander Health Workforce Strategic Framework 2016-2026
- Department of Health Strategic Plan 2021-2025

- HEALTHQ32: A vision for Queensland's health system
- My health, Queensland's future: Advancing health 2026
- Queensland Health Equity, Diversity, and Inclusion Statement of Commitment
- System Outlook to 2026 - for a sustainable health service

Contracting and Performance Management Branch
GPO Box 48
Brisbane QLD 4001 Australia

Tel (+61) (07) 3708 5869

www.health.qld.gov.au

Service Agreement

2019/20 – 2021/22

Townsville Hospital and Health Service

July 2020 Revision

Queensland Government

Townsville Hospital and Health Service

Service Agreement 2019/20 - 2021/22, July 2020 Revision

Published by the State of Queensland (Queensland Health), July 2020

This document is licensed under a Creative Commons Attribution 3.0 Australia licence.
To view a copy of this licence, visit creativecommons.org/licenses/by/3.0/au

© State of Queensland (Queensland Health) **2020**

You are free to copy, communicate and adapt the work, as long as you attribute the State of Queensland (Queensland Health).

For more information contact:
Contracting and Performance Management Branch, Department of Health, GPO Box 48, Brisbane QLD 4001, email SAM@health.qld.gov.au, phone (07) 3708 5869.

An electronic version of this document is available at
www.health.qld.gov.au/system-governance/health-system/managing/default.asp

Disclaimer:
The content presented in this publication is distributed by the Queensland Government as an information source only. The State of Queensland makes no statements, representations or warranties about the accuracy, completeness or reliability of any information contained in this publication. The State of Queensland disclaims all responsibility and all liability (including without limitation for liability in negligence) for all expenses, losses, damages and costs you might incur as a result of the information being inaccurate or incomplete in any way, and for any reason reliance was placed on such information.

Contents

1. Introduction 6
2. Interpretation 6
3. Legislative and regulatory framework 7
4. Health system priorities 7
5. Objectives of the Service Agreement 9
6. Scope 9
7. Performance and Accountability Framework 9
8. Period of this Service Agreement 10
9. Amendments to this Service Agreement 10
10. Publication of amendments 10
11. Cessation of service delivery 10
12. Commencement of a new service 11
13. Provision of data to the Chief Executive 12
14. Dispute resolution 12
15. Force Majeure 15
16. Hospital and Health Service accountabilities 16
17. Department accountabilities 16
18. Insurance 18
19. Indemnity 18
20. Indemnity arrangements for officers, employees and agents 19
21. Legal proceedings 19
22. Sub-contracting 19
23. Counterparts 20

Execution 21

Schedule 1 HHS Accountabilities 22
1. Purpose 22
2. Registration, credentialing and scope of clinical practice 22
3. Clinical Services Capability Framework 22
4. Clinical Prioritisation Criteria 23
5. Service delivery 23
6. Accreditation 24
7. Responsive regulatory process for accreditation 25
8. Achieving health equity for First Nations Queenslanders 26
9. Provision of Clinical Products/Consumables in outpatient settings 27
10. Capital, land, buildings, equipment and maintenance 28
11. Occupational health and safety 30
12. Workforce management 30
13. Medically authorised ambulance transports 31

Schedule 2 Funding, purchased activity and services 32
1. Purpose 32
2. Delivery of purchased activity 32
3. Financial adjustments 33

4. Funding sources 51
5. Funds disbursement 53
6. Purchased services 62
7. Teaching training and research 70
Schedule 3 Performance Measures 74
1. Purpose 74
2. Performance Measures 74
Schedule 4 Data Supply Requirements 79
1. Purpose 79
2. Principles 79
3. Roles and responsibilities 79
Attachment A Data Supply Requirements 81
Schedule 5 Amendments to this Service Agreement 83
1. Purpose 83
2. Principles 83
3. Process to amend this Service Agreement 83
Schedule 6 Definitions 88
Key Documents 95
Abbreviations 97

Figures

Figure 1 Dispute resolution process 13
Figure 2 Inter-HHS dispute resolution process 15
Figure 3 Amendment Proposal negotiation and resolution 85

Tables

Table 1 Specific Funding Commitments 34
Table 2 Purchasing Incentives 2020/21 (Summary) 50
Table 3 Hospital and Health Service funding sources 2020/21 52
Table 4 HHS Finance and Activity Schedule 2019/20 – 2021/22 – Summary by Purchasing Hierarchy 54
Table 5 Minor Capital and Equity 56
Table 6 HHS Finance and Activity Schedule 2019/20 – 2021/22 Other Funding Detail 57
Table 7 Specified Grants 60
Table 8 Hospital and Health Service Service Agreement and State Level Block Payments to state managed funds from Commonwealth payments into national funding pool 61
Table 9 HHS Performance Measures – Key Performance Indicators 75
Table 10 HHS Performance Measures - Safety and Quality Markers 76
Table 11 HHS Performance Measures – Outcome Indicators 77
Table 12 Clinical data 81
Table 13 Non-clinical data 82
Table 14 Amendment Window Exchange Dates 84

1. Introduction

1.1 The Queensland Public Sector Health System is committed to strengthening performance and improving services and programs in order to meet the needs of the community and deliver improved health outcomes to all Queenslanders.

1.2 The development of Service Agreements between the Chief Executive and Hospital and Health Services (HHSs), assists this process by formally assigning accountability for the high-level outcomes and targets to be met during the period to which the Service Agreement relates.

1.3 The content and process for the preparation of this Service Agreement is consistent with the requirements of the *Hospital and Health Boards Act 2011*. As such this Service Agreement specifies:

(a) the Health Services and other services to be provided by the HHS;

(b) the funding which is provided to the HHS for the provision of these services and the way in which the funding is to be provided;

(c) the Performance Measures that the HHS will meet for the services provided;

(d) data supply requirements; and

(e) other obligations of the Parties.

1.4 Fundamental to the success of this Service Agreement is a strong collaboration between the HHS and its Board and the Department. This collaboration is supported through regular Performance Review Meetings attended by representatives from both the HHS and the Department which provide a forum within which a range of aspects of HHS and system wide performance are discussed and jointly addressed.

2. Interpretation

Unless expressed to the contrary, in this Service Agreement:

(a) words in the singular include the plural and vice versa;

(b) any gender includes the other genders;

(c) if a word or phrase is defined its other grammatical forms have corresponding meanings;

(d) “includes” and “including” are not terms of limitation;

(e) no rule of construction will apply to a clause to the disadvantage of a Party merely because that Party put forward the clause or would otherwise benefit from it;

(f) a reference to:

(i) a Party is a reference to a Party to this Service Agreement;

(ii) a person includes a partnership, joint venture, unincorporated association, corporation and a government or statutory body or authority;

(iii) a person includes the person's legal personal representatives, successors, assigns and persons substituted by novation;

(g) any legislation includes subordinate legislation under it and includes that legislation and subordinate legislation as modified or replaced;

(h) a reference to a role, function or organisational unit is deemed to transfer to an equivalent successor role, function or organisational unit in the event of organisational change or restructure in either Party;

(i) an obligation includes a warranty or representation and a reference to a failure to comply with an obligation includes a breach of warranty or representation;

(j) headings do not affect the interpretation of this Service Agreement;

(k) unless the contrary intention appears, a reference to a Schedule, annexure or attachment is a reference to a Schedule, annexure or attachment to this Service Agreement; and

(l) unless the contrary intention appears, words in the Service Agreement that are defined in Schedule 6 'Definitions' have the meaning given to them in that Schedule.

3. Legislative and regulatory framework

3.1 This Service Agreement is regulated by the National Health Reform Agreement and the provisions of the *Hospital and Health Boards Act 2011.*

3.2 The National Health Reform Agreement requires the State of Queensland to establish Service Agreements with each HHS for the purchasing of health services and to implement a performance and accountability framework including processes for remediation of poor performance. The *Hospital and Health Boards Act 2011* states under section 35(3) that the Service Agreement executed between the Chief Executive and the Hospital and Health Board Chair binds each of them.

3.3 The *Hospital and Health Boards Act 2011* recognises and gives effect to the principles and objectives of the national health system agreed by the commonwealth, state and territory governments, including the Medicare principles and health system principles set out in section 4. Section 5 of the *Hospital and Health Boards Act 2011* states that the object of the Act is to establish a Public Sector Health System that delivers high-quality hospital and other Health Services to persons in Queensland having regard to the principles and objectives of the national health system. This Service Agreement is an integral part of implementing these objectives and principles.

4. Health system priorities

4.1 Ensuring the provision of Public Sector Health Services across Queensland requires clear priorities, supportive leadership and staff who work together and across each level of the Public Sector Health System. The Parties recognise that they each have a mutual and reciprocal obligation to work collaboratively with each other, with other Hospital and Health

Services (HHS) and with the Queensland Ambulance Service in the best interests of the Queensland Public Sector Health System.

4.2 The priorities, goals and outcomes for the Queensland Public Sector Health System are defined through:

(a) *Our Future State: Advancing Queensland's Priorities* - the Queensland Government's objectives for the community; and

(b) *My health, Queensland's future: Advancing health 2026* – the vision and strategy for Queensland's health system.

4.3 The Parties will also work collaboratively to deliver the *Queensland Health 2020/21 System Priorities.* The *Queensland Health 2020/21 System Priorities* establishes a tactical framework which will ensure that the Queensland Public Sector Health System delivers sustainable, high quality and timely Health Services during 2020/21, whilst remaining positioned to respond effectively to the COVID-19 pandemic.

4.4 Additionally, the Queensland Government, Premier or the Minister for Health and Minister for Ambulance Services (The Minister) may articulate key priorities, themes and issues from time to time.

4.5 HHSs have a responsibility to ensure that the delivery of Public Sector Health Services in Queensland is consistent with these strategic directions and priorities.

4.6 The Parties will collectively identify, develop, implement and evaluate strategies that support the delivery of priorities identified by the Minister, and which align with a Value-Based Healthcare approach to the delivery of Health Services.

4.7 In accordance with section 9 of the *Financial and Performance Management Standard 2009*, HHSs are required to develop a strategic plan. The HHS's strategic plan will reflect local priorities and will be developed considering the shared Queensland priorities outlined in the Queensland Government's objectives for the community, the Ministers' articulated priorities and *My health, Queensland's future: Advancing health 2026*.

4.8 The Parties have a collective responsibility to contribute to a sustainable Public Sector Health System in Queensland. Planning and delivery of Health Services will be aligned with the system planning agenda set out in *Queensland Health System Outlook to 2026 for a sustainable health service* in order to ensure a coordinated, system-wide response to growing demand for Health Services.

4.9 In delivering Health Services, HHSs are required to meet the applicable conditions of all national agreements and national partnership agreements between the Queensland Government and the Commonwealth Government and commitments under any related implementation plans.

4.10 This Service Agreement is underpinned by and is to be managed in line with the following supporting documents:

(a) Queensland Health System Outlook to 2026 for a sustainable health service;

(b) Performance and Accountability Framework 2020/21; and

(c) Purchasing Policy and Funding Guidelines 2020/21.

5. Objectives of the Service Agreement

This Service Agreement is designed to:

(a) specify the Health Services, teaching, research and other services to be provided by the HHS;

(b) specify the funding to be provided to the HHS for the provision of the services;

(c) specify the Performance Measures for the provision of the services;

(d) specify the performance and other data to be provided by the HHS to the Chief Executive;

(e) provide a platform for greater public accountability; and

(f) facilitate the achievement of State and Commonwealth Government priorities, services, outputs and outcomes while ensuring local input.

6. Scope

6.1 This Service Agreement outlines the services that the Department will purchase from the HHS during the period of this Service Agreement.

6.2 This Service Agreement does not cover the provision of clinical and non-clinical services by the Department, including the Queensland Ambulance Service, to the HHS. Separate arrangements will be established for those services provided by Health Support Queensland and eHealth Queensland.

7. Performance and Accountability Framework

7.1 The Performance and Accountability Framework sets out the framework within which the Department, as the overall manager of Public Health System Performance, monitors and assesses the performance of Public Sector Health Services in Queensland. The systems and processes employed for this purpose include, but are not limited to, assessing and monitoring HHS performance, reporting on HHS performance and, as required, intervening to manage identified performance issues.

7.2 During 2020/21 the Performance and Accountability Framework will support delivery of the *Queensland Health System Priorities 2020/21* which focus on realising positive changes to the Public Sector Health System through providing sustainable, timely, safe and high-quality Health Services in the right setting whilst remaining ready to respond to the COVID-19 pandemic.

7.3 The Performance and Accountability Framework uses Performance Measures to monitor the extent to which HHSs are delivering the high-level objectives set out in this Service Agreement. The Key Performance Indicators and other measures of performance against which the HHS will be assessed and benchmarked are detailed in Schedule 3 of this Service Agreement.

7.4 The Parties agree to constructively implement the Performance and Accountability Framework.

8. Period of this Service Agreement

8.1 This Service Agreement commences on the Effective Date and expires on 30 June 2022. The Service Agreement framework is in place for three years in order to provide HHSs with a level of guidance regarding funding and purchased activity for the outer years.

8.2 In this Service Agreement, references to years are references to the period commencing on 1 July and ending on 30 June unless otherwise stated.

8.3 Using the provisions of the *Hospital and Health Boards Act 2011* as a guide, the Parties will enter into funding and purchased activity negotiations for the following year six months before the end of the current year.

8.4 In accordance with the *Hospital and Health Boards Act 2011* the Parties will enter negotiations for the next Service Agreement at least six months before the expiry of the existing Service Agreement.

9. Amendments to this Service Agreement

9.1 Section 39 of the *Hospital and Health Boards Act 2011* requires that, if the Chief Executive or the HHS wish to amend the terms of a Service Agreement, the Party wishing to amend the Service Agreement must give written notice of the proposed amendment to the other Party.

9.2 The process for amending this Service Agreement is set out in Schedule 5 of this Service Agreement.

10. Publication of amendments

The Department will publish each executed Deed of Amendment within 14 days of the date of execution on www.health.qld.gov.au/system-governance/health-system/managing/default.asp.

11. Cessation of service delivery

11.1 The HHS is required to deliver the Health Services and other services outlined in this Service Agreement for which funding is provided in Schedule 2. Any changes to service delivery must ensure maintenance of care and minimise disruptions to patients.

11.2 The Department and HHS may Terminate or temporarily Suspend a Health Service or other service by mutual agreement having regard to the following obligations:

(a) any proposed Termination or Suspension must be made in writing to the other Party;

(b) where it is proposed to Terminate or Suspend a Statewide Service, or a Regional Service, the HHSs which are in receipt of that service must also be consulted;

(c) the Parties must agree on a reasonable notice period following which Termination, or Suspension, will take effect; and

(d) patient needs, workforce implications, relevant government policy and HHS sustainability are to be considered.

11.3 The Department, in its role as the Queensland Public Health System manager:

(a) may, in its unfettered discretion, not support a requested Termination or Suspension and require the HHS to maintain the service; and

(b) will reallocate existing funding and activity for the Terminated or Suspended service inclusive of baseline Service Agreement funding and in-year growth funding on a pro-rata basis.

11.4 The HHS will:

(a) work with the Department to ensure continuity of care and a smooth transfer of the service to an alternative provider where this is necessary; and

(b) minimise any risk or inconvenience to patients associated with service Termination, Suspension or transfer.

11.5 In the event that a sustainable alternative provider cannot be identified, and this is required, the service and associated patient cohort will continue to remain the responsibility of the HHS.

12. Commencement of a new service

12.1 In the event that the HHS wishes to commence providing a new Health Service, the HHS will notify the Department in writing in advance of commencement.

12.2 The Department will provide a formal response regarding the proposed new Health Service to the HHS in writing. The Department may not agree to purchase the new Health Service or to provide funding on either a recurrent or non-recurrent basis.

12.3 In the event that a change to an established Referral Pathway is proposed which would result in the direction of patient referrals to an alternative HHS on a temporary or a permanent basis:

(a) the new Referral Pathway must be agreed by all impacted HHSs prior to its implementation; and

(b) following agreement of the new Referral Pathway, if there is an identifiable and agreed impact to funding the Department will redistribute funding and activity between HHSs in alignment with new Referral Pathway.

13. Provision of data to the Chief Executive

The HHS will provide to the Chief Executive the performance data and other data, including data pursuant to ad hoc requests, set out in Schedule 4 of this Service Agreement in accordance with the Schedule, including in relation to the form, manner and the times required for the provision of data.

14. Dispute resolution

14.1 The dispute resolution process set out below is designed to resolve disputes which may arise between the Parties to this Service Agreement in a final and binding manner.

14.2 These procedures and any disputes addressed or to be addressed by them are subject to the provisions of the *Hospital and Health Boards Act 2011*, including in respect of any directions issued under that legislation or by Government in respect of any dispute.

14.3 Resolution of disputes will be through a tiered process commencing with the Performance Review Meeting and culminating, if required, with the Minister, as illustrated in Figure 1.

14.4 Use of the dispute resolution process set out in this clause should only occur following the best endeavours of both Parties to agree a resolution to an issue at the local level. The dispute resolution process is not intended for the resolution of ongoing issues or performance related issues. At each stage of the dispute resolution process, the Parties agree to cooperate.

14.5 If a dispute arises in connection with this Service Agreement (including in respect of interpretation of the terms of this Service Agreement), then either Party may give the other a written Notice of Dispute.

14.6 The Notice of Dispute must be provided to the D-SA Contact Person if the notice is being given by the HHS and to the HHS-SA Contact Person if the notice is being given by the Department.

14.7 The Notice of Dispute must contain the following information:

(a) a summary of the matter in dispute;

(b) an explanation of how the Party giving the Notice of Dispute believes the dispute should be resolved and reasons to support that belief;

(c) any information or documents to support the Notice of Dispute; and

(d) a definition and explanation of any financial or Service delivery impact of the dispute.

Figure 1 Dispute resolution process

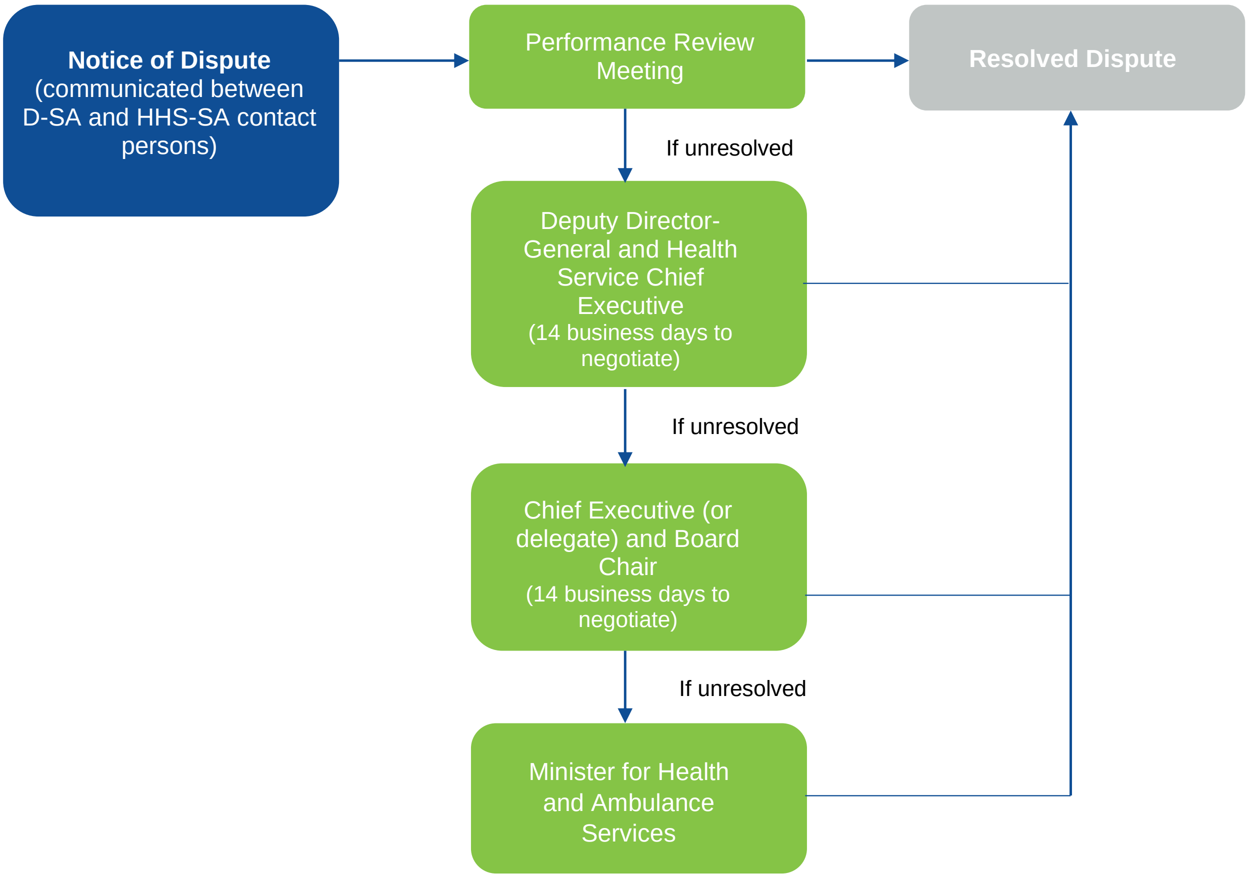

14.8 Resolution of a dispute

(a) Resolution of a dispute at any level is final. The resolution of the dispute is binding on the Parties but does not set a precedent to be adopted in similar disputes between other Parties.

(b) The Parties agree that each dispute (including the existence and contents of each Notice of Dispute) and any exchange of information or documents between the Parties in connection with the dispute is confidential and must not be disclosed to any third party without the prior written consent of the other Party, other than if required by law and only to the extent required by law.

14.9 Continued performance

Notwithstanding the existence of one or more disputes, the HHS must continue to perform and comply with this Service Agreement to the best of their abilities given the circumstances.

14.10 Disputes arising between Hospital and Health Services

(a) In the event of a dispute arising between two or more HHSs (an Inter-HHS Dispute), the process set out in Figure 2 will be initiated. Resolution of Inter-HHS Disputes will be through a tiered process, commencing with local resolution and culminating if required with formal and binding arbitration by the Minister under the provisions of the *Hospital and Health Boards Act 2011*, section 44.

(b) If the HHS wishes to escalate a dispute, the HHS will be expected to demonstrate that best endeavours (including Chair and Board involvement) to resolve the dispute between all parties at an informal and local level have taken place.

(c) Management of inter-HHS relationships should be informed by the following principles:

(i) HHSs should maintain (for both the base level of funding and growth) the proportion of out of HHS work undertaken unless as a result of agreed repatriation of patients.

(ii) All HHSs manage patients from their own catchment population if it is within their clinical capability to do so as specified by the Clinical Services Capability Framework.

(iii) Where it is proposed that a Health Service move from one HHS to another, agreement between the respective Health Service Chief Executives will be secured prior to any change in patient flows. Once agreed, funding will follow the patient.

(iv) All HHSs abide by the agreed dispute resolution process.

(v) All HHSs operate in a manner which is consistent with the health system principles and objectives as set out in the National Health Reform Agreement and the *Hospital and Health Boards Act 2011*.

Figure 2 Inter-HHS dispute resolution process

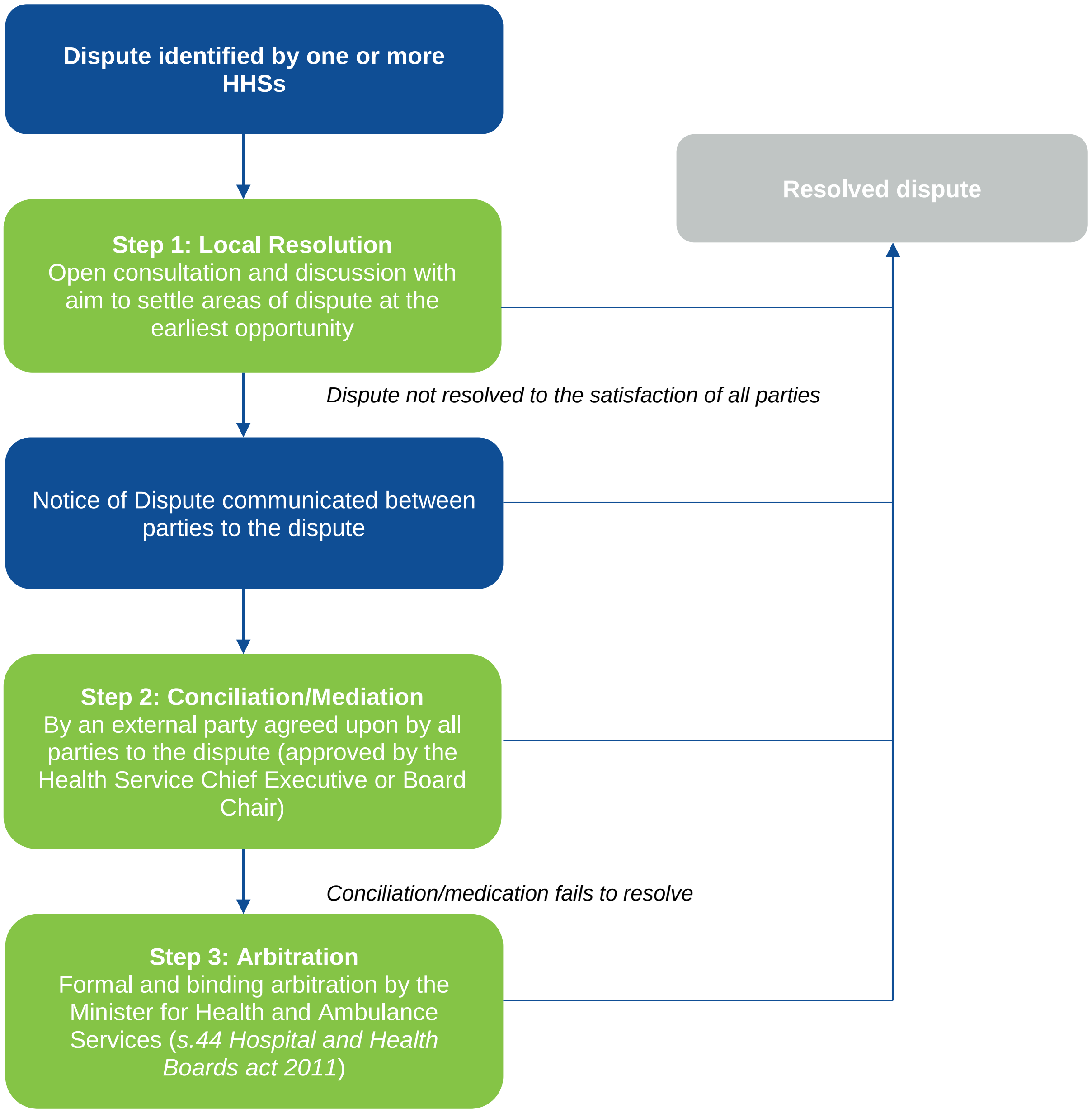

15. Force Majeure

15.1 If a Party (Affected Party) is prevented or hindered by Force Majeure from fully or partly complying with any obligation under this Service Agreement, that obligation may (subject to the terms of this Force Majeure clause) be suspended, provided that if the Affected Party wishes to claim the benefit of this Force Majeure clause, it must:

(a) give prompt written notice of the Force Majeure to the other Party of:

(i) the occurrence and nature of the Force Majeure;

(ii) the anticipated duration of the Force Majeure;

(iii) the effect the Force Majeure has had (if any) and the likely effect the Force Majeure will have on the performance of the Affected Party's

obligations under this Service Agreement; and

(iv) any disaster management plan that applies to the party in respect of the Force Majeure.

(b) use its best endeavours to resume fulfilling its obligations under this Service Agreement as promptly as possible; and

(c) give written notice to the other Party within five days of the cessation of the Force Majeure.

15.2 Without limiting any other powers, rights or remedies of the Chief Executive, if the Affected Party is the HHS and the delay caused by the Force Majeure continues for more than 14 days from the date that the Chief Executive determines that the Force Majeure commenced, the Chief Executive may give directions to the HHS regarding the HHS's performance or non-performance of this Service Agreement during the Force Majeure and the HHS must comply with that direction.

15.3 Neither Party may terminate this Service Agreement due to a Force Majeure event.

16. Hospital and Health Service accountabilities

16.1 Without limiting any other obligations of the HHS, it must comply with:

(a) the terms of this Service Agreement;

(b) all legislation applicable to the HHS, including the *Hospital and Health Boards Act 2011*;

(c) all Cabinet decisions applicable to the HHS;

(d) all Ministerial directives applicable to the HHS;

(e) all agreements entered into between the Queensland and Commonwealth governments applicable to the HHS;

(f) all regulations made under the *Hospital and Health Boards Act 2011*;

(g) all Industrial Instruments applicable to the HHS; and

(h) all health service directives applicable to the HHS.

16.2 The HHS will ensure that the accountabilities set out in Schedule 1 of this Service Agreement are met.

17. Department accountabilities

17.1 Without limiting any other obligations of the Department, it must comply with:

(a) the terms of this Service Agreement;

(b) the legislative requirements as set out within the *Hospital and Health Boards Act 2011*;

(c) all regulations made under the *Hospital and Health Boards Act 2011*; and

(d) all Cabinet decisions applicable to the Department.

17.2 The Department will work in collaboration with HHSs to ensure the Public Sector Health System delivers high quality hospital and other Health Services to persons in Queensland having regard to the principles and objectives of the national health system. In support of realising this objective, in accordance with section 5 of the *Hospital and Health Boards Act 2011* the Department will:

(a) provide overall management of the Queensland Public Sector Health System including health system planning, coordination and standard setting;

(b) provide the HHS with funding specified under Schedule 2 of this Service Agreement;

(c) provide and maintain payroll and rostering systems to the HHS unless agreed otherwise between the Parties;

(d) operate 13 HEALTH as a first point of contact for health advice with timely HHS advice and information where appropriate to local issues; and

(e) balance the benefits of a local and system-wide approach.

17.3 The Department will endeavour to purchase services in line with Clinical Prioritisation Criteria, where these have been developed, in order to improve equity of access and reflect the scope of publicly funded services.

17.4 The Department will maintain a public record of the Clinical Service Capability Framework levels for all public facilities based on the information provided by HHSs.

17.5 **Workforce management**

The Chief Executive agrees to appoint Health Service Employees to:

(a) perform work for the HHS for the purpose of enabling the HHS to perform its functions and exercise powers under the *Hospital and Health Boards Act 2011;* and

(b) deliver the services specified in this Service Agreement.

17.6 The Chief Executive, whether in the capacity of a Person Conducting a Business or Undertaking as an employer or as a building owner or occupier, will consult, cooperate and coordinate with the HHS to ensure legal compliance with the *Work Health and Safety Act 2011* and other legislation as it applies to the scope, nature and location of operations associated with this Service Agreement. This includes, but is not limited to:

(a) codes of practice;

(b) electrical safety legislation;

(c) building and fire safety legislation; and

(d) workers' compensation legislation.

18. Insurance

18.1 The HHS must hold and maintain for the period of this Service Agreement the types and levels of insurances that the HHS considers appropriate to cover its obligations under this Service Agreement.

18.2 Without limiting the types and levels of insurances that the HHS considers appropriate, any insurance policies taken out by the HHS under this clause must include appropriate coverage for the following:

(a) public and product liability insurance;

(b) professional indemnity insurance; and

(c) workers' compensation insurance in accordance with the *Workers' Compensation and Rehabilitation Act 2003* (Qld).

18.3 The HHS will be deemed to comply with its requirements under clauses 18.1 and 18.2(a) and 18.2(b) if it takes out and maintains a current insurance policy with the Queensland Government Insurance Fund.

18.4 Any insurance policies held by the HHS pursuant to this clause must be effected with an insurer that is authorised and licensed to operate in Australia.

18.5 The HHS must maintain a current register of all third-party guarantees.

18.6 The HHS must, if requested by the Department, promptly provide a sufficiently detailed certificate of currency and/or insurance and policy documents for each insurance policy held by the HHS pursuant to this clause.

18.7 The HHS warrants that any exclusions and deductibles that may be applicable under the insurance policies held pursuant to this clause will not impact on the HHS's ability to meet any claim, action or demand or otherwise prejudice the Department's rights under this Service Agreement.

18.8 The HHS must immediately advise the Department if any insurance policy, as required by this clause, is materially modified or cancelled.

19. Indemnity

19.1 The HHS indemnifies the Department against all and any liabilities, claims, actions, demands, costs and expenses made by any person which may be brought against or made upon or incurred by the Department arising directly or indirectly from or in connection with any of the following:

(a) any wilful, unlawful or negligent act or omission of the HHS, a Health Service Employee, Health Executive, Senior Health Service Employee or an officer, employee or agent working for the HHS in the course of the performance or attempted or purported performance of this Service Agreement;

(b) any penalty imposed for breach of any applicable law in relation to the HHS's performance of this Service Agreement; and

(c) a breach of this Service Agreement,

except to the extent that any act or omission by the Department caused or contributed to the liability, claim, action, demand, cost or expense.

19.2 The indemnity referred to in this clause will survive the expiration or termination of this Service Agreement.

20. Indemnity arrangements for officers, employees and agents

20.1 Indemnity arrangements for officers, employees or agents working for the Public Sector Health System are administered in accordance with the following policy documents, as amended from time to time:

(a) Indemnity for Queensland Health Medical Practitioners HR Policy I2 (QH-POL-153:2014); and

(b) Queensland Government Indemnity Guideline.

20.2 The costs of indemnity arrangements provided for Health Service Employees, Health Executives, Senior Health Service Employees, or officers, employees or agents working for the HHS are payable by the HHS.

21. Legal proceedings

21.1 This clause applies if there is any demand, claim, liability or legal proceeding relating to assets, contracts, agreements or instruments relating to the HHS, whether or not they are:

(a) transferred to an HHS under section 307 of the *Hospital and Health Boards Act 2011*; or

(b) retained by the Department.

21.2 Subject to any law, each party must (at its own cost) do all things, execute such documents and share such information in its possession and control that is relevant, and which is reasonably necessary, to enable the other party to institute or defend (as the case may be) any demand, claim, liability or legal proceeding.

22. Sub-contracting

22.1 The Parties acknowledge that the HHS may sub-contract the provision of Health Services and other services that are required to be performed by the HHS under this Service Agreement.

22.2 The HHS must ensure that any sub-contractor who has access to confidential information (as defined in section 139 of the *Hospital and Health Boards Act 2011*) and/or personal information (as defined in section 12 of the *Information Privacy Act 2009*) complies with obligations no less onerous than those imposed on the HHS.

22.3 The HHS agrees that the sub-contracting of services:

(a) will not transfer responsibility for provision of the services to the sub-contractor; and

(b) will not relieve the HHS from any of its liabilities or obligations under this Service Agreement, including but not limited to obligations concerning the provision of data in accordance with Schedule 4 (Data Supply Requirements).

23. Counterparts

23.1 This Service Agreement may be executed in two or more identical copy counterparts, each of which together will be deemed an original, but all of which together will constitute one and the same instrument.

23.2 In the event that any signature executing this Service Agreement or any part of this Service Agreement is delivered by facsimile transmission or by scanned e-mail delivery of a '.pdf' format data file or equivalent, the signature will create a valid and binding obligation of the Party executing (or on whose behalf the signature is executed) with the same force and effect as if the signature page were an original.

23.3 For execution under this clause 23 to be valid the entire Service Agreement upon execution by each individual Party must be delivered to the remaining parties.

Execution

A. The terms of this Service Agreement were agreed under the provisions set out in the *Hospital and* Health *Boards Act,* section 35 on 27 June 2019, and were subsequently amended by the Deed of Amendment entered into pursuant to section 39 of the *Hospital and Health Boards Act 2011* and executed on 10 January 2020; 15 May 2020; 1 June 2020; 25 June 2020 and 29 July 2020.

B. This revised Service Agreement consolidates amendments arising from:

- 2019/20 Amendment Window 2 (in-year variation);
- 2019/20 Amendment Window 3 (in-year variation);
- 2020/21 Amendment Window 1 (annual budget build);
- April 2020 Extra-ordinary Amendment Window; and
- May 2020 Extra-ordinary Amendment Window.

C. Execution source documents are available on the service agreement website https://www.health.qld.gov.au/system-governance/health-system/managing/agreements-deeds.

Schedule 1 HHS Accountabilities

1. Purpose

Without limiting any other obligations of the HHS, this Schedule 1 sets out the key accountabilities that the HHS is required to meet under the terms of this Service Agreement.

2. Registration, credentialing and scope of clinical practice

2.1 The HHS must ensure that:

(a) all persons (including off site reporting radiologists) who provide a clinical service for which there is a national or Queensland legal requirement for registration, have and maintain current registration throughout their employment and only practise within the scope of that registration;

(b) all persons who perform roles for which eligibility for membership of a professional association is a mandatory requirement, have and maintain current eligibility of membership of the relevant professional association throughout their employment in the role; and

(c) all persons who provide a clinical service, and who fall within the scope of current credentialing policies (i.e. including medical, dental, nursing, midwifery and allied health), have a current scope of clinical practice and practise within that scope of clinical practice (which includes practising within their registration conditions and within the scope of the Clinical Services Capability Framework of the facility/s at which the service is provided).

2.2 Confirmation of registration and/or professional memberships is to be undertaken in accordance with the processes outlined in 'Health Professionals Registration: medical officers, nurses, midwives and other health professionals HR Policy B14 (QH-POL-147:2016)', as amended from time to time.

3. Clinical Services Capability Framework

3.1 The HHS must ensure that:

(a) all facilities have undertaken a baseline self-assessment against the Clinical Services Capability Framework (version 3.2);

(b) the Department is notified when a change to the Clinical Services Capability Framework baseline self-assessment occurs through the established public hospital Clinical Services Capability Framework notification process; and

(c) in the event that a Clinical Services Capability Framework module is updated or a new module is introduced, a self-assessment is undertaken against the relevant module and submitted to the Department.

3.2 The HHS is accountable for attesting to the accuracy of the information contained in any Clinical Services Capability Framework self-assessment submitted to the Department.

4. Clinical Prioritisation Criteria

4.1 The HHS must ensure that:

(a) processes for access to specialist surgical and medical services in line with Clinical Prioritisation Criteria are implemented, where these have been developed, in order to improve equity of access to specialist services; and

(b) General Practice Liaison Officer and Business Practice Improvement Officer programs are maintained in order to deliver improved access to specialist outpatient services, including through (but not limited to) their contribution to the development and implementation of Statewide Clinical Prioritisation Criteria.

5. Service delivery

5.1 The HHS will work with collaboratively with other healthcare service providers to ensure that an integrated pathway of care is in place for patients. This will include, but is not limited to:

(a) other HHSs;

(b) Primary Care providers;

(c) non-government organisations; and

(d) private providers.

5.2 The HHS must ensure that:

(a) the Health Services and other outlined in this Service Agreement, for which funding is provided in Schedule 2 ‘Funding and Purchased Activity and Services’ continue to be provided;

(b) the obligations regarding the payment and planning for blood and blood products and best practice as set out under the National Blood Agreement are fulfilled for the facilities for which funding is provided; and

(c) the *Queensland Organ Donation Strategy 2018-2020* is implemented in order to support an increase in organ donation rates in Queensland.

5.3 Through accepting the funding levels defined in Schedule 2 of this Service Agreement, the HHS accepts responsibility for the delivery of the associated programs and reporting requirements to State and Commonwealth bodies as defined by the Department.

6. Accreditation

6.1 All Queensland public hospitals, day procedure services and health care centres (howsoever titled) managed within the framework of HHSs are to maintain accreditation under the Australian Health Service Safety and Quality Accreditation Scheme[1].

6.2 Accreditation will be assessed against the National Safety and Quality Health Service standards[2] (NSQHS) second edition.

6.3 Residential aged care facilities will maintain accreditation by the Aged Care Quality and Safety Commission (ACQSC).

6.4 General practices owned or managed by the HHS are to be externally accredited in accordance with the current edition of the Royal Australian College of General Practitioners (RACGP) accreditation standards and in line with the National General Practice Accreditation Scheme.

6.5 For the purpose of accreditation, the performance of the HHS against the NSQHS and the performance of general practices owned or managed by the HHS against the RACGP accreditation standards can only be assessed by accrediting agencies that are approved by the Australian Commission on Safety and Quality in Healthcare (ACSQHC).

6.6 The HHS will select their accrediting agency from among the approved accrediting agencies. The ACSQHC and the RACGP provide a list of approved accrediting agencies which are published on their respective websites (www.safetyandquality.gov.au and www.racgp.org.au.

6.7 If the HHS does not meet the NSQHS standards accreditation requirements, the HHS has 60 days to address any not met actions. If the HHS does not meet the other accreditation standards requirements (RACGP and ACQSC), a remediation period will be defined by the accrediting agency.

6.8 Following assessment against NSQHS, ACQSC and RACGP standards, the HHS will provide to the Executive Director, Patient Safety and Quality Improvement Service, Department.

(a) immediate advice if a significant patient risk (one where there is a high probability of a substantial and demonstrable adverse impact for patients) is identified during an onsite visit, also identifying the plan of action and timeframe to remedy the issue as negotiated between the surveyors/assessors and/or the respective accrediting agency and the HHS;

(b) a copy of any 'not met' reports within two days of receipt of the report by the HHS;

(c) the accreditation report within seven days of receipt of the report by the HHS; and

(d) immediate advice should any action be rated not-met by the accrediting agency following the remediation period of an accreditation event, resulting in the facility or service not being accredited. Responsive regulatory processes may be enacted under clause 7 below.

---

[1] www.safetyandquality.gov.au/our-work/assessment-to-the-nsqhs-standards/australian-health-service-safety-and-quality-accreditation-scheme/

[2] www.safetyandquality.gov.au/our-work/assessment-to-the-nsqhs-standards/

6.9 The award recognising that the facility or service has met the required accreditation standards will be issued by the assessing accrediting agency for the period determined by their respective accreditation scheme.

6.10 The HHS will apply to an approved accrediting agency for a re-accreditation assessment prior to the expiry of their current accreditation period.

6.11 Where the HHS funds non-government organisations to deliver health and human services the HHS will ensure, from the Effective Date of this Service Agreement, that:

(a) within 12 months HHS procurement processes and service agreements with contracted non-government organisations specify the quality accreditation requirements for mental health services as determined by the Department; and

(b) as the quality accreditation requirements for subsequent funded service types are determined by the Department, procurement processes and service agreements with contracted non-government organisations reflect these requirements within 12 months of their formal communication by the Department to HHSs.

7. Responsive regulatory process for accreditation

7.1 A responsive regulatory process is utilised in the following circumstances:

(a) where a significant patient risk is identified by a certified accrediting agency during an accreditation process; and/or

(b) where an HHS has failed to address ‘not met’ actions of the specified standards within required timeframes.

7.2 An initial regulatory response will begin with a process of verifying the scope, scale and implications of the reported issues, a review of documentation, and may include one or more site visits by nominated specialty experts.

7.3 The regulatory process may include one or a combination of the following actions:

(a) seek further information from the HHS;

(b) request a progress report for the implementation of an action plan;

(c) escalate non-compliance and/or risk to the Performance Review Meeting;

(d) provide advice, information on options or strategies that could be used to address the non-met actions within a designated time frame; and/or

(e) connect the hospital to other hospitals that have addressed similar deficits or have exemplar practice in this area.

7.4 In the case of serious or persistent non-compliance and where required action is not taken by the HHS the response may be escalated. The Department may undertake one or a combination of the following actions:

(a) restrict specified practices/activities in areas/units or services of the HHS where the specified standards have not been met;

(b) suspend particular services at the HHS until the area/s of concern are resolved; and

(c) suspend all service delivery at a facility within an HHS for a period of time.

8. Achieving health equity for First Nations Queenslanders

8.1 The *Queensland Health Statement of Action towards Closing the Gap in Health Outcomes* is a commitment to addressing systemic barriers that may in any way contribute to preventing the achievement of health equity for all First Nations people. The statement is expected to mobilise renewed efforts and prompt new strategies for achieving health equity for First Nations Queenslanders.

8.2 The HHS will develop a Health Equity Strategy (previously referred to as the Closing the Gap Health Plan) to demonstrate the HHS's activities towards achieving health equity for First Nations people. The Health Equity Strategy will supersede the existing Closing the Gap Health Plan and act as the principal accountability mechanism between community and Government in the pursuit of Health Equity for First Nations Queenslanders.

8.3 The Health Equity Strategy will:

(a) be co-designed, co-developed and co-implemented by the First Nations community and the HHS; and

(b) demonstrate an evidence-based approach to priority setting.

8.4 The HHS will review the Health Equity Strategy at least once every three years and will publish the Health Equity Strategy in a way that allows it to be accessed by members of the public.

8.5 In line with the *Queensland Health Statement of Action towards Closing the Gap in Health Outcomes,* the HHS will ensure that commitment and leadership is demonstrated through implementing actions outlined in the Health Equity Strategy. The actions will, at a minimum:

(a) promote and provide opportunities to embed the representation of First Nations people in leadership, governance and the workforce;

(b) improve local engagement and partnerships between the HHS and First Nations people, communities and organisations to enable co-design, co-development and co-implementation;

(c) improve transparency, reporting and accountability in Closing the Gap progress; and

(d) demonstrate co-design, co-development, co-implementation and co-leadership of health programs and strategies.

8.6 The HHS will:

(a) establish and maintain an ongoing dialogue with the Department to progress and report on key actions taken and Health Service initiatives aligned to the *Queensland Health Statement of Action towards Closing the Gap in Health Outcomes*;

(b) establish and maintain an ongoing dialogue with the Department to progress and report on key actions taken and health service initiatives and strategies to attract,

recruit, support and retain a First Nations people workforce and workforce models commensurate to the HHS population and aligned to the benchmarks prescribed in the Workforce Diversity and Inclusion Strategy 2017-2022; and

(c) report publicly on progress against the Health Equity Strategy. Progress will be reported on an annual basis as a minimum.

9. Provision of Clinical Products/Consumables in outpatient settings

9.1 Upon discharge as an inpatient or outpatient, and where products/consumables are provided free of charge or at a subsidised charge, the Treating HHS will bear the initial costs of products/consumables provided to the patient/consumer as part of their care. These costs will be met by the Treating HHS for a sufficient period of time to ensure the patient/consumer incurs no disruption to their access to the Clinical Products/Consumables.

9.2 Unless otherwise determined by the HHS providing the Clinical Products/Consumables, ongoing direct costs (beyond an initial period following discharge as an inpatient) of the provided products/consumables will be borne by the Residential HHS of the outpatient/consumer.

9.3 Where guidelines exist (e.g. Guideline for Compression Garments for Adults with Lymphoedema: Eligibility, Supply and Costing and Guideline for Home Enteral Nutrition Services for Outpatients: Eligibility, Supply and Costing), standardised eligibility criteria and charges should apply.

9.4 Where a patient is supplied with medicines on discharge, or consequent to an outpatient appointment, that are being introduced to a patient’s treatment, the Treating HHS will provide prescription(s) and an adequate initial supply. This will comprise:

(a) for medicines reimbursable under the Pharmaceutical Benefits Scheme (PBS), including the Section 100 Highly Specialised Drugs Program – the quantity that has been clinically-appropriately prescribed or the maximum PBS supply, whichever is the lesser; or

(b) for non-reimbursable medicines, one month’s supply or a complete course of treatment, whichever is the lesser.

9.5 For medicines that are non-reimbursable under the PBS, and which are not included in the Queensland Health List of Approved Medicines (LAM), the Residential HHS will be responsible for ongoing supply, provided that the Treating HHS has provided the Residential HHS with documentary evidence of the gatekeeping approval at the Treating HHS for the non-LAM medicine. This evidence may be:

(a) a copy of the individual patient approval; or

(b) where the medicine is subject to a ‘blanket approval’ at the Treating HHS, a copy of the blanket approval, and a statement that the patient meets the criteria to be included under that approval.

9.6 This evidence is to be provided pro-actively to the Director of Pharmacy (or, for non-Pharmacist sites, the Director of Nursing and the HHS Director of Pharmacy) for the hospital nominated under clause 9.8 below.

9.7 For non-reimbursable medicines listed on the LAM for the condition being treated, the Residential HHS is responsible for ongoing supplies.

9.8 The Treating HHS will inform the patient about the ongoing supply arrangements and agree which hospital, within the patient's Residential HHS, they should attend for repeat supplies. The patient will be advised to contact the pharmacy at the nominated hospital regarding their requirements at least a week before attending for repeat supply.

9.9 PBS-reimbursable prescriptions issued by a public hospital may be dispensed at any other public hospital that has the ability to claim reimbursement. Patients may, in accordance with hospital policy, be encouraged to have their PBS prescriptions dispensed at a private pharmacy of their choice.

10. Capital, land, buildings, equipment and maintenance

10.1 **Capital**

(a) The HHS will:

(i) achieve annual capital expenditure within an acceptable variance to its allocation in the State's published Budget Paper 3 – Capital Statement, as specified in the capital expenditure performance KPI target.

(ii) record capital expenditure data in the capital intelligence portal each month. Data will be published through the System Performance Reporting (SPR) platform.

(iii) achieve all Priority Capital and Health Technology Equipment Replacement Program capital expenditure requirements and associated delivery milestones, as funded, and undertake all capital expenditure performance reporting requirements in the capital intelligence portal on a monthly basis.

(iv) comply with all other capital program reporting requirements, as identified in Schedule 4, Table 13.

10.2 **Asset Management**

(a) The Service Agreement includes funding provision for regular maintenance of the HHS's building portfolio.

(b) The Department has determined that a total sustainable budget allocation that equates to a minimum of 2.81% of the un-depreciated asset replacement value of the Queensland Public Health System's building portfolio is required to sustain the building assets to achieve expected life-cycles. The sustainable budget allocation is a combination of operational and capital maintenance funding.

(c) The HHS will conduct a comprehensive assessment of the maintenance demand for the HHS's building portfolio to ascertain the total maintenance funding

requirements of that portfolio. The assessment must identify the following for the portfolio:

(i) regulatory requirements;

(ii) best practice requirements;

(iii) condition-based requirements;

(iv) lifecycle planning requirements; and

(v) reactive maintenance estimates based on historical information, including backlog maintenance liabilities and risk mitigation strategies.

(d) The HHS will allocate an annual maintenance budget that reasonably takes into account the maintenance demand identified by the assessment in its reasonable considerations, without limiting the scope of such reasonable considerations including financial affordability linked to risk assessment. The annual maintenance budget will equate to either:

(i) 2.81% of the un-depreciated asset replacement value of the HHS's building portfolio; or

(ii) an alternative percentage amount determined by the HHSs as a result of its considerations.

(e) The HHS will submit an annual asset management and maintenance plan, approved by the Health Service Chief Executive, to the Department that:

(i) outlines the maintenance demand assessment undertaken by the HHS under Schedule 1, clause 10.2(c)

(ii) confirms the annual maintenance budget determined by the HHS under Schedule 1, clause 10.2(d)

(f) The HHS will submit an annual Statement of Building Portfolio Compliance to the Department for each year of the Term of this Service Agreement.

(g) The HHS will continue to proactively develop and address the recommendations within the final Asset Management Capability Report that was issued to the HHS as part of the transfer notice process.

10.3 **Property**

(a) The HHS will ensure building and infrastructure assets are managed in accordance with the specifications of any relevant transfer notices published as a gazette notice by the Minister under section 273A of the *Hospital and Health Boards Act 2011.*

(b) For land, buildings and parts of buildings where the Department is, or is intended to be, the exclusive occupier under specific occupancy or ground leases implemented pursuant to clauses 1.7 (c) and 1.8 respectively (where applicable) of a transfer notice, the Department is deemed to be in control of that land, building or part of a building for the purpose of work health and safety law.

10.4 Nothing in clause 10.3(b) of Schedule 1:

(a) removes any work health and safety responsibilities shared with another party or parties in accordance with work health and safety law; or

(b) limits the arrangements for the provision of work health and safety services provided in clause 11.

11. Occupational health and safety

11.1 The HHS, whether in the capacity of a Person Conducting a Business or Undertaking as an employer or as a building owner or occupier, will consult, cooperate and coordinate to ensure legal compliance with the *Work Health and Safety Act 2011* and other legislation as it applies to the scope, nature and location of operations associated with this Service Agreement. This includes, but is not limited to:

(a) codes of practice;

(b) electrical safety legislation;

(c) building and fire safety legislation; and

(d) workers' compensation legislation.

11.2 The HHS will establish, implement and maintain a health and safety management system which conforms to recognised health and safety management system standard AS/NZS 4801 *Occupational Health and Safety Management System* or ISO45001 *Occupational Health and Safety Management Systems* or another standard as agreed with the Chief Executive.

11.3 The HHS will monitor health and safety performance and will provide to the Chief Executive reports of a type, and at the intervals, agreed between the Parties, or as reasonably specified by the Chief Executive.

11.4 The Chief Executive will monitor health and safety performance at the system level. Where significant health and safety risks are identified, or performance against targets is identified as being outside tolerable levels, the Chief Executive may request further information from the HHS to address the issue(s) and/or make recommendations for action.

12. Workforce management

12.1 Subject to a delegation by the Chief Executive under section 46 of the *Hospital and Health Boards Act 2011*, the HHS is responsible for the day-to-day management (the HR Management Functions) of the Health Service Employees provided by the Chief Executive to perform work for the HHS under this Service Agreement.

(a) The HHS will exercise its decision-making power in relation to all HR Management Functions which may be delegated to it by the Chief Executive under section 46 of the *Hospital and Health Boards Act 2011*, in respect of the Health Service Employees, in a lawful and reasonable manner and with due diligence, and in accordance with:

(i) terms and conditions of employment specified by the Department in accordance with section 66 of the *Hospital and Health Boards Act 2011;*

(ii) health service directives, issued by the Chief Executive under section 47 of the *Hospital and Health Boards Act 2011*;

(iii) health employment directives, issued by the Chief Executive under section 51A of the *Hospital and Health Boards Act 2011;*

(iv) any policy document that applies to the Health Service Employee;

(v) any Industrial Instrument that applies to the Health Service Employee;

(vi) the relevant HR delegations manual; and

(vii) any other relevant legislation.

12.2 The HHS must ensure that Health Service Employees are suitably qualified to perform their required functions.

12.3 Persons appointed in an HHS as a Health Executive or Senior Health Service Employees are employees of the HHS

12.4 All HHSs will provide to the Chief Executive human resource, workforce, and health and safety reports of a type, and at the intervals, agreed between the Parties, or as reasonably specified by the Chief Executive.

13. Medically authorised ambulance transports

13.1 The HHS will:

(a) utilise the Queensland Ambulance Service (QAS) for all road ambulance services not provided by the HHS. This includes both paramedic level and patient transport level services where the patient requires clinical care;

(b) follow the *Medically Authorised Ambulance Transports Operational Standards* when utilising QAS services; and

(c) ensure that performance data for ambulance services authorised by the HHS is collected and provided to the Department in line with agreed data supply requirements.

Schedule 2 Funding, purchased activity and services

1. Purpose

This Schedule 2 sets out:

(a) The activity purchased by the Department from the HHS (Table 4, Table 6 and Table 8);

(b) The funding provided for delivery of the purchased activity (Table 4; Table 5; Table 6; and Table 7);

(c) Specific funding commitments (Table 1);

(d) The criteria and processes for financial adjustments associated with the delivery of purchased activity and specific funding commitments;

(e) The sources of funding that this Service Agreement is based on and the manner in which these funds will be provided to the HHS (Table 3); and

(f) An overview of the purchased Health Services and other services which the HHS is required to provide throughout the period of this Service Agreement.

2. Delivery of purchased activity

2.1 The Department and the HHS will monitor actual activity against purchased levels.

2.2 The HHS has a responsibility to actively monitor variances from purchased activity levels and will notify the Department immediately via the D-SA Contact Person as soon as the HHS becomes aware of significant variances.

2.3 The HHS will also notify the Department of deliberate changes to the consistent recording of activity within year that would result in additional activity being recorded for existing Health Services.

2.4 If the HHS wishes to move activity between purchased activity types and levels, for example, activity moving from outpatients to inpatients or from one inpatient Service Related Group (SRG) to another, the HHS must negotiate this with the Department based on a sound needs based rationale.

2.5 With the exception of the programs, services and projects that are specified in Table 1, during 2020/21 no financial adjustment will be applied where the HHS is unable to deliver or exceeds the activity that has been funded, in recognition of the Commonwealth Government’s treatment of the National Health Reform Agreement to support the response to the COVID-19 pandemic.

2.6 The activity purchased through this Service Agreement for 2020/21 is based on the activity purchased recurrently in 2019/20 and includes the productivity dividend.

2.7 The activity purchased in the Service Agreement for 2021/22 will be based on the activity purchased recurrently in 2020/21 including the productivity dividend.

2.8 Any resulting changes to funding and purchased activity levels will be actioned through the processes outlined in Schedules 2 and 5 of this Service Agreement.

2.9 The Department is required to report HHS activity data to the Independent Hospital Pricing Authority and the Administrator of the National Health Funding Pool. The HHS will maintain its efforts to accurately record the delivered activity and submit this data to the Department in accordance with the requirements set out in Schedule 4.

2.10 The HHS should refer to the supporting document to this Service Agreement 'Healthcare Purchasing Policy and Funding Guidelines 2020/21' for details regarding the calculation of Weighted Activity Units. Supporting documents are available on-line as detailed in Appendix 1.

3. Financial adjustments

3.1 **Specific funding commitments**

(a) As part of the Service Agreement Value, the services, programs and projects set out in Table 1 have been purchased by the Department from the HHS. These services will be the focus of detailed monitoring by the Department.

(b) The HHS will promptly notify the D-SA Contact Person if the HHS forecasts an inability to achieve commitments linked to the specific funding commitments included in Table 1.

(c) On receipt of any notice under clause 3.1(b) of Schedule 2, it is at the discretion of the Chief Executive (or delegate) to withdraw allocated funding pro rata to the level of under delivery if the program is not being delivered according to the program objective or is not being delivered in full.

(d) If the Chief Executive (or delegate) decides to withdraw allocated funding, the Chief Executive (or delegate) will immediately issue an Adjustment Notice to the HHS-SA Contact Person confirming any adjustment that has been made in accordance with this clause 3.1 of Schedule 2. The Adjustment Notice will be issued through the process set out in clause 3.4 or 3.5 of Schedule 5.

(e) Following receipt of an Adjustment Notice under clause 3.1(d) of Schedule 2, the Parties will comply with the Adjustment Notice and immediately take steps necessary to give effect to the requirements of that Adjustment Notice.

(f) The Parties acknowledge that adjustments made under this clause 3.1 of Schedule 2 may vary the Service Agreement Value and/or a specific value recorded in Table 1.

(g) Where the Service Agreement Value and/or a specific value recorded in Table 1 is varied, the variation will be recorded in a Deed of Amendment issued following the next available Amendment Window.

Table 1 Specific Funding Commitments

Service/Program/Project	Funding	Activity	Timeframe	Conditions
BreastScreen	*$3,330,956*<br>*(comprises screening activity funding; Incentive funding; fixed allocations – e.g. Biomedical Technology Services, lease of premises)*<br><br>$3,473,565<br>(comprises screening activity funding; Incentive funding; fixed allocations – e.g. Biomedical Technology Services, lease of premised and 2.5% non-labour escalation)	*16,200 total screens*<br>*Incentive 1: 1,250 screens*<br>*Incentive 2: Not accepted by HHS*<br><br>16,200 total screens<br>Incentive 1: 1,350 screens<br>Incentive 2: Not accepted by HHS	*2019/20*<br><br>2020/21	Provision of BreastScreen services targeting women aged 50-74 years old (women 40-49 years and 75+ are also eligible, although not actively recruited).<br>Incentive funding:<br>• Incentive 1: $30 per screen for out of hours screens in the target age group 50 to 74, where BreastScreen Queensland (BSQ) Registry appointment time is before 8am on weekdays, 5pm and after on weekdays and all-day weekends; and<br>• Incentive 2: $90 per screen for 1st and 2nd screens in the target age group 50 to 74 above the 2015/16 BSQ Service specific baseline.<br>Note Incentive 1 and Incentive 2 activity is a subset of the total screening activity.<br>Funds may be withdrawn should the HHS not meet their screening target.
COVID-19 First Nations Response	$2,130,989	0	2020/21	The HHS will implement and deliver the required actions under the HHS First Nations COVID-19 response including the delivery of initiatives and outcomes outlined in memorandum C-ECTF-20-9652.<br>Funding is one-off in nature for discrete and time-limited activities that are directly attributable to managing the impacts of COVID-19 for First Nations peoples and must be in-scope under the existing financial guidelines for COVID-19 expenditure.<br>HHSs are to retain appropriate supporting documentation to substantiate all expenditure under the National Partnership Agreement.

Service/Program/Project	Funding	Activity	Timeframe	Conditions
Oral Health Services	$9,193,765 (recurrent)	177,746 WOOS	2020/21	Delivery consistent with the Oral Health Policy Framework.<br>Funding may be adjusted where the total oral health activity delivered varies from the purchased levels.<br>Oral health activity (WOOS) for the 0-15 year age group shall not be less than that achieved in 2017/18.<br>Oral health activity (WOOS) includes activity claimed under the Child Dental Benefits Schedule but excludes dental treatment delivered under general anaesthetic in public hospitals.
• National Partnership Agreement (NPA) on Adult Public Dental Services<br>• National Partnership Agreement (NPA) on Adult Public Dental Services – First Nations	$522,346<br><br>$102,976	9,497 WOOS<br><br>1872 WOOS	2020/21<br><br>2020/21	Queensland is required to meet two performance targets during 2020/21, which are the 30 September 2020 target (for 1 April to 30 September 2020) and the 31 March 2021 target (for 1 October 2020 to 31 March 2021). HHSs must collectively meet these targets.<br>Funding may be adjusted where the total oral health activity delivered varies from the purchased levels.
Hospital in the Home (HITH): Public Private Partnership (PPP)	*$1,925,000*<br><br>$1,925,000 (recurrent)	*397 WAUs (Q22)*<br><br>397 WAUs (Q22)	*2019/20*<br><br>2020/21	Funding provided to enable HITH services to continue under the current PPP agreement with BlueCare. Healthcare Improvement Unit will be working with the HHS and Central Procurement to develop a panel arrangement of providers for 2018 and beyond.
High risk foot patients seen/managed within 48 hours of referral to ambulatory services	$202,561 (recurrent)<br>$101,280 (recurrent)	*43 WAUs (Q21)*<br>*21 WAUs (Q22)*	*2018/19*<br>*2019/20*	The HHS will provide services as specified in the 2019/20 Ambulatory High-Risk Foot Services specification sheet published on QHEPS.

Service/Program/Project	Funding	Activity	Timeframe	Conditions
Safe and healthy drinking water in Indigenous Local Government areas	*$227,423*<br>$251,426<br>$206,525<br>$210,680<br>$215,411	*0*<br>0<br>0<br>0<br>0	*2019/20*<br>2020/21<br>2021/22<br>2022/23<br>2023/24	Funding is provided to work with Doomadgee Aboriginal Shire Council, Mornington Shire Council and Palm Island Aboriginal Shire Council to improve the capacity of water treatment plant operators such that these Councils can provide a safe and continuous supply of drinking water to their communities.<br>Once sustained improvement in the safety and continuity of the drinking water supply is achieved in the identified Council areas, capacity building should extend to the areas of waste water and solid waste.<br>The HHS will also assist Cairns and Hinterland HHS with the development of a culturally appropriate training package for Indigenous water treatment plant operators equivalent to the existing Certificate III in Water Operations.<br>Funding may only be used for the purposes of the program and the Department may recover or redirect unspent funding.
Evolve Therapeutic Services (ETS)	*$1,714,440*	*0*	*2019/20*	Provision of ETS within allocated resources and in line with the state-wide ETS Manual, noting variation in local contexts.<br>Reporting requirements as defined by the Mental Health, Alcohol and Other Drugs Branch.<br>If program performance requirements are not met in-year funding may be adjusted proportional to the under delivery against the agreed target.

Service/Program/Project	Funding	Activity	Timeframe	Conditions
Enterprise Bargaining (EB)	*$23,137,502*<br>$6,662,712<br>(comprises both recurrent and non-recurrent funding)	*0*<br>0	*2019/20*<br>2020/21	Funding has been allocated in full for the following EB agreements:<br>• *Nurses and Midwives (Queensland Health and Department of Education and Training) Certified Agreement (EB10) 2018* (Base wages and certain entitlements)*;* and<br>• *Medical Officers (Queensland Health) Certified Agreement (MOCA5) 2018.*<br>Legislative amendments have been introduced under the *Industrial Relations Act 2016* to give effect to a 2.5% increases under the following agreements as new agreements are yet to be certified:<br>• *Queensland Public Health Sector Certified Agreement (No.9) 2016;*<br>• *Queensland Health, Building, Engineering & Maintenance Services Certified Agreement (No.6) 2016;* and<br>• *Health Practitioners' and Dental Officers (Queensland Health) Certified agreement (No. 2) 2016.*<br>Funding which has been allocated recurrently in previous years has been recalled for the following streams as wage increase are not yet approved:<br>• HES-DSO;<br>• SES-SO; and<br>• VMO.<br>Subject to the terms and conditions of the agreements once executed a funding adjustment may be required. Full details can be found on the Budget and Analysis SharePoint platform.

Service/Program/Project	Funding	Activity	Timeframe	Conditions
Nurses and Midwives EB10 Innovation Fund:<br>• Enhanced General Practice Testing Initiative<br>• Midwifery Community Access Program<br>• Pregnancy on Palm Project	*$431,558*<br>$93,180	*0*<br>0	*2019/20*<br>2020/21	Funding has been provided under the *Nurses and Midwives (Queensland Health and Department of Education) Certified Agreement (EB10) 2018,* clause 44.6, Innovation Fund.<br>The HHS will deliver the project, evaluation and reporting as outlined in memos:<br>• C-ECTF-19/8745;<br>• C-ECTF-19/8748; and<br>• C-ECTF-19/8750.<br>Funding may be withdrawn if project requirements are not met.
Nurse Navigators	*$4,034,870*<br>$4,983,691<br>$4,983,691<br>$4,983,691 (recurrent)	*0*<br>0<br>0<br>0	*2019/20*<br>2020/21<br>2021/22<br>2022/23	The total Nurse Navigator Program allocation (2015/16 – 2019/20) is 28 NG7 and 2 NG8. All Nurse Navigator Program Full Time Equivalent (FTE) is required to be appointed to a position ID that has 'Nurse Navigator' within the position title.<br>The HHS is ineligible to appoint Nurse Navigator Program FTE to any pre-existing permanent positions which have been renamed to include 'Nurse Navigator' in the position title.<br>The HHS is required to report monthly on:<br>• Employed Nurse Navigator FTE;<br>• Number of Nurse Navigator plans in place; and<br>• Number of patients seen by Nurse Navigators.
Another 100 Midwives (Nursing)	*$964,122*<br>$988,992<br>$469,948	*0*<br>0<br>0	*2019/20*<br>2020/21<br>2021/22	The HHS will deliver the initiatives and outcomes outlined in the performance requirements as per memo C-ECTF-18/8074. Funding may be withdrawn if requirements are not met.
Strength with Immersion Model (SwIM)<br>• Critical Care Clinical Immersions	*$80,000*	*0*	*2019/20*	The HHS will deliver the initiatives and outcomes outlined in the performance requirements as per memo C-ECTF-19/10267.

Service/Program/Project	Funding	Activity	Timeframe	Conditions
Making Tracks towards closing the gap in health outcomes for Indigenous Queenslanders by 2033 - Investment Strategy 2018-21 including the *Queensland Aboriginal and Torres Strait Islander Rheumatic Heart Disease Action Plan 2018-2021*	*$2,899,118*<br>*(2,711,118 + $188,000*)*<br>$2,525,487	*0*<br>0	*2019/20*<br>2020/21	The HHS will deliver the initiatives and outcomes outlined in the performance requirements issued by the Aboriginal and Torres Strait Islander Health Division in memorandum C-ECTF-19/5767.<br>The HHS will implement and support the required actions under the *Queensland Aboriginal and Torres Strait Islander Rheumatic Heart Disease Action Plan 2018-2021* and outlined in the memorandum C-ECTF-19/5767.<br>Funding may be adjusted and/or unspent funds redirected or recovered where project performance requirements are not met.<br>* Funding of $188,000 for the Initiatives under the *Queensland Aboriginal and Torres Strait Islander Rheumatic Heart Disease Action Plan* deferred from 2018/19 to 2019/20.
North Queensland Aboriginal and Torres Strait Islander Sexually Transmissible Infections Action Plan 2016-2021	*$1,104,608*<br>$1,104,608	*0*<br>0	*2019/20*<br>2020/21	The HHS will implement and support the required actions under the *North Queensland Aboriginal and Torres Strait Islander Sexually Transmissible Infections Action Plan 2016-2021* (the Plan) including the delivery of initiatives and outcomes outlined in memorandum C-ECTF-19/5767.<br>Funding may be adjusted and/or unspent funds redirected or recovered where project performance requirements are not met.
• Contact Tracing Support Officer position	*$73,800*<br>$75,720	*0*<br>0	*2019/20*<br>2020/21	Progress with contact tracing is to be reported through the Plan.<br>If the position is not fully operational between 1 November 2018 and 30 June 2021, the HHS is to return unspent labour costs to the Communicable Diseases Branch.

Service/Program/Project	Funding	Activity	Timeframe	Conditions
Queensland Sexual Health Strategy 2016-2021	$137,591 (recurrent)	*0*	*2019/20*	Consistent with *the Queensland Sexual Health Strategy 2016-2021*, provide additional medical officer, advanced practice sexual health nursing or allied health professional hours to the Townsville Sexual Health Service to contribute to an improvement in the capacity to treat Sexually Transmissible Infections, Human Immunodeficiency Virus, viral hepatitis and provide psychological support to patients to enhance engagement in care and adherence with treatment.<br>This investment must increase the occasions of service, irrespective of position type.<br>An annual review of outcomes achieved against the success factors of the *Queensland Sexual Health Strategy* will be conducted by the Department. If program performance requirements are not met in-year funding may be withdrawn.
Community Mental Health Growth Allocation	$469,860 (recurrent)	*0*	*2018/19*	Provision of funding to employ 3.76 additional Full Time Equivalents (FTEs) in support of the HHSs initiatives to enhance the Townsville Older Persons Mental Health Service.<br>Recruitment of FTEs to be monitored by the Mental Health Alcohol and Other Drugs Branch on a regular basis using the Mental Health Establishment Collection, with adjustments to be made in-year if FTEs not all recruited permanently.

Service/Program/Project	Funding	Activity	Timeframe	Conditions
Syphilis in Pregnancy Guideline Implementation Project – Northern Queensland	*$200,000*<br>$55,000	*0*<br>0	*2019/20*<br>2020/21	This non-recurrent funding covers labour and non-labour costs of one or more temporary Clinical Nurse Consultant (NG7) positions to be hosted by Townsville Hospital, to undertake the project across the following Hospital and Health Services:<br>• Cairns and Hinterland;<br>• Central West;<br>• Central Queensland;<br>• Mackay;<br>• North West;<br>• Torres and Cape; and<br>• Townsville.<br>Project management will be provided by the Statewide Maternity and Neonatal Clinical Network, Clinical Excellence Queensland.<br>If the project funding is not fully expended by 30 June 2020, the HHS will return unspent labour and associated non-labour costs to the Communicable Diseases Branch.
Endovascular Clot Retrieval (ECR)	$3,540,000	0	2020/21	Recurrent funding to support the provision of ECR services in addition to the revenue received as Activity Based Funding. Funding may be withdrawn pro-rata is agreed service levels are not achieved in year.
Connecting Care to Recovery 2016-2021: A plan for Queensland's Mental Health<br>• Independent Patient Rights Advisors	$356,000 (recurrent)	*0*	*2017/18*	Independent Patient Rights Advisors are to be employed or engaged in accordance with the *Mental Health Act 2016* and the Chief Psychiatrist Policy on *Independent Patient Rights Advisors*.

Service/Program/Project	Funding	Activity	Timeframe	Conditions
Prisoner health services – revised base funding	$9,408,968 (recurrent)	*0*	*2019/20*	The HHS will use this funding to provide primary health care services for prisoners in:<br>• Townsville Correctional Centre – Men's; and<br>• Townsville Correctional Centre – Women's.<br>In accordance with the Memorandum of Understanding established between Queensland Health and Queensland Corrective Services.
Hospital Car Parking Concession Scheme	$40,000* (recurrent)	*0*	*2019/20*	If program performance requirements are not met in-year funding may be withdrawn.<br>Monthly reporting of car parking concessions data on 'Car Parking Concessions Reporting template' required.<br>Annual Hospital and Health Service Car Parking Concessions Criteria reporting required.<br>*Includes $5,000 to assist with administrative costs.
	$40,000	*0*	*2019/20*	Top up to 2019/20 funding. Includes $20,000 to assist with administrative costs.
	$20,000 (recurrent)	0	2020/21	Administrative support.
Residential aged care facility Support Services (RaSS)	*$690,000*<br>$590,000	*0*<br>0	*2019/20*<br>2020/21	The HHS will deliver the initiatives and outcomes as outlined in the RaSS and Hospital in The Home COVID-19 Funding - memorandum C-ECTF 20/4081.<br>The funding allocated is linked to the National Partnership Agreement on COVID-19 healthcare response and as such it will be a requirement of the HHS to capture accurate activity and expenditure of services delivered under this program. Unspent funds may be withdrawn and returned to the department.

Service/Program/Project	Funding	Activity	Timeframe	Conditions
Hospital in The Home (HITH)	*$190,000*<br><br>$380,000	*39 WAUs*<br><br>78 WAUs	*2019/20*<br><br>2020/21	The HHS will deliver the initiatives and outcomes as outlined in the Residential Aged Care Facility Support Services and HITH COVID-19 Funding - memorandum C-ECTF 20/4081.<br>The HHS will provide a reconciliation of expenditure through End of Year, and unspent funds may be withdrawn and returned to the department.
Care in the Right Setting (CaRS):<br>• Better Health North Queensland: Regional Healthcare in the Home<br>• State-wide Rural and Remote Supportive and Specialist Palliative Care Telehealth Service	*$761,500*<br>$3,300,000<br><br>*$445,811*<br>$724,124	*0*<br>0<br><br>*0*<br>0	*2019/20*<br>2020/21<br><br>*2019/20*<br>2020/21	Services will be provided consistent with the CaRS application(s).<br>If Service commencement does not align with the agreed implementation timeframes funding may be withdrawn on a pro-rata basis.<br>If the agreed Service levels are not provided, funding may be withdrawn.<br>Activity levels will be monitored regularly and cooperation with external evaluators is required.<br>Where this Service includes Service provision to another (receiving) HHS:<br>• If staffing is not available within the HHS to meet the agreed Service levels, the HHS will make alternate arrangements to ensure that the agreed Service levels are provided; and<br>• If the agreed Service levels are not provided, funding may be withdrawn and provided to the receiving HHS.

Service/Program/Project	Funding	Activity	Timeframe	Conditions
Rapid Results Program<br><br>*Delivering what matters: Networked Cardiac Services*	*$608,822*<br><br>$897,640 (recurrent)	*0*<br><br>0	*2019/20*<br><br>2020/21	Funding is provided for the provision of Networked Cardiac Services.<br>The HHS will:<br>• Meet the Service implementation timeframes and provide the Service levels established within the agreed business case;<br>• Provide Services to North West HHS as established within the agreed business case; and<br>• Utilise the Queensland Cardiac Outcomes Registry outreach data module for all data capture and reporting.<br>If Service commencement does not align with the agreed implementation timeframes funding may be withdrawn on a pro-rata basis.<br>If staffing is not available within the HHS to meet the agreed Service levels, the HHS will make alternate arrangements to ensure that the agreed Service levels are provided.<br>If the agreed Service levels are not provided, funding may be withdrawn and provided to North West HHS.<br>Activity levels will be monitored quarterly.
Rapid Results Program<br><br>*Delivering what matters: Advancing Kidney Care 2026 Collaborative*	$784,348 (recurrent)	*81 WAUs (Q22 part WAU backed)*<br><br>162 WAUs (Q22 fully WAU backed)	*2019/20*<br><br>2020/21	Funding is provided for implementation of a vascular access coordination model, kidney supportive care model and transplant coordination model under the *Advancing Kidney Care 2026 Collaborative.*<br>The HHS will ensure that the reporting requirements established for this initiative are met, including the provision of quarterly progress against agreed implementation milestones and outcome measures.

Service/Program/Project	Funding	Activity	Timeframe	Conditions
Rapid Results Program<br><br>*Delivering what matters: Frail and Older persons initiative*				
• RACF support services (RaSS)<br><br>• Geriatric Emergency Department Intervention (GEDI)	$600,00 (recurrent)<br><br>$600,00 (recurrent)	*0*<br><br>*0*	*2019/20*<br><br>*2019/20*	Funding is provided to implement the core principals of a Residential Aged Care Facility Acute Care Support Service and Geriatric Emergency Department Intervention through a hybrid model of care at Townsville Hospital from 1 July 2019.<br>The HHS will:<br>• Establish the service in line with agreed timelines;<br>• Establish a Steering Committee to provide oversight of the service development and operation;<br>• Comply with the agreed reporting requirements, including progress against the identified project outcomes and performance measures; and<br>• Participate in learning sessions and statewide working group meetings.<br>If these conditions are not met or if dedicated Frail Older Persons models of care are ceased, funding may be withdrawn.
Specialist Outpatient Strategy				Funding may be withdrawn if requirements are not met.
• Telehealth	*$615,000*<br><br>$615,000 (recurrent)	*126 WAUs (Q22)*<br><br>126 WAUs (Q22)	*2019/20*<br><br>2020/21	Funding is provided for Telehealth (tele-orthopaedics, paediatric medicine, neurosurgery, neurology, nephrology, oncology).
• General Practitioner with Special Interest (GPwSI)	*$55,460*	*0*	*2019/20*	The HHS will deliver the initiatives and outcomes outlined in memo C-ECTF-19/6469. Funding may be withdrawn if requirements are not met.

Service/Program/Project	Funding	Activity	Timeframe	Conditions
Planned Care Volume Targets – Elective Surgery	$95,223,016 (Funding in existing service agreement)	10,113 elective surgery separations aligned with the elective surgery data collection, as reported on SPR and any outsourced elective surgery activity.<br>19,646 WAUs (Q22).	2020/21	Funding has been quarantined to support the ongoing delivery of planned care volumes during 2020/21. Quarantined funds have been determined based on agreed average Q22 WAUs for 10,113 Elective Surgery Separations (volume target).<br>Volume targets to be reviewed and adjusted through the amendment window process to include any additional planned care investments.<br>Performance will be monitored quarterly with reconciliation of the agreed planned care activity targets (number of day case and overnight treated patients).<br>Should the agreed volume targets not be met, funding will be withdrawn at the calculated HHS average Q22 WAUs **per separation not delivered**:<br>Example:<br>1 Case = 1.94 Q22 WAUs or<br>$9,416 (QEP)<br>There will not be claw back of under delivery against Q22 WAUs target if the volume of elective day case and overnight separations has been delivered.

Service/Program/Project	Funding	Activity	Timeframe	Conditions
Planned Care Volume Targets – Gastrointestinal Endoscopy (GIE)	$11,800,298 (Funding in existing service agreement value)	4,974 Gastrointestinal Endoscopies aligned with the Gastrointestinal Endoscopy data collection, as reported on SPR and any outsourced GIE activity. 2,435 WAUs (Q22).	2020/21	Funding has been quarantined to support the ongoing delivery of planned care volumes during 2020/21. Quarantined funds have been determined based on agreed average Q22 WAUs for 4,974 Gastrointestinal Endoscopy Separations (volume target).<br>Volume targets to be reviewed and adjusted through the amendment window process to include any additional planned care investments.<br>Performance will be monitored quarterly with reconciliation of the agreed planned care activity targets (number treated GIE patients).<br>Should the agreed volume targets not be met, funding will be withdrawn at the calculated HHS average Q22 WAUs per separation not delivered:<br>Example:<br>1 Case = 0.49 Q22 WAUs or<br>$2,373 (QEP)<br>There will not be claw back of under delivery against Q22 WAUs target if the volume of GIE day case and overnight separations has been delivered.

Service/Program/Project	Funding	Activity	Timeframe	Conditions
Planned Care Volume Targets – Specialist Outpatients	$10,141,478 (Funding in existing service agreement value)	37,346 Specialist Outpatient initial service events as per the funding specification, and outsourced activity. 2,092 WAU (Q22).	2020/21	Funding has been quarantined to support the ongoing delivery of planned care volumes during 2020/21. Quarantined funds have been determined based on agreed average Q22 WAUs for 37,346 Initial Service Events (volume target).<br>Volume targets to be reviewed and adjusted through the amendment window process to include any additional planned care investments.<br>Performance will be monitored quarterly with reconciliation of the agreed planned care activity targets (number of initial service events).<br>Should the agreed volume targets not be met, funding will be withdrawn at the calculated HHS average Q22 WAUs per initial service event not delivered:<br>Example:<br>1 Case = 0.056 Q22 WAUs or<br>$272 (QEP)<br>There will not be claw back of under delivery against Q22 WAUs target if the volume of initial service events has been delivered.

3.2 **National Partnership on COVID-19 Response**

(a) The Department will provide additional funding to the HHS under the State Public Health Payment component of the *National Partnership on COVID-19 Response* where actual additional cost is demonstrated.

(b) Funding will be provided where the HHS has:

(i) undertaken activity that is in-scope for the State Public Health Payment during the reporting period; and

(ii) met the reporting requirements in relation to COVID-19 expenditure, as prescribed under the *National Partnership on COVID-19 Response* and by the Department.

(c) The scope of the State Public Health Payment is defined as:

(i) additional costs that are attributable to the treatment of patients with diagnosed or suspected COVID-19; or

(ii) additional costs of activities directed at preventing the spread of COVID-19.

(d) Additional costs that are reimbursed through the State Public Health Payment will be excluded from the calculation of activity eligible for funding under the terms of the National Health Reform Agreement.

(e) There is no guarantee that the HHS will have access to additional funding under the State Public Health Payment.

(f) All funding that is provided through the State Public Health Payment will be subject to a reconciliation to be undertaken by the Administrator of the National Health Funding Pool. The HHS will be required to provide evidence to support claims for additional costs. If the HHS is unable to provide the required evidence on request, funding received may be recalled subject to reconciliation.

(g) Funding adjustments will be actioned through the process set out in clause 3.4 of Schedule 5 of this Service Agreement.

3.3 **Financial adjustments – other**

(a) The purchasing approach includes a range of funding adjustments which aim to incentivise high value care, that is care which delivers the best outcomes at an efficient cost, and dis-incentivise Low Benefit Care. This includes incentive payments for HHS who achieve quality targets in specific areas of priority. The purchasing incentives that apply to this Service Agreement are detailed in Table 2.

(b) The Department must reconcile the applicable purchasing incentives in Table 2 in line with the timeframes specified in the purchasing specification sheet included within the supporting document 'Purchasing Policy and Funding Guidelines 2020/21'. The Department must promptly provide a copy of the reconciliation statement to the HHS-SA Contact Person.

(c) Funding adjustments must be based on the requirements contained in the relevant specification sheet for that purchasing incentive.

(d) If the Parties are unable to reach agreement in relation to any funding adjustments that are identified, the provisions of clause 14 in the standard terms of this Service Agreement will apply to resolve the dispute.

(e) When the Parties have agreed on a funding adjustment, the Chief Executive (or delegate) will issue an Adjustment Notice to the HHS-SA Contact Person confirming any funding adjustment that has been made in accordance with this clause 3.3 of Schedule 2. The Adjustment Notice will be issued through the process set out in clause 3.4(c) of Schedule 5.

(f) Following receipt of an Adjustment Notice under clause 3.4(c) of Schedule 5, the Parties will comply with the Adjustment Notice and immediately take the steps necessary to give effect to the requirements of that Adjustment Notice.

(g) The Parties acknowledge that the funding adjustments may vary the Service Agreement Value recorded in Schedule 2. Where the Service Agreement Value recorded in Schedule 2 is varied, the variation will be recorded in a Deed of Amendment issued following the next available Amendment Window.

Table 2 Purchasing Incentives 2020/21 (Summary)

Incentive	Description	Scope	Status for 2020/21	Funding Adjustment
Quality Improvement Payment (QIP) – Antenatal Visits for First Nations Women	Incentive payments for achieving targets for:<br>• First Nations women attending an antenatal session during their first trimester, and attending at least 5 antenatal visits; and<br>• First Nations women stopping smoking	All HHSs (excluding Children's Health Queensland)	Continues as per 2019/20 with new targets	50% advance payments made to HHSs with balance paid retrospectively based on performance.
Quality Improvement Payment (QIP) - Smoking Cessation (Community Mental Health)	Incentive payments for achieving targets for community mental health patients clinically supported onto the Smoking Cessation Clinical Pathway	All HHSs (excluding Children's Health Queensland and Mater Public Hospitals)	Continues as per 2019/20 with new targets	Paid retrospectively
High Cost Home Support Program	Payment for high cost 24 hour home ventilated patients meeting the eligibility criteria, where funding is not available through existing sources	All HHSs	Continues as per 2019/20	Paid retrospectively based on forecast costs
Telehealth	Incentive payments for additional outpatient activity volume, provision of telehealth consultancy for Inpatients, Emergency Department and Outpatients episodes and Store and Forward assessments	Inpatients, Emergency Department, Outpatients, and Store and Forward - all HHSs	Continues as per 2019/20 with Outpatients scope expanded to include rural and remote facilities across all HHSs	Paid retrospectively
Sentinel Events	Zero payment for national sentinel events	All ABF public hospitals	Continues as per 2019/20	Retrospective adjustment

3.4 Public and private activity/Own Source Revenue

(a) Own Source Revenue comprises Grants and Contributions, User Charges and Other Revenues.

(b) Where an HHS is above its Own Source Revenue target in respect of private patients, it will be able to retain the additional Own Source Revenue with no compensating adjustments to funding from other sources.

(c) Conversely where an HHS is below its Own Source Revenue target in respect of private patients, it will experience a reduction in revenue with no compensating adjustments to funding from other sources.

(d) The HHS will make all efforts to identify and maximise revenue from appropriate third parties to ensure optimisation of available State and Commonwealth funding across the Queensland Public Sector Health System.

(e) The Own Source Revenue identified in Table 3 is an estimate generated by the HHS which allows all third-party funding sources associated with service delivery

to be identified. The HHS will ensure that this estimate is substantiated and accurate to ensure no significant variances to actual revenue generated.

(f) The HHS will routinely revise and update the estimate to ensure alignment between the Service Agreement and Queensland Treasury's reporting system (TRIDATA).

(g) Budget adjustments for changes in Own Source Revenue from private patients will be actioned through the process set out in Schedule 5 of this Service Agreement.

4. Funding sources

4.1 The four main funding sources contributing to the HHS Service Agreement value are:

(a) Commonwealth funding;

(b) State funding;

(c) Grants and Contributions; and

(d) Own Source Revenue.

4.2 Table 3 provides a summary of the funding sources for the HHS and mirrors the total value of the Service Agreement included in Table 4.

Table 3 Hospital and Health Service funding sources 2020/21

Funding Source	Value ($)
**NHRA Funding**	
Activity Based Funding	758,274,983
Clinical Education and Training[3]	-29,041,023
Own Source Revenue contribution in ABF funded services	-49,562,670
**Pool Account – ABF Funding (State and Commonwealth)[4]**	**679,671,290**
Block Funding	137,747,643
Clinical Education and Training[3]	29,041,023
**State Managed Fund – Block Funding (State and Commonwealth)[5]**	**166,788,666**
**Locally Receipted Funds (Including Grants)**	**31,916,217**
**Locally Receipted Own Source Revenue (ABF)**	**49,562,670**
**Locally Receipted Own Source Revenue (Other activities)**	**29,738,014**
**Department of Health Funding[6]**	**121,767,793**
**Total NHRA Funding**	**1,079,444,650**
**NPA Covid-19 Response**	
Activity Based Funding	-
**Hospital Services Payment – ABF Funding (State and Commonwealth)[7]**	**-**
Block Funding	-
Clinical Education and Training[3]	-
**Hospital Services Payment – Block Funding (State and Commonwealth)[5]**	**-**
**Public Health Funding (State and Commonwealth) [8]**	**2,720,988**
**Total NPA – COVID-19 Funding**	**2,720,988**
**TOTAL**	**1,082,165,638**

[3] Clinical Education and Training (CET) is classified as Teaching, Training and Research Funding under the National Model and funded as a Block Funded Service. Under the State Model, CET is included as 'Other ABF' and forms part of the ABF total. To comply with the requirements of the National Health Reform Agreement, funding must be paid as it is received, therefore from a Funding Source perspective, CET has been reclassified to Block Funding.

[4] Pool Account - ABF Funding (State and Commonwealth) includes: Inpatient; Critical Care; Emergency Department; Sub and Non Acute; Mental Health; and Outpatient activities each allocated a proportion of Other ABF Adjustments.

[5] State Managed Fund - Block Funding (State and Commonwealth) includes: block funded hospitals; standalone specialist mental health hospitals; community mental health; and teaching, training and research.

[6] Department of Health Funding represents funding by the Department for items not covered by the National Health Reform Agreement including such items as: Prevention, Promotion and Protection; Depreciation, and other Health Services.

7 Hospital Services Payment - Funding provided under the COVID-19 National Partnership Agreement for activity that is attributable to the diagnosis and treatment of Medicare eligible patients with COVID-19 or suspected of having COVID-19; elective surgeries that have been rescheduled to free up public hospital capacity to respond to the COVID-19 outbreak; and may include activities related to the care of public patients being treated in private hospitals.

[8] Public Health Payment - Funding provided under the COVID-19 National Partnership Agreement for the State public health system's activity attributable to the treatment of patients with diagnosed or suspected COVID-19 or for activities directed at preventing the spread of COVID-19.

5. Funds disbursement

5.1 The Chief Executive will direct the disbursement of both State and Commonwealth funding from the State's National Health Funding Pool Sub Account and the State Managed Fund to the HHS. The Service Agreement and State level block payments to State managed funds from Commonwealth payments into the national funding pool are stated in Table 8.

5.2 However, the State (represented by the Chief Executive) will not:

(a) redirect Commonwealth payments between HHSs;

(b) redirect Commonwealth payments between funding streams (e.g. from ABF to Block Funding); and/or

(c) adjust the payment calculations underpinning the Commonwealth's funding.

5.3 Payment of Activity Based Funding and Block Funding to the HHS will be on a fortnightly basis.

5.4 Further information on the disbursement of funds is available in the supporting document to this Service Agreement 'Purchasing Policy and Funding Guidelines 2020/21'.

Table 4 HHS Finance and Activity Schedule 2019/20 – 2021/22 – Summary by Purchasing Hierarchy

	ABF/NABF	ABF_SPLIT	Service Stream	2019/20 QWAU (Q22)	2019/20 Funding ($) (Price: $4847)	2020/21 QWAU (Q22)	2020/21 Funding) (Price: $4847)	2021/22 QWAU (QTBC)	2021/22 Funding (Price: $TBC)
Allocations excluding COVID-19	ABF	ABF	Inpatients	79,917	$356,387,378	81,446	$359,921,932		
			Outpatients	17,397	$87,039,161	18,229	$91,630,406		
			Procedures & Interventions	13,662	$62,080,857	13,926	$62,486,227		
			Emergency Department	11,850	$53,648,946	12,065	$55,412,549		
			Sub & Non-Acute	10,193	$45,865,407	10,386	$46,208,542		
			Mental Health	7,853	$38,135,957	8,015	$38,129,014		
			Prevention & Primary Care	2,555	$13,048,950	2,471	$13,292,652		
			Other ABF $	0	$53,777,660	0	$55,215,569		
		**ABF Total**		**143,426**	**$709,984,316**	**146,540**	**$722,296,891**		
		ABF Other	CET Funding	0	$28,139,365	0	$29,240,922		
			Specified Grants	0	$6,581,495	0	$6,737,170		
			PPP	0	$0	0	$0		
			EB Quarantined	0	$6,896,022	0	$0		
		**ABF Other Total**		**0**	**$41,616,882**	**0**	**$35,978,093**		
	Other Funding	Other Funding	Block Funded Services	15,117	$108,290,598	15,551	$108,493,730		
			Population Based Community Services	0	$81,317,660	0	$80,894,049		
			Other Specific Funding	0	$123,748,540	0	$123,508,409		
			PY Services moved to ABF	0	$0	0	$0		
			Prevention Services – Public Health	0	$8,969,240	0	$8,273,478		
		**Other Funding Total**		**15,117**	**$322,326,038**	**15,551**	**$321,169,667**		
	**Allocations excluding COVID-19 TOTAL**			**158,543**	**$1,073,927,237**	**162,091**	**$1,079,444,650**		

	ABF/NABF	ABF_SPLIT	Service Stream	2019/20 QWAU (Q22)	2019/20 Funding ($) (Price: $4847)	2020/21 QWAU (Q22)	2020/21 Funding) (Price: $4847)	2021/22 QWAU (QTBC)	2021/22 Funding (Price: $TBC)
**COVID-19 related allocations**	**ABF**	ABF	Inpatients	39	$190,000	-0	$0		
			Outpatients	0	$725,000	0	$0		
			Procedures & Interventions	0	$0	0	$0		
			Emergency Department	0	$0	0	$0		
			Sub & Non-Acute	0	$0	0	$0		
			Mental Health	0	$0	0	$0		
			Prevention & Primary Care	0	$0	0	$0		
			Other ABF $	0	$0	0	$0		
		**ABF Total**		**39**	**$915,000**	**-0**	**$0**		
		ABF Other	CET Funding	0	$0	0	$0		
			Specified Grants	0	$0	0	$0		
			PPP	0	$0	0	$0		
			EB Quarantined	0	$0	0	$0		
		**ABF Other Total**		**0**	**$0**	**0**	**$0**		
	**Other Funding**	Other Funding	Block Funded Services	0	$690,000	0	$590,000		
			Population Based Community Services	0	$0	0	$0		
			Other Specific Funding	0	$1,828,586	0	$2,130,989		
			PY Services moved to ABF	0	$0	0	$0		
			Prevention Services – Public Health	0	$0	0	$0		
		**Other Funding Total**		**0**	**$2,518,586**	**0**	**$2,720,989**		
	**COVID-19 Allocations TOTAL**			**39**	**$3,433,586**	**-0**	**$2,720,989**		
**Grand Total**				**158,582**	**$1,077,360,823**	**162,091**	**$1,082,165,639**		

Table 5 Minor Capital and Equity

	2019/20 $	2020/21 $	2021/22 $
**Minor Capital & Equity**			
**Cash**			
SA 16-17.333 - Minor Capital funding Allocation 2016-17	$4,763,000	$4,763,000	
TVL-Apr17-05 BMRP Output to Equity Swap	$0	$0	
TVL-AW2-Oct17-16 NTFEP - Clarity Autoscan	$0	$0	
TVL-AW3-FEB20-10 Lease funding swap per changes to AASB16 - equity component	$512,542	$0	
TVL-BB2021-42 Lease funding swap per changes to AASB16 - equity component	$0	$483,904	
**Non-Cash**			
-	-	-	
**Grand Total**	**$5,275,542**	**$5,246,904**	

Table 6 HHS Finance and Activity Schedule 2019/20 – 2021/22 Other Funding Detail

Agreement		Service Stream	Category	2019/20 $	2020/21 $	2021/22 $
Allocations excluding COVID-19	Other Funding	Block Funded Services	Block Funded Services	$108,290,598	$108,493,730	
		**Block Funded Services Total**		**$108,290,598**	**$108,493,730**	
		Population Based Community Services	Alcohol, Tobacco & Other Drugs	$4,874,610	$4,192,132	
			Community Care Programs	$801,983	$814,528	
			Community Mental Health	$21,901,603	$22,626,119	
			Community Mental Health – Child & Youth	$12,361,977	$10,988,954	
			Other Community Services	$15,433,827	$16,160,007	
			Other Funding Subsidy/(Contribution)	$21,184,432	$21,184,432	
			Primary Health Care	$4,759,229	$4,927,877	
		**Population Based Community Services Total**		**$81,317,660**	**$80,894,049**	
		Other Specific Funding	Aged Care Assessment Program	$961,655	$721,241	
			Commercial Activities	-$0	-$0	
			Consumer Information Services	$0	$0	
			Depreciation	$62,534,000	$62,464,000	
			Disability Residential Care Services	$0	$0	
			Environmental Health	$13,617	$39,620	
			Home & Community Care (HACC) Program	$3,351,317	$3,351,317	
			Home & Community Medical Aids & Appliances	$315,761	$315,761	
			Home Care Packages	$0	$0	
			Interstate Patients	$3,141,971	$3,141,971	
			Multi-Purpose Health Services	$825,623	$825,623	
			Prisoner Health Services	$9,408,968	$9,408,968	
			Oral Health	$0	$0	
			Patient Transport	$6,513,634	$6,513,634	
			Research	$651,671	$651,671	
			Residential Aged Care	$25,576,351	$26,255,041	
			Specific Allocations	$8,334,977	$7,700,567	
			State-Wide Functions	$374,737	$374,737	
			Transition Care	$1,744,258	$1,744,258	
		**Other Specific Funding Total**		**$123,748,540**	**$123,508,409**	
		Prevention Services – Public Health	Environmental Health (PH)	$1,719,494	$1,424,701	
			Other Community Services (PH)	$7,249,746	$6,848,777	

Agreement		Service Stream	Category	2019/20 $	2020/21 $	2021/22 $
		Prevention Services – Public Health Total		$8,969,240	$8,273,478	
	Allocations excluding COVID-19 Other Funding Total			$322,326,038	$321,169,667	
COVID-19 related allocations	Other Funding	Block Funded Services	Block Funded Services	$690,000	$590,000	
		Block Funded Services Total		$690,000	$590,000	
		Population Based Community Services	Alcohol, Tobacco & Other Drugs	$0	$0	
			Community Care Programs	$0	$0	
			Community Mental Health	$0	$0	
			Community Mental Health – Child & Youth	$0	$0	
			Other Community Services	$0	$0	
			Other Funding Subsidy/(Contribution)	$0	$0	
			Primary Health Care	$0	$0	
		Population Based Community Services Total		$0	$0	
		Other Specific Funding	Aged Care Assessment Program	$0	$0	
			Commercial Activities	$0	$0	
			Consumer Information Services	$0	$0	
			Depreciation	$0	$0	
			Disability Residential Care Services	$0	$0	
			Environmental Health	$0	$0	
			Home & Community Care (HACC) Program	$0	$0	
			Home & Community Medical Aids & Appliances	$0	$0	
			Home Care Packages	$0	$0	
			Interstate Patients	$0	$0	
			Multi-Purpose Health Services	$0	$0	
			Prisoner Health Services	$0	$0	
			Oral Health	$0	$0	
			Patient Transport	$0	$0	
			Research	$0	$0	
			Residential Aged Care	$0	$0	
			Specific Allocations	$1,828,586	$2,130,989	
			State-Wide Functions	$0	$0	
			Transition Care	$0	$0	
		Other Specific Funding Total		$1,828,586	$2,130,989	
			Environmental Health (PH)	$0	$0	

Agreement		Service Stream	Category	2019/20 $	2020/21 $	2021/22 $
		Prevention Services – Public Health	Other Community Services (PH)	$0	$0	
		**Prevention Services – Public Health Total**		**$0**	**$0**	
	**COVID-19 Allocations Other Funding Total**			**$2,518,586**	**$2,720,989**	
**Grand Total**				**$324,844,624**	**$323,890,656**	

Table 7 Specified Grants

Program	2019/20 $	2020/21 $	2021/22 $
High Cost Outliers	$3,146,906	$3,165,142	
Limited Indication Medication Scheme	$600,642	$604,123	
Neonatal and paediatric retrieval	$0	$0	
Neonatal Retrieval Service	$1,620,000	$1,629,388	
Paediatric Retrieval Service	$1,100,000	$1,106,375	
PET Service	$0	$0	
18-19 Purch Initiatives (Final reconciliation) - Rewards	$115,536	$0	
18-19 Purch Initiatives (Final reconciliation) - Withdrawal	-$1,589	$0	
20-21 QIP - Antenatal care for Indigenous women	$0	$232,143	
**Grand Total**	**$6,581,495**	**$6,737,170**	

Table 8 Hospital and Health Service Service Agreement and State Level Block Payments to state managed funds from Commonwealth payments into national funding pool

State:	QLD	Service agreement for financial year:	2020/21
HHS	Townsville	Version for financial year:	
HHS ID		Version effective for payments from:	
		Version status:	07.07.2020

HHS ABF payment requirements:

Expected National Weighted Activity Unit (NWAU)		National efficient price (NEP) (as set by IHPA)
ABF Service group	Projected NWAU – N2021 (Draft)	
Admitted acute public services	80,283	$5,320
Admitted acute private services	4,823	$5,320
Emergency department services	11,904	$5,320
Non-admitted services	14,309	$5,320
Mental health services	6,078	$5,320
Sub-acute services	9,479	$5,320
**LHN ABF Total – excluding COVID-19**	**126,876**	**$5,320**
**LHN ABF Total – COVID-19 NPA**	**0**	**$5,320**

Note:

NWAU estimates do not take account of cross-border activity

Reporting requirements by HHS - total block funding paid (including Commonwealth) per HHS, as set out in Service Agreement:

Amount (Commonwealth and State) for each amount of block funding from state managed fund to LHN:	
**Block funding component**	**Estimated Commonwealth and state block funding contribution (ex GST)**
Block funded hospitals	$99,646,229
Community mental health services	$37,177,413
Teaching, Training and Research	$29,041,023
Home ventilation	$924,000
Other block funded services	$0
**Total block funding for LHN – excluding COVID-19**	**$166,788,666**
**Total funding for LHN under COVID-19 NPA State Public Health Payment**	**$2,720,989**

6. Purchased services

6.1 State-funded Outreach Services

(a) The HHS forms part of a referral network with other HHSs. Where state-funded Outreach Services are currently provided the HHS will deliver these Health Services in line with the following principles:

(i) historical agreements for the provision of Outreach Services will continue as agreed between HHSs;

(ii) funding will remain part of the providing HHS's funding base;

(iii) activity should be recorded at the HHS where the Health Service is being provided; and

(iv) the Department will purchase outreach activity based on the utilisation of the Activity Based Funding (ABF) price when Outreach Services are delivered in an ABF facility.

(b) Where new or expanded state-funded Outreach Services are developed the following principles will apply:

(i) the Department will purchase outreach activity based on the utilisation of the ABF price when Outreach Services are delivered in an ABF facility;

(ii) agreements between HHSs to purchase Outreach Services will be based on a cost recovery model, which will ensure providing sites are not financially disadvantaged and annual increases will be consistent with the ABF model;

(iii) any proposed expansion or commencement of Outreach Services will be negotiated between HHSs;

(iv) the HHS is able to purchase the Outreach Service from the most appropriate provider including private providers or other HHSs. However, when a change to existing Health Services is proposed, a transition period of at least 12 months will apply during which time the HHS will be required to continue to purchase Outreach Services from the HHS currently providing the Health Service;

(v) any changes to existing levels of Outreach Services need to be agreed to by both HHSs and any proposed realignment of funding should be communicated to the Department to ensure that any necessary funding changes are actioned as part of the Service Agreement amendment process and/or the annual negotiation of the Service Agreement Value; and

(vi) the activity should be recorded at the HHS where the Health Service is being provided.

(c) In the event of a disagreement regarding the continued provision of state-funded Outreach Services:

(i) any proposed cessation of Outreach Services will be negotiated between HHSs to mitigate any potential disadvantage or risks to either HHS; and

(ii) redistribution of funding will be agreed between the HHSs and communicated to the Department to action through the Service Agreement amendment processes outlined in Schedule 5 of this Service Agreement.

6.2 **Telehealth services**

(a) The HHS will support implementation of the Department Telehealth program, including the Telehealth Emergency Support Service. The HHS will collaborate with the Department, other HHSs, relevant non-government organisations and Primary Care stakeholders to contribute to an expanded network of Telehealth services to better enable a program of scheduled and unscheduled care.

(b) The HHS will ensure dedicated Telehealth Coordinators progress the Telehealth agenda locally, driving stakeholder engagement, adoption, planning and implementation activities that will support and grow Telehealth enabled services through substitution of existing face to face services and identification of new Telehealth enabled models of care.

(c) The HHS will ensure the Medical Telehealth Lead will collaborate with the network of HHS based Telehealth Coordinators and the Telehealth Support Unit to assist in driving promotion and adoption of Telehealth across the State through intra and cross-HHS clinician led engagement and change management initiatives as well as informing the development and implementation of clinical protocols and new Telehealth enabled models of care.

6.3 **Newborn hearing screening**

(a) In line with the National Framework for Neonatal Hearing Screening the HHS will:

(i) provide newborn hearing screening in all birthing hospitals and screening facilities; and

(ii) provide where applicable, co-ordination, diagnostic audiology, family support, and childhood hearing clinic services which meet the existing screening, audiology and medical protocols available from the Healthy Hearing website.

6.4 **Statewide Services**

The HHS has responsibility for the provision and/or coordination of the following Statewide Services. It is recommended that the HHS establish a Formal Agreement with the recipient HHSs regarding the roles and responsibilities of Statewide Service provision and receipt as described in the Definitions. In the event of a dispute regarding the provision of these services HHSs should refer to clause 14.9 of the main terms and conditions of this Service Agreement.

(a) **Neonatal Retrieval Services**

(i) The HHS is provided with a specified grant to support the statewide neonatal retrieval service referenced in Schedule 2. The HHS will ensure that the delivery and operation of neonatal retrieval services is consistent with the Governance and Operational Framework for statewide Paediatric and Neonatal Retrieval Services. This includes but is not limited to:

(A) contributing to statewide neonatal retrieval services with dedicated clinician resources to be available 24 hours a day, 7 days a week; and

(B) the provision of a dedicated, single point of neonatal medical coordination for Northern Queensland which operates on a 24 hours a day, 7 days a week basis.

(b) **Paediatric Retrieval Services**

(i) The HHS is provided with a specified grant to support the statewide paediatric retrieval service referenced in Schedule 2. The HHS will ensure that the delivery and operation of paediatric retrieval services is consistent with the Governance and Operational Framework for statewide Paediatric and Neonatal Retrieval Services. This includes but is not limited to:

(A) contributing to statewide paediatric retrieval services with dedicated clinician resources to be available 24 hours a day, 7 days a week; and

(B) supporting a dedicated, single point of paediatric medical coordination for Queensland provided by Children's Health Queensland HHS.

6.5 **Statewide and highly specialised clinical services**

The HHS will:

(a) participate in and contribute to the staged review of the purchasing model for identified Statewide and highly specialised clinical services; and

(b) collaborate with the Department and other HHSs in the development of Statewide Services Descriptions through the implementation of the Statewide Services Governance and Risk Management Framework. The Statewide Services Governance and Risk Management Framework guides the Department and HHSs in the strategic management, oversight and delivery of Statewide Services in order to optimise clinical safety and quality and ensure sustainability of services across Queensland.

6.6 **Regional Services**

The HHS has responsibility for the provision and/or coordination of the Regional Services listed below. It is recommended that the HHS establish a Formal Agreement with the recipient HHSs regarding the roles and responsibilities of Regional Service provision and receipt as described in the Definitions. In the event of a dispute regarding the provision of these services HHSs should refer to clause 14.9 of the main terms and conditions of this Service Agreement.

(a) **Aboriginal and Torres Strait Islander Child and Youth Care Coordination**

(i) Services to Cairns and Hinterland, Mackay, North West, Torres and Cape, and Townsville HHSs.

(b) **Basic Physician Training Pathway**

(i) The HHS will undertake the recruitment, selection, allocation and education of Queensland Basic Physician Pathway Trainees for the Far-North Rotation in conjunction with the Cairns and Hinterland HHS.

(ii) These activities will be undertaken in line with the state-wide Queensland Basic Physician Training Pathway model, supported by a Pathway Rotation Coordinator (Senior Medical Officer) and Pathway Project Officer, hosted in the HHS.

(c) **Community Forensic Outreach Service**

(i) Services to Mackay, North West, and Townsville HHSs.

(d) **Dual Disability Program**

(i) Services to Cairns and Hinterland, Mackay, North West, Torres and Cape, and Townsville HHSs.

(e) **Mental Health Clinical Improvement Team Program**

(i) Services to Cairns and Hinterland, Mackay, North West, Torres and Cape and Townsville HHSs.

(f) **North Queensland Forensic Adolescent Mental Health Services**

(i) Services to Cleveland Youth Detention Centre, Cairns and Hinterland, Mackay, North West, Torres and Cape, and Townsville HHSs.

6.7 **Rural and remote clinical support**

This clause does not apply to this HHS.

6.8 **Prevention Services, Primary Care and Community Health Services**

(a) The following funding arrangements will apply to the Prevention, Primary Care and Community Health Services delivered by the HHS:

(i) Department funding for Community Health Services. A pool of funding for these services is allocated to each HHS for a range of Community Health Services and must be used to meet local Primary Care and community healthcare and prevention needs including through delivery of the services identified in Table 6 and HHSs have the discretion to allocate funding across Primary Care and Community Health Services and Prevention Services according to local priorities.

(ii) Department specified funding models for consumer information services, disability, residential care, environmental health, prisoner health services, home and community medical aids, Primary Care, community mental health services, and alcohol and other drugs services. The funding specified for these programs is listed in Table 6 and Department Community Health Service grants.

(iii) Funding from other state government departments and the Commonwealth for specific programs (third party funded services).

(b) **Prevention Services**

The HHS will provide Prevention Services in line with public health related

legislation and the service delivery and reporting requirements outlined in the *Public Health Practice Manual*, including:

(i) **Specialist Public Health Units**

The HHS will:

(A) provide a specialist communicable disease epidemiology and surveillance, disease prevention and control service;

(B) maintain and improve, using a public health approach, the surveillance, prevention and control of notifiable conditions, including the prevention and control of invasive and exotic mosquitos, in accordance with national and/or State guidelines and ensure clinical and provisional notification of specified notifiable conditions are reported in accordance with the *Public Health Act* and Public Health Regulations;

(C) provide a specialist environmental health service, which includes assessment and coordination of local responses to local environmental health risks;

(D) undertake regulatory monitoring, investigation, enforcement and compliance activity on behalf of the Department;

(E) utilise specialist public health units to support the HHS through the provision of advice on prevention strategies and evidence; and

(F) provide specialist communicable disease epidemiology and surveillance, disease prevention and control and environmental health services to North West HHS. Where this includes visits to Mount Isa by Townsville-based Public Health Unit staff, office accommodation and related support will be made available for those staff during their visit.

(ii) **Preventive health services**

The HHS will:

(A) maintain delivery of risk factor prevention and early intervention programs and services targeting nutrition, physical activity, alcohol consumption, tobacco use, overweight and obesity and falls prevention;

(B) maintain delivery of the school-based youth nursing program throughout Queensland secondary schools; and

(C) promote brief interventions, lifestyle modification programs and other prevention, promotion or early intervention activities.

(iii) **Immunisation services**

The HHS will maintain or improve existing immunisation coverage through continuation of current immunisation services including:

(A) national immunisation program;

(B) opportunistic immunisation in healthcare facilities;

(C) special immunisation programs; and

(D) delivery of the annual school immunisation program in accordance with the Guideline for Immunisation Services (QH-GDL-955:2014).

(iv) **Sexually transmissible infections including HIV and viral hepatitis**

The HHS will maintain and improve, using a public health approach, the prevention, testing, treatment and contact tracing of blood borne viruses and sexually transmissible infections with a continued focus on relevant identified target populations such as First Nations peopleand culturally and linguistically diverse populations through Services including, but not limited to:

(A) public health units;

(B) sexual health services;

(C) infectious diseases services;

(D) viral hepatitis services;

(E) syphilis surveillance services;

(F) needle and syringe programs; and

(G) existing clinical outreach and support programs in place between HHSs.

(v) **Tuberculosis services**

(A) The HHS will ensure there is no financial barrier for any person to tuberculosis diagnostic and management services and will ensure that services are available in accordance with the Tuberculosis Control health service directive (qh-hsd-040:2018) and Protocol (qh-hsdptl-040-1:2018).

(B) The Townsville Tuberculosis Control Unit will provide the following services to Mackay, North West and Townsville HHSs:

- patient assessment and management of mycobacterial diseases;
- outpatient services onsite and clinics offsite;
- clinics as required at private health facilities, educational facilities and public areas and workplaces;
- screening services; and
- BCG vaccination services.

(vi) **Public Health Events of State Significance**

The HHS will comply with the *Declaration and Management of a Public Health Event of State Significance* health service directive (qh-hsd-

046:2014).

(vii) **Cancer screening services**

The HHS will:

(A) increase cervical screening rates for women in rural and remote areas and refer clients to relevant preventive health programs such as BreastScreen Queensland, QUIT line, Get Healthy and My Health for Life by maintaining the existing Mobile Women's Health Service;

(B) provide the Department with an annual report detailing the services provided by the Mobile Women's Health Service:

- across Townsville HHS excluding Cardwell; and
- within Mackay HHS – Collinsville and Bowen only;

(C) maintain the existing Healthy Women's Initiative in accordance with the Principles of Practice, Standards and Guidelines for Providers of Cervical Screening Services for First Nations women and national cervical screening policy documents;

(D) ensure that all cervical screening services provided by the HHS are delivered in accordance with the National Competencies for Cervical Screening Providers and national cervical screening policy documents; and

(E) provide timely, appropriate, high quality and safe follow-up diagnostic services within the HHS for National Cervical Screening Program participants in accordance with the *National Cervical Screening Program Guidelines for the Management of Screen-detected Abnormalities, Screening in Specific Populations and Investigation of Abnormal Vaginal Bleeding* (2017) and national cervical screening policy documents.

(F) develop, implement and evaluate a plan to increase participation in bowel cancer screening and provide the Department with an evaluation report at the end of 2021/22 for the following catchment:

- Townsville HHS; and
- North West HHS;

(G) provide timely, appropriate, high quality and safe diagnostic assessment services for National Bowel Cancer Screening Program participants in accordance with the National Health and Medical Research Council's *Clinical Guidelines for Prevention, Early Detection and Management of Colorectal Cancer* (2017). Services to be provided:

- across Townsville HHS.

(H) develop and implement a local service management plan to

increase participation in and guide the delivery of accessible breast screening for women in the target age group (50–74 years) through a BreastScreen Australia accredited service. The screening and assessment services should be delivered in accordance with the BreastScreen Queensland (BSQ) Quality Standards Protocols and Procedures Manual, BreastScreen Australia National Accreditation Standards and national policies. Services to be provided:

- across Townsville HHS excluding Tully SA2;
- within North West HHS excluding Carpentaria SA2; and
- within Mackay HHS for the Bowen and Collinsville SA2s only;

(I) allow the use of the HHS BSQ mobile asset by other HHSs during periods where practical to maximise utilisation of BSQ mobile fleet;

(J) continue to negotiate reciprocal utilisation of BSQ mobile assets with the Cairns and Hinterland HHS;

(K) schedule screening services through provision of BSQ mobile vans to increase accessibility for women living in rural and remote areas. While screening schedules are ideally finalised by HHSs six months in advance, confirmation of mobile sites is required by the BreastScreen Queensland Registry eight weeks prior to commencement at each site to ensure invitations for screening are prepared and distributed to women in the catchment area; and

(L) develop and implement infrastructure plans to manage asset lifecycle performance and replacement schedules including mobile vans. The repair and maintenance services for the BSQ mobile service fleet will be managed and administered by the Mobile Dental Clinic Workshop in Metro South HHS. The HHS will notify the Mobile Dental Clinic Workshop of any repair and maintenance issues and liaise with the Mobile Dental Clinic Workshop to arrange scheduled servicing. The Mobile Dental Clinic Workshop will meet the costs for these services subject to availability of allocated funding for this purpose in any given financial year.

6.9 **Oral health services**

The HHS will ensure that:

(a) oral health services are provided to the Eligible Population at no cost to the patient[9] and that the current range of clinical services will continue;

---

[9] The HHS may provide oral health services on a fee-for-service basis to non-eligible patients in rural and remote areas where private dental services are not available.

(b) oral health services fulfil the relevant obligations related to Commonwealth Government dental funding program/s;

(c) service delivery is consistent with Queensland Health's oral health policy framework; and

(d) the repair, maintenance and relocation service for the mobile dental fleet continues to be provided by the Mobile Dental Clinic Workshop in Metro South HHS.

6.10 **Prisoner health services**

(a) The HHS will:

(i) provide health Services for prisoners consistent with the principles, responsibilities and requirements specified in the Memorandum of Understanding (Prisoner Health Services) between Queensland Health and Queensland Corrective Services;

(ii) provide the Department with an annual report regarding the provision of these Services; and

(iii) establish local collaborative arrangements with Queensland Corrective Services to improve the health and well-being of prisoners and to contribute to the safe operation of the correctional centre.

(b) The HHS will provide health Services for people accommodated in the Cleveland Youth Detention Centre consistent with health Services available to youth in the wider community.

6.11 **Refugee health**

(a) The HHS will implement a health service for refugees, special humanitarian entrants and asylum seekers which provides:

(i) standard initial health assessments, including catch-up vaccination;

(ii) coordination of short-term health management; and

(iii) supported referral to existing services for continuing care, in particular to general practitioners to conduct the medical component of the refugee health assessment.

(b) This service will be operated out of the Townsville Clinic.

6.12 **Adult sexual health clinical forensic examinations**

(a) The HHS will:

(i) provide 24-hour access to clinical forensic examinations for adult victims of sexual assault who present at a public hospital; and

(ii) provide the Department with a quarterly report on the number of examinations provided.

(b) The Service provided will be consistent with the principles of the Queensland Government inter-agency guidelines for responding to people who have

experienced sexual assault and any standards issued pursuant to a Health Service Directive.

7. Teaching training and research

The HHS will provide the teaching, training and research programs for which funding is provided within Schedule 2 of this Service Agreement and as described below:

7.1 **Clinical education and training**

(a) The HHS will:

(i) continue to support and align with the current Student Placement Deed Framework which governs clinical placements from relevant tertiary education providers in Queensland HHS facilities;

(ii) comply with the obligations and responsibilities of Queensland Health under the Student Placement Deed, as appropriate, as operator of the facility at which the student placement is taking place;

(iii) comply with the terms and conditions of students from Australian education providers participating in the Student Placement Deed Framework;

(iv) only accept clinical placements of students from Australian education provides participating in the Student Placement Deed Framework;

(v) continue to provide training placements consistent with and proportionate to the capacity of the HHS. This includes, but is not limited to, planning and resourcing for clinical placement offers in collaboration with other HHSs and the Department, and the provision of placements for the following professional groups relevant to the HHS:

(A) medical students

(B) nursing and midwifery students

(C) pre-entry clinical allied health students

(D) interns

(E) rural generalist trainees

(F) vocational medical trainees

(G) first year nurses and midwives

(H) re-entry to professional register nursing and midwifery candidates

(I) dental students

(J) allied health rural generalist training positions

(K) Aboriginal and Torres Strait Islander Health Workers and Aboriginal and Torres Strait Islander Health Practitioners

(vi) participate in vocational medical rotational training schemes, facilitate the

movement of vocational trainees between HHSs and work collaboratively across HHSs to support education and training program outcomes;

(vii) report, at the intervals and in the format agreed between the Parties, to the Department on the pre-entry clinical placements provided under the Student Placement Deed Framework;

(viii) comply with the state-wide vocational medical training pathway models including:

(A) The Queensland Basic Physician Training Network;

(B) The Queensland General Medicine Advanced Training Network;

(C) The Queensland Intensive Care Training Pathway;

(D) The Queensland Basic Paediatric Training Network;

(E) The Queensland General Paediatric Advanced Training Network; and

(F) The Queensland Neonatal and Perinatal Medicine Advanced Training Network;

(ix) support the provision of placements by the Queensland Physiotherapy Placement Collaborative for physiotherapy pre-entry students via the Physiotherapy Pre-registration Clinical Placement Agreement;

(x) provide clinical area placements for dietetics pre-entry students from additional funding provided through relevant agreements with Universities; and

(xi) oversight profession specific (Clinical Measurement Sciences) and inter-professional statewide allied health clinical education programs.

(b) In addition, the Health Practitioners and Dental Officers (Queensland Health) Certified Agreement (No 2) 2016 (the HP agreement) requires Hospital and Health Services to:

(i) continue to support development of allied health research capacity through continued implementation and retention of health practitioner research positions provided through the HP agreement; and

(ii) support development of allied health clinical education capacity through continued implementation and retention of clinical educator positions provided through the HP agreement, continuing to provide allied health pre-entry clinical placements and maintaining support for allied health HP 3 to 4 rural development pathway positions.

(c) The HHS will maintain or increase its contribution of staff to the Queensland Country Relieving doctors program and the receiving HHS will be responsible for wages, clinical governance and appropriate supervision of the junior medical relievers.

7.2 **Health and medical research**

The HHS will:

(a) Articulate an investment strategy for research (including research targets and Performance Measures) which integrates with the clinical environment to improve clinical outcomes;

(b) Develop mechanisms for completing administrative governance for the approval of research in line with a national benchmark of 25 days (Standard Operating Procedures for Queensland Health Research Governance Officers 2013);

(c) Develop mechanisms for monitoring site research activity in line with jurisdictional commitments and National Health and Medical Research Council Guidelines (*Framework for* Monitoring *Guidance for the national approach to single ethical review of multi-centre research, January 2012*); and

(d) Develop systems to capture research and development expenditure and revenue data and associated information on research.

Schedule 3 Performance Measures

1. Purpose

This Schedule 3 outlines the Performance Measures that apply to the HHS.

2. Performance Measures

2.1 The Performance and Accountability Framework uses Performance Measures to monitor the extent to which the HHS is delivering the high-level objectives set out in this Service Agreement.

2.2 Each Performance Measure is identified under one of four categories:

(a) Safety and Quality Markers which together provide timely and transparent information on the safety and quality of services provided by the HHS;

(b) Key Performance Indicators (KPIs) which are focused on the delivery of key strategic objectives and statewide targets. KPI performance will inform HHS performance assessments;

(c) Outcome Indicators which provide information on specific activities and interventions where there is a reasonable expectation or evidence that they make a positive contribution towards improving the health status and experience of patients; and

(d) supporting indicators which provide contextual information and enable an improved understanding of performance, facilitate benchmarking of performance across HHSs and provide intelligence on potential future areas of focus. Supporting indicators are available on-line as referenced in Appendix 1 to this Service Agreement.

2.3 The HHS should refer to the relevant attribute sheet for each Performance Measure for full details. These are available on-line as referenced in Appendix 1 to this Service Agreement.

2.4 The Performance Measures identified in Table 9; Table 10 and Table 11 are applicable to the HHS unless otherwise specified within the attribute sheet.

2.5 The HHS will meet the target for each KPI identified in Table 9 as specified in the attribute sheet.

2.6 The Performance Measures identified in italic text are for future development.

2.7 Further information on the performance assessment process is provided in the supporting document to this Service Agreement, Performance and Accountability Framework 2020/21 referenced at Appendix 1 to this Service Agreement.

Table 9 HHS Performance Measures – Key Performance Indicators

Key Performance Indicators	
**Safe**	
The health and welfare of service users is paramount	
• Minimise risk	• Avoid harm from care
• Transparency and openness	• Learn from mistakes
**Title**	
Hospital Acquired Complications	
Emergency length of stay:<br>• % of Emergency Department attendances who are admitted as an inpatient, including to a short stay unit, and whose Emergency Department length of stay is within 4 hours	
Number of Emergency Department stays greater than 24 hours	
Emergency Department wait time by triage category	
Rate of face to face community follow up within 1-7 days of discharge from an acute mental health inpatient unit	
**Timely**	
Care is provided within an appropriate timeframe	
• Treatment within clinically recommended time	
**Title**	
Patient off stretcher time:<br>• % of patients transferred from Queensland Ambulance Service into the Emergency Department within 30 minutes	
Elective surgery:<br>• % of category 1 patients treated within the clinically recommended time	
Elective surgery:<br>• Number of ready for care patients waiting longer than the clinically recommended timeframe for their category	
Specialist outpatients:<br>• % of category 1 patients who receive their initial specialist outpatient appointment within the clinically recommended time	
Specialist outpatients:<br>• Number of ready for care category 1 patients waiting longer than clinically recommended for their initial specialist outpatient appointment	
Gastrointestinal endoscopy:<br>• % of category 4 patients who are treated within the clinically recommended time	
Gastrointestinal endoscopy:<br>• Number of patients waiting longer than clinically recommended timeframe for their category	
Access to oral health services:<br>• % of patients on the general care dental wait list waiting for less than the clinically recommended time	

**Equitable**	
Consumers have access to healthcare that is responsive to need and addresses health inequalities	
• Fair access based on need	• Addresses inequalities
**Title**	
Potentially Preventable Hospitalisations – First Nations People	
Telehealth utilisation rates:<br>• Number of non-admitted telehealth service events	
**Efficient**	
Available resources are maximised to deliver sustainable, high quality healthcare	
• Avoid waste	• Minimise financial risk
• Sustainable/productive	• Maximise available resources
**Title**	
Forecast operating position:<br>• Full year<br>• Year to date	
Average sustainable Queensland Health FTE	
Capital expenditure performance	
**Patient Centred**	
Providing Healthcare that is respectful of and responsive to individual patient preferences, needs and values	
• Patient involved in care	• Patient feedback
• Respects patient/person values and preferences	• Care close to home
**Title**	
Proportion of mental health service episodes with a documented care plan	
Proportion of beds vacated by 11am	

Table 10 HHS Performance Measures - Safety and Quality Markers

**Safety and Quality Markers**	
**Safe**	
The health and welfare of service users is paramount	
• Minimise risk<br>• Transparency and openness	• Avoid harm from care<br>• Learn from mistakes
**Title**	
Sentinel Events:<br>• Number of wholly preventable sentinel events	
Hospital Standardised Mortality Ratio	
Healthcare-associated Staphylococcus Aureus (including MRSA) bacteraemia:<br>• Rate per 10,000 patient days	
Severity Assessment Code (SAC) closure rates:<br>• % of incidents closed within the prescribed timeframe	
Unplanned Readmission Rates	

Table 11 HHS Performance Measures – Outcome Indicators

Outcome Indicators	
**Safe**	
**The health and welfare of service users is paramount**	
• Minimise risk<br>• Transparency and openness	• Avoid harm from care<br>• Learn from mistakes
**Title**	
Rate of seclusion events per 1,000 acute mental health admitted patient days	
Rate of absent without approval from acute mental health inpatient care	
**Timely**	
Care is provided within an appropriate timeframe	
• Treatment within clinically recommended time	
**Title**	
Reperfusion therapy for acute ischaemic stroke:<br>• Proportion of patients treated with either IV thrombolytic drugs or endovascular clot retrieval	
Access to emergency dental care:<br>• % of emergency courses of care for adult dental patients that commence within the recommended waiting times	
**Equitable**	
Consumers have access to healthcare that is responsive to need and addresses health inequalities	
• Fair access based on need	• Addresses inequalities
**Title**	
First Nations people representation in the workforce:<br>• % of the workforce who identify as being First Nations people	
Completed general courses of oral health care for First Nations people adult patients	
Low birthweight:<br>• % of low birthweight babies born to Queensland mothers	
**Patient Centred**	
Providing Healthcare that is respectful of and responsive to individual patient preferences, needs and values	
• Patient involved in care	• Patient feedback
• Respects patient/person values and preferences	• Care close to home
**Title**	
Complaints resolved within 35 calendar days	
Advance care planning:<br>• The proportion of approaches made to people who are identified as being at risk of dying within the next 12 months, or suitable for an advance care planning discussion, and who are offered the opportunity to consider, discuss and decide their preferences for care at the end of life	

Effective	
Healthcare that delivers the best achievable outcomes through evidence-based practice	
• Evidence based practice	• Care integration
• Treatment directed to those who benefit	• Optimise Health
• Clinical Capability	

**Title**
Uptake of the smoking cessation clinical pathway for public hospital inpatients and dental clients
Potentially Preventable Hospitalisations – diabetes complications:<br>• The number and proportion of hospitalisations of people with Diabetes complications that could have potentially been prevented through the provision of appropriate non-hospital health services.
Potentially Preventable Hospitalisations – non-diabetes complications:<br>• The number and proportion of hospitalisations of people with non-Diabetes complications that could have potentially been prevented through the provision of appropriate non-hospital health services.
% of oral health activity which is preventive
Cardiac rehabilitation:<br>• Proportion of public cardiac patients that are referred to cardiac rehabilitation and complete a timely patient journey
Adolescent vaccinations administered via the statewide School Immunisation Program

Schedule 4 Data Supply Requirements

1. Purpose

1.1 *The Hospital and Health Boards Act 2011*[10] (s.16(1)(d)) provides that the Service Agreement will state the performance data and other data to be provided by an HHS to the Chief Executive, including how, and how often, the data is to be provided.

1.2 This Schedule 4 specifies the data to be provided by the HHS to the Chief Executive and the requirements for the provision of the data.

2. Principles

2.1 The following principles guide the collection, storage, transfer and disposal of data:

(a) trustworthy – data is accurate, relevant, timely, available and secure;

(b) private – personal information is protected in accordance with the law;

(c) valued – data is a core strategic asset;

(d) managed – collection of data is actively planned, managed and compliant; and

(e) quality – data provided is complete, consistent, undergoes regular validation and is of sufficient quality to enable the purposes outlined in clause 3.2 of this Schedule 4 to be fulfilled.

2.2 The Parties agree to constructively review the data supply requirements as set out in this Schedule 4 on an ongoing basis in order to:

(a) ensure data supply requirements are able to be fulfilled; and

(b) minimise regulatory burden.

3. Roles and responsibilities

3.1 **Hospital and Health Services**

(a) The HHS will:

(i) provide, including the form and manner and at the times specified, the data specified in the data supply requirements (Attachment A to this Schedule 4) in accordance with this Schedule 4;

(ii) provide data in accordance with the provisions of the *Hospital and Health Boards Act 2011, Public Health Act 2005* and *Private Health Facilities Act 1999;*

[10] Section 143(2)(a) of the *Hospital and Health Boards Act 2011* provides that the disclosure of confidential information (as defined in s.139 of the Act) to the Chief Executive by an HHS under a service agreement is a disclosure permitted by an Act.

(iii) provide other HHSs with routine access to data, that is not Patient Identifiable Data, for the purposes of benchmarking and performance improvement;

(iv) provide data as required to facilitate reporting against the Performance Measures set out in Schedule 3 of this Service Agreement;

(v) provide data as specified within the provision of a health service directive;

(vi) provide activity data that complies with the national data provision timeframes required under the Independent Hospital Pricing Authority (IHPA) data plan for Commonwealth funding. Details of the timeframes are specified in the 'Commonwealth Efficient Growth Funding and National Weighted Activity Units (NWAUs)' specification sheet included in the supporting document Purchasing Policy and Funding Guidelines 2020/21 and the clinical placement data supply requirements; and

(vii) as requested by the Chief Executive from time to time, provide to the Chief Executive data, whether or not specified in this Schedule 4 or the Service Agreement, as specified by the Chief Executive in writing to the HHS in the form and manner and at the times specified by the Chief Executive.

(b) Data that is capable of identifying patients will only be disclosed as permitted by, and in accordance with, the *Hospital and Health Boards Act 2011, Public Health Act 2005 and the Private Health Facilities Act 1999.*

3.2 **Department**

The Department will:

(a) produce a monthly performance report which includes:

(i) actual activity compared with purchased activity levels;

(ii) any variance(s) from purchased activity;

(iii) performance information as required by the Department to demonstrate HHS performance against the Performance Measures specified in Schedule 3 of this Service Agreement; and

(iv) performance information as required by the Department to demonstrate the achievement of commitments linked to specifically allocated funding included in Schedule 2 of this Service Agreement.

(b) utilise the data sets provided for a range of purposes including:

(i) to fulfil legislative requirements;

(ii) to deliver accountabilities to state and commonwealth governments;

(iii) to monitor and promote improvements in the safety and quality of Health Services;

(iv) to support clinical innovation; and

(c) advise the HHS of any updates to data supply requirements as they occur.

Attachment A Data Supply Requirements

The HHS should refer to the relevant minimum data set for full details. These are available on-line as referenced in Appendix 1.

Table 12 Clinical data

Data Set	Data Custodian
Aged Care Assessment Team data via the Aged Care Evaluation (ACE) database	Strategic Policy Unit
Alcohol Tobacco and Other Drug Treatment Services	Mental Health Alcohol and Other Drugs Branch
Alcohol and Other Drugs Establishment Collection	Mental Health Alcohol and Other Drugs Branch
Allied Health Clinical Placement Activity Data	Allied Health Professions Office of Queensland
Australian and New Zealand Intensive Care Society (ANZICS) Data Collection	Healthcare Improvement Unit
BreastScreening Clinical Data	Executive Director, Preventive Health Branch
Clinical Incident Data Set	Patient Safety and Quality Improvement Service
Clinical Placement Data (excluding Allied Health)	Workforce Strategy Branch
Consumer Feedback Data Set	Patient Safety and Quality Improvement Service
Elective Surgery Data Collection	Healthcare Improvement Unit
Emergency Data Collection	Healthcare Improvement Unit
Gastrointestinal Endoscopy Data Collection	Healthcare Improvement Unit
Hand Hygiene Compliance Data	Communicable Diseases Branch
Healthcare Infection Surveillance Data	Communicable Diseases Branch
Maternal Deaths	Queensland Maternal and Perinatal Quality Council (through Statistical Services Branch)
Mental Health Act Data	Mental Health Alcohol and Other Drugs Branch
Mental Health Activity Data Collection	Mental Health Alcohol and Other Drugs Branch
Mental Health Carer Experience Survey Collection	Mental Health Alcohol and Other Drugs Branch
Mental Health Establishments Collection	Mental Health Alcohol and Other Drugs Branch
Monthly Activity Collection (including admitted and non-admitted patient activity and bed availability data)	Statistical Services Branch
Newborn Hearing Screening	Children's Health Queensland
Notifications Data	Chief Health Officer
Patient Experience Survey Data	Patient Safety and Quality Improvement Service
Patient Level Costing and Funding Data	HHS Funding and Costing Unit
Perinatal Data Collection	Statistical Services Branch
Queensland Bedside Audit	Patient Safety and Quality Improvement Service
Queensland Health Non-Admitted Patient Data Collection	Statistical Services Branch
Queensland Hospital Admitted Patient Data Collection	Statistical Services Branch
Queensland Needle and Syringe Program (QNSP) data	Chief Health Officer
Queensland Opioid Treatment Program Admissions and Discharges	Chief Health Officer
Radiation Therapy Data Collection	Healthcare Improvement Unit

Data Set	Data Custodian
Residential Mental Health Care Collections	Mental Health Alcohol and Other Drugs Branch
Schedule 8 Dispensing data	Chief Health Officer
School Immunisation Program – Annual Outcome Report	Communicable Diseases Branch
Specialist Outpatient Data Collection	Healthcare Improvement Unit
National Notifiable Diseases Surveillance System	Chief Health Officer
Vaccination Administration data	Chief Health Officer
Variable Life Adjusted Display (VLAD) CM (collection of hospital investigations)	Patient Safety and Quality Improvement Service
Your Experience of Service (YES) Survey Collection (Mental Health)	Mental Health Alcohol and Other Drugs Branch

Table 13 Non-clinical data

Non-Clinical Data Set	Data Custodian
Asbestos management data	Capital and Asset Services Branch
Asset Management<br>• Planning<br>• Maintenance<br>• Maintenance Budget<br>• Statement of Building Portfolio Compliance<br>• Benchmarking & Performance Data	Capital and Asset Services Branch
Conduct and Performance Excellence (CaPE)	Human Resources Branch
Expenditure	Finance Branch
Financial and Residential Activity Collection (FRAC)	Statistical Services Branch
Graduate Nursing Recruitment Data Statewide using the Public Service Commission Graduate Portal System	Office of the Chief Nursing and Midwifery Officer
Hospital Car Parks (including Government Portfolio Model funding arrangements)	Capital and Asset Services Branch
Minimum Obligatory Human Resource Information (MOHRI)	Finance Branch
Minor Capital Funding Program expenditure & forecast data	Finance Branch
Recruitment Data	Human Resources Branch
Revenue	Finance Branch
Queensland Health Workforce & Work Health & Safety Data	Human Resources Branch
Queensland Integrated Safety Information Project (QISIP)Solution Minimum Data Set	Human Resources Branch
Statewide employment matters	Human Resources Branch
Sustaining Capital Reporting Requirements (other than minor capital)	Capital and Asset Services Branch
Whole of Government Asset Management Policies data	Capital and Asset Services Branch

Schedule 5 Amendments to this Service Agreement

1. Purpose

This Schedule 5 sets out the mechanisms through which this Service Agreement may be amended during its term, consistent with the requirements of the *Hospital and Health Boards Act 2011.*

2. Principles

2.1 It is acknowledged that the primary mechanism through which HHS funding adjustments are made is through the budget build process that is undertaken annually in advance of the commencement of the financial year. This approach is intended to provide clarity, certainty and transparency in relation to funding allocations.

2.2 Amendments to the clauses of this Service Agreement should be progressed for consideration as part of the annual budget build process.

2.3 It is recognised that there is a requirement to vary funding and activity in-year. The following principles will guide amendments and amendment processes:

(a) funding allocations to HHSs should occur as early as possible within a financial year if unable to be finalised in advance of a given financial year;

(b) the number of Amendment Windows each year should be minimised to reduce the administrative burden on HHSs and the Department;

(c) Amendment Proposals should be minimised wherever possible and should always be of a material nature;

(d) Amendment Windows 2 and 3 are not intended to include funding or activity variations that could have been anticipated in advance of the financial year;

(e) Amendment Windows are intended to provide a formal mechanism to transact funding or activity variations in response to emerging priorities;

(f) Extraordinary Amendment Windows are not intended to be routinely used.

2.4 The Department remains committed to the ongoing simplification and streamlining of amendment processes.

3. Process to amend this Service Agreement

3.1 The Parties recognise the following mechanisms through which an amendment to this Service Agreement can be made:

(a) Amendment Windows;

(b) Extraordinary Amendment Windows;

(c) periodic adjustments; and

(d) end of year financial adjustments.

3.2 Amendment Windows

(a) In order for the Department to manage amendments across all HHS Service Agreements and their effect on the delivery of Public Sector Health Services in Queensland, proposals to amend this Service Agreement will be negotiated and finalised during set periods of time during the year (Amendment Windows).

(b) Amendment Windows are the primary mechanism through which amendments to this Service Agreement are made.

(c) Amendment Windows occur three times within a given financial year:

(i) Amendment Window 1: Annual Budget Build;

(ii) Amendment Window 2: In-year variation; and

(iii) Amendment Window 3: In-year variation.

(d) A Party that wants to amend the terms of this Service Agreement must give an Amendment Proposal to the other party.

(e) While a Party may submit an Amendment Proposal at any time, an Amendment Proposal will only be formally negotiated and resolved during one of the Amendment Windows outlined in Table 14 (excluding Extraordinary Amendment Windows).

Table 14 Amendment Window Exchange Dates

Amendment Window	Exchange Date	Primary Focus
Amendment Window 2: In-year variation	4 October 2019	2019/20 in-year variations
Amendment Window 3: In-year variation	14 February 2020	2019/20 in-year variations
Amendment Window 1: Annual Budget Build	27 March 2020	2020/21 budget build
Amendment Window 2: In-year variation	9 October 2020	2020/21 in-year variations
Amendment Window 3: In-year variation	12 February 2021	2020/21 in-year variations
Amendment Window 1: Annual Budget Build	26 March 2021	2021/22 budget build
Amendment Window 2: In-year variation	8 October 2021	2021/22 in-year variations
Amendment Window 3: In-year variation	11 February 2022	2021/22 in-year variations

(f) An Amendment Proposal is made by:

(i) the responsible Deputy Director-General signing and providing an Amendment Proposal to the HHS-SA Contact Person through Healthcare Purchasing and System Performance Division prior to the commencement of any Amendment Window; or

(ii) the Health Service Chief Executive signing and providing an Amendment Proposal to the D-SA Contact Person prior to the commencement of any Amendment Window.

(g) A Party giving an Amendment Proposal must provide the other Party with the following information:

(i) the rationale for the proposed amendment;

(ii) the precise drafting for the proposed amendment;

(iii) any information and documents relevant to the proposed amendment; and

(iv) details and explanation of any financial, activity or service delivery impact of the amendment.

(h) Negotiation and resolution of Amendment Proposals will occur during the Negotiation Period through a tiered process, as outlined in Figure 3.

Figure 3 Amendment Proposal negotiation and resolution

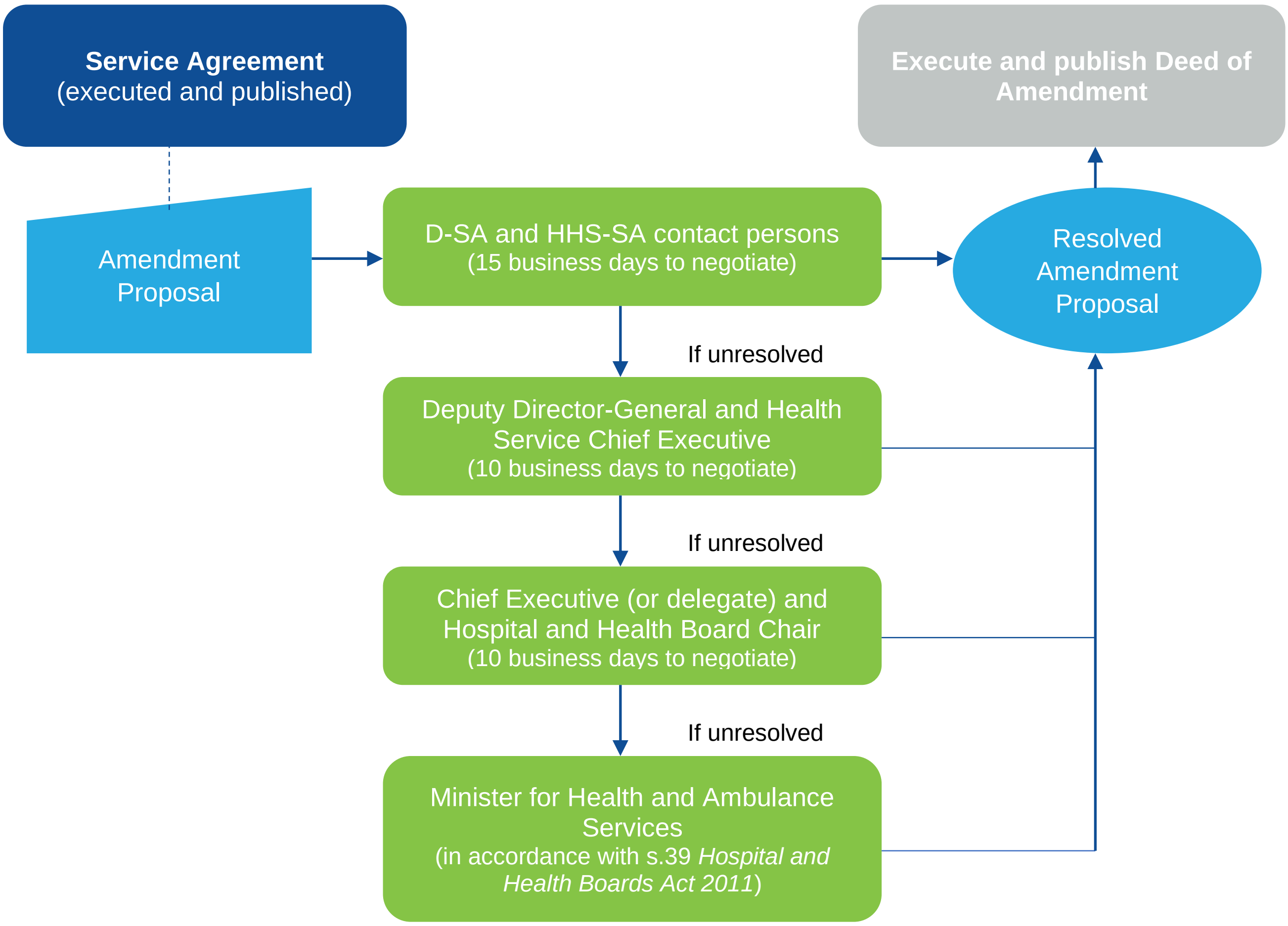

(i) The timeframes identified in Figure 3 may be reduced in the complete discretion of the Chief Executive.

(j) In accordance with section 39(5) of the *Hospital and Health Boards Act 2011*, the Parties must include any terms decided by the Minister in the Service Agreement.

(k) If the Chief Executive at any time:

(i) considers that an amendment agreed with the HHS may or will have associated impacts on other HHSs; or

(ii) considers it appropriate for any other reasons,

then the Chief Executive may:

(iii) propose further amendments to any HHS affected; and

(iv) may address the amendment and/or associated impacts of the

amendment in other ways, including through the exercise of any statutory powers and/or statutory directions under the *Hospital and Health Boards Act 2011*.

(l) Amendment Proposals that are resolved will be documented in a Deed of Amendment to this Service Agreement and executed by the Parties.

(m) Only upon execution of a Deed of Amendment by the Parties will the amendments documented by that Deed of Amendment be deemed to be an amendment to this Service Agreement.

3.3 **Extraordinary Amendment Windows**

(a) A Party that wants to amend the terms of this Service Agreement outside of an Amendment Window outlined in Table 14 must give an Extraordinary Amendment Proposal to the other Party.

(b) An Extraordinary Amendment Proposal may only be formally negotiated and resolved outside of an Amendment Window outlined in Table 14 to facilitate funding allocations where an urgent priority needs to be addressed in a timely manner and an Amendment Window is not available within an acceptable timeframe.

(c) An Extraordinary Amendment Proposal that is issued by or on behalf of the Chief Executive must be given to the HHS-SA Contact Person through Healthcare Purchasing and System Performance Division.

(d) An Extraordinary Amendment Proposal that is issued by or on behalf of the HHS must be given to the D-SA Contact Person through Healthcare Purchasing and System Performance Division.

(e) An Extraordinary Amendment Proposal may be issued by or on behalf of either Party at any time, noting the requirement that it relate to an urgent priority that necessitates timely resolution.

(f) Negotiation and resolution of Extraordinary Amendment Proposals will be through a tiered process as outlined in Figure 3.

(g) The timeframes identified in Figure 3 may be reduced in the complete discretion of the Chief Executive.

(h) In accordance with section 39(5) of the *Hospital and Health Boards Act 2011*, the Parties must include any terms decided by the Minster in the Service Agreement.

(i) Extraordinary Amendment Proposals that are resolved must be executed by both Parties.

(j) The Parties must comply with the terms of the Extraordinary Amendment Proposal from the date that the final Party executed the Extraordinary Amendment Proposal.

(k) The terms of an executed Extraordinary Amendment Proposal will be documented in a Deed of Amendment to this Service Agreement and executed by the Parties. Once executed, the Deed of Amendment will expressly exclude the application of the Extraordinary Amendment Proposal and only the terms of the Deed of Amendment will apply.

3.4 Periodic adjustments

(a) The Service Agreement Value may be adjusted outside of an Amendment Window to allow for funding variations that:

(i) occur on a periodic basis;

(ii) are referenced in the Service Agreement; and

(iii) are based on a clearly articulated formula.

(b) Adjustments to the Service Agreement Value and purchased activity that are required as a result of a periodic adjustment will be made following agreement between the Parties of the data on which the adjustment is based.

(c) The Chief Executive (or delegate) will issue an Adjustment Notice to the HHS-SA Contact Person confirming any funding adjustment that has been made.

(d) Following receipt of an Adjustment Notice, the Parties will comply with the Adjustment Notice and immediately take the steps necessary to give effect to the requirements of the Adjustment Notice.

(e) A Deed of Amendment will not be issued immediately following periodic adjustment. The HHS will be provided with a summary of all transactions made through periodic adjustment on completion.

(f) Any funding adjustments agreed through periodic adjustment which result in a variation to the Service Agreement Value, purchased activity or the requirements specified within Schedule 2 of this Service Agreement will be formalised in a Deed of Amendment issued following the next available Amendment Window.

3.5 End of financial year adjustments

(a) End of year financial adjustments may be determined after the financial year end outside of the Amendment Window process.

(b) The scope will be defined by the Department and informed by Queensland Government Central Agency requirements.

(c) The Department will provide the HHS with a reconciliation of all Service Agreement funding and purchased activity for the prior financial year. This will reflect the agreed position between the Parties following conclusion of the end of year financial adjustments process.

(d) The impact of end of year financial adjustments on subsequent year funding and activity will be incorporated in the Service Agreement through the Deed of Amendment executed following the next available Amendment Window.

(e) This clause will survive expiration of this Service Agreement.

Schedule 6 Definitions

In this Service Agreement:

Activity Based Funding (ABF) means the funding framework for publicly-funded health care services delivered across Queensland. The ABF framework applies to those Queensland public sector health service facilities which are operationally large enough and have the systems which are required to support the framework. The ABF framework allocates health funding to these hospitals based on standardised costs of health care services (referred to as 'activities') delivered. The Framework promotes smarter health care choices and better care by placing greater focus on the value of the health care delivered for the amount of money spent.

Adjustment Notice means the written notice of a proposed funding adjustment made by or on behalf of the Chief Executive in accordance with the terms of this Service Agreement.

Administrator of the National Health Funding Pool means the position established by the *National Health Reform Amendment (Administrator and National Funding Body) Act 2012* for the purposes of administering the National Health Funding Pool according to the National Health Reform Agreement.

Agreement means this Service Agreement.

Ambulatory Care means the care provided to hospital patients who are not admitted to the hospital, such as patients of emergency departments and outpatient clinics. Can also be used to refer to care provided to patients of community-based (non-hospital) healthcare services.

Amendment Proposal means the written notice of a proposed amendment to the terms of this Service Agreement as required under section 39 of the *Hospital and Health Boards Act 2011*.

Amendment Window means the period within which Amendment Proposals are negotiated and resolved. Amendment Windows commence on the relevant Exchange Date as specified in Table 14 Schedule 5 and end at the conclusion of the Negotiation Period.

Block Funding means funding for those services which are outside the scope of ABF.

Business Day means a day which is not a Saturday, Sunday or public holiday in Brisbane.

Chair means the Chair of the Hospital and Health Board.

Chief Executive means the chief executive of the Department.

Clinical Product/Consumable means a product that has been Clinically Prescribed.

Clinically Prescribed means prescribed by appropriately qualified and credentialed clinicians relative to the product.

Clinical Prioritisation Criteria means Statewide minimum criteria to determine if a referral to specialist medical or surgical outpatients is appropriate and, if so, the urgency of that referral.

Clinical Services Capability Framework means the Clinical Services Capability Framework for Public and Licensed Private Health Facilities which provides a standard set of minimum capability criteria for service delivery and planning. The Framework outlines the minimum service requirements, staffing, support services and risk considerations for both public and private health services to ensure safe and appropriately supported clinical service delivery. It applies to all public and private licensed facilities in Queensland. References to the Clinical Services Capability

Framework in this Service Agreement mean the most recent approved version unless otherwise specified.

Community Health Service means non-admitted patient Health Services, excluding hospital outpatient services, typically delivered outside of a hospital setting.

Deed of Amendment means the resolved amendment proposals.

Department means the department administering the *Hospital and Health Boards Act 2011* (Qld), which, at the date of this Service Agreement is known as 'Queensland Health'. To avoid any doubt, the term does not include the Hospital and Health Services.

D-SA Contact Person means the position nominated by the Department as the primary point of contact for all matters relating to this Service Agreement.

Effective Date means1 July 2019.

Efficient Growth means the increased in-scope activity-based services delivered by a HHS measured on a year to year basis in terms of both the Queensland efficient price for any changes in the volume of services provided and the growth in the national efficient price of providing the existing volume of services.

Eligible Population (Oral Health Services) refers to the proportion of the population for whom publicly funded oral health services is to be provided and is defined by the following criteria:

(a) adults, and their dependents, who are Queensland residents; eligible for Medicare and, where applicable, currently in receipt of benefits from at least one of the following concession cards:

 (i) Pensioner Concession Card issued by the Department of Veteran's Affairs;

 (ii) Pensioner Concession Card issued by Centrelink;

 (iii) Health Care Card (this includes Low Income Health Care Card Holders who are automatically eligible for services);

 (iv) Commonwealth Seniors Health Card;

 (v) Queensland Seniors Card.

(b) children who are Queensland residents or attend a Queensland school, are eligible for Medicare, and are:

 (i) eligible for dental program/s funded by the Commonwealth Government; or

 (ii) four years of age or older and have not completed Year 10 of secondary school; or

 (iii) dependents of current concession card holders or hold a current concession card.

Exchange Date means the date on which the Parties must provide Amendment Proposals for negotiation, as specified in Table 14 Schedule 5.

Extraordinary Amendment Window means an Amendment Window that occurs outside of the Amendment Windows specified in Table 14 Schedule 5, in accordance with the provisions of clause 3.3 of Schedule 5.

Force Majeure means an event:

(a) which is outside of the reasonable control of the Party claiming that the event has occurred; and

(b) the adverse effects of which could not have been prevented or mitigated against by that Party by reasonable diligence or precautionary measures, and includes lightning, earthquake, fire, cyclone, flood, natural disasters, health pandemics, acts of terrorism, riots, civil disturbances, industrial disputes and strikes (other than strikes involving that Party, its' agents, employees or suppliers), war (declared or undeclared), revolution, or radioactive contamination.

Formal Agreement means an agreed set of roles and responsibilities relating to the provision and receipt of services designated as Statewide or Regional:

(a) Statewide or Regional service provision

- (i) ensure equitable and timely access to entire catchment (clinical and non-clinical)
- (ii) provide training and consultation Services where this is appropriate within the agreed model of care (clinical and non-clinical)
- (iii) timely discharge or return of patients to their place of residence (clinical Services)
- (iv) adequate communication practices to enable ongoing effective local health care, including with the patient's General Practitioner where required (clinical Services)

(b) Recipient HHS

- (i) utilisation of standardised referral criteria, where they exist, to ensure appropriate use of Statewide Services (clinical services)
- (ii) timely acceptance of patients being transferred out of Statewide Services (back-transfers) (clinical Services)
- (iii) equitable access to ongoing local health care as required (clinical services)

Health Executive means a person appointed as a health executive under section 67(2) of the *Hospital and Health Boards Act 2011.*

Health Service has the same meaning as set out in section 15 of the *Hospital and Health Boards Act 2011.*

Health Service Chief Executive means a health service chief executive appointed for an HHS under section 33 of the *Hospital and Health Boards Act 2011.*

Health Service Employee means all person, appointed as a 'health service employee' for the HHS under section 67(1) of the *Hospital and Health Boards Act 2011.*

Hospital and Health Board means the hospital and health board appointed under section 23 of the *Hospital and Health Boards Act 2011*.

Hospital and Health Service or **HHS** means the Hospital and Health Service to which this Agreement applies unless otherwise specified.

HHS-SA Contact Person means the position nominated by the HHS as the primary point of contact for all matters relating to this Service Agreement.

HR Management Functions means the formal system for managing people within the HHS, including recruitment and selection; onboarding; induction and orientation; capability, learning and development; industrial and employee relations; performance management; work health and safety and well-being; workforce planning; diversity and inclusion; and workforce consultation, engagement and communication.

Industrial Instrument means an industrial instrument made under the *Industrial Relations Act 2016.*

Inter-HHS Dispute means a dispute between two or more HHSs.

Key Performance Indicator means a measure of performance that is used to evaluate the HHSs success in meeting key priorities.

Low Benefit Care means use of an intervention where evidence suggests it confers no or very little benefit on patients, or the risk of harm exceeds the likely benefit.

Minister means the Minister administering the *Hospital and Health Boards Act 2011* (Qld).

National Health Reform Agreement means the document titled *National Health Reform Agreement* made between the Council of Australian Governments (CoAG) in 2011, and incorporating all subsequent amendments agreed between the Commonwealth of Australia and the States and Territories.

Negotiation Period means a period of no less than 15 business days (or such longer period agreed in writing between the Parties) from each Exchange Date.

Notice of Dispute means the written notice of a dispute provided by the Chief Executive or the HHS to the other party or the written notice of a dispute provided by an HHS to another HHS.

Outcome Indicator means a measure of performance that provides information on specific activities and interventions where there is a reasonable expectation or evidence that they make a positive contribution towards improving the health status and experience of patients;

Outreach Service means a Health Service delivered on sites outside of the HHS area to meet or complement local service need. Outreach services include Health Services provided from one HHS to another as well as Statewide Services that may provide Health Services to multiple sites.

Own Source Revenue means, as per Section G3 of the *National Healthcare Agreement*, ‘private patients, compensable patients and ineligible persons may be charged an amount for public hospital services as determined by the state and territory’. The funding for these patients is called own source revenue and includes:

(a) Medicare ineligible patients, such as overseas visitors (not covered under reciprocal agreements), people in community detention and overseas students studying in Australia

(b) compensable patients with an alternate funding source, such as:

  (i) workers’ compensation insurers;

  (ii) motor vehicle accident insurers;

  (iii) personal injury insurers;

  (iv) Department of Defence; and/or

  (v) Department of Veterans’ Affairs; and

  Medicare eligible patients can elect to be treated as a public or private patient, allowing HHS' to recoup a portion of the healthcare service delivery cost.

Party means each of the Chief Executive and the HHS to which this Service Agreement applies.

Patient Identifiable Data means data that could lead to the identification of an individual either directly (for example by name), or through a combination of pieces of data that are unique to that individual.

Performance Review Meeting means the forum established which routinely reviews and discusses a range of aspects of HHS and system wide performance in accordance with the accountabilities contained within this Service Agreement and the Performance and Accountability Framework. Attendance at Performance Review Meetings comprises:

(a) the D-SA Contact Person and the HHS-SA Contact Person;

(b) executives nominated by the Department; and

(c) executives nominated by the HHS.

Performance Measure means a quantifiable indicator that is used to assess how effectively the HHS is meeting identified priorities and objectives.

Person Conducting a Business or Undertaking takes the meaning as defined in the *Work Health and Safety Act 2011,* section 5.

Prevention Services means programs that prevent illness and injury, promote health and wellbeing, create healthy and safe environments, reduce health inequalities and address factors in those communities whose health status is the lowest.

Primary Care means first level healthcare provided by a range of healthcare professionals in socially appropriate and accessible ways and supported by integrated referral systems. It includes health promotion, illness prevention, care of the sick, advocacy and community development.

Public Health Event of State Significance means an event where the actual or potential impact extends beyond the community service by a particular Hospital and Health Service.

Public Sector Health Service has the same meaning as set out in the *Hospital and Health Boards Act 2011.*

Public Sector Health System means the Queensland public sector health system, which is comprised of the Hospital and Health Services and the Department.

Quality Improvement Payment (QIP) means a non-recurrent payment due to the HHS for having met the goals set out in the QIP Purchasing Incentive Specification.

Queensland Government Central Agency means one or all of the Department of the Premier and Cabinet, Queensland Treasury, the Queensland Audit Office, the Public Service Commission and the Office of the Integrity Commissioner.

Regional Service means a clinical (direct or indirect patient care) or non-clinical Health Service funded and delivered, or coordinated and monitored, by an HHS with a catchment of two or more HHSs, but not on a Statewide basis as defined in this Schedule. Service delivery includes facility based, outreach and telehealth service models.

Referral Pathway means the process by which a patient is referred from one clinician to another in order to access the Health Services required to meet their healthcare needs.

Residential HHS means the HHS area, as determined by the *Hospital and Health Boards Regulation 2012*, in which the patient normally resides.

Safety and Quality Marker means a measure of performance that provides timely and transparent information on the safety and quality of Health Services provided by the HHS;

Schedule means this Schedule to the Service Agreement.

Senior Health Service Employee means a person appointed under section 67(2) of the *Hospital and Health Boards Act 2011* in a position prescribed as a 'senior health service employee position' under the *Hospital and Health Boards Regulation 2012.*

Service Agreement means this service agreement including the Schedules and annexures, as amended from time to time.

Service Agreement Value means the figure set out in Schedule 2 as the expected annual value of the services purchased by the Department through this Service Agreement.

State means the State of Queensland.

Statement of Building Portfolio Compliance means a declaration completed by the HHS stating that it has maintained compliance with all mandatory Acts, Regulations, Australian Standards and Codes of Practice applicable to the HHS' building portfolio.

Statewide Service means a service that is delivered by a lead provider to the State. A Statewide Service may be:

(a) a clinical service that is:

  (i) a low volume, highly specialised Health Service delivered from a single location;

  (ii) a highly specialised, or high risk[11], Health Service delivered in multiple locations or

  (iii) a prevention and/or health promotion service.

(b) a support service that is required to enable the delivery of specific direct clinical services; or

(c) services that have a primary role to provide clinical education services and/or training programs.

Statewide Service Description means a document that defines the Service to be provided by the HHS on a statewide basis and how the Statewide Service will be accessed and used by other HHSs across the State, including but not limited to:

(a) an overview of the Statewide Service;

(b) components of the Statewide Service;

(c) eligibility criteria;

(d) Service referrals and pathways; and

(e) governance and capability arrangements for the Statewide Service.

[11] A Health Service that, due to its nature, poses an increased threat of ongoing sustainability, efficiency and affordability.

Supporting Indicator means a measure of performance that provides contextual information to support an assessment of HHS performance.

Suspend and Suspension means to cause the temporary cessation of a service provided by the HHS under the terms of this Service Agreement. Suspension may result from, but is not exclusively due to, limitations in workforce capacity or issues regarding the safety or quality of the service provided.

Telehealth means the delivery of Health Services and information using telecommunication technology, including:

(a) live interactive video and audio links for clinical consultations and education;

(b) store and forward systems to enable the capture of digital images, video, audio and clinical data stored on a computer and forwarded to another location to be studied and reported on by specialists;

(c) teleradiology to support remote reporting and provision of clinical advice associated with diagnostic images; and

(d) telehealth services and equipment for home monitoring of health.

Terminate and Termination means the permanent cessation of a service provided by the HHS under the terms of this Service Agreement.

Treating HHS means the HHS area, as determined by the *Hospital and Health Boards Regulation 2012*, in which a patient is receiving treatment.

Value-Based Healthcare means delivering what matters most to patients in the most efficient way. Value-Based Healthcare is characterised by:

(a) the identification of clearly defined population segments of patients with similar needs around which clinically integrated teams organise and deliver care, rather than designing and organising care around medical specialities, procedures or facilities;

(b) a focus on delivering health outcomes and the experience of receiving care as defined from the patient perspective, not just the system or clinical perspective;

(c) connection between outcomes and the costs required to deliver the outcomes; and

(d) an integrated approach across the full cycle of care with a focus on the goal of health rather than just treatment.

Key Documents

Hospital and Health Services Service Agreements and supporting documents including:

(a) Hospital and Health Services Service Agreements

(b) Queensland Health System Outlook to 2026 for a sustainable health service

(c) Performance and Accountability Framework 2020/21

(d) Purchasing Policy and Funding Guidelines 2020/21

are available at: www.health.qld.gov.au/system-governance/health-system/managing/agreements-deeds

My health, Queensland's future: Advancing health 2026

www.health.qld.gov.au/__data/assets/pdf_file/0025/441655/vision-strat-healthy-qld.pdf

Queensland Health 2020-2021 System Priorities

[link to follow]

Department of Health Strategic Plan

www.health.qld.gov.au/system-governance/strategic-direction/plans/doh-plan

Guideline for Immunisation Services

https://www.health.qld.gov.au/__data/assets/pdf_file/0026/147545/qh-gdl-955.pdf

Queensland Health Statement of Action towards Closing the Gap in health outcomes

https://qheps.health.qld.gov.au/atsihb/html/statement-of-action

HHS Performance Measures and Attribute Sheets

https://qheps.health.qld.gov.au/purchasing-performance/hhs-agreements/performance-kpis

Data Supply Requirements

https://qheps.health.qld.gov.au/purchasing-performance/hhs-agreements/data-reporting-requirements

Australian Commission on Safety and Quality in Healthcare – National Safety and Quality Health Service Standards

https://www.safetyandquality.gov.au/standards/nsqhs-standards

Statewide Services Governance and Risk Management Framework

https://qheps.health.qld.gov.au/spb/html/statewide-services/statewide-services-governance-and-risk-management-framework

Public Health Practice Manual

https://qheps.health.qld.gov.au/__data/assets/pdf_file/0035/667754/public-health-prac-man.pdf

National Healthcare Agreement

http://www.federalfinancialrelations.gov.au/content/national_agreements.aspx

National Health Reform Agreement

www.federalfinancialrelations.gov.au/content/national_health_reform.aspx

Abbreviations

ACQSC	Aged Care Quality and Safety Commission
ABF	Activity Based Funding
ACSQHC	Australian Commission on Safety and Quality in Healthcare
CET	Clinical Education and Training
D-SA	Department – Service Agreement
HHS	Hospital and Health Service
HHS-SA	Hospital and Health Service – Service Agreement
HITH	Hospital in the Home
KPI	Key Performance Indicator
LAM	List of Approved Medicines
Non-ABF	Non-Activity Based Funding
NPA	National Partnership Agreement
NSQHS	National Safety and Quality Health Service Standards
NWAU	National Weighted Activity Unit
PBS	Pharmaceutical Benefits Scheme
QAS	Queensland Ambulance Service
QIP	Quality Improvement Payment
QWAU	Queensland Weighted Activity Unit
RACGP	Royal Australian College of General Practitioners
SA2	Statistical Area Level 2

This page is intentionally left blank.

Queensland Health
www.health.qld.gov.au

Queensland Government

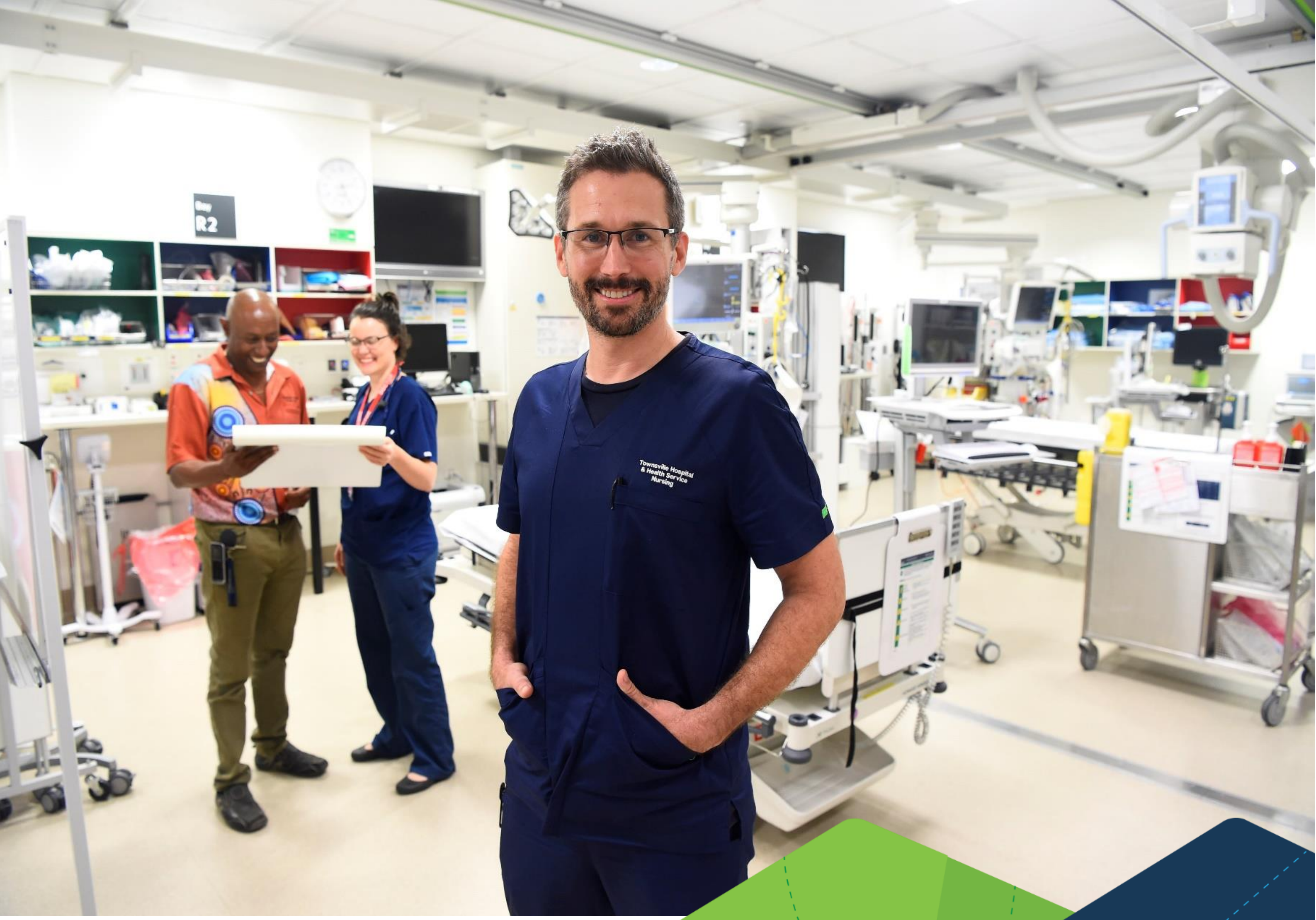

Queensland Health

Service Agreement 2022/23 – 2024/25

Townsville Hospital and Health Service

Queensland Government

Townsville Hospital and Health Service, Service Agreement 2022/23 - 2024/25

Published by the State of Queensland, (Queensland Health), July 2022

This document is licensed under a Creative Commons Attribution 3.0 Australia licence. To view a copy of this licence, visit creativecommons.org/licenses/by/3.0/au

© State of Queensland (Queensland Health) **2022**

You are free to copy, communicate and adapt the work, as long as you attribute the State of Queensland (Queensland Health).

For more information contact:

Contracting and Performance Management Branch, Department of Health, GPO Box 48, Brisbane QLD 4001
Tel (+61) (07) 3708 5869

Email SAM@health.qld.gov.au

An electronic version of this document is available at www.health.qld.gov.au/system-governance/health-system/managing/default.asp

Disclaimer:
The content presented in this publication is distributed by the Queensland Government as an information source only. The State of Queensland makes no statements, representations or warranties about the accuracy, completeness or reliability of any information contained in this publication. The State of Queensland disclaims all responsibility and all liability (including without limitation for liability in negligence) for all expenses, losses, damages and costs you might incur as a result of the information being inaccurate or incomplete in any way, and for any reason reliance was placed on such information.

Acknowledgement

We acknowledge the Traditional and Cultural Custodians of the lands, waters and seas across Queensland, pay our respects to Elders past and present, and recognise the role of current and emerging leaders in shaping a better health system.

We acknowledge the First Nations people in Queensland are both Aboriginal Peoples and Torres Strait Islander Peoples, and support the cultural knowledge, determination and commitment of Aboriginal and Torres Strait Islander communities in caring for health and wellbeing for millennia.

Townsville Hospital and Health Service is committed to honouring Aboriginal and Torres Strait Islander peoples' unique cultural and spiritual relationships to the land, waters, and seas and their rich contribution to society.

Townsville Hospital and Health Service is proud to recognise and celebrate the cultural diversity of our communities and workforce at the following locations:

Location	Traditional Custodians
Townsville	Bindal (Birri Gubba) and Gurrumbilbarra Wulgurukaba
Palm Island	Manbarra<br>Bwgcolman (historical)
Ayr/Home Hill	Bindal/Juru (Birri Gubba)
Charters Towers	Gudjal
Ingham	Nywaigi<br>Warrgamay<br>Bandjin
Cardwell	Girramay
Richmond	Wanamara / Woolgar Valley
Hughenden	Yerunthully

Contents

Acknowledgement 3

Contents 4

Tables 5

- **1.** Introduction 6
- **2.** Scope 6
- **3.** Services 6
- **4.** Performance and Accountability Framework 7
- **5.** Data supply requirements 7
- **6.** Hospital and Health Service accountabilities 8
- **7.** Department accountabilities 9
- **8.** Achieving health equity with First Nations Queenslanders 9
- **9.** General 10
- **10.** Counterparts 11

Execution 12

Schedule 1 HHS profile 13

- **1.** HHS profile 13

Schedule 2 Funding and purchased activity and services 14

- **1.** Introduction 14
- **2.** Purchased health services 15
- **3.** Teaching training and research 17
- **4.** Delivery of purchased activity 18
- **5.** Delivery of purchased services 19
- **6.** Financial adjustments 20
- **7.** Funding sources 24
- **8.** Funds disbursement 24

Schedule 3 Performance Measures 30

- **1.** Performance Measures 30

Schedule 4 Amendments to this service agreement 32

- **1.** Agreed process to amend this service agreement 32

Appendix 1 Reference Documents 34

Tables

Table 1 Statewide Services 15
Table 2 Financial adjustments applied on breach of activity thresholds 20
Table 3 Purchasing Incentives 2022/23 21
Table 4 Townsville HHS Total Funding Allocation by Funding Source 2022/23 26
Table 5 National Health Reform Funding 28
Table 6 Discretely Funded Programs (Non-ABF) 29
Table 7 HHS Performance Measures – Key Performance Indicators 30
Table 8 HHS Performance Measures - Safety and Quality Markers 31
Table 9 HHS Performance Measures – Outcome Indicators 31

1. Introduction

1.1 In performing this service agreement, the Department and the Hospital and Health Service (HHS) will act consistently with the object of the *Hospital and Health Boards Act 2011* (Qld) (Act).

1.2 The Department and the HHS operate as part of a networked system and agree to work collaboratively with each other, with other HHSs and with the Queensland Ambulance Service in the best interests of the Queensland public sector health system.

1.3 The parties will ensure that planning and delivery of health services is consistent with the strategies and priorities set out by government. The parties recognise the importance of the HHS' Health Equity Strategy (as defined in the Act) and the parties commitment to improving health and wellbeing outcomes and achieving health equity for Aboriginal and Torres Strait Islander peoples.

2. Scope

2.1 This service agreement covers the period from 1 July 2022 to 30 June 2025.

2.2 The parties have identified the services to be provided by the HHS, the funding for the provision of those services, the performance measures applicable to the services and data requirements.

3. Services

3.1 In delivering services, the HHS is required to meet:

(a) the applicable conditions of all national agreements and national partnership agreements between the Queensland Government and the Commonwealth Government and commitments under any related implementation plans; and

(b) the applicable conditions of each agreement or arrangement for funding between the Department and the HHS and commitments under any related implementation plans, which conditions and commitments may be recorded separately to this service agreement but for which funding is (or becomes) provided in Schedule 2.

3.2 The HHS is required to deliver the services outlined in this service agreement for which funding is provided in Schedule 2.

3.3 Where issues arise which prevent the HHS from providing a service or necessitate a reduction in the level or scope of a service provided, prompt notification must be made to the Department and impacted HHSs, with appropriate details. The HHS must minimise any clinical risk or adverse impact to patient experience that may result from service disruption. The Department will respond to the HHS on any requirements it has concerning service delivery and any adjustments triggered by under delivery.

3.4 If the HHS wishes to terminate or reduce service levels for a service for which funding is provided in Schedule 2, this will remain subject to negotiation and agreement by the Department at its discretion.

3.5 For any new services proposed during the term of this service agreement, the parties agree:

(a) the service must meet a demonstrated clinical need and provide value for money;

(b) the commencement of a new service, including the implementation of new models of care, may occur where the service has been commissioned by the Department, a funding stream is in place and any conditions relating to the funding have already been agreed; and

(c) if the HHS wishes to commence providing a new service that has not been commissioned by the Department, this will remain subject to agreement by the Department at its discretion.

3.6 It is acknowledged that there may from time to time need to be service delivery changes between HHSs. Management of inter-HHS relationships should be informed by the following principles:

(a) HHSs should maintain the proportion of out of HHS work undertaken unless as a result of agreed repatriation of patients;

(b) each HHS should manage patients from its own catchment population if it is within its clinical capability to do so as specified by the Clinical Services Capability Framework; and

(c) where it is proposed that a service move from one HHS to another, the Department will consider, as part of its review under clause 3.4 and 3.5, whether the respective Health Service Chief Executives endorse the proposed change in patient flows; and the funding required to follow the patient.

4. Performance and Accountability Framework

4.1 The Queensland Health Performance and Accountability Framework (the Performance and Accountability Framework) sets out the framework within which the Department monitors and manages the performance of public sector health services in Queensland.

4.2 The parties will act consistently with the Performance and Accountability Framework.

5. Data supply requirements

5.1 The HHS will provide the Department with all clinical and non-clinical data that is reasonably required to support the effective management of the public sector health system. This will include, but is not limited to, data that is required to:

(a) fulfil legislative obligations;

(b) deliver accountabilities and obligations to State and Commonwealth Governments; including related to the provision and reconciliation of activity data by the Administrator of the National Health Funding Pool;

(c) monitor and support performance improvement;

(d) manage this service agreement;

(e) support clinical innovation; and

(f) facilitate evaluation and audit.

5.2 The parties agree and acknowledge that:

(a) the Department will keep the HHS informed of the Department's data requirements; and

(b) data will be provided as required, or permitted, by law.

6. Hospital and Health Service accountabilities

6.1 The HHS will perform its obligations under this service agreement.

6.2 As applicable to the HHS and its services, the HHS will comply with:

(a) legislation and subordinate legislation, including the Act;

(b) cabinet decisions;

(c) Ministerial directives;

(d) agreements entered into between the Queensland and Commonwealth governments (or agreements with others in furtherance of such agreements), of which it is informed;

(e) agreements entered into between the Department and other Queensland Government entities, of which it is informed;

(f) all industrial instruments;

(g) all health service directives and health employment directives; and

(h) all policies, guidelines and implementation standards, including human resource policies.

6.3 As part of the commitment to achieving First Nations health equity, the HHS will prioritise the elimination of racial discrimination and institutional racism within its service and ensure that Aboriginal and Torres Strait Islander peoples have access to culturally safe and responsive health services.

6.4 The HHS will ensure that effective health service planning and delivery systems are in place, working in collaboration with the Department.

6.5 The HHS will ensure that health service employees employed by the Chief Executive[1] who perform work for the HHS are managed in accordance with any applicable delegations and directions from the Chief Executive.

6.6 The HHS will ensure that effective asset management systems are in place, working in collaboration with the Department.

6.7 The HHS will maintain accreditation to the standards required by the Department.

6.8 The HHS will appropriately perform and fulfil its functions under the Act.

6.9 The HHS will provide to the Chief Executive reports of a type, and at the intervals, agreed between the parties, or as reasonably specified by the Chief Executive.

7. Department accountabilities

7.1 The Department will perform its obligations under this service agreement including, in return for the HHS performing its obligations and delivering the services, providing funding to the HHS as stipulated in this service agreement (as amended).

7.2 The Department will:

(a) comply with applicable legislation and subordinate legislation, including the Act, as relates to this service agreement; and

(b) perform the system manager role (as defined in the Act) through the Chief Executive;

7.3 The Chief Executive will appoint health service employees to perform work for the HHS for the purpose of enabling the HHS to perform its functions under the Act.

7.4 The Chief Executive will consult, cooperate, and coordinate with the HHS to ensure legal compliance with the *Work Health and Safety Act 2011* and other legislation as it applies to the scope, nature and location of operations associated with this service agreement.

7.5 The Chief Executive will appropriately perform and fulfil their functions under the Act.

8. Achieving health equity with First Nations Queenslanders

8.1 Through legislative amendments to the Act and the Hospital and Health Boards Regulation 2012, and the release of *Making Tracks Together: Queensland's Aboriginal and Torres Strait Islander Health Equity Framework* (2021), Queensland Health has strengthened its commitment to improving health and wellbeing outcomes and achieving health equity for First Nations peoples.

8.2 The HHS will develop a Health Equity Strategy to demonstrate the HHS's activities and key performance measures to achieve health equity with First Nations peoples that is compliant with legislative requirements. The Health Equity Strategy will act as the principal

[1] In this service agreement the term Chief Executive takes the meaning applied in the Act, which is the Chief Executive of Queensland Health, but which is generally referred to as the Director-General.

accountability mechanism between community and the HHS in achieving health equity for First Nations Queenslanders and forms a key part of the Queensland Government's commitment to the *National Agreement on Closing the Gap* (2020).

8.3 The HHS will review the Health Equity Strategy at least once every three years and will publish the Health Equity Strategy in a way that allows it to be accessed by members of the public.

8.4 The HHS will ensure that commitment and leadership is demonstrated through implementing the actions and achieving the key performance measures outlined in the Health Equity Strategy.

8.5 The HHS will report publicly on progress against the Health Equity Strategy.

8.6 The HHS will support the implementation of other supplementary policies and strategies to drive health equity across the public health system, including relevant election commitments.

8.7 The HHS will participate as a partner in the design, development and implementation of the new *Queensland First Nations Health Workforce Strategy for Action.*

9. General

9.1 Sub-contracting

(a) The HHS must have appropriate systems in place to ensure that any subcontractor is accredited (as applicable), qualified, and otherwise fit to perform any services for which it is contracted.

(b) The HHS must ensure that any sub-contractor who has access to confidential information (as defined in the Act) or personal information (as defined in the *Information Privacy Act 2009* (Qld)) complies with obligations no less onerous than those imposed on the HHS.

9.2 Insurance

The HHS must:

(a) hold and maintain the types and levels of insurances that the HHS considers appropriate according to its functions and obligations; and

(b) comply with reasonable requests or directions from the Department in this regard.

9.3 Amendment

The process for amending this service agreement is provided for under the Act and further outlined in Schedule 4.

10. Counterparts

10.1 This service agreement may be executed in two or more identical copy counterparts, each of which together will be deemed an original, but all of which together will constitute one and the same instrument.

10.2 In the event that any signature executing this service agreement or any part of this service agreement is delivered by facsimile transmission or by scanned e-mail delivery of a '.pdf' format data file or equivalent of the entire agreement, the signature will create a valid and binding obligation of the party executing (or on whose behalf the signature is executed) with the same force and effect as if the signature page were an original. For the avoidance of doubt, this service agreement may be in the form of an electronic document and may be electronically signed.

10.3 For execution under this clause 10 to be valid the entire service agreement upon execution by each individual party must be delivered to the remaining parties.

Execution

Executed as an agreement in Queensland

Signed by the Chief Executive, Queensland)
Health:)

Signature of Chief Executive

SHAUN DRUMMOND

Name of Chief Executive (print)

(date)

Signed for and on behalf of the Townsville)
Hospital and Health Service:)

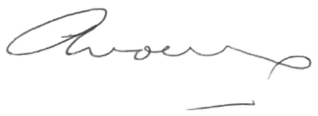

Signature of Hospital and Health Board Chair

ANTHONY MOONEY AM

Name of Hospital and Health Board Chair (print)

23 JUNE 2022

(date)

Execution

Executed as an agreement in Queensland

Signed by the Chief Executive, Queensland Health:)

....

Signature of Chief Executive

SHAUN DRUMMOND

....

Name of Chief Executive (print)

29 June 2022

....

(date)

Signed for and on behalf of the Townsville Hospital and Health Service:)

....

Signature of Hospital and Health Board Chair

....

Name of Hospital and Health Board Chair (print)

....

(date)

Schedule 1 HHS profile

1. HHS profile

This Schedule does not apply to this HHS.

Schedule 2 Funding and purchased activity and services

This Schedule 2 sets out:

(a) the services which are to be provided by the HHS;

(b) the activity purchased by the Department from the HHS;

(c) the funding provided for delivery of the purchased activity;

(d) the criteria and processes for financial adjustments associated with the delivery of purchased activity and specific funding allocations;

(e) the sources of funding that this service agreement is based on and the manner in which these funds will be provided to the HHS.

1. Introduction

1.1 The HHS will deliver the services for which funding is provided in this Schedule 2. In providing these services, the HHS will ensure that:

(a) all statewide and national policy frameworks, guidelines, protocols and implementation standards applicable to the service provided are followed;

(b) participation in national programs is facilitated and supported, including where these programs are provided by the Commonwealth Government;

(c) service delivery partnerships, including with other HHSs, primary care organisations and non-government organisations, are maintained and operate effectively;

(d) collaboration and engagement with other service providers and stakeholders is initiated and maintained to ensure that an integrated system of treatment, care and support is in place and to facilitate the delivery of comprehensive and effective services. This may include but is not limited to:

(i) other HHSs;

(ii) non-government organisations;

(iii) Aboriginal and Torres Strait Islander community-controlled health organisations;

(iv) Queensland Ambulance Service;

(v) services provided through the Department of Health (for example, Pathology Queensland);

(vi) primary care providers;

(vii) other government departments and agencies; and

(viii) private providers;

(e) models of care and service delivery arrangements are consistent with evidence-based practice and offer value for money;

(f) services are provided on an equitable basis to the community, and processes are in place to ensure that services reach identified target populations, high risk groups and hard to reach communities;

(g) referral networks and pathways continue to operate effectively; and

(h) innovation and continuous improvement is supported.

2. Purchased health services

2.1 Table 4, Table 5 and Table 6 outline the activity and service streams which the Department agrees to purchase from the HHS pursuant to this service agreement.

2.2 More generally, this will include the following:

2.3 **Statewide Services**

(a) The designation of a service as a statewide service (either a clinical statewide service or a clinical support statewide service) will be determined by the Department, consistent with the stipulated governance arrangements for such services.

(b) The HHS will:

(i) collaborate with the Department and other HHSs in the implementation of, and adherence to, the governance and oversight arrangements for statewide services;

(ii) participate in, and contribute to, the staged review of the purchasing model for identified statewide services; and

(iii) ensure that referral pathways in and out of each statewide service are followed.

2.4 **Clinical Statewide Services and Clinical Support Statewide Services provided]**

The HHS will provide the statewide services listed in Table 1.

Table 1 Statewide Services

Service Name	Categorisation
ANTS-NQ Retrieval Service (neonates)	Clinical Statewide Service
Yalurin Retrieval Service (children)	Clinical Statewide Service

2.5 **Regional services**

The HHS has responsibility for the provision and/or coordination of the following regional services:

(a) Aboriginal and Torres Strait Islander Child and Youth Care Coordination;

(b) Basic Physician Training Pathway;

(c) Community Forensic Outreach Services;

(d) Dual Disability Program;

(e) Mental Health Clinical Improvement Team Program;

(f) Forensic Adolescent Mental Health Services;

(g) Court liaison services for young people; and

(h) Mental Health Act delegate.

2.6 **Prevention services and population health services**

(a) The HHS will provide a range of services with a focus on the prevention of ill-health and disease, including:

(i) Specialist Public Health Units;

(ii) preventive health services;

(iii) immunisation services;

(iv) sexually transmissible infections including HIV and viral hepatitis;

(v) tuberculosis services; and

(vi) population health screening including, but not limited to, cancer screening services and newborn blood spot screening.

(b) Services will be provided in line with public health related legislation and the service delivery and reporting requirements outlined in the *Public Health Practice Manual*, as these relate to the services provided.

2.7 **Aboriginal and Torres Strait Islander health services**

The HHS will provide Aboriginal and Torres Strait Islander specific health services and initiatives consistent with the principles and objectives of the Queensland Government's *Making Tracks towards closing the gap in health outcomes for Indigenous Queenslanders by 2022 – Policy and Accountability Framework*. These service and initiatives will be delivered in line with guidance from the Aboriginal and Torres Strait Islander Health Division.

2.8 **Mental health alcohol and other drugs services**

The HHS will provide treatment, care and support for individuals who are, or may be, experiencing substance use disorders and/or other mental health disorders, mental health crisis and suicidal distress, and their families and carers. Services will be delivered in line with guidance from the Mental Health Alcohol and Other Drugs Branch:

2.9 **Oral health services**

The HHS will provide oral health services to people who meet the eligibility criteria for accessing public dental services in Queensland. Services will be delivered in line with guidance from the Office of the Chief Dental Officer.

2.10 **Prisoner health services**

The HHS will provide services for prisoners consistent with the principles, responsibilities and requirements specified in the Memorandum of Understanding (Prisoner Health Services) between Queensland Health and Queensland Corrective Services.

2.11 **Youth detention services**

The HHS will provide health services for people accommodated in the Cleveland Youth Detention Centre consistent with health services available to youth in the wider community.

2.12 **Refugee health**

The HHS will operate a health service for refugees, special humanitarian entrants and asylum seekers.

2.13 **State-funded outreach services**

(a) Where state-funded outreach services are provided;

(i) funding for the service will remain part of the providing HHS's funding allocation; and

(ii) the activity must be recorded at the HHS where the outreach service is being provided

(b) Any changes to the provision of outreach services will follow the requirements set out in clause 3 of this service agreement.

3. Teaching training and research

The HHS will provide the teaching, training and research programs for which funding is provided within Schedule 2 and as described below.

3.1 **Clinical education and training**

(a) The HHS will provide education and training placements for the following professional groups consistent with and proportionate to the capacity of the HHS and will support and align with stipulated placement terms governing clinical placements in Queensland Health facilities;

(i) medical students;

(ii) nursing and midwifery students;

(iii) pre-entry clinical allied health students;

(iv) interns;

(v) rural generalist trainees;

(vi) vocational medical trainees;

(vii) first year nurses and midwives;

(viii) re-entry to professional register nursing and midwifery candidates;

(ix) dental students;

(x) allied health rural generalist training positions;

(xi) Aboriginal and Torres Strait Islander Health Workers and Aboriginal and Torres Strait Islander Health Practitioners.

(b) The HHS will comply with the state-wide vocational medical training pathways.

(c) The HHS will support profession specific and inter-profession statewide allied health clinical education programs.

(d) The HHS will continue to implement and retain the following positions provided through clauses to the *Health Practitioners and Dental Officers (Queensland Health) Certified Agreement (No 3)*:

(i) health practitioner research positions provided through the Research Package for Health Practitioners; and

(ii) clinical educator positions provided through the Clinical Education Management Initiative for Health Practitioners.

(e) The HHS will maintain or increase its contribution of staff to the Queensland Country Relieving doctors program and the receiving HHS will be responsible for wages, clinical governance and appropriate supervision of the junior medical relievers.

3.2 **Statewide training, education and research**

This clause does not apply to this HHS.

3.3 **Health and medical research**

The HHS will:

(a) develop and implement a strategy to drive increased research activity and its translation into clinical practice;

(b) support increased and equitable access to clinical trials for patients;

(c) support clinicians to undertake research linked to their practice; and

(d) ensure high quality and timely research governance approval processes.

4. Delivery of purchased activity

4.1 The Department and the HHS will monitor actual activity against purchased levels and will take action as necessary to ensure delivery of purchased levels is achieved. The HHS has a responsibility to only recognise revenue that is linked with actual activity delivered against purchased volumes.

4.2 The HHS will actively monitor variances from purchased activity levels and notify the Department as soon as the HHS becomes aware of significant variances.

4.3 The HHS will also notify the Department of deliberate changes to the consistent recording of activity within year that would result in additional activity being recorded for existing health services.

4.4 If the HHS wishes to convert activity between purchased activity types, programs and levels the HHS must negotiate this with the Department based on a sound needs based rationale.

4.5 The Department will reconcile in-scope activity, as defined in the Activity Reconciliation specification sheet (available online, as detailed in Appendix 1), delivered by the HHS against the purchased in-scope activity targets outlined in Table 4.

4.6 Activity reconciliation will be undertaken in February (for the July to December period) and August (for the January to June period) each year and will be derived through application of the methodology which is documented in the Activity Reconciliation specification sheet.

4.7 Should the HHS be unable to deliver the activity that has been funded a financial adjustment will be applied.

4.8 Under delivery of in-scope activity, as defined in the Activity Reconciliation specification sheet, will be withdrawn from the HHS at 100% of the Queensland Efficient Price (QEP).

4.9 Funding and corresponding activity that is withdrawn from the HHS may be reallocated to an alternate provider that can undertake the activity.

4.10 Any resulting changes to funding and purchased activity levels will be actioned through the processes outlined in Schedules 2 and 4 of this service agreement.

4.11 The HHS will maintain its efforts to accurately record the delivered activity and submit this data to the Department in accordance with the agreed requirements.

5. Delivery of purchased services

5.1 As part of the service agreement, the Department purchases a range of services (or deliverables) from the HHS for the delivery of certain programs and projects.

5.2 These program or project services may be the subject of separately agreed conditions tied to that funding and the focus of detailed monitoring by the Department.

5.3 Conditions may include, but are not limited to:

(a) establishment and/or commencement of services;

(b) delivery of activity;

(c) workforce obligations;

(d) establishment of oversight committees;

(e) opening or upgrades to facilities;

(f) program evaluation;

(g) program management;

(h) reporting or notification obligations; and

(i) attainment of performance standards.

5.4 The HHS will ensure that the conditions are achieved within the stipulated time period.

5.5 The HHS will notify the Department if the HHS forecasts an inability to achieve program or project objectives or the conditions.

5.6 The Department may withdraw allocated funding pro rata to the level of under delivery if the services for a specified program or project are not being fully delivered according to the objectives or conditions.

5.7 Any resulting changes to funding and purchased activity levels will be actioned through the processes outlined in Schedules 2 and 4 of this service agreement.

6. Financial adjustments

6.1 Activity targets

(a) The Department will initiate a joint process with the HHS to determine whether a financial adjustment should be applied in relation to any purchased activity which has breached the thresholds identified bi-annually. This process will take into account any relevant matters that have been identified in a review/analysis of the breach as well as the outcomes of the activity plan implemented to address the activity breach.

(b) Activity will be monitored at the purchasing hierarchy level. Providing the HHS meets all relevant KPIs and specific funding allocations, the HHS has the ability to negotiate the transfer of activity across the purchasing hierarchy with the Department.

(c) Table 2 demonstrates the financial adjustment that will be applied when activity thresholds have been breached.

(d) The HHS may not utilise the provisions within AASB15 *Revenue from Contracts with Customers* to override the application of any financial adjustment made by the Department in line with Table 2.

Table 2 Financial adjustments applied on breach of activity thresholds

Example of Breach	Description	Financial Adjustment
Over performance	Activity exceeds that specified in the service agreement value for in-scope activity as shown in Table 4.	Purchasing contracts are capped and an HHS will not be paid for additional activity with the exception of activity that is in scope for the identified purchasing incentives as set out in Table 3.
Under performance	Activity is below that specified for in-scope activity as shown in Table 4.	Purchased activity and the related funding will be withdrawn at 100% of the Queensland Efficient Price and reallocated to an alternate provider that can undertake the activity.<br>Refer to Table 4 for the HHS QWAU target.
Failure to deliver on service commitments linked to specific funding allocations	Specific funding allocations<br>National Partnership Agreements.	It is at the discretion of the Department to withdraw allocated funding pro rata to the level of under delivery.
For all other types of activity variance, any financial adjustment will be made at the discretion of the Department.		

6.2 National Partnership on COVID-19 Response

(a) The Department will provide additional funding to the HHS under the *National Partnership on COVID-19 Response* where actual additional cost is demonstrated.

(b) Funding will be provided where the HHS has:

(i) undertaken activity that is in-scope for the State Public Health Payment, as defined under the *National Partnership on COVID-19 Response*, during the reporting period; and/or

(ii) undertaken activity that is in-scope for the Hospital Services Payment, as defined under the *National Partnership on COVID-19 Response*, during the reporting period; and

(iii) met the reporting requirements in relation to COVID-19 expenditure, as prescribed under the *National Partnership on COVID-19 Response* and by the Department.

(c) Additional costs that are reimbursed through the State Public Health Payment and the Hospital Services Payment will be excluded from the calculation of activity eligible for funding under the terms of the *National Health Reform Agreement*.

(d) There is no guarantee that the HHS will have access to additional funding under the State Public Health Payment or the Hospital Services Payment.

(e) All funding that is provided through the State Public Health Payment and the Hospital Services Payment will be subject to a reconciliation to be undertaken by the Administrator of the National Health Funding Pool. The HHS will be required to provide evidence to support claims for additional costs. If the HHS is unable to provide the required evidence with their expenditure claim, funding received may be recalled subject to reconciliation.

(f) Funding adjustments will be actioned through the processes outlined in Schedules 2 and 4 of this service agreement.

6.3 Purchasing incentives

(a) The purchasing approach includes a range of funding adjustments which aim to incentivise high quality and high priority activity, support innovation and evidence-based practice, deliver additional capacity through clinically and cost effective models of care and dis-incentivise care which provides insufficient or no benefit for patients. This includes incentive payments for HHSs who achieve targets in specific priority areas. The purchasing incentives are detailed in Table 3. The Department must reconcile the applicable purchasing incentives in Table 3 in line with the timeframes specified in the relevant purchasing specification sheet. The Department must provide a copy of the reconciliation statement to the HHS.

(b) Funding adjustments must be based on the requirements contained in the relevant purchasing specification sheet for that purchasing incentive.

(c) Any resulting changes to funding and purchased activity levels will be actioned through the processes outlined in Schedules 2 and 4 of this service agreement.

Table 3 Purchasing Incentives 2022/23

**Incentive**	
**Quality Improvement Payment (QIP)**	
Antenatal care for First Nations Women	Payments for achieving two Closing the Gap targets for First Nations women:<br>• to attend five or more antenatal visits with their first antenatal first taking place in the first trimester; and<br>• to stop smoking by 20 weeks gestation.
**Purchasing incentives**	
Virtual care incentive	Incentive funding to increase the number of specialist outpatient services which are provided in virtual settings.
Own source revenue growth	Incentivise the recognition of own source revenue through matching growth in own source revenue with public activity growth funding.

Incentive	
**ABF model localisations**	
Sentinel events	Payment withdrawn for sentinel events as per the national ABF model.
Advance Care Planning (ACP)	QWAUs for HHSs who offer ACP discussions to admitted patients, non-admitted outpatients, community health patients and Emergency Department patients.
Emergency Department Did Not Wait (DNW)	Zero QWAUs for DNWs.
Fractured neck of femur	QWAUs reduced by 20% for non-timely surgical treatment of fractured neck of femur.
Hospital in The Home (HITH)	QWAUs increased for Hospital in the Home (HITH) admissions of Residential Aged Care Facility residents.
Out-of-scope services	Nil QWAUs for out-of-scope procedures.
Pre-operative bed days	QWAUs for long stay days above the upper trim point less ICU days reduced equivalent to pre-operative days, up to a maximum of 3 days, for elective episodes.
Smoking cessation (community mental health)	QWAUs for smoking cessation activity for community mental health patients.
Smoking cessation (inpatients)	QWAUs for smoking cessation activity for publicly funded inpatients.
Stroke care	10% QWAU loading for acute stroke patients admitted to Statewide Stroke Clinical Network-endorsed stroke unit care.
Telehealth (admitted patients)	QWAUs for provider-end of in-scope admitted patient telehealth activity.
**Commissioning mechanisms**	
High-cost home support	Funding for approved individuals requiring 24-hour home ventilation.
Patient flow initiative	Provision of non-recurrent WAU-backed funding to participating HHS who successfully implement agreed recommendations.
Rapid access clinics	Recurrent WAU-backed funding to support the implementation of rapid access clinics to reduce pressure on emergency departments.
Expansion of sub-acute and long stay care	Additional funding to increase the availability of and access to care for sub-acute and long stay patients, thereby improving access to care in a range of settings and releasing capacity within acute facilities.
Connected Community Pathways	Funding to incentivise evidence-based and innovative models of care which promote the delivery of care outside acute facilities and support shared-care partnership arrangements.

6.4 **Surgery Connect reimbursements**

(a) The HHS will reimburse the Department for the actual costs of activity for nominated patient referrals to the Surgery Connect program where:

(i) The HHS has nominated the patient referral as HHS funded on entry of the referral in the Surgery Connect Activity Navigator (SCAN); and

(ii) The HHS Chief Finance Officer has recorded approval of the nomination in SCAN;

or

(iii) The HHS has obtained the Department's agreement to retrospectively convert a defined patient cohort to HHS funded status in SCAN.

(b) The HHS may only request retrospective conversion of activity to HHS funded within the financial year in which the activity has taken place.

(c) Any resulting changes to funding and purchased activity levels will be actioned through the processes outlined in Schedules 2 and 4 of this service agreement.

6.5 **Financial adjustments – other**

(a) Notwithstanding the provisions regarding the recognition of revenue as stipulated in AASB1058 *Income of Not-for-Profit Entities* and/or AASB15 *Revenue from Contracts with Customers*, the Department may seek to recover funding from an HHS that was provided through this service agreement which has:

(i) not been utilised in accordance with its intended purpose; and/or

(ii) not been utilised within the prescribed time period to deliver the agreed outcomes/services.

(b) If the Chief Executive (or delegate) determines that previously allocated funding is to be recovered, any resulting changes to funding and purchased activity levels will be actioned through the processes outlined in Schedules 2 and 4 of this service agreement.

6.6 **Public and private activity/own source revenue**

(a) Own source revenue comprises grants and contributions, user charges and other revenues.

(b) Where an HHS is above its own source revenue target, it will be able to retain the additional own source revenue with no compensating adjustments to funding from other sources where the additional revenue is not attributable to a private patient consistent with clauses A9 to A13 and A44 of the *National Health Reform Agreement.*

(c) Where an HHS is below its own source revenue target in respect of private patients, it will experience a reduction in revenue with no compensating adjustments to funding from other sources.

(d) The HHS will make all efforts to identify and maximise revenue from appropriate third parties to ensure optimisation of available State and Commonwealth funding across the Queensland public sector health system.

(e) The own source revenue identified in Table 4 is an estimate generated by the HHS which allows all third-party funding sources associated with service delivery

to be identified. The HHS will ensure that this estimate is substantiated and accurate to ensure no significant variances to actual revenue generated.

(f) The HHS will routinely revise and update the estimate to ensure alignment between the service agreement and Queensland Treasury's reporting system (TRIDATA).

(g) Budget adjustments for changes in own source revenue from private patients will be actioned through the processes set out in Schedule 4 of this service agreement.

7. Funding sources

7.1 The four main funding sources contributing to the HHS service agreement value are:

(a) Commonwealth funding;

(b) State funding;

(c) grants and contributions; and

(d) own source revenue.

7.2 Table 4 provides a summary of the funding sources for the HHS and the total value of the service agreement.

8. Funds disbursement

8.1 The Department agrees to pay the HHS the amount described in Table 4 of Schedule 2, subject to:

(a) parliamentary appropriation and adequate funds being allocated to the Department; and

(b) the terms of this service agreement.

8.2 All payments under this service agreement will be made in accordance with the requirements of the *National Health Reform Agreement* and the Act.

8.3 The Chief Executive will direct the disbursement of both State and Commonwealth funding from the State Pool Account, the State Managed Fund and the Department of Health Expenditure account to the HHS.

8.4 However, the State (represented by the Chief Executive) will not:

(a) redirect Commonwealth payments between HHSs;

(b) redirect Commonwealth payments between funding streams (e.g., from ABF to block funding); and/or

(c) adjust the payment calculations underpinning the Commonwealth's funding.

8.5 The Department will pay state-funded activity-based funding, block funding and system manager funding to the HHS on a fortnightly basis in line with receipt of the State Appropriation Payment. The payment made will be equal to 1/26th of the state-funded component of the service agreement value described in Table 4.

8.6 The Department will pay Commonwealth-funded activity-based funding and block funding on a monthly basis in line with receipt of the Commonwealth payment. The payment made will be equal to 1/12th of the Commonwealth funded component of the service agreement value described in Table 4.

8.7 Where the parties have agreed to amend the service agreement value or the Commonwealth contribution amount changes, the fortnightly and/or monthly payments will be adjusted to reflect the amended service agreement value.

8.8 Service agreement payments may be made outside of these timeframes with the approval of the Department's Chief Finance Officer.

Table 4 Townsville HHS Total Funding Allocation by Funding Source 2022/23

Funding Source	NWAU (N2122)	QWAU (Q24)	Value ($)
**NHRA Funding**			
**ABF Pool**			
ABF Funding (in scope NHRA)[2]			
Commonwealth[2]	136,017		$327,779,965
State		138,418	$392,677,475
State Specified Grants			$20,310,047
State-wide Services			$2,857,701
**State Managed Fund**			
Block Funding			
Small Rural Hospitals		15,366	$87,321,642
Teaching, Training & Research			$30,545,878
Non-Admitted Child & Youth Mental Health			$11,616,069
Non-Admitted Home Ventilation			$718,380
Non-Admitted Mental Health			$25,707,780
Other Non-Admitted Service			$0
Highly Specialised Therapies			$0
**Total NHRA Funding**	**136,017**	**153,784**	**$899,534,938**
**Out of Scope NHRA**			
**Queensland ABF Model**			
DVA		1,783	$11,347,911
NIISQ/MAIC		1,065	$2,295,055
Oral Health		2,062	$10,468,957
BreastScreen		595	$3,798,985
**Total Queensland ABF Funding**	**-**	**5,506**	**$27,910,908**
**Discretely Funded Programs[3]**			
Department of Health			$97,199,584
Locally receipted funds			$16,369,238
**Total Discretely Funded Programs**	**-**	**-**	**$113,568,822**
**Own Source Revenue**			
Private Patient Admitted Revenue[4]	*2,704*	2,988	$15,134,500
Non-Admitted Services		3,238	$9,637,500
Pharmaceuticals Benefits Scheme		3,680	$35,381,634
Other Activities[5]		1,220	$24,839,911
**Total Own Source Revenue**	**-**	**11,126**	**$84,993,545**

[2] The split between Commonwealth and State NHRA funding will change during the year as the purchased activity targets are updated and the National Health Funding Board updates their payment advice accordingly.

[3] Includes all other (non-ABF) State and Commonwealth funded health services including, but not limited to, Prisoner Health, Public Health and Prevention Services.

[4] The estimated value of the revenue earned from private patients, based on OSR estimates provided by the HHS.

[5] Incorporates all OSR which is not identified elsewhere in Table 4.

Funding Source	NWAU (N2122)	QWAU (Q24)	Value ($)
Locally Receipted Funds (exc. Discretely Funded Programs)[6]			$16,148,511
Depreciation			$60,796,000
**NPA COVID-19 Response**			
Hospital Services Payment			$0
State Public Health Payment			$0
COVID-19 Vaccine Payment			$0
**Total NPA COVID-19 Response Funding**	**-**	**-**	**$0**
**GRAND TOTAL**	**136,017**	**170,416**	**$1,202,952,724**

Pool Accounts			
ABF Pool (National Health Funding Pool)[7]			$771,536,097
State Managed Fund[8]			$155,909,750
System Manager			$97,199,584

[6] Includes items such as training programs and donations. Does not include locally receipted funds associated with discretely funded programs, e.g.Transition Care.
[7] Articulates the financial payment made to support in-scope ABF services under the NHRA including DVA, NIISQ, MAIC and Breastscreen Services. Applies to all HHSs except Central West HHS and Torres and Cape HHS.
[8] Articulates the payment made for block funded services under the NHRA, DVA, NIISQ and MAIC services.

Table 5 National Health Reform Funding

NHRA Funding Type	NWAU (N2122)	Commonwealth ($)	State ($)	Other State funding[9] DVA/MAIC/Oral Health/BreastScreen ($)	Total ($)
**National Efficient Price (NEP)**					**$5,597**
**ABF Allocation (NWAU)**					
Emergency Department	13,408	$32,310,301	$40,991,170	$5,895,897	$79,197,369
Acute Admitted	88,422	$213,081,869	$270,330,974	$0	$483,412,842
Admitted Mental Health	7,002	$16,874,897	$21,408,707	$0	$38,283,605
Sub-Acute	9,568	$23,057,930	$29,252,948	$0	$52,310,878
Non-Admitted	17,617	$42,454,968	$53,861,424	$22,015,011	$118,331,403
**Total ABF Pool Allocation**	**136,017**	**$327,779,965**	**$415,845,223**	**$27,910,908**	**$771,536,097**
**Block Allocation**					
Teaching Training and Research	-	$6,298,221	$24,247,656	-	$30,545,878
Small and Rural Hospitals[10]	-	$31,505,927	$55,815,715	-	$87,321,642
Non-Admitted Mental Health	-	$9,789,554	$15,918,226	-	$25,707,780
Non-Admitted Child & Youth Mental Health	-	$1,184,983	$10,431,086	-	$11,616,069
Non-Admitted Home Ventilation	-	$334,444	$383,936	-	$718,380
Other Non-Admitted Services	-	$0	$0	-	$0
Other Public Hospital Programs	-	$0	$0	-	$0
Highly Specialised Therapies	-	$0	$0	-	$0
**Total Block Allocation**	**-**	**$49,113,129**	**$106,796,620**	**-**	**$155,909,750**
**Grand Total Funding Allocation**					**$927,445,846**

[9] State funding transacted through the Pool Account; not covered under the NHRA

[10] Incorporating small regional and rural public hospitals, five specialist mental health facilities (Baillie Henderson Hospital, Jacaranda Place – Queensland Adolescent Extended Treatment Centre, The Park – Centre for Mental Health, Kirwan Rehabilitation Unit and Charters Towers Rehabilitation Unit) and the Ellen Barron Family Centre.

Table 6 Discretely Funded Programs (Non-ABF)

Discretely Funded Programs	$
Aged Care Assessment Program	$1,008,557
Alcohol, Tobacco and Other Drugs	$4,371,802
Community Health Programs	$23,073,970
Disability Residential Aged Care Services	$0
Home and Community Care Program (HACC)	$3,219,238
Interstate Patients (QLD residents)	$3,141,971
Multi-purpose Health Services	$916,172
Other State Funding	$21,087,953
Patient transport	$6,513,634
Prevention Services and Public Health	$8,486,961
Prisoner Health Services	$11,001,851
Research	$651,671
Transition Care	$2,000,000
Residential Aged Care Services	$28,095,041
**TOTAL**	**$113,568,822**

Schedule 3 Performance Measures

1. Performance Measures

1.1 The performance of the HHS will be measured according to the assessment criteria and processes described in the Performance and Accountability Framework.

1.2 The detailed specification for each of the performance measures listed in this service agreement are provided through performance measure attribute sheets.

1.3 The performance measures identified in this service agreement are applicable to the HHS unless otherwise specified within the attribute sheet.

1.4 HHSs are also required to report against the agreed key performance measures in their Health Equity Strategy.

Table 7 HHS Performance Measures – Key Performance Indicators

Key Performance Indicators
Hospital Acquired Complications
Hospital Access Target (admitted patients)<br>• % of emergency stays within 4 hours
Emergency Department stays greater than 24 hours
Emergency Department wait time by triage category
Face to face community follow up within 1-7 days of discharge from an acute mental health inpatient unit
Patient off stretcher time
Lost minutes per ambulance (in development)
Patient flow target: time between the decision to admit and patient leaving the Emergency Department (in development)
Category 1 elective surgery patients treated within the clinically recommended timeframe
Elective surgery patients waiting longer than the clinically recommended timeframe
Emergency Surgery (placeholder - measure to be determined)
Category 1 patients who receive their initial specialist outpatient appointment within the clinically recommended timeframe
Patients waiting longer than clinically recommended for their initial specialist outpatient appointment
Category 4 gastrointestinal endoscopy patients treated within the clinically recommended timeframe
Gastrointestinal endoscopy patients waiting longer than the clinically recommended timeframe
Access to oral health services (adults)
Access to oral health services (children)
Potentially Preventable Hospitalisations – First Nations peoples:<br>• Diabetes complications<br>• Selected conditions
Reduction in the proportion of Aboriginal and Torres Strait Islander failure to attend appointments
Telehealth utilisation rates:<br>• Number of non-admitted telehealth service events
Forecast operating position:<br>• Full year<br>• Year to date

Average sustainable Queensland Health FTE
Capital expenditure performance
Proportion of mental health and alcohol and other drug service episodes with a documented care plan
Proportion of overnight inpatients discharged by 10am

Table 8 HHS Performance Measures - Safety and Quality Markers

Safety and Quality Markers
Sentinel Events
Hospital Standardised Mortality Ratio
Healthcare-associated Staphylococcus Aureus (including MRSA) bacteraemia
Severity Assessment Code (SAC) analysis completion rates
Patient Reported Experience

Table 9 HHS Performance Measures – Outcome Indicators

Outcome Indicators
Rate of seclusion events
Rate of absent without approval from acute mental health inpatient care
Reperfusion therapy for acute ischaemic stroke
Access to emergency dental care
First Nations peoples representation in the workforce
General oral health care for First Nations peoples
% of low birthweight babies born to Queensland mothers
Complaints resolved within 35 calendar days
Advance care planning
Smoking cessation clinical pathway
Potentially Preventable Hospitalisations (diabetes complications)
Potentially Preventable Hospitalisations (non-diabetes complications)
The percentage of oral health activity which is preventive
Cardiac rehabilitation
Adolescent vaccinations administered via the statewide School Immunisation Program

Schedule 4 Amendments to this service agreement

1. Agreed process to amend this service agreement

1.1 The parties acknowledge that this service agreement is subject to amendment, which will generally occur through:

(a) amendment windows;

(b) extraordinary amendment;

(c) periodic adjustments; and

(d) end of financial year reconciliation.

1.2 **Amendment windows**

(a) There will be set periods of time nominated by the Department during the year (amendment windows) in which a party may propose an amendment and the parties will endeavour to negotiate and finalise proposals to amend this service agreement.

(b) Amendment proposals that are agreed will be documented in a deed of amendment to this service agreement.

(c) Further details on the amendment window process, including the timing of amendment windows, is provided online, as detailed in Appendix 1.

1.3 **Extraordinary Amendment**

(a) Outside an amendment window, the Department and the HHS agree to limit any proposal to amend the terms of this service agreement to those where there is an urgent priority need to facilitate a funding allocation (extraordinary amendment). The parties will endeavour to negotiate and finalise any such proposal urgently.

(b) The process for submitting, negotiating and resolving an extraordinary amendment is available online, as detailed at Appendix 1.

(c) Agreed extraordinary amendments will be reflected in a notice issued by the Chief Executive countersigned as accepted by the HHS, which notice will be replaced when the extraordinary amendment is subsequently formalised in a deed of amendment issued following the next amendment window.

1.4 **Periodic adjustments**

(a) The service agreement value (and corresponding purchased activity) may be adjusted at any time to reflect funding variations that:

(i) occur on a periodic basis or in line with adjustments permitted for specific funding allocations;

(ii) are referenced in the service agreement; and

(iii) are based on a clearly articulated formula, an agreed basis or such other reasonably substantiated basis tied to performance.

(periodic adjustment).

(b) Periodic adjustments will be reflected in an adjustment notice issued by the Chief Executive (or delegate) to the HHS, based on relevant data, and subsequently formalised in a deed of amendment issued following the next amendment window.

1.5 **End of financial year reconciliation**

(a) There will be an end of financial year reconciliation process, with the scope defined by the Department and informed by Queensland Government Central Agency requirements.

(b) The Department will provide the HHS with a reconciliation of all service agreement funding and purchased activity for the prior financial year. This will reflect the position following conclusion of the end of financial year adjustments process.

(c) The impact of end of financial year adjustments on subsequent year funding and activity will be incorporated in the service agreement through the deed of amendment executed following the next available amendment window.

(d) This clause will survive expiration of this service agreement.

Appendix 1 Reference Documents

Hospital and Health Boards Act 2011

National Health Reform Agreement (NHRA) 2020-25

System Outlook to 2026 - for a sustainable health service

Queensland Health Performance and Accountability Framework

My health, Queensland's future: Advancing health 2026

Department of Health Strategic Plan 2021-2025

Local Area Needs Assessment (LANA) Framework

Making Tracks Together: Queensland's Aboriginal and Torres Strait Islander Health Equity Framework

Making Tracks toward closing the gap in health outcomes for Indigenous Queenslanders by 2033 - Policy and Accountability Framework

Aboriginal and Torres Strait Islander Health Workforce Strategic Framework 2016-2026

National Agreement on Closing the Gap

Queensland Health Workforce Diversity and Inclusion Strategy 2017 to 2022

Performance Measures Attribute Sheets

Purchasing Initiatives and Funding Specifications

Public Health Practice Manual

National Partnership on COVID-19 Response

Statewide services reference material

Service agreement amendment processes

Data supply requirements

Contracting and Performance Management Branch
GPO Box 48
Brisbane QLD 4001 Australia

Tel (+61) (07) 3708 5869

www.health.qld.gov.au

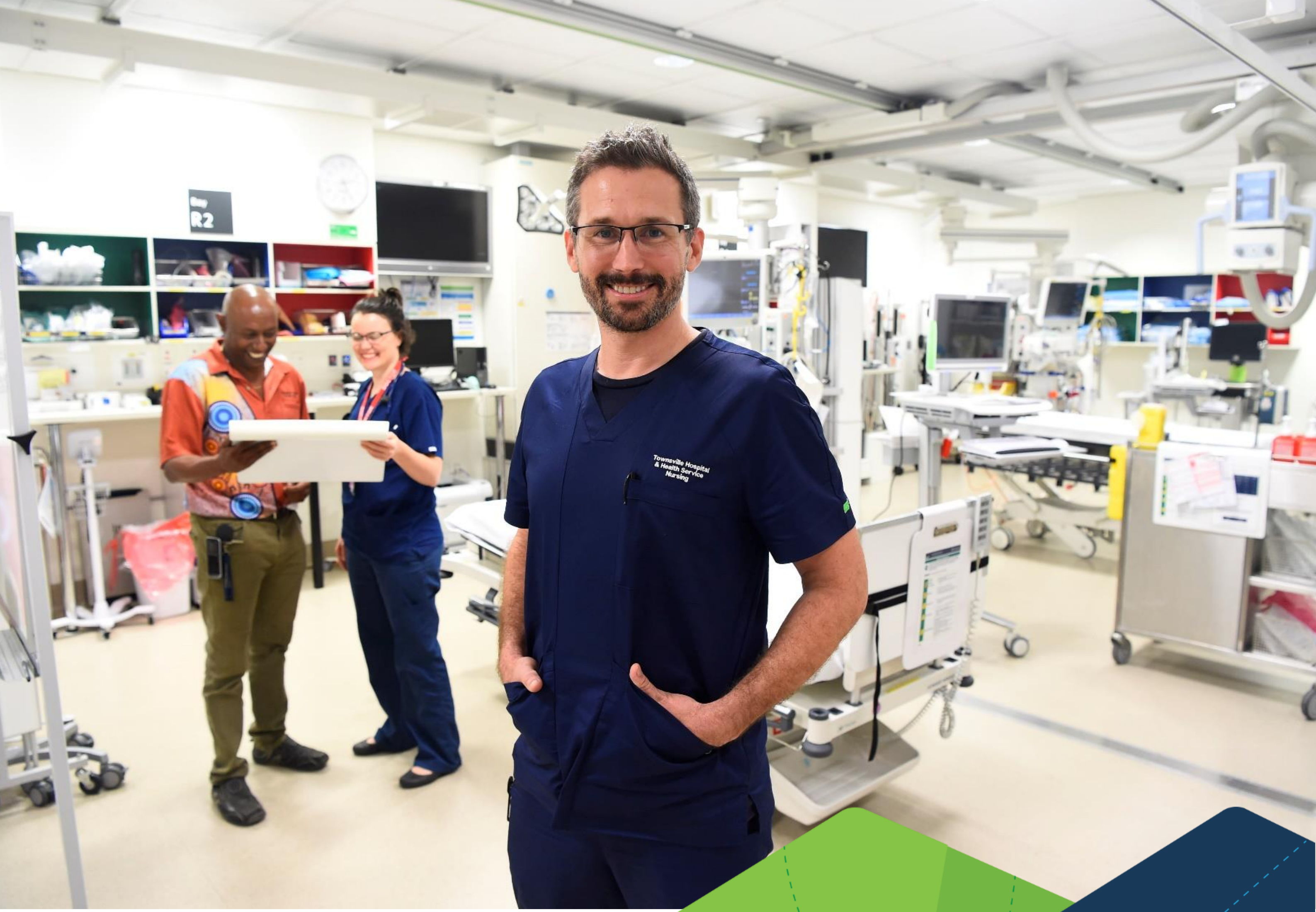

Queensland Health

Service Agreement 2022/23 – 2024/25

Townsville Hospital and Health Service

December 2024 Revision

Queensland Government

Townsville Hospital and Health Service, Service Agreement 2022/23 - 2024/25

Published by the State of Queensland, (Queensland Health), December 2024

This document is licensed under a Creative Commons Attribution 3.0 Australia licence. To view a copy of this licence, visit creativecommons.org/licenses/by/3.0/au

© State of Queensland (Queensland Health) **2024**

You are free to copy, communicate and adapt the work, as long as you attribute the State of Queensland (Queensland Health).

For more information contact:

Contracting and Performance Management Branch, Department of Health, GPO Box 48, Brisbane QLD 4001
Tel (+61) (07) 3708 5869

Email SAM@health.qld.gov.au

An electronic version of this document is available at www.health.qld.gov.au/system-governance/health-system/managing/default.asp

Disclaimer:
The content presented in this publication is distributed by the Queensland Government as an information source only. The State of Queensland makes no statements, representations or warranties about the accuracy, completeness or reliability of any information contained in this publication. The State of Queensland disclaims all responsibility and all liability (including without limitation for liability in negligence) for all expenses, losses, damages and costs you might incur as a result of the information being inaccurate or incomplete in any way, and for any reason reliance was placed on such information.

Acknowledgement

We acknowledge the Traditional and Cultural Custodians of the lands, waters, and seas across Queensland, pay our respects to Elders past and present, and recognise the role of current and emerging leaders in shaping a better health system.

We recognise the First Nations peoples in Queensland are both Aboriginal Peoples and Torres Strait Islander Peoples, and support the cultural knowledge, determination, and commitment of Aboriginal and Torres Strait Islander communities in caring for health and wellbeing for millennia.

Townsville Hospital and Health Service is committed to honouring Aboriginal and Torres Strait Islander peoples' unique cultural and spiritual relationships to the land, waters, and seas and their rich contribution to society.

Townsville Hospital and Health Service is proud to recognise and celebrate the cultural diversity of our communities and workforce at the following locations:

Location	Traditional Custodians
Townsville	Bindal (Birri Gubba) and Gurrumbilbarra Wulgurukaba
Palm Island	Manbarra<br>Bwgcolman (historical)
Ayr/Home Hill	Bindal/Juru (Birri Gubba)
Charters Towers	Gudjal
Ingham	Nywaigi<br>Warrgamay<br>Bandjin
Cardwell	Girramay
Richmond	Wanamara / Woolgar Valley
Hughenden	Yerunthully

Contents

Acknowledgement 3
Contents 4
Tables 5
1. Introduction 6
2. Scope 6
3. Services 6
4. Performance and Accountability Framework 7
5. Outcomes Framework 7
6. Data supply requirements 8
7. Hospital and Health Service accountabilities 8
8. Department accountabilities 10
9. Achieving health equity with First Nations Queenslanders 10
10. Dispute Resolution 11
11. General 11
12. Counterparts 12
Execution 13
Schedule 1 HHS profile 14
1. HHS profile 14
Schedule 2 Funding and purchased activity and services 15
1. Introduction 15
2. Purchased health services 16
3. Teaching, training, and research 19
4. Delivery of purchased activity 20
5. Delivery of purchased services 21
6. Financial adjustments 21
7. Funding sources 25
8. Funds disbursement 25
Schedule 3 Department of Health Provided Services 33
1. In scope services and service schedules 33
Schedule 4 Performance Measures 34
1. Performance Measures 34
Schedule 5 Amendments to this service agreement 38
1. Agreed process to amend this service agreement 38
Appendix 1 Reference Documents 40

Tables

Table 1 Statewide Services....16
Table 2 Financial adjustments applied on breach of activity thresholds....22
Table 3 Funding adjustments 2024/25....22
Table 4 HHS Funding by Outcomes Framework Care Domain....30
Table 5 HHS Total Funding Allocation by Funding Source 2024/25....30
Table 6 National Health Reform Funding....29
Table 7 Discretely Funded Programs (Non-ABF)....32
Table 8 Department of Health Provided Services and Service Schedules....34
Table 9 HHS Performance Measures – Key Performance Indicators....35
Table 10 HHS Performance Measures – Safety and Quality Markers....36
Table 11 HHS Performance Measures – Outcome Indicators....36
Table 12 Statewide Health Equity Key Performance Measures....36

1. Introduction

1.1 In performing this service agreement, the Department and the Hospital and Health Service (HHS) will act consistent with the object of the *Hospital and Health Boards Act 2011* (Qld) (Act).

1.2 The Department and the HHS operate as part of a networked system and agree to work collaboratively with each other, with other HHSs and with the Queensland Ambulance Service in the best interests of the Queensland public sector health system.

1.3 The parties will ensure that planning and delivery of health services is consistent with the strategies and priorities set out by government. The parties recognise the importance of the HHS' Health Equity Strategy (as defined in the Act) and the parties' commitment to improving health and wellbeing outcomes and achieving health equity for Aboriginal and Torres Strait Islander peoples.

2. Scope

2.1 This service agreement covers the period from 1 July 2022 to 30 June 2025.

2.2 The parties have identified the services to be provided by the HHS, the funding for the provision of those services, the performance measures applicable to the services and data requirements.

3. Services

3.1 In delivering services, the HHS is required to meet:

(a) the applicable conditions of all national agreements and national partnership agreements between the Queensland Government and the Commonwealth Government and commitments under any related implementation plans; and

(b) the applicable conditions of each agreement or arrangement for funding between the Department and the HHS and commitments under any related implementation plans, which conditions and commitments may be recorded separately to this service agreement but for which funding is (or becomes) provided in Schedule 2.

3.2 The HHS is required to deliver the services outlined in this service agreement for which funding is provided in Schedule 2.

3.3 Where issues arise which prevent the HHS from providing a service or necessitate a reduction in the level or scope of a service provided, prompt notification must be made to the Department and impacted HHSs, with appropriate details. The HHS must minimise any clinical risk or adverse impact to patient experience that may result from service disruption. The Department will respond to the HHS on any requirements it has concerning service delivery and any adjustments triggered by under delivery.

3.4 If the HHS wishes to terminate or reduce service levels for a service for which funding is provided in Schedule 2, this will remain subject to negotiation and agreement by the Department at its discretion.

3.5 For any new services proposed during the term of this service agreement, the parties agree:

(a) the service must meet a demonstrated clinical need and provide value for money;

(b) the commencement of a new service, including the implementation of new models of care, may occur where the service has been commissioned by the Department, a funding stream is in place and any conditions relating to the funding have already been agreed; and

(c) if the HHS wishes to commence providing a new service that has not been commissioned by the Department, this will remain subject to agreement by the Department at its discretion.

3.6 It is acknowledged that there may, from time to time, need to be service delivery changes between HHSs. Management of inter-HHS relationships should be informed by the following principles:

(a) HHSs should maintain the proportion of out of HHS work undertaken unless as a result of agreed repatriation of patients;

(b) each HHS should manage patients from its own catchment population if it is within its clinical capability to do so as specified by the Clinical Services Capability Framework; and

(c) where it is proposed that a service move from one HHS to another, the Department will consider, as part of its review under clauses 3.4 and 3.5, whether the respective Health Service Chief Executives endorse the proposed change in patient flows; and the funding required to follow the patient.

4. Performance and Accountability Framework

4.1 The Queensland Health Performance and Accountability Framework (the Performance and Accountability Framework) sets out the framework within which the Department monitors and manages the performance of public sector health services in Queensland.

4.2 The parties will act consistent with the Performance and Accountability Framework.

5. Outcomes Framework

5.1 Queensland Health is embarking on a strategic shift in funding focus from “volume” to “outcome” using the Outcomes Framework. This approach aims to link the resources and services required and delivered as part of healthcare activities, to health outcomes for individuals and the population.

5.2 The Outcomes Framework takes a three-tiered approach:

(a) The System Tier (Tier 1), which acts as a strategic tier, and includes four domains to measure the contribution of Queensland Health to the system outcomes.

(b) The Operational Tier (Tier 2) which includes nine (9) Clinical Care Domains, reflecting areas that are important to deliver change and improvement in the short to medium term, and to operationalise the Outcomes Framework.

(c) The Tactical Tier (Tier 3) provides scaffolding to select initiatives for implementation as specific pressures arise. These pressures may include areas identified for improvement through Tier 2.

5.3 In consultation with the State-wide Clinical Networks the indicators below are under further development and shadowing.

Indicator	Care Domain	Clinical Leadership
Percentage of patients who have HBA1C ordered during hospital admission	Chronic and Complex	Diabetes Network
Time to treatment for breast, colorectal and lung cancers	Cancer Care	Cancer Care Network

5.4 Schedule 4 maps existing indicators in the Performance and Accountability Framework to the care domains of the Outcomes Framework.

6. Data supply requirements

6.1 The HHS will provide the Department with all clinical and non-clinical data that is reasonably required to support the effective management of the public sector health system. This will include, but is not limited to, data that is required to:

(a) fulfil legislative obligations;

(b) deliver accountabilities and obligations to State and Commonwealth Governments; including related to the provision and reconciliation of activity data by the Administrator of the National Health Funding Pool;

(c) monitor and support performance improvement;

(d) manage this service agreement;

(e) support clinical innovation; and

(f) facilitate evaluation and audit.

6.2 The parties agree and acknowledge that:

(a) the Department will keep the HHS informed of the Department's data requirements; and

(b) data will be provided as required, or permitted, by law.

6.3 Further details on data supply requirements, including principles that guide the collection, storage, transfer and disposal of data and prescribed timeframes for data submission, are provided online as detailed in Appendix 1.

7. Hospital and Health Service accountabilities

7.1 The HHS will perform its obligations under this service agreement.

7.2 As applicable to the HHS and its services, the HHS will comply with:

(a) legislation and subordinate legislation, including the Act;

(b) cabinet decisions;

(c) Ministerial directives;

(d) agreements entered into between the Queensland and Commonwealth governments (or agreements with others in furtherance of such agreements), of which it is informed;

(e) agreements entered into between the Department and other Queensland Government entities, of which it is informed;

(f) agreements entered into with another HHS(s), including Networked Services Agreements;

(g) all industrial instruments;

(h) all health service directives and health employment directives; and

(i) all policies, guidelines, and implementation standards, including human resource policies.

7.3 As part of the commitment to achieving First Nations health equity, the HHS will prioritise the elimination of racial discrimination and institutional racism within its service and ensure that Aboriginal and Torres Strait Islander peoples have access to culturally safe and responsive health services.

7.4 The HHS will ensure that effective health service planning and delivery systems are in place, working in collaboration with the Department.

7.5 To support the achievement of the Queensland-Commonwealth Partnership's (QTP's) vision and commitment to work together to tackle health system challenges that cannot be overcome by any one organisation, HHSs are required to prepare and submit Joint Regional Needs Assessments in accordance with the framework provided online as detailed in Appendix 1.

7.6 HHSs must operate clinical service delivery consistent with the National Quality and Safety Standards. The HHS is expected to escalate any concerns that arise at the conclusion of a formalised assessment.

7.7 The HHS will ensure that health service employees employed by the Chief Executive[1] who perform work for the HHS are managed in accordance with any applicable delegations and directions from the Chief Executive. The HHS will ensure that effective asset management systems are in place (available online, as detailed in Appendix 1), that comply with the *Queensland Government Building Policy Framework and Guideline*, while working in collaboration with the Department.

7.8 The HHS will maintain accreditation to the standards required by the Department.

7.9 The HHS will appropriately perform and fulfil its functions under the Act.

[1] In this service agreement the term Chief Executive takes the meaning applied in the Act, which is the Chief Executive of Queensland Health, but which is generally referred to as the Director-General.

7.10 The HHS will provide to the Chief Executive reports of a type, and at the intervals, agreed between the parties, or as reasonably specified by the Chief Executive.

8. Department accountabilities

8.1 The Department will perform its obligations under this service agreement including, in return for the HHS performing its obligations and delivering the services, providing funding to the HHS as stipulated in this service agreement (as amended).

8.2 The Department will:

(a) comply with applicable legislation and subordinate legislation, including the Act, as relates to this service agreement;

(b) perform the system manager role (as defined in the Act) through the Chief Executive; and

(c) provide a range of services to the HHS as set out in Schedule 3.

8.3 The Chief Executive will appoint health service employees to perform work for the HHS for the purpose of enabling the HHS to perform its functions under the Act.

8.4 The Chief Executive will consult, cooperate, and coordinate with the HHS to ensure legal compliance with the *Work Health and Safety Act 2011* and other legislation as it applies to the scope, nature and location of operations associated with this service agreement.

8.5 The Chief Executive will appropriately perform and fulfil their functions under the Act.

9. Achieving health equity with First Nations Queenslanders

9.1 Through legislative amendments to the Act and the Hospital and Health Boards Regulation 2012, and the release of *Making Tracks Together: Queensland's Aboriginal and Torres Strait Islander Health Equity Framework* (2021), Queensland Health has strengthened its commitment to improving health and wellbeing outcomes and achieving health equity with First Nations peoples.

9.2 The HHS will develop and resource a First Nations Health Equity Strategy, compliant with legislative requirements. An implementation plan, accompanying the strategy, demonstrates the HHS's activities and key performance measures to achieve health equity with First Nations peoples. The Health Equity Strategy will act as the principal accountability mechanism between the Aboriginal and Torres Strait Islander community and the HHS in achieving health equity with First Nations Queenslanders and forms a key part of the Queensland Government's commitment to the *National Agreement on Closing the Gap* (2020).

9.3 The HHS is required to review the Health Equity Strategy at least once every three years and will publish the Health Equity Strategy in a way that allows it to be accessed by members of the public.

9.4 The HHS will ensure that commitment and leadership is demonstrated through implementing the actions and achieving the key performance measures outlined in the Health Equity Strategy.

9.5 The HHS will report publicly every year on progress against the Health Equity Strategy.

9.6 The HHS will support the implementation of other supplementary policies and strategies to drive health equity across the public health system, including relevant election commitments.

9.7 The HHS will participate as a partner in the implementation and achievement of Queensland’s *HealthQ32 First Nations First Strategy 2032* in addition to HHS commitments within their Health Equity Strategy.

10. Dispute Resolution

10.1 Where a dispute arises in connection to this agreement, either between the department and one or more HHSs or between HHSs, every effort should be made to resolve the dispute at the local level. If local resolution cannot be achieved, the dispute resolution processes, accessible through Appendix 1, must be followed.

11. General

11.1 **Sub-contracting**

(a) The HHS must have appropriate systems in place to ensure that any subcontractor is accredited (as applicable), qualified, and otherwise fit to perform any services for which it is contracted.

(b) The HHS must ensure that any sub-contractor who has access to confidential information (as defined in the Act) or personal information (as defined in the Information Privacy Act 2009 (Qld)) complies with obligations no less onerous than those imposed on the HHS.

11.2 **Insurance**

The HHS must:

(a) hold and maintain the types and levels of insurances that the HHS considers appropriate according to its functions and obligations; and

(b) comply with reasonable requests or directions from the Department in this regard.

11.3 **Amendment**

The process for amending this service agreement is provided for under the Act and further outlined in Schedule 5.

12. Counterparts

12.1 This service agreement may be executed in two or more identical copy counterparts, each of which together will be deemed an original, but all of which together will constitute one and the same instrument.

12.2 In the event that any signature executing this service agreement or any part of this service agreement is delivered by facsimile transmission or by scanned e-mail delivery of a '.pdf' format data file or equivalent of the entire agreement, the signature will create a valid and binding obligation of the party executing (or on whose behalf the signature is executed) with the same force and effect as if the signature page were an original. For the avoidance of doubt, this service agreement may be in the form of an electronic document and may be electronically signed.

12.3 For execution under this clause 12 to be valid, the entire service agreement upon execution by each individual party must be delivered to the remaining parties.

Execution

The terms of this Service Agreement were agreed under the provisions set out in the *Hospital and* Health *Boards Act,* section 35 on 29 June 2022, and were subsequently amended by the Deeds of Amendment entered into pursuant to section 39 of the *Hospital and Health Boards Act 2011* and executed on 22 December 2022, 13 April 2023, 29 June 2023, 22 December 2023, 18 April 2024, 6 August 2024 and 5 December 2024.

This revised Service Agreement consolidates amendments arising from:

- Periodic Adjustment – COVID-19 Funding Transfer September 2022
- Periodic Adjustment – COVID-19 Funding Transfer October 2022
- 2022/23 Amendment Window 2 (in year variation)
- Periodic Adjustment – COVID-19 Funding Transfer December 2022
- 2022/23 Amendment Window 3 (in year variation)
- Periodic Adjustment – COVID-19 Funding Transfer April 2023
- Extraordinary Amendment Window – May 2023
- 2023/24 Amendment Window 1 (Budget Build)
- 2023/24 Amendment Window 2 (in year variation)
- 2023/24 Amendment Window 3 (in year variation)
- 2024/25 Amendment Window 1 (Budget Build)
- 2024/25 Amendment Window 2 (in year variation)

Schedule 1 HHS profile

1. HHS profile

This Schedule does not apply to this HHS.

Schedule 2 Funding and purchased activity and services

This Schedule 2 sets out:

(a) the services which are to be provided by the HHS;

(b) the activity purchased by the Department from the HHS;

(c) the funding provided for delivery of the purchased activity;

(d) the allocation of funding provided against the care domains of the Outcomes Framework;

(e) the criteria and processes for financial adjustments associated with the delivery of purchased activity and specific funding allocations; and

(f) the sources of funding that this service agreement is based on and the way these funds will be provided to the HHS.

1. Introduction

1.1 The HHS will deliver the services for which funding is provided in this Schedule 2. In providing these services, the HHS will ensure that:

(a) all statewide and national policy frameworks, guidelines, protocols and implementation standards applicable to the service provided are followed;

(b) participation in national programs is facilitated and supported, including where these programs are provided by the Commonwealth Government;

(c) service delivery partnerships, including with other HHSs, primary care organisations and non-government organisations, are maintained and operate effectively;

(d) collaboration and engagement with other service providers and stakeholders is initiated and maintained to ensure that an integrated system of treatment, care and support is in place and to facilitate the delivery of comprehensive and effective services. This may include but is not limited to:

(i) other HHSs;

(ii) non-government organisations;

(iii) Aboriginal and Torres Strait Islander community-controlled health organisations;

(iv) Queensland Ambulance Service;

(v) services provided through the Department of Health (for example, Pathology Queensland);

(vi) primary care providers;

(vii) other government departments and agencies; and

(viii) private providers;

(e) models of care and service delivery arrangements are consistent with evidence-based practice and offer value for money;

(f) services are provided on an equitable basis to the community, and processes are in place to ensure that services reach identified target populations, high risk groups and hard to reach communities;

(g) referral networks and pathways continue to operate effectively; and

(h) innovation and continuous improvement are supported.

2. Purchased health services

2.1 Table 4 shows the allocation of funding from the Department to the HHS across the care domains of the Outcomes Framework. Table 5, Table 6, and Table 7 outline the activity and service streams which the Department agrees to purchase from the HHS pursuant to this service agreement.

2.2 More generally, this will include the following:

2.3 **Statewide Services**

(a) The designation of a service as a statewide service (either a clinical statewide service or a clinical support statewide service) will be determined by the Department, consistent with the stipulated governance arrangements for such services.

(b) The HHS will:

(i) collaborate with the Department and other HHSs in the implementation of, and adherence to, the governance and oversight arrangements for statewide services;

(ii) participate in, and contribute to, the staged review of the purchasing model for identified statewide services; and

(iii) ensure that referral pathways in and out of each statewide service are followed.

2.4 **Clinical Statewide Services and Clinical Support Statewide Services provided**

The HHS will provide the statewide services listed in Table 1.

Table 1 Statewide Services

Service Name	Categorisation
ANTS-NQ Retrieval Service (neonates)	Clinical Statewide Service
Yalurin Retrieval Service (children)	Clinical Statewide Service

2.5 Regional services

(a) The HHS has responsibility for the provision and/or coordination of the following regional services:

(i) Aboriginal and Torres Strait Islander Child and Youth Care Coordination

(ii) Basic Physician Training Pathway

(iii) Community Forensic Outreach Services

(iv) Dual Disability Program

(v) Mental Health Clinical Improvement Team Program

(vi) Forensic Adolescent Mental Health Services

(vii) Court liaison services for young people

(viii) Mental Health Act delegate

2.6 Prevention services and public health services

(a) The HHS will provide a range of prevention and public health services to promote and protect health, prevent illness and disease, and manage risk, including:

(i) Specialist Public Health Units

(ii) environmental health services, including risk assessment, regulation and enforcement in relation to environmental hazards, food safety, medicines and therapeutic goods, mosquitos and other vectors, pest management, poisons, radiation safety, chemical safety and water quality;

(iii) communicable disease services including immunisation, blood-borne viruses, sexually transmissible infections, infection control, notifiable conditions, mosquito-borne disease and tuberculosis;

(iv) management of incidents, emergencies and disasters, and disease outbreak readiness and response services;

(v) preventive health services;

(vi) population health screening including, but not limited to, cancer screening services and newborn blood spot screening;

(vii) public health epidemiology and surveillance;

(viii) mitigation and adaptation in response to climate risks.

(b) Services will be provided in line with public health related legislation and the service delivery and reporting requirements outlined in the Public Health Service Schedule and supported by the *Public Health Practice Manual*, as these relate to the services provided.

(c) Delivery of these services may be coordinated through specialist public health units, sexual health services, tuberculosis services, other areas of the HHS, or a combination of these.

2.7 Aboriginal and Torres Strait Islander health services

The HHS will provide Aboriginal and Torres Strait Islander specific health services and initiatives consistent with the principles and objectives of the Queensland Government's *Making Tracks toward closing the gap in health outcomes for Indigenous Queenslanders by 2033 – Policy and Accountability Framework* and the priorities committed to in the HHS's Health Equity Strategy. These services and initiatives will be delivered in line with guidance from the First Nations Health Office and the *First Nations First Strategy 2032.*

2.8 Mental health, alcohol, and other drugs services

The HHS will provide treatment, care and support for individuals who are, or may be, experiencing substance use disorders and/or other mental health disorders, mental health crisis and suicidal distress, and their families and carers. Services will be delivered in line with guidance from the Mental Health, Alcohol and Other Drugs Strategy and Planning Branch.

2.9 Oral health services

The HHS will provide oral health services to people who meet the eligibility criteria for accessing public dental services in Queensland. Services will be delivered in line with guidance from the Office of the Chief Dental Officer.

2.10 Prisoner health services

The HHS will provide services for prisoners consistent with the principles, responsibilities and requirements specified in the Memorandum of Understanding (Prisoner Health Services) between Queensland Health and Queensland Corrective Services.

2.11 Youth detention services

The HHS will provide health services for people accommodated in the Cleveland Youth Detention Centre consistent with health services available to youth in the wider community.

2.12 Refugee health

The HHS will operate a health service for refugees, special humanitarian entrants and asylum seekers.

2.13 State-funded outreach services

(a) Where state-funded outreach services are provided:

- (i) funding for the service will remain part of the providing HHS's funding allocation; and
- (ii) the activity must be recorded at the HHS where the outreach service is being provided.

(b) Any changes to the provision of outreach services will follow the requirements set out in clause 3 of this service agreement.

3. Teaching, training, and research

The HHS will provide the teaching, training and research programs for which funding is provided within Schedule 2 and as described below.

3.1 Clinical education and training

(a) The HHS will provide education and training placements for the following professional groups consistent with and proportionate to the capacity of the HHS and will support and align with stipulated placement terms governing clinical placements in Queensland Health facilities:

- (i) medical students;
- (ii) nursing and midwifery students;
- (iii) pre-entry clinical allied health students;
- (iv) interns;
- (v) rural generalist trainees;
- (vi) vocational medical trainees;
- (vii) first year nurses and midwives;
- (viii) re-entry to professional register nursing and midwifery candidates;
- (ix) dental students;
- (x) allied health rural generalist training positions;
- (xi) Aboriginal and Torres Strait Islander Health Workers and Aboriginal and Torres Strait Islander Health Practitioners.

(b) The HHS will comply with the state-wide vocational medical training pathways.

(c) The HHS will support profession specific and inter-profession statewide allied health clinical education programs.

(d) The HHS will continue to implement and retain the following positions provided through clauses to the *Health Practitioners and Dental Officers (Queensland Health) Certified Agreement (No 4) 2022*:

- (i) health practitioner research positions provided through the Research Package for Health Practitioners; and
- (ii) clinical educator positions provided through the Clinical Education Management Initiative for Health Practitioners.

(e) The HHS will maintain or increase its contribution of staff to the Queensland Country Relieving Doctors and the receiving HHS will be responsible for wages, clinical governance, and appropriate supervision of the junior medical relievers.

3.2 Statewide training, education, and research

This clause does not apply to this HHS.

3.3 **Health and medical research**

The HHS will:

(a) develop and implement a strategy to drive increased research activity and its translation into clinical practice;

(b) support increased and equitable access to clinical trials for patients;

(c) support clinicians to undertake research linked to their practice; and

(d) ensure high quality and timely research governance approval processes.

4. Delivery of purchased activity

4.1 The HHS is required to maintain accurate activity forecasts in the purchased target module of the Decision Support System (DSS) at all times. This information is imperative to the Department's assessment of State performance against the national Soft Cap and for outer-year planning. Activity forecasts must accurately reflect financial forecasts reported to the Finance Branch monthly.

4.2 The Department and the HHS will monitor actual activity against purchased levels and will act as necessary to ensure delivery of purchased levels is achieved. The HHS has a responsibility to only recognise revenue that is linked with actual activity delivered against purchased volumes.

4.3 The HHS will actively monitor variances from purchased activity levels and notify the Department as soon as the HHS becomes aware of significant variances.

4.4 The HHS will also notify the Department of deliberate changes to the consistent recording of activity within year that would result in additional activity being recorded for existing health services.

4.5 The HHS will undertake regular quality audits. The HHS is encouraged to publish its data quality framework describing audits undertaken and results achieved. For further information, refer to the Delivery of Purchased Activity – Requirement for Quality Audits specification sheet as detailed in Appendix 1.

4.6 If the HHS wishes to convert activity between purchased activity types, programs, and levels the HHS must negotiate this with the Department based on a sound needs based rationale.

4.7 The Department will reconcile in-scope activity, as defined in the Activity Reconciliation specification sheet (available online, as detailed in Appendix 1), delivered by the HHS against the purchased in-scope activity targets outlined in Table 5.

4.8 Activity reconciliations will be undertaken in the applicable End of Year Technical Amendment Window and subsequent Amendment Window 2 and will be derived through application of the methodology which is documented in the Activity Reconciliation specification sheet.

4.9 Should the HHS be unable to deliver the activity that has been funded a financial adjustment will be applied.

4.10 Funding and corresponding activity that is withdrawn from the HHS may be reallocated to an alternate provider that can undertake the activity.

4.11 Any resulting changes to funding and purchased activity levels will be actioned through the processes outlined in Schedules 2 and 5 of this service agreement.

4.12 The HHS will maintain its efforts to accurately record the delivered activity and submit this data to the Department in accordance with the agreed requirements.

5. Delivery of purchased services

5.1 As part of the service agreement, the Department purchases a range of services (or deliverables) from the HHS for the delivery of certain programs and projects.

5.2 These program or project services may be the subject of separately agreed conditions tied to that funding and the focus of detailed monitoring by the Department.

5.3 Conditions may include, but are not limited to:

(a) establishment and/or commencement of services;

(b) delivery of activity;

(c) workforce obligations;

(d) establishment of oversight committees;

(e) opening or upgrades to facilities;

(f) program evaluation;

(g) program management;

(h) reporting or notification obligations; and

(i) attainment of performance standards.

5.4 The HHS will ensure that the conditions are achieved within the stipulated time period.

5.5 The HHS will notify the Department if the HHS forecasts an inability to achieve program or project objectives or the conditions.

5.6 The Department may withdraw allocated funding pro rata to the level of under delivery if the services for a specified program or project are not being fully delivered according to the objectives or conditions.

5.7 Any resulting changes to funding and purchased activity levels will be actioned through the processes outlined in Schedules 2 and 5 of this service agreement.

6. Financial adjustments

6.1 **Activity targets**

(a) The Department will initiate a joint process with the HHS to determine whether a financial adjustment should be applied in relation to any purchased activity which

has breached the thresholds identified bi-annually. This process will take into account any relevant matters that have been identified in a review/analysis of the breach as well as the outcomes of the activity plan implemented to address the activity breach.

(b) Activity will be monitored at the purchasing hierarchy level. Providing the HHS meets all relevant KPIs and specific funding allocations, the HHS has the ability to negotiate the transfer of activity across the purchasing hierarchy with the Department.

(c) Table 2 demonstrates the financial adjustment that will be applied when activity thresholds have been breached.

(d) The HHS may not utilise the provisions within AASB15 Revenue from Contracts with Customers to override the application of any financial adjustment made by the Department in line with Table 2.

Table 2 Financial adjustments applied on breach of activity thresholds

Example of Breach	Description	Financial Adjustment
Over performance	Activity exceeds that specified in the service agreement value for in-scope activity as shown in Table 5.	Purchasing contracts are capped and an HHS will not be paid for additional activity apart from activity that is in scope for the identified purchasing incentives as set out in Table 3 (where applicable.)
Under performance	Activity is below that specified for in-scope activity as shown in Table 5.	Purchased activity and the related funding will be withdrawn at 100% of the Queensland Efficient Price and reallocated to an alternate provider that can undertake the activity. The reconciliation will be undertaken as outlined in the Activity Reconciliation Specification. Refer to Table 5 for the HHS QWAU target.
Failure to deliver on service commitments linked to specific funding allocations	Specific funding allocations<br>National Partnership Agreements.	It is at the discretion of the Department to withdraw allocated funding pro rata to the level of under delivery.
For all other types of activity variance, any financial adjustment will be made at the discretion of the Department.		

6.2 Purchasing approach

(a) The purchasing approach includes a range of funding adjustments (purchasing incentives and ABF model localisations) that aim to incentivise high quality and high priority activity, support innovation and evidence-based practice, deliver additional capacity through clinically and cost-effective models of care and dis-incentivise care providing insufficient or no benefit for patients. This includes incentive payments for HHSs who achieve targets in specific priority areas. The funding adjustments are detailed in Table 3. The Department must reconcile the applicable funding adjustments in Table 3 in line with the timeframes specified in

the relevant purchasing specification sheet. The Department must provide a copy of the reconciliation statement to the HHS.

(b) Funding adjustments must be based on the requirements contained in the relevant purchasing specification sheet.

(c) Any resulting changes to funding and purchased activity levels will be actioned through the processes outlined in Schedules 2 and 5 of this service agreement.

Table 3 Funding adjustments 2024/25

Funding adjustment	
**Purchasing incentives**	
Models of care/workforce	This program includes a range of initiatives focusing on incentivising:<br>• specific models of care; and<br>• the use of workforce operating at top of scope where there may be long wait lists and staff have not been available in a traditional model of care.
**ABF model localisations**	
Child Health Checks	QWAU loading for every in-scope check performed.
Unqualified neonate funding	Reduced Diagnosis Related Group (DRG) QWAU for all maternal delivery episodes with a liveborn outcome, discounted by the Diagnosis Related Group (DRG), with QWAUs re-allocated for unqualified neonates.
Maternity care for First Nations women	QWAUs to incentivise maternity care provided to First Nations mothers during pregnancy and to incentivise smoking cessation during pregnancy.
Sentinel events	Payment withdrawn for sentinel events as per the national ABF model.
Advance Care Planning (ACP)	QWAUs for HHSs offering ACP discussions to admitted patients, non-admitted outpatients, community health patients and Emergency Department patients.
Emergency Department Did Not Wait (DNW)	Zero QWAUs for DNWs.
Fractured neck of femur	QWAUs reduced by 20% for non-timely surgical treatment of fractured neck of femur.
Hospital in The Home (HITH)	QWAUs increased by 12.5% for Hospital in the Home (HITH) admissions of Residential Aged Care Facility residents.
Out-of-scope services	Nil QWAUs for out-of-scope procedures.
Pre-operative bed days	QWAUs for long stay days above the upper trim point less ICU days reduced equivalent to pre-operative days, up to a maximum of 3 days, for elective episodes.
Smoking cessation (community mental health)	QWAUs for smoking cessation activity for community mental health patients.
Smoking cessation (inpatients)	QWAUs for smoking cessation activity for publicly funded inpatients.
Stroke care	10% QWAU loading for acute stroke patients admitted to Queensland Stroke Clinical Network-endorsed stroke unit care.
Telehealth (admitted patients)	QWAUs for provider-end of in-scope admitted patient telehealth activity.
Allied Health Led Workforce for Pelvic Health and Gastroenterology	QWAU loading for an in-scope service event for Pelvic Health and Gastroenterology recorded against an Other Health Professional.

Funding adjustment	
Remote Patient Monitoring	QWAU loading for an in-scope non-admitted remote patient monitoring encounter per month per patient.

Surgery Connect reimbursements

(a) The HHS will reimburse the Department for the actual costs of activity for nominated patient referrals to the Surgery Connect program where:

(i) The HHS has nominated the patient referral as HHS funded or HHS Direct on entry of the referral in the Surgery Connect Activity Navigator (SCAN); and

(ii) The HHS Chief Finance Officer has recorded approval of the nomination in SCAN;

or

(iii) The HHS has obtained the Department's agreement to retrospectively convert a defined patient cohort to HHS funded status in SCAN.

(b) The HHS may only request retrospective conversion of activity to HHS funded within the financial year in which the activity has taken place.

(c) Any resulting changes to funding and purchased activity levels will be actioned through the processes outlined in Schedules 2 and 5 of this service agreement.

6.3 Financial adjustments – other

(a) Notwithstanding the provisions regarding the recognition of revenue as stipulated in AASB1058 Income of Not-for-Profit Entities and/or AASB15 Revenue from Contracts with Customers, the Department may seek to recover funding from an HHS that was provided through this service agreement which has:

(i) not been utilised in accordance with its intended purpose; and/or

(ii) not been utilised within the prescribed time period to deliver the agreed outcomes/services.

(b) If the Chief Executive (or delegate) determines that previously allocated funding is to be recovered, any resulting changes to funding and purchased activity levels will be actioned through the processes outlined in Schedules 2 and 5 of this service agreement.

6.4 Public and private activity/own source revenue

(a) Own source revenue comprises grants and contributions, user charges and other revenues.

(b) Where an HHS is above its own source revenue target, it will be able to retain the additional own source revenue with no compensating adjustments to funding from other sources where the additional revenue is not attributable to a private patient

consistent with clauses A9 to A13 and A44 of the *National Health Reform Agreement.*

(c) Where an HHS is below its own source revenue target in respect of private patients, it will experience a reduction in revenue with no compensating adjustments to funding from other sources.

(d) The HHS will make all efforts to identify and maximise revenue from appropriate third parties to ensure optimisation of available State and Commonwealth funding across the Queensland public sector health system.

(e) The own source revenue identified in Table 5 is an estimate generated by the HHS which allows all third-party funding sources associated with service delivery to be identified. The HHS will ensure that this estimate is substantiated and accurate to ensure no significant variances to actual revenue generated.

(f) The HHS will routinely revise and update the estimate to ensure alignment between the service agreement and Queensland Treasury's reporting system (TRIDATA).

(g) Budget adjustments for changes in own source revenue from private patients will be actioned through the processes set out in Schedule 5 of this service agreement.

7. Funding sources

7.1 The four main funding sources contributing to the HHS service agreement value are:

(a) Commonwealth funding;

(b) State funding;

(c) grants and contributions; and

(d) own source revenue.

7.2 Table 5 provides a summary of the funding sources for the HHS and the total value of the service agreement.

7.3 The HHS must undertake regular quality audits to check for potential duplicates in funding source, in particular the National Health Reform Agreement and Medicare given the Commonwealth's contribution to both funding sources. The HHS should take active steps to remedy areas of concern. A consumer's choice of funding arrangement should be reflected on a patient election form.

8. Funds disbursement

8.1 The Department agrees to pay the HHS the amount described in Table 5 of Schedule 2, subject to:

(a) parliamentary appropriation and adequate funds being allocated to the Department; and

(b) the terms of this service agreement.

8.2 All payments under this service agreement will be made in accordance with the requirements of the *National Health Reform Agreement* and the Act.

8.3 The Chief Executive will direct the disbursement of both State and Commonwealth funding from the State Pool Account, the State Managed Fund, and the Department of Health Expenditure account to the HHS.

8.4 However, the State (represented by the Chief Executive) will not:

(a) redirect Commonwealth payments between HHSs;

(b) redirect Commonwealth payments between funding streams (e.g., from ABF to block funding); and/or

(c) adjust the payment calculations underpinning the Commonwealth's funding.

8.5 The Department will pay state-funded activity-based funding, block funding and system manager funding to the HHS on a fortnightly basis in line with receipt of the State Appropriation Payment. The payment made will be equal to 1/26th of the state-funded component of the service agreement value described in Table 5.

8.6 The Department will pay Commonwealth-funded activity-based funding and block funding monthly in line with receipt of the Commonwealth payment. The payment made will be equal to 1/12th of the Commonwealth funded component of the service agreement value described in Table 5.

8.7 Where the parties have agreed to amend the service agreement value or the Commonwealth contribution amount changes, the fortnightly and/or monthly payments will be adjusted to reflect the amended service agreement value.

8.8 Service agreement payments may be made outside of these timeframes with the approval of the Department's Chief Finance Officer.

Table 4 HHS Funding by Outcomes Framework Care Domain 2024/25

Care Domain	Funding $	QWAU (Q27)
Prevention, early intervention, and primary health care	$159,815,709	11,027
Trauma and illness	$348,973,599	52,191
Mental health and alcohol and other drugs	$114,393,791	16,601
Cancer	$127,908,066	20,170
Planned care	$212,967,987	32,029
Maternity and neonates	$106,262,326	16,346
Chronic and complex	$343,707,560	44,854
Statewide services	$9,010,849	301
Depreciation	$81,003,000	0
**TOTAL**	**$1,504,042,887**	**193,519**

Table 5 HHS Total Funding Allocation by Funding Source 2024/25

Funding Source	24-25 NWAU (N2425)	24-25 QWAU (Q27)	24-25 Agreed ($)
**NHRA Funding**			
**ABF Pool**			
**ABF Funding (In scope NHRA)**[2]			
Commonwealth	159,511		$391,641,595
State		159,749	$548,215,914
State Specified Grants			$17,542,370
State-wide Services			$3,875,148
Restoring Planned Care	1,353	1,346	$8,530,000
Long Stay Patient Recovery Funding	748	750	$6,030,000
**Total ABF Funding (in scope NHRA)**	**161,612**	**161,845**	**$975,835,026**
**State Managed Fund**			
**Block Funding (State and Commonwealth)**			
Small rural hospital		5,964	$59,622,088
Teaching, Training and Research			$37,783,786
Other Mental Health	5,438	5,438	$43,126,083
Non-Admitted Home Ventilation			$767,887
Residential Mental Health Services		3,204	$13,451,398
Other Non-admitted Service			$0
Highly Specialised Therapies			$496,139
Other Public Hospital Programs			$0
**Total NHRA Funding**	**161,612**	**176,451**	**$1,131,082,407**
**Out of Scope NHRA**			
**Queensland ABF Model**			
DVA		1,665	$9,784,048
NIISQ/MAIC		459	$2,697,843
Oral Health		1,564	$11,122,529
Oral Health – FFA		106	$625,322
BreastScreen		491	$3,823,466
Child Health Checks		0	$0
**Total Queensland ABF Funding**		**4,285**	**$28,053,209**
**Discretely Funded Programs**[3]			
Department of Health			$124,391,357
Locally Receipted Funds			$20,090,819
Research (Other OSR)			$0
**Total Discretely Funded Programs**			**$144,482,176**

[2] The split between Commonwealth and State NHRA funding will change during the year as the purchased activity targets are updated and the National Health Funding Board updates their payment advice accordingly.

[3] Includes all other (non-ABF) State and Commonwealth funded health services including, but not limited to, Prisoner Health, Public Health and Prevention Services.

Funding Source	24-25 NWAU (N2425)	24-25 QWAU (Q27)	24-25 Agreed ($)
**Own Source Revenue**			
Private Patient Admitted Revenue[4]	3,498	3,450	$20,279,053
Pharmaceuticals Benefits Scheme		4,153	$41,592,650
Non-Admitted Services		3,680	$8,627,836
Other Activities[5]		1,500	$28,125,343
Oral Health – CDBS		0	$965,917
**Total Own Source Revenue**	**-**	**12,783**	**$99,590,799**
Locally Receipted Funds (exc. Discretely Funded Programs)[6]			$19,831,297
Depreciation			$81,003,000
**GRAND TOTAL**	**161,612**	**193,519**	**$1,504,042,888**

Pool Accounts			
ABF Pool (National Health Funding Pool)[7]			$1,003,888,235
State Managed Fund[8]			$155,247,380
System Manager			$124,391,357

[4] The estimated value of the revenue earned from private patients, based on OSR estimates provided by the HHS.

[5] Incorporates all OSR which is not identified elsewhere in Table 5.

[6] Includes items such as training programs and donations. Does not include locally receipted funds associated with discretely funded programs, e.g.Transition Care.

[7] Articulates the financial payment made to support in-scope ABF services under the NHRA including DVA, NIISQ, MAIC and Breastscreen Services. Applies to all HHSs except Central West HHS and Torres and Cape HHS.

[8] Articulates the payment made for block funded services under the NHRA, DVA, NIISQ and MAIC services.

Table 6 National Health Reform Funding

NHRA Funding Type	No. of In-scope services (NWAU)	No. of Out-of-scope services (QWAU)	Total Services (WAU)	ABF NEP ($)	State Price ($)	Funding for In-scope services at NEP ($)	Cwlth contribution for In-scope services ($)	State contribution for In-scope services ($)	State contribution for Out-of-scope services ($)	Total Cwlth and State contribution ($)
National Efficient Price (NEP)		*a,b*		*c*	*d*			*e*		
**ABF Allocation (NWAU)**										
Emergency Department	18,257	198	18,455	$6,465	$5,878	118,030,452	44,825,350	65,197,410	1,324,510	111,347,270
Acute Admitted	99,740	1,510	101,250	$6,465	$5,878	644,818,898	244,887,927	356,183,694	10,113,884	611,185,504
Admitted Mental Health	7,691	0	7,691	$6,465	$5,878	49,722,138	18,883,366	27,465,409	0	46,348,775
Sub-Acute	11,745	1,153	12,898	$6,465	$5,878	75,933,200	28,837,747	41,943,820	7,720,413	78,501,979
Non-Admitted	22,078	3,502	25,580	$6,465	$5,878	142,733,990	54,207,206	78,843,098	23,454,401	156,504,706
**Total ABF Allocation**	**159,511**	**6,362**	**165,873**			**1,031,238,678**	**391,641,595**	**569,633,431**	**42,613,209**	**1,003,888,235**
**Block Allocation**										
Teaching, Training, and Research						0	10,039,687	27,744,099	0	37,783,786

NHRA Funding Type	No. of In-scope services (NWAU)	No. of Out-of-scope services (QWAU)	Total Services (WAU)	ABF NEP ($)	State Price ($)	Funding for In-scope services at NEP ($)	Cwlth contribution for In-scope services ($)	State contribution for In-scope services ($)	State contribution for Out-of-scope services ($)	Total Cwlth and State contribution ($)
Small and Rural Hospitals[9]						0	17,129,594	42,492,493	0	59,622,088
Other Mental Health						0	21,088,180	35,489,301	0	56,577,481
Non-Admitted Home Ventilation						0	260,648	507,239	0	767,887
Other Non-Admitted Services						0	0	0	0	0
Other Public Hospital Programs						0	0	0	0	0
Highly Specialised Therapies						0	429,670	66,469	0	496,139
**Total Block Allocation**						**0**	**48,947,779**	**106,299,601**	**0**	**155,247,380**
**Grand Total Funding Allocation**										**1,159,135,615**

[9] Incorporating small regional and rural public hospitals, four specialist mental health facilities (Baillie Henderson Hospital, Jacaranda Place – Queensland Adolescent Extended Treatment Centre, The Park – Centre for Mental Health and Kirwan Rehabilitation Unit) and the Ellen Barron Family Centre.

Notes

a. QWAU refers to Queensland Weighted Activity Units in Q27 phase (built on N2425)
b. DVA, NIISQ/MAIC, Oral Health, Child Health Checks and BreastScreen
c. Queensland Efficient Price used to Purchase growth QWAUs
d. NWAU x NEP
e. State funding transacted through the Pool/State Managed Fund Account; not covered under the NHRA
- NWAU estimates do not take account of cross-border activity.

Table 7 Discretely Funded Programs (Non-ABF)

Discretely Funded Programs	Revenue Models	$
Aged Care Assessment Program	Commonwealth	$1,028,728
Alcohol, Tobacco and Other Drugs	State	$5,657,992
Community Health Programs	State	$48,697,700
Interstate Patients (QLD Residents)	State	$3,797,033
Other State Funding	State	$2,919,877
Patient Transport: PTSS	State	$7,492,866
Patient Transport: Aeromedical Retrieval	State	$2,216,232
Patient Transport	State	$0
Prevention Services and Public Health	Commonwealth	$10,387,812
	State	$735,294
Prisoner Primary Health Services	State	$12,694,308
	Capitation	$969,518
Disability Residential Care Services	State	$0
Torres Strait Treaty	Commonwealth	$0
Multi-Purpose Health Services	Commonwealth	$1,156,044
Residential Aged Care Services	Commonwealth	$426,929
	Locally Receipted Funds	$14,007,000
	State	$24,735,671
Transition Care	Locally Receipted Funds	$2,751,447
	State	$1,278,550
Research	Commonwealth	$196,804
	OSR	$0
	State	$0
Home and Community Care (HACC) Program	Locally Receipted Funds	$3,332,372
**Discretely Funded Programs Total**		**$144,482,176**
**TOTAL**		**$144,482,176**

Schedule 3 Department of Health Provided Services

1. In scope services and service schedules

Table 8 Department of Health provided services and service schedules

Provider	Service provided	Link to Service Statement
Corporate Services Division (CSD)	• Corporate Enterprise Solutions<br>• Finance Branch:<br>o Accounts Payable Service Provision<br>o Banking and Payment Services<br>• Central Pharmacy<br>• Group Linen Services<br>• Transport and Logistic Services<br>• Supply Chain Services	CSD Service Schedules
eHealth Queensland (eHQ)	• ICT Service	eHQ Service Schedule
Queensland Public Health and Scientific Services Division (QPHaSS)	• Pathology Queensland<br>• Biomedical Technical Services<br>• Public Health Services	QPHaSS Service Schedules

Schedule 4 Performance Measures

1. Performance Measures

1.1 The performance of the HHS will be measured according to the assessment criteria and processes described in the Performance and Accountability Framework.

1.2 Existing performance indicators are mapped to the care domains of the Outcomes Framework.

1.3 The detailed specification for each of the performance measures listed in this service agreement are provided through performance measure attribute sheets.

1.4 The performance measures identified in this service agreement are applicable to the HHS unless otherwise specified within the attribute sheet.

1.5 HHSs are also required to report against the agreed Statewide Health Equity Key Performance Measures (Table 12).

Table 9 HHS Performance Measures – Key Performance Indicators

Outcomes Framework Care Domain	Key Performance Indicators	Indicator Number
Chronic and complex	Hospital Acquired Complications (IHACPA code 8, 11, 13, 14)	31
Chronic and complex	Potentially Preventable Hospitalisations – First Nations Peoples:<br>• Diabetes complications<br>• Selected conditions	 37a<br>37b
Chronic and complex	Potentially avoidable deaths – First Nations Peoples	70
Maternity and neonates	Hospital Acquired Complications (IHACPA code 15,16)	31
Maternity and neonates	Proportion of healthy birthweight babies (2,500 grams to 4,499 grams at birth) – Aboriginal and Torres Strait Islander babies	71
Mental health, alcohol, and other drugs	Face to face community follow up within 1-7 days of discharge from an acute mental health inpatient unit	26
Mental health, alcohol, and other drugs	Proportion of mental health and alcohol and other drug service episodes with a documented care plan	27
Mental health, alcohol, and other drugs	Suicide count and rate – First Nations Peoples	72
Other	Average sustainable Queensland Health FTE	50
Other	Capital expenditure performance	51
Other	Forecast operating position:<br>• Full year<br>• Year to date	 48<br>49
Planned care	Category 1 elective surgery patients treated within the clinically recommended timeframe	7
Planned care	Elective surgery patients waiting longer than the clinically recommended timeframe	9
Planned care	Proportion of overnight inpatients discharged by 10am	12

Outcomes Framework Care Domain	Key Performance Indicators	Indicator Number
Planned care	Category 4 gastrointestinal endoscopy patients treated within the clinically recommended timeframe	13
Planned care	Gastrointestinal endoscopy patients waiting longer than the clinically recommended timeframe	16
Planned care	Category 1 patients who receive their initial specialist outpatient appointment within the clinically recommended timeframe	17
Planned care	Patients waiting longer than clinically recommended for their initial specialist outpatient appointment	19
Planned care	Telehealth utilisation rates:<br>Number of non-admitted telehealth service events	20
Planned care	Hospital Acquired Complications (IHACPA code 1,2,3,4,6,7,9,10,12)	31
Planned care	Missed Opportunity to Treat – Outpatients	73
Prevention, early intervention, and primary health care	Access to oral health services (adults)	21
Prevention, early intervention, and primary health care	Access to oral health services (children)	67
Prevention, early intervention, and primary health care	Potentially avoidable deaths – First Nations Peoples	70
Prevention, early intervention, and primary health care	Suicide count and rate – First Nations Peoples	72
Trauma and illness	Hospital Access Target (Admitted Patients)<br>• % of emergency stays within 4 hours	1
Trauma and illness	Hospital Access Target (All Patients)<br>• % of emergency stays within 4 hours	3
Trauma and illness	Emergency Department wait time by triage category	4
Trauma and illness	Emergency Department stays greater than 24 hours	5
Trauma and illness	Patient off stretcher time	6
Trauma and illness	Lost Minutes	61
Trauma and illness	Emergency Surgery patients treated in hours	62
Trauma and illness	Emergency Surgery patients treated in time	63
Trauma and illness	Transfer of care	69

Table 10 HHS Performance Measures - Safety and Quality Markers

Outcomes Framework Care Domain	Safety and Quality Markers	Indicator Number
Maternity and neonates	Sentinel Events	32
Planned care	Sentinel Events	32
Planned care	Hospital Standardised Mortality Ratio	33
Planned care	Severity Assessment Code (SAC1) analysis completion rates	34
Planned care	Healthcare-associated Staphylococcus Aureus (including MRSA) bacteraemia	35
Planned care	Patient Reported Experience	68

Table 11 HHS Performance Measures – Outcome Indicators

Outcomes Framework Care Domain	Outcome Indicators	Indicator Number
Chronic and complex	Potentially Preventable Hospitalisations (diabetes complications)	38
Chronic and complex	Potentially Preventable Hospitalisations (non-diabetes complications)	39
Chronic and complex	Advance care planning	43
Chronic and complex	Cardiac rehabilitation	44
Maternity and neonates	% of low birthweight babies born to Queensland mothers	41
Mental health, alcohol, and other drugs	Rate of seclusion events	28
Mental health, alcohol, and other drugs	Rate of absent without approval from acute mental health inpatient care	29
Mental health, alcohol, and other drugs	Smoking cessation clinical pathway	42
Other	First Nations peoples' representation in the workforce	47
Planned care	Complaints resolved within 35 calendar days	36
Planned care	Smoking cessation clinical pathway	42
Prevention, early intervention, and primary health care	General oral health care for First Nations peoples	22
Prevention, early intervention, and primary health care	The percentage of oral health activity which is preventive	23
Prevention, early intervention, and primary health care	Access to emergency dental care	24
Prevention, early intervention, and primary health care	Smoking cessation clinical pathway	42
Prevention, early intervention, and primary health care	Adolescent vaccinations administered via the statewide School Immunisation Program	45

Table 12 Statewide Health Equity Key Performance Measures

Outcomes Framework Care Domain	Key Performance Measures	Indicator Number
Chronic and complex	Advance care planning	43
Chronic and complex	Integrated care pathways - Rural and Remote HHSs:<br>• Care pathway in place for patients with identified co-morbidities	60
Maternity and neonates	Proportion of healthy birthweight babies (2,500 grams to 4,499 grams at birth) – Aboriginal and Torres Strait Islander babies	71
Mental health, alcohol, and other drugs	Face to face community follow up within 1-7 days of discharge from an acute mental health inpatient unit	26
Mental health, alcohol, and other drugs<br>Chronic and complex	Suicide count and rate – First Nations People	72
Other	First Nations peoples’ representation in the workforce	47
Planned care	Category 1 elective surgery patients treated within the clinically recommended timeframe	7
Planned care	Category 2 and 3 elective surgery patients treated within the clinically recommended timeframe	8
Planned care	Category 1 patients who receive their initial specialist outpatient appointment within the clinically recommended timeframe	17
Planned care	Category 2 and 3 patients who receive their initial specialist outpatient appointment within the clinically recommended timeframe	18
Prevention, early intervention, and primary health care	General oral health care for First Nations peoples	22
Prevention, early intervention, and primary health care	Potentially avoidable deaths – First Nations peoples	70

Schedule 5 Amendments to this service agreement

1. Agreed process to amend this service agreement

1.1 The parties acknowledge that this service agreement is subject to amendment, which will generally occur through:

(a) amendment windows;

(b) extraordinary amendment;

(c) periodic adjustments; and

(d) end of financial year reconciliation.

1.2 **Amendment windows**

(a) There will be set periods of time nominated by the Department during the year (amendment windows) in which a party may propose an amendment and the parties will endeavour to negotiate and finalise proposals to amend this service agreement.

(b) Amendment proposals that are agreed will be documented in a deed of amendment to this service agreement.

(c) Further details on the amendment window process, including the timing of amendment windows, is provided online as detailed in Appendix 1.

1.3 **Extraordinary Amendment**

(a) Outside an amendment window, the Department and the HHS agree to limit any proposal to amend the terms of this service agreement to those where there is an urgent priority need to facilitate a funding allocation (extraordinary amendment). The parties will endeavour to negotiate and finalise any such proposal urgently.

(b) The process for submitting, negotiating, and resolving an extraordinary amendment is available online as detailed at Appendix 1.

(c) Agreed extraordinary amendments will be reflected in a notice issued by the Chief Executive and countersigned as accepted by the HHS. The notice will be replaced when the extraordinary amendment is subsequently formalised in a deed of amendment issued following the next amendment window.

1.4 **Periodic adjustments**

(a) The service agreement value (and corresponding purchased activity) may be adjusted at any time to reflect funding variations that:

(i) occur on a periodic basis or in line with adjustments permitted for specific funding allocations;

(ii) are referenced in the service agreement; and

(iii) are based on a clearly articulated formula, an agreed basis or such other reasonably substantiated basis tied to performance.

(b) Periodic adjustments will be reflected in an adjustment notice issued by the Chief Executive (or delegate) to the HHS, based on relevant data, and subsequently formalised in a deed of amendment issued following the next amendment window.

1.5 End of financial year reconciliation

(a) There will be an end of financial year reconciliation process, with the scope defined by the Department and informed by Queensland Government Central Agency requirements.

(b) The Department will provide the HHS with a reconciliation of all service agreement funding and purchased activity for the prior financial year. This will reflect the position following conclusion of the end of financial year adjustments process.

(c) The impact of end of financial year adjustments on subsequent year funding and activity will be incorporated in the service agreement through the deed of amendment executed following the next available amendment window.

(d) This clause will survive expiration of this service agreement.

Appendix 1 Reference Documents

Service Agreement:

- Data supply requirements
- Delivery of Purchased Activity – Requirement for Quality Audits specification sheet
- Dispute resolution process - current
- First Nations First Strategy 2032
- Funding Outcomes Framework
- Hospital and Health Boards Act 2011
- Joint Regional Needs Assessment Framework
- Making Tracks Together: Queensland's Aboriginal and Torres Strait Islander Health Equity Framework
- Making Tracks toward closing the gap in health outcomes for Indigenous Queenslanders by 2033 - Policy and Accountability Framework
- National Agreement on Closing the Gap
- National Health Reform Agreement (NHRA) 2020-25
- Performance Measures Attribute Sheets
- Public Health Practice Manual
- Queensland Government Building Policy Framework and Guideline
- Queensland Health Performance and Accountability Framework
- Service agreement amendment processes
- Specifications supporting the Healthcare Purchasing Model
- Statewide services reference material

Supporting Policy documents

- Aboriginal and Torres Strait Islander Health Workforce Strategic Framework 2016-2026
- Department of Health Strategic Plan 2021-2025

- HEALTHQ32: A vision for Queensland's health system
- My health, Queensland's future: Advancing health 2026
- Queensland Health Equity, Diversity, and Inclusion Statement of Commitment
- System Outlook to 2026 - for a sustainable health service

Contracting and Performance Management Branch
GPO Box 48
Brisbane QLD 4001 Australia

Tel (+61) (07) 3708 5869

www.health.qld.gov.au
